# Mortality after paediatric emergency calls for patients with or without pre-existing comorbidity: a nationwide population based cohort study

**DOI:** 10.1186/s13049-024-01212-2

**Published:** 2024-05-28

**Authors:** Vibe Maria Laden Nielsen, Morten Breinholt Søvsø, Regitze Gyldenholm Skals, Lars Bender, Alasdair Ross Corfield, Hans Morten Lossius, Søren Mikkelsen, Erika Frischknecht Christensen

**Affiliations:** 1 Centre for Prehospital and Emergency Research, Department of Clinical Medicine, Aalborg University and Aalborg University Hospital, Selma Lagerløfs Vej 249, Gistrup, 9260 Denmark; 2 Emergency Medical Services, North Denmark Region, Hjulmagervej 20, Aalborg, 9000 Denmark; 3 Unit of Clinical Biostatistics, Aalborg University Hospital, Søndre Skovvej 15, Aalborg, 9000 Denmark; 4 Paediatric Department, Aalborg University Hospital, Reberbansgade 15, Aalborg, 9000 Denmark; 5 Emergency Department, Royal Alexandra Hospital, Paisley PA2 9PN, United Kingdom; 6 Norwegian Air Ambulance Foundation, Postboks 414 Sentrum Oslo 0103, Norway, United Kingdom; 7 The Prehospital Research Unit, Region of Southern Denmark, Odense University Hospital, , J. B. Winsløws Vej 4, Odense C 5000, Denmark

**Keywords:** Paediatric emergency medicine, Emergency medical services, Out-of-hospital cardiac arrest, Air ambulances, Epidemiology

## Abstract

**Background:**

Life-threatening conditions are infrequent in children. Current literature in paediatric prehospital research is centred around trauma and paediatric out-of-hospital cardiac arrests (POHCA). The aims of this study were to (1) outline the distribution of trauma, POHCA or other medical symptoms among survivors and non-survivors after paediatric emergency calls, and (2) to investigate these clinical presentations’ association with mortality in children with and without pre-existing comorbidity, respectively.

**Methods:**

Nationwide population-based cohort study including ground and helicopter emergency medical services in Denmark for six consecutive years (2016–2021). The study included all calls to the emergency number 1-1-2 regarding children ≤ 15 years (N = 121,230). Interhospital transfers were excluded, and 1,143 patients were lost to follow-up. Cox regressions were performed with trauma or medical symptoms as exposure and 7-day mortality as the outcome, stratified by ‘Comorbidity’, ‘Severe chronic comorbidity’ and ’None’ based on previous healthcare visits.

**Results:**

Mortality analysis included 76,956 unique patients (median age 5 (1–12) years). Annual all-cause mortality rate was 7 per 100,000 children ≤ 15 years. For non-survivors *without* any pre-existing comorbidity (*n* = 121), reasons for emergency calls were trauma 18.2%, POHCA 46.3% or other medical symptoms 28.9%, whereas the distribution among the 134 non-survivors *with* any comorbidity was 7.5%, 27.6% and 55.2%, respectively. Compared to trauma patients, age- and sex-adjusted hazard ratio for patients with calls regarding medical symptoms besides POHCA was 0.8 [0.4;1.3] for patients without comorbidity, 1.1 [0.5;2.2] for patients with comorbidity and 6.1 [0.8;44.7] for patients with severe chronic comorbidity.

**Conclusion:**

In both non-survivors with and without comorbidity, a considerable proportion of emergency calls had been made because of various medical symptoms, not because of trauma or POHCA. This outline of diagnoses and mortality following paediatric emergency calls can be used for directing paediatric in-service training in emergency medical services.

**Supplementary Information:**

The online version contains supplementary material available at 10.1186/s13049-024-01212-2.

## Introduction

Injuries are a significant cause of death among children, specifically motor vehicle crashes and intentional injuries [1–4], and traumatic brain injury is the leading cause of injury-related deaths [3, 5, 6]. In recent decades, advances in trauma care organisation and emergency medical services (EMS) have improved, leading to decreased trauma-related mortality in children [1, 5–10]. According to the United States Centers for Disease Control and Prevention, all cause mortality rate in children aged 1–19 years increased by almost 20% in the United States between 2019 and 2021 [11]. The increase predated the COVID-19 pandemic, and in the same period, the number of transport-related injury deaths decreased [11]. In the Scandinavian countries, childhood all cause mortality rate is around 23 deaths per year per 100,000 children aged up to 16 years, with a slight decline during the past decade [12–14]. Yet we do not know how many deaths are preceded by acute illnesses or injuries, owing to the paucity of large-scale epidemiological studies on outcomes after paediatric emergencies.

The objective of this study was to explore reasons for paediatric emergency calls in Denmark, i.e. trauma, paediatric out-of-hospital cardiac arrest (POHCA) or medical symptoms among survivors and 7-day non-survivors, and to investigate their association with mortality. In medical emergencies, pre-existing comorbidity may influence outcome. Consequently, outcomes are reported in predefined subgroups of children without any comorbidity, with comorbidity and with severe chronic comorbidity, respectively.

## Methods

### Study design and population

This was a nationwide population-based cohort study including all calls to the national emergency number (1-1-2) regarding patients aged ≤ 15 years followed by dispatch of either ground or helicopter EMS. The study period was 1 January 2016 to 31 December 2021 plus 30 days of follow-up. Interhospital transfers were excluded. Reporting of the study follows the ‘Strengthening the Reporting of Observational Studies in Epidemiology’ guidelines [15]. 

### Setting

The Danish EMS serve 5,806,081 inhabitants in mixed rural, semi-rural, and urban areas (ground area 42,962 km^2^). Children aged ≤ 15 years comprised 18% of the population amid the study period (per 1 January 2019) [16]. Emergency medical dispatch centres are staffed by health professionals who manage the dispatch of both ground and helicopter EMS units according to the level of urgency (A-E, A = lights and sirens, E = telephone counselling only). All dispatch centres use the criteria based decision support tool *Danish Index for Emergency Care* [17]. The health professionals who answer emergency calls use the index to question the caller to decide on an appropriate main reason for the call and urgency level (i.e. dispatch criterion), which then trigger a certain response. For each call, the patient can only be labelled with one criterion. Ambulances and rapid response vehicles, staffed by emergency medical technicians and paramedics, can be assisted by 24-hour operative physician-staffed mobile emergency care units and/or physician-staffed helicopter EMS. Generally, a physician-staffed unit is engaged in about 20–25% of ambulance dispatches. Further details of the organisation of the prehospital system in Denmark have been described in Supplementary Material 1 and in previous works [18]. 

### Data sources and linkage

All calls to the national emergency number (1-1-2) regarding patients aged ≤ 15 years were collected from. The Danish Quality Database for Prehospital Emergency Medical Services under *The Danish Clinical Quality Program – National Clinical Registries*. Reporting to the registry is mandatory for all Danish EMS [19]. The Danish Civil Registration System contains a personal identification number (PIN) for all residents in Denmark and holds information on migration and vital status [20]. The Danish National Patient Register [21] contains data from all inpatient and outpatient hospital visits and visits to private practitioners in all medical specialities, including ICD-10 diagnoses [22]. Registries were linked using PINs and timestamps of the first ambulance that had arrived at a hospital. For details about the record linkage process, we refer to Supplementary Material 1.

Patients without a valid PIN could not be identified as children of a certain age and were therefore not eligible for the study. Patients with missing PINs might introduce a selection bias, as registering a PIN on the medical record might not be prioritised in critical emergencies. Therefore, we crosschecked mortality with selected variables from prehospital electronic patient medical records for all patients regardless of whether a PIN was present (specific variables are designated in Table 1). Consequently, we were able to investigate the direction and magnitude of this possible selection bias in our mortality analysis.

**Table 1:** Characteristics of children for whom an emergency call had been made in all of Denmark

	Mission level *N* = 93,081		Individual patient level *N* = 76,956	
n, %				
**Order of missions with a unique patient**				
First	72,098	77.5	*NA*	
Second	11,750	12.6		
Third or more	9,233	9.9		
Age*				
Median (IQR)	5	(1–12)	5	(1–12)
0–2 months	3,315	3.6	3,102	4.0
3–11 months	8,556	9.2	6,834	8.9
1–2 years	24,375	26.2	17,943	23.3
3–7 years	18,570	20.0	15,281	19.9
8–15 years	38,265	41.1	33,796	43.9
**Sex**				
Female	42,445	45.6	35,522	46.2
Male	50,636	54.4	41,434	53.8
**Comorbidity**				
None	53,772	57.8	47,563	61.8
Comorbidity (previous or chronic diseases, conditions, or perinatal complications)	35,913	38.6	27,778	36.1
Severe chronic comorbidity (with potentially considerably reduced lifespan)	3,396	3.7	1,615	2.1
**Urgency level**				
A (lights and sirens)	55,209	59.3	*NA*	
B	28,150	30.2		
C-E	5,074	5.5		
Missing data	4,648	5.0		
**Physician dispatched**†				
HEMS physician	863	0.9	*NA*	
MECU physician	20,342	21.9		
None	71,876	77.2		
**Time from emergency call to arrival at hospital**				
< 15 min	205	0.2	*NA*	
15–30 min	8,855	9.5		
30–45 min	22,592	24.3		
45–60 min	23,284	25.0		
1–2 h	23,216	24.9		
> 2 h	612	0.7		
Missing data	14,317	15.4		

No missing data unless otherwise indicated. A unique patient could have had more than one emergency call during the study period, and the table columns display data at both mission level and individual patient level

*HEMS* helicopter emergency medical services; *IQR* interquartile range; *MECU* mobile emergency care unit

*In the ‘Individual patient level’ column, the age reported is the child’s age at the first emergency call

†An event only counts once, even if more than one physician was dispatched (HEMS overrule MECU)

### Exposures and outcomes

Exposures were reasons for emergency call and the outcome was 7-day mortality, and outcomes were presented stratified by comorbidity subgroups. Dispatch criteria were used to define reasons for emergency call. Criteria were grouped into:

1)‘Trauma’ if criteria were:

Large scale accident

Fire or electrical injury

Drowning

Diving accident

Hypothermia – Hyperthermia

Traffic accident

Accidents (not traffic-related)

Minor wounds – fractures – injuries

Violence – abuse

2) ‘Paediatric out-of-hospital cardiac arrest (POHCA)’ if criteria were:

Unconscious adult (after puberty) or Unconscious child (before puberty)

Plus

The patient had received cardiopulmonary resuscitation from either a bystander or from healthcare professionals outside of a hospital according to current reporting standards [23, 24]. 

3) ‘Suspected death’ (a specific dispatch criterion that describes the ‘finding of a lifeless person’).

4) ‘Medical symptoms’ if criteria were any of the remaining criteria in the *Danish Index for Emergency*:

*Care* [17]: ‘Allergic reaction’, ‘Fever’, ‘Poisoning in children’, ‘Headache’, ‘Breathing difficulties’, ‘Psychiatry – suicidal’, ‘Abdominal pain – back pain’, ‘Seizure’, ‘Sick child’, ‘Foreign body in airway’, ‘Ordered mission’, ‘Unclear problem’, ‘Bleeding – non-traumatic’, ‘Chest pain – heart disease’, ‘Diabetes’, ‘Animal and insect bites’, ‘Childbirth’, ‘Gynaecology – pregnancy’, ‘Skin – rash’, ‘Chemicals – gases’, ‘Impaired consciousness – paralysis – vertigo’, ‘Alcohol – poisoning – overdose’, ‘Urinary system’, ‘Ear – nose – throat’ or ‘Eye’.

5) ‘Missing criteria’.

The association between the reasons for emergency call and the outcome 7-day mortality was stratified by comorbidity subgroups. The subgroups were formed based on clinical experience, previous literature [25, 26] and publicly available information on the expected prognosis for each of the diseases and conditions in the ICD-10 classification system. An example of publicly available information was an international web portal for rare diseases, www.orpha.net.First we downloaded a list of all ICD-10 diagnoses and then manually reviewed each one and put them into one of three categories: ‘Comorbidity’ (previous or chronic disease, condition, or perinatal complication), ‘Severe chronic comorbidity’ (diseases or conditions with potentially considerably reduced lifespan) or ‘None’. If patients had not had any visits in those five years, i.e. no previous diagnoses, they were classified as comorbidity subgroup ‘None’.

We then searched all inpatient and outpatient hospital visits and visits to private practitioners from the five years preceding each emergency call for all of the unique patients.

The average number of visits was calculated to underline disease severity in these predefined subgroups. Patients with ‘Severe chronic comorbidity’ had had median (IQR) 88 (45–164) visits in the five years preceding their last emergency call while patients in the ‘Comorbidity’ subgroup had had 16 (9–31) visits.

### Statistics

Summary statistics were used for the grouped exposure, reasons for emergency call. For mortality analysis, patients were considered at risk from the date of emergency call until either death or the end of follow-up. Patients were lost to follow-up if they had no current address in Denmark, PIN was annulled, patient disappeared or travelled abroad as indicated in the Civil Registration System [20]. No data were imputed. All cause mortality estimates were calculated using last-time events only by modified Poisson regression with robust variance estimation [27].

A unique patient could have had more than one emergency call during the study period. These ‘repeat calls’ could influence the mortality analyses, which is why we performed sensitivity analyses using first-time events only and all events, respectively. This means that mortality is reported both at a mission level, i.e. the denominator was number of emergency calls (= missions), and at an individual patient level, i.e. the denominator was unique patients.

In a Cox proportional hazards model with time since inclusion as time scale, we estimated 7-day mortality between the dispatch criteria categories mentioned above in each of the three comorbidity subgroups. Assumptions of proportional hazards were assessed visually by a log-minus-log plot and using Schoenfeld residuals. Hazard rate ratios were reported as crude and adjusted for sex (dichotomous, no missing data) and age (categorical, no missing data) with ‘Trauma’ as reference. Age was grouped according to the Danish Regions’ Paediatric Triage Model used by all of the Danish EMS. Two-sided p values < 0.05 were considered statistically significant. Statistical analyses were performed with Stata/MP 17.0 (*StataCorp LLC, TX 77845, USA*).

## Results

### Characteristics of the study population

**Fig. 1:**
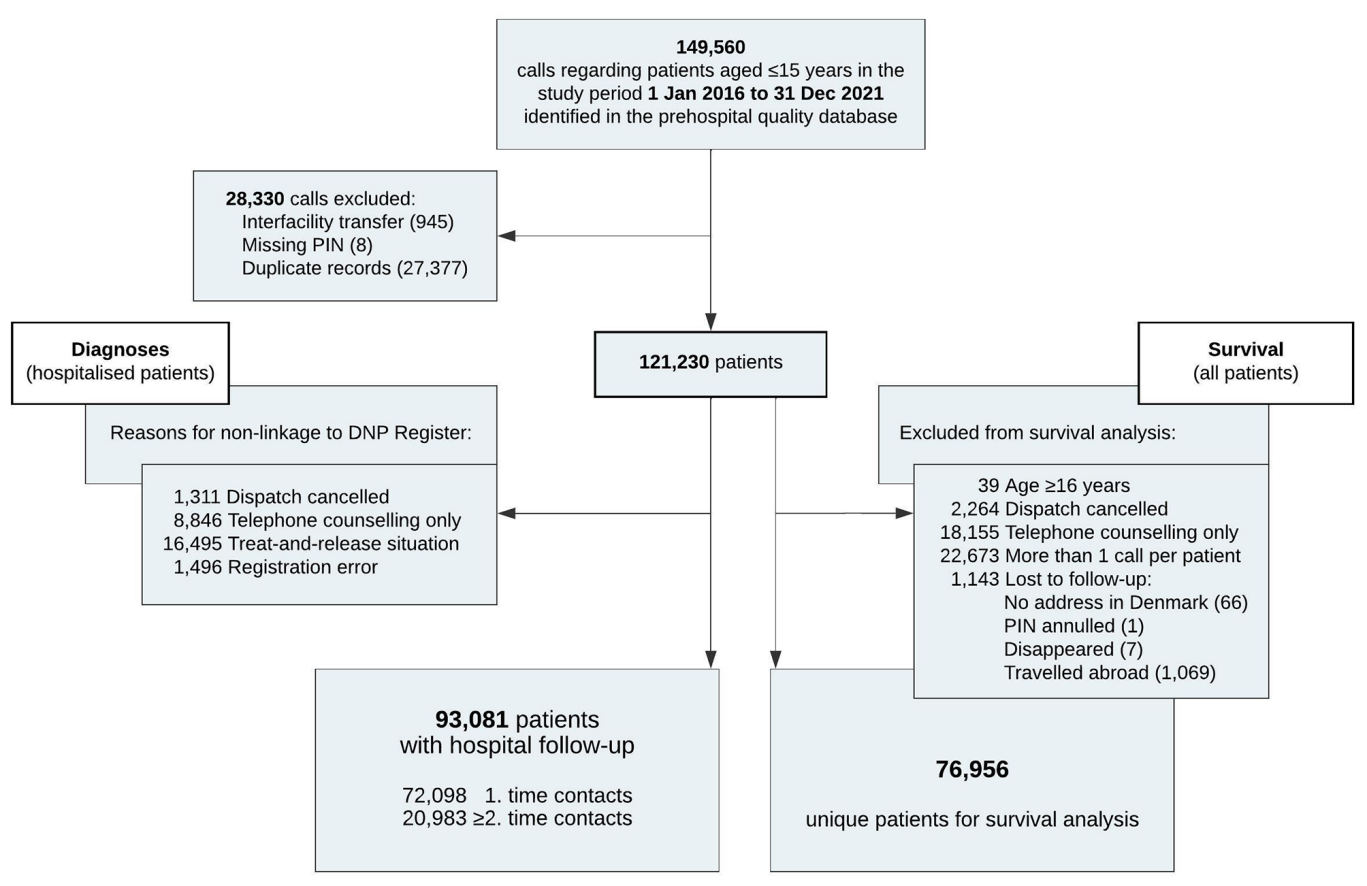
Patient flow chart. A unique patient could have had more than one patient contact during the study period. DNP: Danish National Patient (Register); PIN: personal identification number

The patient flow and reasons for non-linkage to the registries are depicted in Fig. 1. Characteristics of the study population are summarised in Table 2. In addition, primary hospital diagnoses after the dispatch of an ambulance or a helicopter are supplied in Supplementary Material 1, Table S1. Overall, the majority of paediatric emergency calls regarded medical symptoms (67.3%), while almost a third of the calls regarded trauma (27.2%) and few calls regarded POHCAs (0.2%), suspected deaths (< 0.1%) or had missing data (5.3%).

### All cause mortality

**Fig. 2:**
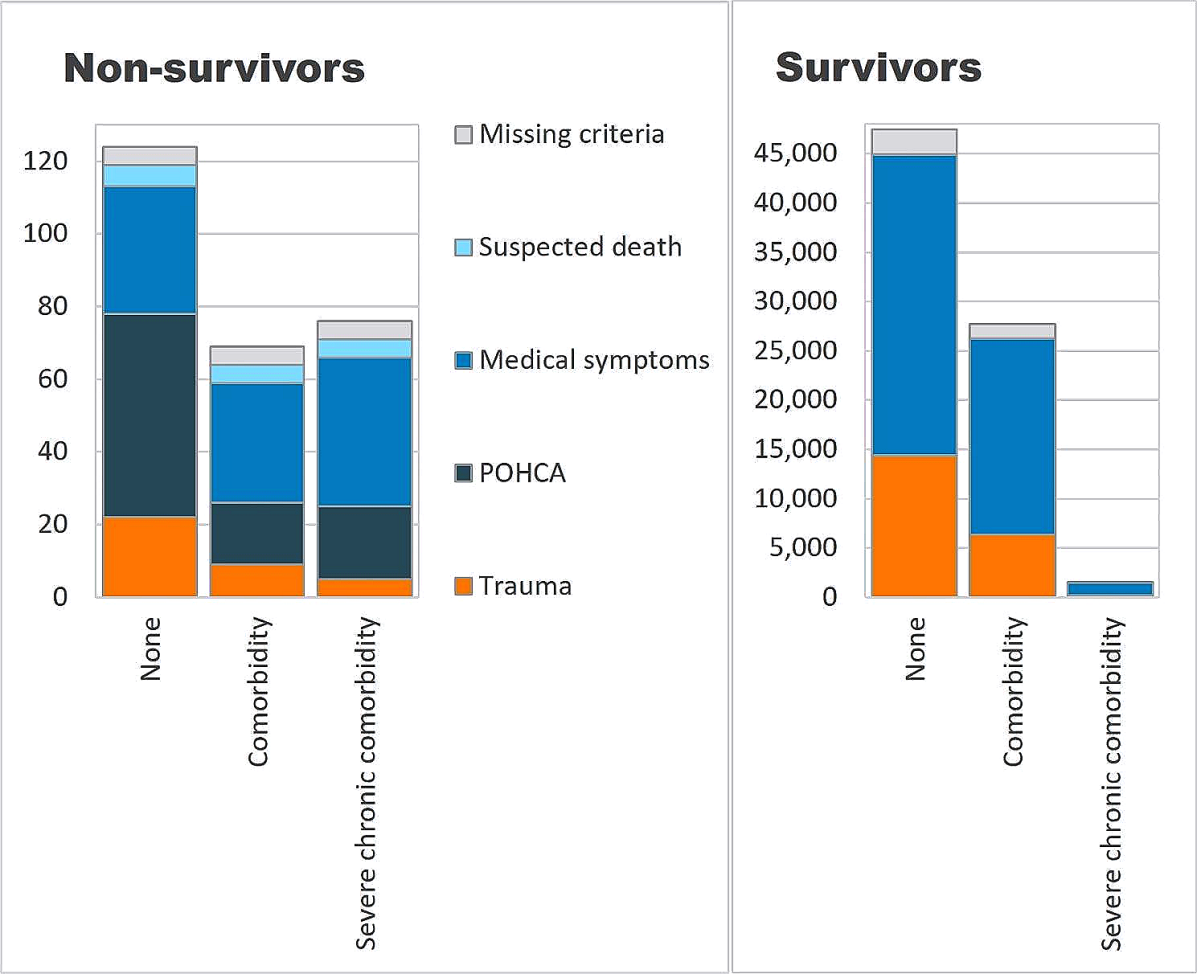
Reasons for emergency call stratified by comorbidity subgroup: ‘None’ (*N* = 47,563), ‘Comorbidity’ (*N* = 27,778), and ‘Severe chronic comorbidity’ (*N* = 1,615). Survivors (*n* = 255) and non-survivors (*n* = 76,701) refer to death within 7 days of the last emergency call. Frequency data are supplied in Supplementary Material 1, Table S2. POHCA: Paediatric out-of-hospital cardiac arrest

The annual all cause mortality rate after a paediatric emergency call was 7.0 per 100,000 children ≤ 15 years (calculated using last-time events only: 428 deaths with a population at risk of 1,023,297). Figure 2 displays reasons for emergency call in survivors and non-survivors within the three comorbidity subgroups (numerical data are supplied in Supplementary Material 1, Table S2).

**Table 2:** Last ICD-10 diagnosis assigned by a physician to patients who died within 7 days of their last emergency call. *n* = 255

No. (%)		*P* value†
**ICD-10 Chapter**		
Sudden cardiac death (I46.1)	40 (15.7)	< 0.001
Injury, poisoning, and other external causes (ST)	35 (13.7)	
Cardiac arrest (I46.0/9)	34 (13.3)	
Congenital disease or perinatal complication (PQ)	30 (11.8)	
Contact with health services (Z)	19 (7.5)	
Other ill-defined and unspecified causes of mortality (R99)	17 (6.7)	
Missing data	17 (6.7)	
Nervous system diseases (G)	16 (6.3)	
Infection (AB)	15 (5.9)	
Cancer (CD)	15 (5.9)	
Respiratory disease or respiratory failure (J)	9 (3.5)	
Other disease, condition, or complication*	8 (3.1)	

*ICD-10* International Statistical Classification of Diseases and Related Health Problems, Tenth Revision

*Other diseases, conditions, or complications that could not be added to any of the groups above

†Calculated by 𝜒^2^ test

### Reasons for emergency call among non-survivors

A quarter of the of the non-survivors (70/255 = 27.5%) had ‘Severe chronic’ conditions. In most non-survivors *without* severe chronic comorbidity, the emergency call had been made because of various medical symptoms or POHCA, and not because of trauma (50/64 = 78.1% [66.0;87.5] in the ‘Comorbidity’ group and 91/121 = 75.2% [66.5;82.6] in group ‘None’, respectively).

**Fig. 3:**
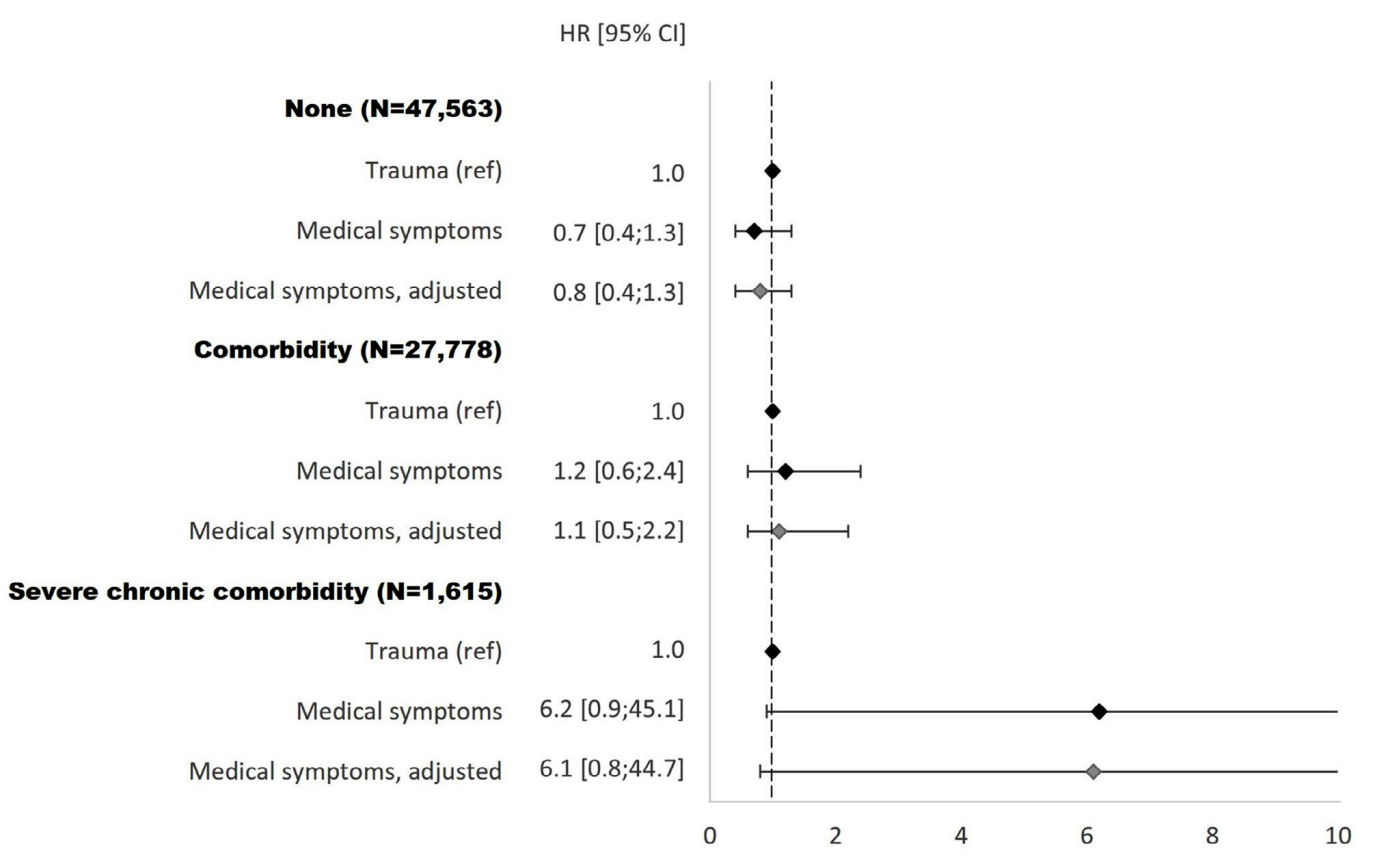
Hazard ratios for 7-day mortality after a patient’s last emergency call according to reasons for calling, stratified by comorbidity subgroup. ‘Medical symptoms’ does not include calls regarding paediatric out-of-hospital cardiac arrest. Crude HRs (black) and adjusted for age and sex (grey). HR: hazard ratio; CI: confidence interval; ref: reference

Patients with emergency calls regarding medical symptoms besides POHCA had a 7-day mortality that was not significantly different from patients with calls regarding trauma (Fig. 3).

The last diagnoses preceding death for the 255 children who did not survive 7 days after the emergency call are supplied in Table 3 (POHCA 29.0% and trauma 13.7%).

**Table 3:** Mortality estimates from Poisson regression on individual patient level versus mission level

		Original dataset with PINs			Dataset with or without PINs
		First-time missions (patient level)	Last-time missions (patient level)	All missions (mission level)	All missions (mission level*)
	N	76,956	76,956	99,281	101,094
**1-day**	Deaths (no.)	175	206	223	273
	Risk (%)	0.23	0.27	0.22	0.27
	95% CI	0.20;0.26	0.23;0.31	0.20;0.26	0.24;0.30
**7-day**	Deaths (no.)	218	255	276	NA
	Risk (%)	0.28	0.33	0.28	
	95% CI	0.25;0.32	0.29;0.37	0.25;0.31	
**30-day**	Deaths (no.)	231	282	318	NA
	Risk (%)	0.30	0.37	0.32	
	95% CI	0.26;0.34	0.33;0.41	0.29;0.36	

*PIN* Personal identification number

*Data contained both patients with and without valid personal identification numbers retrieved from out-of-hospital electronic medical records. From here we identified out-of-hospital deaths (comparable to 1-day mortality) by the following definition: either (1) the patient had been ‘declared dead on scene’ by a prehospital physician (i.e. the patient had certain signs of death when the EMS arrived) or (2) the patient had a Glasgow Coma Score of 3 *plus* any of the variables that are transferred into the Danish Cardiac Arrest Registry [24].

### Comorbidity

Almost half of the cohort had pre-existing comorbidity or severe chronic comorbidity (Table 1). Frequent diseases or conditions in the ‘Comorbidity’ group were: Asthma (3.7% of the entire EMS cohort, *N* = 76,956), epilepsy (2.9%), prematurity/ immaturity (2.8%), neonatal jaundice (1.7%), diabetes (0.8%) and cardiovascular diseases (1.1%) (for details, we refer to Supplementary Material 2). Frequent ‘Severe chronic’ diseases were: Cerebral palsy/hemiplegia/paraplegia/tetraplegia (0.8%), advanced congenital cardiovascular malformations (0.3%) and malignant neoplasms (0.2%).

### Sensitivity analyses

Similar mortality was found when using first-time events only and all events, respectively (Table 3). We also checked for independent censoring (Supplementary Material 1, Table S3).

## Discussion

### Principal findings

Across all ages, injuries comprised 38% of the primary hospital diagnoses, while only 14% of 7-day non-survivors had *Injury, poisoning and certain other consequences of external causes* as their last ICD-10 diagnosis preceding death. The study findings indicate that emergency calls regarding medical symptoms besides POHCA were as critical as calls regarding trauma, as there was no significant difference in 7-day mortality in either of the comorbidity subgroups. A tendency towards higher 7-day mortality from ‘Medical symptoms’ in the ‘Severe chronic comorbidity’ group was observed, though.

### Comparison with other studies

The few previous studies on clinical outcomes for unreferred paediatric EMS populations have reported short term mortality rates comparable to ours (30-day mortality of 0.15–1.60% versus 0.37% in our study) [28–31]. In children, non-cardiac causes of death such as trauma, respiratory insufficiency and shock are predominant [1, 4, 32, 33]. The patients with medical symptoms (chest pain, stomach pain, etc.) could have suffered a cardiac arrest before arrival at a hospital, though they were not in arrest at the time the emergency call was made, which is why they were not regarded as such. Some patients may have been assigned the ‘Unconscious adult/child’ dispatch criterion even though the call regarded a traumatic POHCA, which would cause non-traumatic POHCAs to be overestimated in our study. However, when we manually reviewed the last diagnoses preceding death and let cardiac arrest be ‘overruled’ by the second to last diagnosis (e.g. in case of drowning as described in Supplementary Material 1), the proportion of deaths attributable to non-traumatic POHCA was also around one third (36.5% using dispatch criteria versus 29.0% using ICD-10 diagnoses). Due to the risk of disclosing personally identifiable data, individual ICD-10 diagnostic codes for deceased patients were not possible to report.

Few and conflicting studies have reported the occurrence of comorbidity among paediatric emergency patients. In a systematic overview of 37 studies, only 6% had ‘Chronic disease’ marked as the cause of non-submersion POHCA [34]. In a 10-year single-centre study from Finland, 65% of POHCA patients had ‘suspected or diagnosed chronic or otherwise significant conditions’ [35]. Among Canadian atraumatic POHCA patients, 16.2% of those who died before hospital discharge had had a ‘significant comorbidity’ [36]. 

In our study, this proportion was markedly higher (52.6%). We manually reviewed all ICD-10 codes to distinguish between chronic and severe chronic comorbidities that had a potentially considerably reduced lifespan. The Canadian study by Tijssen et al. only concerned POHCA patients, while ours included all types of emergencies [36].

### Clinical implications and future research

Caring for paediatric patients may create a sense of stress in EMS professionals, and some studies relate this stress to inadequate training in clinical judgement and treatment of children [37, 38]. The sensitivity of triage systems is lower for children with chronic diseases which increases the risk of ‘undertriage’ in these children [39]. Our systematic outline of reasons for emergency call among survivors and 7-day non-survivors, respectively, may be used to plan further educational efforts in Danish EMS. While established trauma protocols have been fully implemented years ago, our results indicate that these efforts may be extended to include in-service training in the management of exacerbations of pre-existing diseases or conditions for the benefit of improving outcomes.

### Limitations and generalisability

The Feudtner system has been used in previous research to report comorbidity/medical complexity. However, it was not directly transferable to present-day Danish settings. Instead, prior in- and out-patient hospital visits or visits to private practitioners were used for dividing patients into comorbidity subgroups. In Denmark, differential diagnoses like diabetes or epilepsy would ordinarily lead to a referral to either a paediatric department (outpatient clinic) or a private practitioner and then followed up by their general practitioner. Some childhood psychiatric diseases may influence mortality [35, 40]. Not all visits to psychiatric facilities are contained in the Danish National Patient Register, however, which hindered the use of two previously validated paediatric comorbidity indexes [41, 42].

Dispatch criterion 27 ‘Psychiatry – suicidal’ is a diverse criterion with symptoms ranging from suicide attempts to a mere suspicion of mental illness. The 19 patients with this criterion were included in ‘Medical symptoms’ because self-injury might be preceded by mental health issues (i.e. medical symptoms).

The study was based on a consecutive and comprehensive data collection from national registries during both pre- and peri-COVID-19 periods. Nonetheless, mortality estimates may not be generalisable to all healthcare systems. The novel methodology of reporting paediatric comorbidity using prior hospital or private practitioner visits (and ICD-10 diagnosis codes) can be applied to research in any healthcare system with nationwide health administrative registries. The patients lost to follow-up did not affect the main outcome of interest, mortality. In Table 3, we presented similar 1-day mortality calculated for patients with known PINs only and for patients with or without known PINs, respectively (i.e. the patients who were lost to follow-up).

## Conclusions

In this nationwide cohort of paediatric EMS patients, medical symptoms besides paediatric out-of-hospital cardiac arrest accounted for almost half of the early deaths from paediatric emergencies. 7-day mortality risk was not significantly different in children with medical symptoms besides POHCA compared to trauma, neither in children with or without pre-existing comorbidity. Among children with ‘Severe chronic comorbidity’, a tendency towards higher mortality from ‘Medical symptoms’ was observed, though. Following the past decades’ advancement in trauma care, EMS should set aside time and resources for scenario training on the initial management of paediatric illnesses and commonly occurring comorbidities.

### Electronic supplementary material

Below is the link to the electronic supplementary material.

Supplementary Material 1

Supplementary Material 2

## Data Availability

Legal restrictions apply to the availability of the dataset, and therefore data are not publicly available. Data are available from the authors upon reasonable request and with permission of the Danish Health Data Authority.

## List of abbreviations

COVID-19: Coronavirus disease 2019
EMS: Emergency medical services
ICD-10: International Statistical Classification of Diseases and Related Health Problems, Tenth Revision
PIN: Personal identification number
POHCA: Paediatric out-of-hospital cardiac arrest
