# Supplementary material for: Mortality after paediatric emergency calls for patients with or without pre-existing comorbidity: a nationwide population based cohort study
Source: Scand J Trauma Resusc Emerg Med. 2024 May 28;32:48. doi: 10.1186/s13049-024-01212-2 (PMC11134704; doi:10.1186/s13049-024-01212-2)
Supplement: Supplementary file 1 — Supplementary Material 1 [file 13049_2024_1212_MOESM1_ESM.pdf]

Supplement 2 Nielsen et al. Mortality after paediatric emergency calls for patients with and without pre-existing comorbidity (2023)

Copyright The Danish Health Data Authority. All classifications maintained and published by the Danish Health Data Authority are publicly available and can be freely used in various IT-solutions etc. However, it is a prerequisite that the classifications themselves are not subjected to commercial use. In addition, there is a requirement that classifications used in various user solutions are updated at all times and thus in accordance with the latest versions of the classifications published by the Danish Health Data Authority. The SKS-dumper (version 1.06) is updated regularly, and this output corresponds to the update of 31 December 2022 15:47:23.

D;Classification of Diseases and Related Health Problems	None	Previous	Chronic
DA00;Kolera	0		
DA000;Kolera forårsaget af Vibrio cholerae	0		
DA001;Kolera forårsaget af Vibrio cholerae eltor	0		
DA009;Kolera UNS	0		
DA01;Tyfus og paratyfus	0		
DA010;Tyfus	0		
DA011;Paratyfus A	0		
DA012;Paratyfus B	0		
DA013;Paratyfus C	0		
DA014;Paratyfus UNS	0		
DA02;Andre salmonellainfektioner	0		
DA020;Salmonellaenterit	0		
DA021;Salmonellasepsis	0		
DA022;Lokaliseret salmonellainfektion	0		
DA028;Anden salmonellainfektion	0		
DA029;Salmonellainfektion UNS	0		
DA03;Bacillær dysenteri	0		
DA030;Dysenteri forårsaget af Shigella dysenteriae	0		
DA031;Dysenteri forårsaget af Shigella flexneri	0		
DA032;Dysenteri forårsaget af Shigella boydii	0		
DA033;Dysenteri forårsaget af Shigella sonnei	0		
DA038;Anden bacillær dysenteri	0		
DA039;Bacillær dysenteri UNS	0		
DA04;Andre bakterielle tarminfektioner	0		

Supplement 2 Nielsen et al. Mortality after paediatric emergency calls for patients with and without pre-existing comorbidity (2023)

DA040;Enteritis forårsaget af enteropatogen Escherichia coli-infektion	0		
DA041;Enteritis forårsaget af enterotoksisk Escherichia coli-infektion	0		
DA042;Enteritis forårsaget af enteroinvasiv Escherichia coli-infektion	0		
DA043;Enteritis forårsaget af enterohæmoragisk Escherichia coli-infektion	0		
DA044;Anden tarminfektion med Escherichia coli	0		
DA045;Enteritis forårsaget af Campylobacter	0		
DA046;Enteritis forårsaget af Yersinia enterocolitica	0		
DA047;Enterokolitis forårsaget af Clostridium difficile	0		
DA048;Anden bakteriel enteritis	0		
DA049;Enteritis forårsaget af bakterier UNS	0		
DA05;Andre bakterielle fødevareforgiftninger IKA	0		
DA050;Fødevareforgiftning forårsaget af stafylokokker	0		
DA051;Botulisme	0		
DA052;Fødevareforgiftning forårsaget af Clostridium perfringens	0		
DA053;Fødevareforgiftning forårsaget af Vibrio parahaemolyticus	0		
DA054;Fødevareforgiftning forårsaget af Bacillus cereus	0		
DA058;Anden bakteriel fødevareforgiftning	0		
DA059;Bakteriel fødevareforgiftning UNS	0		
DA06;Amøbedysenteri	0		
DA060;Akut amøbedysenteri	0		
DA061;Kronisk intestinal amøbeinfektion	0		
DA062;Colitis forårsaget af amøbeinfektion	0		
DA063;Amøbom i tarmen	0		
DA063A;Amøbom UNS	0		
DA064;Leverabsces forårsaget af amøbeinfektion	0		
DA065;Lungeabsces forårsaget af amøbeinfektion	0		
DA066;Hjerneabsces forårsaget af amøbeinfektion	0		
DA067;Amøbeinfektion i huden	0		
DA068;Amøbeinfektion med anden lokalisation	0		
DA068A;Appendicitis forårsaget af amøbeinfektion	0		
DA069;Amøbeinfektion UNS	0		
DA07;Andre tarmsygdomme fremkaldt af protozoer	0		

DA070;Balantidiasis	0			
DA071;Giardiasis	0			
DA072;Cryptosporidiosis	0			
DA073;Isosporiasis	0			
DA078;Anden tarminfektion forårsaget af protozoer	0			
DA078A;Sarcocystose	0			
DA078C;Trichomoniasis intestinalis	0			
DA078D;Coccidiosis intestinalis	0			
DA079;Tarmsygdom forårsaget af protozoer UNS	0			
DA08;Tarminfektioner forårsaget af virus og andre organismer	0			
DA080;Enteritis forårsaget af rotavirus	0			
DA081;Akut gastroenteritis forårsaget af Norwalk-virus	0			
DA082;Enteritis forårsaget af adenovirus	0			
DA083;Enteritis forårsaget af anden virus	0			
DA083A;Gastroenteritis forårsaget af parvovirus	0			
DA084;Tarminfektion forårsaget af virus UNS	0			
DA085;Tarminfektion forårsaget af anden (mikro)organisme UNS	0			
DA09;Anden gastroenteritis og colitis af infektiøs eller ikke specificeret årsag	0			
DA090;Anden og uspecifik gastroenteritis og colitis af infektiøs oprindelse	0			
DA099;Gastroenteritis eller colitis af ikke specificeret årsag	0			
DA15;Tuberkulose i åndedrætsorganer verificeret bakteriologisk og histologisk	0			
DA150;Lungetuberkulose verificeret ved mikroskopi af ekspektorat med eller uden dyrkning	0			
DA151;Lungetuberkulose verificeret alene ved dyrkning	0			
DA152;Lungetuberkulose verificeret histologisk	0			
DA153;Lungetuberkulose verificeret ved ikke angivet metode	0			
DA154;Tuberkulose i intratorakale lymfeknuder verificeret bakteriologisk eller histologisk	0			
DA155;Tuberkulose i struben, luftrøret eller bronkier verificeret bakteriologisk eller histologisk	0			
DA156;Tuberkuløs lungehindebetændelse verificeret bakteriologisk eller histologisk	0			
DA157;Primær tuberkulose i åndedrætsorganerne verificeret bakteriologisk eller histologisk	0			
DA158;Anden tuberkulose i åndedrætsorganerne verificeret bakteriologisk og histologisk	0			
DA158A;Tuberkulose i mediastinum verificeret bakteriologisk og histologisk	0			
DA158B;Tuberkulose i næsen verificeret bakteriologisk og histologisk	0			

DA158C;Tuberkulose i næsesvælget verificeret bakteriologisk og histologisk	0			
DA159;Tuberkulose i åndedrætsorganerne UNS, verificeret bakteriologisk eller histologisk	0			
DA16;Tuberkulose i åndedrætsorganer ikke bakteriologisk eller histologisk verificeret	0			
DA160;Lungetuberkulose, bakteriologisk og histologisk negativt fund	0			
DA161;Lungetuberkulose, bakteriologisk og histologisk undersøgelse ikke er foretaget	0			
DA162;Lungetuberkulose uden angivelse af bakteriologisk eller histologisk verifikation	0			
DA162A;Tuberkuløs lungefibrose UNS	0			
DA162B;Tuberkuløs pneumoni UNS	0			
DA162C;Tuberkuløs pneumothorax UNS	0			
DA163;Tuberkulose i intratorakale lymfekirtler uden angivelse af bakteriologisk eller histologisk ve	0			
DA164;Tuberkulose i struben, luftrøret eller bronkier uden angivelse af bakteriologisk eller histolog	0			
DA165;Tuberkuløs lungehindebetændelse uden angivelse af bakteriologisk eller histologisk verifik	0			
DA167;Primær tuberkulose i åndedrætsorganerne uden angivelse af bakteriologisk eller histologis	0			
DA168;Anden tuberkulose i åndedrætsorganerne uden angivelse af bakteriologisk eller histologisk	0			
DA168A;Tuberkulose i mediastinum UNS	0			
DA168B;Tuberkulose i næsen UNS	0			
DA168C;Tuberkulose i næsesvælget UNS	0			
DA169;Tuberkulose i åndedrætsorganerne UNS, uden angivelse af bakteriologisk eller histologisk	0			
DA17;Tuberkulose i nervesystemet	0			
DA170;Tuberkuløs meningitis	0			
DA171;Tuberkulom i hjernehinder	0			
DA178;Anden form for tuberkulose i nervesystemet	0			
DA179;Tuberkulose i nervesystemet UNS	0			
DA18;Tuberkulose i andre organer	0			
DA180;Tuberkulose i knogler og led	0			
DA181;Tuberkulose i urinveje og kønsorganer	0			
DA182;Tuberkulose i perifere lymfeknuder	0			
DA183;Tuberkulose i tarm, bughinde og mesenteriale lymfeknuder	0			
DA183F;Tuberkulose i retroperitoneale lymfeknuder	0			
DA183G;Tuberkuløs ascites	0			
DA183H;Tuberkulose i mesenteriale lymfeknuder	0			
DA184;Tuberkulose i hud og underhud	0			

Supplement 2 Nielsen et al. Mortality after paediatric emergency calls for patients with and without pre-existing comorbidity (2023)

DA184A;Lupus	0			
DA184B;Lupus excedens	0			
DA184C;Lupus vulgaris	0			
DA184D;Scrofuloderma	0			
DA184F;Tuberculosis lichenoides	0			
DA184G;Tuberculosis subcutis	0			
DA184H;Tuberculosis verrucosa cutis	0			
DA184I;Erythema induratum Bazin	0			
DA184J;Lichen scrofulosorum	0			
DA185;Tuberkulose i øje	0			
DA186;Tuberkulose i øre	0			
DA187;Tuberkulose i binyre	0			
DA187A;Tuberkuløs Addisons sygdom	0			
DA188;Tuberkulose i andet organ	0			
DA19;Miliær tuberkulose	0			
DA190;Akut miliær tuberkulose med enkelt specificeret lokalisation	0			
DA191;Akut miliær tuberkulose med flere lokalisationer	0			
DA192;Akut miliær tuberkulose UNS	0			
DA198;Anden miliær tuberkulose	0			
DA199;Miliær tuberkulose UNS	0			
DA20;Pest	0			
DA200;Byldepest	0			
DA201;Hudpest	0			
DA202;Lungepest	0			
DA203;Meningitis forårsaget af Yersenia pestis	0			
DA207;Sepsisk pest	0			
DA208;Anden form for pest	0			
DA209;Pest UNS	0			
DA21;Tularæmi	0			
DA210;Ulceroglandulær tularæmi	0			
DA211;Okuloglandulær tularæmi	0			
DA212;Pulmonal tularæmi	0			

DA213;Gastrointestinal tularæmi	0			
DA217;Tyføs tularæmi	0			
DA218;Anden form for tularæmi	0			
DA219;Tularæmi UNS	0			
DA22;Miltbrand	0			
DA220;Kutan miltbrand	0			
DA221;Pulmonal miltbrand	0			
DA222;Gastrointestinal miltbrand	0			
DA227;Miltbrandsepsis	0			
DA228;Anden form for miltbrand	0			
DA229;Miltbrand UNS	0			
DA23;Svingefeber	0			
DA230;Brucellose forårsaget af Brucella melitensis	0			
DA231;Brucellose forårsaget af Brucella abortus	0			
DA232;Brucellose forårsaget af Brucella suis	0			
DA233;Brucellose forårsaget af Brucella canis	0			
DA238;Anden brucellose	0			
DA239;Brucellose UNS	0			
DA24;Glanders og melioidosis	0			
DA240;Glanders	0			
DA241;Akut eller fulminant melioidose	0			
DA242;Subakut eller kronisk melioidose	0			
DA243;Anden form for melioidose	0			
DA244;Melioidose UNS	0			
DA25;Rottebidfeber sygdomme	0			
DA250;Spirillose	0			
DA250A;Sodoku	0			
DA251;Streptobacillose	0			
DA259;Rottebidfeber UNS	0			
DA26;Svinerosen	0			
DA260;Kutant erysipeloid	0			
DA267;Sepsis forårsaget af Erysipelothrix rhusiopathiae	0			

DA268;Anden form for erysipeloid	0			
DA269;Erysipeloid UNS	0			
DA27;Leptospirose	0			
DA270;Weils sygdom	0			
DA278;Anden leptospirose	0			
DA279;Leptospirose UNS	0			
DA28;Andre dyreoverførte bakterielle infektioner IKA	0			
DA280;Pasteurellose	0			
DA281;Kattekradsningsfeber	0			
DA282;Yersinia infektion udenfor mave-tarm-kanalen	0			
DA282B;Yersinia sepsis	0			
DA288;Anden dyreoverført bakteriel infektion	0			
DA289;Dyreoverført bakteriel infektion UNS	0			
DA30;Spedalskhed	0			
DA300;Tidlig spedalskhed	0			
DA301;Tuberkuloid spedalskhed	0			
DA302;Borderline tuberkuloid spedalskhed	0			
DA303;Borderline spedalskhed	0			
DA304;Borderline lepromatøs spedalskhed	0			
DA305;Lepromatøs spedalskhed	0			
DA308;Anden form for spedalskhed	0			
DA309;Spedalskhed UNS	0			
DA31;Sygdomme forårsaget af andre mykobakterier	0			
DA310;Mykobakteriel lungeaffektion	0			
DA310A;Lungeinfektion med Mycobacterium avium	0			
DA310B;Lungeinfektion med Mycobacterium intracellularis	0			
DA310C;Lungeinfektion med Mycobacterium kansasii	0			
DA311;Mykobakteriel hudaffektion	0			
DA311A;Hudinfektion med Mycobacterium marinum	0			
DA311B;Hudinfektion med Mycobacterium ulcerans	0			
DA318;Anden mykobakteriel infektion	0			
DA319;Mykobakteriel infektion UNS	0			

DA32;Listeriose	0			
DA320;Listeriose i huden	0			
DA321;Listeriose i centralnervesystemet	0			
DA327;Listeriasepsis	0			
DA328;Anden form for listeriose	0			
DA328A;Okuloglandulær listeriosis	0			
DA329;Listeriose UNS	0			
DA33;Stivkrampe hos nyfødte	0			
DA339;Stivkrampe hos nyfødt	0			
DA34;Obstetrisk stivkrampe	0			
DA349;Stivkrampe i barselsperioden	0			
DA35;Anden form for stivkrampe	0			
DA359;Stivkrampe IKA	0			
DA36;Difteri	0			
DA360;Svælgdifteri	0			
DA360A;Tonsildifteri	0			
DA361;Difteri i nasopharynx	0			
DA362;Larynxdifteri	0			
DA362A;Difterisk laryngotrakeitis	0			
DA363;Difteri i huden	0			
DA368;Anden form for difteri	0			
DA369;Difteri UNS	0			
DA37;Kighoste	0			
DA370;Kighoste forårsaget af Bordetella pertussis	0			
DA371;Kighoste forårsaget af Bordetella parapertussis	0			
DA378;Kighoste forårsaget af anden Bordetella-type	0			
DA379;Kighoste UNS	0			
DA38;Skarlagensfeber	0			
DA389;Skarlagensfeber UNS	0			
DA39;Infektioner med meningokokker	0			
DA390;Meningitis forårsaget af meningokokker	0			
DA391;Waterhouse-Friderichsens syndrom	0			

Supplement 2 Nielsen et al. Mortality after paediatric emergency calls for patients with and without pre-existing comorbidity (2023)

DA392;Akut meningokokbakteriæmi	0			
DA392A;Meningokoksepsis	0			
DA393;Kronisk meningokokbakteriæmi	0			
DA394;Meningokokbakteriæmi UNS	0			
DA395;Hjertesygdom forårsaget af meningokokker	0			
DA398;Anden meningokokinfektion	0			
DA399;Meningokokinfektion UNS	0			
DA40;Sepsis forårsaget af streptokokker	0			
DA400;Sepsis forårsaget af Streptococcus A	0			
DA401;Sepsis forårsaget af Streptococcus B	0			
DA402;Sepsis forårsaget af Streptococcus D og Enterococcus	0			
DA403;Sepsis forårsaget af Streptococcus pneumoniae	0			
DA408;Anden streptokoksepsis	0			
DA409;Streptokoksepsis UNS	0			
DA41;Anden sepsis	0			
DA410;Sepsis forårsaget af Staphylococcus aureus	0			
DA411;Sepsis forårsaget af anden stafylokok	0			
DA411A;Sepsis forårsaget af koagulase-negative stafylokokker	0			
DA412;Sepsis forårsaget af stafylokokker UNS	0			
DA413;Sepsis forårsaget af Haemophilus influenzae	0			
DA414;Sepsis forårsaget af anaerob bakterie	0			
DA415;Sepsis forårsaget af anden gramnegativ organisme	0			
DA415A;Sepsis forårsaget af Escherichia coli	0			
DA418;Anden sepsis IKA	0			
DA419;Sepsis UNS	0			
DA419A;Septisk shock (findes ikke i SKS)	0			
DA419B;Urosepsis	0			
DA419C;Svær sepsis	0			
DA42;Aktinomykose	0			
DA420;Pulmonal aktinomykose	0			
DA421;Abdominal aktinomykose	0			
DA422;Cervikofacial aktinomykose	0			

DA427;Sepsis forårsaget af aktinomykose	0		
DA428;Anden form for aktinomykose	0		
DA429;Aktinomykose UNS	0		
DA43;Nocardiose	0		
DA430;Pulmonal nocardiose	0		
DA431;Kutan nocardiose	0		
DA438;Anden form for nocardiose	0		
DA439;Nocardiose UNS	0		
DA44;Bartonellose	0		
DA440;Systemisk bartonellose	0		
DA441;Kutan og mukokutan bartonellose	0		
DA448;Anden form for bartonellose	0		
DA449;Bartonellose UNS	0		
DA46;Rosen	0		
DA469;Rosen UNS	0		
DA48;Andre bakterielle sygdomme IKA	0		
DA480;Gasgangræn	0		
DA481;Legionærsygdom	0		
DA482;Pontiacfeber	0		
DA483;Toksisk shock-syndrom	0		
DA484;Brasiliansk purpurafeber	0		
DA484A;Systemisk Haemophilus aegypticus-infektion	0		
DA488;Anden bakteriel sygdom	0		
DA488A;Trichomycosis axillaris	0		
DA49;Bakteriel infektion uden angivelse af lokalisation	0		
DA490;Stafylokokinfektion UNS	0		
DA491;Streptokok- og/eller enterokokinfektion, uspecificeret lokalisation	0		
DA491A;Perianal streptokokinfektion (findes ikke i SKS)	0		
DA492;Infektion med Haemophilus influenzae UNS	0		
DA493;Mycoplasmainfektion UNS	0		
DA498;Anden bakteriel infektion uden angivelse af lokalisation	0		
DA499;Bakteriel infektion UNS	0		

DA499A;Bakteriæmi UNS	0		
DA50;Medfødt syfilis	0		
DA500;Tidlig medfødt symptomgivende syfilis	0		
DA501;Tidlig medfødt latent syfilis	0		
DA502;Tidlig medfødt syfilis UNS	0		
DA503;Sen medfødt syfilitisk øjenlidelse	0		
DA504;Sen medfødt neurosyfilis	0		
DA505;Anden sen medfødt symptomatisk syfilis	0		
DA505A;Hutchinsons triade	0		
DA505B;Hutchinsons tænder	0		
DA505C;Syfilitisk saddelnæse	0		
DA506;Sen medfødt seropositiv syfilis uden kliniske manifestationer	0		
DA507;Sen medfødt syfilis UNS	0		
DA509;Medfødt syfilis UNS	0		
DA51;Frisk syfilis	0		
DA510;Primær genital syfilis	0		
DA511;Primær anal syfilis	0		
DA512;Primær syfilis med anden lokalisation	0		
DA513;Sekundær syfilis i hud eller slimhinder	0		
DA513A;Condyloma syphiliticum	0		
DA513F;Syfilitisk mukøst plaque	0		
DA514;Anden sekundær syfilis	0		
DA514A;Syfilitisk lymfeknudesvulst	0		
DA515;Frisk latent syfilis	0		
DA519;Frisk syfilis UNS	0		
DA52;Syfilis i sent stadium	0		
DA520;Kardiovaskulær syfilis	0		
DA521;Sen symptomatisk neurosyfilis	0		
DA521D;Tabes dorsalis	0		
DA522;Asymptomatisk neurosyfilis	0		
DA523;Neurosyfilis UNS	0		
DA527;Anden form for sen symptomatisk syfilis	0		

Supplement 2 Nielsen et al. Mortality after paediatric emergency calls for patients with and without pre-existing comorbidity (2023)

DA527A;Syfilitisk gumma	0			
DA527C;Anden form for sen eller tertiær syfilis	0			
DA528;Sen latent syfilis	0			
DA529;Syfilis i sent stadium UNS	0			
DA53;Anden syfilis og syfilis UNS	0			
DA530;Latent syfilis uden specificering af stadie	0			
DA539;Syfilis UNS	0			
DA54;Gonoré	0			
DA540;Gonokokinfektion i nedre urin- og kønsorganer uden absces i periuretrale eller accessorisk	0			
DA540A;Gonoroisk cervicitis UNS	0			
DA540B;Gonoroisk cystitis UNS	0			
DA540D;Gonoroisk uretritis UNS	0			
DA540E;Gonoroisk vulvovaginitis UNS	0			
DA540F;Gonoroisk balanopostitis	0			
DA541;Gonokokinfektion i nedre urin- og kønsorganer med absces i periuretrale eller accessorisk	0			
DA541A;Urogenital gonoroisk absces	0			
DA541B;Gonoroisk bartholinitis	0			
DA542;Gonoroisk pelvioperitonitis eller anden gonoroisk urogenital infektion	0			
DA543;Gonokokinfektion i øje	0			
DA543A;Gonoroisk purulent konjunktivitis hos nyfødt	0			
DA544;Gonokokinfektion i led og muskler	0			
DA545;Gonokokinfektion i svælget	0			
DA546;Gonokokinfektion i anus og rektum	0			
DA548;Anden gonokokinfektion	0			
DA548B;Gonoroisk dermatitis	0			
DA548G;Gonoroisk sepsis	0			
DA549;Gonokokinfektion UNS	0			
DA55;Venerisk lymfogranulom forårsaget af klamydiainfektion	0			
DA559;Lymfogranulom forårsaget af Chlamydia	0			
DA56;Andre seksuelt overførte klamydiainfektioner	0			
DA560;Klamydiainfektion i nedre urin- og kønsorganer	0			
DA560A;Klamydiacervicitis	0			

DA560C;Klamydiacystitis	0		
DA560D;Klamydiauretritis	0		
DA560E;Vulvovaginitis forårsaget af klamydia	0		
DA561;Klamydiainfektion i nedre bækken eller andet urin- og kønsorgan	0		
DA562;Klamydiainfektion i urin- og kønsorganer UNS	0		
DA563;Klamydiainfektion i anus og rektum	0		
DA564;Klamydiainfektion i svælget	0		
DA568;Seksuelt overført klamydiainfektion med anden lokalisation	0		
DA57;Blød chanker	0		
DA579;Ulcus molle UNS	0		
DA58;Granulom forårsaget af infektion med Calymmatobacterium granulomatis	0		
DA589;Granuloma inguinale UNS	0		
DA59;Infektioner med Trichomonas vaginalis	0		
DA590;Trichomoniasis i urin- og kønsorganer	0		
DA590A;Vaginalt udflåd forårsaget af trichomonasis	0		
DA590C;Trichomonasvaginitis	0		
DA598;Trichomoniasis med anden lokalisation	0		
DA599;Trichomoniasis UNS	0		
DA60;Herpes simplex-infektion i anogenitalregionen	0		
DA600;Herpes simplex-infektion i urin- og kønsorganer	0		
DA601;Herpes simplex-infektion i analregion og endetarm	0		
DA601A;Herpes simplex-infektion i anus	0		
DA601B;Herpes simplex-infektion i perianalregionen	0		
DA601C;Herpes simplex-infektion i rektum	0		
DA609;Herpes simplex-infektion i anogenitalregionen UNS	0		
DA63;Andre fortrinsvis seksuelt overførte sygdomme, som ikke er klassificeret andetsteds	0		
DA630;Condyloma acuminatum i anogenitalregionen	0		
DA630B;Uretrale kondylomer	0		
DA630C;Genitale kondylomer	0		
DA630D;Anale kondylomer	0		
DA630E;Ekstragenitale kondylomer	0		
DA638;Anden fortrinsvis seksuelt overført sygdom IKA	0		

DA638A;Gardnerella infektion	0		
DA64;Seksuelt overført sygdom uden nærmere specificering	0		
DA649;Seksuelt overført sygdom UNS	0		
DA65;Ikke-venerisk syfilis	0		
DA659;Ikke-venerisk syfilis UNS	0		
DA66;Frambøsia	0		
DA660;Tidlig frambøsia	0		
DA661;Multiple frambøsia papillomer	0		
DA662;Andet tidligt hudsymptom ved frambøsia	0		
DA663;Hyperkeratose ved frambøsia	0		
DA664;Gumma og sår ved frambøsia	0		
DA665;Rhinopharyngitis mutilans ved frambøsia	0		
DA666;Knogle- og ledforandringer ved frambøsia	0		
DA667;Anden manifestation ved frambøsia	0		
DA668;Latent frambøsia	0		
DA669;Frambøsia UNS	0		
DA67;Pinta	0		
DA670;Primær manifestation ved pinta	0		
DA671;Sekundær manifestation ved pinta	0		
DA672;Sen manifestation ved pinta	0		
DA673;Blandede manifestationer ved pinta	0		
DA679;Pinta UNS	0		
DA68;Tilbagefaldsfeber	0		
DA680;Tilbagefaldsfeber overført af lus	0		
DA681;Tilbagefaldsfeber overført af flåt	0		
DA689;Tilbagefaldsfeber UNS	0		
DA69;Andre infektioner forårsaget af spirokæter	0		
DA690;Nekrotiserende ulcerøs mundbetændelse	0		
DA690A;Cancrum oris	0		
DA690B;Noma	0		
DA690C;Stomatitis ulcerosa Vincent	0		
DA691;Anden Vincent-infektion	0		

Supplement 2 Nielsen et al. Mortality after paediatric emergency calls for patients with and without pre-existing comorbidity (2023)

DA691A;Angina ulcerosa Vincent	0			
DA691B;Faryngitis forårsaget af spirokæteinfektion	0			
DA691C;Vincents angina	0			
DA691E;Akut nekrotiserende ulcerøs stomatitis	0			
DA691F;Vincents gingivitis	0			
DA691G;Faryngitis forårsaget af fusospirokæteinfektion	0			
DA692;Lymes sygdom	0			
DA692A;Kronisk atrofisk akrodermatit	0			
DA692D;Borrelia polyradikulitis	0			
DA692F;Erythema chronicum migrans	0			
DA698;Anden spirokæteinfektion	0			
DA699;Spirokæteinfektion UNS	0			
DA70;Infektioner forårsaget af Chlamydophila psittaci	0			
DA709;Chlamydophila psittaci-infektion	0			
DA71;Ægyptisk øjensygdom	0			
DA710;Tidligt stadie af trakom	0			
DA711;Aktivt stadie af trakom	0			
DA719;Trakom UNS	0			
DA74;Andre sygdomme forårsaget af klamydia-bakterier	0			
DA740;Klamydiakonjunktivitis	0			
DA748;Anden klamydia-infektion	0			
DA749;Klamydia-infektion UNS	0			
DA75;Rickettsioser overført af lus, lopper og mider	0			
DA750;Plettyfus forårsaget af Rickettsia prowazekii	0			
DA751;Brill-Zinssers sygdom	0			
DA752;Plettyfus forårsaget af Rickettsia typhi	0			
DA753;Plettyfus forårsaget af Rickettsia tsutsugamushi	0			
DA759;Plettyfus UNS	0			
DA77;Rickettsioser overført af flåt	0			
DA770;Rickettsiose forårsaget af Rickettsia rickettsii	0			
DA771;Rickettsiose forårsaget af Rickettsia conorii	0			
DA772;Rickettsiose forårsaget af Rickettsia sibirica	0			

DA773;Rickettsiose forårsaget af Rickettsia australis	0		
DA778;Anden rickettsiose overført af flåt	0		
DA779;Rickettsiose overført af flåt UNS	0		
DA78;Q-feber	0		
DA789;Q-feber UNS	0		
DA79;Andre rickettsioser	0		
DA790;Skyttegravsfeber	0		
DA791;Rickettsiose forårsaget af Rickettsia akari	0		
DA798;Anden rickettsiose	0		
DA798A;Rickettsiose forårsaget af Neorickettsia sennetsu [Ehrlichia sennetsu]	0		
DA798B;Rickettsiose forårsaget af Ehrlichia phagocytophila	0		
DA799;Rickettsiose UNS	0		
DA80;Akut polio	0		
DA800;Akut polio med lammelser forårsaget af vaccine	0		
DA801;Akut polio med lammelser forårsaget af importeret vild virus	0		
DA802;Akut polio med lammelser forårsaget af indenlandsk vild virus	0		
DA803;Anden eller ikke specificeret akut polio med lammelser	0		
DA804;Akut polio uden lammelser	0		
DA809;Akut polio UNS	0		
DA81;Atypiske virusinfektioner i centralnervesystemet			2
DA810;Creutzfeldt-Jakobs sygdom			2
DA811;Subakut skleroserende panencephalitis			2
DA812;Progressiv multifokal leukoencefalopati			2
DA818;Anden atypisk virusinfektion i centralnervesystemet			2
DA818A;Kuru			2
DA818B;Fatal familiær insomni			2
DA819;Atypisk virusinfektion i centralnervesystemet UNS			2
DA819A;Prionsygdom i centralnervesystemet UNS			2
DA82;Hundegalskab	0		
DA820;Sylvatisk rabies (lyssavirus type 1)	0		
DA821;Flagermusrabies (European Bat Lyssavirus - EBL)	0		
DA829;Rabies UNS	0		

DA83;Viral hjernebetændelse overført af myg	0			
DA830;Japansk hjernebetændelse	0			
DA831;Encephalomyelitis equina occidentalis	0			
DA832;Encephalomyelitis equina orientalis	0			
DA833;St Louis-encephalitis	0			
DA834;Australsk encephalitis	0			
DA835;Californisk encephalitis	0			
DA836;Rocio virus-encephalitis	0			
DA838;Anden viral hjernebetændelse overført af myg	0			
DA839;Viral hjernebetændelse overført af myg UNS	0			
DA84;Viral hjernebetændelse overført af flåt	0			
DA840;Encephalitis viralis occidentalis	0			
DA841;Centraleuropæisk viral encephalitis overført af flåt	0			
DA848;Anden viral hjernebetændelse overført af flåt	0			
DA849;Viral hjernebetændelse overført af flåt UNS	0			
DA85;Viral hjernebetændelse IKA	0			
DA850;Hjernebetændelse forårsaget af enterovirus	0			
DA851;Hjernebetændelse forårsaget af adenovirus	0			
DA852;Viral hjernebetændelse overført af leddyr UNS	0			
DA858;Anden viral hjernebetændelse	0			
DA858A;Encephalitis lethargica	0			
DA86;Viral hjernebetændelse UNS	0			
DA869;Viral encephalitis UNS	0			
DA87;Viral hjernehindebetændelse IKA	0			
DA870;Meningitis forårsaget af enterovirus	0			
DA870A;Meningitis forårsaget af coxsackievirus	0			
DA870B;Meningitis forårsaget af echovirus	0			
DA871;Meningitis forårsaget af adenovirus	0			
DA872;Lymfocytær koriomeningitis	0			
DA872A;Lymfocytær meningoencephalitis	0			
DA878;Anden viral hjernehindebetændelse	0			
DA879;Viral hjernehindebetændelse UNS	0			

DA88;Andre virale infektioner i centralnervesystemet IKA	0			
DA880;Febris enteroviralis exanthematica	0			
DA881;Vertigo epidemica	0			
DA888;Anden viral infektion i centralnervesystemet IKA	0			
DA89;Virale infektioner i centralnervesystemet UNS	0			
DA899;Viral infektion i centralnervesystemet UNS	0			
DA909;Denguefeber UNS (findes ikke i SKS)	0			
DA92;Andre virale febersygdomme overført af myg	0			
DA920;Chikungunya virussygdom	0			
DA921;O'nyong-nyong-feber	0			
DA922;Venezuelansk ekvin encephalitis	0			
DA923;West Nile feber	0			
DA924;Rift Valley-feber	0			
DA925;Zika virussygdom	0			
DA928;Anden viral febersygdom overført af myg	0			
DA929;Viral febersygdom overført af myg UNS	0			
DA93;Andre virale febersygdomme overført af leddyr IKA	0			
DA930;Oropouche virussygdom	0			
DA931;Sandfluefeber	0			
DA932;Colorado flåtfeber	0			
DA938;Anden viral febersygdom overført af leddyr	0			
DA94;Viral febersygdom overført af leddyr UNS	0			
DA949;Viral febersygdom overført af leddyr UNS	0			
DA95;Gul feber	0			
DA950;Sylvatisk gul feber	0			
DA951;Urban gul feber	0			
DA959;Gul feber UNS	0			
DA96;Arenavirale febersygdomme med blødninger	0			
DA960;Junin hæmoragisk feber	0			
DA961;Machupo hæmoragisk feber	0			
DA962;Lassafeber	0			
DA968;Anden arenaviral febersygdom med blødninger	0			

DA969;Arenaviral feber med blødninger UNS	0			
DA97;Dengue	0			
DA970;Dengue uden advarselstegn	0			
DA971;Dengue med advarselstegn	0			
DA972;Alvorlig Dengue	0			
DA979;Dengue UNS	0			
DA98;Andre virale febersygdomme med blødninger	0			
DA980;Krim-Congo hæmoragisk feber	0			
DA981;Omsk hæmoragisk feber	0			
DA982;Kyasanurfeber	0			
DA983;Marburgfeber	0			
DA984;Ebolavirus-sygdom	0			
DA985;Hæmoragisk feber med renalt syndrom	0			
DA985A;Epidemisk hæmoragisk feber	0			
DA985B;Hantavirus-sygdom	0			
DA985C;Epidemisk nefropati	0			
DA988;Anden viral febersygdom med blødninger	0			
DA99;Virale febersygdomme med blødninger UNS	0			
DA999;Viral febersygdom med blødninger UNS	0			
DB00;Herpes simplex-infektion	0			
DB000;Herpeseksem	0			
DB001;Herpesudbrud med blærer	0			
DB001A;Herpes facialis	0			
DB001B;Herpes labialis	0			
DB001C;Ekstern otitis forårsaget af Herpes simplex-virus	0			
DB002;Gingivostomatitis eller faryngotonsillitis forårsaget af Herpes simplex-virus	0			
DB002A;Gingivostomatitis forårsaget af Herpes simplex-virus	0			
DB002B;Faryngotonsillitis forårsaget af Herpes simplex-virus	0			
DB002C;Faryngitis forårsaget af Herpes simplex-virus	0			
DB003;Meningitis forårsaget af Herpes simplex-virus	0			
DB004;Encephalitis forårsaget af Herpes simplex-virus	0			
DB004A;Meningoencephalitis forårsaget af Herpes simplex-virus	0			

Supplement 2 Nielsen et al. Mortality after paediatric emergency calls for patients with and without pre-existing comorbidity (2023)

DB005;Herpes simplex-infektion i øje	0			
DB007;Herpes generalisata	0			
DB008;Anden form for Herpes simplex-virus infektion	0			
DB009;Infektion med Herpes simplex-virus UNS	0			
DB01;Skoldkopper	0			
DB010;Meningitis forårsaget af Varicella zoster	0			
DB011;Encephalitis forårsaget af Varicella zoster	0			
DB011A;Encephalomyelitis forårsaget af Varicella zoster	0			
DB012;Pneumoni forårsaget af Varicella zoster	0			
DB018;Skoldkopper med anden komplikation	0			
DB019;Skoldkopper UNS	0			
DB02;Zoster	0			
DB020;Herpes zoster-encephalitis	0			
DB020A;Herpes zoster-meningoencephalitis	0			
DB021;Herpes zoster-meningitis	0			
DB022;Herpes zoster med anden komplikation i nervesystemet	0			
DB023;Herpes zoster i øje	0			
DB027;Dissemineret herpes zoster	0			
DB028;Herpes zoster-infektion med anden komplikation	0			
DB029;Herpes zoster-infektion uden komplikation	0			
DB03;Kopper	0			
DB039;Kopper UNS	0			
DB04;Abekopper (monkeypox)	0			
DB049;Abekopper (monkeypox) UNS	0			
DB05;Mæslinger	0			
DB050;Mæslinger kompliceret med encephalitis	0			
DB051;Mæslinger kompliceret med meningitis	0			
DB052;Mæslinger kompliceret med pneumoni	0			
DB053;Mæslinger kompliceret med mellemørebetændelse	0			
DB054;Mæslinger med tarmkomplikation	0			
DB058;Mæslinger med anden komplikation	0			
DB059;Mæslinger uden komplikationer	0			

Supplement 2 Nielsen et al. Mortality after paediatric emergency calls for patients with and without pre-existing comorbidity (2023)

DB06;Røde hunde	0			
DB060;Røde hunde med neurologisk komplikation	0			
DB068;Røde hunde med anden komplikation	0			
DB069;Røde hunde uden komplikationer	0			
DB07;Virale vorter	0			
DB079;Verrucae virales UNS	0			
DB079A;Verruca plana juvenilis	0			
DB079B;Verruca vulgaris (findes ikke i SKS)	0			
DB079C;Verruca plantaris	0			
DB079D;Epidermodysplasia verruciformis	0			
DB08;Andre virussygdomme karakteriseret ved hud- og slimhindeaffektioner IKA	0			
DB080;Anden poxvirusinfektion	0			
DB080A;Milkers nodule	0			
DB080B;Orf	0			
DB081;Molluscum contagiosum	0			
DB082;Tredagesfeber	0			
DB083;Lussingesyge	0			
DB084;Hånd-, fod- og mundsygdom	0			
DB085;Herpangina	0			
DB088;Anden virussygdom med hud- og slimhindeaffektion	0			
DB088A;Mund- og klovsyge	0			
DB088B;Pharyngitis enteroviralis lymphonodularis	0			
DB088C;Viralt eksantem (findes ikke i SKS)	0			
DB09;Virussygdomme uden nærmere specificering karakteriseret ved hud- og slimhindeaffektione	0			
DB099;Virusinfektion karakteriseret ved hud- og slimhindeaffektion UNS	0			
DB099A;Viralt eksantem UNS	0			
DB15;Akut hepatitis A	0			
DB150;Hepatitis A med leverkoma	0			
DB159;Hepatitis A uden leverkoma	0			
DB159A;Hepatitis A UNS	0			
DB16;Akut hepatitis B	0			
DB160;Akut hepatitis B med coinfektion med Delta agens med leverkoma	0			

Supplement 2 Nielsen et al. Mortality after paediatric emergency calls for patients with and without pre-existing comorbidity (2023)

DB161;Akut hepatitis B med coinfektion med Delta agens uden leverkoma	0		
DB162;Akut hepatitis B uden coinfektion med Delta agens med leverkoma	0		
DB169;Akut hepatitis B uden coinfektion med Delta agens og uden leverkoma	0		
DB169A;Hepatitis B UNS	0		
DB17;Anden akut viral leverbetændelse	0		
DB170;Akut Delta-superinfektion i kronisk hepatitis B	0		
DB171;Akut hepatitis C	0		
DB172;Akut hepatitis E	0		
DB178;Anden akut viral hepatitis	0		
DB179;Akut viral hepatitis UNS	0		
DB18;Kronisk viral leverbetændelse	0		
DB180;Kronisk viral hepatitis B med Delta agens	0		
DB181;Kronisk viral hepatitis B uden Delta agens	0		
DB182;Kronisk viral hepatitis C	0		
DB188;Anden kronisk viral hepatitis	0		
DB189;Kronisk viral hepatitis UNS	0		
DB19;Viral leverbetændelse uden nærmere specificering	0		
DB190;Viral hepatitis UNS med leverkoma			2
DB199;Viral hepatitis UNS uden leverkoma	0		
DB20;Humant immundefekt-virus (HIV) sygdom medførende infektiøse og parasitære sygdomme	0		
DB200;HIV-sygdom med mykobakteriel infektion	0		
DB200A;HIV-sygdom med tuberkulose	0		
DB201;HIV-sygdom med andre bakterielle sygdomme	0		
DB202;HIV-sygdom med cytomegalovirus sygdom	0		
DB203;HIV-sygdom med andre virus infektioner	0		
DB204;HIV-sygdom med candidiasis	0		
DB205;HIV-sygdom med andre svampeinfektioner	0		
DB206;HIV-sygdom med Pneumocystis jirovecii pneumoni	0		
DB207;HIV-sygdom med multiple infektioner	0		
DB208;HIV-sygdom med andre infektiøse og parasitære sygdomme	0		
DB209;HIV-sygdom med infektiøs eller parasitær sygdom UNS	0		
DB22;Humant immundefekt-virus (HIV) sygdom med andre specificerede sygdomme	0		

Supplement 2 Nielsen et al. Mortality after paediatric emergency calls for patients with and without pre-existing comorbidity (2023)

DB220;HIV-encefalopati	0		
DB220A;HIV-demens			2
DB221;HIV-lymfoid interstitiel pneumonitis	0		
DB222;HIV-wasting syndrome	0		
DB227;HIV-sygdom med multiple sygdomme klassificeret andetsteds	0		
DB23;Andre tilstande ved HIV-sygdom	0		
DB230;Akut HIV-sygdom	0		
DB231;HIV-sygdom med (persisterende) generaliseret lymfadenopati	0		
DB232;HIV-sygdom med hæmatologiske og immunologiske forandringer IKA	0		
DB238;Anden eller ikke specificeret symptomatisk HIV-sygdom	0		
DB238A;HIV-associeret lipodystrofisyndrom (HALS)	0		
DB24;HIV-sygdom og AIDS uden nærmere specificering	0		
DB249;AIDS UNS			2
DB25;Cytomegalovirale sygdomme	0		
DB250;Cytomegalovirus-pneumoni	0		
DB251;Cytomegalovirus-hepatitis	0		
DB252;Cytomegalovirus-pankreatitis	0		
DB258;Anden sygdom forårsaget af cytomegalovirus	0		
DB259;Cytomegaloviral sygdom UNS	0		
DB26;Fåresyge	0		
DB260;Fåresygeorkitis	0		
DB261;Fåresygemeningitis	0		
DB262;Fåresygeencephalitis	0		
DB263;Fåresygepankreatitis	0		
DB268;Fåresyge med anden komplikation	0		
DB269;Fåresyge uden komplikationer	0		
DB27;Mononukleose	0		
DB270;Mononukleose forårsaget af Epstein-Barr virus	0		
DB271;Mononukleose forårsaget af cytomegalovirus	0		
DB278;Anden infektiøs mononukleose	0		
DB279;Mononukleose UNS	0		
DB30;Viral konjunktivitis	0		

Supplement 2 Nielsen et al. Mortality after paediatric emergency calls for patients with and without pre-existing comorbidity (2023)

DB300;Keratokonjunktivitis forårsaget af adenovirus	0			
DB301;Konjunktivitis forårsaget af adenovirus	0			
DB301A;Akut follikulær konjunktivitis forårsaget af adenovirus	0			
DB301B;Svømmebassin-konjunktivitis	0			
DB302;Viral faryngokonjunktivitis	0			
DB303;Akut epidemisk hæmoragisk konjunktivitis	0			
DB303A;Konjunktivitis forårsaget af coxsackievirus type 24	0			
DB303B;Konjunktivitis forårsaget af enterovirus type 70	0			
DB303C;Akut hæmoragisk konjunktivitis UNS	0			
DB308;Anden viral konjunktivitis	0			
DB308A;Konjunktivitis forårsaget af Newcastle-virus	0			
DB309;Viral konjunktivitis UNS	0			
DB33;Andre virussygdomme IKA	0			
DB330;Epidemisk myalgi	0			
DB331;Ross River-sygdom	0			
DB332;Viruskarditis	0			
DB333;Retrovirus-infektion IKA	0			
DB334;Hantavirus med (kardio-)pulmonalt syndrom	0			
DB338;Anden virussygdom	0			
DB34;Virussygdom uden angivelse af lokalisation	0			
DB340;Adenovirus-infektion uden angivelse af lokalisation	0			
DB341;Enterovirus-infektion uden angivelse af lokalisation	0			
DB341A;Coxsackievirus-infektion UNS	0			
DB341B;Echovirus-infektion UNS	0			
DB342;Coronavirus-infektion uden angivelse af lokalisation	0			
DB342A;COVID-19-infektion uden angivelse af lokalisation	0			
DB343;Parvovirus-infektion uden angivelse af lokalisation	0			
DB344;Papovavirus-infektion uden angivelse af lokalisation	0			
DB348;Anden virusinfektion uden angivelse af lokalisation	0			
DB349;Virusinfektion UNS	0			
DB349A;Viræmi UNS	0			
DB35;Hudsvamp	0			

DB350;Svampesygdom i hovedbunden eller skægregionen	0			
DB350A;Tinea capitis	0			
DB350B;Tinea barbae	0			
DB350C;Kerion	0			
DB351;Neglesvamp	0			
DB352;Håndsvamp	0			
DB353;Fodsvamp	0			
DB354;Svampesygdom på kroppen	0			
DB354A;Dermatophytosis perigenitalis	0			
DB355;Tinea imbricata	0			
DB356;Svampesygdom på benet	0			
DB356B;Dermatophytosis inguinalis	0			
DB358;Anden form for dermatofytose	0			
DB358A;Dermatophytosis disseminata	0			
DB358B;Favus	0			
DB359;Dermatofytose UNS	0			
DB36;Andre overfladiske svampeinfektioner	0			
DB360;Pityriasis versicolor	0			
DB361;Tinea nigra	0			
DB362;Hvid piedra	0			
DB363;Sort piedra	0			
DB368;Anden overfladisk svampeinfektion	0			
DB368A;Pityrosporum folliculitis	0			
DB369;Overfladisk svampeinfektion UNS	0			
DB37;Infektion med Candida albicans	0			
DB370;Candidiasis i mundhule	0			
DB370A;Trøske	0			
DB371;Candidiasis i lunge	0			
DB372;Candidiasis i hud og negle	0			
DB372A;Candidiasis cutis	0			
DB372B;Candidiasis unguinis	0			
DB373;Candidiasis i vulva og vagina	0			

Supplement 2 Nielsen et al. Mortality after paediatric emergency calls for patients with and without pre-existing comorbidity (2023)

DB373A;Candidiasis-vaginitis	0		
DB373B;Candidiasis-vulvitis	0		
DB373C;Candidiasis-vulvovaginitis	0		
DB374;Urogenital candidiasis med anden lokalisation	0		
DB375;Candidiasis-meningitis	0		
DB376;Candidiasis-endokarditis	0		
DB377;Candidiasis-sepsis	0		
DB378;Candidiasis med anden lokalisation	0		
DB378A;Candidiasis-keilitis	0		
DB378B;Candidiasis-enteritis	0		
DB378C;Candidiasis-øsofagitis	0		
DB379;Candidiasis UNS	0		
DB38;Coccidioidomykose	0		
DB380;Akut pulmonal coccidioidomykose	0		
DB381;Kronisk pulmonal coccidioidomykose	0		
DB382;Pulmonal coccidioidomykose UNS	0		
DB383;Kutan coccidioidomykose	0		
DB384;Coccidioido-meningitis	0		
DB387;Dissemineret coccidioidomykose	0		
DB388;Anden form for coccidioidomykose	0		
DB389;Coccidioidomykose UNS	0		
DB39;Histoplasmose	0		
DB390;Akut pulmonal Histoplasmosis capsulati	0		
DB391;Kronisk pulmonal Histoplasmosis capsulati	0		
DB392;Pulmonal Histoplasmosis capsulati UNS	0		
DB393;Dissemineret Histoplasmosis capsulati	0		
DB394;Histoplasmosis capsulati UNS	0		
DB395;Histoplasmosis duboisii	0		
DB399;Histoplasmose UNS	0		
DB40;Blastomykose	0		
DB400;Akut pulmonal blastomykose	0		
DB401;Kronisk pulmonal blastomykose	0		

DB402;Pulmonal blastomykose UNS	0			
DB403;Kutan blastomykose	0			
DB407;Dissemineret blastomykose	0			
DB408;Anden form for blastomykose	0			
DB409;Blastomykose UNS	0			
DB41;Paracoccidioidomykose	0			
DB410;Pulmonal paracoccidioidomykose	0			
DB411;Dissemineret paracoccidioidomykose	0			
DB418;Anden form for paracoccidioidomykose	0			
DB419;Paracoccidioidomykose UNS	0			
DB42;Sporotrikose	0			
DB420;Pulmonal sporotrikose	0			
DB421;Lymfokutan sporotrikose	0			
DB427;Dissemineret sporotrikose	0			
DB428;Anden form for sporotrikose	0			
DB429;Sporotrikose UNS	0			
DB43;Kromomykose og fæomykotisk absces	0			
DB430;Kutan kromomykose	0			
DB431;Fæomykotisk hjerneabsces	0			
DB432;Subkutan fæomykotisk absces	0			
DB438;Anden form for kromomykose	0			
DB439;Kromomykose UNS	0			
DB44;Aspergillose	0			
DB440;Invasiv pulmonal aspergillose	0			
DB441;Anden pulmonal aspergillose	0			
DB441A;Pulmonalt aspergillom	0			
DB441B;Trakeobronkial aspergillose	0			
DB442;Tonsillær aspergillose	0			
DB447;Dissemineret aspergillose	0			
DB448;Anden form for aspergillose	0			
DB448A;Kolonisering med aspergillus	0			
DB448B;Ekstern otitis ved aspergillose	0			

DB449;Aspergillosis UNS	0		
DB45;Kryptokokkose	0		
DB450;Pulmonal kryptokokkose	0		
DB451;Cerebral kryptokokkose	0		
DB451B;Meningocerebral kryptokokkose	0		
DB452;Kutan kryptokokkose	0		
DB453;Ossøs kryptokokkose	0		
DB457;Dissemineret kryptokokkose	0		
DB458;Anden form for kryptokokkose	0		
DB459;Kryptokokkose UNS	0		
DB46;Zygomykose	0		
DB460;Pulmonal mukormykose	0		
DB461;Rinocerebral mukormykose	0		
DB462;Gastrointestinal mukormykose	0		
DB463;Kutan mukormykose	0		
DB463A;Subkutan mukormykose	0		
DB464;Dissemineret mukormykose	0		
DB465;Mukormykose UNS	0		
DB468;Anden form for zygomykose	0		
DB468A;Entomophthoromykose	0		
DB469;Zygomykose UNS	0		
DB469B;Fykomykose UNS	0		
DB47;Mycetoma	0		
DB470;Eumycetom	0		
DB471;Aktinomycetom	0		
DB479;Mycetoma UNS	0		
DB48;Andre mykoser IKA	0		
DB480;Lobomykose	0		
DB481;Rinosporidiose	0		
DB482;Allescheriasis	0		
DB483;Geotrikose	0		
DB484;Penicillose	0		

DB485;Pneumocystose	0			
DB487;Opportunistisk mykose	0			
DB488;Anden mykose	0			
DB49;Ikke nærmere specificerede mykoser	0			
DB499;Mykose UNS	0			
DB499A;Fungæmi UNS	0			
DB50;Plasmodium falciparum-malaria	0			
DB500;Plasmodium falciparum-malaria med cerebrale komplikationer	0			
DB500A;Cerebral malaria UNS	0			
DB508;Anden alvorlig eller kompliceret Plasmodium falciparum-malaria	0			
DB509;Plasmodium falciparum-malaria uden komplikationer	0			
DB51;Plasmodium vivax-malaria	0			
DB510;Plasmodium vivax-malaria med miltruptur	0			
DB518;Plasmodium vivax-malaria med anden komplikation	0			
DB519;Plasmodium vivax-malaria uden komplikationer	0			
DB52;Plasmodium malariae-malaria	0			
DB520;Plasmodium malariae-malaria med nefropati	0			
DB528;Plasmodium malariae-malaria med anden komplikation	0			
DB529;Plasmodium malariae-malaria uden komplikationer	0			
DB53;Anden parasitologisk verificeret malaria	0			
DB530;Plasmodium ovale-malaria	0			
DB531;Malaria forårsaget af abe-plasmodier	0			
DB538;Anden parasitologisk verificeret malaria IKA	0			
DB538A;Parasitologisk verificeret malaria UNS	0			
DB54;Malaria uden nærmere specificering	0			
DB549;Klinisk malaria, ikke parasitologisk verificeret	0			
DB55;Leishmaniasis	0			
DB550;Visceral leishmaniasis	0			
DB551;Kutan leishmaniasis	0			
DB552;Mukokutan leishmaniasis	0			
DB559;Leishmaniasis UNS	0			
DB56;Afrikansk sovesyge	0			

DB560;Vestafrikansk sovesyge	0			
DB561;Østafrikansk sovesyge	0			
DB569;Afrikansk sovesyge UNS	0			
DB57;Chagas' sygdom	0			
DB570;Akut Chagas' sygdom med hjertekomplikationer	0			
DB571;Akut Chagas' sygdom uden hjertekomplikationer	0			
DB572;Kronisk Chagas' sygdom med hjertekomplikationer	0			
DB572A;Trypanosomiasis UNS i område, hvor Chagas' sygdom forekommer	0			
DB573;Kronisk Chagas' sygdom med gastrointestinale komplikationer	0			
DB574;Chagas' sygdom med neurologiske komplikationer	0			
DB575;Chagas' sygdom med anden komplikation	0			
DB579;Chagas' sygdom UNS	0			
DB58;Toksoplasmose	0			
DB580;Toksoplasmose i øje	0			
DB581;Hepatitis ved toksoplasmose	0			
DB582;Meningoencephalitis ved toksoplasmose	0			
DB583;Pulmonal toksoplasmose	0			
DB588;Toksoplasmose med anden lokalisation IKA	0			
DB589;Toksoplasmose UNS	0			
DB599;Pneumocystose UNS (findes ikke i SKS)	0			
DB60;Andre sygdomme forårsaget af protozoer IKA	0			
DB600;Babesiose	0			
DB600A;Piroplasmose	0			
DB601;Acanthamoebiasis	0			
DB602;Naegleriasis	0			
DB608;Anden sygdom forårsaget af protozoer	0			
DB608A;Mikrosporidiose	0			
DB64;Sygdomme forårsaget af protozoer UNS	0			
DB649;Sygdom forårsaget af protozoer UNS	0			
DB65;Skistosomiasis	0			
DB650;Skistosomiasis forårsaget af Schistosoma haematobium	0			
DB651;Skistosomiasis forårsaget af Schistosoma mansoni	0			

Supplement 2 Nielsen et al. Mortality after paediatric emergency calls for patients with and without pre-existing comorbidity (2023)

DB652;Skistosomiasis forårsaget af Schistosoma japonicum	0			
DB653;Dermatitis cercaria	0			
DB658;Anden type skistosomiasis	0			
DB659;Skistosomiasis UNS	0			
DB66;Andre ikte-infektioner	0			
DB660;Opisthorchiasis	0			
DB661;Clonorchiasis	0			
DB662;Dicrocoeliasis	0			
DB663;Fascioliasis	0			
DB664;Paragonimiasis	0			
DB665;Fasciolopsiasis	0			
DB668;Anden ikte-infektion	0			
DB669;Ikte-infektion UNS	0			
DB67;Ekinokokkose	0			
DB670;Infektion med Echinococcus granulosus i leveren	0			
DB671;Infektion med Echinococcus granulosus i lunge	0			
DB672;Infektion med Echinococcus granulosus i knogle	0			
DB673;Infektion med Echinococcus granulosus med anden eller multiple lokalisationer	0			
DB674;Infektion med Echinococcus granulosus UNS	0			
DB675;Infektion med Echinococcus multilocularis i leveren	0			
DB676;Infektion med Echinococcus multilocularis med anden eller multiple lokalisationer	0			
DB677;Infektion med Echinococcus multilocularis UNS	0			
DB678;Ekinokokinfektion i leveren UNS	0			
DB679;Anden eller ikke specificeret ekinokokinfektion	0			
DB68;Infektion med bændelorm	0			
DB680;Taeniasis forårsaget af Taenia solium	0			
DB681;Taeniasis forårsaget af Taenia saginata	0			
DB689;Taeniasis UNS	0			
DB69;Cysticerkose	0			
DB690;Cysticerkose i centralnervesystemet	0			
DB691;Cysticerkose i øje	0			
DB698;Cysticerkose med anden lokalisation	0			

DB699;Cysticerkose UNS	0			
DB70;Diphyllobothriasis og sparganosis	0			
DB700;Diphyllobothriasis	0			
DB701;Sparganosis	0			
DB71;Andre bændelormeinfestationer	0			
DB710;Hymenolepiasis	0			
DB711;Dipylidiasis	0			
DB718;Anden bændelormeinfestation	0			
DB719;Infestation med bændelorm UNS	0			
DB72;Dracunculiasis	0			
DB729;Dracunculiasis UNS	0			
DB73;Onchocerciasis	0			
DB739;Onchocerkose UNS	0			
DB74;Filariose	0			
DB740;Wuchereria bancrofti filariasis	0			
DB741;Brugia malayi filariasis	0			
DB742;Brugia timori filariasis	0			
DB743;Loa loa filariasis	0			
DB744;Mansonelliasis	0			
DB748;Anden filariose	0			
DB748B;Dirofilariose	0			
DB749;Filariose UNS	0			
DB75;Trikinose	0			
DB759;Trikinose UNS	0			
DB76;Infestation med hageorm	0			
DB760;Ancylostomiasis	0			
DB761;Infestation med Necator americanus	0			
DB768;Anden form for infestation med hageorm	0			
DB769;Infestation med hageorm UNS	0			
DB769A;Larva migrans cutanea UNS	0			
DB77;Askariose	0			
DB770;Intestinal ascariasis	0			

DB778;Ascariasis med anden komplikation	0			
DB779;Ascariasis UNS	0			
DB78;Strongyloidose	0			
DB780;Intestinal strongyloidiasis	0			
DB781;Kutan strongyloidiasis	0			
DB787;Dissemineret strongyloidiasis	0			
DB789;Strongyloidiasis UNS	0			
DB79;Infektion med piskeorm	0			
DB799;Infektion med piskeorm UNS	0			
DB80;Infektion med børneorm	0			
DB809;Infektion med børneorm UNS	0			
DB81;Andre intestinale ormeinfestationer IKA	0			
DB810;Anisakiasis	0			
DB811;Intestinal capillariasis	0			
DB812;Trichostrongyliasis	0			
DB813;Intestinal angiostrongyliasis	0			
DB814;Blandet intestinal ormeinfestation	0			
DB818;Anden intestinal ormeinfestation	0			
DB82;Intestinale infestationer med parasitter uden nærmere specificering	0			
DB820;Intestinal ormesygdom UNS	0			
DB829;Intestinal infestation med parasitter UNS	0			
DB83;Andre ormesygdomme	0			
DB830;Larva migrans visceralis	0			
DB830A;Toxocariasis	0			
DB831;Gnathostomiasis	0			
DB832;Angiostrongyliasis forårsaget af Parastrongylus cantonensis	0			
DB833;Syngamiasis	0			
DB834;Intern infestation med igler	0			
DB838;Anden ormesygdom	0			
DB839;Ormesygdom UNS	0			
DB85;Infestation med lus	0			
DB850;Infestation med hovedlus	0			

DB851;Infestation med kropslus	0			
DB852;Infestation med lus UNS	0			
DB853;Infestation med fladlus	0			
DB854;Blandingsinfestation med lus	0			
DB86;Fnat	0			
DB869;Scabies UNS	0			
DB869A;Scabies crustosa	0			
DB869B;Persisterende noduli ved scabies	0			
DB87;Myiasis	0			
DB870;Kutan myiasis	0			
DB871;Myiasis i sår	0			
DB872;Okulær myiasis	0			
DB873;Nasofaryngeal myiasis	0			
DB874;Myiasis i øre	0			
DB878;Myiasis med anden lokalisation	0			
DB879;Myiasis UNS	0			
DB88;Andre infestationer med leddyr	0			
DB880;Anden sygdom fremkaldt af mider	0			
DB881;Infestation med sandlopper	0			
DB882;Infestation med andet leddyr	0			
DB883;Ekstern infestation med igler	0			
DB888;Anden infestation med leddyr	0			
DB888A;Cheyletiella-infestation	0			
DB888B;Infestation med væggelus	0			
DB888C;Infestation med lopper	0			
DB888D;Infestation med hårsækmider	0			
DB889;Infestation med leddyr UNS	0			
DB89;Sygdom forårsaget af parasit UNS	0			
DB899;Parasitær sygdom UNS	0			
DB899A;Parasitæmi UNS	0			
DB90;Følger efter tuberkulose	0			
DB900;Følger efter tuberkulose i centralnervesystemet	0			

DB901;Følger efter tuberkulose i urin- og kønsorganer	0		
DB902;Følger efter tuberkulose i knogler og led	0		
DB908;Følger efter tuberkulose i andet organ	0		
DB909;Følger efter tuberkulose i åndedrætsorgan og uden angivelse af lokalisation	0		
DB91;Følger efter polio	0		
DB919;Følger efter poliomyelitis	0		
DB92;Følger efter spedalskhed	0		
DB929;Følger efter lepra	0		
DB94;Følger efter andre infektiøse og parasitære sygdomme	0		
DB940;Følger efter trakom	0		
DB941;Følger efter viral hjernebetændelse	0		
DB942;Følger efter viral leverbetændelse	0		
DB948;Følger efter anden infektiøs eller parasitær sygdom	0		
DB948A;Følger efter COVID-19	0		
DB949;Følger efter infektiøs eller parasitær sygdom UNS	0		
DB95;Streptokokker og stafylokokker som årsag til sygdom	0		
DB950;Streptokokker, gruppe A, som årsag til sygdom	0		
DB951;Streptokokker, gruppe B, som årsag til sygdom	0		
DB952;Streptokokker, gruppe D, som årsag til sygdom	0		
DB953;Pneumokokker som årsag til sygdom	0		
DB954;Anden streptokok som årsag til sygdom	0		
DB955;Streptokokker UNS som årsag til sygdom	0		
DB956;Staphylococcus aureus som årsag til sygdom	0		
DB956A;Staphylococcus aureus methicillin resistent (MRSA)	0		
DB957;Anden stafylokok som årsag til sygdom	0		
DB958;Stafylokok UNS som årsag til sygdom	0		
DB96;Andre bakterier som årsag til sygdom	0		
DB960;Mycoplasma pneumoniae som årsag til sygdom	0		
DB961;Klebsiella pneumoniae om årsag til sygdom	0		
DB962;Escherichia coli som årsag til sygdom	0		
DB963;Haemophilus influenzae som årsag til sygdom	0		
DB964;Proteus som årsag til sygdom	0		

Supplement 2 Nielsen et al. Mortality after paediatric emergency calls for patients with and without pre-existing comorbidity (2023)

DB965;Pseudomonas som årsag til sygdom	0		
DB966;Bacillus fragilis som årsag til sygdom	0		
DB967;Clostridium perfringens som årsag til sygdom	0		
DB968;Anden bakterie som årsag til sygdom	0		
DB97;Virus som årsag til sygdom	0		
DB970;Adenovirus som årsag til sygdom	0		
DB971;Enterovirus som årsag til sygdom	0		
DB972;Coronavirus som årsag til sygdom	0		
DB972A;COVID-19 svær akut respiratorisk syndrom	0		
DB972B;Multisystem inflammatorisk syndrom associeret med COVID-19	0		
DB972B1;Multisystem Inflammatory Syndrome in Children (MIS-C)	0		
DB973;Retrovirus som årsag til sygdom	0		
DB974;Respiratorisk syncytialvirus (RS-virus) som årsag til sygdom	0		
DB975;Reovirus som årsag til sygdom	0		
DB976;Parvovirus som årsag til sygdom	0		
DB977;Papillomavirus som årsag til sygdom	0		
DB978;Anden virus som årsag til sygdom	0		
DB98;Andre infektiøse agentia som årsag til sygdom	0		
DB980;Helicobacter pylori som årsag til sygdom	0		
DB981;Vibrio vulnificus som årsag til sygdom	0		
DB99;Andre eller ikke nærmere specificerede infektiøse sygdomme	0		
DB999;Anden eller ikke specificeret infektionssygdom	0		
DC00;Læbekræft		1	
DC000;Kræft i overlæbens yderside		1	
DC001;Kræft i underlæbens yderside		1	
DC002;Kræft i læbens yderside UNS		1	
DC003;Kræft i overlæbens slimhinde		1	
DC004;Kræft i underlæbens slimhinde		1	
DC005;Kræft i læbens slimhinde UNS		1	
DC006;Kræft i mundvig		1	
DC008;Kræft i læbe overgribende flere lokalisationer		1	
DC009;Læbekræft UNS		1	

DC01;Kræft i basis af tunge		1	
DC019;Kræft i tungebasis		1	
DC02;Kræft i andre og ikke specificerede dele af tungen		1	
DC020;Kræft i tungens overside		1	
DC021;Kræft i tungeranden		1	
DC021A;Kræft i tungespidsen		1	
DC022;Kræft i tungens underside		1	
DC022A;Kræft i tungebåndet		1	
DC023;Kræft i tungens forreste to tredjedele UNS		1	
DC024;Kræft i tonsilla lingualis		1	
DC028;Kræft i tungen overgribende flere lokalisationer		1	
DC029;Tungekræft UNS		1	
DC03;Kræft i tandkødet		1	
DC030;Kræft i tandkødet i overmunden		1	
DC031;Kræft i tandkødet i undermunden		1	
DC039;Kræft i tandkødet UNS		1	
DC04;Kræft i mundgulvet		1	
DC040;Kræft i mundgulvets forreste del		1	
DC041;Kræft i mundgulvets laterale del		1	
DC048;Kræft i mundgulvet overgribende flere lokalisationer		1	
DC049;Kræft i mundgulvet UNS		1	
DC05;Kræft i ganen		1	
DC050;Kræft i den hårde gane		1	
DC051;Kræft i den bløde gane		1	
DC052;Kræft i drøbelen		1	
DC058;Kræft i ganen overgribende flere lokalisationer		1	
DC059;Ganekræft UNS		1	
DC06;Kræft i andre og ikke specificerede dele af mundhulen		1	
DC060;Kræft i kindens slimhinde		1	
DC061;Kræft i vestibulum oris		1	
DC062;Kræft i slimhinden bag kindtænderne		1	
DC068;Kræft i mundhulen overgribende flere lokalisationer		1	

DC069;Kræft i mundhulen UNS		1	
DC07;Kræft i ørespytkirtel		1	
DC079;Kræft i ørespytkirtel		1	
DC08;Kræft i andre og ikke specificerede store spytkirtler		1	
DC080;Kræft i glandula submandibularis		1	
DC081;Kræft i glandula sublingualis		1	
DC088;Kræft i stor spytkirtel overgribende flere lokalisationer		1	
DC089;Kræft i stor spytkirtel UNS		1	
DC09;Kræft i tonsiller		1	
DC090;Kræft i tonsilleje		1	
DC091;Kræft i ganebuen		1	
DC091A;Kræft i forreste ganebue		1	
DC091B;Kræft i bageste ganebue		1	
DC092;Kræft i tonsilla palatina		1	
DC098;Kræft i tonsil overgribende flere lokalisationer		1	
DC099;Kræft i tonsil UNS		1	
DC10;Kræft i mundsvælget		1	
DC100;Kræft i vallecula epiglottica		1	
DC101;Kræft i strubelågets forflade		1	
DC102;Kræft i oropharynx lateralvæg		1	
DC103;Kræft i oropharynx bagvæg		1	
DC104;Kræft i brankialcyste eller brankialfure		1	
DC104A;Kræft i brankialcyste		1	
DC104B;Kræft i brankialfure		1	
DC108;Kræft i mundsvælget overgribende flere lokalisationer		1	
DC109;Kræft i mundsvælget UNS		1	
DC11;Kræft i næsesvælget		1	
DC110;Kræft i næsesvælgets loft		1	
DC111;Kræft i næsesvælgets bagvæg		1	
DC112;Kræft i næsesvælgets sidevæg		1	
DC113;Kræft i næsesvælgets forvæg		1	
DC118;Kræft i næsesvælget overgribende flere lokalisationer		1	

Supplement 2 Nielsen et al. Mortality after paediatric emergency calls for patients with and without pre-existing comorbidity (2023)

DC119;Kræft i næsesvælget UNS		1	
DC12;Kræft i recessus piriformis		1	
DC129;Kræft i recessus piriformis		1	
DC13;Kræft i hypopharynx		1	
DC130;Kræft i regio postcricoideae		1	
DC131;Kræft i plica aryepiglottica		1	
DC132;Kræft i hypopharynx bagvæg		1	
DC138;Kræft i hypopharynx overgribende flere lokalisationer		1	
DC139;Kræft i hypopharynx UNS		1	
DC14;Kræft med andre og dårligt specificerede lokalisationer i læbe, mundhule og svælg		1	
DC140;Kræft i svælget UNS		1	
DC141;Kræft i laryngopharynx		1	
DC142;Kræft i Waldeyers ring		1	
DC148;Kræft i læbe, mundhule og svælg overgribende flere lokalisationer		1	
DC15;Kræft i spiserøret			2
DC153;Kræft i spiserørets øverste tredjedel			2
DC154;Kræft i spiserørets midterste tredjedel			2
DC155;Kræft i spiserørets nederste tredjedel			2
DC158;Kræft i spiserøret overgribende flere lokalisationer			2
DC159;Kræft i spiserøret UNS			2
DC16;Kræft i mavesækken			2
DC160;Kræft i cardia			2
DC161;Kræft i fundus ventriculi			2
DC162;Kræft i corpus ventriculi			2
DC163;Kræft i antrum pyloricum			2
DC164;Kræft i pylorus			2
DC165;Kræft i curvatura gastrica minor			2
DC166;Kræft i curvatura gastrica major			2
DC168;Kræft i mavesækken overgribende flere lokalisationer			2
DC169;Kræft i mavesækken UNS			2
DC17;Kræft i tyndtarmen			2
DC170;Kræft i tolvfingertarmen			2

DC171;Kræft i jejunum			2
DC172;Kræft i ileum			2
DC173;Kræft i Meckels divertikel			2
DC178;Kræft i tyndtarmen overgribende flere lokalisationer			2
DC179;Kræft i tyndtarmen UNS			2
DC18;Kræft i tyktarmen			2
DC180;Kræft i caecum			2
DC180A;Kræft i valva ileocaecalis			2
DC181;Kræft i blindtarmen			2
DC182;Kræft i colon ascendens			2
DC183;Kræft i flexura coli dextra			2
DC184;Kræft i colon transversum			2
DC185;Kræft i flexura coli sinistra			2
DC186;Kræft i colon descendens			2
DC187;Kræft i colon sigmoideum			2
DC188;Kræft i tyktarmen overgribende flere lokalisationer			2
DC188A;Hereditær non-polypøs kolorektal cancer (HNPCC)			2
DC189;Kræft i tyktarmen UNS			2
DC20;Kræft i endetarmen			2
DC209;Kræft i endetarmen			2
DC21;Kræft i endetarmsåbningen og analkanalen			2
DC210;Kræft i endetarmsåbningen UNS			2
DC211;Kræft i analkanalen			2
DC212;Neoplasma malignum cloacogenica ani			2
DC218;Kræft i endetarm, endetarmsåbning og analkanal overgribende flere lokalisationer			2
DC22;Kræft i leveren og intrahepatiske galdegange			2
DC220;Hepatocellulært karcinom			2
DC221;Kræft i intrahepatiske galdegange			2
DC221A;Cholangiocarcinoma, intrahepatisk			2
DC222;Hepatoblastom			2
DC223;Angiosarkom i leveren			2
DC224;Anden form for sarkom i leveren			2

DC227;Anden kræft i leveren			2
DC229;Kræft i leveren UNS			2
DC23;Kræft i galdeblæren			2
DC239;Kræft i galdeblæren			2
DC24;Kræft i andre og ikke specificerede dele af galdeveje			2
DC240;Kræft i ekstrahepatiske galdeveje			2
DC240A;Kræft i ductus choledochus			2
DC240B;Kræft i ductus cysticus			2
DC240C;Kræft i ductus hepaticus communis			2
DC240D;Kræft i ductus hepaticus dexter			2
DC240E;Kræft i ductus hepaticus sinister			2
DC241;Kræft i papilla Vateri			2
DC248;Kræft i galdeveje overgribende flere lokalisationer			2
DC249;Kræft i galdeveje UNS			2
DC25;Kræft i bugspytkirtlen			2
DC250;Kræft i caput pancreatis			2
DC251;Kræft i corpus pancreatis			2
DC252;Kræft i cauda pancreatis			2
DC253;Kræft i ductus pancreaticus			2
DC254;Kræft i Langerhanske øer			2
DC257;Kræft i andre dele af pancreas			2
DC258;Kræft i pancreas overgribende flere lokalisationer			2
DC259;Kræft i pancreas UNS			2
DC26;Kræft i andre og dårligt specificerede fordøjelsesorganer			2
DC260;Kræft i mave-tarm-kanalen UNS			2
DC261;Kræft i milten			2
DC268;Kræft i fordøjelsesorganerne overgribende flere lokalisationer			2
DC269;Kræft i fordøjelseorganerne UNS			2
DC30;Kræft i næsehulen og mellemøret			2
DC300;Kræft i næsehulen			2
DC300A;Kræft i concha nasi			2
DC300B;Kræft i næseskillevæggen			2

DC300C;Kræft i vestibulum nasi			2
DC301;Kræft i mellemøre			2
DC301A;Kræft i cellulae mastoideae			2
DC301B;Kræft i tuba auditiva			2
DC31;Kræft i bihuler			2
DC310;Kræft i kæbehulen			2
DC311;Kræft i sinus ethmoidalis			2
DC312;Kræft i pandehulen			2
DC313;Kræft i sinus sphenoidalis			2
DC318;Kræft i bihuler overgribende flere lokalisationer			2
DC319;Kræft i bihule UNS			2
DC32;Kræft i strubehovedet			2
DC320;Kræft i glottis			2
DC320A;Kræft i stemmelæbe			2
DC321;Kræft i strubehovedet lokaliseret oven for glottis			2
DC321A;Kræft i epiglottis' bagflade			2
DC321B;Kræft i epiglottis lokaliseret over os hyoideum			2
DC321C;Kræft i laryngeale side af plica aryepiglottica			2
DC321D;Kræft i falsk stemmelæbe			2
DC322;Kræft i strubehovedet lokaliseret neden for glottis			2
DC323;Kræft i brusk i strubehovedet			2
DC328;Kræft i strubehovedet overgribende flere lokalisationer			2
DC329;Kræft i strubehovedet UNS			2
DC33;Kræft i luftrøret			2
DC339;Kræft i luftrøret			2
DC34;Kræft i bronkier og lunge			2
DC340;Kræft i hovedbronkie			2
DC340A;Kræft i lungehilus			2
DC340B;Kræft i carina tracheae			2
DC341;Kræft i lungens øverste lap			2
DC342;Kræft i lungens mellemste lap			2
DC343;Kræft i lungens nederste lap			2

DC348;Kræft i bronkier og lunge overgribende flere lokalisationer			2
DC349;Kræft i lunge UNS			2
DC37;Kræft i thymus			2
DC379;Kræft i thymus			2
DC38;Kræft i hjerte, mediastinum og lungehinde			2
DC380;Kræft i hjertet			2
DC380A;Kræft i perikardiet			2
DC381;Kræft i mediastinum anterius			2
DC382;Kræft i mediastinum posterius			2
DC383;Kræft i mediastinum UNS			2
DC384;Kræft i lungehinde			2
DC388;Kræft i hjerte, mediastinum og lungehinde overgribende flere lokalisationer			2
DC39;Kræft med andre og dårligt specificerede lokalisationer i åndedrætsorganer og organer i brysthulen			2
DC390;Kræft i øvre luftveje UNS			2
DC398;Kræft i brysthule og luftveje overgribende flere lokalisationer			2
DC399;Kræft i åndedrætsorgan eller organ i brysthule med dårligt specificeret lokalisation			2
DC40;Kræft i knogler og ledbrusk i ekstremiteter			2
DC400;Kræft i skulderblad, lange knogler eller ledbrusk i overekstremitet			2
DC400A;Kræft i ledbrusk på lang knogle i overekstremitet			2
DC400B;Kræft i ledbrusk på skulderblad			2
DC400C;Knoglekræft i lang knogle i overekstremitet			2
DC400D;Knoglekræft i skulderblad			2
DC401;Kræft i kort knogle eller ledbrusk i overekstremitet			2
DC401A;Kræft i ledbrusk på kort knogle i overekstremitet			2
DC401B;Knoglekræft i kort knogle i overekstremitet			2
DC402;Kræft i lang knogle eller ledbrusk i underekstremitet			2
DC402A;Kræft i ledbrusk på lang knogle i underekstremitet			2
DC402B;Knoglekræft i lang knogle i underekstremitet			2
DC402M;Kræft i lang knogle/ledbrusk i underekstremitet m.metastaser (findes ikke i SKS)			2
DC403;Kræft i kort knogle eller ledbrusk i underekstremitet			2
DC403A;Kræft i ledbrusk på kort knogle i underekstremitet			2
DC403B;Knoglekræft i kort knogle i underekstremitet			2

DC408;Kræft i knogle og ledbrusk i ekstremitet overgribende flere lokalisationer			2
DC409;Kræft i knogle eller ledbrusk i ekstremitet UNS			2
DC41;Kræft i knogler og ledbrusk med andre og ikke specificerede lokalisationer			2
DC410;Kræft i kranie eller ansigtsknogle			2
DC410A;Kræft i kranieknogle			2
DC410B;Kræft i ansigtsknogle UNS			2
DC410C;Kræft i ledbrusk i kraniet			2
DC410D;Kræft i ledbrusk i ansigtet			2
DC410E;Knoglekræft i overkæben			2
DC410F;Knoglekræft i øjenhule			2
DC410G;Kræft i ledbrusk i overkæben			2
DC411;Kræft i underkæben			2
DC411A;Kræft i ledbrusk i underkæben			2
DC412;Kræft i rygsøjlen			2
DC412A;Kræft i ledbrusk i rygsøjlen			2
DC412B;Knoglekræft i ryghvirvel			2
DC413;Kræft i ribben, brystben eller kraveben			2
DC413A;Kræft i ledbrusk på kraveben			2
DC413B;Kræft i ribbensbrusk			2
DC413C;Kræft i brystbenets ledbrusk			2
DC413D;Kræft i kraveben			2
DC413E;Kræft i ribben			2
DC413F;Kræft i brystbenet			2
DC414;Kræft i bækken, korsben eller haleben			2
DC414A;Kræft i brusk i halebenet			2
DC414B;Kræft i brusk i bækkenet			2
DC414C;Kræft i brusk i korsbenet			2
DC414D;Knoglekræft i halebenet			2
DC414E;Knoglekræft i bækkenknogle			2
DC414F;Knoglekræft i korsbenet			2
DC418;Kræft i knogle eller ledbrusk overgribende flere lokalisationer			2
DC419;Kræft i knogle eller ledbrusk UNS			2

DC419A;Kræft i ledbrusk UNS			2
DC419B;Kræft i knogle UNS			2
DC43;Modermærkekræft i huden			2
DC430;Malignt melanom i hud på læbe			2
DC431;Malignt melanom i hud på øjenlåg			2
DC431A;Malignt melanom i øjenkrog			2
DC431B;Malignt melanom i øjenlåg			2
DC432;Malignt melanom i hud på øre eller i ydre øregang			2
DC432A;Malignt melanom i hud på øre			2
DC432B;Malignt melanom i hud i ydre øregang			2
DC433;Malignt melanom med anden eller ikke specificeret lokalisation i ansigtet			2
DC434;Malignt melanom i hud på skalpen eller halsen			2
DC434A;Malignt melanom i hud på skalpen			2
DC434B;Malignt melanom i hud på halsen			2
DC435;Malignt melanom i hud på kroppen			2
DC435A;Malignt melanom i hud i anus			2
DC435B;Malignt melanom i hud på bryst			2
DC435C;Malignt melanom i hud i perineum			2
DC435L;Malignt melanom i anale marginalzone			2
DC436;Malignt melanom i hud på overekstremitet			2
DC436A;Malignt melanom i hud på skulder			2
DC436B;Malignt melanom i hud på arm			2
DC436C;Malignt melanom i hud på hånd eller finger			2
DC437;Malignt melanom i hud på underekstremitet			2
DC437A;Malignt melanom i hud på ben			2
DC437B;Malignt melanom i hud på hofte			2
DC437C;Malignt melanom i hud på fod eller tå			2
DC438;Malignt melanom overgribende flere lokalisationer			2
DC439;Malignt melanom i huden UNS			2
DC44;Anden hudkræft			2
DC440;Anden hudkræft på læbe			2
DC441;Anden hudkræft på øjenlåg			2

DC441A;Anden hudkræft i øjenkrog			2
DC442;Anden hudkræft på øre eller i ydre øregang			2
DC442A;Anden hudkræft på øre			2
DC442B;Anden hudkræft i ydre øregang			2
DC443;Anden hudkræft i ansigtet med anden eller ikke specificeret lokalisation			2
DC444;Anden hudkræft på skalpen eller halsen			2
DC444A;Anden hudkræft på skalpen			2
DC444B;Anden hudkræft på halsen			2
DC445;Anden hudkræft på kroppen			2
DC445A;Anden hudkræft i anus			2
DC445B;Anden hudkræft på bryst			2
DC445C;Anden hudkræft i perineum			2
DC446;Anden hudkræft på overekstremitet			2
DC446A;Anden hudkræft på skulder			2
DC446B;Anden hudkræft på arm			2
DC446C;Anden hudkræft på hånd eller finger			2
DC447;Anden hudkræft på underekstremitet			2
DC447A;Anden hudkræft på hofte			2
DC447B;Anden hudkræft på ben			2
DC447C;Anden hudkræft på fod eller tå			2
DC448;Anden hudkræft overgribende flere lokalisationer			2
DC449;Anden hudkræft UNS			2
DC45;Maligne mesoteliomer			2
DC450;Malignt mesoteliom i lungehinde			2
DC451;Malignt mesoteliom i bughinden			2
DC452;Malignt mesoteliom i perikardiet			2
DC457;Malignt mesoteliom med anden lokalisation			2
DC459;Malignt mesoteliom UNS			2
DC46;Kaposis sarkom			2
DC460;Kaposis sarkom i huden			2
DC461;Kaposis sarkom i bindevæv			2
DC462;Kaposis sarkom i ganen			2

DC463;Kaposis sarkom i lymfeknuder			2
DC467;Kaposis sarkom med anden lokalisation			2
DC468;Kaposis sarkom i multiple lokalisationer			2
DC469;Kaposis sarkom UNS			2
DC47;Kræft i perifere nerver og autonome nervesystem			2
DC470;Kræft i perifer nerve eller autonome nervesystem i hoved, ansigt eller hals			2
DC470A;Kræft i perifer nerve i hovedet			2
DC470B;Kræft i perifer nerve i halsen			2
DC470C;Kræft i perifer nerve i ansigtet			2
DC470D;Kræft i autonome nervesystem i hovedet			2
DC470E;Kræft i autonome nervesystem i halsen			2
DC470F;Kræft i autonome nervesystem i ansigtet			2
DC471;Kræft i perifer nerve eller autonome nervesystem i overekstremitet			2
DC471A;Kræft i perifer nerve i skulderregion			2
DC471B;Kræft i perifer nerve i arm eller hånd			2
DC471C;Kræft i autonome nervesystem i skulderregion			2
DC471D;Kræft i autonome nervesystem i arm eller hånd			2
DC472;Kræft i perifer nerve eller autonome nervesystem i underekstremitet			2
DC472A;Kræft i perifer nerve i hofteregion			2
DC472B;Kræft i perifer nerve i ben eller fod			2
DC472C;Kræft i autonome nervesystem i hofteregion			2
DC472D;Kræft i autonome nervesystem i ben eller fod			2
DC473;Kræft i perifer nerve eller autonome nervesystem i thorax			2
DC473A;Kræft i perifer nerve i thorax			2
DC473B;Kræft i autonome nervesystem i thorax			2
DC474;Kræft i perifer nerve eller autonome nervesystem i abdomen			2
DC474A;Kræft i perifer nerve i abdomen			2
DC474B;Kræft i autonome nervesystem i abdomen			2
DC475;Kræft i perifer nerve eller autonome nervesystem i bækkenet			2
DC475A;Kræft i perifer nerve i bækkenet			2
DC475B;Kræft i autonome nervesystem i bækkenet			2
DC476;Kræft i perifer nerve eller autonome nervesystem i truncus UNS			2

DC476A;Kræft i perifer nerve i truncus			2
DC476B;Kræft i autonome nervesystem i truncus			2
DC478;Kræft i perifere nerver eller autonome nervesystem overgribende flere lokalisationer			2
DC478A;Kræft i perifere nerver overgribende flere lokalisationer			2
DC478B;Kræft i autonome nervesystem overgribende flere lokalisationer			2
DC479;Kræft i perifer nerve eller autonome nervesystem UNS			2
DC479A;Kræft i perifer nerve UNS			2
DC479B;Kræft i autonome nervesystem UNS			2
DC48;Kræft i bughinden og i bughulens bagvæg			2
DC480;Kræft i retroperitoneum			2
DC481;Kræft i specificeret del af peritoneum			2
DC481A;Kræft i mesenteriet			2
DC481B;Kræft i mesocolon			2
DC481C;Kræft i oment			2
DC482;Kræft i peritoneum UNS			2
DC488;Kræft i bughinden og bughulens bagvæg overgribende flere lokalisationer			2
DC49;Kræft i andet bindevæv og bløddelsvæv			2
DC490;Kræft i bindevæv og bløddelsvæv i hoved, ansigt eller hals			2
DC490A;Kræft i bindevæv og bløddelsvæv i hovedet			2
DC490B;Kræft i bindevæv og bløddelsvæv i halsen			2
DC490C;Kræft i bindevæv og bløddelsvæv i ansigtet			2
DC491;Kræft i bindevæv og bløddelsvæv i overekstremitet			2
DC491A;Kræft i bindevæv og bløddelsvæv i skulderregion			2
DC491B;Kræft i bindevæv og bløddelsvæv i arm			2
DC492;Kræft i bindevæv og bløddelsvæv i underekstremitet			2
DC492A;Kræft i bindevæv og bløddelsvæv i ben			2
DC492B;Kræft i bindevæv og bløddelsvæv i hofteregion			2
DC493;Kræft i bindevæv og bløddelsvæv i thorax			2
DC493A;Kræft i bindevæv og bløddelsvæv i armhule			2
DC493B;Kræft i bindevæv og bløddelsvæv i mellemgulvet			2
DC494;Kræft i bindevæv og bløddelsvæv i abdomen			2
DC495;Kræft i bindevæv og bløddelsvæv i bækkenet			2

DC495A;Kræft i bindevæv og bløddelsvæv i perineum			2
DC496;Kræft i bindevæv og bløddelsvæv i truncus UNS			2
DC498;Kræft i bindevæv og bløddelsvæv overgribende flere lokalisationer			2
DC499;Kræft i bindevæv og bløddelsvæv UNS			2
DC50;Brystkræft			2
DC500;Kræft i brystvorte eller areola mammae			2
DC500A;Kræft i areolae mammae			2
DC500B;Kræft i brystvorte			2
DC501;Brystkræft i den centrale del af mamma			2
DC502;Brystkræft i den øvre mediale kvadrant af mamma			2
DC503;Brystkræft i den nedre mediale kvadrant af mamma			2
DC504;Brystkræft i den øvre laterale kvadrant af mamma			2
DC505;Brystkræft i den nedre laterale kvadrant af mamma			2
DC506;Brystkræft i processus axillaris mammae			2
DC508;Brystkræft overgribende flere lokalisationer			2
DC509;Brystkræft UNS			2
DC51;Kræft i ydre kvindelige kønsorganer			2
DC510;Kræft i stor kønslæbe			2
DC510A;Kræft i Bartholins kirtel			2
DC511;Kræft i lille kønslæbe			2
DC512;Kræft i klitoris			2
DC518;Kræft i ydre kvindelige kønsorganer overgribende flere lokalisationer			2
DC519;Kræft i ydre kvindelige kønsorganer UNS			2
DC52;Kræft i vagina			2
DC529;Vaginakræft			2
DC53;Kræft i livmoderhalsen			2
DC539;Livmoderhalskræft			2
DC54;Kræft i livmoderen			2
DC549;Livmoderkræft			2
DC55;Kræft i livmoderen uden nærmere specificeret lokalisation			2
DC559;Kræft i livmoderen uden nærmere specificeret lokalisation			2
DC56;Kræft i æggestok			2

Supplement 2 Nielsen et al. Mortality after paediatric emergency calls for patients with and without pre-existing comorbidity (2023)

DC569;Æggestokkræft			2
DC569A;Ekstragonadal germinalcelletumor (ovarie)			2
DC57;Kræft i andre og ikke specificerede kvindelige kønsorganer			2
DC570;Kræft i æggeleder			2
DC571;Kræft i ligamentum latum uteri			2
DC572;Kræft i ligamentum teres uteri			2
DC573;Kræft i parametriet			2
DC574;Kræft i adnexa uteri			2
DC577;Kræft i anden specificeret struktur i kvindeligt kønsorgan			2
DC578;Kræft i kvindelige kønsorganer overgribende flere lokalisationer			2
DC579;Kræft i kvindeligt kønsorgan UNS			2
DC58;Kræft i moderkagen			2
DC589;Kræft i moderkagen UNS			2
DC589A;Choriokarcinom i placenta			2
DC589B;Chorionepiteliom i placenta			2
DC60;Kræft i penis			2
DC600;Kræft i forhuden			2
DC601;Kræft i glans penis			2
DC602;Kræft i corpus penis			2
DC608;Kræft i penis overgribende flere lokalisationer			2
DC609;Kræft i penis UNS			2
DC61;Kræft i blærehalskirtlen			2
DC619;Prostatakræft			2
DC619Y;PSA-recidiv efter tidligere intenderet kurativ behandling			2
DC619Z;Kastrationsresistent prostatakræft (CRPC)			2
DC62;Testikelkræft			2
DC620;Kræft i ikke-nedstegen testikel			2
DC621;Kræft i testikel i scrotum			2
DC629;Testikelkræft UNS			2
DC629A;Ekstragonadal germinalcelletumor (testis)			2
DC63;Kræft i andre og ikke specificerede mandlige kønsorganer			2
DC630;Kræft i bitestikel			2

DC631;Kræft i sædstreng			2
DC632;Kræft i scrotum			2
DC637;Kræft i andet mandligt kønsorgan			2
DC638;Kræft i mandlige kønsorganer overgribende flere lokalisationer			2
DC639;Kræft i mandligt kønsorgan UNS			2
DC64;Nyrekræft			2
DC649;Nyrekræft			2
DC649X;Lokalrecidiv fra nyrekræft (findes ikke i SKS)			2
DC65;Kræft i nyrebækken			2
DC659;Kræft i nyrebækken			2
DC66;Kræft i urinleder			2
DC669;Kræft i urinleder			2
DC67;Kræft i urinblæren			2
DC677;Kræft i urachus			2
DC679;Kræft i urinblæren UNS			2
DC68;Kræft i andre og ikke specificerede urinorganer			2
DC680;Kræft i urinrøret			2
DC681;Kræft i glandula paraurethralis s. bulbourethralis			2
DC688;Kræft i urinorganer overgribende flere lokalisationer			2
DC689;Kræft i urinorgan UNS			2
DC69;Kræft i øje og omgivende strukturer			2
DC690;Kræft i konjunktiva			2
DC691;Kræft i cornea			2
DC692;Kræft i retina			2
DC692A;Retinoblastom			2
DC693;Kræft i choroidea			2
DC694;Kræft i corpus ciliare			2
DC695;Kræft i tårekirtel eller tårekanal			2
DC695A;Kræft i tårekanal			2
DC695B;Kræft i tårekirtel			2
DC696;Kræft i øjenhule			2
DC698;Kræft i øje overgribende flere lokalisationer			2

DC699;Kræft i øje UNS			2
DC699A;Kræft i øjeæble			2
DC70;Kræft i hjernehinde og rygmarvshinde			2
DC700;Kræft i hjernehinde			2
DC701;Kræft i rygmarvshinde			2
DC709;Kræft i hjernehinde eller rygmarvshinde UNS			2
DC71;Kræft i hjernen			2
DC710;Kræft i storhjernen			2
DC710A;Kræft i corpus callosum			2
DC710B;Supratentoriel kræft i storhjernen UNS			2
DC711;Kræft i hjernens pandelap			2
DC712;Kræft i hjernens tindingelap			2
DC713;Kræft i hjernens isselap			2
DC714;Kræft i hjernens nakkelap			2
DC715;Intraventrikulær kræft i hjernen			2
DC716;Kræft i lillehjernen			2
DC717;Kræft i hjernestammen eller 4. ventrikel			2
DC717A;Kræft i 4. ventrikel			2
DC717B;Kræft i hjernestammen			2
DC717C;Infratentoriel kræft i hjernen UNS			2
DC718;Kræft i hjernen overgribende flere lokalisationer			2
DC719;Kræft i hjernen UNS			2
DC72;Kræft i rygmarv, kranienerver og andre dele af centralnervesystemet			2
DC720;Kræft i rygmarven			2
DC721;Kræft i cauda equina			2
DC722;Kræft i lugtenerve			2
DC722A;Kræft i bulbus olfactorius			2
DC723;Kræft i synsnerve			2
DC724;Kræft i hørenerve			2
DC725;Kræft i anden hjernenerve eller hjernenerve UNS			2
DC728;Kræft i centralnervesystemet overgribende flere lokalisationer			2
DC729;Kræft i centralnervesystemet UNS			2

DC73;Kræft i skjoldbruskkirtlen			2
DC739;Kræft i skjoldbruskkirtlen			2
DC74;Kræft i binyre			2
DC740;Kræft i binyrebark			2
DC741;Kræft i binyremarv			2
DC749;Kræft i binyre UNS			2
DC75;Kræft i andre endokrine kirtler og lignende strukturer			2
DC750;Kræft i biskjoldbruskkirtel			2
DC751;Kræft i hypofysen			2
DC752;Kræft i ductus craniopharyngeus			2
DC753;Kræft i corpus pineale			2
DC754;Kræft i glomus caroticum			2
DC755;Kræft i corpus para-aorticus eller andet paraganglion			2
DC755A;Kræft i corpus para-aorticus			2
DC755B;Kræft i andet paraganglion			2
DC755M;Kræft i corpus para-aorticus/andet paraganglion m.metastaser (findes ikke i SKS)			2
DC758;Pluriglandulær kræft UNS			2
DC759;Kræft i endokrin kirtel UNS			2
DC76;Kræft med andre og dårligt specificerede lokalisationer			2
DC760;Kræft i hoved, ansigt eller hals uden nærmere specificeret lokalisation			2
DC760A;Kræft i hovedet uden nærmere specificeret lokalisation			2
DC760B;Kræft i halsen uden nærmere specificeret lokalisation			2
DC760C;Kræft i ansigtet uden nærmere specificeret lokalisation			2
DC761;Kræft i thorax uden nærmere specificeret lokalisation			2
DC762;Kræft i abdomen uden nærmere specificeret lokalisation			2
DC763;Kræft i bækkenet uden nærmere specificeret lokalisation			2
DC764;Kræft i overekstremitet uden nærmere specificeret lokalisation		1	
DC765;Kræft i underekstremitet uden nærmere specificeret lokalisation		1	
DC767;Kræft med anden dårligt specificeret lokalisation			2
DC768;Kræft med anden eller dårligt specificeret lokalisation overgribende flere lokalisationer			2
DC77;Metastaser og kræft UNS i lymfeknuder			2
DC770;Metastase eller kræft UNS i lymfeknude i hoved, ansigt eller hals			2

DC770A;Metastase i lymfeknude i hovedet			2
DC770A1;Regional lymfeknudemetastase i hovedet			2
DC770A2;Fjernmetastase i lymfeknude i hovedet			2
DC770B;Kræft UNS i lymfeknude i hovedet			2
DC770C;Metastase i lymfeknude i halsen			2
DC770C1;Regional lymfeknudemetastase i halsen			2
DC770C2;Fjernmetastase i lymfeknude i halsen			2
DC770D;Kræft UNS i lymfeknude i halsen			2
DC770E;Metastase i lymfeknude i ansigtet			2
DC770E1;Regional lymfeknudemetastase i ansigtet			2
DC770E2;Fjernmetastase i lymfeknude i ansigtet			2
DC770F;Kræft UNS i lymfeknude i ansigtet			2
DC770G;Metastase i supraclaviculær lymfeknude			2
DC770G1;Regional supraclaviculær lymfeknudemetastase			2
DC770G2;Fjernmetastase i supraclaviculær lymfeknude			2
DC770H;Kræft UNS i supraclaviculær lymfeknude			2
DC771;Metastase eller kræft UNS i intratorakal lymfeknude			2
DC771A;Metastase i intratorakal lymfeknude			2
DC771A1;Regional intratorakal lymfeknudemetastase			2
DC771A2;Fjernmetastase i intratorakal lymfeknude			2
DC771B;Kræft UNS i intratorakal lymfeknude			2
DC772;Metastase eller kræft UNS i intraabdominal lymfeknude			2
DC772A;Metastase i intraabdominal lymfeknude			2
DC772A1;Regional intraabdominal lymfeknudemetastase			2
DC772A2;Fjernmetastase i intraabdominal lymfeknude			2
DC772B;Kræft UNS i intraabdominal lymfeknude			2
DC773;Metastase eller kræft UNS i lymfeknude i aksil eller overekstremiteten			2
DC773A;Metastase i lymfeknude i aksil			2
DC773A1;Regional lymfeknudemetastase i aksil			2
DC773A2;Fjernmetastase i lymfeknude i aksil			2
DC773B;Kræft UNS i lymfeknude i aksil			2
DC773C;Metastase i lymfeknude i overekstremitet			2

DC773C1;Regional lymfeknudemetastase i overekstremitet			2
DC773C2;Fjernmetastase i lymfeknude i overekstremitet			2
DC773D;Kræft UNS i lymfeknude i overekstremitet			2
DC773E;Metastase i pektoral lymfeknude			2
DC773E1;Regional pektoral lymfeknudemetastase			2
DC773E2;Fjernmetastase i pektoral lymfeknude			2
DC773F;Kræft UNS i pektoral lymfeknude			2
DC774;Metastase eller kræft UNS i lymfeknude i lyske eller underekstremitet			2
DC774A;Metastase i lymfeknude i underekstremitet			2
DC774A1;Regional lymfeknudemetastase i underekstremitet			2
DC774A2;Fjernmetastase i lymfeknude i underekstremitet			2
DC774B;Kræft UNS i lymfeknude i underekstremitet			2
DC774C;Metastase i lymfeknude i lyske			2
DC774C1;Regional lymfeknudemetastase i lyske			2
DC774C2;Fjernmetastase i lymfeknude i lyske			2
DC774D;Kræft UNS i lymfeknude i lyske			2
DC775;Metastase eller kræft UNS i intrapelvin lymfeknude			2
DC775A;Metastase i intrapelvin lymfeknude			2
DC775A1;Regional intrapelvin lymfeknudemetastase			2
DC775A2;Fjernmetastase i intrapelvin lymfeknude			2
DC775B;Kræft UNS i intrapelvin lymfeknude			2
DC778;Metastase eller kræft UNS i lymfeknuder i multiple lokalisationer			2
DC778A;Metastase i lymfeknuder i multiple lokalisationer			2
DC778A1;Multiple regionale lymfeknudemetastaser			2
DC778A2;Fjernmetastaser i lymfeknuder i multiple lokalisationer			2
DC778B;Kræft UNS i lymfeknuder i multiple lokalisationer			2
DC779;Metastase eller kræft UNS i lymfeknude UNS			2
DC779A;Metastase i lymfeknude UNS			2
DC779A1;Regional lymfeknudemetastase UNS			2
DC779A2;Fjernmetastase i lymfeknude UNS			2
DC779B;Kræft UNS i lymfeknude UNS			2
DC78;Metastaser i åndedrætsorganer og fordøjelsessystemet			2

DC780;Fjernmetastase i lunge			2
DC781;Fjernmetastase i mediastinum			2
DC782;Fjernmetastase i lungehinde			2
DC782A;Carcinomatosis pleurae			2
DC782B;Malign pleural effusion			2
DC783;Fjernmetastase med anden eller ikke specificeret lokalisation i åndedrætsorgan			2
DC784;Fjernmetastase i tyndtarmen			2
DC784A;Fjernmetastase i duodenum			2
DC785;Fjernmetastase i tyktarmen eller endetarmen			2
DC785A;Fjernmetastase i tyktarmen			2
DC785B;Fjernmetastase i endetarmen			2
DC786;Metastase i retroperitonale rum eller i peritoneum			2
DC786A;Malign ascites UNS			2
DC786B;Metastase i peritoneum			2
DC786C;Metastase i retroperitoneale rum			2
DC786D;Carcinomatosis peritonei			2
DC786E;Pseudomyxoma peritonei			2
DC787;Fjernmetastase i leveren			2
DC788;Fjernmetastase med anden eller ikke specificeret lokalisation i fordøjelsesorgan			2
DC79;Metastaser i andre specificerede lokalisationer			2
DC790;Fjernmetastase i nyre eller nyrebækken			2
DC790A;Fjernmetastase i nyrebækken			2
DC790B;Fjernmetastase i nyre			2
DC791;Fjernmetastase i andet urinorgan eller mandligt kønsorgan			2
DC791I;Fjernmetastase i urinblæren			2
DC791J;Fjernmetastase i prostata			2
DC791S;Fjernmetastase i penis			2
DC791T;Fjernmetastase i testikel			2
DC791U;Fjernmetastase i urinleder			2
DC791V;Fjernmetastase i urinrøret			2
DC792;Fjernmetastase i huden			2
DC793;Fjernmetastase i hjernen eller hjernehinder			2

DC793A;Fjernmetastase i hjernen			2
DC793B;Fjernmetastase i hjernehinde			2
DC793C;Fjernmetastase i rygmarvshinde			2
DC794;Fjernmetastase med anden eller ikke specificeret lokalisation i nervesystemet			2
DC795;Fjernmetastase i knogle eller knoglemarven			2
DC795A;Fjernmetastase i knoglemarven			2
DC795B;Fjernmetastase i knogle			2
DC795C;Fjernmetastase i ryghvirvel			2
DC795D;Fjernmetastase i ribben			2
DC795E;Fjernmetastase i kraniet			2
DC796;Fjernmetastase i æggestok			2
DC797;Fjernmetastase i binyre			2
DC798;Metastase UNS			2
DC798A;Carcinomatosis UNS			2
DC798C;Fjernmetastaser og regionale lymfeknudemetastaser UNS			2
DC798D;Fjernmetastase UNS			2
DC798E;In-transit metastase i hud			2
DC798F;Fjernmetastaser i multiple lokalisationer			2
DC798Z;Fjernmetastaser i anden specificeret lokalisation IKA			2
DC799;Sekundær kræftsygdom UNS			2
DC80;Ikke nærmere specificeret kræft (ukendt primærtumor)			2
DC800;Primær kræftsygdom uden kendt lokalisation			2
DC809;Kræftsygdom UNS			2
DC809A;Malign kakeksi			2
DC81;Hodgkin lymfomer			2
DC810;Nodulært lymfocytdomineret Hodgkin lymfom			2
DC811;Klassisk Hodgkin lymfom med nodulær sklerose			2
DC812;Klassisk Hodgkin lymfom med blandet cellularitet			2
DC813;Klassisk lymfocytfattigt Hodgkin lymfom			2
DC814;Klassisk lymfocytrigt Hodgkin lymfom			2
DC817;Andet klassisk Hodgkin lymfom			2
DC819;Hodgkin lymfom UNS			2

DC82;Follikulære lymfomer			2
DC820;Follikulært lymfom, grad I			2
DC821;Follikulært lymfom, grad II			2
DC822;Follikulært lymfom, grad III			2
DC825;Diffust follikelcenter lymfom			2
DC826;Kutant follikelcenter lymfom			2
DC827;Andet follikulært lymfom			2
DC829;Follikulært lymfom UNS			2
DC83;Ikke-follikulære lymfomer			2
DC830;Småcellet B-celle lymfom			2
DC830B;Lymfoplasmacytært lymfom			2
DC830C;Nodalt marginalzonelymfom			2
DC830D;Splenisk marginalzonelymfom			2
DC831;Mantle celle lymfom (MCL)			2
DC831A;Centrocytisk lymfom			2
DC831B;Malign lymfomatøs polypose			2
DC833;Diffust storcellet B-celle lymfom			2
DC833A;Anaplastisk diffust storcellet B-celle lymfom			2
DC833B;CD30-positivt diffust storcellet B-celle lymfom			2
DC833C;Centroblastært diffust storcellet B-celle lymfom			2
DC833D;Immunoblastært diffust storcellet B-celle lymfom			2
DC833E;Plasmablastært diffust storcellet B-celle lymfom			2
DC833F;T-cellerigt diffust storcellet B-celle lymfom			2
DC835;Lymfoblastært lymfom			2
DC835A;Lymfoblastært B-celle lymfom			2
DC835B;Lymfoblastært T-celle lymfom			2
DC835C;Lymfoblastært lymfom UNS			2
DC837;Burkitt lymfom			2
DC837A;Atypisk Burkitt lymfom			2
DC837B;Burkitt-lignende lymfom			2
DC838;Andet ikke-follikulært lymfom			2
DC838A;Primært effusionslymfom (af B-celle type)			2

DC838B;Intravaskulært storcellet B-celle lymfom			2
DC839;Ikke-follikulært (diffust) lymfom UNS			2
DC84;Modne NK/T-celle lymfomer			2
DC840;Mycosis fungoides			2
DC840A;Mycosis fungoides associeret med follikulær mucinose			2
DC840B;Pagetoid retikulose			2
DC840C;Granulomatøs slack skin (lymphoma malignum)			2
DC841;Sézarys sygdom			2
DC844;Perifert T-celle lymfom UNS			2
DC844F;Lymfoepitelioidt lymfom			2
DC844H;Modent T-celle lymfom UNS			2
DC845;Andet modent NK/T-celle lymfom			2
DC846;Anaplastisk storcellet lymfom, ALK-positivt			2
DC846A;Anaplastisk storcellet lymfom, CD30-positivt			2
DC847;Anaplastisk storcellet lymfom, ALK-negativt			2
DC848;Kutant T-celle lymfom UNS			2
DC849;Modent NK/T-celle lymfom UNS			2
DC85;Andre og ikke specificerede non-Hodgkin lymfomer			2
DC851;B-celle lymfom UNS			2
DC852;Mediastinalt (tymisk) storcellet B-celle lymfom			2
DC857;Andet non-Hodgkin lymfom			2
DC857B;Polymorf posttransplantations lymfoproliferativ sygdom (PTLD)			2
DC859;Lymfom (neoplasi) UNS			2
DC86;Andre NK/T-celle lymfomer			2
DC860;Ekstranodalt NK/T-celle lymfom, nasal type			2
DC861;Hepatosplenisk T-celle lymfom			2
DC862;Enteropati-type (intestinalt) T-celle lymfom			2
DC863;Subkutant panniculitis-lignende T-celle lymfom			2
DC864;Blastisk NK-celle lymfom			2
DC865;Angioimmunoblastært T-celle lymfom			2
DC866;Primært kutane CD30-positive T-celle proliferationer			2
DC866B;Primært kutant anaplastisk storcellet lymfom			2

DC866C;Primært kutant CD30-positivt storcellet lymfom			2
DC88;Maligne immunoproliferative sygdomme			2
DC880;Waldenströms makroglobulinæmi			2
DC882;Anden heavy chain disease			2
DC882A;Gamma heavy chain disease			2
DC882B;My heavy chain disease			2
DC883;Immunoproliferativ tyndtarmssygdom			2
DC883A;Alpha heavy chain disease			2
DC883B;Middelhavs lymfom			2
DC884;Ekstranodalt marginalzone B-celle lymfom			2
DC884A;Lymfom af mucosa-associeret lymfoidt væv (MALT-lymfom)			2
DC884B;Lymfom af hud-associeret lymfoidt væv (SALT-lymfom)			2
DC884C;Lymfom af bronkie-associeret lymfoidt væv (BALT-lymfom)			2
DC887;Anden malign immunoproliferativ sygdom			2
DC889;Malign immunoproliferativ sygdom UNS			2
DC90;Maligne plasmacelle neoplasier			2
DC900;Myelomatose			2
DC901;Plasmacelle leukæmi			2
DC902;Solitært ikke-ossøst plasmacytom			2
DC903;Solitært ossøst plasmacytom			2
DC91;Lymfatiske leukæmier			2
DC910;Akut lymfoblastær leukæmi (ALL)			2
DC911;Kronisk lymfatisk leukæmi af B-celle type (B-CLL)			2
DC911A;Lymfoplasmacytær leukæmi			2
DC911B;Richter syndrom			2
DC913;Prolymfocyt leukæmi af B-celle type			2
DC914;Hårcelle leukæmi			2
DC915;Adult T-celle lymfom/leukæmi (HTLV-1-associeret)			2
DC915A;Adult T-celle lymfom/leukæmi (HTLV-1-associeret), akut variant			2
DC915B;Adult T-celle lymfom/leukæmi (HTLV-1-associeret), kronisk variant			2
DC915C;Adult T-celle lymfom/leukæmi (HTLV-1-associeret), lymfomatoid variant			2
DC915D;Adult T-celle lymfom/leukæmi (HTLV-1-associeret), smouldering variant			2

Supplement 2 Nielsen et al. Mortality after paediatric emergency calls for patients with and without pre-existing comorbidity (2023)

DC916;Prolymfocyt leukæmi af T-celle type			2
DC917;Anden lymfatisk leukæmi			2
DC917B;Large granular T-celle lymfocytær leukæmi			2
DC918;Moden B-celle leukæmi af Burkitt-type			2
DC919;Lymfatisk leukæmi UNS			2
DC92;Myeloide leukæmier			2
DC920;Akut myeloblastær leukæmi (AML)			2
DC920A;Akut myeloblastær leukæmi med minimal differentiering			2
DC920B;Akut myeloblastær leukæmi med modning			2
DC920C;Akut myeloblastær leukæmi, AML1/ETO			2
DC920D;Akut myeloblastær leukæmi, AML M0			2
DC920E;Akut myeloblastær leukæmi, AML M1			2
DC920F;Akut myeloblastær leukæmi, AML M2			2
DC920G;Akut myeloblastær leukæmi, AML med t(8;21)			2
DC920H;Akut myeloblastær leukæmi UNS (uden FAB-klassificering)			2
DC921;Kronisk myeloid leukæmi (CML), BCR/ABL-positiv			2
DC921A;Kronisk myeloid leukæmi med blastkrise			2
DC922;Atypisk kronisk myeloid leukæmi, BCR/ABL-negativ			2
DC923;Myeloidt sarkom			2
DC923A;Chloroma			2
DC923B;Granulocytært sarkom			2
DC924;Akut myeloblastær leukæmi, AML M3			2
DC924A;Akut myeloblastær leukæmi M3 med t(15;17) og varianter			2
DC925;Akut myeloblastær leukæmi, AML M4			2
DC925A;Akut myeloblastær leukæmi M4 Eo med inv(16) eller t(16;16)			2
DC926;Akut myeloblastær leukæmi med 11q23-abnormalitet			2
DC926A;Akut myeloblastær leukæmi med variation af MLL-gen			2
DC927;Anden myeloid leukæmi			2
DC927B;Kronisk neutrofil leukæmi			2
DC928;Akut myeloid leukæmi med multilinje dysplasi			2
DC929;Myeloid leukæmi UNS			2
DC93;Monocytære leukæmier			2

DC930;Akut monoblastær leukæmi, AML M5			2
DC930A;Akut monoblastær, AML M5a			2
DC930B;Akut monoblastær, AML M5b			2
DC931;Kronisk myelomonocytær leukæmi, CMML			2
DC931A;Kronisk myelomonocytær leukæmi, CMML-1			2
DC931B;Kronisk myelomonocytær leukæmi, CMML-2			2
DC931C;Kronisk myelomonocytær, CMML med eosinofili			2
DC933;Juvenil myelomonocytær leukæmi			2
DC937;Anden monocytær leukæmi			2
DC939;Monocytær leukæmi UNS			2
DC94;Andre leukæmier af specificerede celletyper			2
DC940;Akut erytroid leukæmi, M6 (a)(b)			2
DC940A;Erytroleukæmi UNS			2
DC942;Akut megakaryoblastær leukæmi, M7			2
DC943;Mastcelle leukæmi			2
DC944;Akut myelofibrose			2
DC946;Uklassificerbar myelodysplasi/myeloproliferativ sygdom			2
DC947;Anden leukæmi			2
DC947B;Aggressiv NK-celle leukæmi			2
DC947C;Akut basofil leukæmi			2
DC95;Leukæmier af ikke specificeret celletype			2
DC950;Akut leukæmi af ikke specificeret celletype			2
DC950C;Akut bilineær leukæmi			2
DC950D;Akut leukæmi af blandet linearitet			2
DC951;Kronisk leukæmi af ikke specificeret celletype			2
DC957;Anden leukæmi af ikke specificeret celletype			2
DC959;Leukæmi UNS			2
DC96;Andre og ikke specificerede maligne neoplasier fra lymfoidt og hæmatopoietisk væv			2
DC960;Multifokal og multisystemisk (dissemineret) Langerhans-celle histiocytosis			2
DC960A;Histiocytosis X, multisystemisk			2
DC962;Malign mastcelle tumor			2
DC962B;Mastcelle sarkom			2

DC964;Dendritcelle sarkom (accessoriske celler)			2
DC964A;Interdigiterende dendritcelle sarkom			2
DC964B;Langerhans-celle sarkom			2
DC964C;Follikulært dendritcelle sarkom			2
DC965;Multifokal og unisystemisk Langerhans-celle histiocytose			2
DC965A;Hand-Schüller-Christian sygdom			2
DC965B;Histiocytosis X, multifokal			2
DC966;Unifokal Langerhans-celle histiocytose			2
DC966A;Eosinofilt granulom			2
DC966B;Histiocytosis X, unifokal			2
DC966C;Histiocytosis X UNS			2
DC966D;Langerhans-celle histiocytose UNS			2
DC967;Anden malign neoplasi fra lymfoidt eller hæmatopoietisk væv			2
DC967A;Histiocytært sarkom			2
DC968;Malign histiocytose UNS			2
DC969;Malign neoplasi fra lymfoidt eller hæmatopoietisk væv UNS			2
DC99;Kliniske og parakliniske fund ved kræftsygdom			2
DC991;Lokalrecidiv UNS			2
DC991A;Lokalrecidiv i efterladt væv			2
DC991B;Lokalrecidiv i marginalzone			2
DC991C;Lokalrecidiv i hud			2
DC991D;Lokalrecidiv i slimhinde			2
DC991E;Lokalrecidiv i bindevæv			2
DC991Y;Lokalrecidiver i multiple lokalisationer			2
DD00;Carcinoma in situ på læber, i mundhulen, spiserøret og mavesækken		1	
DD000;Carcinoma in situ på læbe, i mundhulen eller svælget		1	
DD000A;Carcinoma in situ i mundhulen		1	
DD000B;Carcinoma in situ på læbe		1	
DD000C;Carcinoma in situ i svælget		1	
DD001;Carcinoma in situ i spiserøret		1	
DD002;Carcinoma in situ i mavesækken		1	
DD01;Carcinoma in situ i andre og ikke specificerede fordøjelsesorganer		1	

DD010;Carcinoma in situ i tyktarmen		1	
DD012;Carcinoma in situ i endetarmen		1	
DD013;Carcinoma in situ i endetarmsåbningen eller analkanalen		1	
DD013A;Carcinoma in situ i endetarmsåbningen		1	
DD013B;Carcinoma in situ i analkanalen		1	
DD014;Carcinoma in situ i anden eller ikke specificeret del af tarmen		1	
DD014A;Carcinoma in situ i tyndtarmen		1	
DD015;Carcinoma in situ i leveren, galdeblæren eller galdeveje		1	
DD015A;Carcinoma in situ ampullae Vateri		1	
DD015B;Carcinoma in situ i leveren		1	
DD015C;Carcinoma in situ i galdeblæren		1	
DD015D;Carcinoma in situ i galdeveje		1	
DD017;Carcinoma in situ i fordøjelsesorganer med anden lokalisation		1	
DD017A;Carcinoma in situ i pancreas		1	
DD019;Carcinoma in situ i fordøjelseskanalen UNS		1	
DD02;Carcinoma in situ i mellemøre og åndedrætsorganer		1	
DD020;Carcinoma in situ i strubehovedet		1	
DD021;Carcinoma in situ i luftrøret		1	
DD022;Carcinoma in situ i bronkie eller lunge		1	
DD022A;Carcinoma in situ i bronkie		1	
DD022B;Carcinoma in situ i lunge		1	
DD023;Carcinoma in situ med anden lokalisation i åndedrætsorganer		1	
DD023A;Carcinoma in situ i mellemøre		1	
DD023B;Carcinoma in situ i næsehulen		1	
DD023C;Carcinoma in situ i bihule		1	
DD024;Carcinoma in situ i åndedrætsorgan UNS		1	
DD03;Melanoma in situ		1	
DD030;Melanoma in situ på læbe		1	
DD031;Melanoma in situ på øjenlåg		1	
DD031A;Melanoma in situ canthi oculi		1	
DD032;Melanoma in situ på øre eller i ydre øregang		1	
DD032A;Melanoma in situ på øre		1	

DD032B;Melanoma in situ i ydre øregang		1	
DD033;Melanoma in situ i ansigtet med anden eller ikke specificeret lokalisation		1	
DD033A;Melanoma in situ på næsen		1	
DD034;Melanoma in situ på skalpen eller halsen		1	
DD034A;Melanoma in situ på skalpen		1	
DD034B;Melanoma in situ på halsen		1	
DD035;Melanoma in situ på kroppen		1	
DD035A;Melanoma in situ (i hud) ved endetarmsåbningen		1	
DD035B;Melanoma in situ på bryst		1	
DD035C;Melanoma in situ i marginalzonen i endetarmsåbningen		1	
DD035G;Melanoma in situ i vulva		1	
DD036;Melanoma in situ på overekstremitet		1	
DD036A;Melanoma in situ på skulder		1	
DD036B;Melanoma in situ på arm		1	
DD037;Melanoma in situ på underekstremitet		1	
DD037A;Melanoma in situ på ben		1	
DD037B;Melanoma in situ på hofte		1	
DD038;Melanoma in situ med anden lokalisation		1	
DD039;Melanoma in situ UNS		1	
DD04;Carcinoma in situ i huden		1	
DD040;Carcinoma in situ i hud på læbe		1	
DD041;Carcinoma in situ i hud på øjenlåg		1	
DD041A;Carcinoma in situ i hud i øjenkrog		1	
DD042;Carcinoma in situ i hud på øre eller i ydre øregang		1	
DD042A;Carcinoma in situ i hud på øre		1	
DD042B;Carcinoma in situ i hud i ydre øregang		1	
DD043;Carcinoma in situ i hud i ansigtet med anden eller ikke specificeret lokalisation		1	
DD043A;Carcinoma in situ i hud i ansigtet med anden lokalisation		1	
DD043B;Carcinoma in situ i hud i ansigtet UNS		1	
DD043C;Carcinoma in situ i hud på næsen		1	
DD044;Carcinoma in situ i hud på skalpen eller halsen		1	
DD044A;Carcinoma in situ i hud på skalpen		1	

DD044B;Carcinoma in situ i hud på halsen		1	
DD045;Carcinoma in situ i hud på kroppen		1	
DD045A;Carcinoma in situ i hud ved endetarmsåbningen		1	
DD045B;Carcinoma in situ i hud på bryst		1	
DD045C;Carcinoma in situ i marginalzonen i endetarmsåbningen		1	
DD046;Carcinoma in situ i hud på overekstremitet		1	
DD046A;Carcinoma in situ i hud på skulder		1	
DD046B;Carcinoma in situ i hud på arm		1	
DD047;Carcinoma in situ i hud på underekstremitet		1	
DD047A;Carcinoma in situ i hud på ben		1	
DD047B;Carcinoma in situ i hud på hofte		1	
DD048;Carcinoma in situ i huden med anden lokalisation		1	
DD049;Carcinoma in situ i huden UNS		1	
DD05;Carcinoma in situ i bryst		1	
DD050;Lobulært carcinoma in situ i mamma		1	
DD051;Intraduktalt carcinoma in situ i mamma		1	
DD056;Pagets sygdom i bryst (brystvorte)		1	
DD057;Carcinoma in situ med anden lokalisation i mamma		1	
DD059;Carcinoma in situ i mamma UNS		1	
DD06;Carcinoma in situ i livmoderhalsen		1	
DD069;Carcinoma in situ i livmoderhalsen UNS		1	
DD07;Carcinoma in situ med andre og ikke specificerede lokalisationer i kønsorganer		1	
DD070;Endometriehyperplasi med atypi		1	
DD071;Carcinoma in situ i vulva		1	
DD071A;Vulvær intraepitelial neoplasi (VIN), grad III		1	
DD072;Carcinoma in situ i vagina		1	
DD072A;Vaginal intraepitelial neoplasi (VAIN), grad III		1	
DD073;Carcinoma in situ med anden eller ikke specificeret lokalisation i kvindelige kønsorganer		1	
DD073A;Carcinoma in situ i æggestok		1	
DD073B;Carcinoma in situ med anden lokalisation i kvindeligt kønsorgan		1	
DD073B1;Tubalt intraepithelialt karcinom		1	
DD073C;Carcinoma in situ i kvindeligt kønsorgan UNS		1	

DD074;Carcinoma in situ på penis		1	
DD075;Carcinoma in situ i prostata		1	
DD076;Carcinoma in situ med anden eller ikke specificeret lokalisation i mandlige kønsorganer		1	
DD076A;Carcinoma in situ med anden lokalisation i mandligt kønsorgan		1	
DD076B;Carcinoma in situ i mandligt kønsorgan UNS		1	
DD076T;Carcinoma in situ i testikel		1	
DD09;Carcinoma in situ med andre eller ikke specificerede lokalisationer		1	
DD090;Carcinoma in situ (Tis) i urinblæren		1	
DD091;Carcinoma in situ (Tis) med anden eller ikke specificeret lokalisation i urinveje		1	
DD091A;Carcinoma in situ i nyre		1	
DD091B;Carcinoma in situ i nyrebækken		1	
DD091C;Carcinoma in situ i urinleder		1	
DD091D;Carcinoma in situ i urinrør		1	
DD092;Carcinoma in situ i øje		1	
DD093;Carcinoma in situ i endokrin kirtel		1	
DD093A;Carcinoma in situ i skjoldbruskkirtlen		1	
DD093B;Carcinoma in situ i anden endokrin kirtel		1	
DD095;Non-invasiv papillær tumor (Ta) i urinblæren		1	
DD096;Non-invasiv papillær tumor (Ta) i anden eller ikke specificeret lokalisation i urinveje		1	
DD096B;Non-invasiv papillær tumor (Ta) i nyrebækken		1	
DD096C;Non-invasiv papillær tumor (Ta) i urinleder		1	
DD096D;Non-invasiv papillær tumor (Ta) i urinrøret		1	
DD097;Carcinoma in situ med anden lokalisation		1	
DD097A;Carcinoma in situ på næsen UNS		1	
DD099;Carcinoma in situ UNS		1	
DD10;Godartede tumorer i læber, mundhulen og svælget		1	
DD100;Godartet tumor i læbe		1	
DD101;Godartet tumor i tungen		1	
DD101A;Godartet tumor i tonsilla lingualis		1	
DD102;Godartet tumor i mundgulvet		1	
DD103;Godartet tumor i mundhulen med anden eller ikke specificeret lokalisation		1	
DD103A;Godartet tumor i munden UNS		1	

Supplement 2 Nielsen et al. Mortality after paediatric emergency calls for patients with and without pre-existing comorbidity (2023)

DD103B;Papillomatosis mucosae facies oralis palati		1		
DD104;Godartet tumor i tonsil		1		
DD104A;Godartet tumor i tonsilla palatina		1		
DD105;Godartet tumor med anden lokalisation i mundsvælget		1		
DD106;Godartet tumor i næsesvælget		1		
DD106A;Juvenilt angiofibrom i rhinopharynx		1		
DD107;Godartet tumor i hypopharynx		1		
DD109;Godartet tumor i svælget UNS		1		
DD11;Godartede tumorer i store spytkirtler		1		
DD110;Godartet tumor i ørespytkirtel		1		
DD117;Godartet tumor i anden stor spytkirtel		1		
DD117A;Godartet tumor i glandula sublingualis		1		
DD117B;Godartet tumor i glandula submandibularis		1		
DD119;Godartet tumor i stor spytkirtel UNS		1		
DD12;Godartede tumorer i tyktarmen, endetarmen og endetarmsåbningen		1		
DD120;Godartet tumor i caecum		1		
DD120A;Godartet tumor i valvula ileocaecalis		1		
DD121;Godartet tumor i blindtarmen		1		
DD122;Godartet tumor i colon ascendens		1		
DD123;Godartet tumor i colon transversum		1		
DD123A;Godartet tumor i flexura coli dextra		1		
DD123B;Godartet tumor i flexura coli sinistra		1		
DD124;Godartet tumor i colon descendens		1		
DD125;Godartet tumor i colon sigmoideum		1		
DD126;Godartet tumor i tyktarmen uden nærmere specificeret lokalisation		1		
DD126A;Adenomatosis coli		1		
DD126B;Polyposis hereditaria coli		1		
DD126C;Multiple godartede tumorer i tyktarmen		1		
DD126F;Familiær adenomatøs polypose (FAP)		1		
DD128;Godartet tumor i endetarmen		1		
DD129;Godartet tumor i endetarmsåbningen eller analkanalen		1		
DD129A;Godartet tumor i endetarmsåbningen		1		

Supplement 2 Nielsen et al. Mortality after paediatric emergency calls for patients with and without pre-existing comorbidity (2023)

DD129B;Godartet tumor i analkanalen		1	
DD13;Godartede tumorer i andre og dårligt specificerede dele af fordøjelsessystemet		1	
DD130;Godartet tumor i spiserøret		1	
DD131;Godartet tumor i mavesækken		1	
DD132;Godartet tumor i tolvfingertarmen		1	
DD133;Godartet tumor i anden eller ikke specificeret del af tyndtarmen		1	
DD134;Godartet tumor i leveren		1	
DD134A;Godartet tumor i intrahepatiske galdegange		1	
DD135;Godartet tumor i ekstrahepatiske galdeveje		1	
DD136;Godartet tumor i pancreas		1	
DD137;Godartet tumor i Langerhanske øer		1	
DD137A;Insulinom		1	
DD137B;Glucagonom		1	
DD139;Godartet tumor i fordøjelsessystemet UNS		1	
DD139A;Godartet tumor i milten UNS		1	
DD14;Godartede tumorer i mellemøre og åndedrætsorganer		1	
DD140;Godartet tumor i mellemøre, næsehulen eller bihule		1	
DD140A;Godartet tumor i mellemøre		1	
DD140B;Godartet tumor i næsehulen		1	
DD140C;Godartet tumor i bihule		1	
DD140D;Godartet tumor i næsebrusken		1	
DD141;Godartet tumor i strubehovedet		1	
DD142;Godartet tumor i luftrøret		1	
DD143;Godartet tumor i bronkie eller lunge		1	
DD143A;Godartet tumor i bronkie		1	
DD143B;Godartet tumor i lunge		1	
DD144;Godartet tumor i åndedrætsorgan UNS		1	
DD15;Godartede tumorer i andre og dårligt specificerede lokalisationer i brysthulen		1	
DD150;Godartet tumor i thymus		1	
DD151;Godartet tumor i hjertet		1	
DD152;Godartet tumor i mediastinum		1	
DD157;Godartet tumor i andet organ i brysthulen		1	

Supplement 2 Nielsen et al. Mortality after paediatric emergency calls for patients with and without pre-existing comorbidity (2023)

DD159;Godartet tumor i brysthulen UNS		1	
DD16;Godartede tumorer i knogle og ledbrusk		1	
DD160;Godartet tumor i knogle eller ledbrusk i skulderblad eller lang knogle i overekstremitet		1	
DD160C;Godartet tumor i knogle eller ledbrusk i lang knogle i overekstremitet		1	
DD160D;Godartet tumor i knogle eller ledbrusk i skulderblad		1	
DD161;Godartet tumor i knogle eller ledbrusk i kort knogle i overekstremitet		1	
DD162;Godartet tumor i knogle eller ledbrusk i lang knogle i underekstremitet		1	
DD163;Godartet tumor i knogle eller ledbrusk i kort knogle i underekstremitet		1	
DD163C;Eksostose under tånegl		1	
DD164;Godartet tumor i knogle eller ledbrusk i kranie eller ansigt		1	
DD164C;Godartet tumor i knogle eller ledbrusk i kranieknogle		1	
DD164D;Godartet tumor i knogle eller ledbrusk i ansigtsknogle		1	
DD164E;Godartet tumor i knogle eller ledbrusk i overkæben		1	
DD165;Godartet tumor i knogle eller ledbrusk i underkæben		1	
DD166;Godartet tumor i knogle eller ledbrusk i rygsøjlen		1	
DD167;Godartet tumor i knogle eller ledbrusk i ribben, brystben eller clavicula		1	
DD167D;Godartet tumor i knogle eller ledbrusk i clavicula		1	
DD167E;Godartet tumor i knogle eller ledbrusk i ribben		1	
DD167F;Godartet tumor i knogle eller ledbrusk i brystbenet		1	
DD168;Godartet tumor i knogle eller ledbrusk i bækken, os sacrum eller os coccygis		1	
DD168D;Godartet tumor i knogle eller ledbrusk i os coccygis		1	
DD168E;Godartet tumor i knogle eller ledbrusk i bækkenet		1	
DD168F;Godartet tumor i knogle eller ledbrusk i os sacrum		1	
DD169;Godartet tumor i knogle eller ledbrusk UNS		1	
DD17;Lipomer		1	
DD170;Lipom i hud eller underhud på hoved, ansigt eller hals		1	
DD170A;Lipom i hud på hovedet		1	
DD170B;Lipom i hud på halsen		1	
DD170C;Lipom i hud på ansigtet		1	
DD170D;Lipom i underhud på hovedet		1	
DD170E;Lipom i underhud på halsen		1	
DD170F;Lipom i underhud på ansigtet		1	

DD171;Lipom i hud eller underhud på kroppen		1	
DD171C;Lipom i hud eller underhud på mamma		1	
DD172;Lipom i hud eller underhud på ekstremitet		1	
DD173;Lipom i hud eller underhud med anden eller ikke specificeret lokalisation		1	
DD174;Lipom i intratorakalt organ		1	
DD175;Lipom i intraabdominalt organ		1	
DD176;Lipom i sædstrengen		1	
DD177;Lipom med anden lokalisation		1	
DD177A;Peritonealt lipom		1	
DD177B;Retroperitonealt lipom		1	
DD177C;Lipom i mamma		1	
DD179;Lipom UNS		1	
DD18;Godartede tumorer i blodkar og lymfekar		1	
DD180;Hæmangiom		1	
DD180A;Intrakutant hæmangiom		1	
DD180B;Blue rubber bleb-nævus		1	
DD180C;Kimuras sygdom		1	
DD180D;Diffust hæmangiom		1	
DD180E;Kasabach-Merritt syndrom		1	
DD180F;Kapillært hæmangiom		1	
DD181;Lymfangiom		1	
DD181A;Hygroma		1	
DD181B;Lymfhæmangiom		1	
DD189;Godartet tumor i blodkar eller lymfekar UNS		1	
DD19;Godartede tumorer i mesotelialt væv		1	
DD190;Godartet mesoteliom i lungehinde		1	
DD191;Godartet mesoteliom i peritoneum		1	
DD197;Godartet tumor i mesotelialt væv med anden lokalisation		1	
DD199;Godartet tumor i mesotelialt væv UNS		1	
DD20;Godartede tumorer i bindevæv i peritoneum og retroperitoneum		1	
DD200;Godartet tumor i bindevæv i retroperitoneum		1	
DD201;Godartet tumor i bindevæv i peritoneum		1	

DD21;Andre godartede tumorer i bindevæv og andre bløddele		1	
DD210;Godartet tumor i bindevæv eller andre bløddele i hoved, ansigt eller hals		1	
DD210A;Godartet tumor i bindevæv i hovedet		1	
DD210B;Godartet tumor i bindevæv i halsen		1	
DD210C;Godartet tumor i bindevæv i ansigtet		1	
DD211;Godartet tumor i bindevæv i overekstremitet		1	
DD211A;Godartet tumor i bindevæv i skulder		1	
DD212;Godartet tumor i bindevæv i underekstremitet		1	
DD212B;Godartet tumor i bindevæv i hofte		1	
DD213;Godartet tumor i bindevæv i thorax		1	
DD214;Godartet tumor i bindevæv i abdomen		1	
DD215;Godartet tumor i bindevæv i bækkenet		1	
DD216;Godartet tumor i bindevæv i kroppen UNS		1	
DD216A;Godartet tumor i bindevæv på ryggen		1	
DD217;Godartet tumor i bindevæv med anden lokalisation		1	
DD219;Godartet tumor i bindevæv UNS		1	
DD22;Nævus		1	
DD220;Nævus på læbe		1	
DD221;Nævus på øjenlåg		1	
DD222;Nævus på øre eller i ydre øregang		1	
DD222A;Nævus på øre		1	
DD222B;Nævus i ydre øregang		1	
DD223;Nævus med anden eller ikke specificeret lokalisation i ansigtet		1	
DD224;Nævus på skalpen eller halsen		1	
DD224A;Nævus på skalpen		1	
DD224B;Nævus på halsen		1	
DD225;Nævus på kroppen		1	
DD225A;Nævus i anus		1	
DD225B;Nævus på mamma		1	
DD225C;Perianalt nævus		1	
DD226;Nævus på overekstremitet		1	
DD226A;Nævus på skulder		1	

DD227;Nævus på underekstremitet		1	
DD227A;Nævus på hofte		1	
DD228;Nævus med anden lokalisation		1	
DD229;Nævus UNS		1	
DD229Q;Nævus spilus		1	
DD23;Andre godartede tumorer i huden		1	
DD230;Godartet tumor i hud på læbe		1	
DD231;Godartet tumor i hud på øjenlåg		1	
DD231A;Godartet tumor i hud i øjenkrog		1	
DD232;Godartet tumor i hud på øre eller i ydre øregang		1	
DD232A;Godartet tumor i hud på øre		1	
DD232B;Godartet tumor i hud i ydre øregang		1	
DD233;Godartet tumor i hud i ansigtet med anden eller ikke specificeret lokalisation		1	
DD234;Godartet tumor i hud på skalpen eller halsen		1	
DD234A;Godartet tumor i hud på skalpen		1	
DD234B;Godartet tumor i hud på halsen		1	
DD235;Godartet tumor i hud på kroppen		1	
DD235A;Godartet tumor i hud i anus		1	
DD235B;Godartet tumor i hud på mamma		1	
DD235C;Perianal godartet tumor i huden		1	
DD235J;Neoplasma benignum cutis trunci, dermatofibrom (findes ikke i SKS)		1	
DD236;Godartet tumor i hud på overekstremitet		1	
DD236A;Godartet tumor i hud på skulder		1	
DD237;Godartet tumor i hud på underekstremitet		1	
DD237A;Godartet tumor i hud på hofte		1	
DD239;Godartet tumor i huden UNS		1	
DD24;Godartede tumorer i mamma		1	
DD249;Godartet tumor i mamma UNS		1	
DD249A;Fibroadenom i mamma		1	
DD249C;Papilloma ductuli mammae		1	
DD249D;Adenom i brystvorte		1	
DD249E;Benign phylloides tumor i bryst		1	

DD249F;Harmartom i mamma		1		
DD249G;Fibromatose i mamma		1		
DD249W;Anden godartet tumor i mamma		1		
DD25;Fibromyom i livmoderen		1		
DD250;Submukøst fibromyom i livmoderen		1		
DD250A;Submukøst fibromyom type 0 i livmoderen		1		
DD250B;Submukøst fibromyom type 1 i livmoderen		1		
DD250C;Submukøst fibromyom type 2 i livmoderen		1		
DD251;Intramuralt fibromyom i livmoderen		1		
DD252;Subserøst fibromyom i livmoderen		1		
DD259;Fibromyom i livmoderen UNS		1		
DD26;Andre godartede tumorer i livmoderen		1		
DD260;Godartet tumor i livmoderhalsen		1		
DD261;Godartet tumor i corpus uteri		1		
DD267;Godartet tumor i livmoderen med anden lokalisation		1		
DD269;Godartet tumor i livmoderen UNS		1		
DD27;Godartede tumorer i æggestok		1		
DD270;Serøst cystadenom i æggestok		1		
DD271;Mucinøst cystadenom i æggestok		1		
DD272;Dermoidcyste i æggestok		1		
DD278;Anden form for godartet tumor i æggestok		1		
DD279;Godartet tumor i æggestok UNS		1		
DD28;Godartede tumorer i andre og ikke specificerede kvindelige kønsorganer		1		
DD280;Godartet tumor i vulva		1		
DD281;Godartet tumor i vagina		1		
DD282;Godartet tumor i æggeleder eller livmoderens ligamenter		1		
DD282A;Godartet tumor i ligamentum lati		1		
DD282B;Godartet tumor i ligamentum rotundi		1		
DD282C;Godartet tumor i æggeleder		1		
DD287;Godartet tumor i andet kvindeligt kønsorgan		1		
DD289;Godartet tumor i kvindeligt kønsorgan UNS		1		
DD29;Godartede tumorer i mandlige kønsorganer		1		

Supplement 2 Nielsen et al. Mortality after paediatric emergency calls for patients with and without pre-existing comorbidity (2023)

DD290;Godartet tumor i penis		1	
DD291;Godartet tumor i prostata		1	
DD292;Godartet tumor i testikel		1	
DD293;Godartet tumor i epididymis		1	
DD294;Godartet tumor i scrotum		1	
DD297;Godartet tumor i mandlige kønsorganer med anden lokalisation		1	
DD297A;Godartet tumor i sædstreng		1	
DD297B;Godartet tumor i tunica vaginalis		1	
DD297C;Godartet tumor i vesicula seminalis		1	
DD299;Godartet tumor i mandligt kønsorgan UNS		1	
DD30;Godartede tumorer i nyrer og urinveje		1	
DD300;Godartet tumor i nyre		1	
DD301;Godartet tumor i nyrebækken		1	
DD302;Godartet tumor i urinleder		1	
DD303;Godartet tumor i urinblæren		1	
DD304;Godartet tumor i urinrøret		1	
DD307;Godartet tumor i urinveje med anden lokalisation		1	
DD309;Godartet tumor i nyre eller urinveje UNS		1	
DD31;Godartede tumorer i øje og omgivende strukturer		1	
DD310;Godartet tumor i konjunktiva		1	
DD311;Godartet tumor i hornhinde		1	
DD312;Godartet tumor i nethinde		1	
DD313;Godartet tumor i choroidea		1	
DD314;Godartet tumor i corpus ciliare		1	
DD315;Godartet tumor i tårekirtel eller tårekanal		1	
DD315A;Godartet tumor i tårekanal		1	
DD315B;Godartet tumor i tårekirtel		1	
DD315C;Godartet tumor i tåresæk		1	
DD316;Godartet tumor i øjenhule		1	
DD316A;Retrobulbær godartet tumor		1	
DD319;Godartet tumor i øje UNS		1	
DD32;Godartede tumorer i hjernehinder og rygmarvshinder		1	

DD320;Intrakranielt meningeom		1	
DD321;Intraspinalt meningeom		1	
DD329;Meningeom UNS		1	
DD33;Godartede tumorer i hjernen og andre dele af centralnervesystemet		1	
DD330;Supratentoriel godartet tumor i hjernen		1	
DD330A;Godartet tumor i hjernens pandelap		1	
DD330B;Godartet tumor i hjernens nakkelap		1	
DD330C;Godartet tumor i hjernens isselap		1	
DD330D;Godartet tumor i hjernens tindingelap		1	
DD331;Infratentoriel godartet tumor i hjernen		1	
DD331A;Godartet tumor i lillehjernen		1	
DD331B;Godartet tumor i hjernens 4. ventrikel		1	
DD331C;Godartet tumor i hjernestammen		1	
DD331D;Godartet tumor i hjernebroen		1	
DD332;Godartet tumor i hjernen UNS		1	
DD333;Godartet tumor i hjernenerve		1	
DD333A;Godartet tumor i bulbus olfactorius		1	
DD333B;Godartet tumor i hørenerve		1	
DD334;Godartet tumor i rygmarven		1	
DD337;Godartet tumor i anden del af centralnervesystemet		1	
DD339;Godartet tumor i centralnervesystemet UNS		1	
DD34;Godartede tumorer i skjoldbruskkirtlen		1	
DD349;Godartet tumor i skjoldbruskkirtlen UNS		1	
DD35;Godartede tumorer i andre og ikke specificerede endokrine kirtler		1	
DD350;Godartet tumor i binyre		1	
DD350A;Fæokromocytom		1	
DD351;Godartet tumor i biskjoldbruskkirtel		1	
DD352;Godartet tumor i hypofysen		1	
DD352A;Adenom i hypofysen		1	
DD353;Godartet tumor i ductus craniopharyngeus		1	
DD354;Godartet tumor i corpus pineale		1	
DD355;Godartet tumor i glomus caroticum		1	

Supplement 2 Nielsen et al. Mortality after paediatric emergency calls for patients with and without pre-existing comorbidity (2023)

DD356;Godartet tumor i corpus para-aorticus eller andet paraganglion		1	
DD356A;Godartet tumor i corpus para-aorticus		1	
DD356B;Godartet tumor i paraganglion		1	
DD356C;Godartet tumor i glomus jugulare		1	
DD357;Godartet tumor i anden endokrin kirtel		1	
DD358;Neoplasma benignum pluriglandulare		1	
DD359;Godartet tumor i endokrin kirtel UNS		1	
DD36;Godartede tumorer med anden eller ikke specificeret lokalisation		1	
DD360;Godartet tumor i lymfeknude		1	
DD361;Godartet tumor i perifer nerve eller det autonome nervesystem		1	
DD361A;Godartet tumor i perifer nerve		1	
DD361B;Godartet tumor i det autonome nervesystem		1	
DD367;Godartet tumor med anden lokalisation		1	
DD367A;Castleman sygdom		1	
DD369;Godartet tumor uden nærmere specificeret lokalisation		1	
DD37;Tumorer i mundhule og fordøjelsesorganer af usikker og ukendt karakter			2
DD370;Ikke specificeret tumor i læbe, mundhule eller svælg		1	
DD370A;Ikke specificeret tumor i mundhulen		1	
DD370B;Ikke specificeret tumor i læbe		1	
DD370C;Ikke specificeret tumor i svælget		1	
DD371;Ikke specificeret tumor i mavesækken			2
DD372;Ikke specificeret tumor i tyndtarmen			2
DD373;Ikke specificeret tumor i blindtarmen			2
DD374;Ikke specificeret tumor i tyktarmen			2
DD375;Ikke specificeret tumor i endetarmen			2
DD376;Ikke specificeret tumor i leveren, galdeblæren eller galdeveje			2
DD376A;Ikke specificeret tumor i leveren			2
DD376B;Ikke specificeret tumor i galdeblæren			2
DD376C;Ikke specificeret tumor i galdeveje			2
DD377;Ikke specificeret tumor i andet fordøjelsesorgan			2
DD377A;Ikke specificeret tumor i anus			2
DD377B;Ikke specificeret tumor i spiserøret			2

DD377C;Ikke specificeret tumor i pancreas			2
DD379;Ikke specificeret tumor i fordøjelsesorgan UNS			2
DD38;Tumorer i mellemøre, åndedrætsorganer og organer i brysthulen af usikker og ukendt karakter			2
DD380;Ikke specificeret tumor i strubehovedet			2
DD381;Ikke specificeret tumor i luftrør, bronkie eller lunge			2
DD381A;Ikke specificeret tumor i bronkie			2
DD381B;Ikke specificeret tumor i lunge			2
DD381C;Ikke specificeret tumor i luftrøret			2
DD382;Ikke specificeret tumor i lungehinde			2
DD383;Ikke specificeret tumor i mediastinum			2
DD384;Ikke specificeret tumor i thymus			2
DD385;Ikke specificeret tumor i andet åndedrætsorgan			2
DD385A;Ikke specificeret tumor i mellemøre			2
DD385B;Ikke specificeret tumor i næsebrusken			2
DD385C;Ikke specificeret tumor i næsehulen			2
DD385D;Ikke specificeret tumor i bihule			2
DD389;Ikke specificeret tumor i åndedrætsorgan eller luftveje UNS			2
DD39;Tumorer i kvindelige kønsorganer af usikker og ukendt karakter			2
DD390;Ikke specificeret tumor i livmoderen			2
DD391;Ikke specificeret tumor i æggestok			2
DD391A;Ovarietumor af borderline type			2
DD392;Ikke specificeret tumor i placenta			2
DD392A;Chorioadenoma destruens			2
DD392B;Mola hydatidosa maligna			2
DD397;Ikke specificeret tumor med anden lokalisation i kvindeligt kønsorgan			2
DD397A;Ikke specificeret hudtumor i kvindeligt kønsorgan			2
DD399;Ikke specificeret tumor i kvindeligt kønsorgan UNS			2
DD40;Tumorer i mandlige kønsorganer af usikker og ukendt karakter			2
DD400;Ikke specificeret tumor i prostata			2
DD401;Ikke specificeret tumor i testikel			2
DD407;Ikke specificeret tumor med anden lokalisation i mandligt kønsorgan			2
DD407A;Ikke specificeret hudtumor i mandligt kønsorgan			2

DD409;Ikke specificeret tumor i mandligt kønsorgan UNS			2
DD41;Tumorer i nyre og urinveje af usikker og ukendt karakter			2
DD410;Ikke specificeret tumor i nyre			2
DD411;Ikke specificeret tumor i nyrebækken			2
DD412;Ikke specificeret tumor i urinleder			2
DD413;Ikke specificeret tumor i urinrøret			2
DD414;Ikke specificeret tumor i urinblæren			2
DD417;Ikke specificeret tumor med anden lokalisation i urinveje			2
DD419;Ikke specificeret tumor med ikke nærmere specificeret lokalisation i nyre eller urinveje			2
DD42;Tumorer i hjerne- og rygmarvshinder af usikker og ukendt karakter			2
DD420;Ikke specificeret tumor i hjernehinde			2
DD421;Ikke specificeret tumor i rygmarvshinde			2
DD429;Ikke specificeret tumor med ikke nærmere specificeret lokalisation i hjerne- eller rygmarvshinde			2
DD43;Tumorer i hjerne og hjernenerver af usikker og ukendt karakter			2
DD430;Supratentoriel ikke specificeret tumor i hjernen			2
DD430B;Ikke specificeret tumor i hjernens frontallap			2
DD430C;Ikke specificeret tumor i hjernens nakkelap			2
DD430D;Ikke specificeret tumor i hjernens isselap			2
DD430E;Ikke specificeret tumor i hjernens tindingelap			2
DD430F;Ikke specificeret tumor i hjerneventrikel			2
DD431;Infratentoriel ikke specificeret tumor i hjernen			2
DD431G;Ikke specificeret tumor i hjernestammen			2
DD431H;Ikke specificeret tumor i lillehjernen			2
DD431J;Ikke specificeret tumor i hjernens 4. ventrikel			2
DD432;Ikke specificeret tumor med anden eller ikke nærmere specificeret lokalisation i hjerne			2
DD433;Ikke specificeret tumor i hjernenerve			2
DD434;Ikke specificeret tumor i rygmarven			2
DD437;Ikke specificeret tumor med anden lokalisation i centralnervesystemet			2
DD439;Ikke specificeret tumor i ikke nærmere specificeret lokalisation i centralnervesystemet			2
DD44;Tumorer i endokrine kirtler af usikker og ukendt karakter			2
DD440;Ikke specificeret tumor i skjoldbruskkirtlen			2
DD441;Ikke specificeret tumor i binyre			2

Supplement 2 Nielsen et al. Mortality after paediatric emergency calls for patients with and without pre-existing comorbidity (2023)

DD442;Ikke specificeret tumor i biskjoldbruskkirtel			2
DD443;Ikke specificeret tumor i hypofysen			2
DD444;Ikke specificeret tumor i ductus craniopharyngeus			2
DD445;Ikke specificeret tumor i corpus pinealis			2
DD446;Ikke specificeret tumor i glomus caroticum			2
DD447;Ikke specificeret tumor i corpus para-aorticus eller andet paraganglion			2
DD447A;Ikke specificeret tumor i corpus para-aorticus			2
DD447B;Ikke specificeret tumor i andet paraganglion			2
DD448;Ikke specificeret tumor i flere endokrine kirtler			2
DD448A;Multipel endokrin adenomatose			2
DD449;Ikke specificeret tumor i endokrin kirtel UNS			2
DD45;Polycythaemia vera			2
DD459;Polycythaemia vera			2
DD46;Myelodysplastiske syndromer			2
DD460;Refraktær cytopeni med unilineær dysplasi			2
DD460A;Refraktær anæmi uden sideroblaster			2
DD460B;Refraktær neutropeni			2
DD460C;Refraktær trombopeni			2
DD461;Refraktær anæmi med ringsideroblaster			2
DD462;Refraktær anæmi med overskud af blastceller			2
DD462A;Refraktær anæmi med overskud af blastceller, RAEB I			2
DD462B;Refraktær anæmi med overskud af blastceller, RAEB II			2
DD464;Refraktær anæmi UNS			2
DD465;Refraktær anæmi med multilinje dysplasi			2
DD466;Myelodysplastisk syndrom med isoleret del(5q) kromosomabnormitet			2
DD467;Andet myelodysplastisk syndrom			2
DD469;Myelodysplastisk syndrom UNS			2
DD47;Andre neoplasier fra lymfoidt og hæmatopoietisk væv af usikker og ukendt karakter			2
DD470;Histiocytær eller mastcelle neoplasi af usikker eller ukendt karakter			2
DD470A;Mastcelle tumor UNS			2
DD470B;Indolent mastocytosis			2
DD470C;Systemisk mastocytose, associeret med klonal hæmatopoietisk non-mastcelle sygdom (SM-AHNMD)			2

DD471;Kronisk myeloproliferativt syndrom			2
DD471B;Myeloproliferativ sygdom UNS			2
DD472;Monoklonal gammopati med ubestemt signifikans (MGUS)			2
DD472A;Monoklonal gammopati med ubestemt signifikans (MGUS) IgM type			2
DD472B;Monoklonal gammopati med ubestemt signifikans (MGUS) non-IGM type			2
DD473;Essentiel trombocytæmi			2
DD474;Primær og sekundær myelofibrose			2
DD474A;Primær myelofibrose			2
DD474B;Sekundær myelofibrose efter polycytæmia vera			2
DD474C;Sekundær myelofibrose efter essentiel trombocytose			2
DD475;Hypereosinofilt syndrom			2
DD477;Anden neoplasi fra lymfoidt eller hæmatopoietisk væv af usikker eller ukendt karakter			2
DD477A;Lymfomatoid granulomatose			2
DD477C;Tidlig post-transplantations lymfoproliferativ sygdom (tidlig PTLD)			2
DD477D;Lymfomatoid papulose			2
DD479;Neoplasi fra lymfoidt eller hæmatopoietisk væv af usikker eller ukendt karakter			2
DD479A;Lymfoproliferativ sygdom UNS			2
DD479B;Monoklonal B-celle lymfocytose (MBL)			2
DD48;Tumorer af usikker og ukendt karakter med andre og ikke nærmere specificerede lokalisationer			2
DD480;Ikke specificeret tumor i knogle eller ledbrusk			2
DD481;Ikke specificeret tumor i bindevæv eller andre bløddele			2
DD482;Ikke specificeret tumor i perifer nerve eller autonome nervesystem			2
DD482A;Ikke specificeret tumor i parasympatisk ganglion			2
DD482B;Ikke specificeret tumor i perifert ganglion			2
DD482C;Ikke specificeret tumor i sympatisk ganglion			2
DD482D;Ikke specificeret tumor i autonome nervesystem			2
DD482E;Ikke specificeret tumor i perifer nerve			2
DD483;Ikke specificeret tumor i retroperitoneum			2
DD484;Ikke specificeret tumor i peritoneum			2
DD485;Ikke specificeret tumor i huden			2
DD485A;Ikke specificeret tumor i hud i anus			2
DD485B;Ikke specificeret tumor i hud på mamma			2

Supplement 2 Nielsen et al. Mortality after paediatric emergency calls for patients with and without pre-existing comorbidity (2023)

DD485C;Ikke specificeret tumor i hud i perineum			2
DD485D;Ikke specificeret tumor i hud på øjenlåg			2
DD486;Ikke specificeret tumor i mamma			2
DD486A;Phylloides tumor i bryst af usikker eller ukendt karakter			2
DD487;Ikke specificeret tumor med anden lokalisation			2
DD487A;Ikke specificeret tumor i hjertet			2
DD487B;Ikke specificeret tumor i perifer nerve i øjenhule			2
DD487C;Ikke specificeret tumor i øje UNS			2
DD487D;Ikke specificeret tumor i mediastinum UNS			2
DD489;Tumor af usikker eller ukendt karakter uden nærmere specificeret lokalisation			2
DD50;Jernmangelanæmi		1	
DD500;Kronisk blødningsanæmi		1	
DD501;Jernmangelanæmi forårsaget af malabsorption af jern		1	
DD501A;Plummer-Vinson syndrom		1	
DD508;Jernmangelanæmi af anden årsag		1	
DD508A;Jernmangelanæmi forårsaget af insufficient indtag af jern		1	
DD509;Jernmangelanæmi UNS		1	
DD509A;Hypokrom anæmi UNS		1	
DD51;Anæmi forårsaget af vitamin B12-mangel		1	
DD510;Anæmi forårsaget af vitamin B12-mangel ved mangel på intrinsic factor		1	
DD511;Anæmi forårsaget af malabsorption af vitamin B12 med proteinuri		1	
DD511A;Arvelig megaloblastær anæmi		1	
DD512;Anæmi forårsaget af mangel på transkobalamin II		1	
DD513;Anden anæmi forårsaget af ernæringsbetinget mangel på vitamin B12		1	
DD513A;Veganæmi		1	
DD518;Anden anæmi forårsaget af vitamin B12-mangel		1	
DD519;Anæmi forårsaget af vitamin B12-mangel UNS		1	
DD52;Anæmi forårsaget af folinsyremangel		1	
DD520;Anæmi forårsaget af ernæringsbetinget folinsyremangel		1	
DD521;Anæmi forårsaget af medikamentelt betinget folinsyremangel		1	
DD528;Anden anæmi forårsaget af folinsyremangel		1	
DD529;Anæmi forårsaget af folinsyremangel UNS		1	

DD53;Andre ernæringsbetingede anæmier		1	
DD530;Proteinmangelanæmi		1	
DD531;Anden megaloblastær anæmi IKA		1	
DD531A;Megaloblastær anæmi UNS		1	
DD532;Anæmi forårsaget af vitamin C-mangel		1	
DD538;Anden ernæringsbetinget anæmi		1	
DD538A;Anæmi forårsaget af kobbermangel		1	
DD538B;Anæmi forårsaget af molybdænmangel		1	
DD538C;Anæmi forårsaget af zinkmangel		1	
DD538M;Anæmi ved malabsorption efter tidligere kirurgisk indgreb UNS		1	
DD539;Ernæringsbetinget anæmi UNS		1	
DD55;Anæmi forårsaget af enzymatiske forstyrrelser		1	
DD550;Anæmi forårsaget af glukose-6-fosfat-dehydrogenasemangel		1	
DD551;Anæmi forårsaget af anden forstyrrelse i glutationomsætningen		1	
DD552;Anæmi forårsaget af forstyrrelser i de glykolytiske enzymer		1	
DD553;Anæmi forårsaget af forstyrrelser i nukleotidomsætningen		1	
DD558;Anden anæmi forårsaget af enzymatisk forstyrrelse		1	
DD559;Anæmi forårsaget af enzymatisk forstyrrelse UNS		1	
DD56;Talassæmier		1	
DD560;Alfa-talassæmi		1	
DD561;Beta-talassæmi		1	
DD561A;Thalassaemia major		1	
DD561C;Thalassaemia intermedia		1	
DD562;Delta-beta talassæmi		1	
DD563;Thalassaemia minor		1	
DD564;Arveligt persisterende føtalt hæmoglobin (HPFH)		1	
DD568;Anden form for talassæmi		1	
DD569;Talassæmi UNS		1	
DD57;Seglcellesygdomme		1	
DD570;Seglcelleanæmi med krise		1	
DD571;Seglcelleanæmi uden krise		1	
DD572;Dobbelt heterozygot seglcelleanæmi		1	

DD572A;Hb-SC sygdom		1	
DD572B;Hb-SD sygdom		1	
DD572C;Hb-SE sygdom		1	
DD572D;Seglcelle talassæmi		1	
DD573;Heterozygot hæmoglobin S		1	
DD578;Anden form for seglcellesygdom		1	
DD58;Andre arvelige hæmolytiske anæmier		1	
DD580;Arvelig hæmolytisk anæmi forårsaget af sfærocytose		1	
DD581;Arvelig hæmolytisk anæmi forårsaget af elliptocytose		1	
DD581A;Medfødt elliptocytose		1	
DD582;Anden hæmoglobinopati		1	
DD582A;Abnormt hæmoglobin UNS		1	
DD582B;Medfødt Heinz Body anæmi		1	
DD582C;Hb-C sygdom		1	
DD582D;Hb-D sygdom		1	
DD582E;Hb-E sygdom		1	
DD588;Anden arvelig hæmolytisk anæmi		1	
DD588A;Stomatocytose		1	
DD589;Arvelig hæmolytisk anæmi UNS		1	
DD59;Erhvervede hæmolytiske anæmier		1	
DD590;Autoimmun hæmolytisk anæmi forårsaget af lægemiddel		1	
DD591;Anden autoimmun hæmolytisk anæmi		1	
DD591A;Kuldeagglutininsyndrom		1	
DD592;Hæmolytisk ikke-autoimmun anæmi forårsaget af lægemiddel		1	
DD593;Hæmolytisk-uræmisk syndrom		1	
DD594;Anden hæmolytisk ikke-autoimmun anæmi		1	
DD594A;Mekanisk hæmolytisk anæmi		1	
DD594B;Mikroangiopatisk hæmolytisk anæmi		1	
DD594C;Toksisk hæmolytisk anæmi		1	
DD595;Paroxysmal nokturn hæmoglobinuri		1	
DD596;Hæmoglobinuri ved hæmolyse forårsaget af anden ydre årsag		1	
DD596A;Kuldehæmoglobinuri		1	

Supplement 2 Nielsen et al. Mortality after paediatric emergency calls for patients with and without pre-existing comorbidity (2023)

DD596B;Marchhæmoglobinuri		1	
DD598;Anden erhvervet hæmolytisk anæmi		1	
DD599;Erhvervet hæmolytisk anæmi UNS		1	
DD599A;Idiopatisk (kronisk) hæmolytisk anæmi		1	
DD60;Erhvervet pure red cell aplasi			2
DD600;Kronisk erhvervet pure red cell aplasi			2
DD601;Forbigående erhvervet pure red cell aplasi			2
DD608;Anden erhvervet pure red cell aplasi			2
DD609;Erhvervet pure red cell aplasi UNS			2
DD61;Andre aplastiske anæmier			2
DD610;Konstitutionel aplastisk anæmi			2
DD610A;Blackfan-Diamond syndrom			2
DD610B;Fanconis anæmi			2
DD610C;Pancytopeni med misdannelser			2
DD610D;Familiær hypoplastisk anæmi			2
DD610E;Aplasi af røde blodlegemer hos barn (medfødt)(primær)			2
DD611;Aplastisk anæmi forårsaget af lægemiddel			2
DD612;Aplastisk anæmi forårsaget af anden ydre påvirkning			2
DD613;Idiopatisk aplastisk anæmi			2
DD618;Anden aplastisk anæmi			2
DD619;Aplastisk anæmi UNS			2
DD619A;Pancytopeni UNS			2
DD619B;Hypoplastisk anæmi UNS			2
DD619C;Medullær hypoplasi			2
DD619D;Panmyelophthisis			2
DD62;Akut anæmi efter blødning	0		
DD629;Akut blødningsanæmi UNS	0		
DD63;Anæmi ved kroniske sygdomme klassificeret andetsteds		1	
DD630;Anæmi ved neoplastisk sygdom			2
DD638;Anæmi ved anden kronisk sygdom klassificeret andetsteds		1	
DD64;Andre anæmier		1	
DD640;Arvelig sideroblastær anæmi		1	

DD640A;Kønsbunden hypokrom sideroblastær anæmi		1		
DD641;Sekundær sideroblastær anæmi forårsaget af anden sygdom		1		
DD642;Sekundær sideroblastær anæmi forårsaget af lægemiddel eller toksin		1		
DD643;Anden sideroblastær anæmi		1		
DD643A;Sideroblastær anæmi UNS		1		
DD644;Medfødt dyserytropoietisk anæmi		1		
DD648;Anden anæmi		1		
DD648A;Leukoerytroblastær anæmi		1		
DD648B;Infantil pseudoleukæmi		1		
DD649;Anæmi UNS		1		
DD65;Dissemineret intravaskulær koagulation		1		
DD659;Dissemineret intravaskulær koagulation		1		
DD659A;Erhvervet afibrinogenæmi		1		
DD659B;Erhvervet fibrinolytisk blødning		1		
DD659C;Fibrinolytisk purpura		1		
DD659D;Purpura fulminans		1		
DD66;Arvelig faktor VIII-mangel		1		
DD669;Hæmofili A		1		
DD669A;Hæmofili UNS (findes ikke i SKS)		1		
DD669B;Hæmofili A, symptomatisk hos kvindelig bærer		1		
DD669C;Hæmofili A mild		1		
DD669D;Hæmofili A moderat		1		
DD669E;Hæmofili A svær		1		
DD67;Arvelig faktor IX-mangel		1		
DD679;Hæmofili B		1		
DD679A;Hæmofili B, symptomatisk hos kvindelig bærer		1		
DD679B;Hæmofili B mild		1		
DD679C;Hæmofili B moderat		1		
DD679D;Hæmofili B svær		1		
DD68;Andre koagulationsdefekter		1		
DD680;von Willebrands sygdom		1		
DD680A;von Willebrands sygdom type 1		1		

Supplement 2 Nielsen et al. Mortality after paediatric emergency calls for patients with and without pre-existing comorbidity (2023)

DD680B;von Willebrands sygdom type 2		1	
DD680B1;von Willebrands sygdom type 2A		1	
DD680B2;von Willebrands sygdom type 2B		1	
DD680B3;von Willebrands sygdom type 2M		1	
DD680B4;von Willebrands sygdom type 2N		1	
DD680C;von Willebrands sygdom type 3		1	
DD681;Arvelig faktor XI-mangel		1	
DD682;Arvelig mangel på andre koagulationsfaktorer		1	
DD682A;Medfødt afibrinogenæmi		1	
DD683;Blødningsforstyrrelse forårsaget af cirkulerende antikoagulantia		1	
DD683A;Hyperheparinæmi		1	
DD684;Erhvervet koagulationsfaktormangel		1	
DD684A;Erhvervet koagulationsfaktormangel ved hypoprothrombinæmi		1	
DD684B;Erhvervet koagulationsfaktormangel forårsaget af K-vitaminmangel		1	
DD684C;Erhvervet koagulationsfaktormangel forårsaget af leversygdom		1	
DD685;Primær trombofili		1	
DD685A;Koagulationsfaktor V Leiden mutation bp 1691 (heterozygot)		1	
DD685B;Koagulationsfaktor V Leiden mutation bp 1691 (homozygot)		1	
DD685E;Prothrombin, mutation bp 20210 (heterozygot)		1	
DD685F;Prothrombin, mutation bp 20210 (homozygot)		1	
DD685G;Methylenetetrahydrofolat reduktase, mutat.bp 677 (homozygot) (findes ikke i SKS)		1	
DD685K;Protein C mangel		1	
DD685L;Protein S mangel		1	
DD685M;Antithrombin III mangel		1	
DD686;Anden trombofili		1	
DD686E;Kardiolipinantistofsyndrom		1	
DD686F;Antifosfolipidsyndrom		1	
DD686G;Vaccineinduceret immun trombotisk trombocytopeni (VITT)		1	
DD688;Anden koagulationsdefekt		1	
DD689;Koagulationsdefekt UNS		1	
DD69;Purpura og andre tilstande med blødningstendens		1	
DD690;Allergisk purpura		1	

Supplement 2 Nielsen et al. Mortality after paediatric emergency calls for patients with and without pre-existing comorbidity (2023)

DD690A;Purpura anaphylactica		1	
DD690B;Schönlein-Henochs purpura		1	
DD690C;Purpura non thrombocytopenica haemorrhagica		1	
DD690D;Purpura non thrombocytopenica idiopathica		1	
DD690E;Purpura vascularis		1	
DD690F;Vasculitis allergica		1	
DD691;Blodpladedefekter		1	
DD691A;Bernard-Soulier syndrom		1	
DD691B;Glanzmanns trombasteni		1	
DD691C;Trombasteni (hæmoragisk) (hereditær)		1	
DD692;Anden purpura uden trombocytmangel eller defekt		1	
DD692B;Purpura senilis		1	
DD692C;Purpura simplex		1	
DD692D;Purpura UNS		1	
DD692E;Purpura annularis telangiectodes		1	
DD692F;Gardner-Diamonds syndrom		1	
DD692H;Purpura hyperglobulinaemica		1	
DD693;Idiopatisk trombocytopenisk purpura		1	
DD694;Anden primær trombocytopeni		1	
DD694A;Føtal og neonatal alloimmun trombocytopeni (FNAIT)		1	
DD695;Sekundær trombocytopeni		1	
DD696;Trombocytopeni UNS		1	
DD698;Anden tilstand med blødningstendens		1	
DD698A;Fragilitas capillaris (hereditaria)		1	
DD698B;Vaskulær pseudohæmofili		1	
DD699;Blødningstendens UNS		1	
DD70;Neutropeni			2
DD709;Neutropeni UNS			2
DD709A;Neutropeni og agranulocytose forårsaget af lægemiddel			2
DD709B;Toksisk neutropeni			2
DD709C;Medfødt neutropeni			2
DD709D;Cyklisk neutropeni			2

DD709E;Neutropenisk splenomegali			2
DD71;Funktionelle forstyrrelser i polymorfkærnede neutrofile celler		1	
DD719;Funktionelle forstyrrelser i polymorfkærnede neutrofile celler UNS		1	
DD719A;Medfødt dysfagocytose		1	
DD719B;Progressiv septisk granulomatose		1	
DD719C;Kronisk granulomatøs sygdom hos børn		1	
DD719D;Defekt i cellemembranreceptorkomplex (CR3)		1	
DD72;Andre forstyrrelser i hvide blodlegemer		1	
DD720;Genetisk betinget leukocytanomali		1	
DD720A;Alder's anomali		1	
DD720B;Hereditær leukocytær hypersegmentation		1	
DD720C;Hereditær leukocytær hyposegmentation		1	
DD720D;Hereditær leukomelanopati		1	
DD720E;May-Hegglins anomali		1	
DD720F;Pelger-Huëts anomali		1	
DD721;Eosinofili		1	
DD721A;Allergisk eosinofili		1	
DD721B;Hereditær eosinofili		1	
DD728;Anden forstyrrelse i hvide blodlegemer		1	
DD728A;Leukocytose		1	
DD728B;Leukæmoid reaktion		1	
DD728C;Lymfocytose (symptomatisk)		1	
DD728D;Lymfopeni		1	
DD728E;Monocytose (symptomatisk)		1	
DD728F;Plasmacytose		1	
DD728G;Pseudolymfom		1	
DD728H;Leukopeni		1	
DD729;Sygdom i hvide blodlegemer UNS		1	
DD73;Sygdomme i milten		1	
DD730;Nedsat miltfunktion		1	
DD730A;Atrofi af milten		1	
DD730B;Erhvervet mangel af milten		1	

DD731;Øget miltfunktion		1		
DD732;Kronisk stasemilt		1		
DD733;Miltabsces		1		
DD734;Miltcyste		1		
DD735;Miltinfarkt		1		
DD735A;Spontan miltruptur		1		
DD735B;Milttorsion		1		
DD738;Anden sygdom i milten		1		
DD738A;Fibrosis lienis		1		
DD738C;Perisplenitis		1		
DD738D;Splenitis UNS		1		
DD739;Sygdom i milten UNS		1		
DD74;Methæmoglobinæmi		1		
DD740;Medfødt methæmoglobinæmi		1		
DD740A;Medfødt methæmoglobinreduktasemangel		1		
DD740B;Hæmoglobin-M (Hb-M) sygdom		1		
DD748;Anden methæmoglobinæmi		1		
DD748A;Erhvervet methæmoglobinæmi		1		
DD748B;Toksisk methæmoglobinæmi		1		
DD748C;Sulfhæmoglobinæmi		1		
DD749;Methæmoglobinæmi UNS		1		
DD75;Andre sygdomme i blod og bloddannende organer		1		
DD750;Familiær polycytæmi		1		
DD751;Sekundær polycytæmi		1		
DD751C;Højdebetinget polycytæmi		1		
DD751D;Erythropoietin-induceret polycytæmi		1		
DD751E;Hypoxisk polycytæmi		1		
DD751F;Sekundær erytrocytose		1		
DD751G;Sekundær trombocytose		1		
DD751H;Sekundær leukocytose		1		
DD758;Anden sygdom i blod eller bloddannende væv		1		
DD758C;Idiopatisk cytopeni af ukendt signifikans (ICUS)		1		

DD758D;Klonal cytopeni af ukendt signifikans (CCUS)		1	
DD759;Sygdom i blod eller bloddannende væv UNS		1	
DD76;Andre sygdomme, der involverer lymforetikulære og retikulohistiocytære væv			2
DD761;Hæmofagocytær lymfhistiocytose			2
DD761A;Familiær hæmofagocytær retikulose			2
DD762;Hæmofagocytært syndrom, infektionsassocieret			2
DD763;Andet histiocytært syndrom			2
DD763A;Retikulohistiocytom (gigantocellulært)			2
DD763B;Xanthogranulom		1	
DD763D;Sinus histiocytose med massiv lymfadenopati		1	
DD77;Andre sygdomme i blod og bloddannende organer ved sygdomme klassificeret andetsteds		1	
DD779;Sygdom i blod og bloddannende organer ved sygdom klassificeret andetsteds		1	
DD779A;Fibrose i milt ved sygdom klassificeret andetsteds		1	
DD80;Immundefekt med overvejende antistofmangel		1	
DD800;Arvelig hypogammaglobulinæmi		1	
DD800A;Autosomal recessiv agammaglobulinæmi (Swiss type)		1	
DD800B;X-bundet agammaglobulinæmi med væksthormonmangel		1	
DD801;Ikke-familiær hypogammaglobulinæmi		1	
DD801A;Hypogammaglobulinæmi UNS		1	
DD801B;Agammaglobulinæmi med immunglobulinbærende B-lymfocyter		1	
DD801C;Common variable agammaglobulinemia (CVA-gamma)		1	
DD802;Selektiv IgA-mangel		1	
DD803;Selektiv IgG-mangel		1	
DD804;Selektiv IgM-mangel		1	
DD805;Immundefekt med øget IgM		1	
DD806;Antistofmangel med hyperimmunglobulinæmi		1	
DD807;Hypogammaglobulinaemia neonatorum transitoria		1	
DD808;Anden immundefekt med overvejende antistofmangel		1	
DD808A;Kappa light chain-mangel		1	
DD809;Immundefekt med overvejende antistofmangel UNS		1	
DD81;Kombinerede immundefekter			2
DD810;Svær kombineret immundefekt (SCID) med retikulær dysgenesi			2

DD811;Svær kombineret immundefekt (SCID) med lavt antal T- og B-celler			2
DD812;Svær kombineret immundefekt (SCID) med lavt eller normalt antal B-celler			2
DD813;Adenosindeaminase (ADA) mangel			2
DD814;Nezelofs syndrom			2
DD815;Purinnukleosidfosforylase (PNP) mangel			2
DD816;Histokompatibilitetskompleksmangel, klasse I			2
DD817;Histokompatibilitetskompleksmangel, klasse II			2
DD818;Anden kombineret immundefekt			2
DD819;Kombineret immundefekt UNS			2
DD819A;Svær kombineret immundefekt (SCID) UNS			2
DD82;Immundefekt i forbindelse med andre alvorlige defekter			2
DD820;Wiskott-Aldrichs syndrom		1	
DD821;DiGeorges syndrom		1	
DD822;Immundefekt med korte ekstremiteter			2
DD823;Immundefekt ved abnorm reaktion på Epstein-Barr virus			2
DD823A;Kønsbunden lymfoproliferativ sygdom			2
DD824;Hyperimmunoglobin E (IgE) syndrom			2
DD828;Immundefekt ved andre større specificerede defekter			2
DD829;Immundefekt associeret med større defekt UNS			2
DD83;Almindelige variable immundefekter		1	
DD830;Immundefekt med overvejende abnormt B-celletal og -funktion		1	
DD831;Immundefekt med overvejende forstyrrelse i T-celler		1	
DD832;Immundefekt med autoantistoffer mod B- eller T-celler		1	
DD838;Anden almindelig variabel immundefekt		1	
DD839;Almindelig variabel immundefekt UNS		1	
DD84;Andre immundefekter		1	
DD840;Defekt i lymfocytfunktionsantigen-1		1	
DD841;Defekt i komplementsystemet		1	
DD841A;Hereditært angioødem		1	
DD841B;C1-esteraseinhibitor-mangel		1	
DD848;Anden immundefekt		1	
DD849;Immundefekt UNS		1	

DD86;Sarkoidose		1	
DD860;Sarkoidose i lunger		1	
DD861;Sarkoidose i lymfeknuder		1	
DD862;Sarkoidose i både lunger og lymfeknuder		1	
DD863;Sarkoidose i hud		1	
DD863A;Lupus pernio		1	
DD868;Sarkoidose med anden lokalisation eller flere lokalisationer		1	
DD868A;Febris uveoparotidea (Heerfordt)		1	
DD868G;Akut sarkoidose UNS		1	
DD869;Sarkoidose UNS		1	
DD89;Andre forstyrrelser i immunsystemet IKA		1	
DD890;Polyklonal hypergammaglobulinæmi		1	
DD890A;Polyklonal gammopati UNS		1	
DD890B;Benign hypergammaglobulinæmisk purpura		1	
DD891;Kryoglobulinæmi		1	
DD892;Hypergammaglobulinæmi UNS		1	
DD898;Anden forstyrrelse i immunsystemet IKA		1	
DD899;Sygdom i immunsystemet UNS		1	
DE00;Medfødt jodmangelsyndrom		1	
DE000;Medfødt jodmangelsyndrom med neurologiske symptomer		1	
DE001;Medfødt jodmangelsyndrom med myksødem		1	
DE002;Medfødt jodmangelsyndrom af blandet form		1	
DE009;Medfødt jodmangelsyndrom UNS		1	
DE01;Jodmangelrelaterede lidelser i skjoldbruskkirtlen og beslægtede tilstande		1	
DE010;Endemisk diffus struma forårsaget af jodmangel		1	
DE011;Endemisk knudestruma forårsaget af jodmangel		1	
DE012;Endemisk jodmangel struma UNS		1	
DE018;Anden struma eller beslægtet tilstand forårsaget af jodmangel		1	
DE02;Kompenseret jodmangelbetinget myksødem		1	
DE029;Kompenseret jodmangelbetinget myksødem UNS		1	
DE03;Anden hypothyroidisme		1	
DE030;Medfødt myksødem med diffus struma		1	

DE030A;Medfødt myksødem UNS		1	
DE031;Medfødt myksødem uden struma		1	
DE031A;Aplasi af skjoldbruskkirtlen med myksødem		1	
DE031B;Medfødt hypoplasi af skjoldbruskkirtlen		1	
DE031C;Medfødt hypothyroidisme UNS		1	
DE032;Hypothyroidisme forårsaget af lægemiddel eller andet fremmed agens		1	
DE033;Myksødem efter infektion		1	
DE034;Erhvervet atrofi af skjoldbruskkirtlen		1	
DE035;Myksødematøs koma		1	
DE038;Anden form for hypothyroidisme		1	
DE039;Hypothyroidisme UNS		1	
DE04;Anden atoksisk struma		1	
DE040;Atoksisk diffus struma		1	
DE041;Atoksisk struma med solitært adenom		1	
DE042;Atoksisk multinodøs struma		1	
DE042A;Cystisk struma UNS		1	
DE048;Anden form for atoksisk struma		1	
DE048A;Atoksisk recidivstruma		1	
DE049;Atoksisk struma UNS		1	
DE05;Thyrotoksikose		1	
DE050;Thyrotoksikose med diffus struma		1	
DE051;Thyrotoksikose med toksisk solitært adenom		1	
DE052;Thyrotoksikose med toksisk multinodøs struma		1	
DE052A;Toksisk nodøs struma UNS		1	
DE053;Thyrotoksikose forårsaget af ektopisk thyroideavæv		1	
DE054;Thyrotoksikose ved overdosering af thyroideahormon		1	
DE055;Thyrotoksisk krise		1	
DE058;Anden form for thyrotoksikose		1	
DE058A;Hyperthyreose ved overproduktion af thyroideastimulerende hormon (TSH)		1	
DE058B;Toksisk redicivstruma		1	
DE058C;Thyrotoksikose uden struma		1	
DE059;Thyrotoksikose UNS		1	

Supplement 2 Nielsen et al. Mortality after paediatric emergency calls for patients with and without pre-existing comorbidity (2023)

DE059A;Thyrotoksisk hjertesygdom		1	
DE06;Betændelse i skjoldbruskkirtlen		1	
DE060;Akut thyroiditis		1	
DE060A;Absces i skjoldbruskkirtlen		1	
DE060B;Thyroiditis suppurativa		1	
DE061;Subakut thyroiditis		1	
DE061C;Thyroiditis non suppurativa		1	
DE062;Kronisk thyroiditis med forbigående thyrotoksikose		1	
DE063;Autoimmun thyroiditis		1	
DE063A;Hashimotos thyroiditis		1	
DE063B;Struma lymphomatosa		1	
DE064;Thyroiditis forårsaget af lægemiddel		1	
DE065;Anden kronisk betændelse i skjoldbruskkirtlen		1	
DE065A;Thyroiditis fibrosa		1	
DE065B;Thyroiditis lignosa		1	
DE065C;Riedels struma		1	
DE065D;Kronisk thyroiditis UNS		1	
DE069;Betændelse i skjoldbruskkirtlen UNS		1	
DE07;Andre sygdomme i skjoldbruskkirtlen		1	
DE070;Forøget sekretion af kalcitonin		1	
DE070A;C-celle hyperplasi af skjoldbruskkirtlen		1	
DE071;Dyshormogenetisk struma		1	
DE071A;Pendreds syndrom		1	
DE071B;Familiær dyshormogen struma		1	
DE078;Anden forstyrrelse i skjoldbruskkirtlen		1	
DE078B;Blødning i skjoldbruskkirtlen		1	
DE078C;Infarkt i skjoldbruskkirtlen		1	
DE078D;Sick-euthyroid syndrom		1	
DE078E;Abnormitet i thyroidea-bindende globulin		1	
DE079;Forstyrrelse i skjoldbruskkirtlen UNS		1	
DE10;Type 1-diabetes		1	
DE100;Type 1-diabetes med koma		1	

Supplement 2 Nielsen et al. Mortality after paediatric emergency calls for patients with and without pre-existing comorbidity (2023)

DE101;Type 1-diabetes med ketoacidose		1	
DE102;Type 1-diabetes med nyrekomplikation		1	
DE103;Type 1-diabetes med øjenkomplikation		1	
DE104;Type 1-diabetes med neurologisk komplikation		1	
DE105;Type 1-diabetes med komplikationer i perifere karsystem		1	
DE105A;Type 1-diabetes med perifer angiopati		1	
DE105B;Type 1-diabetes med fodsår		1	
DE105C;Type 1-diabetes med gangræn		1	
DE105D;Type 1-diabetes med mikroangiopati		1	
DE106;Type 1-diabetes med anden komplikation		1	
DE107;Type 1-diabetes med multiple komplikationer		1	
DE108;Type 1-diabetes med komplikation UNS		1	
DE109;Type 1-diabetes uden komplikationer		1	
DE109A;Type 1-diabetes UNS		1	
DE11;Type 2-diabetes		1	
DE110;Type 2-diabetes med koma		1	
DE111;Type 2-diabetes med ketoacidose		1	
DE112;Type 2-diabetes med nyrekomplikation		1	
DE113;Type 2-diabetes med øjenkomplikation		1	
DE114;Type 2-diabetes med neurologisk komplikation		1	
DE115;Type 2-diabetes med komplikationer i perifere karsystem		1	
DE115A;Type 2-diabetes med perifer angiopati		1	
DE115B;Type 2-diabetes med fodsår		1	
DE115C;Type 2-diabetes med gangræn		1	
DE115D;Type 2-diabetes med mikroangiopati		1	
DE116;Type 2-diabetes med anden komplikation		1	
DE117;Type 2-diabetes med multiple komplikationer		1	
DE118;Type 2-diabetes med komplikation UNS		1	
DE119;Type 2-diabetes uden komplikationer		1	
DE119A;Type 2-diabetes UNS		1	
DE12;Diabetes forårsaget af underernæring		1	
DE120;Diabetes forårsaget af underernæring med koma		1	

Supplement 2 Nielsen et al. Mortality after paediatric emergency calls for patients with and without pre-existing comorbidity (2023)

DE121;Diabetes forårsaget af underernæring med ketoacidose		1	
DE122;Diabetes forårsaget af underernæring med nyrekomplikation		1	
DE123;Diabetes forårsaget af underernæring med øjenkomplikation		1	
DE124;Diabetes forårsaget af underernæring med neurologisk komplikation		1	
DE125;Diabetes forårsaget af underernæring med komplikationer i perifere karsystem		1	
DE125A;Diabetes forårsaget af underernæring med perifer angiopati		1	
DE125B;Diabetes forårsaget af underernæring med fodsår		1	
DE125C;Diabetes forårsaget af underernæring med gangræn		1	
DE125D;Diabetes forårsaget af underernæring med mikroangiopati		1	
DE126;Diabetes forårsaget af underernæring med anden komplikation		1	
DE127;Diabetes forårsaget af underernæring med multiple komplikationer		1	
DE128;Diabetes forårsaget af underernæring med komplikation UNS		1	
DE129;Diabetes forårsaget af underernæring uden komplikationer		1	
DE13;Andre former for diabetes		1	
DE130;Anden diabetes med koma		1	
DE131;Anden diabetes med ketoacidose		1	
DE132;Anden diabetes med nyrekomplikation		1	
DE133;Anden diabetes med øjenkomplikationer		1	
DE134;Anden diabetes med neurologisk komplikation		1	
DE135;Anden diabetes med komplikationer i perifere karsystem		1	
DE135A;Anden diabetes med perifer angiopati		1	
DE135B;Anden diabetes med fodsår		1	
DE135C;Anden diabetes med gangræn		1	
DE135D;Anden diabetes med mikroangiopati		1	
DE136;Anden diabetes med anden komplikation		1	
DE137;Anden diabetes med multiple komplikationer		1	
DE138;Anden diabetes med komplikation UNS		1	
DE139;Anden diabetes uden komplikationer		1	
DE14;Ikke specificeret diabetes		1	
DE140;Diabetes UNS med koma		1	
DE141;Diabetes UNS med ketoacidose		1	
DE142;Diabetes UNS med nyrekomplikation		1	

Supplement 2 Nielsen et al. Mortality after paediatric emergency calls for patients with and without pre-existing comorbidity (2023)

DE143;Diabetes UNS med øjenkomplikation		1	
DE144;Diabetes UNS med neurologisk komplikation		1	
DE145;Diabetes UNS med komplikationer i perifere karsystem		1	
DE145A;Diabetes UNS med perifer angiopati		1	
DE145B;Diabetes UNS med fodsår		1	
DE145C;Diabetes UNS med gangræn		1	
DE145D;Diabetes UNS med mikroangiopati		1	
DE146;Diabetes UNS med anden komplikation		1	
DE147;Diabetes UNS med multiple komplikationer		1	
DE148;Diabetes UNS med komplikation UNS		1	
DE149;Diabetes UNS uden komplikationer		1	
DE15;Ikke-diabetisk hypoglykæmisk koma		1	
DE159;Hypoglykæmisk koma UNS		1	
DE159B;Hypoglykæmisk koma ved hyperinsulisme		1	
DE16;Andre forstyrrelser i bugspytkirtlens interne sekretion		1	
DE160;Hypoglykæmi uden koma forårsaget af lægemiddel		1	
DE161;Anden form for hypoglykæmi		1	
DE161A;Betacellehyperplasi i pancreas med hyperinsulinisme		1	
DE161B;Encefalopati efter hypoglykæmisk koma		1	
DE161C;Funktionel hyperinsulinisme		1	
DE161D;Hyperinsulinisme UNS		1	
DE162;Hypoglykæmi UNS	0		
DE163;Øget glukagonsekretion		1	
DE163A;Betacellehyperplasi i pancreas med glukagonoverproduktion		1	
DE164;Abnorm gastrinsekretion		1	
DE164A;Hypergastrinæmi		1	
DE164B;Zollinger-Ellisons syndrom		1	
DE168;Andre forstyrrelser i pancreas interne sekretion		1	
DE168B;Øget sekretion af pankreatisk polypeptid (PPP)		1	
DE168C;Øget sekretion af pankreatisk somatostatin (SS-14)		1	
DE168D;Øget sekretion af pankreatisk vasoaktivt intestinalt polypeptid (VIP)		1	
DE168F;Øget sekretion af pankreatisk væksthormon releasing hormon (GHRH)		1	

DE169;Forstyrrelse i pancreas interne sekretion UNS			1	
DE169A;Hyperplasi af pancreas endokrine celler UNS			1	
DE20;Hypoparathyroidisme			1	
DE200;Idiopatisk hypoparathyroidisme			1	
DE201;Pseudohypoparathyroidisme			1	
DE208;Anden hypoparathyroidisme			1	
DE209;Hypoparathyroidisme UNS			1	
DE21;Hyperparathyroidisme og andre sygdomme i biskjoldbruskkirtel			1	
DE210;Primær hyperparathyroidisme			1	
DE210A;Hyperplasi af biskjoldbruskkirtel			1	
DE210B;Osteitis fibrosa cystica generalisata			1	
DE211;Sekundær hyperparathyroidisme			1	
DE212;Anden form for hyperparathyroidisme			1	
DE212A;Tertiær hyperparathyroidisme			1	
DE213;Hyperparathyroidisme UNS			1	
DE213A;Hyperparathyroidismekrise			1	
DE214;Anden sygdom i biskjoldbruskkirtel			1	
DE215;Sygdom i biskjoldbruskkirtel UNS			1	
DE22;Øget aktivitet i hypofysen			1	
DE220;Hypofysær kæmpevækst eller akromegali			1	
DE220A;Akromegali			1	
DE220C;Hypofysær kæmpevækst			1	
DE221;Hyperprolaktinæmi			1	
DE222;Øget sekretion af antidiuretisk hormon (ADH)			1	
DE222A;Syndrome of inappropriate secretion of antidiuretic hormone (SIADH)			1	
DE228;Anden øget hypofyseaktivitet			1	
DE228A;Pubertas praecox centralis			1	
DE229;Øget hypofyseaktivitet UNS			1	
DE23;Nedsat aktivitet og andre sygdomme i hypofysen			1	
DE230;Nedsat hormonsekretion fra hypofysen			1	
DE230A;Isoleret nedsat sekretion af gonadotropin			1	
DE230B;Isoleret nedsat sekretion af hypofysehormon UNS			1	

Supplement 2 Nielsen et al. Mortality after paediatric emergency calls for patients with and without pre-existing comorbidity (2023)

DE230C;Isoleret nedsat sekretion af væksthormon		1	
DE230D;Kallmanns syndrom		1	
DE230E;Lorain-Levis dværgvækst		1	
DE230F;Nekrose af hypofysen efter fødsel		1	
DE230G;Sheehans syndrom		1	
DE230H;Simmonds syndrom		1	
DE230I;Hypofysær kakeksi		1	
DE231;Hypopituitarisme forårsaget af lægemiddel		1	
DE232;Diabetes insipidus		1	
DE233;Hypotalamisk dysfunktion IKA		1	
DE236;Anden sygdom i hypofysen		1	
DE236A;Absces i hypofysen		1	
DE236B;Dystrophia adiposogenitalis efter intrakraniel læsion		1	
DE236C;Dystrophia adiposogenitalis		1	
DE236E;Apoplexia pituitaria		1	
DE237;Sygdom i hypofysen UNS		1	
DE24;Cushings syndrom		1	
DE240;Hypofysært betinget Cushings sygdom		1	
DE241;Nelsons syndrom		1	
DE242;Cushings syndrom forårsaget af lægemiddel		1	
DE243;Ektopisk ACTH syndrom		1	
DE244;Alkoholinduceret pseudo-Cushings syndrom		1	
DE248;Anden form for Cushings syndrom		1	
DE249;Cushings syndrom UNS		1	
DE25;Adrenogenitale sygdomme		1	
DE250;Medfødt adrenogenitalt syndrom med enzymdefekt		1	
DE250B;Medfødt 21-hydroxylasemangel		1	
DE250C;Medfødt salttabende binyrebarkhyperplasi		1	
DE250D;Medfødt binyrebarkhyperplasi		1	
DE258;Anden form for adrenogenitalt syndrom		1	
DE258A;Idiopatisk adrenogenitalt syndrom		1	
DE259;Adrenogenitalt syndrom UNS		1	

DE26;Hyperaldosteronisme		1	
DE260;Primær hyperaldosteronisme		1	
DE260B;Primær hyperaldosteronisme ved bilateral binyrehyperplasi		1	
DE261;Sekundær hyperaldosteronisme		1	
DE268;Anden form for hyperaldosteronisme		1	
DE268A;Bartters syndrom		1	
DE269;Hyperaldosteronisme UNS		1	
DE27;Andre binyresygdomme		1	
DE270;Anden øget binyrebarksekretion		1	
DE270A;Øget ACTH produktion uden Cushings sygdom		1	
DE270B;Præmatur adrenarche		1	
DE271;Primær binyrebarkinsufficiens		1	
DE271A;Addisons sygdom		1	
DE271B;Autoimmun adrenalitis		1	
DE272;Addisonkrise		1	
DE273;Binyrebarkinsufficiens forårsaget af lægemiddel		1	
DE274;Anden og ikke specificeret binyrebarkinsufficiens		1	
DE274A;Binyrebarkinsufficiens UNS		1	
DE274B;Blødning i binyre		1	
DE274C;Anden binyrebarkinsufficiens		1	
DE274D;Hypoaldosteronisme		1	
DE274E;Binyreinfarkt		1	
DE275;Øget katekolaminsekretion		1	
DE278;Anden binyresygdom		1	
DE278A;Abnormt kortisonbindende globulin		1	
DE279;Binyresygdom UNS		1	
DE28;Forstyrrelse i æggestokkenes funktion		1	
DE280;Øget østrogen niveau		1	
DE281;Øget androgen niveau		1	
DE282;Polycystisk ovariesyndrom (PCOS)		1	
DE282A;Sklerocystisk ovariesyndrom		1	
DE282C;Polycystiske ovarier uden anovulation		1	

Supplement 2 Nielsen et al. Mortality after paediatric emergency calls for patients with and without pre-existing comorbidity (2023)

DE283;Primær ovarial hypofunktion		1		
DE283A;Præmatur menopause UNS		1		
DE283B;Østrogenresistenssyndrom		1		
DE288;Anden forstyrrelse i æggestokkenes funktion		1		
DE288A;Ovarial hyperfunktion UNS		1		
DE289;Forstyrrelse i æggestokkenes funktion UNS		1		
DE29;Forstyrrelse i testiklernes funktion		1		
DE290;Testikulær hyperfunktion		1		
DE291;Testikulær hypofunktion		1		
DE291A;Defekt testikulær androgensyntese		1		
DE291B;5-alfa-reduktase mangel med mandlig pseudohermafroditisme		1		
DE291C;Testikulær hypogonadisme UNS		1		
DE298;Anden forstyrrelse i testiklernes funktion		1		
DE299;Forstyrrelse i testiklernes funktion UNS		1		
DE30;Hormonelle forstyrrelser i puberteten IKA		1		
DE300;Konstitutionelt forsinket pubertet		1		
DE301;For tidlig pubertet		1		
DE308;Anden hormonel forstyrrelse i puberteten		1		
DE308A;Præmatur thelarche		1		
DE309;Hormonel forstyrrelse i puberteten UNS		1		
DE31;Funktionsforstyrrelser i flere endokrine kirtler		1		
DE310;Autoimmun polyglandulær insufficiens		1		
DE310A;Schmidts syndrom		1		
DE311;Polyglandulær hyperfunktion		1		
DE318;Anden polyglandulær funktionsforstyrrelse		1		
DE319;Polyglandulær funktionsforstyrrelse UNS		1		
DE32;Sygdomme i thymus		1		
DE320;Varig hyperplasi af thymus		1		
DE321;Absces i thymus		1		
DE328;Anden sygdom i thymus		1		
DE329;Sygdom i thymus UNS		1		
DE34;Sygdomme i andre endokrine kirtler		1		

DE340;Karcinoidt syndrom		1	
DE341;Anden øget sekretion af intestinale hormoner		1	
DE342;Ektopisk hormonsekretion IKA		1	
DE343;Lille højde IKA		1	
DE343A;Dværgvækst af Laron-type		1	
DE343B;Konstitutionel dværgvækst		1	
DE343C;Nanismus psychosocialis		1	
DE344;Konstitutionel kæmpevækst		1	
DE345;Androgen-insensitivitetssyndrom		1	
DE345A;Testikulær feminisering		1	
DE345B;Mandlig pseudohermafroditisme ved androgeninsensivitet		1	
DE348;Anden sygdom i endokrine kirtler		1	
DE348A;Dysfunktion af corpus pineale		1	
DE348B;Progeria			2
DE348C;Werners syndrom		1	
DE349;Endokrin sygdom UNS		1	
DE35;Endokrine sygdomme som følge af sygdom klassificeret andetsteds		1	
DE350;Sygdom i skjoldbruskkirtel som følge af sygdom klassificeret andetsteds		1	
DE351;Sygdom i binyre som følge af sygdom klassificeret andetsteds		1	
DE358;Anden endokrin sygdom som følge af sygdom klassificeret andetsteds		1	
DE40;Proteinmangelsygdom hos børn		1	
DE409;Proteinmangelsygdom hos børn UNS		1	
DE41;Svækkelse forårsaget af underernæring		1	
DE419;Svækkelse forårsaget af underernæring UNS		1	
DE42;Svær afmagring som følge af protein- og energimangel		1	
DE429;Svær afmagring som følge af proteinmangel UNS		1	
DE43;Ikke specificeret svær protein- og energimangelsygdom		1	
DE439;Svær protein- og energimangelsygdom UNS		1	
DE439A;Hungerødem		1	
DE44;Moderat og mild protein- og energimangelsygdom		1	
DE440;Moderat protein- og energimangelsygdom		1	
DE441;Mild protein- og energimangelsygdom		1	

DE45;Forsinket udvikling som følge af protein- og energimangel		1	
DE459;Nedsat vækst som følge af underernæring		1	
DE46;Ikke specificeret protein- og energiunderernæring		1	
DE469;Protein- og energiunderernæring UNS		1	
DE47;Anden undervægt		1	
DE470;Undervægt som følge af for lavt kalorieindtag, BMI<18,5		1	
DE50;A-vitaminmangel		1	
DE500;A-vitaminmangel med tørhed af konjunktiva		1	
DE501;A-vitaminmangel med Bitots pletter og øjentørhed		1	
DE502;A-vitaminmangel med tørhed i hornhinde		1	
DE503;A-vitaminmangel med kronisk sår og tørhed i hornhinde		1	
DE504;A-vitaminmangel med keratomalaci		1	
DE505;A-vitaminmangel med natteblindhed		1	
DE506;A-vitaminmangel med ar i hornhinden efter øjentørhed		1	
DE507;A-vitaminmangel med andre øjensymptomer		1	
DE507A;A-vitaminmangel med xeroftalmi UNS		1	
DE508;A-vitaminmangel med anden manifestation		1	
DE508B;A-vitaminmangel med follikulær keratose		1	
DE508C;A-vitaminmangel med xeroderma		1	
DE509;A-vitaminmangel UNS		1	
DE51;Tiaminmangel		1	
DE511;Beriberi		1	
DE511A;Tør beriberi		1	
DE511B;Våd beriberi		1	
DE512;Wernickes encefalopati		1	
DE518;Tiaminmangel med anden manifestation		1	
DE519;Tiaminmangel UNS		1	
DE52;Niacinmangel		1	
DE529;Niacinmangel UNS		1	
DE529A;Alkoholisk pellagra		1	
DE53;Andre B-vitamin mangeltilstande		1	
DE530;Riboflavinmangel		1	

DE531;Pyridoxinmangel		1	
DE538;Anden B-vitaminmangel		1	
DE538A;Folinsyremangel		1	
DE538B;Pantotensyremangel		1	
DE538C;Biotinmangel		1	
DE538D;Cyanokobalaminmangel		1	
DE538E;Folatmangel		1	
DE539;B-vitaminmangel UNS		1	
DE54;C-vitaminmangel		1	
DE549;C-vitaminmangel UNS		1	
DE55;D-vitaminmangel		1	
DE550;Aktiv rakitis		1	
DE559;D-vitaminmangel UNS		1	
DE56;Andre vitaminmangel-tilstande		1	
DE560;E-vitaminmangel		1	
DE561;K-vitaminmangel		1	
DE568;Anden vitaminmangel		1	
DE569;Vitaminmangel UNS		1	
DE58;Ernæringsbetinget kalciummangel		1	
DE589;Kalciummangel UNS		1	
DE59;Ernæringsbetinget selenmangel		1	
DE599;Selenmangel UNS		1	
DE60;Ernæringsbetinget zinkmangel		1	
DE609;Zinkmangel UNS		1	
DE61;Mangel på andre sporelementer		1	
DE610;Kobbermangel		1	
DE611;Jernmangel		1	
DE612;Magnesiummangel		1	
DE613;Manganmangel		1	
DE614;Krommangel		1	
DE615;Molybdænmangel		1	
DE616;Vanadiummangel		1	

DE617;Samtidig mangel på flere sporelementer		1	
DE618;Mangel på andet sporelement		1	
DE619;Sporelementmangel UNS		1	
DE63;Andre ernæringsbetingede mangeltilstande		1	
DE630;Mangel på essentielle fedtsyrer (EFA)		1	
DE631;Fejlernæring som følge af misforhold i fødens sammensætning		1	
DE638;Anden kostmangel		1	
DE639;Kostmangel UNS		1	
DE64;Følger efter underernæring og ernæringsbetingede mangeltilstande		1	
DE640;Følger efter protein-energimangel		1	
DE641;Følger efter A-vitaminmangel		1	
DE642;Følger efter C-vitaminmangel		1	
DE643;Følger efter rakitis		1	
DE648;Følger efter anden ernæringsbetinget mangeltilstand		1	
DE648A;Pseudoglukagonom syndrom		1	
DE648B;KZ-syndrom		1	
DE649;Følger efter ernæringsbetinget mangeltilstand UNS		1	
DE65;Lokaliseret fedme		1	
DE658;Anden lokaliseret fedme		1	
DE658A;Abdomen pendens		1	
DE658B;Abdomen pendens postgestationis		1	
DE659;Lokaliseret fedme UNS		1	
DE66;Overvægt og fedme		1	
DE660;Fedme som følge af for stort kalorieindtag		1	
DE660A;Overvægt (BMI 25-29,9)		1	
DE660B;Fedme (BMI 30-34,9)		1	
DE660C;Svær fedme (BMI 35-39,9)		1	
DE660E;Ekstrem fedme, BMI 40-44.9		1	
DE660F;Ekstrem fedme, BMI 45-49.9		1	
DE660G;Ekstrem fedme, BMI 50-54.9		1	
DE660H;Ekstrem fedme, BMI 55+		1	
DE661;Fedme forårsaget af lægemiddel		1	

DE662;Ekstrem fedme med hypoventilation		1	
DE668;Anden overvægt eller fedme		1	
DE669;Overvægt UNS		1	
DE67;Anden overernæring		1	
DE670;A-hypervitaminose		1	
DE671;Hyperkarotinæmi		1	
DE672;B6-hypervitaminose		1	
DE673;D-hypervitaminose		1	
DE678;Anden overernæring		1	
DE68;Følger af overernæring		1	
DE689;Følger af overernæring UNS		1	
DE70;Forstyrrelser i omsætningen af aromatiske aminosyrer		1	
DE700;Klassisk fenylketonuri		1	
DE701;Hyperfenylalaninæmi		1	
DE702;Forstyrrelser i tyrosinomsætningen		1	
DE702A;Alkaptonuri		1	
DE702B;Hypertyrosinæmi		1	
DE703;Albinisme		1	
DE703A;Okulær albinisme		1	
DE703B;Okulokutan albinisme		1	
DE703F;Piebaldisme		1	
DE708;Anden forstyrrelse i omsætningen af aromatiske aminosyrer		1	
DE708A;Forstyrrelse i tryptofanomsætningen		1	
DE708B;Forstyrrelse i histidinomsætningen		1	
DE709;Forstyrrelser i omsætningen af aromatiske aminosyrer UNS		1	
DE71;Forstyrrelser i omsætningen af forgrenede aminosyrer og fedtsyrer		1	
DE710;Ahornsirup-urin-sygdom (MSUD)		1	
DE711;Anden forstyrrelse i omsætningen af forgrenede aminosyrer		1	
DE711A;Hyperleucin-isoleucinæmi		1	
DE711B;Hypervalinæmi		1	
DE711C;Isovalerinacidæmi		1	
DE711D;Metylmalonacidæmi		1	

Supplement 2 Nielsen et al. Mortality after paediatric emergency calls for patients with and without pre-existing comorbidity (2023)

DE711E;Propionacidæmi		1	
DE712;Forstyrrelse i omsætningen af forgrenede aminosyrer UNS		1	
DE713;Forstyrrelse i fedtomsætningen		1	
DE713A;Adrenoleukodystrofi (Addison-Schilder)			2
DE713B;Muskel-karnitinpalmityltransferasemangel		1	
DE713C;Acyl-CoA dehydrogenasemangel		1	
DE713C1;Kortkædet acyl-CoA dehydrogenasemangel (SCADD)		1	
DE713C2;Mellemkædet Acyl-CoA dehydrogenasemangel (MCADD)		1	
DE713C3;Langkædet 3-OH-acyl-CoA dehydrogenasemangel (LCHADD)		1	
DE713C4;Meget langkædet acyl-CoA dehydrogenasemangel (VLCADD)		1	
DE72;Andre forstyrrelser i aminosyreomsætningen		1	
DE720;Forstyrrelse i aminosyretransporten		1	
DE720A;Cystinose		1	
DE720B;Cystinuri		1	
DE720C;Fanconis syndrom		1	
DE720D;Hartnups sygdom		1	
DE720E;Lowes syndrom		1	
DE721;Forstyrrelse i omsætningen af svovlholdige aminosyrer		1	
DE721A;Cystationinuri		1	
DE721B;Homocystinuri		1	
DE721C;Metioninæmi		1	
DE721D;Sulfitoxidasemangel			2
DE721E;Methylentetrahydrofolat reduktase defekt		1	
DE722;Forstyrrelse i urinstofcyklus			2
DE722A;Argininæmi			2
DE722B;Arginosuccinaciduri			2
DE722C;Citrullinæmi			2
DE722D;Hyperammonæmi			2
DE723;Forstyrrelse i lysin og hydroxylysinomsætningen		1	
DE723A;Glutaraciduri		1	
DE723B;Hydroxylysinæmi		1	
DE723C;Hyperlysinæmi		1	

DE724;Forstyrrelse i ornitinomsætningen		1	
DE725;Forstyrrelser i glycinomsætningen			2
DE725A;Nonketotisk hyperglycinæmi			2
DE725B;Hyperhydroxyprolinæmi			2
DE725C;Hyperprolinæmi type I og II			2
DE725D;Sarkosinæmi			2
DE728;Anden forstyrrelse i aminosyreomsætningen		1	
DE728A;Forstyrrelse i beta-aminosyreomsætningen		1	
DE728B;Forstyrrelse i gamma-glutamylcyklus		1	
DE728E;Prolidasemangel		1	
DE728F;2,8-dihydroxyadenuri		1	
DE729;Forstyrrelse i aminosyreomsætningen UNS		1	
DE73;Laktoseintolerans		1	
DE730;Medfødt laktasemangel		1	
DE731;Erhvervet laktasemangel		1	
DE738;Anden laktoseintolerans		1	
DE738A;Laktosemalabsorption		1	
DE739;Laktoseintolerans UNS		1	
DE74;Andre forstyrrelser i kulhydratomsætningen		1	
DE740;Forstyrrelse i glykogenaflejringen		1	
DE740A;Glykogenose type IV			2
DE740B;Glykogenose type III		1	
DE740C;Leverfosforylase-mangel		1	
DE740E;Hjerteglykogenose			2
DE740F;Glykogenose type VI		1	
DE740G;Glykogenose type V		1	
DE740H;Glykogenose type II			2
DE740I;Glykogenose type VII			2
DE740J;Glykogenose type I		1	
DE741;Forstyrrelse i fruktoseomsætningen		1	
DE741B;Fruktose-1,6-difosfatasemangel		1	
DE741C;Essentiel fruktosuri		1	

DE741D;Medfødt fruktoseintolerance		1	
DE742;Forstyrrelse i galaktoseomsætningen		1	
DE742A;Galaktokinasemangel		1	
DE742B;Galaktosæmi		1	
DE743;Anden forstyrrelse i optagelsen af kulhydrat i tarmen		1	
DE743A;Sukrasemangel		1	
DE743B;Glukose-galaktosemalabsorption		1	
DE744;Forstyrrelse i pyruvatomsætningen og glukoneogenesen			2
DE744A;Fosfoenol-pyruvatkarboxykinasemangel			2
DE744B;Pyruvatdehydrogenasemangel			2
DE744C;Pyruvatkarboxylasemangel			2
DE748;Anden forstyrrelse i kulhydratomsætningen		1	
DE748A;Renal glukosuri		1	
DE748B;Oxalose		1	
DE748C;Hyperoxaluri UNS		1	
DE748D;Essentiel pentosuri		1	
DE748E;Enterisk hyperoxaluri		1	
DE748G;Idiopatisk hyperoxaluri		1	
DE749;Forstyrrelse i kulhydratomsætningen UNS		1	
DE75;Forstyrrelse i sfingolipidomsætningen og andre lipidaflejringsforstyrrelser			2
DE750;GM2-gangliosidose			2
DE750A;GM2-gangliosidose hos voksen		1	
DE750B;GM2-gangliosidose hos barn UNS			2
DE750C;Sandhoffs sygdom (type2)			2
DE750D;Tay-Sachs' sygdom			2
DE751;Anden gangliosidose			2
DE751A;Gangliosidose UNS			2
DE751B;GM1-gangliosidose			2
DE751C;GM3-gangliosidose			2
DE751D;Mukolipidose IV			2
DE752;Anden sfingolipidose			2
DE752A;Farbers syndrom			2

DE752B;Fabrys sygdom		1	
DE752C;Gauchers sygdom			2
DE752D;Krabbes sygdom			2
DE752E;Metakromatisk leukodystrofi			2
DE752F;Lipidosis cerebrosidea			2
DE752G;Lipidosis sphingomyelinea			2
DE752H;Niemann-Picks sygdom			2
DE753;Sfingolipidose UNS			2
DE754;Neuronal ceroid lipofuskinose			2
DE754B;Neuronal ceroid lipofuskinose type 2			2
DE754C;Neuronal ceroid lipofuskinose type 4		1	
DE754E;Neuronal ceroid lipofuskinose type 3			2
DE754F;Neuronal ceroid lipofuskinose type 1			2
DE755;Anden lipidaflejringssygdom			2
DE755A;Cholesterosis cerebrotendinosa		1	
DE755B;Wolmans sygdom			2
DE756;Lipidaflejringssygdom UNS			2
DE76;Forstyrrelser i glukosaminoglykanomsætningen			2
DE760;Mukopolysakkaridose type I			2
DE760A;Hurler-Scheie's syndrom			2
DE760B;Hurlers syndrom			2
DE760C;Scheie's syndrom		1	
DE761;Mukopolysakkaridose type II			2
DE761A;Hunter’s syndrom (findes ikke i SKS)			2
DE762;Anden mukopolysakkaridose			2
DE762A;Beta-glukuronidasemangel		1	
DE762B;Mukopolysakkaridose type VI			2
DE762C;Mukopolysakkaridose type IV			2
DE762E;Mukopolysakkaridose type III			2
DE762G;Mukopolysakkaridose type VII			2
DE762H;Mukopolysakkaridose type IX		1	
DE763;Mukopolysakkaridose UNS			2

Supplement 2 Nielsen et al. Mortality after paediatric emergency calls for patients with and without pre-existing comorbidity (2023)

DE768;Anden forstyrrelse i glukosaminoglykanomsætningen			2
DE769;Forstyrrelse i glukosaminoglykanomsætningen UNS			2
DE77;Forstyrrelser i glykoproteinomsætningen			2
DE770;Posttranslational defekt i lysosomale enzymer			2
DE770A;Mukolipidose II			2
DE770B;Mukolipidose III		1	
DE771;Defekt i glykoproteinnedbrydningen			2
DE771A;Aspartylglukosaminuri		1	
DE771B;Fukosidose			2
DE771C;Mannosidose			2
DE771D;Mukolipidose I			2
DE778;Anden forstyrrelse i glykoproteinomsætningen			2
DE779;Forstyrrelse i glykoproteinomsætningen UNS			2
DE78;Forstyrrelser i lipoproteinomsætningen og andre lipidæmier		1	
DE780;Hyperkolesterolæmi		1	
DE780A;Hyperbetalipoproteinæmi		1	
DE780B;Familiær hyperkolesterolæmi		1	
DE780B1;Familiær hyperkolesterolæmi, heterozygot		1	
DE780B2;Familiær hyperkolesterolæmi, homozygot		1	
DE780C;Hyperlipidæmi gruppe A		1	
DE780D;Fredrickson type IIa-hyperlipoproteinæmi		1	
DE780E;Low-density-lipoprotein hyperlipoproteinæmi		1	
DE781;Hyperglyceridæmi		1	
DE781A;Endogen hyperglyceridæmi		1	
DE781B;Hyperlipidæmi gruppe B		1	
DE781C;Fredrickson type IV hyperlipoproteinæmi		1	
DE781D;Very-low-density-lipoprotein hyperlipoproteinæmi		1	
DE781E;Hyperpræbetalipoproteinæmi		1	
DE782;Blandet hyperlipidæmi		1	
DE782A;Betalipoproteinæmi		1	
DE782B;Hyperbetalipoproteinæmi med præbetalipoproteinæmi		1	
DE782C;Hyperkolesterolæmi med endogen hyperglyceridæmi		1	

DE782D;Hyperlipidæmi gruppe C		1	
DE782E;Fredrickson type IIb/III hyperlipoproteinæmi		1	
DE782F;Xanthoma tuberosum		1	
DE782G;Xanthoma tubo-eruptivum		1	
DE782H;Xanthoma planum		1	
DE782J;Xanthomatose		1	
DE783;Hyperkylomikronæmi		1	
DE783A;Hyperglyceridæmi, blandingsform		1	
DE783B;Hyperlipidæmi gruppe D		1	
DE783C;Fredrickson, type I/V hyperlipoproteinæmi		1	
DE784;Anden hyperlipidæmi		1	
DE784A;Familiær kombineret hyperlipidæmi		1	
DE785;Hyperlipidæmi UNS		1	
DE786;Lipoproteinmangel		1	
DE786A;Aalfalipoproteinæmi		1	
DE786B;Abetalipoproteinæmi		1	
DE786C;High-density-lipoprotein-mangel		1	
DE786E;Hypobetalipoproteinæmi		1	
DE786F;Lecitin-kolesterol-acyltransferase-mangel		1	
DE788;Anden forstyrrelse i lipoproteinomsætningen		1	
DE788A;Subkutan lipogranulomatose		1	
DE788B;Hyalinose i hud eller slimhinde		1	
DE789;Forstyrrelse i lipoproteinomsætningen UNS		1	
DE79;Forstyrrelser i purin- eller pyrimidinomsætningen		1	
DE790;Asymptomatisk hyperurikæmi		1	
DE791;Lesch-Nyhans syndrom			2
DE798;Anden forstyrrelse i purin- eller pyrimidinomsætningen		1	
DE798A;Arvelig xantinuri		1	
DE798B;Uratsten med hyperurikæmi		1	
DE798C;Uratsten uden hyperurikæmi		1	
DE799;Forstyrrelse i purin- eller pyrimidinomsætningen UNS		1	
DE80;Forstyrrelser i porfyrin- eller bilirubinomsætningen		1	

Supplement 2 Nielsen et al. Mortality after paediatric emergency calls for patients with and without pre-existing comorbidity (2023)

DE800;Familiær erytropoietisk porfyri		1	
DE801;Porphyria cutanea tarda		1	
DE802;Anden form for porfyri		1	
DE802A;Hereditær koproporfyri		1	
DE802B;Intermitterende porfyri		1	
DE802C;Porphyria hepatica		1	
DE802D;Porfyri UNS		1	
DE802E;Porphyria variegata		1	
DE802F;Porphyria e dialysis		1	
DE802G;Porphyria hepatoerythropoietica		1	
DE803;Katalase- eller peroxidasedefekt		1	
DE803A;Akatalasæmi		1	
DE803B;Forstyrrelse af peroxidaseomsætningen		1	
DE804;Hyperbilirubinæmi type 1		1	
DE804A;Icterus non haemolyticus congenitus familiaris		1	
DE805;Crigler-Najjars sygdom			2
DE806;Anden forstyrrelse i bilirubinomsætningen		1	
DE806A;Hyperbilirubinæmi type 2		1	
DE806B;Rotors syndrom		1	
DE807;Forstyrrelse i bilirubinomsætningen UNS		1	
DE83;Forstyrrelser i mineralomsætningen		1	
DE830;Forstyrrelser i kobberomsætningen		1	
DE830A;Menkes' syndrom			2
DE830B;Wilsons sygdom		1	
DE831;Forstyrrelser i jernomsætningen		1	
DE831A;Hæmokromatose		1	
DE832;Forstyrrelser i zinkomsætningen		1	
DE832A;Acrodermatitis enteropathica		1	
DE833;Forstyrrelser i fosforomsætningen og fosfataser		1	
DE833A;Hypofosfatæmi		1	
DE833A1;Familiær hypofosfatæmi		1	
DE833B;D-vitaminresistent rakitis		1	

Supplement 2 Nielsen et al. Mortality after paediatric emergency calls for patients with and without pre-existing comorbidity (2023)

DE834;Forstyrrelse i magnesiumomsætningen		1	
DE834A;Hypermagnesiæmi		1	
DE834B;Hypomagnesiæmi		1	
DE835;Forstyrrelser i kalciumomsætningen		1	
DE835A;Familiær hypokalcurisk hyperkalcæmi		1	
DE835B;Idiopatisk hyperkalciuri		1	
DE835C;Hyperkalcæmi UNS		1	
DE835D;Hypokalcæmi UNS		1	
DE838;Anden forstyrrelse i mineralomsætningen		1	
DE839;Forstyrrelse i mineralomsætningen UNS		1	
DE84;Cystisk fibrose		1	
DE840;Cystisk fibrose med lungemanifestationer		1	
DE841;Cystisk fibrose med tarmmanifestationer		1	
DE848;Cystisk fibrose med anden manifestation		1	
DE849;Cystisk fibrose UNS		1	
DE85;Amyloidose			2
DE850;Arvelig amyloidose uden neurologiske symptomer			2
DE850A;Familiær middelhavsfeber			2
DE851;Arvelig nerveamyloidose			2
DE851A;Amyloidose med polyneuropati			2
DE852;Arvelig amyloidose UNS			2
DE853;Sekundær systemisk amyloidose			2
DE853B;Amyloidosis ved hæmodialyse			2
DE854;Lokaliseret amyloidose			2
DE858;Anden amyloidose			2
DE858A;AL amyloidose			2
DE859;Amyloidose UNS			2
DE86;Udtørring og nedsat ekstracellulærvolumen	0		
DE869;Volumennedsættelse af plasma eller ekstracellulær væske	0		
DE869A;Dehydrering	0		
DE869B;Hypovolæmi uden shock	0		
DE87;Andre forstyrrelser i vand-, elektrolyt- og syre-basebalance	0		

DE870;Hyperosmolalitet eller hypernatriæmi	0		
DE870A;Hypernatriæmi	0		
DE870B;Hyperosmolalitet	0		
DE871;Hypoosmolalitet eller hyponatriæmi	0		
DE871A;Hyponatriæmi	0		
DE871B;Hypoosmolalitet	0		
DE872;Acidose	0		
DE872A;Laktacidose	0		
DE872B;Metabolisk acidose	0		
DE872C;Respiratorisk acidose	0		
DE873;Alkalose	0		
DE873A;Metabolisk alkalose	0		
DE873B;Respiratorisk alkalose	0		
DE874;Blandet forstyrrelse i syre-basebalancen	0		
DE875;Hyperkaliæmi	0		
DE876;Hypokaliæmi	0		
DE877;Væskeoverskud	0		
DE877A;Væskeretention	0		
DE878;Forstyrrelse i vand- eller elektrolytbalancen IKA	0		
DE878A;Hyperkloræmi	0		
DE878B;Hypokloræmi	0		
DE878C;Idiopatisk hypocitraturi	0		
DE88;Andre metaboliske forstyrrelser		1	
DE880;Forstyrrelse i plasmaproteinomsætningen IKA		1	
DE880A;Alfa-1-antitrypsinmangel		1	
DE880B;Alfa-1-proteinaseinhibitormangel		1	
DE880C;Bisalbuminæmi		1	
DE881;Lipodystrofi IKA		1	
DE881A;Lipodystrofi UNS		1	
DE882;Lipomatose IKA		1	
DE882A;Lipomatosis dolorosa Dercum		1	
DE882B;Lipomatose UNS		1	

Supplement 2 Nielsen et al. Mortality after paediatric emergency calls for patients with and without pre-existing comorbidity (2023)

DE883;Tumor lysis-syndrom			2
DE888;Anden metabolisk forstyrrelse		1	
DE888A;Adenolipomatosum subcutis colli		1	
DE888B;Trimetylaminuri		1	
DE888C;Metabolisk syndrom		1	
DE888D;Plasmakolinesterasemangel		1	
DE889;Omsætningsforstyrrelse UNS		1	
DE89;Endokrine forstyrrelser og omsætningsforstyrrelser efter behandling IKA		1	
DE890;Myksødem efter behandling		1	
DE890A;Myksødem efter strålebehandling		1	
DE890B;Myksødem efter operation		1	
DE891;Hypoinsulinæmi efter operation		1	
DE891A;Hyperglykæmi efter pankreatektomi		1	
DE891C;Hypoinsulinæmi efter pankreatektomi		1	
DE892;Hypoparathyroidisme efter behandling		1	
DE892A;Hypoparathyroidisme efter operation		1	
DE892B;Hypoparathyroidisme efter strålebehandling		1	
DE893;Hypopituitarisme efter behandling		1	
DE893A;Hypopituitarisme efter strålebehandling		1	
DE893B;Hypopituitarisme efter operation		1	
DE894;Hypogonadisme efter behandling af ovarier		1	
DE894A;Hypogonadisme efter strålebehandling af ovarier		1	
DE894B;Hypogonadisme efter operation på ovarier		1	
DE895;Hypogonadisme efter behandling af testikler		1	
DE895A;Hypogonadisme efter strålebehandling af testikler		1	
DE895B;Hypogonadisme efter operation på testikler		1	
DE896;Nedsat produktion af binyrehormon efter behandling		1	
DE898;Anden endokrin forstyrrelse eller omsætningsforstyrrelse efter behandling		1	
DE899;Endokrin forstyrrelse eller omsætningsforstyrrelse efter behandling UNS		1	
DF00;Demens ved Alzheimers sygdom			2
DF000;Demens ved Alzheimers sygdom med tidlig debut			2
DF0000;Demens ved tidlig Alzheimers sygdom uden andre symptomer			2

Supplement 2 Nielsen et al. Mortality after paediatric emergency calls for patients with and without pre-existing comorbidity (2023)

DF0001;Demens ved tidlig Alzheimers sygdom med paranoide symptomer			2
DF0002;Demens ved tidlig Alzheimers sygdom med hallucinationer			2
DF0003;Demens ved tidlig Alzheimers sygdom med depressive symptomer			2
DF0004;Demens ved tidlig Alzheimers sygdom med blandede symptomer			2
DF001;Demens ved Alzheimers sygdom med sen debut			2
DF0010;Demens ved sen Alzheimers sygdom uden andre symptomer			2
DF0011;Demens ved sen Alzheimers sygdom med paranoide symptomer			2
DF0012;Demens ved sen Alzheimers sygdom med hallucinationer			2
DF0013;Demens ved sen Alzheimers sygdom med depressive symptomer			2
DF0014;Demens ved sen Alzheimers sygdom med blandede symptomer			2
DF002;Demens ved Alzheimers sygdom af atypisk eller blandet type			2
DF0020;Demens ved atypisk Alzheimers sygdom uden andre symptomer			2
DF0021;Demens ved atypisk Alzheimers sygdom med paranoide symptomer			2
DF0022;Demens ved atypisk Alzheimers sygdom med hallucinationer			2
DF0023;Demens ved atypisk Alzheimers sygdom med depressive symptomer			2
DF0024;Demens ved atypisk Alzheimers sygdom med blandede symptomer			2
DF009;Demens ved Alzheimers sygdom UNS			2
DF0090;Demens uden andre symptomer ved Alzheimers sygdom UNS			2
DF0091;Demens med paranoide symptomer ved Alzheimers sygdom UNS			2
DF0092;Demens med hallucinationer ved Alzheimers sygdom UNS			2
DF0093;Demens med depressive symptomer ved Alzheimers sygdom UNS			2
DF0094;Demens med blandede symptomer ved Alzheimers sygdom UNS			2
DF01;Vaskulær demens			2
DF010;Vaskulær demens med akut indsætten			2
DF0100;Vaskulær demens med akut indsætten uden andre symptomer			2
DF0101;Vaskulær demens med akut indsætten med paranoide symptomer			2
DF0102;Vaskulær demens med akut indsætten med hallucinationer			2
DF0103;Vaskulær demens med akut indsætten med depressive symptomer			2
DF0104;Vaskulær demens med akut indsætten med blandede symptomer			2
DF011;Multiinfarktdemens			2
DF0110;Multiinfarktdemens uden andre symptomer			2
DF0111;Multiinfarktdemens med paranoide symptomer			2

Supplement 2 Nielsen et al. Mortality after paediatric emergency calls for patients with and without pre-existing comorbidity (2023)

DF0112;Multiinfarktdemens med hallucinationer			2
DF0113;Multiinfarktdemens med depressive symptomer			2
DF0114;Multiinfarktdemens med blandede symptomer			2
DF012;Subkortikal vaskulær demens			2
DF0120;Subkortikal vaskulær demens uden andre symptomer			2
DF0121;Subkortikal vaskulær demens med paranoide symptomer			2
DF0122;Subkortikal vaskulær demens med hallucinationer			2
DF0123;Subkortikal vaskulær demens med depressive symptomer			2
DF0124;Subkortikal vaskulær demens med blandede symptomer			2
DF013;Blandet kortikal og subkortikal vaskulær demens			2
DF0130;Blandet kortikal og subkortikal vaskulær demens uden andre symptomer			2
DF0131;Blandet kortikal og subkortikal vaskulær demens med paranoide symptomer			2
DF0132;Blandet kortikal og subkortikal vaskulær demens med hallucinationer			2
DF0133;Blandet kortikal og subkortikal vaskulær demens med depressive symptomer			2
DF0134;Blandet kortikal og subkortikal vaskulær demens med blandede symptomer			2
DF018;Anden vaskulær demens			2
DF0180;Anden vaskulær demens uden andre symptomer			2
DF0181;Anden vaskulær demens med paranoide symptomer			2
DF0182;Anden vaskulær demens med hallucinationer			2
DF0183;Anden vaskulær demens med depressive symptomer			2
DF0184;Anden vaskulær demens med blandede symptomer			2
DF019;Vaskulær demens UNS			2
DF0190;Vaskulær demens uden andre symptomer UNS			2
DF0191;Vaskulær demens med paranoide symptomer UNS			2
DF0192;Vaskulær demens med hallucinationer UNS			2
DF0193;Vaskulær demens med depressive symptomer UNS			2
DF0194;Vaskulær demens med blandede symptomer UNS			2
DF02;Demens ved andre sygdomme klassificeret andetsteds			2
DF020;Demens ved Picks sygdom			2
DF0200;Demens ved Picks sygdom uden andre symptomer			2
DF0201;Demens ved Picks sygdom med paranoide symptomer			2
DF0202;Demens ved Picks sygdom med hallucinationer			2

Supplement 2 Nielsen et al. Mortality after paediatric emergency calls for patients with and without pre-existing comorbidity (2023)

DF0203;Demens ved Picks sygdom med depressive symptomer			2
DF0204;Demens ved Picks sygdom med blandede symptomer			2
DF021;Demens ved Creutzfeldt-Jakobs sygdom			2
DF0210;Demens ved Creutzfeldt-Jakobs sygdom uden andre symptomer			2
DF0211;Demens ved Creutzfeldt-Jakobs sygdom med paranoide symptomer			2
DF0212;Demens ved Creutzfeldt-Jakobs sygdom med hallucinationer			2
DF0213;Demens ved Creutzfeldt-Jakobs sygdom med depressive symptomer			2
DF0214;Demens ved Creutzfeldt-Jakobs sygdom med blandede symptomer			2
DF022;Demens ved Huntingtons sygdom			2
DF0220;Demens ved Huntingtons sygdom uden andre symptomer			2
DF0221;Demens ved Huntingtons sygdom med paranoide symptomer			2
DF0222;Demens ved Huntingtons sygdom med hallucinationer			2
DF0223;Demens ved Huntingtons sygdom med depressive symptomer			2
DF0224;Demens ved Huntingtons sygdom med blandede symptomer			2
DF023;Demens ved Parkinsons sygdom			2
DF0230;Demens ved Parkinsons sygdom uden andre symptomer			2
DF0231;Demens ved Parkinsons sygdom med paranoide symptomer			2
DF0232;Demens ved Parkinsons sygdom med hallucinationer			2
DF0233;Demens ved Parkinsons sygdom med depressive symptomer			2
DF0234;Demens ved Parkinsons sygdom med blandede symptomer			2
DF024;Demens ved HIV-sygdom			2
DF0240;Demens ved HIV-sygdom uden andre symptomer			2
DF0241;Demens ved HIV-sygdom med paranoide symptomer			2
DF0242;Demens ved HIV-sygdom med hallucinationer			2
DF0243;Demens ved HIV-sygdom med depressive symptomer			2
DF0244;Demens ved HIV-sygdom med blandede symptomer			2
DF028;Demens ved anden sygdom klassificeret andetsteds			2
DF0280;Demens uden andre symptomer ved anden sygdom			2
DF0281;Demens med paranoide symptomer ved anden sygdom			2
DF0282;Demens med hallucinationer ved anden sygdom			2
DF0283;Demens med depressive symptomer ved anden sygdom			2
DF0284;Demens med blandede symptomer ved anden sygdom			2

Supplement 2 Nielsen et al. Mortality after paediatric emergency calls for patients with and without pre-existing comorbidity (2023)

DF03;Ikke specificeret demens			2
DF039;Demens UNS			2
DF0390;Demens uden andre symptomer UNS			2
DF0391;Demens med paranoide symptomer UNS			2
DF0392;Demens med hallucinationer UNS			2
DF0393;Demens med depressive symptomer UNS			2
DF0394;Demens med blandede symptomer UNS			2
DF04;Organisk amnestisk syndrom ikke forårsaget af alkohol eller psykoaktive stoffer		1	
DF049;Organisk amnestisk syndrom, ikke fremkaldt af psykoaktive stoffer		1	
DF05;Delir, ikke fremkaldt af alkohol eller psykoaktive stoffer		1	
DF050;Delir uden demens		1	
DF051;Delir ved demens			2
DF058;Andet delir		1	
DF059;Delir UNS		1	
DF06;Andre psykiske lidelser som følge af hjerneskade, hjernedysfunktion eller legemlig sygdom		1	
DF060;Organisk hallucinose		1	
DF061;Organisk kataton sindslidelse		1	
DF062;Organisk paranoid eller skizofreniform sindslidelse		1	
DF063;Organisk affektiv sindslidelse		1	
DF0630;Organisk manisk sindslidelse		1	
DF0631;Organisk bipolar affektiv sindslidelse		1	
DF0632;Organisk depressiv sindslidelse		1	
DF0633;Organisk affektiv blandingstilstand		1	
DF064;Organisk angsttilstand		1	
DF065;Organisk dissociativ tilstand		1	
DF066;Organisk emotionel instabilitet og asteni		1	
DF067;Organisk kognitiv forstyrrelse af lettere grad		1	
DF0670;Organisk kognitiv forstyrrelse af lettere grad ikke associeret med somatisk sygdom		1	
DF0671;Organisk kognitiv forstyrrelse af lettere grad associeret med somatisk sygdom		1	
DF068;Anden organisk psykisk lidelse		1	
DF069;Organisk psykisk lidelse UNS		1	
DF07;Personligheds- og adfærdsforstyrrelser forårsaget af sygdom, beskadigelse eller dysfunktion af hjernen		1	

DF070;Organisk personlighedsforstyrrelse		1	
DF071;Postencefalitisk syndrom		1	
DF072;Posttraumatisk hjernesyndrom		1	
DF078;Anden organisk personligheds- eller adfærdsforstyrrelse		1	
DF079;Organisk personligheds- eller adfærdsforstyrrelse UNS		1	
DF09;Ikke specificeret organisk eller symptomatisk mental lidelse eller personlighedsforstyrrelse		1	
DF099;Organisk eller symptomatisk mental lidelse eller personlighedsforstyrrelse UNS		1	
DF10;Psykiske lidelser og adfærdsmæssige forstyrrelser forårsaget af brug af alkohol		1	
DF100;Akut alkoholintoksikation	0		
DF1000;Akut ukompliceret alkoholintoksikation	0		
DF1001;Akut alkoholintoksikation med fysisk traume	0		
DF1002;Akut alkoholintoksikation med andre somatiske komplikationer	0		
DF1003;Akut alkoholintoksikation med delir	0		
DF1004;Akut alkoholintoksikation med perceptionsforvrængninger	0		
DF1005;Akut alkoholintoksikation med koma	0		
DF1006;Akut alkoholintoksikation med kramper	0		
DF1007;Akut alkoholintoksikation med patologisk rus	0		
DF101;Skadelig brug af alkohol		1	
DF102;Alkoholafhængighedssyndrom		1	
DF1020;Alkoholafhængighedssyndrom men afholdende		1	
DF1021;Alkoholafhængighedssyndrom men afholdende i beskyttet miljø		1	
DF1022;Alkoholafhængighedssyndrom i vedligeholdelses- eller substitutionsbehandling		1	
DF1023;Alkoholafhængighedssyndrom men afholdende under aversiv medicinsk behandling		1	
DF1024;Alkoholafhængighedssyndrom aktivt brugende		1	
DF1025;Alkoholafhængighedssyndrom med kontinuerligt brug		1	
DF1026;Alkoholafhængighedssyndrom med episodisk brug		1	
DF103;Abstinenstilstand som følge af alkoholbrug		1	
DF1030;Abstinenstilstand som følge af alkoholbrug uden kramper		1	
DF1031;Abstinenstilstand forårsaget af alkoholbrug med kramper		1	
DF104;Delirøs abstinenstilstand som følge af alkoholbrug		1	
DF1040;Delirøs abstinenstilstand som følge af alkoholbrug uden kramper		1	
DF1041;Delirøs abstinenstilstand forårsaget af alkoholbrug med kramper		1	

Supplement 2 Nielsen et al. Mortality after paediatric emergency calls for patients with and without pre-existing comorbidity (2023)

DF105;Alkoholpsykose		1	
DF1050;Skizofrenilignende alkoholpsykose		1	
DF1051;Paranoid alkoholpsykose		1	
DF1052;Hallucinatorisk alkoholpsykose		1	
DF1053;Polymorf alkoholpsykose		1	
DF1054;Alkoholpsykose med depressive symptomer		1	
DF1055;Alkoholpsykose med maniske symptomer		1	
DF1056;Alkoholpsykose med blandede symptomer		1	
DF106;Amnestisk syndrom som følge af alkoholbrug		1	
DF107;Sen psykotisk eller residual tilstand forårsaget af alkoholbrug		1	
DF1070;Flashbacks som følge af alkoholbrug		1	
DF1071;Personligheds- eller adfærdsforstyrrelse forårsaget af alkoholbrug		1	
DF1072;Residual affektiv sindslidelse som følge af alkoholbrug		1	
DF1073;Demens som følge af alkoholbrug		1	
DF1074;Anden vedvarende kognitiv forstyrrelse som følge af alkoholbrug		1	
DF1075;Sent indsættende psykotisk tilstand som følge af alkoholbrug		1	
DF108;Anden psykisk lidelse eller adfærdsforstyrrelse forårsaget af alkoholbrug		1	
DF109;Psykisk lidelse eller adfærdsforstyrrelse forårsaget af alkoholbrug UNS		1	
DF11;Psykiske lidelser og adfærdsmæssige forstyrrelser forårsaget af brug af opioider		1	
DF110;Akut opioidintoksikation	0		
DF1100;Akut ukompliceret opioidintoksikation	0		
DF1101;Akut opioidintoksikation med fysisk traume	0		
DF1102;Akut opioidintoksikation med andre somatiske komplikationer	0		
DF1103;Akut opioidintoksikation med delir	0		
DF1104;Akut opioidintoksikation med perceptionsforvrængninger	0		
DF1105;Akut opioidintoksikation med koma	0		
DF1106;Akut opioidintoksikation med kramper	0		
DF111;Skadelig brug af opioider		1	
DF112;Opioidafhængighedssyndrom		1	
DF1120;Opioidafhængighedssyndrom men afholdende		1	
DF1121;Opioidafhængighedssyndrom men afholdende i beskyttet miljø		1	
DF1122;Opioidafhængighedssyndrom i vedligeholdelses- eller substitutionsbehandling		1	

Supplement 2 Nielsen et al. Mortality after paediatric emergency calls for patients with and without pre-existing comorbidity (2023)

DF1123;Opioidafhængighedssyndrom men afholdende under aversiv medikamentel behandling		1	
DF1124;Opioidafhængighedssyndrom aktivt brugende		1	
DF1125;Opioidafhængighedssyndrom med kontinuerligt brug		1	
DF1126;Opioidafhængighedssyndrom med episodisk brug		1	
DF113;Abstinenstilstand som følge af opioidbrug		1	
DF1130;Abstinenstilstand som følge af opioidbrug uden kramper		1	
DF1131;Abstinenstilstand forårsaget af opioidbrug med kramper		1	
DF114;Delirøs abstinenstilstand som følge af opioidbrug		1	
DF1140;Delirøs abstinenstilstand som følge af opioidbrug uden kramper		1	
DF1141;Delirøs abstinenstilstand forårsaget af opioidbrug med kramper		1	
DF115;Opioidpsykose		1	
DF1150;Skizofrenilignende opioidpsykose		1	
DF1151;Paranoid opioidpsykose		1	
DF1152;Hallucinatorisk opioidpsykose		1	
DF1153;Polymorf opioidpsykose		1	
DF1154;Opioidpsykose med depressive symptomer		1	
DF1155;Opioidpsykose med maniske symptomer		1	
DF1156;Opioidpsykose med blandede symptomer		1	
DF117;Sen psykotisk eller residual tilstand forårsaget af opioidbrug		1	
DF1170;Flashbacks som følge af opioidbrug		1	
DF1171;Personligheds- eller adfærdsforstyrrelse forårsaget af opioidbrug		1	
DF1172;Residual affektiv sindslidelse som følge af opioidbrug		1	
DF1173;Demens som følge af opioidbrug		1	
DF1174;Anden vedvarende kognitiv forstyrrelse som følge af opioidbrug		1	
DF1175;Sent indsættende psykotisk tilstand som følge af opioidbrug		1	
DF118;Anden psykisk lidelse eller adfærdsforstyrrelse forårsaget af opioidbrug		1	
DF119;Psykisk lidelse eller adfærdsforstyrrelse forårsaget af opioidbrug UNS		1	
DF12;Psykiske lidelser og adfærdsmæssige forstyrrelser forårsaget af brug af cannabis		1	
DF120;Akut cannabisintoksikation	0		
DF1200;Akut ukompliceret cannabisintoksikation	0		
DF1201;Akut cannabisintoksikation med fysisk traume	0		
DF1202;Akut cannabisintoksikation med andre somatiske komplikationer	0		

DF1203;Akut cannabisintoksikation med delir	0		
DF1204;Akut cannabisintoksikation med perceptionsforvrængninger	0		
DF1205;Akut cannabisintoksikation med koma	0		
DF1206;Akut cannabisintoksikation med kramper	0		
DF121;Skadelig brug af cannabis		1	
DF122;Cannabisafhængighedssyndrom		1	
DF1220;Cannabisafhængighedssyndrom men afholdende		1	
DF1221;Cannabisafhængighedssyndrom men afholdende i beskyttet miljø		1	
DF1222;Cannabisafhængighedssyndrom i vedligeholdelses- eller substitutionsbehandling		1	
DF1223;Cannabisafhængighedssyndrom men afholdende under aversiv medikamentel behandling		1	
DF1224;Cannabisafhængighedssyndrom aktivt brugende		1	
DF1225;Cannabisafhængighedssyndrom med kontinuerligt brug		1	
DF1226;Cannabisafhængighedssyndrom med episodisk brug		1	
DF125;Cannabispsykose		1	
DF1250;Skizofrenilignende cannabispsykose		1	
DF1251;Paranoid cannabispsykose		1	
DF1252;Hallucinatorisk cannabispsykose		1	
DF1253;Polymorf cannabispsykose		1	
DF1254;Cannabispsykose med depressive symptomer		1	
DF1255;Cannabispsykose med maniske symptomer		1	
DF1256;Cannabispsykose med blandede symptomer		1	
DF127;Sen psykotisk eller residual tilstand forårsaget af cannabisbrug		1	
DF1270;Flashbacks som følge af cannabisbrug		1	
DF1271;Personligheds- eller adfærdsforstyrrelse forårsaget af cannabisbrug		1	
DF1272;Residual affektiv sindslidelse som følge af cannabisbrug		1	
DF1273;Demens som følge af cannabisbrug		1	
DF1274;Anden vedvarende kognitiv forstyrrelse som følge af cannabisbrug		1	
DF1275;Sent indsættende psykotisk tilstand som følge af cannabisbrug		1	
DF128;Anden psykisk lidelse eller adfærdsforstyrrelse forårsaget af cannabisbrug		1	
DF129;Psykisk lidelse eller adfærdsforstyrrelse forårsaget af cannabisbrug UNS		1	
DF13;Psykiske lidelser og adfærdsmæssige forstyrrelser forårsaget af brug af sedativa eller hypnotika		1	
DF130;Akut intoksikation med sedativa eller hypnotika	0		

Supplement 2 Nielsen et al. Mortality after paediatric emergency calls for patients with and without pre-existing comorbidity (2023)

DF1300;Akut ukompliceret intoksikation med sedativa eller hypnotika	0		
DF1301;Akut intoksikation med sedativa eller hypnotika med fysisk traume	0		
DF1302;Akut intoksikation med sedativa eller hypnotika med andre somatiske komplikationer	0		
DF1303;Akut intoksikation med sedativa eller hypnotika med delir	0		
DF1304;Akut intoksikation med sedativa eller hypnotika med perceptionsforvrængninger	0		
DF1305;Akut intoksikation med sedativa eller hypnotika med koma	0		
DF1306;Akut intoksikation med sedativa eller hypnotika med kramper	0		
DF131;Skadelig brug af sedativa eller hypnotika		1	
DF132;Afhængighedssyndrom ved brug af sedativa eller hypnotika		1	
DF1320;Afhængighedssyndrom ved brug af sedativa eller hypnotika men afholdende		1	
DF1321;Afhængighedssyndrom ved brug af sedativa eller hypnotika men afholdende i beskyttet miljø		1	
DF1322;Afhængighedssyndrom ved brug af sedativa eller hypnotika i vedligeholdelses- eller substitutionsbehandling		1	
DF1323;Afhængighedssyndrom ved brug af sedativa eller hypnotika men afholdende under aversiv medikamentel behandlir		1	
DF1324;Afhængighedssyndrom ved brug af sedativa eller hypnotika aktivt brugende		1	
DF1325;Afhængighedssyndrom ved brug af sedativa eller hypnotika med kontinuerligt brug		1	
DF1326;Afhængighedssyndrom ved brug af sedativa eller hypnotika med episodisk brug		1	
DF133;Abstinenstilstand som følge af brug af sedativa eller hypnotika		1	
DF1330;Abstinenstilstand som følge af brug af sedativa eller hypnotika uden kramper		1	
DF1331;Abstinenstilstand forårsaget af brug af sedativa eller hypnotika med kramper		1	
DF134;Delirøs abstinenstilstand som følge af brug af sedativa eller hypnotika		1	
DF1340;Delirøs abstinenstilstand som følge af brug af sedativa eller hypnotika uden kramper		1	
DF1341;Delirøs abstinenstilstand forårsaget af brug af sedativa eller hypnotika med kramper		1	
DF135;Psykotisk tilstand ved brug af sedativa eller hypnotika		1	
DF1350;Skizofrenilignende psykotisk tilstand ved brug af sedativa eller hypnotika		1	
DF1351;Paranoid psykotisk tilstand ved brug af sedativa eller hypnotika		1	
DF1352;Hallucinatorisk psykotisk tilstand ved brug af sedativa eller hypnotika		1	
DF1353;Polymorf psykotisk tilstand ved brug af sedativa eller hypnotika		1	
DF1354;Psykotisk tilstand med depressive symptomer ved brug af sedativa eller hypnotika		1	
DF1355;Psykotisk tilstand med maniske symptomer ved brug af sedativa eller hypnotika		1	
DF1356;Blandet psykotisk tilstand ved brug af sedativa eller hypnotika		1	
DF137;Sen psykotisk eller residual tilstand som følge af brug af sedativa eller hypnotika		1	
DF1370;Flashbacks som følge af brug af sedativa eller hypnotika		1	

Supplement 2 Nielsen et al. Mortality after paediatric emergency calls for patients with and without pre-existing comorbidity (2023)

DF1371;Personligheds- eller adfærdsforstyrrelser som følge af brug af sedativa eller hypnotika		1	
DF1372;Residual affektiv sindslidelse som følge af brug af sedativa eller hypnotika		1	
DF1373;Demens som følge af brug af sedativa eller hypnotika		1	
DF1374;Anden vedvarende kognitiv forstyrrelse som følge af brug af sedativa eller hypnotika		1	
DF1375;Sent indsættende psykotisk tilstand som følge af brug af sedativa eller hypnotika		1	
DF138;Anden psykisk lidelse eller adfærdsforstyrrelse som følge af brug af sedativa eller hypnotika		1	
DF139;Psykisk lidelse eller adfærdsforstyrrelse som følge af brug af sedativa eller hypnotika UNS		1	
DF14;Psykiske lidelser og adfærdsmæssige forstyrrelser forårsaget af brug af kokain		1	
DF140;Akut kokainintoksikation	0		
DF1400;Akut ukompliceret kokainintoksikation	0		
DF1401;Akut kokainintoksikation med fysisk traume	0		
DF1402;Akut kokainintoksikation med andre somatiske komplikationer	0		
DF1403;Akut kokainintoksikation med delirøs tilstand	0		
DF1404;Akut kokainintoksikation med perceptionsforvrængninger	0		
DF1405;Akut kokainintoksikation med koma	0		
DF1406;Akut kokainintoksikation med kramper	0		
DF141;Skadelig brug af kokain		1	
DF142;Afhængighedssyndrom ved brug af kokain		1	
DF1420;Afhængighedssyndrom ved brug af kokain men afholdende		1	
DF1421;Afhængighedssyndrom ved brug af kokain men afholdende i beskyttet miljø		1	
DF1422;Afhængighedssyndrom ved brug af kokain i vedligeholdelses- eller substitutionsbehandling		1	
DF1423;Afhængighedssyndrom ved brug af kokain men afholdende under aversiv medikamentel behandling		1	
DF1424;Afhængighedssyndrom ved brug af kokain aktivt brugende		1	
DF1425;Afhængighedssyndrom ved brug af kokain med kontinuerligt brug		1	
DF1426;Afhængighedssyndrom ved brug af kokain med episodisk brug		1	
DF143;Abstinenstilstand som følge af brug af kokain		1	
DF1430;Abstinenstilstand som følge af brug af kokain uden kramper		1	
DF1431;Abstinenstilstand forårsaget af brug af kokain med kramper		1	
DF145;Kokainpsykose		1	
DF1450;Skizofrenilignende kokainpsykose		1	
DF1451;Paranoid kokainpsykose		1	
DF1452;Hallucinatorisk kokainpsykose		1	

Supplement 2 Nielsen et al. Mortality after paediatric emergency calls for patients with and without pre-existing comorbidity (2023)

DF1453;Polymorf kokainpsykose		1	
DF1454;Kokainpsykose med depressive symptomer		1	
DF1455;Kokainpsykose med maniske symptomer		1	
DF1456;Kokainpsykose med blandede symptomer		1	
DF147;Sen psykotisk eller residual tilstand forårsaget af kokainbrug		1	
DF1470;Flashbacks som følge af kokainbrug		1	
DF1471;Personligheds- eller adfærdsforstyrrelse forårsaget af kokainbrug		1	
DF1472;Residual affektiv sindslidelse som følge af kokainbrug		1	
DF1473;Demens som følge af kokainbrug		1	
DF1474;Anden vedvarende kognitiv forstyrrelse som følge af kokainbrug		1	
DF1475;Sent indsættende psykotisk tilstand som følge af kokainbrug		1	
DF148;Anden psykisk lidelse eller adfærdsforstyrrelse forårsaget af kokainbrug		1	
DF149;Psykisk lidelse eller adfærdsforstyrrelse forårsaget af kokainbrug UNS		1	
DF15;Psykiske lidelser og adfærdsmæssige forstyrrelser forårsaget af brug af andre centralstimulerende stoffer		1	
DF150;Akut intoksikation med andet centralstimulerende stof	0		
DF1500;Akut ukompliceret intoksikation med andet centralstimulerende stof	0		
DF1501;Akut intoksikation med andet centralstimulerende stof med fysisk traume	0		
DF1502;Akut intoksikation med andet centralstimulerende stof med andre somatiske komplikation	0		
DF1503;Akut intoksikation med andet centralstimulerende stof med delir	0		
DF1504;Akut intoksikation med andet centralstimulerende stof med perceptionsforvrængninger	0		
DF1505;Akut intoksikation med andet centralstimulerende stof med koma	0		
DF1506;Akut intoksikation med andet centralstimulerende stof med kramper	0		
DF151;Skadelig brug af andet centralstimulerende stof		1	
DF152;Afhængighedssyndrom ved brug af andet centralstimulerende stof		1	
DF1520;Afhængighedssyndrom ved brug af andet centralstimulerende stof men afholdende		1	
DF1521;Afhængighedssyndrom ved brug af andet centralstimulerende stof men afholdende i beskyttet miljø		1	
DF1522;Afhængighedssyndrom ved brug af andet centralstimulerende stof i vedligeholdelses- eller substitutionsbehandling		1	
DF1523;Afhængighedssyndrom ved brug af andet centralstimulerende stof men afholdende under aversiv medikamentel be		1	
DF1524;Afhængighedssyndrom ved brug af andet centralstimulerende stof aktivt brugende		1	
DF1525;Afhængighedssyndrom ved brug af andet centralstimulerende stof med kontinuerligt brug		1	
DF1526;Afhængighedssyndrom ved brug af andet centralstimulerende stof med episodisk brug		1	
DF153;Abstinenstilstand forårsaget af brug af andet centralstimulerende stof		1	

Supplement 2 Nielsen et al. Mortality after paediatric emergency calls for patients with and without pre-existing comorbidity (2023)

DF1530;Abstinenstilstand forårsaget af brug af andre centralstimulerende stoffer uden kramper		1	
DF1531;Abstinenstilstand forårsaget af brug af andre centralstimulerende stoffer med kramper		1	
DF155;Psykotisk tilstand ved brug af andet centralstimulerende stof		1	
DF1550;Skizofrenilignende psykotisk tilstand ved brug af andet centralstimulerende stof		1	
DF1551;Paranoid psykotisk tilstand ved brug af andet centralstimulerende stof		1	
DF1552;Hallucinatorisk psykotisk tilstand ved brug af andet centralstimulerende stof		1	
DF1553;Polymorf psykotisk tilstand ved brug af andet centralstimulerende stof		1	
DF1554;Psykotisk tilstand med depressive symptomer ved brug af andet centralstimulerende stof		1	
DF1555;Psykotisk tilstand med maniske symptomer ved brug af andet centralstimulerende stof		1	
DF1556;Blandet psykotisk tilstand ved brug af andet centralstimulerende stof		1	
DF157;Sen psykotisk eller residual tilstand forårsaget af brug af andet centralstimulerende stof		1	
DF1570;Flashbacks forårsaget af brug af andet centralstimulerende stof		1	
DF1571;Personligheds- eller adfærdsforstyrrelse forårsaget af brug af andet centralstimulerende stof		1	
DF1572;Residual affektiv sindslidelse forårsaget af brug af andet centralstimulerende stof		1	
DF1573;Demens forårsaget af brug af andet centralstimulerende stof		1	
DF1574;Anden vedvarende kognitiv forstyrrelse forårsaget af brug af andet centralstimulerende stof		1	
DF1575;Sent indsættende psykotisk tilstand forårsaget af brug af andet centralstimulerende stof		1	
DF158;Anden psykisk lidelse eller adfærdsforstyrrelse forårsaget af brug af andet centralstimulerende stof		1	
DF159;Psykisk lidelse eller adfærdsforstyrrelse forårsaget af brug af andet centralstimulerende stof UNS		1	
DF16;Psykiske lidelser og adfærdsmæssige forstyrrelser forårsaget af brug af hallucinogener		1	
DF160;Akut hallucinogenintoksikation	0		
DF1600;Akut ukompliceret hallucinogenintoksikation	0		
DF1601;Akut hallucinogenintoksikation med fysisk traume	0		
DF1602;Akut hallucinogenintoksikation med andre somatiske komplikationer	0		
DF1603;Akut hallucinogenintoksikation med delir	0		
DF1604;Akut hallucinogenintoksikation med perceptionsforvrængninger	0		
DF1605;Akut hallucinogenintoksikation med koma	0		
DF1606;Akut hallucinogenintoksikation med kramper	0		
DF161;Skadelig brug af hallucinogen		1	
DF162;Afhængighedssyndrom ved brug af hallucinogen		1	
DF1620;Afhængighedssyndrom ved brug af hallucinogen men afholdende		1	
DF1621;Afhængighedssyndrom ved brug af hallucinogen men afholdende i beskyttet miljø		1	

Supplement 2 Nielsen et al. Mortality after paediatric emergency calls for patients with and without pre-existing comorbidity (2023)

DF1622;Afhængighedssyndrom ved brug af hallucinogen i vedligeholdelses- eller substitutionsbehandling		1	
DF1623;Afhængighedssyndrom ved brug af hallucinogen men afholdende under aversiv medikamentel behandling		1	
DF1624;Afhængighedssyndrom ved brug af hallucinogen aktivt brugende		1	
DF1625;Afhængighedssyndrom ved brug af hallucinogen med kontinuerligt brug		1	
DF1626;Afhængighedssyndrom ved brug af hallucinogen med episodisk brug		1	
DF165;Hallucinogenpsykose		1	
DF1650;Skizofrenilignende hallucinogenpsykose		1	
DF1651;Paranoid hallucinogenpsykose		1	
DF1652;Hallucinatorisk hallucinogenpsykose		1	
DF1653;Polymorf hallucinogenpsykose		1	
DF1654;Hallucinogenpsykose med depressive symptomer		1	
DF1655;Hallucinogenpsykose med maniske symptomer		1	
DF1656;Hallucinogenpsykose med blandede symptomer		1	
DF167;Sen psykotisk eller residual tilstand forårsaget af hallucinogenbrug		1	
DF1670;Flashbacks som følge af hallucinogenbrug		1	
DF1671;Personligheds- eller adfærdsforstyrrelse forårsaget af hallucinogenbrug		1	
DF1672;Residual affektiv sindslidelse som følge af hallucinogenbrug		1	
DF1673;Demens som følge af hallucinogenbrug		1	
DF1674;Anden vedvarende kognitiv forstyrrelse som følge af hallucinogenbrug		1	
DF1675;Sent indsættende psykotisk tilstand som følge af hallucinogenbrug		1	
DF168;Anden psykisk lidelse eller adfærdsforstyrrelse forårsaget af hallucinogenbrug		1	
DF169;Psykisk lidelse eller adfærdsforstyrrelse forårsaget af hallucinogenbrug UNS		1	
DF17;Psykiske lidelser og adfærdsmæssige forstyrrelser forårsaget af brug af tobak		1	
DF170;Akut tobaksintoksikation	0		
DF1700;Akut ukompliceret tobaksintoksikation	0		
DF1701;Akut tobaksintoksikation med fysisk traume	0		
DF1702;Akut tobaksintoksikation med andre somatiske komplikationer	0		
DF1703;Akut tobaksintoksikation med delir	0		
DF1704;Akut tobaksintoksikation med perceptionsforvrængninger	0		
DF1705;Akut tobaksintoksikation med koma	0		
DF1706;Akut tobaksintoksikation med kramper	0		
DF171;Skadelig brug af tobak		1	

DF172;Afhængighedssyndrom ved brug af tobak		1	
DF1720;Afhængighedssyndrom ved brug af tobak men afholdende		1	
DF1721;Afhængighedssyndrom ved brug af tobak men afholdende i beskyttet miljø		1	
DF1722;Afhængighedssyndrom ved brug af tobak i vedligeholdelses- eller substitutionsbehandling		1	
DF1723;Afhængighedssyndrom ved brug af tobak men afholdende under aversiv medikamentel behandling		1	
DF1724;Afhængighedssyndrom ved brug af tobak aktivt brugende		1	
DF1725;Afhængighedssyndrom ved brug af tobak med kontinuerligt brug		1	
DF1726;Afhængighedssyndrom ved brug af tobak med episodisk brug		1	
DF178;Anden psykisk lidelse eller adfærdsforstyrrelse forårsaget af tobaksbrug		1	
DF179;Psykisk lidelse eller adfærdsforstyrrelse forårsaget af tobaksbrug UNS		1	
DF18;Psykiske lidelser og adfærdsmæssige forstyrrelser forårsaget af brug af flygtige opløsningsmidler		1	
DF180;Akut intoksikation med flygtigt opløsningsmiddel	0		
DF1800;Akut ukompliceret intoksikation med flygtige opløsningsmidler	0		
DF1801;Akut intoksikation med flygtige opløsningsmidler med fysisk traume	0		
DF1802;Akut intoksikation med flygtige opløsningsmidler med andre somatiske komplikationer	0		
DF1803;Akut intoksikation med flygtige opløsningsmidler med delir	0		
DF1804;Akut intoksikation med flygtige opløsningsmidler med perceptionsforvrængninger	0		
DF1805;Akut intoksikation med flygtige opløsningsmidler med koma	0		
DF1806;Akut intoksikation med flygtige opløsningsmidler med kramper	0		
DF181;Skadelig brug af flygtigt opløsningsmiddel		1	
DF182;Afhængighedssyndrom ved brug af flygtigt opløsningsmiddel		1	
DF1820;Afhængighedssyndrom ved brug af flygtige opløsningsmidler men afholdende		1	
DF1821;Afhængighedssyndrom ved brug af flygtige opløsningsmidler men afholdende i beskyttet miljø		1	
DF1822;Afhængighedssyndrom ved brug af flygtige opløsningsmidler i vedligeholdelses- eller substitutionsbehandling		1	
DF1823;Afhængighedssyndrom ved brug af flygtige opløsningsmidler men afholdende under aversiv medikamentel behandl		1	
DF1824;Afhængighedssyndrom ved brug af flygtige opløsningsmidler aktivt brugende		1	
DF1825;Afhængighedssyndrom ved brug af flygtige opløsningsmidler med kontinuerligt brug		1	
DF1826;Afhængighedssyndrom ved brug af flygtige opløsningsmidler med episodisk brug		1	
DF185;Psykotisk tilstand ved brug af flygtige opløsningsmidler		1	
DF1850;Skizofrenilignende psykotisk tilstand ved brug af flygtige opløsningsmidler		1	
DF1851;Paranoid psykotisk tilstand ved brug af flygtige opløsningsmidler		1	
DF1852;Hallucinatorisk psykotisk tilstand ved brug af flygtige opløsningsmidler		1	

Supplement 2 Nielsen et al. Mortality after paediatric emergency calls for patients with and without pre-existing comorbidity (2023)

DF1853;Polymorf psykotisk tilstand ved brug af flygtige opløsningsmidler		1	
DF1854;Psykotisk tilstand med depressive symptomer ved brug af flygtige opløsningsmidler		1	
DF1855;Psykotisk tilstand med maniske symptomer ved brug af flygtige opløsningsmidler		1	
DF1856;Blandet psykotisk tilstand ved brug af flygtige opløsningsmidler		1	
DF186;Amnestisk syndrom som følge af brug af flygtige opløsningsmidler		1	
DF187;Sen psykotisk eller residual tilstand forårsaget af brug af flygtige opløsningsmidler		1	
DF1870;Flashbacks som følge af brug af flygtige opløsningsmidler		1	
DF1871;Personligheds- eller adfærdsforstyrrelse forårsaget af brug af flygtige opløsningsmidler		1	
DF1872;Residual affektiv sindslidelse som følge af brug af flygtige opløsningsmidler		1	
DF1873;Demens som følge af brug af flygtige opløsningsmidler		1	
DF1874;Anden vedvarende kognitiv forstyrrelse som følge af brug af flygtige opløsningsmidler		1	
DF1875;Sent indsættende psykotisk tilstand som følge af brug af flygtige opløsningsmidler		1	
DF188;Anden psykisk lidelse eller adfærdsforstyrrelse forårsaget af brug af flygtige opløsningsmidler		1	
DF189;Psykisk lidelse eller adfærdsforstyrrelse forårsaget af brug af flygtige opløsningsmidler UNS		1	
DF19;Psykiske lidelser og adfærdsmæssige forstyrrelser forårsaget af brug af multiple eller andre psykoaktive stoffer		1	
DF190;Akut intoksikation med multiple eller andre psykoaktive stoffer	0		
DF1900;Akut ukompliceret intoksikation med flere eller andre psykoaktive stoffer	0		
DF1901;Akut intoksikation med flere eller andre psykoaktive stoffer med fysisk traume	0		
DF1902;Akut intoksikation med flere eller andre psykoaktive stoffer med andre somatiske komplik	0		
DF1903;Akut intoksikation med flere eller andre psykoaktive stoffer med delir	0		
DF1904;Akut intoksikation med flere eller andre psykoaktive stoffer med perceptionsforvrængning	0		
DF1905;Akut intoksikation med flere eller andre psykoaktive stoffer med koma	0		
DF1906;Akut intoksikation med flere eller andre psykoaktive stoffer med kramper	0		
DF191;Skadelig brug af flere eller andre psykoaktive stoffer		1	
DF192;Afhængighedssyndrom ved brug af flere eller andre psykoaktive stoffer		1	
DF1920;Afhængighedssyndrom ved brug af flere eller andre psykoaktive stoffer men afholdende		1	
DF1921;Afhængighedssyndrom ved brug af flere eller andre psykoaktive stoffer men afholdende i beskyttet miljø		1	
DF1922;Afhængighedssyndrom ved brug af flere eller andre psykoaktive stoffer i vedligeholdelses- eller substitutionsbehanc		1	
DF1923;Afhængighedssyndrom ved brug af flere eller andre psykoaktive stoffer men afholdende under aversiv medikament		1	
DF1924;Afhængighedssyndrom ved brug af flere eller andre psykoaktive stoffer aktivt brugende		1	
DF1925;Afhængighedssyndrom ved brug af flere eller andre psykoaktive stoffer med kontinuerligt brug		1	
DF1926;Afhængighedssyndrom ved brug af flere eller andre psykoaktive stoffer med episodisk brug		1	

Supplement 2 Nielsen et al. Mortality after paediatric emergency calls for patients with and without pre-existing comorbidity (2023)

DF193;Abstinenstilstand som følge af brug af flere eller andre psykoaktive stoffer		1	
DF1930;Abstinenstilstand som følge af brug af flere eller andre psykoaktive stoffer uden kramper		1	
DF1931;Abstinenstilstand forårsaget af brug af flere eller andre psykoaktive stoffer med kramper		1	
DF194;Delirøs abstinenstilstand som følge af brug af flere eller andre psykoaktive stoffer		1	
DF1940;Delirøs abstinenstilstand forårsaget af brug af flere eller andre psykoaktive stoffer uden kramper		1	
DF1941;Delirøs abstinenstilstand forårsaget af brug af flere eller andre psykoaktive stoffer med kramper		1	
DF195;Psykotisk tilstand ved brug af flere eller andre psykoaktive stoffer		1	
DF1950;Skizofrenilignende psykotisk tilstand ved brug af flere eller andre psykoaktive stoffer		1	
DF1951;Paranoid psykotisk tilstand ved brug af flere eller andre psykoaktive stoffer		1	
DF1952;Hallucinatorisk psykotisk tilstand ved brug af flere eller andre psykoaktive stoffer		1	
DF1953;Polymorf psykotisk tilstand ved brug af flere eller andre psykoaktive stoffer		1	
DF1954;Psykotisk tilstand med depressive symptomer ved brug af flere eller andre psykoaktive stoffer		1	
DF1955;Psykotisk tilstand med maniske symptomer ved brug af flere eller andre psykoaktive stoffer		1	
DF1956;Blandet psykotisk tilstand ved brug af flere eller andre psykoaktive stoffer		1	
DF196;Amnestisk syndrom som følge af brug af flere eller andre psykoaktive stoffer		1	
DF197;Sen psykotisk eller residual tilstand forårsaget af brug af flere eller andre psykoaktive stoffer		1	
DF1970;Flashbacks som følge af brug af flere eller andre psykoaktive stoffer		1	
DF1971;Personligheds- eller adfærdsforstyrrelser forårsaget af brug af flere eller andre psykoaktive stoffer		1	
DF1972;Residual affektiv sindslidelse som følge af brug af flere eller andre psykoaktive stoffer		1	
DF1973;Demens som følge af brug af flere eller andre psykoaktive stoffer		1	
DF1974;Anden vedvarende kognitiv forstyrrelse som følge af brug af flere eller andre psykoaktive stoffer		1	
DF1975;Sent indsættende psykotisk tilstand som følge af brug af flere eller andre psykoaktive stoffer		1	
DF198;Anden psykisk lidelse eller adfærdsforstyrrelse som følge af brug af flere eller andre psykoaktive stoffer		1	
DF199;Psykisk lidelse eller adfærdsforstyrrelse som følge af brug af flere eller andre psykoaktive stoffer UNS		1	
DF20;Skizofreni		1	
DF200;Paranoid skizofreni		1	
DF2000;Kontinuerlig paranoid skizofreni		1	
DF2001;Episodisk paranoid skizofreni med fremadskridende defekt		1	
DF2002;Episodisk paranoid skizofreni med stabil defekt		1	
DF2003;Episodisk remitterende paranoid skizofreni		1	
DF2004;Paranoid skizofreni med inkomplet remission		1	
DF2005;Paranoid skizofreni med komplet remission		1	

DF2008;Paranoid skizofreni med andet forløb		1	
DF2009;Paranoid skizofreni med ubestemt forløb		1	
DF201;Hebefren skizofreni		1	
DF2010;Kontinuerlig hebefren skizofreni		1	
DF2011;Episodisk hebefren skizofreni med fremadskridende defekt		1	
DF2012;Episodisk hebefren skizofreni med stabil defekt		1	
DF2013;Episodisk remitterende hebefren skizofreni		1	
DF2014;Hebefren skizofreni med inkomplet remission		1	
DF2015;Hebefren skizofreni med komplet remission		1	
DF2018;Hebefren skizofreni med andet forløb		1	
DF2019;Hebefren skizofreni med ubestemt forløb		1	
DF202;Kataton skizofreni		1	
DF2020;Kontinuerlig kataton skizofreni		1	
DF2021;Episodisk kataton skizofreni med fremadskridende defekt		1	
DF2022;Episodisk kataton skizofreni med stabil defekt		1	
DF2023;Episodisk remitterende kataton skizofreni		1	
DF2024;Kataton skizofreni med inkomplet remission		1	
DF2025;Kataton skizofreni med komplet remission		1	
DF2028;Kataton skizofreni med andet forløb		1	
DF2029;Kataton skizofreni med forløb ubestemt		1	
DF203;Udifferentieret skizofreni		1	
DF2030;Kontinuerlig udifferentieret skizofreni		1	
DF2031;Episodisk udifferentieret skizofreni med fremadskridende defekt		1	
DF2032;Episodisk udifferentieret skizofreni med stabil defekt		1	
DF2033;Episodisk remitterende udifferentieret skizofreni		1	
DF2034;Udifferentieret skizofreni med inkomplet remission		1	
DF2035;Udifferentieret skizofreni med komplet remission		1	
DF2038;Udifferentieret skizofreni med andet forløb		1	
DF2039;Udifferentieret skizofreni med ubestemt forløb		1	
DF204;Post-skizofren depression		1	
DF2040;Kontinuerlig post-skizofren depression		1	
DF2041;Episodisk post-skizofren depression med fremadskridende defekt		1	

DF2042;Episodisk post-skizofren depression med stabil defekt		1	
DF2043;Episodisk remitterende post-skizofren depression		1	
DF2044;Post-skizofren depression med inkomplet remission		1	
DF2045;Post-skizofren depression med komplet remission		1	
DF2048;Post-skizofren depression med andet forløb		1	
DF2049;Post-skizofren depression med ubestemt forløb		1	
DF205;Skizofren residual-tilstand		1	
DF2050;Kontinuerlig skizofren residualtilstand		1	
DF2051;Episodisk skizofren residualtilstand med fremadskridende defekt		1	
DF2052;Episodisk skizofren residualtilstand med stabil defekt		1	
DF2053;Episodisk remitterende skizofren residualtilstand		1	
DF2054;Skizofren residualtilstand med inkomplet remission		1	
DF2055;Skizofren residualtilstand med komplet remission		1	
DF2058;Skizofren residualtilstand med andet forløb		1	
DF2059;Skizofren residualtilstand med ubestemt forløb		1	
DF206;Simpel skizofreni		1	
DF2060;Kontinuerlig simpel skizofreni		1	
DF2061;Episodisk simpel skizofreni med fremadskridende defekt		1	
DF2062;Episodisk simpel skizofreni med stabil defekt		1	
DF2063;Episodisk remitterende simpel skizofreni		1	
DF2064;Simpel skizofreni med inkomplet remission		1	
DF2065;Simpel skizofreni med komplet remission		1	
DF2068;Simpel skizofreni med andet forløb		1	
DF2069;Simpel skizofreni med ubestemt forløb		1	
DF208;Skizofreni af anden type		1	
DF2080;Kontinuerlig skizofreni af anden type		1	
DF2081;Episodisk skizofreni af anden type med fremadskridende defekt		1	
DF2082;Episodisk skizofreni af anden type med stabil defekt		1	
DF2083;Episodisk remitterende skizofreni af anden type		1	
DF2084;Skizofreni af anden type med inkomplet remission		1	
DF2085;Skizofreni af anden type med komplet remission		1	
DF2088;Skizofreni af anden type med andet forløb		1	

DF2089;Skizofreni af anden type med ubestemt forløb		1		
DF209;Skizofreni UNS		1		
DF2090;Kontinuerlig skizofreni UNS		1		
DF2091;Episodisk skizofreni UNS med fremadskridende defekt		1		
DF2092;Episodisk skizofreni UNS med stabil defekt		1		
DF2093;Episodisk remitterende skizofreni UNS		1		
DF2094;Skizofreni UNS med inkomplet remission		1		
DF2095;Skizofreni UNS med komplet remission		1		
DF2098;Skizofreni UNS med andet forløb		1		
DF2099;Skizofreni UNS med ubestemt forløb		1		
DF21;Skizotypisk sindslidelse		1		
DF219;Skizotypisk sindslidelse UNS		1		
DF22;Paranoide psykoser		1		
DF220;Enkel paranoia		1		
DF228;Anden paranoid psykose		1		
DF229;Paranoid psykose UNS		1		
DF23;Akutte og forbigående psykoser		1		
DF230;Akut polymorf non-skizofreniform psykose		1		
DF2300;Akut polymorf non-skizofreniform non-reaktiv psykose		1		
DF2301;Akut polymorf non-skizofreniform reaktiv psykose		1		
DF231;Akut polymorf skizofreniform psykose		1		
DF2310;Akut polymorf skizofreniform non-reaktiv psykose		1		
DF2311;Akut polymorf skizofreniform reaktiv psykose		1		
DF232;Akut skizofreniform psykose		1		
DF2320;Akut skizofreniform non-reaktiv psykose		1		
DF2321;Akut skizofreniform reaktiv psykose		1		
DF233;Akut paranoid psykose		1		
DF2330;Akut paranoid non-reaktiv psykose		1		
DF2331;Akut paranoid reaktiv psykose		1		
DF238;Anden akut eller forbigående psykose		1		
DF2380;Anden non-reaktiv psykose		1		
DF2381;Anden reaktiv psykose		1		

Supplement 2 Nielsen et al. Mortality after paediatric emergency calls for patients with and without pre-existing comorbidity (2023)

DF239;Akut eller forbigående psykose UNS		1		
DF2390;Akut eller forbigående non-reaktiv psykose UNS		1		
DF2391;Akut eller forbigående reaktiv psykose UNS		1		
DF24;Induceret psykose		1		
DF249;Induceret paranoid psykose		1		
DF25;Skizoaffektive psykoser		1		
DF250;Skizoaffektiv psykose af manisk type		1		
DF2500;Skizoaffektiv psykose af manisk type med maniske og skizofrene symptomer fuldt ud samtidige		1		
DF2501;Skizoaffektiv psykose af manisk type med skizofrene symptomer ud over varigheden af de maniske symptomer		1		
DF251;Skizoaffektiv psykose af depressiv type		1		
DF2510;Skizoaffektiv psykose af depressiv type med depressive og skizofrene symptomer fuldt ud samtidige		1		
DF2511;Skizoaffektiv psykose af depressiv type med skizofrene symptomer ud over varigheden af de depressive symptome		1		
DF252;Skizoaffektiv psykose af blandet manisk-depressiv type		1		
DF2520;Skizoaffektiv psykose af blandet manisk-depressiv type med affektive og skizofrene symptomer fuldt ud samtidige		1		
DF2521;Skizoaffektiv psykose af blandet manisk-depressiv type med skizofrene symptomer ud over varigheden af de affekt		1		
DF258;Skizoaffektiv psykose af anden type		1		
DF2580;Skizoaffektiv psykose af anden type med affektive og skizofrene symptomer fuldt ud samtidige		1		
DF2581;Skizoaffektiv psykose af anden type med skizofrene symptomer ud over varigheden af de affektive symptomer		1		
DF259;Skizoaffektiv psykose UNS		1		
DF2590;Skizoaffektiv psykose UNS med affektive og skizofrene symptomer fuldt ud samtidige		1		
DF2591;Skizoaffektiv psykose UNS med skizofrene symptomer ud over varigheden af de affektive symptomer		1		
DF28;Andre ikke-organiske psykoser		1		
DF289;Anden ikke-organisk psykose UNS		1		
DF29;Ikke specificeret ikke-organisk psykose		1		
DF299;Ikke-organisk psykose UNS		1		
DF30;Manisk enkeltepisode		1		
DF300;Hypoman enkeltepisode		1		
DF301;Manisk enkeltepisode uden psykotiske symptomer		1		
DF302;Manisk enkeltepisode med psykotiske symptomer		1		
DF3020;Manisk enkeltepisode med stemningskongruente psykotiske symptomer		1		
DF3021;Manisk enkeltepisode med stemningsinkongruente psykotiske symptomer		1		
DF308;Manisk enkeltepisode af anden type		1		

DF309;Manisk enkeltepisode UNS		1	
DF309A;Mani UNS		1	
DF31;Bipolar affektiv sindslidelse		1	
DF310;Bipolar affektiv sindslidelse i hypoman episode		1	
DF311;Bipolær affektiv sindslidelse i manisk episode uden psykotiske symptomer		1	
DF312;Bipolar affektiv sindslidelse i manisk episode med psykotiske symptomer		1	
DF3120;Bipolær affektiv sindslidelse i manisk episode med stemningskongruente psykotiske symptomer		1	
DF3121;Bipolær affektiv sindslidelse i manisk episode med stemningsinkongruente psykotiske symptomer		1	
DF313;Bipolar affektiv sindslidelse i lettere eller moderat depressiv episode		1	
DF314;Bipolær affektiv sindslidelse i svær depressiv episode uden psykotiske symptomer		1	
DF315;Bipolær affektiv sindslidelse i svær depressiv episode med psykotiske symptomer		1	
DF3150;Bipolær affektiv sindslidelse i svær depressiv episode med stemningskongruente psykotiske symptomer		1	
DF3151;Bipolær affektiv sindslidelse i svær depressiv episode med stemningsinkongruente psykotiske symptomer		1	
DF316;Bipolar affektiv sindslidelse i episode med blandingstilstand		1	
DF317;Bipolar affektiv sindslidelse i remission		1	
DF318;Anden form for bipolar affektiv sindslidelse		1	
DF319;Bipolar affektiv sindslidelse UNS		1	
DF32;Depressiv enkeltepisode		1	
DF320;Depressiv enkeltepisode af lettere grad		1	
DF3200;Depressiv non-melankoliform enkeltepisode af lettere grad		1	
DF3201;Depressiv melankoliform enkeltepisode af lettere grad		1	
DF321;Depressiv enkeltepisode af moderat grad		1	
DF3210;Depressiv non-melankoliform enkeltepisode af moderat grad		1	
DF3211;Depressiv melankoliform enkeltepisode af moderat grad		1	
DF322;Depressiv enkeltepisode af svær grad uden psykotiske symptomer		1	
DF323;Depressiv enkeltepisode af svær grad med psykotiske symptomer		1	
DF3230;Depressiv enkeltepisode af svær grad med stemningskongruente psykotiske symptomer		1	
DF3231;Depressiv enkeltepisode af svær grad med stemningsinkongruente psykotiske symptomer		1	
DF328;Depressiv enkeltepisode af anden type		1	
DF329;Depressiv enkeltepisode UNS		1	
DF33;Periodisk depression		1	
DF330;Periodisk depression i episode af lettere grad		1	

DF3300;Periodisk non-melankoliform depression i episode af lettere grad		1	
DF3301;Periodisk melankoliform depression i episode af lettere grad		1	
DF331;Periodisk depression i episode af moderat grad		1	
DF3310;Periodisk non-melankoliform depression i episode af moderat grad		1	
DF3311;Periodisk melankoliform depression i episode af moderat grad		1	
DF332;Periodisk depression i episode af svær grad uden psykotiske symptomer		1	
DF333;Periodisk depression i episode af svær grad med psykotiske symptomer		1	
DF3330;Periodisk depression i episode af svær grad med stemningskongruente psykotiske symptomer		1	
DF3331;Periodisk depression i episode af svær grad med stemningsinkongruente psykotiske symptomer		1	
DF334;Periodisk depression i remission		1	
DF338;Periodisk depression af anden type		1	
DF339;Periodisk depression UNS		1	
DF34;Vedvarende affektive tilstande		1	
DF340;Cyklotymi		1	
DF3400;Cyklotym personlighedsstruktur		1	
DF3401;Cyklotym tilstand		1	
DF341;Dystymi		1	
DF3410;Depressiv personlighedsstruktur		1	
DF3411;Kronisk lettere depressionstilstand		1	
DF348;Kronisk forstemningstilstand af anden type		1	
DF349;Kronisk forstemningstilstand UNS		1	
DF38;Andre affektive sindslidelser eller tilstande		1	
DF380;Anden affektiv sindslidelse, enkeltepisode		1	
DF3800;Anden affektiv sindslidelse, enkeltepisode med blandingstilstand		1	
DF381;Anden periodisk affektiv sindslidelse		1	
DF3810;Affektiv sindslidelse med tilbagevendende, kortvarige depressive episoder		1	
DF388;Anden affektiv sindslidelse eller tilstand		1	
DF39;Ikke specificeret affektiv sindslidelse eller tilstand		1	
DF399;Affektiv sindslidelse UNS		1	
DF40;Fobiske angsttilstande		1	
DF400;Agorafobi		1	
DF4000;Agorafobi uden panikangst		1	

Supplement 2 Nielsen et al. Mortality after paediatric emergency calls for patients with and without pre-existing comorbidity (2023)

DF4001;Agorafobi med panikangst		1	
DF401;Socialfobi		1	
DF402;Enkelfobi		1	
DF402A;Parasitofobi		1	
DF408;Fobisk angst af anden type		1	
DF408A;Venerofobi		1	
DF409;Fobisk angst UNS		1	
DF41;Andre angsttilstande		1	
DF410;Panikangst		1	
DF4100;Moderat panikangst		1	
DF4101;Svær panikangst		1	
DF411;Generaliseret angst		1	
DF412;Lettere angst-depressionstilstand		1	
DF413;Anden blandet angsttilstand		1	
DF418;Anden angsttilstand		1	
DF418A;Fødselsangst		1	
DF418B;Angsthysteri		1	
DF419;Angsttilstand UNS		1	
DF42;Obsessiv-kompulsiv tilstand		1	
DF420;Overvejende obsessiv tilstand		1	
DF421;Overvejende kompulsiv tilstand		1	
DF422;Blandet obsessiv-kompulsiv tilstand		1	
DF428;Anden obsessiv-kompulsiv tilstand		1	
DF429;Obsessiv-kompulsiv tilstand UNS		1	
DF43;Reaktioner på svær belastning og tilpasningsreaktioner		1	
DF430;Akut belastningsreaktion		1	
DF4300;Let akut belastningsreaktion		1	
DF4301;Moderat akut belastningsreaktion		1	
DF4302;Svær akut belastningsreaktion		1	
DF431;Posttraumatisk belastningsreaktion		1	
DF432;Tilpasningsreaktion		1	
DF4320;Kortvarig depressiv tilpasningsreaktion		1	

Supplement 2 Nielsen et al. Mortality after paediatric emergency calls for patients with and without pre-existing comorbidity (2023)

DF4321;Langvarig depressiv tilpasningsreaktion		1	
DF4322;Blandet angst-depressiv tilpasningsreaktion		1	
DF4323;Tilpasningsreaktion med andre emotionelle symptomer		1	
DF4324;Tilpasningsreaktion med overvejende adfærdsforstyrrelse		1	
DF4325;Tilpasningsreaktion med adfærdssymptomer og emotionelle symptomer		1	
DF4328;Anden tilpasningsreaktion		1	
DF438;Anden belastningsreaktion		1	
DF439;Belastningsreaktion UNS		1	
DF44;Dissociative tilstande og forstyrrelser		1	
DF440;Dissociativ amnesi		1	
DF441;Dissociativ fugue		1	
DF442;Dissociativ stupor		1	
DF443;Dissociativ trance- eller besættelsestilstand		1	
DF444;Dissociativ bevægelsesforstyrrelse		1	
DF445;Dissociative kramper		1	
DF446;Dissociativ sanseforstyrrelse		1	
DF446A;Psykogent høretab		1	
DF447;Blandet dissociativ tilstand		1	
DF448;Anden dissociativ tilstand eller forstyrrelse		1	
DF4480;Gansers syndrom		1	
DF4481;Multipel personlighed		1	
DF4482;Forbigående dissociativ tilstand i barndom eller adolescens		1	
DF4488;Anden dissociativ tilstand		1	
DF4488A;Søvnrelateret dissociativ sygdom		1	
DF449;Dissociativ tilstand eller forstyrrelse UNS		1	
DF45;Somatoforme tilstande		1	
DF450;Somatiseringstilstand		1	
DF451;Udifferentieret somatoform tilstand		1	
DF452;Helbredsangst		1	
DF452A;Dysmorfofobi		1	
DF453;Somatoform autonom dysfunktion		1	
DF4530;Somatoform autonom dysfunktion i det kardiovaskulære system		1	

Supplement 2 Nielsen et al. Mortality after paediatric emergency calls for patients with and without pre-existing comorbidity (2023)

DF4531;Somatoform autonom dysfunktion i det øvre gastrointestinale system		1	
DF4532;Somatoform autonom dysfunktion i det nedre gastrointestinale system		1	
DF4533;Somatoform autonom dysfunktion i det respiratoriske system		1	
DF4534;Somatoform autonom dysfunktion i det urogenitale system		1	
DF4538;Somatoform autonom dysfunktion i andet organ eller system		1	
DF454;Vedvarende somatoform smertetilstand		1	
DF458;Anden somatoform tilstand		1	
DF459;Somatoform tilstand UNS		1	
DF48;Andre nervøse tilstande		1	
DF480;Neurasteni		1	
DF481;Depersonalisations-derealisations syndrom		1	
DF488;Anden nervøs tilstand		1	
DF489;Nervøs tilstand UNS		1	
DF50;Spiseforstyrrelser		1	
DF500;Nervøs spisevægring		1	
DF501;Atypisk nervøs spisevægring		1	
DF502;Nervøs spiseanfaldstilbøjelighed		1	
DF503;Atypisk nervøs spiseanfaldstilbøjelighed		1	
DF504;Trøstespisning		1	
DF505;Opkastning forbundet med anden psykisk forstyrrelse		1	
DF508;Anden spiseforstyrrelse		1	
DF508A;Tvangsoverspisning (BED)		1	
DF509;Spiseforstyrrelse UNS		1	
DF51;Ikke-organiske søvnforstyrrelser	0		
DF510;Ikke-organisk søvnløshed	0		
DF5100;Søvnløshed UNS	0		
DF5101;Akut søvnløshed	0		
DF5102;Paradoks søvnløshed	0		
DF5103;Psykofysiologisk søvnløshed	0		
DF5104;Idiopatisk søvnløshed	0		
DF5105;Søvnløshed betinget af mental sygdom	0		
DF511;Ikke-organisk øget søvntrang	0		

Supplement 2 Nielsen et al. Mortality after paediatric emergency calls for patients with and without pre-existing comorbidity (2023)

DF5110;Hypersomni uden kendt årsag	0		
DF5111;Adfærdsbetinget utilstrækkelig søvn	0		
DF512;Ikke-organisk søvnrytmeforstyrrelse	0		
DF5120;Forstyrret søvnrytme UNS	0		
DF5121;Forstyrret søvnrytme på grund af tidszoneskift	0		
DF5122;Forstyrret søvnrytme på grund af skifteholdsarbejde	0		
DF5123;Forstyrret søvnrytme på grund af forsinket søvntid	0		
DF513;Søvngængeri	0		
DF514;Søvnrædsel	0		
DF515;Natlige mareridt	0		
DF518;Anden form for ikke-organisk søvnforstyrrelse	0		
DF518A;Eksternt betinget søvnforstyrrelse UNS	0		
DF519;Ikke-organisk søvnforstyrrelse UNS	0		
DF52;Ikke-organiske seksuelle funktionsforstyrrelser	0		
DF520;Nedsat eller manglende seksuallyst	0		
DF521;Seksuel aversion eller manglende seksuel lystfølelse	0		
DF5210;Seksuel aversion	0		
DF5211;Manglende seksuel lystfølelse	0		
DF522;Ikke-organisk genital dysfunktion	0		
DF522A;Psykogen impotens	0		
DF523;Hæmmet orgasme	0		
DF524;Præmatur ejakulation	0		
DF525;Ikke-organisk vaginisme	0		
DF526;Ikke-organisk dyspareuni	0		
DF527;Øget seksualdrift	0		
DF528;Anden ikke-organisk seksuel dysfunktion	0		
DF529;Ikke-organisk seksuel dysfunktion UNS	0		
DF53;Psykiske lidelser i barselsperioden IKA		1	
DF530;Lettere psykisk lidelse i barselsperioden IKA		1	
DF530A;Barselsdepression		1	
DF531;Svær psykisk lidelse i barselsperioden IKA		1	
DF538;Anden psykisk lidelse i barselsperioden IKA		1	

Supplement 2 Nielsen et al. Mortality after paediatric emergency calls for patients with and without pre-existing comorbidity (2023)

DF539;Psykisk lidelse i barselsperioden UNS		1	
DF54;Psykiske faktorer forbundet med sygdomme klassificeret andetsteds		1	
DF549;Psykiske faktorer forbundet med sygdom klassificeret andetsteds		1	
DF55;Misbrug af ikke-afhængighedsskabende stoffer		1	
DF550;Misbrug af antidepressiva		1	
DF551;Misbrug af laksantia		1	
DF552;Misbrug af ikke-psykoaktive analgetika		1	
DF553;Misbrug af antacida		1	
DF554;Misbrug af vitaminer		1	
DF555;Misbrug af steroider eller hormoner		1	
DF556;Misbrug af naturmedicin		1	
DF558;Misbrug af andre ikke-afhængighedsskabende stoffer		1	
DF559;Misbrug af ikke-afhængighedsskabende stoffer UNS		1	
DF59;Adfærdsændringer forbundet med fysiologiske og fysiske faktorer uden nærmere specificering		1	
DF599;Adfærdsændring UNS forbundet med fysiologisk eller fysisk faktor		1	
DF60;Specifikke forstyrrelser af personlighedsstrukturen		1	
DF600;Paranoid personlighedsstruktur		1	
DF601;Skizoid personlighedsstruktur		1	
DF602;Dyssocial personlighedsstruktur		1	
DF603;Emotionelt ustabil personlighedsstruktur		1	
DF6030;Emotionelt ustabil personlighedsstruktur af impulsiv type		1	
DF6031;Emotionelt ustabil personlighedsstruktur af borderline type		1	
DF604;Histrionisk personlighedsstruktur		1	
DF605;Tvangspræget personlighedsstruktur		1	
DF606;Ængstelig personlighedsstruktur		1	
DF607;Dependent personlighedsstruktur		1	
DF608;Anden forstyrrelse af personlighedsstrukturen		1	
DF609;Forstyrrelse i personlighedsstrukturen UNS		1	
DF61;Forstyrrelser i personlighedsstruktur af blandet og anden type		1	
DF610;Forstyrrelse i personlighedsstruktur af blandet type		1	
DF611;Personlighedsændring ved primær affektiv lidelse eller angsttilstand		1	
DF62;Ikke-organiske personlighedsændringer		1	

DF620;Personlighedsændring efter katastrofeoplevelse		1	
DF621;Personlighedsændring efter psykisk sygdom		1	
DF628;Anden personlighedsændring		1	
DF629;Personlighedsændring UNS		1	
DF63;Patologiske vane- og impulshandlinger		1	
DF630;Patologisk spillelidenskab		1	
DF631;Pyromani		1	
DF632;Kleptomani		1	
DF633;Trikotillomani		1	
DF638;Anden patologisk vane- og impulshandling		1	
DF639;Patologisk vane- og impulshandling UNS		1	
DF65;Seksuelle afvigelser		1	
DF650;Fetichisme		1	
DF652;Exhibitionisme		1	
DF653;Voyeurisme		1	
DF640;Transseksualisme (findes ikke i SKS)	0		
DF649;Kønsidentitetsforstyrrelse UNS (findes ikke i SKS)	0		
DF654;Pædofili		1	
DF656;Blandet seksuel afvigelser		1	
DF658;Anden seksuel afvigelse		1	
DF659;Seksuel afvigelse UNS		1	
DF66;Seksuelle udviklings- og orienteringsforstyrrelser		1	
DF660;Seksuel modningsforstyrrelse		1	
DF661;Egodyston seksualorientering		1	
DF662;Seksuel forholdsforstyrrelse		1	
DF668;Anden psykoseksuel udviklingsforstyrrelse		1	
DF669;Psykoseksuel udviklingsforstyrrelse UNS		1	
DF68;Andre forstyrrelser i personlighedsstruktur og adfærd hos voksne		1	
DF680;Psykisk udbygning af somatiske symptomer		1	
DF681;Sygdomsefterligning helt eller delvist bevidst		1	
DF681A;Secretans syndrom		1	
DF681B;Patomimi		1	

Supplement 2 Nielsen et al. Mortality after paediatric emergency calls for patients with and without pre-existing comorbidity (2023)

DF681C;Selvmutilation UNS		1	
DF681D;Münchhausens syndrom		1	
DF688;Anden forstyrrelse i personlighed, struktur og adfærd hos voksen		1	
DF69;Forstyrrelser i personlighedsstruktur og adfærd uden nærmere specificering		1	
DF699;Forstyrrelse i personlighedsstruktur og adfærd UNS		1	
DF70;Mental retardering af lettere grad		1	
DF700;Lettere mental retardering med ringe påvirkning af adfærd		1	
DF701;Lettere mental retardering med væsentlig påvirkning af adfærd		1	
DF708;Lettere mental retardering med anden påvirkning af adfærd		1	
DF709;Lettere mental retardering med påvirkning af adfærd UNS		1	
DF71;Mental retardering af middelsvær grad		1	
DF710;Middelsvær mental retardering med ringe påvirkning af adfærd		1	
DF711;Middelsvær mental retardering med væsentlig påvirkning af adfærd		1	
DF718;Middelsvær mental retardering med anden påvirkning af adfærd		1	
DF719;Middelsvær mental retardering med påvirkning af adfærd UNS		1	
DF72;Mental retardering af sværere grad			2
DF720;Sværere mental retardering med ringe påvirkning af adfærd			2
DF721;Sværere mental retardering med væsentlig påvirkning af adfærd			2
DF728;Sværere mental retardering med anden påvirkning af adfærd			2
DF729;Sværere mental retardering med påvirkning af adfærd UNS			2
DF73;Mental retardering i sværeste grad			2
DF730;Mental retardering i sværeste grad med ringe påvirkning af adfærd			2
DF731;Mental retardering i sværeste grad med væsentlig påvirkning af adfærd			2
DF738;Mental retardering i sværeste grad med med anden påvirkning af adfærd			2
DF739;Mental retardering i sværeste grad med påvirkning af adfærd UNS			2
DF78;Anden mental retardering		1	
DF780;Anden mental retardering med ringe påvirkning af adfærd		1	
DF781;Anden mental retardering med væsentlig påvirkning af adfærd		1	
DF788;Anden mental retardering med anden påvirkning af adfærd		1	
DF789;Anden mental retardering med påvirkning af adfærd UNS		1	
DF79;Mental retardering uden nærmere specificering		1	
DF790;Mental retardering UNS med ringe påvirkning af adfærd		1	

DF791;Mental retardering UNS med væsentlig påvirkning af adfærd		1	
DF798;Mental retardering UNS med anden påvirkning af adfærd		1	
DF799;Mental retardering UNS		1	
DF80;Specifikke udviklingsforstyrrelser af tale og sprog		1	
DF800;Specifik sprogartikulationsforstyrrelse		1	
DF801;Ekspressiv dysfasi		1	
DF802;Impressiv dysfasi		1	
DF803;Epileptisk afasi		1	
DF808;Anden udviklingsforstyrrelse af tale eller sprog		1	
DF809;Udviklingsforstyrrelse af tale og sprog UNS		1	
DF809A;Verbal dyspraksi		1	
DF81;Specifikke udviklingsforstyrrelser af skolefærdigheder	0		
DF810;Specifik læseforstyrrelse	0		
DF811;Specifik staveforstyrrelse	0		
DF812;Specifik regneforstyrrelse	0		
DF813;Blandet udviklingsforstyrrelse af skolefærdigheder	0		
DF818;Anden udviklingsforstyrrelse af skolefærdigheder	0		
DF819;Udviklingsforstyrrelse af skolefærdigheder UNS	0		
DF82;Specifikke udviklingsforstyrrelser af motoriske færdigheder		1	
DF829;Specifik udviklingsforstyrrelse af motoriske færdigheder		1	
DF829A;Oral dyspraksi		1	
DF83;Blandede udviklingsforstyrrelser af specifikke færdigheder		1	
DF839;Blandet udviklingsforstyrrelse af specifikke færdigheder		1	
DF84;Gennemgribende mentale udviklingsforstyrrelser		1	
DF840;Infantil autisme		1	
DF841;Atypisk autisme		1	
DF8410;Autisme atypisk med hensyn til begyndelsesalder		1	
DF8411;Autisme atypisk med hensyn til symptomatologi		1	
DF8412;Autisme atypisk med hensyn til både begyndelsesalder og symptomatologi		1	
DF842;Retts syndrom		1	
DF843;Anden disintegrativ forstyrrelse i barndommen		1	
DF844;Hyperaktivitetsforstyrrelse med mental retardering og bevægelsesstereotypier		1	

Supplement 2 Nielsen et al. Mortality after paediatric emergency calls for patients with and without pre-existing comorbidity (2023)

DF845;Aspergers syndrom		1	
DF848;Anden gennemgribende mental udviklingsforstyrrelse		1	
DF849;Gennemgribende mental udviklingsforstyrrelse UNS		1	
DF88;Andre psykiske udviklingsforstyrrelser		1	
DF889;Psykisk udviklingsforstyrrelse IKA		1	
DF89;Psykiske udviklingsforstyrrelser uden nærmere specificering		1	
DF899;Psykisk udviklingsforstyrrelse UNS		1	
DF90;Hyperkinetiske forstyrrelser		1	
DF900;Forstyrrelse af aktivitet og opmærksomhed		1	
DF901;Hyperkinetisk adfærdsforstyrrelse		1	
DF908;Anden hyperkinetisk forstyrrelse		1	
DF909;Hyperkinetisk forstyrrelse UNS		1	
DF91;Adfærdsforstyrrelser		1	
DF910;Adfærdsforstyrrelse begrænset til familien		1	
DF911;Usocialiseret adfærdsforstyrrelse		1	
DF912;Socialiseret adfærdsforstyrrelse		1	
DF913;Oppositionel adfærdsforstyrrelse		1	
DF918;Anden adfærdsforstyrrelse		1	
DF919;Adfærdsforstyrrelse UNS		1	
DF92;Blandede adfærds- og følelsesmæssige forstyrrelser		1	
DF920;Depressiv adfærdsforstyrrelse		1	
DF928;Andre blandede adfærds- og følelsesmæssig forstyrrelser		1	
DF929;Blandet adfærds- og følelsesmæssig forstyrrelse UNS		1	
DF93;Emotionelle forstyrrelser opstået i barndommen		1	
DF930;Abnorm separationsangst i barndom		1	
DF931;Fobisk angsttilstand i barndom		1	
DF932;Socialangsttilstand i barndom		1	
DF933;Overdreven søskendejalousi		1	
DF938;Anden emotionel forstyrrelse i barndom		1	
DF9380;Generel angsttilstand i barndommen		1	
DF939;Emotionel forstyrrelse i barndom UNS		1	
DF94;Sociale funktionsforstyrrelser i barndom og adolescens		1	

DF940;Elektiv mutisme		1	
DF941;Reaktiv tilknytningsforstyrrelse i barndom		1	
DF942;Uselektiv, uhæmmet tilknytnings- eller kontaktform		1	
DF948;Anden social funktionsforstyrrelse		1	
DF949;Social funktionsforstyrrelse UNS		1	
DF95;Tics		1	
DF950;Forbigående tic		1	
DF951;Kroniske motoriske eller vokale tics		1	
DF952;Kombinerede vokale og multiple motoriske tics		1	
DF958;Anden tic-lidelse		1	
DF959;Tics UNS		1	
DF98;Andre adfærdsmæssige og emotionelle forstyrrelser opstået i barndom eller adolescens		1	
DF980;Ikke-organisk enuresis		1	
DF9800;Ikke-organisk enuresis alene om natten		1	
DF9801;Ikke-organisk enuresis alene om dagen		1	
DF9802;Ikke-organisk enuresis både nat og dag		1	
DF981;Ikke-organisk encoprese		1	
DF9810;Ikke-organisk encoprese med manglende sfinkterkontrol		1	
DF9811;Ikke-organisk encoprese med normal sfinkterkontrol og fæces		1	
DF9812;Ikke-organisk encoprese med flydende fæces		1	
DF982;Spiseforstyrrelse i barndommen		1	
DF983;Pica i barndommen		1	
DF984;Bevægelsesstereotypier		1	
DF9840;Ikke-selvskadende bevægelsesstereotypier		1	
DF9841;Selvskadende bevægelsesstereotypier		1	
DF9842;Blandede bevægelsesstereotypier		1	
DF985;Stammen		1	
DF986;Støden		1	
DF988;Anden adfærdsmæssig og emotionel forstyrrelse opstået i barndom eller adolescens		1	
DF988A;Onychophagia		1	
DF988B;Onychotillomania		1	
DF988C;Opmærksomhedsforstyrrelse uden hyperaktivitet		1	

Supplement 2 Nielsen et al. Mortality after paediatric emergency calls for patients with and without pre-existing comorbidity (2023)

DF989;Adfærdsmæssig eller emotionel forstyrrelse i barndom eller adolescens UNS		1	
DF99;Psykiske lidelser eller forstyrrelser ikke nærmere specificeret		1	
DF999;Psykisk lidelse eller forstyrrelse UNS		1	
DG00;Meningitis forårsaget af bakterier IKA		1	
DG000;Meningitis forårsaget af Haemophilus influenzae		1	
DG001;Meningitis forårsaget af pneumokokker		1	
DG002;Meningitis forårsaget af andre streptokokker		1	
DG003;Meningitis forårsaget af stafylokokker		1	
DG008;Anden bakteriel meningitis		1	
DG008A;Meningitis forårsaget af Klebsiella		1	
DG008B;Meningitis forårsaget af Escherichia coli		1	
DG009;Bakteriel meningitis UNS		1	
DG009A;Meningitis purulenta UNS		1	
DG01;Meningitis ved bakterielle sygdomme klassificeret andetsteds		1	
DG019;Meningitis ved bakteriesygdom klassificeret andetsteds		1	
DG02;Meningitis ved andre infektiøse og parasitære sygdomme klassificeret andetsteds		1	
DG020;Meningitis ved viral sygdom klassificeret andetsteds		1	
DG021;Meningitis ved mykose klassificeret andetsteds		1	
DG028;Meningitis ved anden infektiøs eller parasitær sygdom klassificeret andetsteds		1	
DG03;Hjernehindebetændelse af andre og ikke specificerede årsager		1	
DG030;Ikke-infektiøs meningitis		1	
DG031;Kronisk meningitis		1	
DG032;Godartet recidiverende meningitis		1	
DG038;Meningitis af anden årsag		1	
DG039;Meningitis UNS		1	
DG04;Hjerne- og rygmarvsbetændelse		1	
DG040;Akut dissemineret encephalitis		1	
DG040A;Encephalitis eller encephalomyelitis efter vaccination		1	
DG041;Human T-celle lymphotrop virus associeret myelopati		1	
DG042;Bakteriel meningoencephalitis eller meningomyelitis IKA		1	
DG042A;Bakteriel meningomyelitis IKA		1	
DG042B;Bakteriel meningoencephalitis IKA		1	

DG048;Anden encephalitis, myelitis eller encephalomyelitis		1	
DG048A;Postinfektiøs encephalitis IKA		1	
DG048B;Postinfektiøs encephalomyelitis IKA		1	
DG048C;Postinfektiøs myelitis IKA		1	
DG049;Encephalitis, myelitis eller encephalomyelitis UNS		1	
DG049A;Encephalitis UNS		1	
DG049B;Encephalomyelitis UNS		1	
DG049C;Myelitis UNS		1	
DG049D;Cerebral ventrikulitis UNS		1	
DG05;Hjerne- og rygmarvsbetændelse ved sygdomme klassificeret andetsteds		1	
DG050;Encephalitis, myelitis og encephalomyelitis ved bakterielle sygdomme klassificeret andetsteds		1	
DG050P;Encephalitis ved bakteriel sygdom klassificeret andetsteds		1	
DG050R;Encephalomyelitis ved bakteriel sygdom klassificeret andetsteds		1	
DG050S;Myelitis ved bakteriel sygdom klassificeret andetsteds		1	
DG051;Encephalitis, myelitis og encephalomyelitis ved viral sygdom klassificeret andetsteds		1	
DG051U;Encephalitis ved viral sygdom klassificeret andetsteds		1	
DG051V;Encephalomyelitis ved viral sygdom klassificeret andetsteds		1	
DG051X;Myelitis ved viral sygdom klassificeret andetsteds		1	
DG052;Encephalitis, myelitis og encephalomyelitis ved anden infektiøs eller parasitær sygdom klassificeret andetsteds		1	
DG052K;Encephalitis ved anden infektiøs eller parasitær sygdom klassificeret andetsteds		1	
DG052L;Myelitis ved anden infektiøs eller parasitær sygdom klassificeret andetsteds		1	
DG052M;Encephalomyelitis ved anden infektiøs eller parasitær sygdom klassificeret andetsteds		1	
DG052N;Meningoencephalitis ved anden infektiøs eller parasitær sygdom klassificeret andetsteds		1	
DG058;Encephalitis, myelitis og encephalomyelitis ved anden sygdom klassificeret andetsteds		1	
DG06;Intrakraniel og intraspinal absces og granulom		1	
DG060;Intrakraniel absces eller granulom		1	
DG060A;Intrakraniel epidural absces		1	
DG060B;Intrakraniel subdural absces		1	
DG060C;Intrakraniel otogen absces		1	
DG060E;Absces i lillehjernen		1	
DG060F;Absces i storhjernen		1	
DG060G;Granulom i lillehjernen		1	

Supplement 2 Nielsen et al. Mortality after paediatric emergency calls for patients with and without pre-existing comorbidity (2023)

DG060H;Intrakranielt subduralt granulom		1		
DG060I;Intrakranielt otogent granulom		1		
DG060J;Granulom i storhjernen		1		
DG060K;Intrakranielt granulom UNS		1		
DG060L;Intrakranielt epiduralt granulom		1		
DG061;Intraspinal absces eller granulom		1		
DG061A;Intraspinal subdural absces		1		
DG061C;Intraspinal epidural absces		1		
DG061E;Intraspinalt epiduralt granulom		1		
DG061F;Intraspinalt granulom UNS		1		
DG061G;Intraspinalt subduralt granulom		1		
DG062;Epidural eller subdural absces UNS		1		
DG062A;Epidural absces UNS		1		
DG062C;Subdural absces UNS		1		
DG07;Intrakranielle og intraspinale abscesser og granulomer ved sygdomme klassificeret andetsteds		1		
DG079;Intrakraniel eller intraspinal absces eller granulom ved sygdom klassificeret andetsteds		1		
DG079A;Intraspinal absces ved sygdom klassificeret andetsteds		1		
DG079B;Intrakraniel absces ved sygdom klassificeret andetsteds		1		
DG079J;Intrakranielt granulom ved sygdom klassificeret andetsteds		1		
DG079K;Intraspinalt granulom ved sygdom klassificeret andetsteds		1		
DG08;Intrakraniel og intraspinal flebitis og tromboflebitis		1		
DG089;Intrakraniel eller intraspinal flebitis eller tromboflebitis		1		
DG09;Følger efter betændelsessygdomme i centralnervesystemet		1		
DG099;Følge efter inflammatorisk sygdom i centralnervesystemet		1		
DG10;Huntingtons sygdom		1		
DG109;Huntingtons sygdom		1		
DG11;Arvelig ataksi og paraplegi		1		
DG110;Ataxia nonprogressiva congenita		1		
DG111;Ataxia cerebellaris med tidlig debut		1		
DG111A;Kønsbunden recessiv ataxia spinocerebellaris		1		
DG111B;Ataxia spinalis hereditaria		1		
DG111C;Friedreichs ataksi		1		

Supplement 2 Nielsen et al. Mortality after paediatric emergency calls for patients with and without pre-existing comorbidity (2023)

DG112;Ataxia cerebellaris med sen debut		1	
DG113;Ataxia cerebellaris med defekt DNA-reparation		1	
DG113A;Ataxia telangiectasia		1	
DG114;Arvelig spastisk paraplegi			2
DG118;Anden arvelig ataksi		1	
DG119;Arvelig ataksi UNS		1	
DG12;Spinale muskelatrofier og beslægtede syndromer		1	
DG120;Atrophia musculorum spinalis, type I			2
DG121;Anden arvelig spinal muskelatrofi		1	
DG121A;Atrophia musculorum spinalis, voksen form		1	
DG121B;Atrophia musculorum spinalis, skapuloperoneal form		1	
DG121C;Atrophia musculorum spinalis, distal form		1	
DG121D;Atrophia musculorum spinalis, type II			2
DG121E;Atrophia musculorum spinalis, type III		1	
DG121F;Progressiv bulbær parese hos barn			2
DG122;Sygdom i motorneuroner			2
DG122A;Atrophia musculorum progressiva spinalis			2
DG122B;Atrophia musculorum progressiva myelopathica			2
DG122C;Atrophia musculorum Duchenne-Aran			2
DG122D;Atrophia musculorum progressiva			2
DG122E;Paralysis spinalis progressiva			2
DG122F;Paralysis bulbaris progressiva			2
DG122G;Amyotrofisk lateralsklerose			2
DG128;Anden spinal muskelatrofi eller beslægtet syndrom		1	
DG129;Spinal muskelatrofi UNS		1	
DG13;Generelle atrofier, som primært afficerer centralnervesystemet, ved sygdomme klassificeret andetsteds			2
DG130;Paraneoplastisk neuromyopati og neuropati			2
DG131;Anden generel atrofi i centralnervesystemet ved neoplastisk sygdom			2
DG132;Generel atrofi, som primært afficerer centralnervesystemet, ved myksødem		1	
DG138;Generel atrofi, som primært afficerer centralnervesystemet, ved anden sygdom		1	
DG14;Postpoliosyndrom		1	
DG149;Postpolio myelitisk syndrom		1	

DG20;Parkinsons sygdom		1	
DG209;Parkinsons sygdom		1	
DG209A;Hemiparkinsonisme		1	
DG21;Sekundær parkinsonisme		1	
DG210;Malignt neuroleptikasyndrom		1	
DG211;Anden medikamentel parkinsonisme		1	
DG212;Sekundær parkinsonisme forårsaget af andet eksternt agens		1	
DG213;Postencephalitisk parkinsonisme		1	
DG214;Vaskulær parkinsonisme		1	
DG218;Anden sekundær parkinsonisme		1	
DG218A;Parkinsonismus arterioscleroticus		1	
DG219;Sekundær parkinsonisme UNS		1	
DG22;Parkinsonisme ved sygdomme klassificeret andetsteds		1	
DG229;Parkinsonisme ved sygdom klassificeret andetsteds		1	
DG23;Andre degenerative sygdomme i basalganglier			2
DG230;Hallervorden-Spatz sygdom			2
DG231;Progressiv supranukleær oftalmoplegi			2
DG232;Multipel system atrofi, parkinson type (MSA-P)			2
DG233;Multipel system atrofi, cerebellar type (MSA-C)			2
DG238;Anden degenerativ sygdom i basalganglier			2
DG239;Degenerativ sygdom i basalganglier UNS			2
DG24;Dystoni		1	
DG240;Dystoni forårsaget af lægemiddel		1	
DG240A;Dyskinesia tardiva		1	
DG241;Idiopatisk familiær dystoni		1	
DG242;Idiopatisk ikke-familiær dystoni		1	
DG243;Spastisk torticollis		1	
DG244;Idiopatisk orofacial dystoni		1	
DG245;Blefarospasme		1	
DG248;Anden dystoni		1	
DG249;Dystoni UNS		1	
DG25;Andre ekstrapyramidale sygdomme og bevægeforstyrrelser		1	

Supplement 2 Nielsen et al. Mortality after paediatric emergency calls for patients with and without pre-existing comorbidity (2023)

DG250;Essentiel tremor		1	
DG251;Tremor forårsaget af lægemiddel		1	
DG252;Anden form for tremor		1	
DG252A;Intentionstremor		1	
DG253;Myoclonus		1	
DG253A;Myoclonus forårsaget af lægemiddel		1	
DG254;Chorea forårsaget af lægemiddel		1	
DG255;Anden form for chorea		1	
DG255A;Chorea UNS		1	
DG256;Tics forårsaget af lægemiddel eller andre organiske tics		1	
DG256A;Tics på organisk grundlag UNS		1	
DG258;Anden ekstrapyramidal sygdom eller bevægeforstyrrelse		1	
DG258A;Restless legs syndrom		1	
DG258D;Stiff man-syndrom		1	
DG259;Ekstrapyramidal sygdom eller bevægeforstyrrelse UNS		1	
DG26;Ekstrapyramidale sygdomme og bevægeforstyrrelser ved sygdomme klassificeret andetsteds		1	
DG269;Ekstrapyramidal sygdom eller bevægeforstyrrelse ved sygdom klassificeret andetsteds		1	
DG30;Alzheimers sygdom			2
DG300;Alzheimers sygdom med tidlig debut			2
DG301;Alzheimers sygdom med sen debut			2
DG308;Anden form for Alzheimers sygdom			2
DG309;Alzheimers sygdom UNS			2
DG31;Andre degenerative sygdomme i nervesystemet IKA			2
DG310;Lokaliseret hjerneatrofi			2
DG310A;Progressiv isoleret afasi			2
DG310B;Picks sygdom			2
DG311;Senil degeneration af hjernen IKA			2
DG312;Degenerative forandringer i nervesystemet forårsaget af alkohol		1	
DG312A;Alkoholisk cerebellar ataksi		1	
DG312B;Alkoholisk cerebral degeneration		1	
DG312C;Alkoholisk cerebellar degeneration		1	
DG312D;Alkoholisk dysfunktion af det autonome nervesystem		1	

DG312E;Alkoholisk encefalopati		1	
DG318;Anden degenerativ sygdom i nervesystemet			2
DG318A;Alpers' sygdom			2
DG318B;Hjernebarksdegeneration			2
DG318C;Subakut nekrotiserende encefalopati			2
DG318E;Lewy body sygdom			2
DG319;Degenerativ sygdom i nervesystemet UNS			2
DG32;Andre degenerative tilstande i nervesystemet ved sygdomme klassificeret andetsteds			2
DG320;Subakut degeneration af rygmarven ved sygdom klassificeret andetsteds			2
DG328;Anden degenerativ tilstand i nervesystemet ved sygdom klassificeret andetsteds			2
DG35;Dissemineret sklerose		1	
DG359;Dissemineret sklerose UNS		1	
DG359A;Attakvis dissemineret sklerose		1	
DG359B;Primær dissemineret sklerose		1	
DG359C;Progressiv dissemineret sklerose		1	
DG36;Anden akut dissemineret demyelinisering		1	
DG360;Neuromyelitis optica		1	
DG361;Akut eller subakut hæmoragisk leukoencephalitis		1	
DG368;Anden akut dissemineret demyelinisering		1	
DG369;Akut dissemineret demyelinisering UNS		1	
DG37;Andre demyeliniserende sygdomme i centralnervesystemet			2
DG370;Diffus cerebral sklerose			2
DG370A;Encephalitis periaxialis			2
DG371;Demyelinisatio centralis corporis callosi			2
DG372;Myelinolysis pontis centralis			2
DG373;Akut tranversel myelitis ved demyeliniserende sygdom i centralnervesystemet		1	
DG373A;Akut tranversel myelitis UNS		1	
DG374;Subakut nekrotiserende myelitis		1	
DG375;Encephalitis periaxialis concentrica			2
DG378;Anden demyeliniserende sygdom i centralnervesystemet			2
DG379;Demyeliniserende sygdom i centralnervesystemet UNS			2
DG40;Epilepsi		1	

DG400;Fokal idiopatisk epilepsi		1	
DG400A;Benign børneepilepsi med centrotemporale spikes		1	
DG400B;Benign børneepilepsi med occipitale paroxysmer		1	
DG400D;Autosomal dominant natlig frontallapsepilepsi		1	
DG401;Fokal epilepsi kun med simple anfald		1	
DG401A;Frontallapsepilepsi kun med simple fokale anfald		1	
DG401B;Parietallapsepilepsi kun med simple fokale anfald		1	
DG401C;Occipitallapsepilepsi kun med simple fokale anfald		1	
DG401D;Temporallapsepilepsi kun med simple fokale anfald		1	
DG401E;Temporallapsepilepsi med hippocampussklerose kun med simple anfald		1	
DG402;Fokal epilepsi med komplekse anfald eller generaliseret tonisk-klonisk anfald (GTCS)		1	
DG402A;Frontallapsepilepsi med komplekse anfald eller generaliseret tonisk-klonisk anfald (GTCS)		1	
DG402B;Parietallapsepilepsi med komplekse fokale anfald eller generaliseret tonisk-klonisk anfald (GTCS)		1	
DG402C;Occipitallapsepilepsi med komplekse fokale anfald eller generaliseret tonisk-klonisk anfald (GTCS)		1	
DG402D;Temporallapsepilepsi med komplekse fokale anfald eller generaliseret tonisk-klonisk anfald (GTCS)		1	
DG402E;Temporallapsepilepsi med hippocampussklerose med komplekse anfald eller generaliseret tonisk-klonisk anfald (G		1	
DG403;Generaliseret idiopatisk epilepsi		1	
DG403A;Benign myoklon epilepsi i barndommen		1	
DG403B;Epilepsi med myoklone-astatiske anfald		1	
DG403C;Børneabsenceepilepsi		1	
DG403D;Epilepsi med myoklone absencer		1	
DG403E;Juvenil absenceepilepsi		1	
DG403F;Juvenil myoklon epilepsi		1	
DG403G;Idiopatisk generaliseret epilepsi kun med generaliseret tonisk-klonisk anfald (GTCS)		1	
DG403P;Neonatale anfald		1	
DG404;Epileptisk encefalopati		1	
DG404A;Tidlig myoklonusencefalopati		1	
DG404B;Ohtahara syndrom		1	
DG404C;Infantile spasmer		1	
DG404D;Svær myoklon epilepsi i barndommen		1	
DG404E;Lennox-Gastaut's syndrom (LGS)		1	
DG404G;Epilepsi med kontinuerlige spike waves under slow wave søvn (findes ikke i SKS)		1	

Supplement 2 Nielsen et al. Mortality after paediatric emergency calls for patients with and without pre-existing comorbidity (2023)

DG404H;Rasmussens syndrom		1	
DG405;Specielt epileptisk syndrom		1	
DG405K;Epilepsia partialis continua		1	
DG405L;Epilepsi med kontinuerlige spike waves under slow wave søvn		1	
DG406;Generaliseret tonisk-klonisk anfald UNS		1	
DG407;Abscenser uden grand mal anfald		1	
DG408;Anden epilepsi		1	
DG408A;Refleksepilepsier		1	
DG408B;Søvnrelateret epilepsi		1	
DG409;Epilepsi UNS		1	
DG409A;Uspecificeret epilepsi med GTCS		1	
DG41;Status epilepticus		1	
DG410;Generaliseret tonisk-klonisk status epilepticus		1	
DG411;Non-konvulsivt status epilepticus af absencetype		1	
DG412;Non-konvulsivt komplekst partielt status epilepticus		1	
DG418;Anden form for status epilepticus		1	
DG419;Status epilepticus UNS		1	
DG43;Migræne		1	
DG430;Migræne uden aura		1	
DG431;Migræne med aura		1	
DG431A;Aura uden hovedpine		1	
DG431B;Migræne-ekvivalenter		1	
DG431C;Hemicrania hemiplegica familiaris		1	
DG432;Status migrainosus		1	
DG433;Migræne med komplikation		1	
DG433A;Migræne med cerebralt infarkt		1	
DG433B;Migræne med hemiplegi		1	
DG438;Anden form for migræne		1	
DG438A;Hemicrania ophthalmoplegica		1	
DG438B;Hemicrania retinalis		1	
DG438C;Atypisk migræne		1	
DG439;Migræne UNS		1	

DG44;Andre hovedpinesyndromer			1	
DG440;Hortons hovedpine			1	
DG440A;Episodisk klyngehovedpine			1	
DG440B;Kronisk paroksysmal hemikrani			1	
DG441;Vaskulær hovedpine IKA			1	
DG442;Spændingshovedpine			1	
DG443;Kronisk posttraumatisk hovedpine			1	
DG444;Hovedpine forårsaget af lægemiddel IKA			1	
DG448;Andet hovedpinesyndrom			1	
DG45;Transitorisk cerebral iskæmi og beslægtede syndromer			1	
DG450;Vertebrobasilært syndrom			1	
DG450A;Arteria vertebralis-syndrom			1	
DG450B;Arteria basilaris-syndrom			1	
DG451;Arteria carotis-syndrom			1	
DG452;Insufficiens af flere eller dobbeltsidige præcerebrale arterier			1	
DG452A;Insufficiens af dobbeltsidige præcerebrale arterier			1	
DG453;Amaurosis fugax			1	
DG454;Global forbigående amnesi			1	
DG458;Anden transitorisk cerebral iskæmi eller beslægtet syndrom			1	
DG459;Transitorisk anfald af cerebral iskæmi UNS			1	
DG459A;Spasme i cerebral arterie			1	
DG46;Symptomkomplekser ved cerebrovaskulære sygdomme			1	
DG460;Arteria cerebri media-syndrom			1	
DG461;Arteria cerebri anterior-syndrom			1	
DG462;Arteria cerebri posterior-syndrom			1	
DG463;Cerebrovaskulært hjernestammesyndrom			1	
DG463A;Benedikts syndrom			1	
DG463B;Claudes syndrom			1	
DG463C;Fovilles syndrom			1	
DG463D;Millard-Gublers syndrom			1	
DG463E;Wallenbergs syndrom			1	
DG463F;Webers syndrom			1	

DG464;Cerebrovaskulært cerebellart syndrom		1	
DG465;Isoleret motorisk lakunært syndrom		1	
DG466;Isoleret sensorisk lakunært syndrom		1	
DG467;Andet lakunært syndrom		1	
DG468;Andet vaskulært syndrom ved cerebrovaskulær sygdom		1	
DG47;Søvnforstyrrelser		1	
DG470;Insomni		1	
DG4700;Fysiologisk insomni		1	
DG4702;Insomni forårsaget af lægemiddel eller andet aktivt stof		1	
DG4703;Insomni forårsaget af sygdom klassificeret andetsteds		1	
DG471;Hypersomni		1	
DG4710;Organisk hypersomni UNS		1	
DG4711;Periodisk hypersomni		1	
DG4712;Idiopatisk hypersomni med lang søvntid		1	
DG4713;Idiopatisk hypersomni uden lang søvntid		1	
DG4714;Hypersomni betinget af lægemiddel eller andet aktivt stof		1	
DG4715;Hypersomni betinget af sygdom klassificeret andetsteds		1	
DG472;Forstyrret søvnrytme		1	
DG4720;Forstyrret søvnrytme UNS		1	
DG4721;Forstyrret søvnrytme på grund af forsinket søvnfase		1	
DG4722;Forstyrret søvnrytme på grund af tidlig søvnfase		1	
DG4723;Forstyrret søvnrytme på grund af uregelmæssig søvnfase		1	
DG4724;Forstyrret søvnrytme på grund af frit løbende søvnfase		1	
DG4725;Anden tilstand med forstyrret søvnrytme på grund af lægemiddel eller andet aktivt stof		1	
DG4726;Forstyrret søvnrytme på grund af sygdom klassificeret andetsteds		1	
DG473;Søvnapnø		1	
DG4730;Søvnapnø ved søvnrelateret respirationsygdom UNS		1	
DG4731;Primær central søvnapnø		1	
DG4732;Obstruktiv søvnapnø		1	
DG4734;Idiopatisk søvnrelateret ikke-obstruktiv hypoventilation		1	
DG4735;Søvnrelateret hypoventilation eller hypoxæmi på grund af medicinsk sygdom		1	
DG4735A;Søvnrelateret hypoventilation eller hypoxæmi på grund af lunge- eller karsygdom		1	

Supplement 2 Nielsen et al. Mortality after paediatric emergency calls for patients with and without pre-existing comorbidity (2023)

DG4735B;Søvnrelateret hypoventilation eller hypoxæmi på grund af nedre luftvejsobstruktion		1	
DG4735C;Søvnrelateret hypoventilation eller hypoxæmi på grund af neuromuskulær sygdom eller sygdom i brystvæggen		1	
DG4735D;Søvnrelateret hypoventilation eller hypoxæmi på grund af sygdom i centralnervesystemet		1	
DG4735E;Kongenit centralt hypoventilationssyndrom		1	
DG4736;Central apnø forårsaget af lægemiddel		1	
DG4739;Anden central apnø på grund af sygdom klassificeret andetsteds		1	
DG474;Narkolepsi og katapleksi		1	
DG4741;Narkolepsi med katapleksi		1	
DG4742;Narkolepsi uden katapleksi		1	
DG4743;Narkolepsi udløst af sygdom klassificeret andetsteds		1	
DG4744;Narkolepsi UNS		1	
DG475;Parasomni UNS		1	
DG4751;Natlig konfusion (søvndrukken)		1	
DG4752;Adfærdssyndrom ved REM-søvn (RBD)		1	
DG4753;Søvnparalyse		1	
DG4754;Parasomni på grund af lægemiddel eller substans		1	
DG4755;Parasomni på grund af medicinsk sygdom		1	
DG4756;Catathrenia		1	
DG4757;Exploding head syndrome		1	
DG4759;Søvn-relateret overspisning		1	
DG475A;Parasomni hyppigt associeret med REM søvn		1	
DG475B;Parasomni hyppigt associeret med NREM søvn		1	
DG475W;Anden parasomni		1	
DG476;Søvnrelateret bevægesygdom		1	
DG4760;Søvnrelateret rytmisk bevægeforstyrrelse UNS		1	
DG4761;Rastløse ben		1	
DG4762;Periodisk bevægelser i ekstremiteterne		1	
DG4763;Søvnrelaterede benkramper		1	
DG4764;Tænderskæren		1	
DG4765;Søvnrelateret rytmisk bevægeforstyrrelse		1	
DG4766;Søvnrelateret rytmisk bevægeforstyrrelse udløst af lægemiddel eller substans		1	
DG4767;Søvnrelateret rytmisk bevægeforstyrrelse udløst af medicinsk sygdom		1	

DG478;Anden form for søvnforstyrrelse		1		
DG478B;Menstruationsrelateret hypersomni		1		
DG479;Søvnforstyrrelse UNS		1		
DG50;Sygdomme i ansigtets følenerve		1		
DG500;Trigeminusneuralgi		1		
DG500A;Paroksysmatisk ansigtssmertesyndrom		1		
DG500B;Neuralgia nervi supraorbitalis		1		
DG500C;Neuritis nervi trigemini (V)		1		
DG500D;Neuritis nervi supraorbitalis		1		
DG500E;Tics douloureux		1		
DG501;Atypiske ansigtssmerter		1		
DG508;Anden sygdom i ansigtets følenerve		1		
DG509;Sygdom i ansigtets følenerve UNS		1		
DG51;Sygdomme i ansigtets bevægenerve		1		
DG510;Paralyse af ansigtets bevægenerve		1		
DG511;Ganglionitis geniculi		1		
DG512;Melkersson-Rosenthals syndrom		1		
DG513;Spasmus hemifacialis clonicus		1		
DG514;Myokymia facialis		1		
DG518;Anden sygdom i ansigtets bevægenerve		1		
DG518A;Degeneratio nervi facialis		1		
DG518B;Hemiatrophia nervi facialis		1		
DG518C;Neuralgia nervi facialis (VII)		1		
DG518D;Neuritis nervi facialis (VII)		1		
DG519;Sygdom i ansigtets bevægenerve UNS		1		
DG52;Sygdomme i andre kranienerver		1		
DG520;Sygdom i lugtenerven		1		
DG521;Sygdom i nervus glossopharyngeus (IX)		1		
DG521A;Neuralgia nervi glossopharyngei		1		
DG522;Sygdom i nervus vagus (X)		1		
DG523;Sygdom i nervus hypoglossus (XII)		1		
DG527;Sygdom samtidigt i flere kranienerver		1		

DG528;Sygdom i anden kranienerve		1	
DG529;Sygdom i kranienerve UNS		1	
DG53;Forandringer i kranienerver ved sygdomme klassificeret andetsteds		1	
DG530;Parese af flere kranienerver ved infektiøs eller parasitær sygdom klassificeret andetsteds		1	
DG530A;Ganglionitis geniculata postherpetica		1	
DG530B;Neuralgia nervi trigemini (V) postherpetica		1	
DG530C;Neuralgia cranialis postherpetica		1	
DG531;Paralyse af flere kranienerver ved infektiøs eller parasitær sygdom klassificeret andetsteds		1	
DG532;Parese af flere kranienerver ved sarkoidose		1	
DG533;Parese af flere kranienerver ved neoplastisk sygdom		1	
DG538;Affektion af kranienerver ved anden sygdom klassificeret andetsteds		1	
DG54;Sygdomme i nerverødder og nerveplekser		1	
DG540;Lidelse i plexus brachialis		1	
DG540A;Brachialisneuralgi		1	
DG540B;Costa cervicalis-syndrom		1	
DG540C;Skalenersyndrom		1	
DG541;Lidelse i plexus lumbosacralis		1	
DG542;Lidelse i cervikal nerverod IKA		1	
DG543;Lidelse i torakal nerverod IKA		1	
DG544;Lidelse i lumbal nerverod IKA		1	
DG545;Amyotrophia neuralgica		1	
DG546;Fantomsyndrom med smerter		1	
DG547;Fantomsyndrom uden smerter		1	
DG547A;Fantomsyndrom UNS		1	
DG548;Anden sygdom i nerverod eller nerveplexus		1	
DG549;Sygdom i nerverod eller nerveplexus UNS		1	
DG55;Kompression af nerverødder og nerveplekser ved sygdomme klassificeret andetsteds		1	
DG550;Kompression af nerverod eller nerveplexus ved neoplastisk sygdom		1	
DG550A;Kompression af nerveplexus ved neoplastisk sygdom		1	
DG550B;Kompression af nerverod ved neoplastisk sygdom		1	
DG551;Kompression af nerverod eller nerveplexus ved diskuslidelse i rygsøjlen		1	
DG551A;Kompression af nerveplexus ved diskuslidelse i rygsøjlen		1	

Supplement 2 Nielsen et al. Mortality after paediatric emergency calls for patients with and without pre-existing comorbidity (2023)

DG551B;Kompression af nerverod ved diskuslidelse i rygsøjlen		1	
DG552;Kompression af nerverod eller nerveplexus ved spondylose		1	
DG552A;Kompression af nerveplexus ved spondylose		1	
DG552B;Kompression af nerverod ved spondylose		1	
DG553;Kompression af nerverod eller nerveplexus ved anden ryglidelse		1	
DG558;Kompression af nerverod eller nerveplexus ved anden sygdom klassificeret andetsteds		1	
DG56;Mononeuropati i arm		1	
DG560;Karpaltunnelsyndrom		1	
DG561;Anden neuropati i nervus medianus		1	
DG562;Neuropati i nervus ulnaris		1	
DG562A;Paralysis nervi ulnaris tarda		1	
DG563;Neuropati i nervus radialis		1	
DG564;Kausalgi i arm		1	
DG568;Anden mononeuropati i overekstremitet		1	
DG568A;Interdigitalt neurom på overekstremitet		1	
DG569;Mononeuropati på overekstremitet UNS		1	
DG57;Mononeuropati i ben		1	
DG570;Neuropati i nervus ischiadicus		1	
DG571;Meralgia paraesthetica		1	
DG571A;Laesio nervi cutanei femoris lateralis syndrom		1	
DG572;Neuropati i nervus femoralis		1	
DG573;Neuropati i nervus peronaeus		1	
DG574;Neuropati i nervus tibialis		1	
DG575;Tarsaltunnelsyndrom		1	
DG576;Neuropati i nervus plantaris		1	
DG576A;Mortons metatarsalgia		1	
DG578;Anden mononeuropati på underekstremitet		1	
DG578A;Interdigitalt neurom på underekstremitet		1	
DG579;Mononeuropati på underekstremitet UNS		1	
DG58;Andre mononeuropatier		1	
DG580;Interkostal neuropati		1	
DG587;Neuropati samtidigt i flere enkelte nerver		1	

DG588;Anden mononeuropati		1	
DG589;Mononeuropati UNS		1	
DG59;Mononeuropati ved sygdomme klassificeret andetsteds		1	
DG590;Diabetisk mononeuropati		1	
DG598;Mononeuropati ved anden sygdom klassificeret andetsteds		1	
DG60;Arvelige og idiopatiske neuropatier		1	
DG600;Arvelig motorisk-sensorisk neuropati		1	
DG600A;Hypertrofisk peroneal muskelatrofi		1	
DG600B;Neuropatisk muskelatrofi		1	
DG600C;Axonal peroneal muskelatrofi		1	
DG600D;Atrophia Charcot-Marie-Tooth		1	
DG600E;Déjerine-Sottas sygdom		1	
DG600F;Hypertrofisk neuropati i barndommen		1	
DG600G;Arvelig motorisk-sensorisk neuropati, type I-IV		1	
DG600I;Roussy-Lévys syndrom		1	
DG601;Refsums sygdom		1	
DG602;Neuropati ved ataxia hereditaria		1	
DG603;Idiopatisk progressiv neuropati		1	
DG608;Anden form for arvelig motorisk-sensorisk neuropati		1	
DG608A;Morvans sygdom		1	
DG608B;Nelatons syndrom		1	
DG608C;Recessiv arvelig sensorisk neuropati		1	
DG608D;Dominant arvelig sensorisk neuropati		1	
DG609;Arvelig eller idiopatisk neuropati UNS		1	
DG61;Inflammatoriske polyneuropatier		1	
DG610;Guillain-Barrés syndrom		1	
DG611;Serumneuropati		1	
DG618;Anden inflammatorisk polyneuropati		1	
DG619;Inflammatorisk polyneuropati UNS		1	
DG62;Andre polyneuropatier		1	
DG620;Polyneuropati forårsaget af lægemiddel		1	
DG621;Alkoholisk polyneuropati		1	

DG622;Polyneuropati forårsaget af andet toksisk agens		1	
DG628;Anden polyneuropati		1	
DG628A;Strålingsudløst polyneuropati		1	
DG629;Polyneuropati UNS		1	
DG63;Polyneuropatier ved sygdomme klassificeret andetsteds		1	
DG630;Polyneuropati ved infektiøs eller parasitær sygdom klassificeret andetsteds		1	
DG631;Polyneuropati ved neoplastisk sygdom klassificeret andetsteds		1	
DG632;Diabetisk polyneuropati		1	
DG633;Polyneuropati ved anden endokrin eller metabolisk sygdom klassificeret andetsteds		1	
DG634;Polyneuropati ved underernæring klassificeret andetsteds		1	
DG635;Polyneuropati ved generaliseret bindevævssygdom klassificeret andetsteds		1	
DG636;Polyneuropati ved anden muskulosskeletal sygdom klassificeret andetsteds		1	
DG638;Polyneuropati ved anden sygdom klassificeret andetsteds		1	
DG64;Andre sygdomme i det perifere nervesystem		1	
DG649;Anden sygdom i perifere nervesystem		1	
DG649A;Sygdom i perifere nervesystem UNS		1	
DG70;Myasthenia gravis og andre neuromuskulære sygdomme		1	
DG700;Myasthenia gravis		1	
DG701;Toksisk neuromuskulær sygdom		1	
DG702;Medfødt og udviklingsrelateret myasteni		1	
DG708;Anden neuromuskulær sygdom		1	
DG709;Neuromuskulær sygdom UNS		1	
DG71;Primære muskelsygdomme		1	
DG710;Muskeldystrofi		1	
DG710A;Dystrophia musculorum oculopharyngealis		1	
DG710B;Dystrophia musculorum progressiva			2
DG710C;Dystrophia musculorum oculi		1	
DG710D;Dystrophia musculorum typus limb-girdle		1	
DG710E;Dystrophia musculorum scapuloperonealis		1	
DG710F;Dystrophia musculorum progressiva hereditaria			2
DG710G;Dystrophia musculorum benigna		1	
DG710H;Duchennes muskeldystrofi			2

Supplement 2 Nielsen et al. Mortality after paediatric emergency calls for patients with and without pre-existing comorbidity (2023)

DG710I;Dystrophia musculorum facioscapulohumeralis		1	
DG710J;Dystrophia musculorum distalis		1	
DG711;Myoton sygdom		1	
DG711A;Dystrophia myotonica			2
DG711B;Dominant myotonia congenita			2
DG711C;Recessiv myotonia congenita			2
DG711D;Myotonia congenita UNS			2
DG711E;Myotoni forårsaget af lægemiddel		1	
DG711F;Myotonia symptomatica		1	
DG711G;Myotonia chondrodystrophica		1	
DG711I;Neuromyotonia		1	
DG711J;Paramyotonia congenita		1	
DG711K;Pseudomyotonia		1	
DG712;Medfødt myopati			2
DG712A;Medfødt muskeldystrofi UNS			2
DG712B;Medfødt flernukleær myopati			2
DG712C;Medfødt smånukleær myopati			2
DG712D;Medfødt centronukleær myopati			2
DG713;Mitokondriel myopati IKA			2
DG718;Anden primær muskelsygdom		1	
DG719;Primær muskelsygdom UNS		1	
DG72;Andre muskelsygdomme		1	
DG720;Myopati forårsaget af lægemiddel		1	
DG721;Alkoholisk myopati		1	
DG722;Myopati forårsaget af andet toksisk agens		1	
DG723;Paralysis periodica		1	
DG723A;Hypokaliæmisk periodisk paralyse		1	
DG723B;Hyperkaliæmisk periodisk paralyse		1	
DG723C;Myoton periodisk paralyse		1	
DG723D;Normokaliæmisk periodisk paralyse		1	
DG724;Inflammatorisk myopati IKA		1	
DG728;Anden myopati		1	

DG729;Myopati UNS		1	
DG73;Neuromuskulære og muskulære sygdomme ved sygdomme klassificeret andetsteds		1	
DG730;Myastenisk syndrom ved endokrin sygdom		1	
DG731;Lambert-Eatons syndrom		1	
DG732;Andet myastenisk syndrom ved neoplastisk sygdom			2
DG733;Myastenisk syndrom ved anden sygdom klassificeret andetsteds		1	
DG734;Myopati ved infektiøs eller parasitær sygdom klassificeret andetsteds		1	
DG734C;Inklusionslegeme myositis		1	
DG735;Myopati ved endokrin sygdom		1	
DG736;Myopati ved metabolisk sygdom		1	
DG737;Myopati ved anden sygdom klassificeret andetsteds		1	
DG80;Cerebral parese			2
DG800;Spastisk tetraplegisk cerebral parese			2
DG801;Spastisk diplegisk cerebral parese			2
DG802;Spastisk hemiplegisk cerebral parese			2
DG803;Dyskinetisk cerebral parese			2
DG803A;Atetotisk cerebral parese			2
DG803B;Chorea cerebralis			2
DG804;Ataktisk cerebral parese			2
DG808;Anden form for cerebral parese			2
DG808A;Blandet syndrom ved cerebral parese			2
DG809;Cerebral parese UNS			2
DG809A;Cerebral parese (findes ikke i SKS)			2
DG81;Hemiplegi			2
DG810;Slap hemiplegi			2
DG811;Spastisk hemiplegi			2
DG819;Hemiplegi UNS			2
DG82;Paraplegi og tetraplegi			2
DG820;Slap paraplegi			2
DG821;Spastisk paraplegi			2
DG822;Paraplegi UNS			2
DG822A;Paraparesis af underekstremiteter UNS			2

DG823;Slap tetraplegi			2
DG824;Spastisk tetraplegi			2
DG825;Tetraplegi UNS			2
DG83;Andre syndromer med lammelse		1	
DG830;Diplegi af overekstremiteter		1	
DG831;Monoplegi af underekstremitet		1	
DG832;Monoplegi af overekstremitet		1	
DG833;Monoplegi UNS		1	
DG834;Cauda equina-syndrom		1	
DG834A;Neurogen urinblære ved cauda equina syndrom		1	
DG834B;Neurogen claudicatio intermittens		1	
DG835;Locked-in-syndrom			2
DG838;Andet paralytisk syndrom			2
DG838A;Paralysis postepileptica		1	
DG839;Paralytisk syndrom UNS			2
DG90;Sygdomme i autonome nervesystem		1	
DG900;Idiopatisk perifer autonom neuropati		1	
DG900A;Sinus caroticus-synkope		1	
DG901;Familiær dysautonomi		1	
DG902;Horners syndrom		1	
DG904;Autonom dysrefleksi		1	
DG908;Anden sygdom i autonome nervesystem		1	
DG909;Sygdom i autonome nervesystem UNS		1	
DG909A;Neurogen ortostatisk hypotension		1	
DG909B;Shy-Dragers syndrom			2
DG91;Hydrocefalus		1	
DG910;Kommunikerende hydrocefalus		1	
DG911;Obstruktiv hydrocefalus		1	
DG912;Normaltrykshydrocefalus		1	
DG913;Posttraumatisk hydrocefalus UNS		1	
DG918;Anden form for hydrocefalus		1	
DG919;Hydrocefalus UNS		1	

Supplement 2 Nielsen et al. Mortality after paediatric emergency calls for patients with and without pre-existing comorbidity (2023)

DG92;Toksiske sygdomme i hjernen		1	
DG929;Toksisk encefalopati		1	
DG929A;Malersyndrom		1	
DG93;Andre hjernesygdomme		1	
DG930;Hjernecyste		1	
DG930A;Araknoidalcyste		1	
DG930B;Erhvervet porencefalcyste		1	
DG931;Anoksisk hjerneskade IKA			2
DG932;Benign intrakraniel trykforøgelse		1	
DG933;Postviralt træthedssyndrom		1	
DG933A;Benign myalgisk encephalomyelitis		1	
DG934;Encefalopati UNS		1	
DG935;Compressio cerebri			2
DG935A;Hjernestammekompression			2
DG935B;Hjerneinkarceration			2
DG935C;Hjernestammeinkarceration			2
DG936;Hjerneødem			2
DG937;Reyes syndrom		1	
DG938;Anden hjernesygdom			2
DG938A;Strålingsudløst encefalopati			2
DG939;Hjernesygdom UNS			2
DG94;Andre hjernesygdomme ved sygdomme klassificeret andetsteds			2
DG940;Hydrocefalus ved infektiøs eller parasitær sygdom klassificeret andetsteds		1	
DG941;Hydrocefalus ved neoplastisk sygdom klassificeret andetsteds			2
DG942;Hydrocefalus ved anden sygdom klassiificeret andetsteds		1	
DG943;Encefalopati i sygdom klassificeret andetsteds		1	
DG948;Anden hjernesygdom ved sygdom klassificeret andetsteds		1	
DG95;Andre sygdomme i rygmarven		1	
DG950;Syringomyeli eller syringobulbi		1	
DG950A;Syringobulbi		1	
DG950B;Syringomyeli		1	
DG951;Vaskulær myelopati		1	

DG951A;Blødning i rygmarven		1	
DG951B;Akut rygmarvsinfarkt		1	
DG951C;Subakut nekrotiserende myelopati		1	
DG951D;Rygmarvsødem		1	
DG951E;Ikke-pyogen intraspinal flebitis		1	
DG951F;Ikke-pyogen intraspinal tromboflebitis		1	
DG951G;Arteriel trombose i rygmarven		1	
DG952;Rygmarvskompression UNS		1	
DG952A;Medullært tværsnitssyndrom forårsaget af kompression		1	
DG958;Anden sygdom i rygmarv		1	
DG958A;Strålingsudløst myelopati			2
DG958B;Myelopati forårsaget af lægemiddel		1	
DG958C;Neurogen urinblære ved rygmarvsbeskadigelse		1	
DG959;Sygdom i rygmarven UNS		1	
DG96;Andre sygdomme i centralnervesystemet IKA		1	
DG960;Liquorrhoea cerebrospinalis		1	
DG960A;Otoliquorrhea cerebrospinalis		1	
DG960B;Rhinoliquorrhea cerebrospinalis		1	
DG961;Sygdom i meninges IKA		1	
DG961A;Adhaesiones meningum		1	
DG968;Anden sygdom i centralnervesystemet		1	
DG969;Sygdom i centralnervesystemet UNS		1	
DG97;Sygdomme i nervesystemet som følge af indgreb IKA		1	
DG970;Liquorrhoea cerebrospinalis efter lumbalpunktur		1	
DG971;Anden reaktion efter spinal- eller lumbalpunktur		1	
DG972;Nedsat intrakranielt tryk ved ventrikulær shunt		1	
DG978;Anden sygdom i nervesystemet som følge af indgreb		1	
DG978A;Hjerneanoksi som følge af indgreb		1	
DG979;Sygdom i nervesystemet som følge af indgreb UNS		1	
DG98;Andre sygdomme i nervesystemet IKA		1	
DG989;Anden sygdom i nervesystemet IKA		1	
DG99;Andre sygdomme i nervesystemet ved sygdomme klassificeret andetsteds		1	

Supplement 2 Nielsen et al. Mortality after paediatric emergency calls for patients with and without pre-existing comorbidity (2023)

DG990;Autonom neuropati ved endokrin eller metabolisk sygdom klassificeret andetsteds		1	
DG991;Anden sygdom i det autonome nervesystem ved sygdom klassificeret andetsteds		1	
DG992;Myelopati ved sygdom klassificeret andetsteds		1	
DG998;Anden sygdom i nervesystemet ved sygdom klassificeret andetsteds		1	
DH00;Hordeolum og chalazion	0		
DH000;Hordeolum og anden dyb betændelse i øjenlåg	0		
DH000A;Absces i øjenlåg	0		
DH000B;Furunkel i øjenlåg	0		
DH000C;Bygkorn	0		
DH000D;Flegmone i øjenlåg	0		
DH001;Chalazion	0		
DH01;Anden betændelse i øjenlåg	0		
DH010;Blefaritis	0		
DH011;Ikke-infektiøs dermatitis på øjenlåg	0		
DH011A;Eksematøs dermatitis på øjenlåg	0		
DH011B;Kontaktdermatitis på øjenlåg	0		
DH011C;Allergisk dermatitis på øjenlåg	0		
DH011D;Lupus erythematosus discoides palpebrae	0		
DH011E;Xeroderma palpebrae	0		
DH018;Anden betændelse af øjenlåg	0		
DH019;Betændelse af øjenlåg UNS	0		
DH02;Andre sygdomme i øjenlåg	0		
DH020;Entropion eller trichiasis på øjenlåg	0		
DH020A;Entropion palpebrae	0		
DH020B;Trichiasis palpebrae	0		
DH021;Ectropion palpebrae	0		
DH022;Lagoftalmi	0		
DH023;Blefarokalasi	0		
DH024;Blefaroptose	0		
DH025;Anden forstyrrelse i funktion af øjenlåg	0		
DH025A;Ankyloblepharon acquisitum	0		
DH025B;Blefarofimose	0		

DH025D;Retractio palpebrae	0			
DH026;Xanthelasma palpebrae	0			
DH027;Anden degenerativ lidelse i øjenlåg eller periorbital region	0			
DH027A;Chloasma palpebrae	0			
DH027B;Madarosis palpebrae	0			
DH027C;Vitiligo palpebrae	0			
DH028;Anden sygdom i øjenlåg	0			
DH028A;Retineret fremmedlegeme i øjenlåg	0			
DH028B;Dermatochalasis palpebrae	0			
DH028C;Hypertrichosis palpebrae	0			
DH029;Sygdom i øjenlåg UNS	0			
DH03;Affektion af øjenlåg ved sygdomme klassificeret andetsteds	0			
DH030;Affektion af øjenlåg ved parasitær infestation klassificeret andetsteds	0			
DH031;Affektion af øjenlåg ved anden infektiøs sygdom klassificeret andetsteds	0			
DH038;Affektion af øjenlåg ved anden sygdom klassificeret andetsteds	0			
DH04;Sygdomme i tåreapparatet	0			
DH040;Betændelse i tårekirtel	0			
DH041;Anden sygdom i tårekirtel	0			
DH041A;Atrofi af tårekirtel	0			
DH041B;Cyste i tårekirtel	0			
DH041C;Dacryocystectasia	0			
DH041D;Dacryocystocele	0			
DH041E;Dacryops	0			
DH041F;Xerophthalmus	0			
DH042;Epifora	0			
DH043;Akut eller ikke specificeret betændelse i tåreveje	0			
DH043A;Akut betændelse i tårekanal	0			
DH043B;Betændelse i tårekanal UNS	0			
DH043C;Akut betændelse i tåresæk	0			
DH043D;Betændelse i tåresæk UNS	0			
DH043E;Dacryopericystitis acuta	0			
DH043F;Dacryopericystitis UNS	0			

Supplement 2 Nielsen et al. Mortality after paediatric emergency calls for patients with and without pre-existing comorbidity (2023)

DH044;Kronisk betændelse i tåreapparat	0			
DH044A;Kronisk betændelse i tårekanal	0			
DH044C;Mucocele lacrimalis	0			
DH045;Stenose eller insufficiens i tåreveje	0			
DH045A;Dacryolithiasis	0			
DH045B;Eversio puncti lacrimalis	0			
DH045C;Stenosis viarum lacrimalium	0			
DH045D;Stenosis ducti nasolacrimalis	0			
DH045E;Stenosis sacci lacrimalis	0			
DH045F;Stenosis canaliculi lacrimalis	0			
DH046;Anden forandring i tåreapparat	0			
DH046A;Fistula lacrimalis	0			
DH048;Anden sygdom i tåreapparat	0			
DH049;Sygdom i tåreapparat UNS	0			
DH05;Sygdomme i øjenhule	0			
DH050;Akut betændelse i øjenhule	0			
DH050A;Absces i øjenhule	0			
DH050B;Cellulitis orbitae	0			
DH050C;Osteomyelitis orbitae	0			
DH050D;Periostitis orbitae	0			
DH050E;Flegmone i øjenhule	0			
DH050F;Tenonitis orbitae	0			
DH051;Kronisk betændelse i øjenhule	0			
DH051A;Granulom i øjenhule	0			
DH052;Eksoftalmi	0			
DH052A;Eksoftalmi forårsaget af blødning i øjenhule	0			
DH052B;Eksoftalmi forårsaget af ødem i øjenhule	0			
DH053;Deformering af øjenhule	0			
DH053A;Atrofi af øjenhule	0			
DH053B;Dysostosis orbitae	0			
DH053D;Exostosis orbitae	0			
DH054;Enofthalmi	0			

Supplement 2 Nielsen et al. Mortality after paediatric emergency calls for patients with and without pre-existing comorbidity (2023)

DH055;Retineret fremmedlegeme i øjenhule	0		
DH055B;Retrobulbært retineret fremmedlegeme i øjenhule	0		
DH058;Anden sygdom i øjenhule	0		
DH058A;Cyste i øjenhule	0		
DH058B;Myopati i ydre øjenmuskel	0		
DH059;Sygdom i øjenhule UNS	0		
DH06;Sygdomme i tåreapparat og øjenhule ved sygdomme klassificeret andetsteds	0		
DH060;Sygdom i tåreapparatet ved sygdom klassificeret andetsteds	0		
DH061;Parasitær infektion i øjenhule ved sygdom klassificeret andetsteds	0		
DH062;Eksoftalmi ved struma	0		
DH063;Anden sygdom i øjenhule ved sygdom klassificeret andetsteds	0		
DH10;Betændelse i øjets bindehinde	0		
DH100;Mukopurulent konjunktivitis	0		
DH101;Akut allergisk konjunktivitis	0		
DH102;Anden akut konjunktivitis	0		
DH102A;Akut epidemisk konjunktivitis eller keratokonjunktivitis (adenovirus)	0		
DH102B;Akut medikamentelt induceret konjunktivitis eller keratokonjunktivitis	0		
DH102C;Toksisk konjunktivitis eller keratokonjunktivitis	0		
DH103;Akut konjunktivitis UNS	0		
DH104;Kronisk konjunktivitis	0		
DH104A;Kronisk allergisk konjunktivitis	0		
DH104A0;Helårs (perennial) konjunktivitis	0		
DH104A1;Vernal konjunktivitis eller keratokonjunktivitis	0		
DH104B;Kronisk follikulær konjunktivitis	0		
DH104C;Kronisk papillær konjunktivitis	0		
DH104D;Kronisk konjunktivitis eller keratokonjunktivitis sicca uden Sjögrens syndrom	0		
DH105;Blefarokonjunktivitis	0		
DH106;Cikatriciel konjunktivitis	0		
DH108;Anden form for konjunktivitis	0		
DH109;Konjunktivitis UNS	0		
DH11;Andre sygdomme i øjets bindehinde	0		
DH110;Pterygium	0		

Supplement 2 Nielsen et al. Mortality after paediatric emergency calls for patients with and without pre-existing comorbidity (2023)

DH111;Degeneration af og aflejring i konjunktiva	0		
DH111B;Forkalkning i konjunktiva	0		
DH111C;Degeneration af konjunktiva	0		
DH111D;Pigmentering af konjunktiva	0		
DH111E;Tørhed af conjunctiva (findes ikke i SKS)	0		
DH111F;Pingueculum	0		
DH111G;Degeneration af eller aflejring i konjunktiva UNS	0		
DH112;Ardannelse i konjunktiva	0		
DH112A;Symblepharon	0		
DH113;Blødning i konjunktiva	0		
DH113A;Subkonjunktival blødning	0		
DH114;Anden vaskulær lidelse eller cyste i konjunktiva	0		
DH114A;Aneurisme i konjunktiva	0		
DH114B;Cyste i konjunktiva	0		
DH114C;Hyperæmi af konjunktiva	0		
DH114D;Ødem i konjunktiva (chemosis)	0		
DH118;Anden sygdom i konjunktiva	0		
DH118A;Pseudopterygium	0		
DH119;Sygdom i konjunktiva UNS	0		
DH13;Sygdomme i øjets bindehinde ved sygdomme klassificeret andetsteds	0		
DH130;Filariasis i konjunktiva	0		
DH131;Konjunktivitis ved infektion eller parasitær sygdom klassificeret andetsteds	0		
DH132;Konjunktivitis ved anden sygdom klassificeret andetsteds	0		
DH133;Pemphigus oculi	0		
DH134;Konjunktivitis eller keratokonjunktivitis ved anden sygdom klassificeret andetsteds	0		
DH134A;Konjunktivitis eller keratokonjunktivitis sicca ved Sjögrens syndrom	0		
DH134B;Konjunktivitis eller keratokonjunktivitis sicca ved graft versus host sygdom	0		
DH134C;Konjunktivitis eller keratokonjunktivitis ved atopi	0		
DH134D;Blefarokonjunktivitis eller blefarokeratokonjunktivitis ved okulær rosacea	0		
DH138;Anden sygdom i konjunktiva ved sygdom klassificeret andetsteds	0		
DH15;Sygdomme i sclera	0		
DH150;Betændelse i sclera	0		

DH151;Episcleritis	0		
DH158;Anden sygdom i sclera	0		
DH158B;Staphyloma equatorialis	0		
DH159;Sygdom i sclera UNS	0		
DH16;Keratitis	0		
DH160;Sår i hornhinde	0		
DH160B;Ulcus centrale corneae	0		
DH160F;Ulcus perforatum corneae	0		
DH160G;Ulcus marginale corneae	0		
DH160G0;Perifer ulcerativc keratit	0		
DH160G1;Mooren's ulcus	0		
DH160H;Ulcus corneae med hypopyon	0		
DH161;Ikke infektiøs-keratitis eller keratopati	0		
DH161A;Keratitis stellata (findes ikke i SKS)	0		
DH161C;Keratitis punctata superficialis	0		
DH161D;Keratitis nummularis (findes ikke i SKS)	0		
DH161F;Keratitis filamentosa	0		
DH161G;Keratitis actinica	0		
DH161G0;Sneblindhed	0		
DH161G1;Svejserøjne	0		
DH161J;Neurotrofisk keratopati	0		
DH161K;Ekspositionskeratitis eller ekspositionskeratopati	0		
DH161M;Anden specificeret ikke infektiøs keratitis eller keratopati	0		
DH161N;Ikke infektiøs keratitis eller keratopati UNS	0		
DH162;Keratokonjunktivitis	0		
DH162A;Keratokonjunktivitis phlyctaenularis	0		
DH163;Keratitis profunda interstitialis	0		
DH164;Pannus corneae	0		
DH165;Infektiøs keratitis	0		
DH165A;Bakteriel keratitis	0		
DH165B;Keratitis forårsaget af Herpes Simplex virus	0		
DH165B0;Keratitis dendritica	0		

Supplement 2 Nielsen et al. Mortality after paediatric emergency calls for patients with and without pre-existing comorbidity (2023)

DH165B1;Keratitis disciformis	0		
DH165B2;Endothelitis	0		
DH165C;Keratitis som led i Herpes Zoster	0		
DH165D;Keratitis forårsaget af Acanthamøba	0		
DH165E;Keratitis forårsaget af gær- eller skimmelsvamp	0		
DH165F;Anden specificeret mikrobiel keratitis	0		
DH165G;Mikrobiel keratitis UNS	0		
DH168;Anden form for keratitis	0		
DH169;Keratitis UNS	0		
DH17;Ar og uklarheder i cornea	0		
DH170;Leukoma adhaerens	0		
DH171;Anden central opacitet i cornea	0		
DH178;Anden cicatrice eller opacitet i cornea	0		
DH179;Cicatrice eller opacitet i cornea UNS	0		
DH18;Andre sygdomme i hornhinde	0		
DH180;Pigmentering eller aflejring i hornhinde	0		
DH180A;Haematocornea	0		
DH180B;Kayser-Fleischer ring		1	
DH180C;Krukenbergs spindel	0		
DH180D;Pigmentatio corneae	0		
DH180E;Stählis pigmentlinje	0		
DH180F;Anden specificeret aflejring i hornhinde	0		
DH180G;Aflejring i hornhinde UNS	0		
DH181;Bulløs keratopati	0		
DH181A;Bulløs keratopati efter kataraktoperation	0		
DH181B;Bulløs keratopati sekundært til intraokulært implantat	0		
DH181C;Bulløs keratopati sekundært til glaukom	0		
DH181D;Bulløs keratopati sekundært til uveit	0		
DH181E;Anden specificeret form for bulløs keratopati	0		
DH181F;Bulløs keratopati UNS	0		
DH182;Anden form for ødem i hornhinde	0		
DH183;Forandringer i hornhindens membraner	0		

DH183A;Haab'ske striae	0			
DH183B;Hydrops corneae	0			
DH184;Hornhindedegeneration	0			
DH184A;Arcus senilis	0			
DH184B;Keratopatia bandularis	0			
DH184C;Salzmann's nodulære degeneration	0			
DH184D;Terrien's marginale degeneration	0			
DH184E;Shagrinering af hornhinden (Crocodile shagreen)	0			
DH184F;Lipid degeneration sekundært til karindvækst	0			
DH185;Familiær corneadystrofi	0			
DH185A;Fuchs corneadystrofi	0			
DH185B;Epitel basalmembran dystrofi (Cogans dystrofi/map-dot-fingerprint dystrofi)	0			
DH185C;Granulær dystrofi type 1	0			
DH185D;Lattice dystrofi	0			
DH185E;Makulær dystrofi	0			
DH185F;Posterior polymorf dystrofi	0			
DH185G;Anden specificeret familiær cornea dystrofi	0			
DH185H;Famililær cornea dystrofi UNS	0			
DH186;Keratoconus	0			
DH187;Anden deformitet af hornhinde	0			
DH187A;Descemetocele	0			
DH187B;Ectasia corneae	0			
DH187B0;Ectasia corneae efter refraktiv kirurgi	0			
DH187D;Staphyloma corneae	0			
DH188;Anden sygdom i hornhinde	0			
DH188A;Anaesthesia corneae	0			
DH188B;Erosio recidivans corneae	0			
DH188C;Hypaesthesia corneae	0			
DH189;Sygdom i hornhinde UNS	0			
DH19;Sygdomme i øjets senehinde og hornhinde ved sygdomme klassificeret andetsteds	0			
DH190;Skleritis eller episkleritis ved sygdom klasssificeret andetsteds	0			
DH190B;Episkleritis ved sygdom klassificeret andetsteds	0			

Supplement 2 Nielsen et al. Mortality after paediatric emergency calls for patients with and without pre-existing comorbidity (2023)

DH190E;Skleritis ved sygdom klassificeret andetsteds	0			
DH191;Keratitis el. keratokonjunktivitis f.a. Herpes simplex-virus (findes ikke i SKS)	0			
DH191A;Keratitis dendritica (findes ikke i SKS)	0			
DH192;Keratitis og keratokonjunktivitis ved anden infektiøs eller parasitær sygdom klassificeret ar	0			
DH193;Keratitis og keratokonjunktivitis ved anden sygdom klassificeret andetsteds	0			
DH193C;Keratokonjunctivitis ved sygdom klassificeret andetsteds (findes ikke i SKS)	0			
DH198;Anden sygdom i sclera eller cornea ved sygdom klassificeret andetsteds	0			
DH20;Betændelse i regnbuehinde og strålelegeme	0			
DH200;Akut og subakut anterior uveitis	0			
DH200F;Iritis acuta (findes ikke i SKS)	0			
DH200H;Iritis recidivans (findes ikke i SKS)	0			
DH200I;Uveitis anterior acuta (findes ikke i SKS)	0			
DH200J;Iridocyclitis recidivans (findes ikke i SKS)	0			
DH200K;Uveitis anterior subacuta (findes ikke i SKS)	0			
DH201;Kronisk anterior uveitis	0			
DH202;Linseinduceret anterior uveitis	0			
DH208;Anden form for anterior uveitis	0			
DH209;Anterior uveitis UNS	0			
DH21;Andre sygdomme i regnbuehinde og strålelegeme	0			
DH210;Hyphaema	0			
DH211;Anden vaskulær forandring i iris og corpus ciliare	0			
DH211A;Rubeosis iridis	0			
DH212;Degeneration af iris eller corpus ciliare	0			
DH212A;Atrofi af iris	0			
DH212B;Miotisk pupilcyste	0			
DH212C;Degeneration af iris	0			
DH212D;Degeneration af corpus ciliare	0			
DH212E;Degeneratio iridis marginalis pupillae	0			
DH212F;Degeneratio iridis pigmentosa	0			
DH212G;Iridoschisis	0			
DH212H;Iris translucens	0			
DH212I;Pupilsømsatrofi	0			

DH213;Cyste i iris, corpus ciliare eller forreste øjenkammer	0			
DH213A;Cyste i iris efter implantation	0			
DH213B;Cyste i iris forårsaget af parasit	0			
DH213C;Cystis iridis exudativa	0			
DH213D;Iriscyste UNS	0			
DH213E;Cyste i forreste øjenkammer UNS	0			
DH213F;Cystis camerae anterioris exudativa	0			
DH213G;Cyste i forreste øjenkammer efter implantation	0			
DH213H;Cyste i corporis ciliare UNS	0			
DH213I;Cystis corporis ciliaris exudativa	0			
DH213J;Cyste i corporis ciliare forårsaget af parasit	0			
DH213K;Cyste i corporis ciliare efter implantation	0			
DH213L;Cyste i forreste øjenkammer forårsaget af parasit	0			
DH214;Pupilmembraner	0			
DH214A;Iris bombé	0			
DH214B;Occlusio pupillae	0			
DH215;Anden adhærence eller rift i iris og corpus ciliare	0			
DH215A;Goniosynechia	0			
DH215B;Iridodialysis	0			
DH215C;Recessio anguli iridocornealis	0			
DH215D;Synechiae iridis posteriores	0			
DH215E;Synechiae iridis anteriores	0			
DH215F;Synekkier i iris UNS	0			
DH218;Anden forandring i iris eller corpus ciliare	0			
DH219;Forandring i iris eller corpus ciliare UNS	0			
DH22;Sygdomme i regnbuehinde og strålelegeme ved sygdomme klassificeret andetsteds	0			
DH220;Iridocyklitis ved infektiøs eller parasitær sygdom klassificeret andetsteds	0			
DH221;Iridocyklitis ved anden sygdom klassificeret andetsteds	0			
DH228;Anden sygdom i iris eller corpus ciliare ved sygdom klassificeret andetsteds	0			
DH25;Aldersbetinget grå stær (>=50 år)	0			
DH250;Begyndende aldersbetinget grå stær	0			
DH250B;Bagre polstær	0			

Supplement 2 Nielsen et al. Mortality after paediatric emergency calls for patients with and without pre-existing comorbidity (2023)

DH250E;Forreste polstær	0			
DH251;Aldersbetinget nukleær grå stær	0			
DH258;Anden eller kombineret aldersbetinget grå stær	0			
DH259;Aldersbetinget grå stær (>=50 år) UNS	0			
DH26;Andre former for grå stær	0			
DH260;Grå stær hos småbørn, børn, unge og voksne	0			
DH260A;Grå stær hos småbørn (<2 år)	0			
DH260B;Grå stær hos børn og unge (2 år til <18 år)	0			
DH260C;Grå stær hos yngre voksne (18 år til <50 år)	0			
DH261;Traumatisk grå stær	0			
DH262;Grå stær sekundært til anden øjensygdom	0			
DH263;Grå stær forårsaget af lægemiddel	0			
DH264;Efterstær	0			
DH265;Kapsulophimosis	0			
DH268;Anden form for grå stær	0			
DH269;Grå stær UNS	0			
DH269A;Svulmende grå stær	0			
DH27;Andre forandringer i øjets krystallinske linse	0			
DH270;Afaki	0			
DH271;Dislokation af øjets krystallinske linse	0			
DH271B;Luksation af øjets krystallinske linse (findes ikke i SKS)	0			
DH278;Anden forandring i øjets krystallinske linse	0			
DH278A;Linserest efter grå stær operation	0			
DH279;Forandring i øjets krystallinske linse UNS	0			
DH28;Grå stær og andre sygdomme i øjets krystallinske linse ved sygdomme klassificeret andets	0			
DH280;Diabetisk grå stær	0			
DH281;Grå stær ved endokrin, ernæringsbetinget eller metabolisk sygdom klassificeret andetsted	0			
DH282;Grå stær ved anden sygdom klassificeret andetsteds	0			
DH288;Anden sygdom i øjets krystallinske linse ved sygdom klassificeret andetsteds	0			
DH30;Betændelse i øjets årehinde og nethinde	0			
DH300;Fokal posterior uveitis	0			
DH301;Dissemineret posterior uveitis	0			

Supplement 2 Nielsen et al. Mortality after paediatric emergency calls for patients with and without pre-existing comorbidity (2023)

DH302;Intermediær uveitis	0			
DH308;Anden form for posterior uveitis	0			
DH309;Posterior uveitis UNS	0			
DH31;Andre forandringer i øjets årehinde	0			
DH310;Korioretinale ar	0			
DH310A;Ar i macula lutea	0			
DH310B;Retinopathia solaris	0			
DH311;Degeneratio chorioideae	0			
DH311A;Atrophia chorioideae	0			
DH311B;Sclerosis chorioideae	0			
DH312;Dystrophia choroidalis hereditaria	0			
DH312A;Atrophia gyrata chorioideae	0			
DH312B;Choroideremia	0			
DH312C;Dystrophia choroideae peripapillaris	0			
DH312D;Dystrophia choroideae areolaris	0			
DH312E;Dystrophia choroideae generalisata	0			
DH312F;Dystrophia choroideae centralis	0			
DH313;Blødning eller ruptur i årehinden	0			
DH313A;Haemorrhagia chorioideae expulsiva	0			
DH313B;Haemorrhagia chorioideae	0			
DH313C;Koroidalruptur	0			
DH314;Koroidalløsning	0			
DH318;Anden forandring i årehinde	0			
DH319;Forandring i årehinde UNS	0			
DH32;Forandringer i øjets årehinde og nethinde ved sygdomme klassificeret andetsteds	0			
DH320;Korioretinitis ved infektiøs eller parasitær sygdom klassificeret andetsteds	0			
DH328;Anden forandring i øjets årehinde og nethinde ved sygdom klassificeret andetsteds	0			
DH33;Nethindeløsning	0			
DH330;Nethindeløsning med ruptur	0			
DH330A;Nethindeløsning med ruptur, uden proliferativ vitreoretinopati (PVR)	0			
DH330A0;Nethindeløsning med ruptur, uden proliferativ vitreoretinopati (PVR), tilliggende makula	0			
DH330A1;Nethindeløsning med ruptur, uden proliferativ vitreoretinopati (PVR), afløst makula	0			

Supplement 2 Nielsen et al. Mortality after paediatric emergency calls for patients with and without pre-existing comorbidity (2023)

DH330B;Nethindeløsning med ruptur, med proliferativ vitreoretinopati (PVR)	0			
DH330B0;Nethindeløsning med ruptur, med proliferativ vitreoretinopati (PVR), tilliggende makula	0			
DH330B1;Nethindeløsning med ruptur, med proliferativ vitreoretinopati (PVR), afløst makula	0			
DH331;Retinoskise eller cyste i retina	0			
DH331A;Cystis retinae	0			
DH331B;Cystis orae serratae	0			
DH331C;Cystis retina e parasitica UNS	0			
DH331D;Pseudocystis retinae	0			
DH331E;Retinoschisis	0			
DH332;Amotio retinae serosa	0			
DH332A;Nethindeløsning uden ruptur	0			
DH333;Ruptur af retina uden løsning	0			
DH333A;Foramen retinae sine amotione	0			
DH333B;Operculum retinae sine amotione	0			
DH334;Amotio retinae tractionis	0			
DH334A;Proliferativ vitreoretinopati med amotio retinae	0			
DH334B;Diabetisk retinal traktionsamotio	0			
DH335;Anden form for nethindeløsning	0			
DH34;Okklusion af retinale blodkar	0			
DH340;Transitorisk okklusion af retinalarterie	0			
DH340A;Ischaemia transitoria retinae	0			
DH341;Occlusio arteriae centralis retinae	0			
DH341A;Embolia arteriae centralis retinae	0			
DH341B;Thrombosis arteriae centralis retinae	0			
DH342;Anden form for okklusion af retinalarterie	0			
DH342A;Kolesterolplaques i retina	0			
DH342B;Microembolus retinae	0			
DH342C;Occlusio arteriae retinae partialis	0			
DH348;Anden vaskulær okklusion i retina	0			
DH348A;Occlusio venae retinae incipiens	0			
DH348B;Occlusio venae retinae UNS	0			
DH348C;Occlusio venae retinae partialis	0			

DH348D;Occlusio venae centralis retinae	0			
DH348E;Thrombosis venae retinae UNS	0			
DH348F;Thrombosis venae centralis retinae	0			
DH349;Okklusion af retinalt blodkar UNS	0			
DH35;Andre forandringer i øjets nethinde	0			
DH350;Retinopati og angiopati i retina	0			
DH350A;Angiopathia retinae	0			
DH350B;Fundus hypertonicus	0			
DH350C;Retinalt mikroaneurisme	0			
DH350D;Neovascularisatio retinae	0			
DH350E;Perivasculitis retinae	0			
DH350F;Retinopathia exudativa	0			
DH350G;Retinopathia gravidarum	0			
DH350H;Hypertensiv retinopati	0			
DH350I;Retinopati UNS	0			
DH350J;Varices retinae	0			
DH350K;Vasculitis retinae	0			
DH351;Præmaturitetsretinopati	0			
DH351A;Retrolental fibroplasi	0			
DH352;Anden proliferativ retinopati	0			
DH352A;Epiretinal fibrose	0			
DH353;Degeneratio maculae luteae et polus posterior retinae	0			
DH353A;Cystis maculae luteae	0			
DH353B;Degeneratio disciformis maculae luteae	0			
DH353C;Degeneratio maculae luteae senilis	0			
DH353D;Degeneratio polus posterior retinae	0			
DH353E;Degeneratio maculae luteae	0			
DH353F;Foramen maculae luteae	0			
DH353G;Kuhnt-Junius degeneration	0			
DH353H;Maculopathia toxica	0			
DH353J;Våd aldersrelateret makuladegeneration med subretinal karnydannelse	0			
DH353K;Våd aldersrelateret makuladegeneration uden subretinal karnydannelse	0			

DH353L;Tør aldersrelateret makuladegeneration (AMD)	0		
DH353M;Vitreomakulær traktion	0		
DH353N;Lamellært makulært hul	0		
DH353P;Makulære druser	0		
DH353Q;Angioid striae	0		
DH354;Perifer retinal degeneration	0		
DH354A;Degeneratio retinae palisadis	0		
DH354B;Degeneratio retinae reticularis	0		
DH354C;Degeneratio retinae latticis	0		
DH354D;Degeneratio retinae microcystoidis	0		
DH355;Familiær retinadystrofi	0		
DH355A;Dystrophia retinae albi punctata	0		
DH355B;Dystrophia tapetoretinalis	0		
DH355C;Dystrophia retinae vitelliformis	0		
DH355D;Dystrophia vitreo retinalis	0		
DH355E;Dystrophia retinae pigmentaria	0		
DH355F;Stargardts sygdom	0		
DH356;Retinal blødning	0		
DH357;Separation af retinale lag	0		
DH357A;Amotio epithelii pigmenti retinae	0		
DH357B;Chorioretinopathia centralis serosa	0		
DH358;Anden forandring i nethinde	0		
DH358A;Retinale druser	0		
DH359;Forandring i nethinde UNS	0		
DH36;Forandringer i øjets nethinde ved sygdomme klassificeret andetsteds	0		
DH360;Diabetisk retinopati UNS	0		
DH360H;Simpel diabetisk retinopati	0		
DH360J;Proliferativ diabetisk retinopati	0		
DH360K;Diabetisk makulopati	0		
DH368;Anden retinal forandring ved sygdom klassificeret andetsteds	0		
DH368A;Dystrophia retinae, ved forstyrrelse i lipidmetabolismen (findes ikke i SKS)	0		
DH368D;Retinopati ved anden sygdom klassificeret andetsteds	0		

DH368D1;Retinal dystrofi ved anden sygdom klassificeret andetsteds	0			
DH368D2;Proliferativ retinopati ved anden sygdom klassificeret andetsteds	0			
DH40;Glaukom	0			
DH400;Glaukom suspekt	0			
DH400A;Okulær hypertension	0			
DH400B;Suspekt vinkellukning	0			
DH400C;Akut vinkellukning	0			
DH400D;Suspekt papilekskavation	0			
DH400E;Pseudoexfoliationssyndrom uden glaukom	0			
DH400F;Pigmentdispersionssyndrom uden glaukom	0			
DH400G;Okulær hypertension sekundært til øjeninflammation	0			
DH400H;Okulær hypertension sekundært til anden øjensygdom	0			
DH400J;Okulær hypertension sekundært til lægemiddel	0			
DH401;Primært åbenvinklet glaukom	0			
DH401A;Normaltryksglaukom	0			
DH402;Primær lukketvinklet glaukom	0			
DH402D;Glaukom sekundært til akut vinkellukning	0			
DH403;Traumatisk glaukom	0			
DH404;Glaukom sekundært til øjeninflammation	0			
DH405;Glaukom sekundært til anden øjensygdom	0			
DH405A;Glaukom sekundært til pseudoexfoliationssyndrom	0			
DH405B;Glaukom sekundært til pigmentdispersionssyndrom	0			
DH405C;Neovaskulært glaukom	0			
DH406;Glaukom forårsaget af lægemiddel	0			
DH408;Anden form for glaukom	0			
DH409;Glaukom UNS	0			
DH42;Grøn stær ved sygdomme klassificeret andetsteds	0			
DH420;Glaukom ved endokrin, ernæringsbetinget eller metabolisk sygdom klassificeret andetsted	0			
DH428;Glaukom ved anden sygdom klassificeret andetsteds	0			
DH43;Forandringer i øjets glaslegeme	0			
DH430;Prolapsus corporis vitrei	0			
DH431;Blødning i øjets glaslegeme	0			

DH432;Krystallinske aflejringer i øjets glaslegeme	0		
DH433;Anden opacitet i øjets glaslegeme	0		
DH433A;Membrani corpus vitreum	0		
DH438;Anden forandring i øjets glaslegeme	0		
DH438C;Degeneratio corporis vitrei	0		
DH438D;Kollaps af øjets glaslegeme	0		
DH439;Forandring i øjets glaslegeme UNS	0		
DH44;Forandringer i øjeæblet	0		
DH440;Purulent endoftalmitis	0		
DH440A;Absces i øjets glaslegeme	0		
DH440B;Panoftalmitis	0		
DH441;Anden form for endoftalmitis	0		
DH441A;Endoftalmitis forårsaget af parasit UNS	0		
DH441B;Ophthalmia sympathica	0		
DH441C;Ophthalmia nodosa	0		
DH441D;Uveitis sympathica	0		
DH441E;Panuveitis	0		
DH442;Degenerativ myopi	0		
DH443;Anden degenerativ forandring i øjeæble	0		
DH443A;Chalcosis	0		
DH443B;Siderosis oculi	0		
DH444;Nedsat intraokulært tryk	0		
DH445;Degenerationsforandring i øjeæble	0		
DH445C;Phthisis bulbi	0		
DH446;Retineret intraokulært magnetisk fremmedlegeme	0		
DH447;Retineret intraokulært ikke-magnetisk fremmedlegeme	0		
DH448;Anden forandring øjeæble	0		
DH448A;Anophthalmus acquisitus	0		
DH448B;Haemophthalmus	0		
DH448C;Luxatio bulbi oculi	0		
DH448D;Ikke-traumatisk orbitalt kompartmentsyndrom	0		
DH449;Forandring i øjeæble UNS	0		

Supplement 2 Nielsen et al. Mortality after paediatric emergency calls for patients with and without pre-existing comorbidity (2023)

DH45;Forandringer i øjets glaslegeme og øjeæble ved sygdomme klassificeret andetsteds	0		
DH450;Blødning i øjets glaslegeme ved sygdom klassificeret andetsteds	0		
DH451;Endoftalmitis ved sygdom klassificeret andetsteds	0		
DH458;Anden forandring i øjets glaslegeme og øjeæble ved sygdom klassificeret andetsteds	0		
DH46;Inflammationer i synsnerve		1	
DH469;Inflammation i synsnerve UNS		1	
DH469A;Neuritis nervi optici retrobulbaris		1	
DH469B;Neuropathia optica non-ischaemica		1	
DH469C;Papillitis retinae		1	
DH47;Andre forandringer i synsnerve og synsbaner		1	
DH470;Forandring i synsnerve IKA		1	
DH470A;Kompression af synsnerven		1	
DH470B;Haemorrhagia vaginae nervi optici		1	
DH470C;Iskæmisk neuropati af synsnerven (AION)		1	
DH471;Ødem af discus nervi optici UNS		1	
DH472;Optikusatrofi		1	
DH472A;Pallor temporalis disci nervi optici		1	
DH473;Anden forandring i synsnervepapillen		1	
DH473A;Druser i synsnervepapillen		1	
DH473B;Pseudopapilødem		1	
DH474;Forandring i chiasma opticum		1	
DH475;Forandring i de øvrige synsbaner		1	
DH475A;Disordo tracti optici		1	
DH475B;Disordo corporis geniculati lateralis		1	
DH475C;Disordo radiationis opticae		1	
DH476;Forandring i synsbarken		1	
DH477;Forandring i synsbane UNS		1	
DH48;Forandringer i synsnerve og synsbaner ved sygdomme klassificeret andetsteds		1	
DH480;Optikusatrofi ved sygdom klassificeret andetsteds		1	
DH481;Retrobulbær neuritis ved sygdom klassificeret andetsteds		1	
DH488;Anden forandring i synsnerve og synsbaner ved sygdom klassificeret andetsteds		1	
DH49;Skelen ved lammelse af øjenmuskler		1	

DH490;Okulomotoriusparese	0			
DH491;Troklearisparese	0			
DH492;Abducensparese	0			
DH493;Total oftalmoplegi	0			
DH494;Progressiv ekstern oftalmoplegi	0			
DH498;Anden skelen forårsaget af lammelse	0			
DH498A;Kearns-Sayres syndrom	0			
DH498B;Ekstern oftalmoplegi UNS	0			
DH499;Skelen ved lammelse UNS	0			
DH50;Andre former for skelen	0			
DH500;Konvergent konkomiterende skelen	0			
DH500A;Esotropia alternans	0			
DH500B;Esotropia monocularis	0			
DH501;Divergent konkomiterende skelen	0			
DH501A;Exotropia monocularis	0			
DH501B;Exotropia alternans	0			
DH502;Vertikal skelen	0			
DH502A;Hypertropia	0			
DH502B;Hypotropia	0			
DH503;Heterotropia intermittens	0			
DH503A;Esotropia intermittens monocularis	0			
DH503B;Esotropia intermittens alternans	0			
DH503C;Exotropia intermittens monocularis	0			
DH503D;Exotropia intermittens alternans	0			
DH504;Anden eller ikke specificeret heterotropi	0			
DH504A;Cyclotropi	0			
DH504B;Heterotropi UNS	0			
DH504C;Hypertropia (findes ikke i SKS)	0			
DH504E;Mikrotropi	0			
DH504F;Monofiksationssyndrom	0			
DH504G;Strabismus concomitans UNS	0			
DH505;Heterofori	0			

DH505A;Esofori	0		
DH505B;Eksofori	0		
DH505C;Hyperphoria alternans	0		
DH506;Strabismus mechanicus	0		
DH506A;Browns skede syndrom	0		
DH506B;Skelen forårsaget af adhærencer	0		
DH508;Anden form for skelen	0		
DH508A;Duanes syndrom	0		
DH509;Skelen UNS	0		
DH51;Andre forstyrrelser i samsynsfunktionen	0		
DH510;Konjugeret blikparese	0		
DH511;Konvergensforstyrrelse	0		
DH512;Internukleær oftalmoplegi	0		
DH518;Anden forstyrrelse i samsynsfunktionen	0		
DH519;Forstyrrelse i samsynsfunktionen UNS	0		
DH52;Forstyrrelser i refraktion og akkommodation	0		
DH520;Hypermetropi	0		
DH521;Myopi	0		
DH522;Astigmatisme	0		
DH522A;Uregelmæssig astigmatisme	0		
DH523;Anisometropi eller aniseikoni	0		
DH523A;Aniseikoni	0		
DH523B;Anisometropi	0		
DH524;Presbyopia	0		
DH525;Akkommodationsforstyrrelse UNS	0		
DH525A;Akkommodationsspasme	0		
DH525B;Ophthalmoplegia interna (partialis) (totalis)	0		
DH526;Anden refraktionsanomali	0		
DH527;Refraktionsanomali UNS	0		
DH53;Synsforstyrrelser	0		
DH530;Amblyopia ex anopsia	0		
DH530A;Amblyopia strabismica	0		

DH530B;Amblyopia deprivationis	0			
DH530C;Amblyopia anisometropica	0			
DH531;Subjektive synsforstyrrelser	0			
DH531A;Asthenopia	0			
DH531B;Dagblindhed	0			
DH531C;Halovisus	0			
DH531D;Metamorphopsia	0			
DH531F;Photophobia	0			
DH531G;Scotoma scintillans	0			
DH531H;Pludseligt synstab	0			
DH532;Dobbeltsyn	0			
DH533;Andre forstyrrelser i det binokulære syn	0			
DH533A;Synsfusion med defekt stereopsi	0			
DH533B;Abnorm retinal korrespondance	0			
DH533C;Suppression af binokulært syn	0			
DH533D;Simultan visuel perception uden fusion	0			
DH534;Synsfeltsdefekter	0			
DH534A;Anopsia quadrantis	0			
DH534B;Forstørret blind plet	0			
DH534C;Hemianopsia homonyma	0			
DH534D;Hemianopsia heteronyma	0			
DH534E;Scotoma annulare	0			
DH534F;Scotoma Bjerrum	0			
DH534G;Scotoma centrale	0			
DH534H;Scotoma arcuatum	0			
DH534I;Generel synsfeltsbegrænsning	0			
DH535;Farvesyns defekter	0			
DH535A;Achromatopsia acquisita	0			
DH535B;Deuteranomalia	0			
DH535C;Deuteranopi	0			
DH535D;Protanomalia	0			
DH535E;Protanopia	0			

DH535F;Tritanomalia	0		
DH535G;Tritanopia	0		
DH535H;Achromatopsia	0		
DH536;Natteblindhed	0		
DH538;Anden synsforstyrrelse	0		
DH539;Synsforstyrrelse UNS	0		
DH54;Blindhed og svagsyn		1	
DH540;Dobbeltsidig blindhed		1	
DH541;Svær synsnedsættelse på begge øjne		1	
DH542;Moderat synsnedsættelse på begge øjne		1	
DH543;Let eller ingen synsnedsættelse på begge øjne		1	
DH544;Blindhed på et øje		1	
DH545;Svær synsnedsættelse på eet øje		1	
DH546;Moderat synsnedsættelse på eet øje		1	
DH547;Synsnedsættelse UNS		1	
DH55;Nystagmus og andre abnorme øjenbevægelser	0		
DH559;Nystagmus UNS	0		
DH559A;Nystagmus deprivatus	0		
DH559B;Nystagmus latens	0		
DH559C;Nystagmus dissociatus	0		
DH559D;Nystagmus congenitus	0		
DH559X;Abnorme øjenbevægelser UNS	0		
DH57;Andre abnorme tilstande i øje og øjenomgivelser	0		
DH570;Abnorm pupilfunktion	0		
DH570A;Adies pupil	0		
DH571;Øjensmerter	0		
DH578;Anden abnorm tilstand i øje eller øjenomgivelser	0		
DH579;Abnorm tilstand i øje eller øjenomgivelser UNS	0		
DH58;Andre sygdomme i øje og øjenomgivelser ved sygdomme klassificeret andetsteds		1	
DH580;Abnorm pupilfunktion ved sygdom klassificeret andetsteds		1	
DH581;Synsforstyrrelse ved sygdom klassificeret andetsteds		1	
DH588;Anden sygdom i øje ved sygdom klassificeret andetsteds		1	

Supplement 2 Nielsen et al. Mortality after paediatric emergency calls for patients with and without pre-existing comorbidity (2023)

DH59;Forandringer i øje og øjenomgivelser efter indgreb IKA		1	
DH590;Bulløs keratopati ved afaki efter kataraktoperation		1	
DH598;Anden sygdom i øje eller øjenomgivelser efter indgreb		1	
DH598A;Korioretinalt ar efter indgreb for nethindeløsning		1	
DH598B;Postoperativ endoftalmi		1	
DH599;Forandring i øje eller øjenomgivelser efter indgreb UNS		1	
DH60;Betændelse i ydre øre	0		
DH600;Absces i ydre øre	0		
DH600A;Absces i ydre øregang	0		
DH601;Flegmone i ydre øre	0		
DH601B;Flegmone i ydre øregang	0		
DH602;Malign ekstern otitis	0		
DH603;Anden form for ekstern infektiøs otitis	0		
DH603A;Badeotitis	0		
DH603B;Otitis externa haemorrhagica	0		
DH603C;Otitis externa diffusa	0		
DH604;Kolesteatom i ydre øre	0		
DH604A;Keratosis obturans meatus acustici externi	0		
DH605;Akut ikke-infektiøs ekstern otitis	0		
DH605B;Akut eksem i ydre øre	0		
DH608;Anden form for ekstern otitis	0		
DH608A;Kronisk ekstern otitis UNS	0		
DH609;Ekstern otitis UNS	0		
DH61;Andre sygdomme i ydre øre	0		
DH610;Perikondritis i ydre øre	0		
DH610A;Chondrodermatitis nodularis helicis	0		
DH611;Ikke-infektiøs sygdom i øremusling	0		
DH611A;Erhvervet misdannelse af øremusling	0		
DH612;Cerumen impactum	0		
DH612A;Cerumen excessivum	0		
DH613;Erhvervet forsnævring af ydre øregang	0		
DH618;Anden sygdom i ydre øre	0		

Supplement 2 Nielsen et al. Mortality after paediatric emergency calls for patients with and without pre-existing comorbidity (2023)

DH618A;Eksostose i ydre øregang	0			
DH619;Sygdom i ydre øre UNS	0			
DH62;Forandringer i ydre øre ved sygdomme klassificeret andetsteds	0			
DH620;Ekstern otitis ved bakteriel sygdom klassificeret andetsteds	0			
DH621;Ekstern otitis ved virussygdom klassificeret andetsteds	0			
DH622;Ekstern otitis ved mykose klassificeret andetsteds	0			
DH623;Ekstern otitis ved anden infektiøs eller parasitær sygdom klassificeret andetsteds	0			
DH624;Ekstern otitis ved anden sygdom klassificeret andetsteds	0			
DH628;Anden forandring i ydre øre ved sygdom klassificeret andetsteds	0			
DH65;Mellemørebetændelse uden pusdannelse	0			
DH650;Akut serøs mellemørebetændelse	0			
DH650A;Otitis media secretorica subacuta	0			
DH651;Anden form for akut mellemørebetændelse uden pusdannelse	0			
DH651C;Akut mellemørebetændelse uden pusdannelse UNS	0			
DH651D;Subakut mellemørebetændelse uden pusdannelse UNS	0			
DH652;Kronisk serøs mellemørebetændelse	0			
DH653;Kronisk mucinøs mellemørebetændelse	0			
DH653B;Otitis media chronica secretoria (findes ikke i SKS)	0			
DH654;Anden form for kronisk mellemørebetændelse uden pusdannelse	0			
DH654C;Kronisk mellemørebetændelse uden pusdannelse UNS	0			
DH659;Mellemørebetændelse uden pusdannelse UNS	0			
DH66;Purulent og ikke specificeret mellemørebetændelse	0			
DH660;Akut purulent mellemørebetændelse	0			
DH660A;Otitis media acuta perforata	0			
DH661;Kronisk purulent tubotympanisk mellemørebetændelse	0			
DH662;Kronisk purulent attikoantral mellemørebetændelse	0			
DH663;Anden form for kronisk purulent mellemørebetændelse	0			
DH664;Purulent mellemørebetændelse UNS	0			
DH669;Mellemørebetændelse UNS	0			
DH67;Mellemørebetændelse ved sygdomme klassificeret andetsteds	0			
DH670;Mellemørebetændelse ved bakteriel sygdom klassificeret andetsteds	0			
DH671;Mellemørebetændelse ved virussygdom klassificeret andetsteds	0			

Supplement 2 Nielsen et al. Mortality after paediatric emergency calls for patients with and without pre-existing comorbidity (2023)

DH678;Mellemørebetændelse ved anden sygdom klassificeret andetsteds	0		
DH68;Betændelse og tillukning af det eustakiske rør	0		
DH680;Otosalpingitis	0		
DH681;Tillukning af det eustakiske rør	0		
DH69;Andre sygdomme i det eustakiske rør	0		
DH690;Tuba auditiva patulosa	0		
DH698;Anden sygdom i det eustakiske rør	0		
DH699;Sygdom i det eustakiske rør UNS	0		
DH70;Mastoiditis og beslægtede sygdomme		1	
DH700;Akut mastoiditis		1	
DH701;Kronisk mastoiditis		1	
DH702;Petrositis		1	
DH708;Anden form for mastoiditis og beslægtede sygdom		1	
DH709;Mastoiditis UNS		1	
DH71;Kolesteatom i mellemøre		1	
DH719;Kolesteatom i mellemøre UNS		1	
DH72;Perforation af trommehinde	0		
DH720;Central perforation af trommehinde	0		
DH721;Atticus-perforation af trommehinde	0		
DH721A;Pars flaccida-perforation af trommehinde	0		
DH722;Anden form for marginal perforation af trommehinde	0		
DH728;Anden form for perforation af trommehinde	0		
DH728A;Total perforation af trommehinde	0		
DH728B;Multiple perforationer af trommehinde	0		
DH729;Perforation af trommehinde UNS	0		
DH73;Anden sygdom i trommehinde	0		
DH730;Akut trommehindebetændelse	0		
DH730A;Bulløs trommehindebetændelse	0		
DH731;Kronisk trommehindebetændelse	0		
DH738;Anden sygdom i trommehinde	0		
DH738A;Trommehinderetraktion	0		
DH739;Sygdom i trommehinde UNS	0		

DH74;Andre sygdomme i mellemøre og processus mastoideus	0
DH740;Tympanosklerose	0
DH741;Sammenvoksninger i mellemøret	0
DH742;Luksation af øreknogler	0
DH743;Anden erhvervet abnormitet i øreknogler	0
DH743A;Ankylosis ossiculae auditus	0
DH743B;Delvis destruktion af øreknogle	0
DH744;Polyp i mellemøre	0
DH748;Anden sygdom i mellemøre eller processus mastoideus	0
DH749;Sygdom i mellemøre eller processus mastoideus UNS	0
DH75;Andre sygdomme i mellemøre og processus mastoideus ved sygdomme klassificeret andet	0
DH750;Mastoiditis ved infektiøs eller parasitær sygdom klassificeret andetsteds	0
DH758;Anden sygdom i mellemøre eller processus mastoideus ved sygdom klassificeret andetste	0
DH80;Otosklerose	0
DH800;Otosklerose som involverer det ovale vindue, ikke-oblitererende	0
DH801;Otosklerose som involverer det ovale vindue, oblitererende	0
DH802;Kokleær otosklerose	0
DH808;Anden form for otosklerose	0
DH809;Otosklerose UNS	0
DH81;Forstyrrelser i ligevægtssansen	0
DH810;Ménières sygdom	0
DH810A;Endolymfatisk hydrops	0
DH811;Vertigo paroxystica benigna	0
DH812;Vestibularis neuritis	0
DH813;Anden form for perifer vertigo	0
DH813A;Lermoyez' syndrom	0
DH813B;Vertigo auralis labyrinthica	0
DH813C;Perifer vertigo UNS	0
DH813D;Otogen vertigo	0
DH814;Central vertigo	0
DH814A;Nystagmus positionalis centralis	0
DH818;Anden forstyrrelse i ligevægtssansen	0

DH819;Forstyrrelse i ligevægtssansen UNS	0			
DH82;Svimmelhed ved sygdomme klassificeret andetsteds	0			
DH829;Vertigo ved sygdom klassificeret andetsteds	0			
DH83;Andre sygdomme i indre øre	0			
DH830;Labyrintitis	0			
DH831;Labyrintfistel	0			
DH832;Labyrintdysfunktion	0			
DH832A;Labyrinthypersensibilitet	0			
DH832B;Labyrinthypofunktion	0			
DH833;Støjskade på indre øre	0			
DH833A;Trauma acustica	0			
DH838;Anden sygdom i indre øre	0			
DH839;Sygdom i indre øre UNS	0			
DH90;Konduktivt og perceptivt høretab	0			
DH900;Dobbeltsidigt konduktivt høretab	0			
DH901;Enkeltsidigt konduktivt høretab med normal hørelse på modsat side	0			
DH902;Konduktivt høretab UNS	0			
DH903;Dobbeltsidigt perceptivt høretab	0			
DH904;Enkeltsidigt perceptivt høretab med normal hørelse på modsat side	0			
DH905;Perceptivt høretab UNS	0			
DH905A;Neuralt høretab UNS	0			
DH905B;Centralt høretab UNS	0			
DH905C;Sensorisk høretab UNS	0			
DH905D;Sensorineuralt høretab UNS	0			
DH905E;Medfødt døvhed UNS	0			
DH906;Dobbeltsidigt blandet konduktivt og perceptivt høretab	0			
DH907;Enkeltsidigt blandet konduktivt og perceptivt høretab med normal hørelse på modsat side	0			
DH908;Blandet konduktivt og perceptivt høretab UNS	0			
DH91;Andre former for høretab	0			
DH910;Ototoksisk høretab	0			
DH911;Aldersbetinget høretab	0			
DH912;Akut idiopatisk høretab	0			

DH912A;Akut høretab UNS	0		
DH913;Døvstumhed IKA	0		
DH918;Andet høretab	0		
DH918A;Medfødt ikke-arveligt høretab UNS	0		
DH918A1;Medfødt ikke-arveligt høretab, asfyksi	0		
DH918A2;Medfødt ikke-arveligt høretab, rhesus-immunisering	0		
DH918A3;Medfødt ikke-arveligt høretab, CMV	0		
DH918A4;Medfødt ikke-arveligt høretab, toksoplasmose	0		
DH918A5;Medfødt ikke-arveligt høretab, rubella	0		
DH919;Høretab UNS	0		
DH919A;Høretab for høje toner UNS	0		
DH919B;Høretab for dybe toner UNS	0		
DH92;Øresmerter og øreflåd	0		
DH920;Øresmerter	0		
DH921;Øreflåd	0		
DH922;Blødning fra øre	0		
DH93;Andre øresygdomme IKA	0		
DH930;Degenerativ eller vaskulær sygdom i øre	0		
DH930A;Degenerativ lidelse i øre	0		
DH930B;Vaskulær lidelse i øre	0		
DH930C;Forbigående iskæmisk døvhed	0		
DH931;Tinnitus	0		
DH932;Anden perceptiv hørelidelse	0		
DH932A;Diplacusis	0		
DH932B;Hyperacusis	0		
DH932C;Temporære skift i høretærskel	0		
DH933;Sygdom i hørenerven	0		
DH933A;Auditiv neuropati	0		
DH933B;Auditory processing disorder (APD)	0		
DH938;Anden øresygdom	0		
DH938A;Sent arveligt høretab	0		
DH939;Sygdom i øre UNS	0		

Supplement 2 Nielsen et al. Mortality after paediatric emergency calls for patients with and without pre-existing comorbidity (2023)

DH94;Andre øresygdomme ved sygdom klassificeret andetsteds	0		
DH940;Auditiv neuropati ved infektiøs eller parasitær sygdom klassificeret andetsteds	0		
DH948;Anden øresygdom ved sygdom klassificeret andetsteds	0		
DH948A;Immunologisk høretab	0		
DH95;Sygdomme i øre og processus mastoideus efter indgreb IKA	0		
DH950;Recidiverende kolesteatom i kavitet efter mastektomi	0		
DH951;Anden følgetilstand efter mastoidektomi	0		
DH951A;Mukøs cyste i kavitet efter mastoidektomi	0		
DH951B;Granulationsvæv i kavitet efter mastoidektomi	0		
DH951C;Kronisk mastoiditis efter mastoidektomi	0		
DH958;Anden sygdom i øre eller processus mastoideus efter indgreb	0		
DH959;Sygdom i øre eller processus mastoideus efter indgreb UNS	0		
DI00;Gigtfeber uden affektion af hjertet		1	
DI009;Gigtfeber uden hjertesygdom		1	
DI009A;Subakut reumatisk feber UNS		1	
DI009B;Akut reumatisk feber UNS		1	
DI01;Akut gigtfeber med affektion af hjertet		1	
DI010;Akut reumatisk perikarditis		1	
DI011;Akut reumatisk endokarditis		1	
DI011A;Akut reumatisk hjerteklapsygdom		1	
DI012;Akut reumatisk myokarditis		1	
DI018;Anden form for akut reumatisk hjertesygdom		1	
DI018A;Akut reumatisk pankarditis		1	
DI019;Akut reumatisk hjertesygdom UNS		1	
DI02;Reumatisk chorea		1	
DI020;Reumatisk chorea med affektion af hjertet		1	
DI020A;Chorea UNS med affektion af hjertet		1	
DI029;Reumatisk chorea uden affektion af hjertet		1	
DI05;Reumatiske affektioner af mitralklappen		1	
DI050;Reumatisk mitralstenose		1	
DI051;Reumatisk mitralinsufficiens		1	
DI052;Reumatisk mitralstenose med insufficiens		1	

Supplement 2 Nielsen et al. Mortality after paediatric emergency calls for patients with and without pre-existing comorbidity (2023)

DI058;Anden form for reumatisk mitralklapaffektion		1	
DI059;Reumatisk mitralklapaffektion UNS		1	
DI06;Reumatiske affektioner af aortaklappen		1	
DI060;Reumatisk aortaklapstenose		1	
DI061;Reumatisk aortaklapinsufficiens		1	
DI062;Reumatisk aortaklapstenose med insufficiens		1	
DI068;Anden form for reumatisk aortaklapaffektion		1	
DI069;Reumatisk aortaklapaffektion UNS		1	
DI07;Reumatiske affektioner af trikuspidalklappen		1	
DI070;Reumatisk trikuspidalstenose		1	
DI071;Reumatisk trikuspidalinsufficiens		1	
DI072;Reumatisk trikuspidalstenose med insufficiens		1	
DI078;Anden form for reumatisk trikuspidalklapaffektion		1	
DI079;Reumatisk trikuspidalklapaffektion UNS		1	
DI08;Affektioner af flere hjerteklapper		1	
DI080;Affektioner af både mitralklap og aortaklap		1	
DI080A;Reumatisk affektion af både mitralklap og aortaklap		1	
DI081;Affektioner af både mitralklap og trikuspidalklap		1	
DI081A;Reumatisk affektion af både mitralklap og trikuspidalklap		1	
DI082;Affektion af både trikuspidalklap og aortaklap		1	
DI082A;Reumatisk affektion af både trikuspidalklap og aortaklap		1	
DI083;Affektioner af både mitralklap, trikuspidalklap og aortaklap		1	
DI083A;Reumatisk affektion af både mitralklap, trikuspidalklap og aortaklap		1	
DI088;Anden form for affektion inddragende flere hjerteklapper		1	
DI089;Affektion af flere hjerteklapper UNS		1	
DI09;Andre reumatiske hjertesygdomme		1	
DI090;Reumatisk myokarditis		1	
DI091;Reumatisk endokarditis uden angivelse af afficeret klap		1	
DI091A;Kronisk reumatisk endokarditis		1	
DI091B;Kronisk reumatisk hjerteklapsygdom		1	
DI092;Kronisk reumatisk perikarditis		1	
DI092A;Reumatisk perikardieadhærence		1	

DI092B;Kronisk reumatisk mediastinoperikarditis		1	
DI092C;Kronisk reumatisk myoperikarditis		1	
DI098;Anden reumatisk hjertesygdom		1	
DI098A;Reumatisk pulmonalklapstenose med insufficiens		1	
DI098B;Reumatisk pulmonalklapinsufficiens		1	
DI098C;Reumatisk pulmonalklapaffektion UNS		1	
DI098E;Reumatisk pulmonalklapstenose		1	
DI099;Reumatisk hjertesygdom UNS		1	
DI099A;Reumatisk hjertesvigt		1	
DI10;Blodtryksforhøjelse af ukendt årsag		1	
DI109;Essentiel hypertension		1	
DI11;Hypertensiv hjertesygdom		1	
DI110;Hypertensiv hjertesygdom med inkompenseret hjertesvigt		1	
DI119;Hypertensiv hjertesygdom uden inkompensation		1	
DI119A;Hypertensiv hjertesygdom UNS		1	
DI12;Hypertensiv nyresygdom		1	
DI120;Hypertensiv nyresygdom med nyresvigt		1	
DI129;Hypertensiv nyresygdom uden nyresvigt		1	
DI129A;Hypertensiv nyresygdom UNS		1	
DI13;Blodtryksforhøjelse med både hjertesygdom og nyresygdom		1	
DI130;Hypertensiv hjertesygdom og nyresygdom med hjertesvigt		1	
DI131;Hypertensiv hjertesygdom og nyresygdom med nyresvigt		1	
DI132;Hypertensiv hjertesygdom og nyresygdom med hjertesvigt og nyresvigt		1	
DI139;Hypertensiv hjertesygdom og nyresygdom UNS		1	
DI15;Blodtryksforhøjelse med kendt årsag		1	
DI150;Renovaskulær hypertension		1	
DI151;Hypertension sekundært til anden nyresygdom		1	
DI152;Hypertension sekundært til endokrin sygdom		1	
DI158;Anden form for sekundær hypertension		1	
DI159;Sekundær hypertension UNS		1	
DI20;Angina pectoris		1	
DI200;Ustabil angina pectoris		1	

DI200B;Klinisk vurderet ustabil angina pectoris		1	
DI200C;Ustabil angina pectoris med dokumenteret iskæmi		1	
DI201;Prinzmetals angina pectoris		1	
DI208;Anden form for angina pectoris		1	
DI208D;Mikrovaskulær angina		1	
DI208E;Stabil angina pectoris		1	
DI208E1;Klinisk vurderet angina pectoris		1	
DI208E2;Angina pectoris med dokumenteret iskæmi		1	
DI209;Angina pectoris UNS		1	
DI21;Akut myokardieinfarkt		1	
DI210;Anteriort akut myokardieinfarkt med Q-taksudvikling		1	
DI210A;Anteriort non-ST-elevations akut myokardieinfarkt med Q-taksudvikling		1	
DI210B;Anteriort ST-elevations akut myokardieinfarkt med Q-taksudvikling		1	
DI211;Inferiort eller posteriort akut myokardieinfarkt med Q-taksudvikling		1	
DI211A;Inferiort eller posteriort non-ST-elevations akut myokardieinfarkt med Q-taksudvikling		1	
DI211B;Inferiort eller posteriort ST-elevations akut myokardieinfarkt med Q-taksudvikling		1	
DI213;ST-elevations akut myokardieinfarkt		1	
DI213A;ST-elevations akut myokardieinfarkt, anteriort		1	
DI213B;ST-elevations akut myokardieinfarkt, inferiort/posteriort		1	
DI213C;ST-elevations akut myokardieinfarkt, grenblok		1	
DI214;Non-ST-elevations akut myokardieinfarkt		1	
DI219;Akut myokardieinfarkt UNS		1	
DI219A;Type 2 myokardieinfarkt		1	
DI23;Komplikationer i efterforløbet af akut myokardieinfarkt		1	
DI230;Hæmoperikardium efter akut myokardieinfarkt		1	
DI231;Atrieseptumruptur efter akut myokardieinfarkt		1	
DI232;Ventrikelseptumruptur efter akut myokardieinfarkt		1	
DI233;Ruptur i hjertevæg uden hæmoperikardium efter akut myokardieinfarkt		1	
DI234;Ruptur af chordae tendineae efter akut myokardieinfarkt		1	
DI235;Papillærmuskelruptur efter akut myokardieinfarkt		1	
DI236;Trombose i atrie eller ventrikel efter akut myokardieinfarkt		1	
DI236A;Trombose i atrieaurikel efter akut myokardieinfarkt		1	

Supplement 2 Nielsen et al. Mortality after paediatric emergency calls for patients with and without pre-existing comorbidity (2023)

DI236B;Trombose i ventrikel akut myokardieinfarkt		1	
DI238;Anden akut komplikation i efterforløbet af akut myokardieinfarkt		1	
DI238A;Perikardieansamling efter akut myokardieinfarkt		1	
DI24;Andre former for akut iskæmisk hjertesygdom		1	
DI240;Koronartrombose uden infarkt		1	
DI240A;Arteriel eller venøs koronaremboli uden infarkt		1	
DI241;Postmyokardieinfarktsyndrom		1	
DI248;Anden form for akut iskæmisk hjertesygdom		1	
DI248A;Insufficientia coronaria		1	
DI249;Akut iskæmisk hjertesygdom UNS		1	
DI25;Kronisk iskæmisk hjertesygdom		1	
DI251;Arteriosklerotisk hjertesygdom		1	
DI252;Gammelt myokardieinfarkt		1	
DI252A;Tidligere myokardieinfarkt (non-Q-tak)		1	
DI252B;Tidligere myokardieinfarkt (Q-tak, anteriort)		1	
DI252C;Tidligere myokardieinfarkt (Q-tak, inferiort/posteriort)		1	
DI253;Hjerteaneurisme		1	
DI254;Koronararterieaneurisme og -dissektion		1	
DI254A;Fistula arteriovenosa coronaria acquisita		1	
DI254B;Spontan Koronararteriedissektion (SKAD)		1	
DI255;Iskæmisk kardiomyopati		1	
DI256;Stum myokardieiskæmi		1	
DI258;Anden form for kronisk iskæmisk hjertesygdom		1	
DI259;Kronisk iskæmisk hjertesygdom UNS		1	
DI26;Blodprop i lunge		1	
DI260;Lungeemboli med akut cor pulmonale		1	
DI260A;Akut cor pulmonale UNS		1	
DI269;Lungeemboli uden akut cor pulmonale		1	
DI269A;Lungeemboli UNS		1	
DI27;Anden pulmonal hjertesygdom		1	
DI270;Essentiel pulmonal arteriel hypertension		1	
DI271;Kyfoskoliotisk hjertesygdom		1	

DI272;Kronisk tromboembolisk pulmonal hypertension		1	
DI278;Anden pulmonal hjertesygdom		1	
DI278A;Eisenmengers syndrom			2
DI279;Pulmonal hjertesygdom UNS		1	
DI279A;Cor pulmonale UNS		1	
DI28;Andre sygdomme i lungekredsløbet		1	
DI280;Pulmonal arteriovenøs fistel		1	
DI281;Aneurisme i lungearterie		1	
DI288;Anden sygdom i lungekredsløbet		1	
DI288A;Ruptur af lungekar		1	
DI288B;Stenose i lungekar		1	
DI289;Sygdom i lungekredsløb UNS		1	
DI30;Akut perikarditis		1	
DI300;Akut idiopatisk perikarditis		1	
DI301;Akut infektiøs perikarditis		1	
DI301A;Perikarditis forårsaget af pneumokokker		1	
DI301B;Purulent perikarditis		1	
DI301C;Perikarditis forårsaget af stafylokokker		1	
DI301D;Perikarditis forårsaget af streptokokker		1	
DI301E;Perikarditis forårsaget af virus		1	
DI308;Anden form for akut perikardititis		1	
DI309;Akut perikarditis UNS		1	
DI31;Andre sygdomme i hjertesækken		1	
DI310;Kronisk adhesiv perikarditis		1	
DI310B;Perikardieadhærencer		1	
DI310C;Kronisk mediastinoperikarditis med adhærencer		1	
DI311;Kronisk konstriktiv perikarditis		1	
DI311A;Forkalkning i perikardiet		1	
DI312;Hæmoperikardium ikke klasssificeret andetsteds		1	
DI313;Non-inflammatorisk ekssudativ perikarditis		1	
DI313A;Kyloperikardium		1	
DI318;Anden sygdom i perikardiet		1	

DI318A;Fokale perikardieadhæsioner		1	
DI318B;Kronisk (recidiverende) perikarditis UNS		1	
DI319;Sygdom i perikardiet UNS		1	
DI319A;Hjertetamponade		1	
DI32;Perikarditis ved sygdomme klassificeret andetsteds		1	
DI320;Perikarditis ved bakteriel sygdom klassificeret andetsteds		1	
DI321;Perikarditis ved anden infektiøs eller parasitær sygdom klassificeret andetsteds		1	
DI328;Perikarditis ved anden sygdom klassificeret andetsteds		1	
DI33;Akut og subakut endokarditis		1	
DI330;Akut eller subakut infektiøs endokarditis		1	
DI339;Akut endokarditis UNS		1	
DI34;Ikke-reumatiske sygdomme i mitralklappen		1	
DI340;Mitralinsufficiens		1	
DI341;Mitralklapsprolaps		1	
DI342;Ikke-reumatisk mitralstenose		1	
DI348;Anden sygdom i mitralklap		1	
DI348A;Sekundær mitralinsufficiens		1	
DI349;Ikke-reumatisk mitralklaplidelse UNS		1	
DI35;Ikke-reumatiske sygdomme i aortaklappen		1	
DI350;Aortastenose		1	
DI351;Aortainsufficiens		1	
DI352;Aortastenose med insufficiens		1	
DI358;Anden form for aortaklapsygdom		1	
DI358A;Aortaklapsklerose		1	
DI359;Aortaklapsygdom UNS		1	
DI36;Ikke-reumatiske sygdomme i trikuspidalklappen		1	
DI360;Ikke-reumatisk trikuspidalstenose		1	
DI361;Ikke-reumatisk trikuspidalinsufficiens		1	
DI362;Ikke-reumatisk trikuspidalklapstenose med insufficiens		1	
DI368;Anden form for ikke-reumatisk trikuspidalklapsygdom		1	
DI368A;Sekundær trikuspidalinsufficiens		1	
DI369;Ikke-reumatisk trikuspidalklapsygdom UNS		1	

DI37;Sygdomme i pulmonalklappen		1	
DI370;Pulmonalklapstenose		1	
DI371;Pulmonalklapinsufficiens		1	
DI372;Pulmonalklapstenose med insufficiens		1	
DI378;Anden form for pulmonalklapsygdom		1	
DI379;Pulmonalklapsygdom UNS		1	
DI38;Endokarditis uden angivelse klapaffektion		1	
DI389;Endokarditis UNS		1	
DI39;Endokarditis og lidelser i hjerteklapper ved sygdomme klassificeret andetsteds		1	
DI390;Affektion af mitralklappen ved sygdom klassificeret andetsteds		1	
DI391;Affektion af aortaklappen ved sygdom klassificeret andetsteds		1	
DI392;Affektion af trikuspidalklappen ved sygdom klassificeret andetsteds		1	
DI393;Affektion af pulmonalklappen ved sygdom klassificeret andetsteds		1	
DI394;Affektion af flere hjerteklapper ved sygdom klassificeret andetsteds		1	
DI398;Endokarditis UNS ved sygdom klassificeret andetsteds		1	
DI40;Akut myokarditis		1	
DI400;Infektiøs myokarditis		1	
DI401;Idiopatisk myokarditis		1	
DI408;Anden form for akut myokarditis		1	
DI408A;Toxisk myokarditis		1	
DI409;Akut myokarditis UNS		1	
DI41;Myokarditis ved sygdom klassificeret andetsteds		1	
DI410;Myokarditis ved bakteriel sygdom klassificeret andetsteds		1	
DI411;Myokarditis ved viral sygdom klassificeret andetsteds		1	
DI412;Myokarditis ved anden infektiøs eller parasitær sygdom klassificeret andetsteds		1	
DI418;Myokarditis ved anden sygdom klassificeret andetsteds		1	
DI42;Kardiomyopati		1	
DI420;Dilateret kardiomyopati		1	
DI421;Obstruktiv hypertrofisk kardiomyopati		1	
DI421A;Hypertrofisk subaortastenose		1	
DI422;Hypertrofisk kardiomyopati, non-obstruktiv		1	
DI423;Endomyocarditis eosinophilica		1	

Supplement 2 Nielsen et al. Mortality after paediatric emergency calls for patients with and without pre-existing comorbidity (2023)

DI423A;Fibrosis endomyocardii tropica		1	
DI423B;Löfflers endokarditis		1	
DI424;Fibroelastosis endocardii		1	
DI424A;Medfødt kardiomyopati		1	
DI425;Anden form for restriktiv kardiomyopati			2
DI426;Alkoholisk kardiomyopati		1	
DI427;Kardiomyopati forårsaget af lægemiddel eller andet agens		1	
DI428;Anden form for kardiomyopati		1	
DI428A;Arytmogen højre ventrikel dysplasi (ARVD)		1	
DI428B;Takotsubo kardiomyopati		1	
DI429;Kardiomyopati UNS		1	
DI43;Kardiomyopati ved sygdom klassificeret andetsteds		1	
DI430;Kardiomyopati ved infektiøs eller parasitær sygdom klassificeret andetsteds		1	
DI431;Kardiomyopati ved metabolisk sygdom klassificeret andetsteds		1	
DI432;Kardiomyopati ved ernæringssygdom klassificeret andetsteds		1	
DI438;Kardiomyopati ved anden sygdom klassificeret andetsteds		1	
DI44;Atrioventrikulært blok og venstresidigt grenblok		1	
DI440;1° Forlænget AV-overledning		1	
DI441;2° Intermitterende AV-overledning		1	
DI441B;2° intermitterende AV-overledning Type 1		1	
DI441C;2° intermitterende AV-overledning Type 2		1	
DI441D;2:1 AV overledning (blok)		1	
DI441E;Avanceret AV blok med 2 eller flere konsekutive manglende overledninger		1	
DI442;3° Ophævet AV-overledning (AV-blok)		1	
DI442A;Paroksystisk ophævet overledning (AV blok)		1	
DI443;Atrioventrikulært hjerteblok UNS		1	
DI443A;Anden form for atrioventrikulært hjerteblok		1	
DI444;Venstresidigt anteriort fascikelblok		1	
DI445;Venstresidigt posteriort fascikelblok		1	
DI446;Andet eller ikke specificeret venstresidigt grenblok		1	
DI446A;Venstresidigt atrioventrikulært hemiblok UNS		1	
DI446B;Atrioventrikulært hemiblok UNS		1	

DI447;Venstresidigt grenblok UNS		1	
DI45;Andre ledningsforstyrrelser i hjertet		1	
DI450;Højresidigt fascikelblok		1	
DI451;Anden eller ikke specificeret form for højresidigt grenblok		1	
DI451A;Højresidigt atrioventrikulært grenblok		1	
DI452;Bifascikulært blok		1	
DI453;Trifascikulært blok		1	
DI454;Intraventrikulært blok UNS		1	
DI455;Andet hjerteblok		1	
DI455A;Sinusbradykardi		1	
DI455B;Sinuatrialt blok		1	
DI455C;Sinusarrest		1	
DI455G;Atriventrikulær (nodal) eskapaderytme		1	
DI455H;Accelereret atrioventrikulær (nodal) eskapaderytme		1	
DI455K;Ventrikulær eskapaderytme		1	
DI455L;Accelereret idioventrikulær rytme		1	
DI456;Præexcitationssyndrom		1	
DI456A;Accessorisk ledningsbundt		1	
DI456AA;Retrogradt ledende accessorisk ledningsbundt		1	
DI456B;Lown-Ganong-Levines syndrom		1	
DI456C;Wolff-Parkinson-White konfiguration med delta-tak uden takykardi		1	
DI456D;Wolff-Parkinson-Whites syndrom med delta-tak		1	
DI456DA;Wolff-Parkinson-Whites syndrom med orthodrom takykardi		1	
DI456DB;Wolff-Parkinson-Whites syndrom med antidrom takykardi		1	
DI456E;Skjult Wolff-Parkinson-Whites syndrom		1	
DI458;Anden ledningsforstyrrelse i hjertet		1	
DI458M;Supraventrikulær takykardi via Mahaimfibre		1	
DI458N;Permanent reciprokerende junktional takykardi (PJRT)		1	
DI459;Ledningsforstyrrelse i hjertet UNS		1	
DI459A;Adams-Stokes' syndrom		1	
DI46;Hjertestop		1	
DI460;Hjertestop med vellykket genoplivning		1	

Supplement 2 Nielsen et al. Mortality after paediatric emergency calls for patients with and without pre-existing comorbidity (2023)

DI461;Pludselig hjertedød			2
DI469;Hjertestop UNS		1	
DI47;Anfaldsvis takykardi		1	
DI470;Ventrikulær takykardi (reentry)		1	
DI470A;Ventrikulær takykardi med iskæmisk hjertesygdom		1	
DI470B;Arytmogen højre ventrikel-takykardi		1	
DI470C;Ventrikulær takykardi med anden strukturel hjertesygdom		1	
DI470D;Idiopatisk (fascikulær) venstre-ventrikel takykardi		1	
DI470H;Idiopatisk højre ventrikel-takykardi		1	
DI470HA;Paroxysmatisk ventrikulær takykardi fra højre ventrikels udløbsdel		1	
DI470HB;Repetitiv højre ventrikel-takykardi		1	
DI471;Supraventrikulær takykardi med smalle QRS-komplekser		1	
DI471E;Sinusarytmi		1	
DI471EA;Fasisk sinusarytmi		1	
DI471EB;Non-fasisk sinusarytmi		1	
DI471F;Sinustakykardi		1	
DI471FA;Fysiologisk sinustakykardi		1	
DI471G;Sinusknude reentry takykardi		1	
DI471H;Uhensigtsmæssig sinustakykardi		1	
DI471J;Postural ortostatisk takykardisyndrom (POTS)		1	
DI471L;Atrial takykardi		1	
DI471LA;Atrial takykardi med intermitterende overledning		1	
DI471M;Multifokal atrial takykardi		1	
DI471N;Postoperativ reentry atrial takykardi		1	
DI471P;AV junktional takykardi		1	
DI471PA;Fokal junktional takykardi		1	
DI471PB;Non-paroksysmatisk junktional takykardi		1	
DI471R;AV nodal reentry takykardi (AVNRT) UNS		1	
DI471RA;Almindelig form af AV nodal reentry takykardi (slow-fast)		1	
DI471RB;Sjælden form af AV nodal reentry takykardi (fast-slow)		1	
DI471RC;AV nodal reentry takykardi (slow-slow)		1	
DI471X;Supraventrikulær takykardi med brede QRS-komplekser		1	

Supplement 2 Nielsen et al. Mortality after paediatric emergency calls for patients with and without pre-existing comorbidity (2023)

DI472;Ventrikulær takykardi		1	
DI472A;Ventrikulær takykardi UNS		1	
DI472B;Ventrikulær takykardi uden påviselig strukturel hjertesygdom		1	
DI472D;Torsade de pointes ventrikulær takykardi		1	
DI472E;Medfødt langt QT-syndrom		1	
DI472EA;Romano-Ward medfødt langt QT-syndrom		1	
DI472EB;Jervell-Lange-Nielsens medfødt langt QT-syndrom		1	
DI472F;Erhvervet langt QT-syndrom		1	
DI472FA;Langt QT-syndrom forårsaget af lægemiddel		1	
DI472H;Polymorf ventrikulær takykardi uden langt QT-interval		1	
DI472L;Brugadamønster i EKG		1	
DI472LA;Brugadamønster i EKG, type 1		1	
DI472LB;Brugadamønster i EKG, type 2		1	
DI472LC;Brugadamønster i EKG, type 3		1	
DI472M;Brugadas syndrom		1	
DI472N;Kort QT-interval i EKG		1	
DI472NA;Kort QT-syndrom		1	
DI478;Anden takykardi		1	
DI478A;Takykardi med brede QRS-komplekser med ukendt mekanisme		1	
DI479;Paroksysmatisk takykardi UNS		1	
DI48;Atrieflagren og atrieflimren		1	
DI480;Paroksysmatisk atrieflimren		1	
DI481;Persisterende atrieflimren		1	
DI482;Kronisk atrieflimren		1	
DI483;Typisk atrieflagren		1	
DI484;Atypisk atrieflagren		1	
DI489;Atrieflagren eller atrieflimren UNS		1	
DI49;Andre hjerterytmeforstyrrelser		1	
DI490;Ventrikelflagren eller ventrikelflimren		1	
DI490A;Ventrikelflagren		1	
DI490B;Ventrikelflimren		1	
DI490BA;Idiopatisk ventrikelflimren		1	

Supplement 2 Nielsen et al. Mortality after paediatric emergency calls for patients with and without pre-existing comorbidity (2023)

DI491;Supraventrikulære ekstrasystoler	0		
DI491A;Sinusknude-ekstrasystoler	0		
DI491B;Atriale ekstrasystoler	0		
DI491C;Overledte atriale ekstrasystoler	0		
DI491D;Ikke-overledte atriale ekstrasystoler	0		
DI491E;Multifokale atriale ekstrasystoler	0		
DI492;AV junktionale ekstrasystoler	0		
DI493;Ventrikulære ekstrasystoler	0		
DI493A;Ventrikulære ekstrasystoler fra højre ventrikels udløbsdel	0		
DI493B;Ventrikulære ekstrasystoler fra venstre ventrikel	0		
DI493C;Ventrikulære ekstrasystoler fra ukendt sted	0		
DI494;Anden form for ekstrasystoli	0		
DI494A;Ekstrasystoli UNS	0		
DI495;Syg sinusknude-syndrom		1	
DI495B;Takykardi-bradykardi syndrom		1	
DI498;Anden hjerterytmeforstyrrelse		1	
DI499;Hjerterytmeforstyrrelse UNS		1	
DI499A;Søvnrelateret hjertearytmi		1	
DI50;Hjertesvigt		1	
DI500;Kronisk hjerteinsufficiens		1	
DI500A;Højresidig hjerteinsufficiens		1	
DI501;Venstresidig hjerteinsufficiens		1	
DI501A;Asthma cardiale		1	
DI501B;Kardielt lungeødem		1	
DI501C;Kardiel lungestase		1	
DI501D;Biventrikulær hjerteinsufficiens		1	
DI509;Hjertesvigt UNS		1	
DI51;Dårligt definerede hjertesygdomme og komplikationer til hjertesygdomme		1	
DI510;Erhvervet defekt i hjerteskillevæg		1	
DI510A;Erhvervet ventrikelseptumdefekt		1	
DI510B;Erhvervet atrieseptumdefekt		1	
DI511;Ruptur af chordae tendineae IKA		1	

DI511A;Mitralklapinsufficiens ved chordaruptur		1	
DI512;Papillærmuskelruptur i hjertet IKA		1	
DI513;Intrakardiel trombose IKA		1	
DI513A;Atrietrombose i hjertet IKA		1	
DI513B;Aurikeltrombose i hjertet IKA		1	
DI513C;Ventrikeltrombose i hjertet IKA		1	
DI514;Myokarditis UNS		1	
DI514A;Myokardiefibrose		1	
DI514B;Kronisk interstitiel myokarditis		1	
DI515;Myokardiedegeneration		1	
DI515A;Degeneratio myocardii lipoidica		1	
DI515B;Degeneratio myocardii senilis		1	
DI516;Kardiovaskulær sygdom UNS		1	
DI517;Kardiomegali		1	
DI517A;Dilatatio cordis		1	
DI517B;Dilatatio ventriculorum cordis		1	
DI517C;Hjertehypertrofi		1	
DI518;Anden dårligt defineret hjertesygdom		1	
DI518A;Akut karditis		1	
DI518B;Kronisk karditis		1	
DI519;Hjertesygdom UNS		1	
DI52;Andre hjertesygdomme ved sygdomme klassificeret andetsteds		1	
DI520;Anden hjertesygdom ved bakteriel sygdom klassificeret andetsteds		1	
DI521;Anden hjertesygdom ved anden infektiøs eller parasitær sygdom klassificeret andetsteds		1	
DI521C;Cor pulmonale ved sygdom klassificeret andetsteds		1	
DI528;Anden form for hjertesygdom ved anden sygdom klassificeret andetsteds		1	
DI60;Subaraknoidalblødning		1	
DI600;Subaraknoidalblødning fra karotissifonen eller bifurkaturen		1	
DI601;Subaraknoidalblødning fra arteria cerebri media		1	
DI602;Subaraknoidalblødning fra arteria communicans anterior		1	
DI603;Subaraknoidalblødning fra arteria communicans posterior		1	
DI604;Subaraknoidalblødning fra arteria basilaris		1	

DI605;Subaraknoidalblødning fra arteria vertebralis		1	
DI606;Subaraknoidalblødning fra anden intrakraniel arterie		1	
DI606A;Subaraknoidalblødning fra arteria cerebri posterior		1	
DI606B;Subaraknoidalblødning fra arteria cerebri anterior		1	
DI606C;Subaraknoidalblødning fra flere intrakranielle arterier		1	
DI607;Subaraknoidalblødning fra intrakraniel arterie UNS		1	
DI607A;Bristet medfødt intrakranielt sakkulært aneurisme		1	
DI608;Anden form for subaraknoidalblødning		1	
DI609;Subaraknoidalblødning UNS		1	
DI609A;Bristet (medfødt) intrakranielt aneurisme UNS		1	
DI61;Hjerneblødning		1	
DI610;Subkortikal blødning i hjernehemisfære		1	
DI610A;Dybtliggende blødning i hjernehemisfære		1	
DI611;Kortikal blødning i hjernehemisfære		1	
DI611A;Blødning i hjernens overflade		1	
DI611B;Haemorrhagia lobi cerebri		1	
DI612;Intracerebral blødning i hjernehemisfære UNS		1	
DI613;Blødning i hjernestammen		1	
DI614;Blødning i lillehjernen		1	
DI615;Blødning i hjerneventrikel		1	
DI616;Blødning flere steder i hjernen		1	
DI618;Anden form for hjerneblødning		1	
DI619;Hjerneblødning UNS		1	
DI62;Andre ikke-traumatiske intrakranielle blødninger		1	
DI620;Akut ikke-traumatisk subdural blødning		1	
DI621;Ikke-traumatisk epidural blødning		1	
DI629;Ikke-traumatisk intrakraniel blødning UNS		1	
DI63;Hjerneinfarkt		1	
DI630;Hjerneinfarkt forårsaget af trombose i præcerebral arterie		1	
DI631;Hjerneinfarkt forårsaget af emboli i præcerebral arterie		1	
DI632;Hjerneinfarkt forårsaget af tillukning eller stenose i præcerebral arterie UNS		1	
DI633;Hjerneinfarkt forårsaget af trombose i cerebral arterie		1	

DI634;Hjerneinfarkt forårsaget af emboli i cerebral arterie		1	
DI635;Hjerneinfarkt forårsaget af tillukning eller stenose i cerebral arterie UNS		1	
DI636;Hjerneinfarkt forårsaget af ikke-pyogen cerebral venøs trombose		1	
DI638;Anden form for hjerneinfarkt		1	
DI639;Hjerneinfarkt UNS		1	
DI64;Slagtilfælde uden oplysning om blødning eller infarkt		1	
DI649;Apoplexia cerebri UNS		1	
DI65;Okklusioner og stenoser af præcerebrale arterier uden hjerneinfarkt		1	
DI650;Okklusion eller stenose af arteria vertebralis uden hjerneinfarkt		1	
DI650A;Okklusion af arteria vertebralis uden hjerneinfarkt		1	
DI650B;Stenose af arteria vertebralis uden hjerneinfarkt		1	
DI651;Okklusion eller stenose af arteria basilaris uden hjerneinfarkt		1	
DI651A;Okklusion af arteria basilaris uden hjerneinfarkt		1	
DI651B;Stenose af arteria basilaris uden hjerneinfarkt		1	
DI652;Okklusion eller stenose af arteria carotis uden hjerneinfarkt		1	
DI652A;Okklusion af arteria carotis uden hjerneinfarkt		1	
DI652B;Stenose af arteria carotis uden hjerneinfarkt		1	
DI653;Okklusion eller stenose af flere eller bilaterale præcerebrale arterier uden hjerneinfarkt		1	
DI653A;Okklusion af bilaterale præcerebrale arterier uden hjerneinfarkt		1	
DI653B;Okklusion af flere præcerebrale arterier uden hjerneinfarkt		1	
DI653C;Stenose flere præcerebrale arterier uden hjerneinfarkt		1	
DI653D;Stenose af bilaterale præcerebrale arterier uden hjerneinfarkt		1	
DI658;Okklusion eller stenose af anden præcerebral arterie uden hjerneinfarkt		1	
DI659;Okklusion eller stenose af præcerebral arterie uden hjerneinfarkt UNS		1	
DI66;Okklusioner og stenoser af cerebrale arterier uden hjerneinfarkt		1	
DI660;Okklusion eller stenose af arteria cerebri media uden hjerneinfarkt		1	
DI660A;Okklusion af arteria cerebri media uden hjerneinfarkt		1	
DI660B;Stenose af arteria cerebri media uden hjerneinfarkt		1	
DI661;Okklusion eller stenose af arteria cerebri anterior uden hjerneinfarkt		1	
DI661A;Okklusion af arteria cerebri anterior uden hjerneinfarkt		1	
DI661B;Stenose af arteria cerebri anterior uden hjerneinfarkt		1	
DI662;Okklusion eller stenose af arteria cerebri posterior uden hjerneinfarkt		1	

DI662A;Okklusion af arteria cerebri posterior uden hjerneinfarkt		1	
DI662B;Stenose af arteria cerebri posterior uden hjerneinfarkt		1	
DI663;Okklusion eller stenose af cerebellar arterie uden hjerneinfarkt		1	
DI663A;Okklusion af cerebellar arterie uden hjerneinfarkt		1	
DI663B;Stenose af cerebellar arterie uden hjerneinfarkt		1	
DI664;Okklusion eller stenose af flere eller bilaterale cerebrale arterier uden hjerneinfarkt		1	
DI664A;Okklusion af bilaterale cerebrale arterier uden hjerneinfarkt		1	
DI664B;Okklusion af flere cerebrale arterier uden hjerneinfarkt		1	
DI664C;Stenose af bilaterale cerebrale arterier uden hjerneinfarkt		1	
DI664D;Stenose af flere cerebrale arterier uden hjerneinfarkt		1	
DI668;Okklusion eller stenose af andre cerebrale arterier uden hjerneinfarkt		1	
DI668A;Okklusion af en eller flere af arteriae perforantes cerebri uden hjerneinfarkt		1	
DI669;Okklusion eller stenose af cerebrale arterie UNS uden hjerneinfarkt		1	
DI67;Andre cerebrovaskulære sygdomme		1	
DI670;Dissektion af cerebral arterie uden ruptur		1	
DI671;Cerebralt aneurisme uden ruptur		1	
DI671A;Erhvervet cerebral arteriovenøs fistel		1	
DI672;Cerebral aterosklerose		1	
DI672A;Atheroma arteriae cerebri		1	
DI673;Progressiv vaskulær leukoencefalopati		1	
DI673A;Binswangers sygdom		1	
DI674;Hypertensiv encefalopati		1	
DI675;Moyamoya-sygdom		1	
DI676;Ikke-pyogen intrakraniel venøs trombose		1	
DI676A;Ikke-pyogen trombose i sinus venosi cerebri		1	
DI677;Cerebral arteritis IKA		1	
DI677A;Primær cerebral vaskulitis		1	
DI678;Anden cerebrovaskulær sygdom		1	
DI678A;Akut cerebrovaskulær insufficiens		1	
DI678B;Cerebral (kronisk) iskæmi		1	
DI679;Cerebrovaskulær sygdom UNS		1	
DI68;Karforandringer i hjernen ved sygdomme klassificeret andetsteds		1	

Supplement 2 Nielsen et al. Mortality after paediatric emergency calls for patients with and without pre-existing comorbidity (2023)

DI680;Cerebral amyloid angiopati		1	
DI681;Cerebral arteritis ved infektiøs eller parasitær sygdom klassificeret andetsteds		1	
DI682;Cerebral arteritis ved anden sygdom klassificeret andetsteds		1	
DI688;Anden karforandring i hjernen ved sygdom klassificeret andetsteds		1	
DI69;Senfølger efter karsygdomme i hjernen		1	
DI690;Senfølge efter tidligere subaraknoidalblødning		1	
DI691;Senfølge efter tidligere hjerneblødning		1	
DI692;Senfølge efter tidligere anden form for ikke-traumatisk intrakraniel blødning		1	
DI693;Senfølge efter tidligere hjerneinfarkt		1	
DI694;Senfølge efter tidligere apoplexia cerebri		1	
DI698;Senfølge efter tidligere anden eller ikke nærmere specificeret cerebrovaskulær sygdom		1	
DI70;Åreforkalkning		1	
DI700;Aterosklerose i aorta		1	
DI701;Aterosklerose i nyrearterie		1	
DI702;Aterosklerose i arterie i underekstremitet		1	
DI702A;Aterosklerotisk gangræn		1	
DI702B;Mönckebergs mediasklerose		1	
DI708;Aterosklerose i anden arterie		1	
DI708A;Aterosklerotisk retinopati		1	
DI709;Aterosklerose UNS		1	
DI71;Aorta-aneurisme og aortadissektion		1	
DI710;Aortadissektion UNS		1	
DI710A;Aortadissektion, type A		1	
DI710B;Aortadissektion, type B		1	
DI711;Rumperet torakalt aorta-aneurisme		1	
DI712;Torakalt aorta-aneurisme uden ruptur		1	
DI713;Rumperet abdominalt aorta-aneurisme		1	
DI714;Abdominalt aorta-aneurisme uden ruptur		1	
DI715;Rumperet torakoabdominalt aorta-aneurisme		1	
DI716;Torakoabdominalt aorta-aneurisme uden ruptur		1	
DI718;Rumperet aorta-aneurisme UNS		1	
DI719;Aorta-aneurisme UNS uden ruptur		1	

DI719A;Dilateret aorta		1	
DI719B;Hyalin nekrose i aorta		1	
DI72;Andre aneurismer		1	
DI720;Aneurisme på arteria carotis		1	
DI721;Aneurisme på arterie i overekstremitet		1	
DI722;Aneurisme på nyrearterie		1	
DI723;Aneurisme på arteria iliaca		1	
DI724;Aneurisme på arterie i underekstremitet		1	
DI725;Aneurisme i anden præcerebral arterie		1	
DI726;Aneurisme på arteria vertebralis		1	
DI728;Aneurisme med anden lokalisation		1	
DI729;Aneurisme UNS		1	
DI73;Andre sygdomme i perifere kar		1	
DI730;Raynauds syndrom		1	
DI731;Thromboangiitis obliterans		1	
DI738;Anden sygdom i perifere kar		1	
DI738A;Akrocyanose		1	
DI738B;Akroparæstesi		1	
DI738C;Erytrocyanose		1	
DI738D;Erytromelalgi		1	
DI738E;Pseudokaposi		1	
DI739;Sygdom i perifere kar UNS		1	
DI739A;Claudicatio intermittens		1	
DI739B;Arteriespasme UNS		1	
DI739C;Iskæmiske hvilesmerter i underekstremitet		1	
DI74;Emboli og trombose i arterier		1	
DI740;Emboli eller trombose i aorta abdominalis		1	
DI740A;Bifurcatura aortae-syndrom		1	
DI740B;Embolia aortae abdominalis		1	
DI740C;Leriches syndrom		1	
DI740D;Thrombosis aortae abdominalis		1	
DI741;Emboli eller trombose i aorta med anden eller ikke specificeret lokalisation		1	

DI741A;Emboli i aorta UNS		1	
DI741B;Trombose i aorta UNS		1	
DI742;Emboli eller trombose i arterie i overekstremitet		1	
DI742A;Emboli i arterie i overekstremitet		1	
DI742B;Trombose i arterie i overekstremitet		1	
DI743;Emboli eller trombose i arterie i underekstremitet		1	
DI743A;Emboli i arterie i underekstremitet		1	
DI743B;Trombose i arterie i underekstremitet		1	
DI744;Emboli eller trombose i arterie i ekstremitet UNS		1	
DI744A;Emboli i arterie i ekstremitet UNS		1	
DI744B;Trombose i arterie i ekstremitet UNS		1	
DI744C;Embolia arteriae periphericae		1	
DI745;Emboli eller trombose i arteria iliaca		1	
DI745A;Emboli i arteria iliaca		1	
DI745B;Trombose i arteria iliaca		1	
DI748;Emboli eller trombose i anden arterie		1	
DI749;Emboli eller trombose i arterie UNS		1	
DI77;Andre sygdomme i arterier og arterioler		1	
DI770;Erhvervet arteriovenøs fistel		1	
DI770A;Varikøst aneurisme		1	
DI770B;Erhvervet spinal arteriovenøs malformation		1	
DI771;Arteriestriktur		1	
DI772;Arterieruptur		1	
DI772A;Erosio arteriae		1	
DI772B;Fistula arteriae		1	
DI773;Fibromuskulær hyperplasi		1	
DI774;Arteria coeliaca-kompressionssyndrom		1	
DI775;Arterienekrose		1	
DI776;Arteritis UNS		1	
DI776A;Endarteritis UNS		1	
DI778;Anden sygdom i arterier og arterioler		1	
DI778A;Malign atrofisk papulose		1	

DI779;Sygdom i arterier og arterioler UNS		1	
DI78;Sygdomme i kapillærer		1	
DI780;Arvelig hæmoragisk telangiektasi		1	
DI781;Ikke-neoplastisk nævus		1	
DI781A;Naevus araneus		1	
DI781B;Naevus senilis		1	
DI781D;Naevus anaemicus		1	
DI788;Anden sygdom i kapillærer		1	
DI788A;Sekundær telangiektasi		1	
DI788B;Telangiektasi UNS		1	
DI788C;Telangiectasia actinica		1	
DI788D;Telangiectasia cosmetica		1	
DI789;Kapillærsygdom UNS		1	
DI79;Forandringer i arterier, arterioler og kapillærer ved sygdomme klassificeret andetsteds		1	
DI790;Aorta-aneurisme ved sygdom klassificeret andetsteds		1	
DI791;Aortitis ved sygdom klassificeret andetsteds		1	
DI792;Perifer angiopati ved sygdom klassificeret andetsteds		1	
DI798;Anden forandring i arterier, arterioler og kapillærer ved sygdom klassificeret andetsteds		1	
DI80;Årebetændelse		1	
DI800;Superficiel flebitis eller tromboflebitis i underekstremitet		1	
DI800A;Superficiel flebitis i underekstremitet		1	
DI800B;Superficiel tromboflebitis i underekstremitet		1	
DI801;Flebitis eller tromboflebitis i vena femoralis		1	
DI801A;Flebitis i vena femoralis		1	
DI801B;Tromboflebitis i vena femoralis		1	
DI802;Dyb flebitis eller tromboflebitis i anden vene i underekstremitet		1	
DI802A;Dyb flebitis i anden vene i underekstremitet		1	
DI802B;Dyb tromboflebitis i anden vene i underekstremitet		1	
DI803;Flebitis eller tromboflebitis i underekstremitet UNS		1	
DI803A;Venøs emboli i underekstremitet UNS		1	
DI803B;Flebitis i underekstremitet UNS		1	
DI803C;Dyb flebitis i underekstremitet UNS		1	

DI803D;Tromboflebitis i underekstremitet UNS		1	
DI803E;Dyb tromboflebitis i underekstremitet UNS		1	
DI803F;Venøs trombose i underekstremitet UNS		1	
DI808;Flebitis eller tromboflebitis med anden lokalisation		1	
DI808A;Flebitis med anden lokalisation		1	
DI808B;Tromboflebitis med anden lokalisation		1	
DI808C;Mondors sygdom		1	
DI808D;Flebitis eller tromboflebitis i vena jugularis		1	
DI809;Flebitis eller tromboflebitis UNS		1	
DI81;Trombose i portåren		1	
DI819;Trombose i vena porta		1	
DI82;Anden venøs emboli og trombose		1	
DI820;Budd-Chiaris syndrom		1	
DI821;Thrombophlebitis migrans		1	
DI822;Emboli eller trombose i vena cava		1	
DI822A;Emboli i vena cava		1	
DI822B;Trombose i vena cava		1	
DI823;Emboli eller trombose i vena renalis		1	
DI823A;Emboli i vena renalis		1	
DI823B;Trombose i vena renalis		1	
DI828;Emboli eller trombose i anden vene		1	
DI829;Emboli eller trombose i vene UNS		1	
DI829A;Emboli i vene UNS		1	
DI829B;Trombose i vene UNS		1	
DI83;Varicer i underekstremiteter		1	
DI830;Varicer i underekstremitet med ulcus		1	
DI830A;Varicøst ulcus på underekstremitet		1	
DI830B;Ulcus cruris post-thromboticum		1	
DI831;Varicer i underekstremitet med eksem		1	
DI831A;Stasedermatitis		1	
DI832;Varicer i underekstremitet med både ulcus og eksem		1	
DI839;Varicer i underekstremitet uden ulcus og eksem		1	

DI839A;Varicer UNS		1	
DI839B;Flebektasi		1	
DI842;Interne hæmorroider uden komplikation (findes ikke i SKS)		1	
DI849;Hæmorroider UNS uden komplikation (findes ikke i SKS)		1	
DI85;Varicer i spiserøret		1	
DI850;Øsofagusvaricer med blødning		1	
DI859;Øsofagusvaricer uden blødning		1	
DI86;Varicer med anden lokalisation		1	
DI860;Varices sublinguales		1	
DI861;Varicer i scrotum		1	
DI862;Varices pelvis		1	
DI863;Varices vulvae		1	
DI864;Varicer i mavesækken		1	
DI864A;Varicer i mavesækken med blødning		1	
DI868;Varicer med anden lokalisation		1	
DI868A;Varices septi nasi		1	
DI87;Andre sygdomme i vener		1	
DI870;Posttrombotisk syndrom		1	
DI871;Compressio venae		1	
DI871A;Strictura venosa		1	
DI871B;Vena cava superior-syndrom		1	
DI871C;Vena cava inferior-syndrom		1	
DI872;Venøs insufficiens		1	
DI872A;Klapinsufficiens i dybe vener på underekstremitet		1	
DI878;Anden sygdom i vener		1	
DI879;Sygdom i vene UNS		1	
DI88;Uspecifik betændelse i lymfekirtler		1	
DI880;Lymfadenitis i mesenteriet UNS		1	
DI880A;Akut lymfadenitis i mesenteriet		1	
DI880B;Kronisk lymfadenitis i mesenteriet		1	
DI881;Anden kronisk lymfadenitis		1	
DI881A;Kronisk lymfadenitis UNS		1	

DI888;Anden lymfadenitis UNS		1	
DI889;Lymfadenitis UNS		1	
DI89;Andre ikke-infektiøse sygdommme i lymfesystemet		1	
DI890;Lymfødem IKA		1	
DI890A;Lymfangiektasi		1	
DI890B;Sekundært lymfødem		1	
DI890C;Pakydermi		1	
DI891;Lymfangitis		1	
DI891A;Lymphangitis subacuta		1	
DI891B;Lymphangitis scleroticans penis		1	
DI891C;Lymphangitis scleroticans vulvae		1	
DI898;Anden ikke-infektiøs sygdom i lymfesystemet		1	
DI898A;Chylocele non filariasis		1	
DI898B;Lipomelanotisk retikulose		1	
DI898C;Histiocytær nekrotiserende lymfadenitis		1	
DI899;Ikke-infektiøs sygdom i lymfesystemet UNS		1	
DI95;Lavt blodtryk		1	
DI950;Idiopatisk hypotension		1	
DI951;Ortostatisk hypotension		1	
DI952;Hypotension forårsaget af lægemiddel		1	
DI958;Anden form for hypotension		1	
DI958A;Kronisk hypotension		1	
DI959;Hypotension UNS		1	
DI97;Kredsløbsforstyrrelser efter indgreb IKA		1	
DI970;Postkardiotomisyndrom		1	
DI971;Anden funktionsforstyrrelse efter hjertekirurgi		1	
DI971A;Hjertesvigt efter hjertekirurgi		1	
DI972;Lymfødem efter mastektomi		1	
DI978;Anden kredsløbsforstyrrelse efter indgreb IKA		1	
DI979;Kredsløbsforstyrrelse efter indgreb UNS		1	
DI98;Andre tilstande i kredsløbet ved sygdomme klassificeret andetsteds		1	
DI980;Syfilitisk kredsløbssygdom		1	

Supplement 2 Nielsen et al. Mortality after paediatric emergency calls for patients with and without pre-existing comorbidity (2023)

DI981;Kredsløbssygdom ved infektiøs eller parasitær sygdom klassificeret andetsteds		1	
DI982;Øsofagusvaricer uden blødning ved sygdom klassificeret andetsteds		1	
DI988;Anden tilstand i kredsløbet ved sygdom klassificeret andetsteds		1	
DI99;Andre og ikke nærmere specificerede kardiovaskulære sygdomme		1	
DI999;Kredsløbssygdom UNS		1	
DJ00;Forkølelse	0		
DJ009;Akut nasofaryngitis UNS	0		
DJ009B;Catarrhalia acuta (findes ikke i SKS)	0		
DJ01;Akut bihulebetændelse	0		
DJ010;Akut kæbehulebetændelse	0		
DJ011;Akut pandehulebetændelse	0		
DJ012;Akut betændelse i sibensceller	0		
DJ013;Akut sfenoidal sinuitis	0		
DJ014;Akut pansinuitis	0		
DJ018;Anden form for akut bihulebetændelse	0		
DJ019;Akut bihulebetændelse UNS	0		
DJ02;Akut faryngitis	0		
DJ020;Akut streptokok faryngitis	0		
DJ020A;Septisk angina	0		
DJ028;Akut faryngitis forårsaget af anden organisme	0		
DJ029;Akut faryngitis UNS	0		
DJ029A;Akut ulcerøs faryngitis	0		
DJ029B;Akut kataralsk faryngitis	0		
DJ029C;Akut gangrænøs faryngitis	0		
DJ03;Akut halsbetændelse	0		
DJ030;Akut streptokok tonsillitis	0		
DJ038;Akut tonsillitis forårsaget af anden organisme	0		
DJ039;Akut tonsillitis UNS	0		
DJ039A;Akut kataralsk tonsillitis	0		
DJ039B;Akut ulcerøs tonsillitis	0		
DJ039C;Akut infektiøs tonsillitis	0		
DJ039D;Akut gangrænøs tonsillitis	0		

DJ039E;Akut folikulær tonsillitis	0			
DJ039F;Akut recidiverende tonsillitis	0			
DJ04;Akut laryngitis og trakeitis	0			
DJ040;Akut laryngitis	0			
DJ040A;Akut ulcerøs laryngitis	0			
DJ040B;Laryngitis subglottica acuta	0			
DJ040C;Akut flegmonøs laryngitis	0			
DJ040D;Akut ødematøs laryngitis	0			
DJ041;Akut trakeitis	0			
DJ042;Akut laryngotrakeitis	0			
DJ05;Pseudocroup og akut epiglottitis	0			
DJ050;Akut obstruktiv laryngitis	0			
DJ051;Epiglottitis acuta	0			
DJ06;Akutte infektioner i øvre luftveje med flere og ikke specificerede lokalisationer	0			
DJ060;Akut laryngofaryngitis	0			
DJ068;Anden akut øvre luftvejsinfektion med flere lokalisationer	0			
DJ069;Akut øvre luftvejsinfektion UNS	0			
DJ09;Influenza forårsaget af identificerede zoonotiske eller pandemiske typer influenzavirus	0			
DJ091;Influenza med påvist influenzavirus A	0			
DJ091A;Influenza med påvist influenzavirus A(H1N1)	0			
DJ091B;Influenza med påvist influenzavirus A(H5N1)	0			
DJ10;Influenza forårsaget af identificerede sæsonbestemte typer influenzavirus	0			
DJ100;Influenza med lungebetændelse forårsaget af anden type influenzavirus	0			
DJ101;Influenza med anden luftvejsmanifestation forårsaget af anden type influenzavirus	0			
DJ101A;Influenza med laryngitis forårsaget af anden type influenzavirus	0			
DJ101B;Influenza med faryngitis forårsaget af anden type influenzavirus	0			
DJ101C;Influenza med pleuraekssudat forårsaget af anden type influenzavirus	0			
DJ108;Influenza med anden manifestation forårsaget af anden type influenzavirus	0			
DJ108A;Influenza med gastroenteritis forårsaget af anden type influenzavirus	0			
DJ108C;Influenza m encefalitis forårs. af an ident. type infl.virus (findes ikke i SKS)	0			
DJ11;Influenza uden påvist influenzavirus	0			
DJ110;Influenza med lungebetændelse uden påvist influenzavirus	0			

Supplement 2 Nielsen et al. Mortality after paediatric emergency calls for patients with and without pre-existing comorbidity (2023)

DJ111;Influenza med anden luftvejsmanifestation uden påvist influenzavirus	0			
DJ111A;Influenza med laryngitis uden påvist influenzavirus	0			
DJ111B;Influenza med faryngitis uden påvist influenzavirus	0			
DJ111C;Influenza med pleuraekssudat uden påvist influenzavirus	0			
DJ118;Influenza med anden manifestation uden påvist influenzavirus	0			
DJ118B;Influenza med gastroenteritis uden påvist influenzavirus	0			
DJ118D;Mellemørebetændelse ved influenza UNS	0			
DJ12;Virus-lungebetændelse IKA	0			
DJ120;Pneumoni forårsaget af adenovirus	0			
DJ121;Pneumoni forårsaget af respiratorisk syncytialvirus	0			
DJ122;Pneumoni forårsaget af parainfluenzavirus	0			
DJ123;Pneumoni forårsaget af humant metapneumovirus	0			
DJ128;Anden viruspneumoni	0			
DJ128A;Svær akut respiratorisk syndrom	0			
DJ129;Viruspneumoni UNS	0			
DJ13;Pneumokok-lungebetændelse	0			
DJ139;Pneumoni forårsaget af Streptococcus pneumoniae	0			
DJ139A;Lobær pneumokokpneumoni	0			
DJ139B;Bronkopneumoni forårsaget af Streptococcus pneumoniae	0			
DJ14;Hæmofilus-lungebetændelse	0			
DJ149;Pneumoni forårsaget af Haemophilus influenzae	0			
DJ149A;Bronkopneumoni forårsaget af Haemophilus influenza	0			
DJ149B;Lobær pneumoni forårsaget af Haemophilus influenza	0			
DJ15;Anden bakteriel lungebetændelse IKA	0			
DJ150;Pneumoni forårsaget af Klebsiella pneumoniae	0			
DJ151;Pneumoni forårsaget af Pseudomonas	0			
DJ152;Pneumoni forårsaget af stafylokokker	0			
DJ153;Pneumoni forårsaget af streptokokker, gruppe B	0			
DJ154;Pneumoni forårsaget af anden streptokok	0			
DJ155;Pneumoni forårsaget af Escherichia coli	0			
DJ156;Pneumoni forårsaget af anden gram-negativ bakterie	0			
DJ156A;Pneumoni forårsaget af Serratia marcescens	0			

Supplement 2 Nielsen et al. Mortality after paediatric emergency calls for patients with and without pre-existing comorbidity (2023)

DJ157;Pneumoni forårsaget af Mycoplasma pneumoniae	0			
DJ158;Anden bakteriel pneumoni	0			
DJ159;Bakteriel pneumoni UNS	0			
DJ16;Lungebetændelse forårsaget af andre infektiøse agentia IKA	0			
DJ160;Klamydiapneumoni	0			
DJ168;Pneumoni forårsaget af andet infektiøst agens	0			
DJ17;Lungebetændelse ved sygdomme klassificeret andetsteds	0			
DJ170;Pneumoni ved bakteriel infektion klassificeret andetsteds	0			
DJ171;Pneumoni ved virusinfektion klassificeret andetsteds	0			
DJ171A;Pneumonia, varicella (findes ikke i SKS)	0			
DJ172;Pneumoni ved mykose klassificeret andetsteds	0			
DJ173;Pneumoni ved parasitær sygdom klassificeret andetsteds	0			
DJ178;Pneumoni ved anden sygdom klassificeret andetsteds	0			
DJ18;Lungebetændelse forårsaget af ikke nærmere specificeret mikroorganisme	0			
DJ180;Bronkopneumoni UNS	0			
DJ181;Lobær pneumoni UNS	0			
DJ182;Hypostatisk pneumoni UNS	0			
DJ188;Anden pneumoni forårsaget af ikke specificeret mikroorganisme UNS	0			
DJ189;Pneumoni UNS	0			
DJ20;Akut bronkitis	0			
DJ200;Akut bronkitis forårsaget af Mycoplasma pneumoniae	0			
DJ200A;Akut bronkitis forårsaget af Mycoplasma pneumoniae med bronkospasme	0			
DJ201;Akut bronkitis forårsaget af Haemophilus influenzae	0			
DJ201A;Akut bronkitis forårsaget af Haemophilus influenzae med bronkospasme	0			
DJ202;Akut bronkitis forårsaget af streptokokker	0			
DJ202A;Akut bronkitis forårsaget af streptokokker med bronkospasme	0			
DJ203;Akut bronkitis forårsaget af coxsackie-virus	0			
DJ203A;Akut bronkitis forårsaget af coxsackie-virus med bronkospasme	0			
DJ204;Akut bronkitis forårsaget af parainfluenzaevirus	0			
DJ204A;Akut bronkitis forårsaget af parainfluenzaevirus med bronkospasme	0			
DJ205;Akut bronkitis forårsaget af respiratorisk syncytialvirus	0			
DJ205A;Akut bronkitis forårsaget af respiratorisk syncytialvirus med bronkospasme	0			

DJ206;Akut bronkitis forårsaget af rhinovirus	0			
DJ206A;Akut bronkitis forårsaget af rhinovirus med bronkospasme	0			
DJ207;Akut bronkitis forårsaget af echovirus	0			
DJ207A;Akut bronkitis forårsaget af echovirus med bronkospasme	0			
DJ208;Akut bronkitis forårsaget af anden mikroorganisme	0			
DJ208A;Akut bronkitis forårsaget af anden mikroorganisme med bronkospasme	0			
DJ209;Akut bronkitis UNS	0			
DJ209A;Akut bronkitis UNS med bronkospasme	0			
DJ21;Akut bronkiolitis	0			
DJ210;Akut bronkiolitis forårsaget af respiratorisk syncytialvirus	0			
DJ211;Akut bronkiolitis forårsaget af humant metapneumovirus	0			
DJ218;Akut bronkiolitis forårsaget af anden mikroorganisme	0			
DJ219;Akut bronkiolitis UNS	0			
DJ22;Ikke nærmere specificerede akutte infektioner i nedre luftveje	0			
DJ229;Akut nedre luftvejsinfektion UNS	0			
DJ30;Høfeber og allergisk rinitis	0			
DJ300;Vasomotorisk rinitis	0			
DJ300B;Erhvervsbetinget vasomotorisk rinitis	0			
DJ301;Pollenudløst allergisk rinitis	0			
DJ301A;Febris autumnalis	0			
DJ301B;Febris aestivalis	0			
DJ301C;Febris vernalis	0			
DJ302;Anden sæsonbetinget allergisk rinitis	0			
DJ303;Ikke-sæsonbetinget allergisk rinitis	0			
DJ303A;Erhvervsbetinget ikke-sæsonbetinget allergisk rinitis	0			
DJ304;Allergisk rinitis UNS	0			
DJ31;Kronisk næsekatar, næsesvælgkatar og svælgkatar	0			
DJ310;Kronisk rinitis	0			
DJ311;Kronisk nasofaryngitis	0			
DJ312;Kronisk faryngitis	0			
DJ32;Kronisk bihulebetændelse	0			
DJ320;Kronisk kæbehulebetændelse	0			

Supplement 2 Nielsen et al. Mortality after paediatric emergency calls for patients with and without pre-existing comorbidity (2023)

DJ321;Kronisk pandehulebetændelse	0		
DJ322;Kronisk betændelse i sibensceller	0		
DJ323;Kronisk sfenoidal sinuitis	0		
DJ324;Kronisk pansinuitis	0		
DJ328;Anden form for kronisk bihulebetændelse	0		
DJ329;Kronisk bihulebetændelse UNS	0		
DJ33;Næsepolyp	0		
DJ330;Polyp i næsehulen	0		
DJ331;Polypøs sinusdegeneration	0		
DJ331A;Woakes' ethmoiditis	0		
DJ338;Anden form for næsepolyp	0		
DJ338A;Polypus sinus sphenoidalis	0		
DJ338B;Polyp i kæbehule	0		
DJ338C;Polyp i sibenscelle	0		
DJ338D;Polyp i pandehule	0		
DJ339;Næsepolyp UNS	0		
DJ34;Andre sygdomme i næse og bihuler	0		
DJ340;Absces, furunkel eller karbunkel i næsen	0		
DJ340A;Absces i næsen	0		
DJ340B;Karbunkel i næsen	0		
DJ340D;Flegmone i næsen	0		
DJ340E;Sår i næsen	0		
DJ340F;Sår i næseskillevæggen	0		
DJ340G;Nekrose i næseskillevæggen	0		
DJ340H;Nekrose i næsen	0		
DJ340I;Flegmone i næseskillevæggen	0		
DJ340J;Furunkel i næsen	0		
DJ341;Cyste eller mukocele i næsehule eller bihule	0		
DJ341A;Cyste i bihule	0		
DJ341B;Mukocele i bihule	0		
DJ341C;Cyste i næsehule	0		
DJ341D;Mukocele i næsehule	0		

DJ342;Næseseptum deviation	0			
DJ343;Hypertrophia conchae nasalis	0			
DJ343A;Concha bullosa	0			
DJ348;Anden sygdom i næsehule eller bihule	0			
DJ348A;Rhinolithiasis	0			
DJ348B;Perforation af næseskillevæggen UNS	0			
DJ348C;Insufficientia alae nasi	0			
DJ35;Kroniske sygdomme i tonsiller og adenoide vegetationer	0			
DJ350;Kronisk tonsillitis	0			
DJ351;Hypertrofi af tonsiller	0			
DJ351A;Hypertrofi af ganetonsiller	0			
DJ351B;Hypertrofi af tungetonsiller	0			
DJ352;Adenoide vegetationer	0			
DJ353;Hypertrofi af tonsiller med adenoide vegetationer	0			
DJ358;Anden kronisk sygdom i tonsiller og adenoide vegetationer	0			
DJ359;Kronisk sygdom i tonsiller eller adenoide vegetationer UNS	0			
DJ36;Halsbyld	0			
DJ369;Peritonsillær absces	0			
DJ37;Kronisk strubekatar og luftrørskatar	0			
DJ370;Kronisk laryngitis	0			
DJ370A;Tør laryngitis	0			
DJ370B;Kataralsk laryngitis	0			
DJ370C;Hypertrofisk laryngitis	0			
DJ371;Kronisk laryngotrakeitis	0			
DJ38;Sygdomme i stemmebånd og struben IKA	0			
DJ380;Paralyse af stemmebånd eller struben	0			
DJ380A;Ikke-traumatisk nervus recurrens parese	0			
DJ380B;Paralyse af stemmebånd	0			
DJ380C;Paralyse af stemmebånd efter kirurgisk indgreb	0			
DJ380C1;Permanent paralyse af stemmebånd efter kirurgisk indgreb	0			
DJ381;Polyp på stemmebånd eller i struben	0			
DJ381A;Polyp i larynx	0			

DJ381B;Polyp på stemmebånd	0			
DJ382;Noduli vocalis	0			
DJ382A;Betændelse i stemmebånd	0			
DJ383;Anden sygdom i stemmebånd	0			
DJ383A;Leukokeratose i stemmebånd	0			
DJ383B;Stemmebåndsgranulom	0			
DJ383C;Cellulitis i stemmebånd	0			
DJ383D;Absces i stemmebånd	0			
DJ383E;Leukoplaki på stemmebånd	0			
DJ383V;Vocal Cord Dysfunction	0			
DJ384;Larynxødem	0			
DJ384A;Supraglottisk ødem	0			
DJ384B;Glottisødem	0			
DJ384C;Subglottisk ødem	0			
DJ384D;Reincke-ødem i stemmebånd	0			
DJ385;Laryngospasme	0			
DJ385A;Laryngismus stridulus	0			
DJ385B;Spasmus glottidis	0			
DJ386;Laryngostenose	0			
DJ387;Anden sygdom i larynx	0			
DJ387A;Pachyderma laryngis	0			
DJ387B;Perikondritis i larynx	0			
DJ387C;Nekrose i larynx	0			
DJ387D;Ulcus i larynx	0			
DJ387E;Sygdom i larynx UNS	0			
DJ387F;Cellulitis i larynx	0			
DJ387G;Absces i larynx	0			
DJ388;Inducerbar laryngeal obstruktion (ILO)	0			
DJ388A;Glottisk inducerbar laryngeal obstruktion (ILO)	0			
DJ388A1;Anstrengelsesudløst glottisk inducerbar laryngeal obstruktion (EILO)	0			
DJ388A2;Irritantudløst glottisk inducerbar laryngeal obstruktion (ILO)	0			
DJ388B;Supraglottisk inducerbar laryngeal obstruktion (ILO)	0			

Supplement 2 Nielsen et al. Mortality after paediatric emergency calls for patients with and without pre-existing comorbidity (2023)

DJ388B1;Anstrengelsesudløst supraglottisk inducerbar laryngeal obstruktion (EILO)	0		
DJ388B2;Irritantudløst supraglottisk inducerbar laryngeal obstruktion (ILO)	0		
DJ388C;Kombineret glottisk og supraglottisk inducerbar laryngeal obstruktion (ILO)	0		
DJ388C1;Anstrengelsesudløst kombineret glottisk og supraglottisk inducerbar laryngeal obstruktio	0		
DJ388C2;Irritantudløst kombineret glottisk og supraglottisk inducerbar laryngeal obstruktion (ILO)	0		
DJ39;Andre sygdomme i øvre luftveje	0		
DJ390;Retrofaryngeal eller parafaryngeal absces	0		
DJ390A;Perifaryngeal absces	0		
DJ390B;Retrofaryngeal absces	0		
DJ390C;Parafaryngeal absces	0		
DJ391;Anden absces i pharynx	0		
DJ391A;Absces i pharynx	0		
DJ391B;Absces i nasopharynx	0		
DJ391C;Cellulitis i pharynx	0		
DJ392;Anden sygdom i pharynx	0		
DJ392A;Ødem i nasopharynx	0		
DJ392B;Cyste i pharynx	0		
DJ392C;Cyste i nasopharynx	0		
DJ392D;Pharynxødem	0		
DJ398;Anden sygdom i øvre luftveje	0		
DJ398A;Absces i trakea	0		
DJ399;Sygdom i øvre luftveje UNS	0		
DJ40;Bronkitis ikke specificeret som akut eller kronisk	0		
DJ409;Bronkitis UNS	0		
DJ41;Kronisk bronkitis uden obstruktion		1	
DJ410;Simpel kronisk bronkitis		1	
DJ411;Mukopurulent kronisk bronkitis		1	
DJ418;Blandet simpel og mukopurulent kronisk bronkitis		1	
DJ42;Kronisk bronkitis ikke nærmere specificeret		1	
DJ429;Kronisk bronkitis UNS		1	
DJ429A;Kronisk trakeobronkitis		1	
DJ429B;Kronisk trakeitis		1	

DJ43;Lungeemfysem		1	
DJ430;Enkeltsidigt lungeemfysem		1	
DJ431;Panlobulært lungeemfysem		1	
DJ431A;Panacinært emfysem		1	
DJ432;Centrilobulært emfysem		1	
DJ438;Anden form for lungeemfysem		1	
DJ439;Lungeemfysem UNS		1	
DJ439A;Bulløst lungeemfysem		1	
DJ44;Kronisk obstruktiv lungesygdom		1	
DJ440;Kronisk obstruktiv lungesygdom med akut nedre luftvejs infektion		1	
DJ441;Kronisk obstruktiv lungesygdom med akut eksacerbation UNS		1	
DJ448;Anden form for kronisk obstruktiv lungesygdom		1	
DJ448A;Kronisk obstruktiv bronkitis		1	
DJ448B;Kronisk astmatisk bronkitis		1	
DJ448C;Kronisk bronkitis med emfysem		1	
DJ449;Kronisk obstruktiv lungesygdom UNS		1	
DJ45;Astma		1	
DJ450;Allergisk astma		1	
DJ450A;Erhvervsbetinget allergisk astma		1	
DJ451;Ikke-allergisk astma		1	
DJ451A;Erhvervsbetinget ikke-allergisk astma		1	
DJ458;Astma af blandet type		1	
DJ459;Astma UNS		1	
DJ46;Status asthmaticus		1	
DJ469;Status asthmaticus UNS		1	
DJ47;Udvidelse af bronkier		1	
DJ479;Bronkiektasi		1	
DJ60;Støvlunge forårsaget af kulstøv		1	
DJ609;Pneumokoniose forårsaget af kulstøv		1	
DJ609A;Anthracosilicosis		1	
DJ61;Støvlunge forårsaget af asbest og andre mineralfibre		1	
DJ619;Pneumokoniose forårsaget af asbest og andre mineralfibre		1	

DJ62;Støvlunge forårsaget af silikater		1	
DJ620;Pneumokoniose forårsaget af talkum		1	
DJ628;Anden form for silikose		1	
DJ628A;Silikose med lungefibrose		1	
DJ628B;Silikose UNS		1	
DJ63;Støvlunge forårsaget af andet uorganisk støv		1	
DJ630;Lungealuminose		1	
DJ631;Lungefibrose forårsaget af bauxit		1	
DJ632;Berylliose		1	
DJ633;Lungefibrose forårsaget af grafit		1	
DJ634;Lungesiderose		1	
DJ635;Lungefibrose forårsaget af tinoksid		1	
DJ638;Pneumokoniose forårsaget af andet uorganisk støv		1	
DJ638A;Lungefibrose forårsaget af stenstøv		1	
DJ638B;Lungefibrose forårsaget af metalstøv		1	
DJ64;Støvlunge UNS		1	
DJ649;Pneumokoniose UNS		1	
DJ65;Støvlunge med tuberkulose		1	
DJ659;Pneumokoniose med tuberkulose UNS		1	
DJ66;Luftvejssygdomme forårsaget af organisk støv		1	
DJ660;Byssinose		1	
DJ661;Hørarbejdersygdom		1	
DJ662;Cannabinose		1	
DJ668;Luftvejssygdom forårsaget af andet organisk støv		1	
DJ67;Pneumonitis forårsaget af hypersensitivitet overfor organisk støv		1	
DJ670;Tærskerlunger		1	
DJ671;Bagassose		1	
DJ672;Fugleholderlunger		1	
DJ673;Suberose		1	
DJ674;Maltarbejderlunger		1	
DJ675;Svampedyrkerlunger		1	
DJ676;Cryptostromose		1	

Supplement 2 Nielsen et al. Mortality after paediatric emergency calls for patients with and without pre-existing comorbidity (2023)

DJ677;Luftfugterlunger		1	
DJ678;Allergisk alveolitis forårsaget af andet organisk støv		1	
DJ678A;Pelsværkarbejderlunger		1	
DJ678B;Sequoiose		1	
DJ678C;Ostevaskerlunger		1	
DJ678D;Kaffeastma		1	
DJ678E;Fiskemelsarbejderlunger		1	
DJ679;Allergisk alveolitis forårsaget af organisk støv UNS		1	
DJ679A;Hypersensitivitetspneumonitis UNS		1	
DJ68;Luftvejssygdomme forårsaget af indåndede kemikalier, gasser, røg og dampe		1	
DJ680;Bronkitis eller pneumonitis forårsaget af indåndede kemikalier, gasser, røg eller dampe		1	
DJ680B;Bronkitis forårsaget af indåndede kemikalier, gasser, røg eller dampe		1	
DJ680C;Pneumonitis forårsaget af indåndede kemikalier, gasser, røg eller dampe		1	
DJ681;Lungeødem forårsaget af indåndede kemikalier, gasser, røg eller dampe		1	
DJ682;Inflammation i øvre luftveje forårsaget af indåndede kemikalier, gasser, røg eller dampe		1	
DJ683;Anden akut eller subakut tilstand i luftveje forårsaget af indåndede kemikalier, gasser, røg eller dampe		1	
DJ684;Kronisk sygdom i luftveje forårsaget af indåndede kemikalier, gasser, røg eller dampe		1	
DJ684A;Lungefibrose forårsaget af indåndede kemikalier, gasser, røg eller dampe		1	
DJ684B;Lungeemfysem forårsaget af indåndede kemikalier, gasser, røg eller dampe		1	
DJ684C;Oblitererende bronkiolitis forårsaget af indåndede kemikalier, gasser, røg eller dampe		1	
DJ688;Anden sygdom i luftveje forårsaget af indåndede kemikalier, gasser, røg eller dampe		1	
DJ689;Sygdom i luftveje UNS forårsaget af indåndede kemikalier, gasser, røg eller dampe		1	
DJ69;Lungeinflammation forårsaget af aspiration af faste og flydende stoffer		1	
DJ690;Aspirationspneumoni forårsaget af fødeemner eller maveindhold		1	
DJ691;Aspirationspneumoni forårsaget af olie eller fedtstoffer		1	
DJ698;Aspirationspneumoni forårsaget af andet fast eller flydende stof		1	
DJ698A;Aspirationspneumoni forårsaget af blod		1	
DJ70;Andre tilstande i luftvejene forårsaget af andre ydre påvirkninger		1	
DJ700;Strålepneumonitis		1	
DJ701;Lungefibrose efter bestråling		1	
DJ702;Akut interstitiel lungesygdom forårsaget af lægemiddel		1	
DJ703;Kronisk interstitiel lungesygdom forårsaget af lægemiddel		1	

DJ704;Interstitiel lungesygdom forårsaget af lægemiddel UNS		1	
DJ708;Anden tilstand i luftvejene efter anden ydre påvirkning		1	
DJ709;Tilstand i luftvejene efter ydre påvirkning UNS		1	
DJ80;Respirationssvigt som følge af alveolebeskadigelse		1	
DJ801;Diffus alveolær skade (DAD)		1	
DJ809;Alveolebeskadigelse med respirationssvigt (ARDS)		1	
DJ809A;Mild ARDS		1	
DJ809B;Moderat ARDS		1	
DJ809C;Svær ARDS		1	
DJ81;Lungeødem		1	
DJ819;Lungeødem UNS		1	
DJ82;Lungeeosinofili		1	
DJ821;Eosinofil pneumoni		1	
DJ829;Eosinofile lungeinfiltrater		1	
DJ84;Andre interstitielle lungesygdomme		1	
DJ840;Alveolær eller parietoalveolær sygdom		1	
DJ840A;Proteinosis alveolaris pulmonum		1	
DJ840B;Microlithiasis alveolaris pulmonum		1	
DJ841;Anden interstitiel lungesygdom med fibrose		1	
DJ841A;Idiopatisk lungefibrose med usual interstitial pneumonia (UIP)		1	
DJ841B;Idiopatisk lungefibrose med non-specific interstitial pneumonia		1	
DJ841C;Idiopatisk lungefibrose UNS		1	
DJ841D;Akut interstitiel pneumoni		1	
DJ841F;Bronkiolitis obliterans organiserende pneumoni (BOOP)		1	
DJ841G;Deskvamativ interstitiel pneumoni		1	
DJ841X;Lungefibrose UNS		1	
DJ848;Anden interstitiel lungesygdom		1	
DJ848A;Allergisk bronkopulmonal aspergillose		1	
DJ849;Interstitiel lungesygdom UNS		1	
DJ85;Byld i lunge og i mediastinum		1	
DJ850;Gangræn og nekrose i lunge		1	
DJ851;Lungeabsces med pneumoni		1	

DJ852;Lungeabsces UNS		1	
DJ853;Absces i mediastinum		1	
DJ86;Pyothorax		1	
DJ860;Pleuraempyem med fistel		1	
DJ869;Pleuraempyem UNS		1	
DJ90;Væskeansamling i lungehinde IKA		1	
DJ909;Pleuraeffusion IKA		1	
DJ91;Væskeansamling i lungehinde ved sygdom klassificeret andetsteds		1	
DJ919;Pleuraeffusion ved sygdom klassificeret andetsteds		1	
DJ92;Fortykkelse og forkalkning af lungehinde		1	
DJ920;Pleurale plaques og belægninger efter kendt asbesteksponering		1	
DJ929;Pleurale plaques og belægninger uden kendt asbesteksponering		1	
DJ93;Pneumothorax		1	
DJ930;Spontan trykpneumothorax		1	
DJ931;Anden spontan pneumothorax		1	
DJ938;Anden form for pneumothorax		1	
DJ939;Pneumothorax UNS		1	
DJ94;Andre sygdomme i lungehinderne		1	
DJ940;Kylothorax		1	
DJ941;Fibrothorax		1	
DJ942;Hæmothorax		1	
DJ942A;Hæmopneumothorax		1	
DJ948;Anden sygdom i lungehinder		1	
DJ948A;Hydrothorax		1	
DJ949;Sygdom i lungehinder UNS		1	
DJ95;Tilstande i åndedrætsorganer efter indgreb IKA		1	
DJ950;Trakeostomiproblemer			2
DJ950A;Sepsis udgået fra trakeostomistoma			2
DJ950B;Trakeo-øsofageal fistel efter trakeostomi			2
DJ950C;Blødning fra trakeostomistoma			2
DJ950D;Luftvejsobstruktion ved trakeostomi			2
DJ951;Akut respirationsinsufficiens efter thoraxkirurgi		1	

Supplement 2 Nielsen et al. Mortality after paediatric emergency calls for patients with and without pre-existing comorbidity (2023)

DJ952;Akut respirationsinsufficiens efter ekstratorakal kirurgi		1		
DJ953;Kronisk respirationsinsufficiens efter kirurgi UNS		1		
DJ954;Postaspirationssyndrom		1		
DJ955;Subglottisk stenose efter indgreb		1		
DJ958;Anden tilstand i respirationsveje efter indgreb		1		
DJ958A;Pneumothorax efter indgreb på brystvæg og lunge		1		
DJ959;Tilstand i respirationsveje UNS efter indgreb		1		
DJ96;Respirationsinsufficiens IKA		1		
DJ960;Akut respirationsinsufficiens		1		
DJ961;Kronisk respirationsinsufficiens		1		
DJ969;Respirationsinsufficiens UNS		1		
DJ98;Andre tilstande i åndedrætsorganer		1		
DJ980;Sygdom i luftrør eller bronkier IKA		1		
DJ980A;Bronkolitiasis		1		
DJ980B;Forkalkning i bronkier		1		
DJ980C;Forkalkning i luftrør		1		
DJ980D;Trakealstenose		1		
DJ980E;Sår i luftrør		1		
DJ980F;Sår i bronkie		1		
DJ980G;Bronkialstenose		1		
DJ980H;Dyskinesia tracheobronchialis		1		
DJ980I;Collapsus tracheobronchialis		1		
DJ981;Atelektase i lunge		1		
DJ982;Interstitielt lungeemfysem		1		
DJ982A;Mediastinalt emfysem		1		
DJ983;Kompensatorisk emfysem		1		
DJ984;Anden lungesygdom		1		
DJ984A;Pulmolitiasis		1		
DJ984B;Forkalkning i lunge		1		
DJ984C;Lungecyste		1		
DJ985;Sygdom i mediastinum IKA		1		
DJ985A;Retractio mediastini		1		

DJ985B;Mediastinal fibrose		1	
DJ985C;Mediastinalt hernie		1	
DJ985D;Mediastinitis UNS		1	
DJ986;Sygdom i diafragma		1	
DJ986A;Parese af nervus phrenicus		1	
DJ986B;Paralyse af diafragma		1	
DJ986C;Relaxatio diaphragmatica		1	
DJ986D;Diaphragmatitis		1	
DJ988;Anden tilstand i åndedrætsorganer		1	
DJ988A;Isoleret ciliedyskinesi		1	
DJ989;Tilstand i åndedrætsorgan UNS		1	
DJ99;Lungeforandringer ved sygdom klassificeret andetsteds		1	
DJ990;Lungeforandringer ved reumatoid sygdom		1	
DJ991;Lungeforandringer ved anden bindevævssygdom		1	
DJ998;Lungeforandringer ved anden sygdom klassificeret andetsteds		1	
DK00;Forstyrrelser i tænders udvikling og frembrud	0		
DK000;Anodonti	0		
DK000A;Aplasi af tand	0		
DK001;Overtallige tænder	0		
DK002;Afvigelser i størrelse og form af tænder	0		
DK002A;Dens evaginatus	0		
DK002B;Dens invaginatus	0		
DK002C;Enamelom	0		
DK002D;Abnorm form på tand UNS	0		
DK002E;Macrodontia	0		
DK002G;Microdontia	0		
DK003;Opacitet i tandemalje	0		
DK003A;Fluorosis dentalis	0		
DK004;Forstyrrelse i tanddannelse	0		
DK004A;Turners tænder	0		
DK004B;Hypoplasi af tandemalje	0		
DK005;Arvelig tandanomali IKA	0		

Supplement 2 Nielsen et al. Mortality after paediatric emergency calls for patients with and without pre-existing comorbidity (2023)

DK005A;Amelogenesis imperfecta	0			
DK005B;Dentinogenesis imperfecta	0			
DK005C;Dysplasi af tand	0			
DK006;Abnormt tandfrembrud	0			
DK006A;Dentitio praecox	0			
DK006B;Eruptio dentium tarda	0			
DK006C;Dens deciduus persistens	0			
DK006D;Dens neonatalis	0			
DK007;Eruptiopati	0			
DK007A;Dentitio difficilis	0			
DK008;Anden forstyrrelse i tandudviklingen	0			
DK008A;Endogen misfarvning af tænder UNS	0			
DK008B;Misfarvning af tænder før frembrud	0			
DK009;Forstyrrelse i tandudviklingen UNS	0			
DK01;Ikke-frembrudt tand	0			
DK010;Retineret tand	0			
DK011;Impakteret tand	0			
DK011A;Dens non eruptus med obstruktion fra en anden tand (findes ikke i SKS)	0			
DK02;Huller i tænder	0			
DK020;Karies i tandemaljen	0			
DK021;Karies i tandbenet	0			
DK022;Karies i tandcementen	0			
DK023;Caries dentium arrestum	0			
DK024;Odontoklasi	0			
DK025;Karies med pulpa eksponering	0			
DK028;Anden form for karies	0			
DK029;Karies UNS	0			
DK03;Andre sygdomme i det hårde tandvæv	0			
DK030;Attritio dentium	0			
DK031;Abrasio dentium	0			
DK032;Erosio dentium	0			
DK033;Resorptio dentium	0			

DK033A;Granuloma internum pulpae dentium	0			
DK033B;Resorptio dentium externi	0			
DK033C;Resorptio dentium interni	0			
DK034;Hypercementosis	0			
DK035;Ankylosis dento-alveolaris	0			
DK036;Tandbelægninger	0			
DK036A;Accretio dentis	0			
DK036B;Tandsten	0			
DK036C;Ydre misfarvning af tænder UNS	0			
DK037;Misfarvning af tænder efter frembrud	0			
DK038;Anden sygdom i det hårde tandvæv	0			
DK038A;Adamantina irradiata	0			
DK038B;Dentinum sensitivum	0			
DK038C;Enamelum irradiatum	0			
DK039;Sygdom i det hårde tandvæv UNS	0			
DK04;Sygdom i tandpulpa og det periapikale væv	0			
DK040;Pulpitis	0			
DK040A;Absces i tandpulpa	0			
DK040B;Polyp i tandpulpa	0			
DK041;Nekrose i tandpulpa	0			
DK041A;Gangræn i tandpulpa	0			
DK042;Degeneration af tandpulpa	0			
DK043;Abnorm dentindannelse i tandpulpa	0			
DK044;Akut apikal parodontitis	0			
DK045;Kronisk apikal parodontitis	0			
DK045A;Periapikalt granulom	0			
DK045B;Apikalt granulom	0			
DK046;Periapikal tandabsces med fistel	0			
DK046A;Alveolær tandabsces med fistel	0			
DK046B;Fistula dentalis	0			
DK046C;Fistula processus alveolaris	0			
DK046D;Fistula periapicalis	0			

Supplement 2 Nielsen et al. Mortality after paediatric emergency calls for patients with and without pre-existing comorbidity (2023)

DK046E;Parulis med fistel	0		
DK047;Periapikal tandabsces uden fistel	0		
DK047A;Alveolær tandabsces uden fistel	0		
DK047B;Parulis UNS	0		
DK048;Tandrodscyste	0		
DK048A;Periodontal cyste	0		
DK048B;Periapikal cyste	0		
DK048C;Apikal cyste	0		
DK049;Anden eller ikke specificeret sygdom i tandpulpa eller periapikalt væv	0		
DK05;Gingivitis og parodontale sygdomme	0		
DK050;Akut gingivitis	0		
DK051;Kronisk gingivitis	0		
DK051A;Ulcerøs gingivitis	0		
DK051B;Gingivitis UNS	0		
DK051C;Hyperplastisk gingivitis	0		
DK051D;Kronisk gingivitis forårsaget af lægemiddel	0		
DK052;Akut parodontitis	0		
DK052A;Parodontal absces	0		
DK052B;Akut marginal parodontitis	0		
DK052C;Akut perikoronitis	0		
DK053;Kronisk parodontitis	0		
DK053A;Parodontitis UNS	0		
DK053B;Kronisk perikoronitis	0		
DK054;Parodontose	0		
DK054A;Juvenil parodontose	0		
DK055;Anden parodontal sygdom	0		
DK056;Parodontal sygdom UNS	0		
DK06;Andre sygdomme i tandkød og tilstødende væv	0		
DK060;Gingivaretraktion	0		
DK061;Gingival hyperplasi	0		
DK061A;Gingival fibromatose	0		
DK061B;Irritativ hyperplasi af processus alveolaris efter tab af tænder	0		

Supplement 2 Nielsen et al. Mortality after paediatric emergency calls for patients with and without pre-existing comorbidity (2023)

DK062;Traumatiske forandringer i gingiva eller processus alveolaris efter tab af tænder	0		
DK062A;Traumatiske forandringer processus alveolaris efter tab af tænder	0		
DK062B;Traumatiske forandringer i gingiva	0		
DK068;Anden sygdom i gingiva eller processus alveolaris efter tab af tænder	0		
DK068A;Gingivacyste	0		
DK068B;Epulis gigantocellularis UNS	0		
DK068C;Epulis fibrosus	0		
DK068D;Pyogent granulom i gingiva	0		
DK068E;Granuloma teleangiectaticum gingivae	0		
DK068F;Perifert kæmpecellegranulom i underkæben	0		
DK068G;Perifert kæmpecellegranulom i overkæben	0		
DK068H;Misfarvning af gingiva	0		
DK068I;Erytroplaki på gingiva	0		
DK068K;Melanoplaki på gingiva	0		
DK068L;Forreste mundhulefistel	0		
DK069;Sygdom i tandkød eller tilstødende væv UNS	0		
DK07;Dento-faciale anomalier	0		
DK070;Større abnormiteter i kæbernes dimensioner	0		
DK070A;Hyperplasi af overkæben	0		
DK070B;Hyperplasi af underkæben	0		
DK070C;Hypoplasi af overkæben	0		
DK070D;Hypoplasi af underkæben	0		
DK071;Abnormt forhold mellem kæber og basis cranii	0		
DK071A;Asymmetrisk underkæbe	0		
DK071B;Asymmetrisk overkæbe	0		
DK071C;Prominerende overkæbe	0		
DK071D;Prominerende underkæbe	0		
DK071E;Vigende overkæbe	0		
DK071F;Vigende underkæbe	0		
DK072;Bidanomali	0		
DK072A;Åbent frontalt bid	0		
DK072B;Dybt bid	0		

Supplement 2 Nielsen et al. Mortality after paediatric emergency calls for patients with and without pre-existing comorbidity (2023)

DK072C;Dybt bid med fortandssammenbid	0			
DK072D;Åbent lateralt bid	0			
DK072E;Invertering af tand	0			
DK072F;Krydsbid	0			
DK072G;Midtlinjeforskydning af fortænder	0			
DK072H;Mesial okklusion af tænder	0			
DK072I;Distal okklusion af tænder	0			
DK072J;Maksilært overbid	0			
DK072K;Ekstremt overbid	0			
DK072L;Horisontalt overbid	0			
DK073;Fejlstilling af tænder	0			
DK073A;Diastema dentium	0			
DK073B;Ektopi af tænder	0			
DK073C;Rotation af tænder	0			
DK073D;Spredtstilling af tænder	0			
DK073E;Trangstilling af tænder	0			
DK073F;Transposition af tænder	0			
DK074;Malokklusion UNS	0			
DK075;Funktionsforstyrrelser i kæber og ansigt	0			
DK075A;Funktionsforstyrrelser i kæber og ansigt med malokklusion	0			
DK075B;Constrictio maxillarum	0			
DK075C;Forstyrrelse i tyggefunktionen	0			
DK076;Lidelse i kæbeled	0			
DK076A;Smertebetinget funktionsforstyrrelse i kæbeled	0			
DK076B;Klik i kæbeled	0			
DK076C;Ankylose i kæbeled	0			
DK076D;Slidgigt i kæbeled	0			
DK076E;Kondromatose i kæbeled	0			
DK076F;Dislokation af ledskive i kæbeled	0			
DK076M;Mandibulær dysfunktion UNS	0			
DK078;Anden dento-facial anomali	0			
DK079;Dento-facial anomali UNS	0			

Supplement 2 Nielsen et al. Mortality after paediatric emergency calls for patients with and without pre-existing comorbidity (2023)

DK08;Andre sygdomme i tænder og støttevæv	0			
DK080;Tab af tænder ved systemisk sygdom	0			
DK081;Tab af tænder ved ulykke, ekstraktion eller parodontal sygdom	0			
DK081A;Tab af tænder ved ulykke	0			
DK081B;Tab af tænder ved parodontal sygdom	0			
DK081C;Tab af tænder ved ekstraktion	0			
DK081D;Partiel tandløshed	0			
DK082;Atrofi af processus alveolaris efter tab af tænder	0			
DK083;Retineret tandrod	0			
DK083A;Tandsekvester	0			
DK088;Anden sygdom i tænder eller støttevæv	0			
DK088A;Hypertrofi af processus alveolaris UNS	0			
DK088B;Tandpine UNS	0			
DK088C;Ujævn processus alveolaris	0			
DK089;Sygdom i tænder eller støttevæv UNS	0			
DK089A;Blødning fra processus alveolaris UNS	0			
DK09;Cyster i mundregionen IKA	0			
DK090;Odontogen udviklingscyste	0			
DK090A;Cystis periodontalis lateralis	0			
DK090B;Cystis primordialis	0			
DK090C;Cystis dentalis follicularis	0			
DK090D;Keratocystis odontogenica	0			
DK091;Ikke-odontogen udviklingscyste i mundregionen	0			
DK091A;Cystis nasopalatina	0			
DK091B;Cystis processus alveolaris	0			
DK091C;Cystis fissuralis maxillae	0			
DK091D;Cystis mediana palati	0			
DK091E;Cystis fissuralis mandibulae	0			
DK091F;Cystis canalis incisivus	0			
DK092;Anden kæbecyste	0			
DK092A;Cystis maxillae traumatica	0			
DK092B;Overkæbecyste UNS	0			

DK092C;Cystis mandibulae aneurysmatica	0			
DK092D;Cystis maxillae haemorrhagica	0			
DK092E;Cystis maxillae aneurysmatica	0			
DK092F;Cystis mandibulae haemorrhagica	0			
DK092G;Cystis mandibulae traumatica	0			
DK092H;Underkæbecyste UNS	0			
DK092I;Cystis residualis	0			
DK098;Anden cyste i mundregionen IKA	0			
DK098A;Cystis nasolabialis	0			
DK098B;Cystis lymphoepithelialis regionis oralis	0			
DK098C;Cystis nasoalveolaris	0			
DK098D;Cystis dermoides regionis oralis	0			
DK098E;Epsteins perle	0			
DK099;Cyste i mundregionen UNS	0			
DK10;Andre sygdomme i kæber	0			
DK100;Udviklingsforstyrrelse i kæbe	0			
DK100A;Cystis latens mandibulae	0			
DK100B;Cystis latens maxillae	0			
DK100C;Udviklingsforstyrrelse i overkæbe	0			
DK100D;Udviklingsforstyrrelse i underkæbe	0			
DK100E;Cystis statica mandibulae	0			
DK100F;Torus palatinus	0			
DK100G;Torus mandibularis	0			
DK101;Centralt kæmpecellegranulom i kæbe	0			
DK101A;Centralt kæmpecellegranulom i overkæben	0			
DK101B;Centralt kæmpecellegranulom i underkæben	0			
DK102;Betændelsestilstand i kæbe	0			
DK102A;Ostitis maxillae	0			
DK102B;Ostitis mandibulae	0			
DK102C;Osteomyelitis mandibulae	0			
DK102D;Osteomyelitis maxillae	0			
DK102E;Osteoradionecrosis mandibulae	0			

DK102F;Osteoradionecrosis maxillae	0			
DK102G;Periostitis mandibulae	0			
DK102H;Periostitis maxillae	0			
DK102I;Sequestrum maxillae	0			
DK102J;Sequestrum mandibulae	0			
DK103;Ostitis alveolaris	0			
DK103B;Periostitis alveolaris	0			
DK108;Anden sygdom i kæbe	0			
DK108A;Atrofi af underkæben	0			
DK108B;Atrofi af overkæben	0			
DK108C;Cherubismus mandibulae	0			
DK108D;Cherubismus maxillae	0			
DK108E;Exostosis maxillae	0			
DK108F;Exostosis mandibulae	0			
DK108G;Hyperplasia condylaris unilateralis mandibulae	0			
DK108H;Hypoplasia condylaris unilateralis mandibulae	0			
DK108I;Ostitis fibrosa mandibulae	0			
DK108J;Ostitis fibrosa maxillae	0			
DK109;Sygdom i kæbe UNS	0			
DK11;Sygdomme i spytkirtler	0			
DK110;Atrofi af spytkirtel	0			
DK110A;Atrofi af glandula parotidea	0			
DK111;Hypertrofi af spytkirtel	0			
DK111A;Hypertrofi af glandula parotidea	0			
DK111B;Hypertrofi af glandula submandibularis	0			
DK111C;Hypertrofi af glandula sublingualis	0			
DK112;Betændelse i spytkirtel	0			
DK112A;Purulent parotitis	0			
DK112B;Kronisk parotitis	0			
DK112C;Akut parotitis	0			
DK112D;Sialodochitis	0			
DK113;Absces i spytkirtel	0			

Supplement 2 Nielsen et al. Mortality after paediatric emergency calls for patients with and without pre-existing comorbidity (2023)

DK113A;Abscessus glandulae submandibularis	0			
DK113B;Abscessus glandulae sublingualis	0			
DK113C;Abscessus glandulae submandibularis	0			
DK113D;Abscessus glandulae parotideae	0			
DK114;Fistel i spytkirtel	0			
DK114A;Fistel i glandula parotidea	0			
DK115;Spytsten	0			
DK115A;Spytsten i glandula parotidea	0			
DK116;Mukocele i spytkirtel	0			
DK116A;Cystis glandulae sublingualis	0			
DK116B;Cystis glandulae parotideae	0			
DK116C;Ranula	0			
DK117;Sekretionsforstyrrelse i spytkirtel	0			
DK117A;Hyposalivatio	0			
DK117B;Salivatio excessiva	0			
DK117C;Hypersalivatio	0			
DK117D;Mundtørhed forårsaget af sekretionsforstyrrelse i spytkirtler	0			
DK118;Anden sygdom i spytkirtel	0			
DK118A;Erhvervet tillukning af udførselsgang fra spytkirtel	0			
DK118B;Erhvervet deformering af udførselsgang fra spytkirtel	0			
DK118C;Godartet lymfoepitelial læsion i spytkirtel	0			
DK118E;Sialectasia	0			
DK118F;Sialometaplasia necroticans	0			
DK118G;Sialose	0			
DK118H;Stenosis ductus salivarii UNS	0			
DK118I;Strictura ductus salivarii UNS	0			
DK118J;Forsnævring af udførselsgang fra glandula parotidea	0			
DK119;Sygdom i spytkirtel UNS	0			
DK12;Betændelse i mundslimhinden og beslægtede sygdomme	0			
DK120;Aftøs mundbetændelse	0			
DK120A;Aphthae oris recurrentes	0			
DK120B;Bednars after	0			

Supplement 2 Nielsen et al. Mortality after paediatric emergency calls for patients with and without pre-existing comorbidity (2023)

DK120C;Periadenitis mucosa necrotica recurrens	0			
DK120D;Stomatitis herpetiformis	0			
DK121;Anden form for stomatitis	0			
DK121A;Ulcerøs mundbetændelse	0			
DK121B;Stomatitis UNS	0			
DK121C;Stomatitis catarrhalis	0			
DK122;Flegmone eller absces i munden	0			
DK122A;Abscessus submandibularis	0			
DK122B;Absces i munden	0			
DK13;Andre sygdomme i læber og mundslimhinde	0			
DK130;Sygdom i læberne	0			
DK130A;Abscessus labii oris	0			
DK130B;Cheilitis exfoliativa	0			
DK130C;Cheilitis UNS	0			
DK130D;Cheilitis glandularis	0			
DK130E;Cheilitis angularis IKA	0			
DK130F;Cheilodynia	0			
DK130G;Cystis labii oris	0			
DK130H;Fissura labii oris	0			
DK130I;Fistula labii oris	0			
DK130J;Hypertrofi af læber	0			
DK131;Kindbidning og læbebidning	0			
DK131A;Læbebidning	0			
DK131B;Kindbidning	0			
DK132;Leukoplakia eller anden forstyrrelse i mundslimhinden	0			
DK132A;Acanthosis linguae	0			
DK132B;Erythroplakia oris	0			
DK132C;Erythroplakia linguae	0			
DK132D;Erythroplakia buccae	0			
DK132E;Leukoedema oris	0			
DK132F;Leukoedema linguae	0			
DK132G;Leukokeratosis nicotina palati	0			

DK132H;Leukoplakia oris	0		
DK132I;Leukoplakia linguae	0		
DK132J;Leukoplakia buccae	0		
DK132K;Leukoplakia gingivae	0		
DK132M;Misfarvning af mundslimhinden UNS	0		
DK132N;Hyperplasia epithelialis focalis (FEH)	0		
DK133;Håret leukoplaki	0		
DK134;Granulom eller granulomlignende sygdom i mundslimhinden	0		
DK134A;Granuloma purulenta mucosae oris	0		
DK134B;Granuloma mucosae oris	0		
DK134C;Granuloma eosinophilica mucosae oris	0		
DK134D;Xanthoma verrucosa mucosae oris	0		
DK134E;Cheilitis granulomatosa	0		
DK135;Submukøs fibrose i munden	0		
DK135A;Submukøs fibrose i tungen	0		
DK136;Hypertrophia irritativa mucosae oris	0		
DK137;Anden eller ikke nærmere specificeret sygdom i mundslimhinde	0		
DK137A;Ulcus mucosae buccae	0		
DK137B;Ulcus mucosae palati	0		
DK137C;Hyperplasia papilliformis mucosae oris palati	0		
DK137D;Allergia mucosae oris	0		
DK137E;Orodynia	0		
DK137F;Ulcus mucosae oris UNS	0		
DK14;Sygdomme i tungen	0		
DK140;Glossitis	0		
DK140A;Absces i tungen	0		
DK140B;Papillitis linguae	0		
DK140C;Traumatisk ulcus på tungen	0		
DK140D;Ulcus på tungen UNS	0		
DK141;Lingua geographica	0		
DK142;Glossitis rhombica mediana	0		
DK143;Hypertrofi af tungepapiller	0		

Supplement 2 Nielsen et al. Mortality after paediatric emergency calls for patients with and without pre-existing comorbidity (2023)

DK143A;Lingua (villosa) nigra	0		
DK144;Atrofi af tungepapiller	0		
DK144A;Atrofisk glottitis	0		
DK145;Lingua plicata	0		
DK145A;Erhvervede fissurer i tungen	0		
DK145B;Lingua scrotalis	0		
DK146;Smerter i tungen	0		
DK146A;Glossopyrosis	0		
DK148;Anden sygdom i tungen	0		
DK148A;Atrofi af tungen	0		
DK148B;Hemiatrofi af tungen	0		
DK148C;Hypertrofi af tungen	0		
DK148D;Erhvervet makroglossi	0		
DK148F;Eosinofilt ulcus på tungen	0		
DK148G;Lingua indentata	0		
DK149;Sygdom i tunge UNS	0		
DK20;Betændelse i spiserøret		1	
DK209;Øsofagitis UNS		1	
DK209A;Absces i spiserøret		1	
DK209B;Peptisk øsofagitis		1	
DK209C;Øsofagitis forårsaget af kemikalie		1	
DK209D;Eosinofil øsofagitis		1	
DK21;Gastro-øsofageal refluks		1	
DK210;Gastro-øsofageal refluks med øsofagitis		1	
DK219;Gastro-øsofageal refluks uden øsofagitis		1	
DK219A;Søvnrelateret gastro-øsofageal refluks		1	
DK219B;Gastro-øsofageal refluks UNS		1	
DK22;Andre sygdomme i spiserøret		1	
DK220;Cardia-akalasi		1	
DK221;Ulcus i spiserøret		1	
DK221A;Erosion i spiserøret		1	
DK221B;Peptisk ulcus i spiserøret		1	

DK221C;Ulcus i spiserøret forårsaget af svampeinfektion		1	
DK221D;Ulcus i spiserøret forårsaget af kemikalie		1	
DK221E;Ulcus i spiserøret forårsaget af lægemiddel		1	
DK221F;Ulcerativ øsofagitis UNS		1	
DK222;Obstruktion af spiserøret		1	
DK222A;Kompression af spiserøret		1	
DK222B;Stenose i spiserøret		1	
DK222C;Striktur i spiserøret		1	
DK223;Perforation af spiserøret		1	
DK223A;Ruptur af spiserøret		1	
DK224;Spiserørsdyskinesi		1	
DK224A;Øsofaguspasmer		1	
DK225;Erhvervet øsofagusdivertikel		1	
DK225A;Traktionsdivertikel i spiserøret		1	
DK225B;Pulsionsdivertikel i øsofagus		1	
DK226;Mallory-Weiss' syndrom		1	
DK227;Barretts øsofagus		1	
DK228;Anden sygdom i spiserøret		1	
DK228A;Cystis oesophagi		1	
DK228B;Dilatatio oesophagi		1	
DK228C;Fistula tracheo-oesophagealis		1	
DK228D;Fistula broncho-oesophagealis		1	
DK228E;Functio laesa oesophagi		1	
DK228F;Blødning i spiserøret UNS		1	
DK228G;Leukoplakia oesophagi		1	
DK228H;Megaloøsofagus		1	
DK228I;Presbyøsofagus		1	
DK229;Sygdom i øsofagus UNS		1	
DK23;Sygdom i øsofagus ved sygdom klassificeret andetsteds		1	
DK230;Tuberkuløs øsofagitis		1	
DK231;Megaøsofagus ved Chagas' sygdom		1	
DK238;Forstyrrelser i øsofagus ved anden sygdom klassificeret andetsteds		1	

DK25;Mavesår		1	
DK250;Akut mavesår med blødning		1	
DK250A;Akut ulcus i cardia med blødning		1	
DK250B;Akut ulcus i corpus ventriculi med blødning		1	
DK250C;Akut jukstapylorisk ulcus med blødning		1	
DK250D;Akut præpylorisk ulcus med blødning		1	
DK250E;Akut pylorusulcus med blødning		1	
DK250F;Exulceratio simplex Dieulafoy med blødning		1	
DK251;Akut mavesår med perforation		1	
DK251A;Akut ulcus i cardia med perforation		1	
DK251B;Akut pylorusulcus med perforation		1	
DK251C;Akut ulcus i corpus ventriculi med perforation		1	
DK251D;Akut jukstapylorisk ulcus med perforation		1	
DK251E;Akut præpylorisk ulcus med perforation		1	
DK252;Akut mavesår med blødning og perforation		1	
DK252A;Akut ulcus i cardia med blødning og perforation		1	
DK252B;Akut pylorusulcus med blødning og perforation		1	
DK252C;Akut ulcus i corpus ventriculi med blødning og perforation		1	
DK252D;Akut jukstapylorisk ulcus med blødning og perforation		1	
DK252E;Akut præpylorisk ulcus med blødning og perforation		1	
DK253;Akut mavesår uden blødning eller perforation		1	
DK253A;Akut ulcus i cardia uden blødning eller perforation		1	
DK253B;Akut pylorusulcus uden blødning eller perforation		1	
DK253C;Akut ulcus i corpus ventriculi uden blødning eller perforation		1	
DK253D;Akut jukstapylorisk ulcus uden blødning eller perforation		1	
DK253E;Akut præpylorisk ulcus uden blødning eller perforation		1	
DK254;Kronisk eller ikke specificeret mavesår med blødning		1	
DK254A;Kronisk ulcus i cardia med blødning		1	
DK254B;Ulcus UNS i cardia med blødning		1	
DK254C;Kronisk pylorusulcus med blødning		1	
DK254D;Pylorusulcus UNS med blødning		1	
DK254E;Kronisk ulcus i corpus ventriculi med blødning		1	

DK254F;Ulcus UNS i corpus ventriculi med blødning		1	
DK254G;Kronisk jukstapylorisk ulcus med blødning		1	
DK254H;Jukstapylorisk ulcus UNS med blødning		1	
DK254I;Kronisk præpylorisk ulcus med blødning		1	
DK254J;Præpylorisk ulcus UNS med blødning		1	
DK255;Kronisk eller ikke specificeret mavesår med perforation		1	
DK255A;Kronisk ulcus i cardia med perforation		1	
DK255B;Ulcus UNS i cardia med perforation		1	
DK255C;Kronisk pylorusulcus med perforation		1	
DK255D;Pylorusulcus UNS med perforation		1	
DK255E;Kronisk ulcus i corpus ventriculi med perforation		1	
DK255F;Ulcus UNS i corpus ventriculi med perforation		1	
DK255G;Kronisk jukstapylorisk ulcus med perforation		1	
DK255H;Jukstapylorisk ulcus UNS med perforation		1	
DK255I;Kronisk præpylorisk ulcus med perforation		1	
DK255J;Præpylorisk ulcus UNS med perforation		1	
DK256;Kronisk eller ikke specificeret mavesår med blødning og perforation		1	
DK256A;Kronisk ulcus i cardia med blødning og perforation		1	
DK256B;Ulcus UNS i cardia med blødning og perforation		1	
DK256C;Kronisk pylorusulcus med blødning og perforation		1	
DK256D;Pylorusulcus UNS med blødning og perforation		1	
DK256E;Kronisk ulcus i corpus ventriculi med blødning og perforation		1	
DK256F;Ulcus UNS i corpus ventriculi med blødning og perforation		1	
DK256G;Kronisk jukstapylorisk ulcus med blødning og perforation		1	
DK256H;Jukstapylorisk ulcus UNS med blødning og perforation		1	
DK256I;Kronisk præpylorisk ulcus med blødning og perforation		1	
DK256J;Præpylorisk ulcus UNS med blødning og perforation		1	
DK257;Kronisk mavesår uden blødning eller perforation		1	
DK257A;Kronisk ulcus i cardia uden blødning eller perforation		1	
DK257B;Kronisk pylorusulcus uden blødning eller perforation		1	
DK257C;Kronisk ulcus i corpus ventriculi uden blødning eller perforation		1	
DK257D;Kronisk jukstapylorisk ulcus uden blødning eller perforation		1	

DK257E;Kronisk præpylorisk ulcus uden blødning eller perforation		1	
DK259;Mavesår UNS uden blødning eller perforation		1	
DK259A;Mavesår UNS		1	
DK26;Sår på tolvfingertarmen		1	
DK260;Akut duodenalulcus med blødning		1	
DK260A;Akut postpylorisk ulcus med blødning		1	
DK261;Akut duodenalulcus med perforation		1	
DK261A;Akut postpylorisk ulcus med perforation		1	
DK262;Akut duodenalulcus med blødning og perforation		1	
DK262A;Akut postpylorisk ulcus med blødning og perforation		1	
DK263;Akut duodenalulcus uden blødning eller perforation		1	
DK263A;Akut postpylorisk ulcus uden blødning eller perforation		1	
DK264;Kronisk eller ikke specificeret duodenalulcus med blødning		1	
DK264A;Kronisk duodenalulcus med blødning		1	
DK264B;Duodenalulcus UNS med blødning		1	
DK264C;Kronisk postpylorisk ulcus med blødning		1	
DK264D;Postpylorisk ulcus UNS med blødning		1	
DK265;Kronisk eller ikke specificeret duodenalulcus med perforation		1	
DK265A;Kronisk duodenalulcus med perforation		1	
DK265B;Duodenalulcus UNS med perforation		1	
DK265C;Kronisk postpylorisk ulcus med perforation		1	
DK265D;Postpylorisk ulcus UNS med perforation		1	
DK266;Kronisk eller ikke specificeret duodenalulcus med blødning og perforation		1	
DK266A;Kronisk duodenalulcus med blødning og perforation		1	
DK266B;Duodenalulcus UNS med blødning og perforation		1	
DK266C;Kronisk postpylorisk ulcus med blødning og perforation		1	
DK266D;Postpylorisk ulcus UNS med blødning og perforation		1	
DK267;Kronisk duodenalulcus uden blødning eller perforation		1	
DK267A;Kronisk postpylorisk ulcus uden blødning eller perforation		1	
DK267B;Ulcus duodeni, kronisk uden blødning eller perforation (findes ikke i SKS)		1	
DK269;Duodenalulcus UNS uden blødning eller perforation		1	
DK27;Mavesår og sår på tolvfingertarm		1	

DK270;Akut gastroduodenalt ulcus med blødning		1	
DK271;Akut gastroduodenalt ulcus med perforation		1	
DK272;Akut gastroduodenalt ulcus med blødning og perforation		1	
DK273;Akut gastroduodenalt ulcus uden blødning eller perforation		1	
DK274;Kronisk eller ikke specificeret gastroduodenalt ulcus med blødning		1	
DK275;Kronisk eller ikke specificeret gastroduodenalt ulcus med perforation		1	
DK276;Kronisk eller ikke specificeret gastroduodenalt ulcus med blødning og perforation		1	
DK277;Kronisk gastroduodenalt ulcus uden blødning eller perforation		1	
DK279;Gastroduodenalt ulcus UNS uden blødning eller perforation		1	
DK28;Recidivsår efter gastroenterostomi		1	
DK280;Akut gastrointestinalt sår med blødning		1	
DK280A;Akut gastrojejunalt sår med blødning		1	
DK280B;Akut gastrokolisk sår med blødning		1	
DK281;Akut gastrointestinalt sår med perforation		1	
DK281A;Akut gastrojejunalt sår med perforation		1	
DK281C;Akut gastrokolisk sår med perforation		1	
DK282;Akut gastrointestinalt sår med blødning og perforation		1	
DK282A;Akut gastrojejunalt sår med blødning og perforation		1	
DK282C;Akut gastrokolisk sår med blødning og perforation		1	
DK283;Akut gastrointestinalt sår uden blødning og perforation		1	
DK283A;Akut gastrojejunalt sår uden blødning og perforation		1	
DK283C;Akut gastrokolisk sår uden blødning og perforation		1	
DK284;Kronisk eller ikke specificeret gastrointestinalt sår med blødning		1	
DK284A;Kronisk gastrojejunalt sår med blødning		1	
DK284B;Gastrojejunalt sår UNS med blødning		1	
DK284C;Kronisk gastrokolisk sår med blødning		1	
DK284D;Gastrokolisk sår UNS med blødning		1	
DK285;Kronisk eller ikke specificeret gastrointestinalt sår med perforation		1	
DK285A;Kronisk gastrojejunalt sår med perforation		1	
DK285B;Gastrojejunalt sår UNS med perforation		1	
DK285C;Kronisk gastrokolisk sår med perforation		1	
DK285D;Gastrokolisk sår UNS med perforation		1	

DK286;Kronisk eller ikke specificeret gastrointestinalt sår med blødning og perforation		1	
DK286A;Kronisk gastrojejunalt sår med blødning og perforation		1	
DK286B;Gastrojejunalt sår UNS med blødning og perforation		1	
DK286C;Kronisk gastrokolisk sår med blødning og perforation		1	
DK286D;Gastrokolisk sår UNS med blødning og perforation		1	
DK287;Kronisk gastrointestinalt sår uden blødning og perforation		1	
DK287A;Kronisk gastrojejunalt sår uden blødning og perforation		1	
DK287B;Kronisk gastrokolisk sår uden blødning og perforation		1	
DK289;Gastrointestinalt sår UNS uden blødning eller perforation		1	
DK29;Gastroduodenitis		1	
DK290;Akut blødende gastritis		1	
DK291;Anden form for akut mavekatar		1	
DK292;Alkoholisk gastritis		1	
DK293;Kronisk superficiel gastritis		1	
DK294;Kronisk atrofisk gastritis		1	
DK295;Kronisk gastritis UNS		1	
DK296;Anden form for mavekatar		1	
DK296A;Hypertrofisk gastritis		1	
DK296B;Granulomatøs gastritis		1	
DK297;Mavekatar UNS		1	
DK298;Duodenitis		1	
DK298A;Akut blødende duodenitis		1	
DK299;Gastroduodenitis UNS		1	
DK30;Funktionelt fordøjelsesbesvær		1	
DK309;Funktionel dyspepsi UNS		1	
DK31;Andre sygdomme i mavesæk og duodenum		1	
DK310;Akut dilatation af mavesækken		1	
DK310A;Akut atoni af mavesækken		1	
DK311;Hypertrofisk pylorostenose		1	
DK311A;Pylorostenose UNS		1	
DK312;Timeglasformet striktur eller stenose i mavesækken		1	
DK312A;Stenose i mavesækken		1	

Supplement 2 Nielsen et al. Mortality after paediatric emergency calls for patients with and without pre-existing comorbidity (2023)

DK312B;Stenose i cardia		1	
DK312C;Striktur af mavesækken		1	
DK313;Pylorospasme IKA		1	
DK314;Divertikel i mavesækken		1	
DK314A;Divertikel i cardia		1	
DK315;Obstruktion af duodenum		1	
DK315A;Stenose i duodenum		1	
DK315B;Striktur af duodenum		1	
DK316;Fistel fra mavesækken eller duodenum		1	
DK316A;Fistel fra mavesækken UNS		1	
DK316B;Gastrojejunal fistel		1	
DK316C;Fistel fra duodenum		1	
DK316D;Gastrokolisk fistel		1	
DK316E;Gastrointestinal fistel		1	
DK317;Polyp i mavesækken eller duodenum		1	
DK318;Anden sygdom i ventrikel eller duodenum		1	
DK318A;Achylia gastrica		1	
DK318B;Dysfunctio pylori		1	
DK318C;Ectasia ventriculi		1	
DK318D;Gastroptosis		1	
DK318E;Hyperchlorhydria		1	
DK318F;Hypersecretio gastrica		1	
DK318G;Prolapsus mucosae pylori		1	
DK318H;Retentio ventriculi		1	
DK318J;Timeglasformet kontraktur i mavesækken		1	
DK319;Sygdom i mavesækken eller duodenum UNS		1	
DK35;Akut blindtarmsbetændelse		1	
DK352;Akut appendicitis med generaliseret peritonitis		1	
DK353;Akut appendicitis med lokaliseret peritonitis		1	
DK353A;Akut appendicitis med peritoneal absces		1	
DK353B;Akut appendicitis med periappendikulær absces		1	
DK358;Anden og ikke specificeret akut appendicitis		1	

DK358A;Akut appendicitis UNS		1	
DK358B;Akut gangrænøs appendicitis		1	
DK358C;Akut flegmonøs appendicitis		1	
DK359;Akut blindtarmsbetændelse uden specifikation (findes ikke i SKS)		1	
DK359B;Appendicitis acuta phlegmonosa (findes ikke i SKS)		1	
DK36;Andre former for blindtarmsbetændelse		1	
DK369;Kronisk eller recidiverende appendicitis		1	
DK37;Blindtarmsbetændelse UNS		1	
DK379;Appendicitis UNS		1	
DK38;Andre sygdomme i blindtarmen		1	
DK380;Lymfoid hyperplasi af appendix		1	
DK381;Konkrement i appendix		1	
DK381A;Koprolit i appendix		1	
DK382;Appendixdivertikel		1	
DK383;Appendixfistel		1	
DK388;Anden sygdom i blindtarmen		1	
DK388A;Blindtarmscyste		1	
DK388B;Invagination af blindtarmen		1	
DK388C;Blindtarmsmukocele		1	
DK388D;Neuroma appendicis		1	
DK389;Sygdom i blindtarmen UNS		1	
DK40;Lyskebrok		1	
DK400;Bilateralt ingvinalhernie med ileus uden gangræn		1	
DK401;Bilateralt ingvinalhernie med gangræn		1	
DK402;Bilateralt ingvinalhernie uden ileus eller gangræn		1	
DK402A;Bilateralt ingvinalhernie UNS		1	
DK403;Unilateralt ingvinalhernie med ileus uden gangræn		1	
DK403A;Unilateralt inkarcereret ingvinalhernie med ileus uden gangræn		1	
DK403B;Unilateralt irreponibelt ingvinalhernie med ileus uden gangræn		1	
DK404;Unilateralt ingvinalhernie med gangræn		1	
DK409;Ingvinalhernie UNS uden ileus eller gangræn		1	
DK41;Lårbrok		1	

DK410;Bilateralt femoralhernie med ileus uden gangræn		1	
DK411;Bilateralt femoralhernie med gangræn		1	
DK412;Bilateralt femoralhernie uden ileus eller gangræn		1	
DK412A;Bilateralt femoralhernie UNS		1	
DK413;Unilateralt femoralhernie med ileus uden gangræn		1	
DK413A;Unilateralt inkarcereret femoralhernie med ileus uden gangræn		1	
DK413B;Unilateralt irreponibelt femoralhernie med ileus uden gangræn		1	
DK414;Unilateralt femoralhernie med gangræn		1	
DK419;Femoralhernie UNS uden ileus eller gangræn		1	
DK42;Navlebrok		1	
DK420;Umbilikalhernie med ileus uden gangræn		1	
DK420A;Inkarcereret umbilikalhernie		1	
DK420B;Irreponibelt umbilikalhernie		1	
DK421;Umbilikalhernie med gangræn		1	
DK429;Umbilikalhernie uden ileus eller gangræn		1	
DK43;Bugvægsbrok		1	
DK430;Incisionalhernie med ileus uden gangræn		1	
DK431;Incisionalhernie med gangræn		1	
DK432;Incisionalhernie uden ileus eller gangræn		1	
DK433;Parastomalt hernie med ileus uden gangræn		1	
DK434;Parastomalt hernie med gangræn		1	
DK435;Parastomalt hernie uden ileus eller gangræn		1	
DK435A;Kolostomihernie uden ileus eller gangræn		1	
DK435B;Ileostomihernie uden ileus eller gangræn		1	
DK435C;Urostomihernie uden ileus eller gangræn		1	
DK435D;Andet parastomalt hernie uden ileus eller gangræn		1	
DK436;Andet ventralhernie med ileus uden gangræn		1	
DK436A;Inkarcereret ventralhernie uden gangræn		1	
DK436B;Irreponibelt ventralhernie uden gangræn		1	
DK437;Andet ventralhernie med gangræn		1	
DK439;Ventralhernie UNS uden ileus eller gangræn		1	
DK44;Brok i mellemgulvet		1	

DK440;Diafragmahernie med ileus uden gangræn		1	
DK440A;Inkarcereret diafragmahernie uden gangræn		1	
DK440B;Irreponibelt diafragmahernie uden gangræn		1	
DK441;Diafragmahernie med gangræn		1	
DK449;Diafragmahernie uden ileus eller gangræn		1	
DK45;Andre former for brok i bugvæggen		1	
DK450;Andet abdominalhernie med ileus uden gangræn		1	
DK450B;Navlesnorshernie med ileus uden gangræn		1	
DK450C;Intraabdominalt hernie med ileus uden gangræn		1	
DK450D;Hernia ischiadica med ileus uden gangræn		1	
DK450E;Hernia ischiorectalis med ileus uden gangræn		1	
DK450F;Hernia linea alba med ileus uden gangræn		1	
DK450G;Lumbalhernie med ileus uden gangræn		1	
DK450H;Hernia mesenterialis med ileus uden gangræn		1	
DK450I;Hernia obturatoria med ileus uden gangræn		1	
DK450J;Hernia omentalis med ileus uden gangræn		1	
DK450K;Hernia perinealis med ileus uden gangræn		1	
DK450L;Hernia pudendalis med ileus uden gangræn		1	
DK450M;Hernia retroperitonealis med ileus uden gangræn		1	
DK451;Andet abdominalhernie med gangræn		1	
DK451B;Navlesnorshernie med gangræn		1	
DK451C;Intraabdominalt hernie med gangræn		1	
DK451D;Hernia ischiadica med gangræn		1	
DK451E;Hernia ischiorectalis med gangræn		1	
DK451F;Hernia lineae albae med gangræn		1	
DK451G;Lumbalhernie med gangræn		1	
DK451H;Hernia mesenterialis med gangræn		1	
DK451I;Hernia obturatoria med gangræn		1	
DK451J;Hernia omentalis med gangræn		1	
DK451K;Hernia perinealis med gangræn		1	
DK451L;Hernia pudendalis med gangræn		1	
DK451M;Hernia retroperitonealis med gangræn		1	

DK458;Andet abdominalhernie uden ileus eller gangræn		1	
DK458B;Navlesnorshernie uden ileus eller gangræn		1	
DK458C;Intraabdominalt hernie uden ileus eller gangræn		1	
DK458D;Hernia ischiadica uden ileus eller gangræn		1	
DK458E;Hernia ischiorectalis uden ileus eller gangræn		1	
DK458F;Hernia lineae albae uden ileus eller gangræn		1	
DK458G;Lumbalhernie uden ileus eller gangræn		1	
DK458H;Hernia mesenterialis uden ileus eller gangræn		1	
DK458I;Hernia obturatoria uden ileus eller gangræn		1	
DK458J;Hernia omentalis uden ileus eller gangræn		1	
DK458K;Hernia perinealis uden ileus eller gangræn		1	
DK458L;Hernia pudendalis uden ileus eller gangræn		1	
DK458M;Hernia retroperitonealis uden ileus eller gangræn		1	
DK46;Abdominalt brok ikke nærmere specificeret		1	
DK460;Abdominalhernie UNS med ileus uden gangræn		1	
DK460A;Inkarcereret abdominalhernie UNS uden gangræn		1	
DK460B;Irreponibelt abdominalhernie UNS uden gangræn		1	
DK461;Abdominalhernie UNS med gangræn		1	
DK469;Abdominalhernie UNS uden ileus eller gangræn		1	
DK469A;Abdominalhernie UNS		1	
DK50;Crohns sygdom		1	
DK500;Crohns sygdom i tyndtarmen		1	
DK500A;Crohns sygdom i duodenum		1	
DK500B;Crohns sygdom i ileum		1	
DK500C;Crohns sygdom i jejunum		1	
DK500D;Ileitis terminalis		1	
DK501;Crohns sygdom i tyktarmen		1	
DK501D;Crohns sygdom i endetarmen		1	
DK508;Anden form for Crohns sygdom		1	
DK508A;Crohns sygdom i både tyndtarmen og tyktarmen		1	
DK508C;Crohns sygdom med forandringer i mundhulen		1	
DK508D;Crohns sygdom med ileocækal lokalisation		1	

DK509;Crohns sygdom UNS		1	
DK51;Ulcerøs colitis		1	
DK510;Ulcerøs pancolitis		1	
DK512;Ulcerøs proktitis		1	
DK513;Ulcerøs proktosigmoiditis		1	
DK514;Inflammatoriske polypper		1	
DK515;Venstresidig ulcerøs colitis		1	
DK515A;Venstresidig proktocolitis		1	
DK515B;Venstresidig hemicolitis		1	
DK518;Anden form for ulcerøs colitis		1	
DK518B;Ulcerøs colitis med forandringer i mundhulen		1	
DK519;Ulcerøs colitis UNS		1	
DK52;Andre ikke-infektiøse betændelsestilstande i mavesækken og tarmene		1	
DK520;Gastroenteritis eller colitis forårsaget af stråling		1	
DK520A;Colitis forårsaget af stråling		1	
DK520B;Gastroenteritis forårsaget af stråling		1	
DK521;Toksisk gastroenteritis eller colitis		1	
DK521A;Toksisk colitis		1	
DK521B;Toksisk enteritis		1	
DK521C;Toksisk gastroenteritis		1	
DK522;Gastroenteritis eller colitis forårsaget af allergi eller fødemiddel		1	
DK522A;Fødemiddelallergi UNS		1	
DK522B;Allergia gastrointestinalis (fødeallergi) (findes ikke i SKS)		1	
DK522C;Colitis forårsaget af fødemiddelallergi		1	
DK522D;Enteritis forårsaget af fødemiddelallergi		1	
DK522E;Enterocolitis forårsaget af fødemiddelallergi		1	
DK522F;Gastroenteritis forårsaget af fødemiddelallergi		1	
DK523;Ikke nærmere specificeret colitis		1	
DK528;Anden form for ikke-infektiøs gastroenteritis eller colitis		1	
DK528A;Eosinofil gastritis		1	
DK528B;Eosinofil gastroenteritis		1	
DK528C;Pouchitis		1	

Supplement 2 Nielsen et al. Mortality after paediatric emergency calls for patients with and without pre-existing comorbidity (2023)

DK528D;Mikroskopisk colitis		1	
DK528D1;Kollagen colitis		1	
DK528D2;Lymfocytær colitis		1	
DK529;Anden ikke-infektiøs gastroenteritis eller colitis UNS		1	
DK529A;Ikke-infektiøs colitis UNS		1	
DK529B;Ikke-infektiøs diaré UNS		1	
DK529B1;Kemoterapi-induceret diaré		1	
DK529C;Ikke-infektiøs enteritis UNS		1	
DK529D;Ikke-infektiøs enterocolitis UNS		1	
DK529E;Ikke-infektiøs gastroenteritis UNS		1	
DK529F;Ikke-infektiøs ileitis UNS		1	
DK529G;Ikke-infektiøs jejunitis UNS		1	
DK529H;Ikke-infektiøs sigmoiditis UNS		1	
DK55;Karsygdomme i tarm		1	
DK550;Akut karsygdom i tarm		1	
DK550A;Akut fulminant iskæmisk colitis		1	
DK550B;Subakut iskæmisk colitis		1	
DK550C;Mesenterialemboli		1	
DK550D;Akut tarminfarkt		1	
DK550E;Mesenterialinfarkt		1	
DK550F;Akut tyndtarmsiskæmi		1	
DK550H;Mesenterialtrombose		1	
DK551;Kronisk karsygdom i tarm		1	
DK551A;Mesenteriel aterosklerose		1	
DK551B;Kronisk iskæmisk colitis		1	
DK551C;Kronisk iskæmisk enteritis		1	
DK551D;Kronisk iskæmisk enterocolitis		1	
DK551E;Vaskulær insufficiens i mesenteriet		1	
DK551F;Iskæmisk tarmstriktur		1	
DK552;Angiodysplasi i tyktarmen		1	
DK558;Anden karsygdom i tarmen		1	
DK558A;Angiodysplasi i tyndtarmen		1	

DK558B;Angiodysplasi i duodenum		1	
DK558C;Angiodysplasi i jejunum		1	
DK558D;Angiodysplasi i ileum		1	
DK559;Karsygdom i tarm UNS		1	
DK56;Paralytisk ileus og tarmobstruktion ikke forårsaget af hernie		1	
DK560;Paralytisk ileus		1	
DK560A;Paralytisk tyktarmsileus		1	
DK560B;Paralytisk tyndtarmsileus		1	
DK561;Invagination		1	
DK561A;Invaginatio colicolica		1	
DK561B;Invaginatio ileocolica		1	
DK561C;Invaginatio ileoiliaca		1	
DK561D;Invaginatio recti		1	
DK562;Volvulus		1	
DK562A;Torsion af Meckels divertikel		1	
DK562B;Tyktarmsvolvulus		1	
DK562C;Tyndtarmsvolvulus		1	
DK563;Galdestensileus		1	
DK564;Anden form for tarmobstruktion		1	
DK564A;Koprolitiasis med tarmobstruktion		1	
DK564B;Enterolitiasis med tarmobstruktion		1	
DK565;Tarmadhærencer med tarmobstruktion		1	
DK565A;Abdominale adhærencer med tarmobstruktion		1	
DK565B;Mesenteriale adhærencer med tarmobstruktion		1	
DK565C;Omentadhærencer med tarmobstruktion		1	
DK565D;Peritoneale adhærencer med tarmobstruktion		1	
DK565E;Strengileus		1	
DK566;Anden eller ikke specificeret tarmobstruktion		1	
DK566A;Tyktarmsobstruktion UNS		1	
DK566B;Tyndtarmsobstruktion UNS		1	
DK566C;Subileus		1	
DK566D;Mesenterietorsion		1	

Supplement 2 Nielsen et al. Mortality after paediatric emergency calls for patients with and without pre-existing comorbidity (2023)

DK566E;Omenttorsion		1	
DK566F;Tyndtarmsstenose		1	
DK566G;Tyktarmsstenose		1	
DK566H;Ogilvies syndrom		1	
DK567;Ileus UNS		1	
DK57;Divertikler og betændelse i divertikler i tarmen		1	
DK570;Divertikulose eller divertikulitis i tyndtarmen med perforation eller absces		1	
DK570A;Divertikulitis i tyndtarmen med absces		1	
DK570B;Divertikulitis i tyndtarmen med perforation		1	
DK570C;Divertikulitis i tyndtarmen med peritonitis		1	
DK571;Divertikulose eller divertikulitis i tyndtarmen uden perforation eller absces		1	
DK571A;Divertikulitis i tyndtarmen uden perforation		1	
DK571B;Divertikulitis i tyndtarmen UNS		1	
DK572;Divertikulose eller divertikulitis i tyktarmen med perforation eller absces		1	
DK572A;Divertikulitis i tyktarmen med absces		1	
DK572B;Divertikulitis i tyktarmen med perforation		1	
DK572C;Divertikulitis i tyktarmen med peritonitis		1	
DK573;Divertikulose eller divertikulitis i tyktarmen uden perforation eller absces		1	
DK573A;Divertikulitis i tyktarmen uden perforation		1	
DK573B;Divertikulitis i tyktarmen UNS		1	
DK573C;Divertikler i tyktarmen uden perforation		1	
DK573D;Divertikler i tyktarmen UNS		1	
DK573E;Pericolitis		1	
DK573F;Perisigmoiditis		1	
DK574;Divertikler i både tyndtarmen og tyktarmen med perforation eller absces		1	
DK574A;Divertikler i både tyndtarmen og tyktarmen med perforation		1	
DK575;Divertikler i både tyndtarmen og tyktarmen uden perforation eller absces		1	
DK578;Divertikler uden angivelse af lokalisation med perforation eller absces		1	
DK579;Divertikler uden angivelse af lokalisation uden perforation eller absces		1	
DK579A;Peridiverticulitis UNS		1	
DK58;Irritabel tyktarm	0		
DK580;Irritabel tyktarm med diaré	0		

Supplement 2 Nielsen et al. Mortality after paediatric emergency calls for patients with and without pre-existing comorbidity (2023)

DK589;Irritabel tyktarm uden diaré	0		
DK59;Andre forstyrrelser i tarmfunktionen	0		
DK590;Forstoppelse	0		
DK590A;Kronisk forstoppelse	0		
DK591;Funktionel diaré	0		
DK592;Neurogen tarmfunktionsforstyrrelse IKA		1	
DK593;Megacolon IKA		1	
DK593B;Dilatatio coli		1	
DK593C;Toksisk megacolon IKA		1	
DK593D;Toksisk megasigmoideum IKA		1	
DK594;Analspasme		1	
DK594A;Proctalgia fugax		1	
DK598;Anden forstyrrelse i tarmfunktionen		1	
DK598A;Atonia coli		1	
DK598B;Atonia intestini		1	
DK598C;Contractura recti		1	
DK598D;Sterkoraldiaré		1	
DK598E;Dysfunctio coli		1	
DK599;Forstyrrelse i tarmfunktionen UNS		1	
DK60;Fissur og rift i og omkring endetarmen		1	
DK600;Akut analfissur		1	
DK601;Kronisk analfissur		1	
DK602;Analfissur UNS		1	
DK603;Analfistel		1	
DK603A;Ekstrasfinkterisk analfistel		1	
DK603B;Transsfinkterisk analfistel		1	
DK603C;Perianal fistel (findes ikke i SKS)		1	
DK603D;Intersfinkterisk analfistel		1	
DK603E;Submukøs analfistel		1	
DK603F;Suprasfinkterisk analfistel		1	
DK604;Rektalfistel		1	
DK604A;Rektokutan fistel		1	

DK605;Anorektal fistel		1	
DK61;Byld i og omkring endetarmen		1	
DK610;Analabsces		1	
DK610A;Perianalabsces		1	
DK610B;Analflegmone		1	
DK611;Rektalabsces		1	
DK611A;Perirektal absces		1	
DK612;Anorektal absces		1	
DK613;Iskiorektal absces		1	
DK614;Intersfinkterisk analabsces		1	
DK62;Andre sygdomme i endetarmen og endetarmsåbningen		1	
DK620;Anal polyp		1	
DK621;Rektal polyp		1	
DK622;Analprolaps		1	
DK623;Rektalprolaps		1	
DK624;Stenose i anus eller rektum		1	
DK624A;Striktur i anus		1	
DK624B;Striktur i rektum		1	
DK624C;Striktur i analsfinkteren		1	
DK624D;Stenose i anus		1	
DK624E;Stenose i rektum		1	
DK625;Blødning fra anus eller rektum		1	
DK625A;Blødning fra anus		1	
DK625B;Blødning fra rektum		1	
DK626;Ulcus i anus eller rektum		1	
DK626A;Ulcus i anus		1	
DK626B;Ulcus i rektum		1	
DK626C;Ulcus stercoralis		1	
DK627;Proktitis forårsaget af stråling		1	
DK628;Anden sygdom i anus eller rektum		1	
DK628A;Cystis ani		1	
DK628B;Cystis recti		1	

DK628C;Kraurosis ani		1	
DK628D;Leukoplakia ani		1	
DK628E;Leukoplakia recti		1	
DK628F;Megarektum		1	
DK628G;Melanosis recti		1	
DK628H;Ikke-traumatisk perforation af rektum		1	
DK628I;Periproctitis		1	
DK628J;Proctalgia		1	
DK628K;Proctitis allergica		1	
DK628L;Proktitis UNS		1	
DK628M;Relaxatio ani		1	
DK628N;Anal streptokokkose		1	
DK628P;Anal og perianal dysplasi		1	
DK628P1;Anal dysplasi		1	
DK628P1A;Let anal dysplasi		1	
DK628P1B;Moderat anal dysplasi		1	
DK628P1C;Svær anal dysplasi		1	
DK628P2;Perianal dysplasi		1	
DK628P2A;Let perianal dysplasi		1	
DK628P2B;Moderat perianal dysplasi		1	
DK628P2C;Svær perianal dysplasi		1	
DK629;Sygdom i anus eller rektum UNS		1	
DK63;Andre tarmsygdomme		1	
DK630;Tarmabsces		1	
DK631;Ikke-traumatisk perforation af tarmen		1	
DK631A;Spontan tarmruptur		1	
DK632;Tarmfistel		1	
DK632A;Caekalfistel		1	
DK632B;Colonfistel		1	
DK632C;Enterokolisk fistel		1	
DK632D;Ileorektal fistel		1	
DK632F;Enterokutan fistel		1	

DK632G;Enterorektal fistel		1	
DK632H;Jejunumfistel		1	
DK632I;Rektosigmoidal fistel		1	
DK632J;Sigmoideumfistel		1	
DK633;Tarmsår		1	
DK634;Nedsynkning af tarm		1	
DK634A;Proctoptosis		1	
DK634B;Ptosis coli		1	
DK634C;Ptosis intestini tenuis		1	
DK635;Colonpolyp UNS		1	
DK638;Anden tarmsygdom		1	
DK638A;Tarmgangræn		1	
DK638B;Tarmblødning UNS		1	
DK638C;Blødning fra mave-tarm-kanalen UNS		1	
DK638D;Melanosis coli		1	
DK638E;Prolapsus coli		1	
DK638F;Prolapsus intestini tenuis		1	
DK639;Tarmsygdom UNS		1	
DK64;Hæmorider og perianalt hæmatom		1	
DK640;Grad 1 hæmorider		1	
DK641;Grad 2 hæmorider		1	
DK642;Grad 3 hæmorider		1	
DK643;Grad 4 hæmorider		1	
DK644;Marisker		1	
DK645;Perianalt hæmatom		1	
DK648;Andre hæmorider		1	
DK649;Hæmorider UNS		1	
DK65;Bughindebetændelse		1	
DK650;Akut peritonitis		1	
DK650A;Abdominalabsces		1	
DK650B;Abscessus bursae omentalis		1	
DK650C;Abscessus fossae rectovesicalis		1	

DK650D;Abscessus intraperitonealis		1	
DK650E;Absces i mesenteriet		1	
DK650F;Absces i omentet		1	
DK650G;Bækkenabsces		1	
DK650H;Abscessus rectovaginalis		1	
DK650I;Abscessus retrocaecalis		1	
DK650J;Abscessus retroperitonealis		1	
DK650K;Abscessus subdiaphragmaticus		1	
DK650L;Abscessus subhepaticus		1	
DK650M;Akut diffus peritonitis		1	
DK650N;Akut purulent peritonitis		1	
DK650O;Akut subfrenisk peritonitis		1	
DK650P;Lokaliseret peritonitis		1	
DK658;Anden form for peritonitis		1	
DK658A;Galdeperitonitis		1	
DK658B;Omentitis		1	
DK658C;Fedtnekrose i mesenteriet		1	
DK658E;Kronisk proliferativ peritonitis		1	
DK658F;Urinperitonitis		1	
DK658G;Polyserositis peritonei		1	
DK658H;Saponificatio mesenterica		1	
DK658I;Spontan bakteriel peritonitis UNS		1	
DK659;Peritonitis UNS		1	
DK66;Andre sygdomme i bughinden		1	
DK660;Sammenvoksninger i bughinden		1	
DK660A;Adhaesiones diaphragmaticae		1	
DK660B;Adhaesiones intestini		1	
DK660C;Adhaesiones mesenterii		1	
DK660D;Adhaesiones omenti		1	
DK660E;Adhaesiones pelvis viri		1	
DK660F;Adhaesiones ventriculi		1	
DK661;Blødning i peritoneum		1	

DK668;Anden sygdom i bughinden		1	
DK668A;Cyste i peritoneum		1	
DK668C;Peritonitis forårsaget af stråling		1	
DK669;Sygdom i bughinde UNS		1	
DK67;Sygdomme i bughinde ved infektiøs sygdom klassificeret andetsteds		1	
DK670;Klamydiaperitonitis		1	
DK671;Gonoroisk peritonitis		1	
DK672;Sen syfilitisk peritonitis		1	
DK673;Tuberkuløs peritonitis		1	
DK678;Anden sygdom i bughinde ved infektiøs sygdom klassificeret andetsteds		1	
DK70;Alkoholisk leversygdom		1	
DK700;Alkoholisk fedtlever		1	
DK701;Alkoholisk leverbetændelse		1	
DK702;Alkoholisk leverfibrose		1	
DK703;Alkoholisk levercirrose		1	
DK703A;Cirrhosis hepatis Laennec		1	
DK704;Alkoholisk leverinsufficiens		1	
DK704A;Akut alkoholisk leverinsufficiens		1	
DK704B;Kronisk alkoholisk leverinsufficiens		1	
DK704C;Alkoholisk leverinsufficiens med koma		1	
DK704D;Alkoholisk leverinsufficiens UNS		1	
DK709;Alkoholisk leversygdom UNS		1	
DK71;Toksisk leversygdom		1	
DK710;Toksisk leversygdom med kolestase		1	
DK710A;Leversygdom med kolestase forårsaget af lægemiddel		1	
DK711;Toksisk leversygdom med nekrose		1	
DK711A;Leverkoma ved leversygdom forårsaget af lægemiddel		1	
DK711B;Leverkoma ved toksisk leversygdom		1	
DK711C;Leversygdom med nekrose forårsaget af lægemiddel		1	
DK712;Toksisk leversygdom med akut hepatitis		1	
DK712A;Leversygdom med akut hepatitis forårsaget af lægemiddel		1	
DK713;Toksisk leversygdom med kronisk persisterende hepatitis		1	

Supplement 2 Nielsen et al. Mortality after paediatric emergency calls for patients with and without pre-existing comorbidity (2023)

DK713A;Leversygdom med kronisk persisterende hepatitis forårsaget af lægemiddel		1	
DK714;Toksisk leversygdom med kronisk lobulær hepatitis		1	
DK714A;Leversygdom med kronisk lobulær hepatitis forårsaget af lægemiddel		1	
DK715;Toksisk leversygdom med kronisk aktiv hepatitis		1	
DK715A;Leversygdom med kronisk aktiv hepatitis forårsaget af lægemiddel		1	
DK715B;Leversygdom med lupoid hepatitis forårsaget af lægemiddel		1	
DK715D;Toksisk leversygdom med lupoid hepatitis		1	
DK716;Toksisk leversygdom med hepatitis IKA		1	
DK716A;Leversygdom forårsaget af lægemiddel IKA		1	
DK717;Toksisk leversygdom med fibrose eller cirrose		1	
DK717A;Levercirrose forårsaget af lægemiddel		1	
DK717B;Toksisk levercirrose		1	
DK718;Toksisk leversygdom med anden manifestation i leveren		1	
DK719;Toksisk leversygdom UNS		1	
DK72;Leverinsufficiens IKA		1	
DK720;Akut eller subakut leverinsufficiens		1	
DK720A;Akut hepatitis med leversvigt IKA		1	
DK720C;Malign hepatitis med leversvigt IKA		1	
DK720D;Levercellenekrose med leversvigt		1	
DK720E;Akut fulminant hepatitis med leversvigt IKA		1	
DK720F;Subakut fulminant hepatitis med leversvigt IKA		1	
DK721;Kronisk leverinsufficiens		1	
DK729;Leversvigt UNS		1	
DK729A;Gul leveratrofi		1	
DK73;Kronisk leverbetændelse IKA		1	
DK730;Kronisk persisterende hepatitis IKA		1	
DK731;Kronisk lobulær hepatitis IKA		1	
DK732;Kronisk aktiv hepatitis IKA		1	
DK732B;Autoimmun hepatitis type I med glatmuskulaturcelle-antistof		1	
DK732C;Autoimmun hepatitis type II uden glatmuskulaturcelle-antistof		1	
DK732D;Autoimmun kæmpecellehepatitis		1	
DK732E;Autoimmun hepatitis med samtidig primær biliær cirrose		1	

DK732F;Autoimmun hepatitis med samtidig primær skleroserende kolangitis		1	
DK732G;Autoimmun hepatitis UNS		1	
DK738;Anden form for kronisk hepatitis IKA		1	
DK739;Kronisk hepatitis UNS		1	
DK74;Fibrose i lever og skrumpelever		1	
DK740;Leverfibrose		1	
DK740A;Bantis syndrom		1	
DK740B;Fibrosis hepatolienalis		1	
DK741;Leversklerose		1	
DK742;Leverfibrose med sklerose		1	
DK743;Primær biliær levercirrose		1	
DK743A;Ikke-purulent kronisk destruktiv kolangitis		1	
DK744;Sekundær biliær levercirrose		1	
DK745;Biliær levercirrose UNS		1	
DK746;Anden eller ikke specificeret levercirrose		1	
DK746B;Levercirrose af blandet type		1	
DK746C;Makronodulær levercirrose		1	
DK746D;Mikronodulær levercirrose		1	
DK746E;Portal levercirrose		1	
DK746F;Postnekrotisk levercirrose		1	
DK746G;Levercirrose UNS		1	
DK746H;Kryptogen levercirrose		1	
DK75;Andre former for betændelse i leveren		1	
DK750;Leverabsces		1	
DK750A;Hæmatogen leverabsces		1	
DK750B;Lymfogen leverabsces		1	
DK750C;Leverabsces ved kolangitis		1	
DK750D;Leverabsces ved pyleflebitis		1	
DK751;Pyleflebitis		1	
DK752;Reaktiv hepatitis UNS		1	
DK753;Granulomatøs hepatitis IKA		1	
DK754;Autoimmun hepatitis		1	

DK754A;Lupoid hepatitis IKA		1	
DK758;Anden inflammatorisk leversygdom		1	
DK759;Inflammatorisk leversygdom UNS		1	
DK759A;Hepatitis UNS		1	
DK76;Andre leversygdomme		1	
DK760;Fedtdegeneration i leveren IKA		1	
DK760A;Ikke-alkoholisk fedtdegeneration i leveren		1	
DK760B;Ikke-alkoholisk fedtdegeneration i leveren med enzymforhøjelse		1	
DK760C;Ikke-alkoholisk fedtdegeneration i leveren med enzymforhøjelse og hepatitis		1	
DK761;Leverstase		1	
DK761A;Leverfibrose ved hjertesygdom		1	
DK761B;Kronisk leverstase ved hjertesygdom		1	
DK762;Central hæmoragisk levernekrose		1	
DK763;Leverinfarkt		1	
DK764;Peliosis hepatis		1	
DK764A;Angiomatosis hepatis		1	
DK765;Tillukning af levervener		1	
DK766;Portal hypertension		1	
DK766A;Portal hypertensiv gastropati uden blødning		1	
DK766B;Portal hypertensiv gastropati med blødning		1	
DK767;Hepatorenalt syndrom		1	
DK768;Anden leversygdom		1	
DK768A;Hepar constrictum		1	
DK768B;Hepatosplenomegalia		1	
DK768C;Melanosis hepatis		1	
DK768D;Ptosis hepatis		1	
DK768E;Levercyste		1	
DK769;Leversygdom UNS		1	
DK77;Leverlidelse ved sygdomme klassificeret andetsteds		1	
DK770;Leversygdom ved infektiøse eller parasitære sygdomme klassificeret andetsteds		1	
DK778;Leversygdom ved anden sygdom klassificeret andetsteds		1	
DK80;Galdesten		1	

Supplement 2 Nielsen et al. Mortality after paediatric emergency calls for patients with and without pre-existing comorbidity (2023)

DK800;Sten i galdeblæren med akut kolecystitis		1	
DK800A;Sten i ductus cysticus med akut kolecystitis		1	
DK800D;Galdestenskolik med akut kolecystitis		1	
DK801;Sten i galdeblæren med kronisk kolecystitis		1	
DK801A;Sten i ductus cysticus med kronisk kolecystitis		1	
DK801C;Sten i galdeblæren med kolecystitis UNS		1	
DK802;Sten i galdeblæren uden kolecystitis		1	
DK802A;Sten i ductus cysticus uden kolecystitis		1	
DK802D;Kolelitiasis UNS		1	
DK802E;Galdestenskolik i galdeblæren uden kolecystitis		1	
DK802F;Galdestenskolik i galdeblæren UNS		1	
DK803;Sten i galdegang med kolangitis		1	
DK803A;Sten i ductus choledochus med kolangitis		1	
DK803B;Sten i ductus hepaticus med kolangitis		1	
DK803C;Galdestenskolik med kolangitis		1	
DK804;Sten i galdegang med kolecystitis		1	
DK804A;Sten i ductus choledochus med kolangitis og kolecystitis		1	
DK804B;Sten i ductus choledochus med kolecystitis		1	
DK804C;Sten i ductus hepaticus med kolangitis og kolecystitis		1	
DK804D;Sten i ductus hepaticus med kolecystitis		1	
DK804E;Galdestenskolik med kolangitis og kolecystitis		1	
DK805;Galdesten uden kolangitis eller kolecystitis		1	
DK805B;Sten i ductus choledochus UNS		1	
DK805C;Sten i ductus choledochus uden kolangitis eller kolecystitis		1	
DK805E;Sten i ductus hepaticus uden kolangitis eller kolecystitis		1	
DK805F;Sten i ductus hepaticus UNS		1	
DK805J;Galdestenskolik uden kolangitis eller kolecystitis		1	
DK805K;Galdestenskolik UNS		1	
DK808;Anden form for galdesten		1	
DK81;Galdeblærebetændelse		1	
DK810;Akut kolecystitis		1	
DK810A;Galdeblæreabsces		1	

DK810B;Gangrænøs kolecystitis		1	
DK810C;Purulent kolecystitis		1	
DK810D;Galdeblæregangræn		1	
DK811;Kronisk kolecystitis		1	
DK818;Anden form for kolecystitis		1	
DK819;Kolecystitis UNS		1	
DK82;Andre sygdomme i galdeblæren		1	
DK820;Aflukning af ductus cysticus		1	
DK820A;Striktur af ductus cysticus		1	
DK821;Hydrops vesicae felleae		1	
DK822;Perforation af galdeblæren		1	
DK823;Fistel fra galdeblæren		1	
DK823A;Fistula cholecystocolica		1	
DK823B;Fistula cholecystoduodenalis		1	
DK824;Kolesterolose i galdeblæren		1	
DK828;Anden sygdom i galdeblæren		1	
DK828A;Adhaesiones ductus cystici		1	
DK828B;Adhaesiones vesicae felleae		1	
DK828C;Atrophia ductus cystici		1	
DK828D;Atrophia vesicae felleae		1	
DK828E;Cystis ductus cystici		1	
DK828F;Cystis vesicae felleae		1	
DK828G;Dyskinesia ductus cystici		1	
DK828H;Dyskinesia vesicae felleae		1	
DK828I;Hypertrophia ductus cystici		1	
DK828J;Hypertrophia vesicae felleae		1	
DK828K;Torsio vesicae felleae		1	
DK828L;Ulcus ductus cystici		1	
DK828M;Ulcus vesicae felleae		1	
DK829;Sygdom i galdeblæren UNS		1	
DK83;Andre sygdomme i galdevejene		1	
DK830;Kolangitis		1	

DK830A;Ascenderende kolangitis		1		
DK830B;Primær kolangitis		1		
DK830C;Purulent kolangitis		1		
DK830D;Recidiverende kolangitis		1		
DK830E;Sekundær kolangitis		1		
DK830F;Primær skleroserende kolangitis		1		
DK830G;Sekundær skleroserende kolangitis		1		
DK831;Galdegangsobstruktion		1		
DK831B;Striktur af galdegang		1		
DK832;Perforation af galdegang		1		
DK833;Fistel fra galdegang		1		
DK833A;Fistula choledochoduodenalis		1		
DK833B;Fistula cholocolica		1		
DK833C;Fistula gastrobiliaris		1		
DK834;Spasme i sphincter Oddi		1		
DK835;Galdegangscyste		1		
DK838;Anden sygdom i galdevejene		1		
DK838A;Galdegangsadhærencer		1		
DK838B;Galdegangsatrofi		1		
DK838C;Erhvervet deformation af galdegange		1		
DK838D;Galdegangsdyskinesi		1		
DK838E;Galdegangshypertofi		1		
DK838F;Blødning i galdegang		1		
DK839;Galdevejssygdom UNS		1		
DK85;Akut betændelse i bugspytkirtel		1		
DK850;Idiopatisk akut pankreatitis		1		
DK851;Akut pankreatitis forårsaget af galdevejslidelse		1		
DK851A;Galdestenspankreatitis		1		
DK852;Akut alkoholisk pankreatitis		1		
DK853;Akut pankreatitis forårsaget af lægemiddel		1		
DK858;Anden form for akut pankreatitis		1		
DK858A;Pancreasabsces		1		

DK858B;Akut hæmoragisk pankreatitis		1	
DK858C;Akut (adipøs) pancreasnekrose		1	
DK858D;Subakut pankreatitis		1	
DK858E;Infektiøs pancreasnekrose		1	
DK858F;Recidiverende akut pankreatitis		1	
DK859;Akut pankreatitis UNS		1	
DK86;Andre sygdomme i bugspytkirtlen		1	
DK860;Kronisk alkoholisk pankreatitis		1	
DK861;Anden form for kronisk pankreatitis		1	
DK861A;Kronisk infektiøs pankreatitis		1	
DK861B;Kronisk pankreatitis UNS		1	
DK861D;Kronisk recidiverende pankreatitis		1	
DK861E;Arvelig pankreatitis		1	
DK861F;Autoimmun pankreatitis		1	
DK862;Pancreascyste		1	
DK863;Pseudocyste i pancreas		1	
DK868;Anden sygdom i pancreas		1	
DK868A;Atrofi af pancreas		1	
DK868B;Pancreassten		1	
DK868C;Degeneration af pancreas		1	
DK868D;Pancreasfibrose		1	
DK868E;Pankreatikoduodenal fistel		1	
DK868F;Pancreasfistel		1	
DK868G;Pancreasblødning UNS		1	
DK868H;Infantilismus pancreaticus		1	
DK868I;Pancreasinsufficiens		1	
DK868J;Fedtnekrose af pancreas		1	
DK868K;Aseptisk pancreasnekrose		1	
DK868L;Pancreasnekrose UNS		1	
DK868M;Pancreas divisum		1	
DK869;Pancreassygdom UNS		1	
DK87;Sygdomme i galdeblæren, galdevejene og bugspytkirtlen ved sygdomme klassificeret andetsteds		1	

Supplement 2 Nielsen et al. Mortality after paediatric emergency calls for patients with and without pre-existing comorbidity (2023)

DK870;Sygdom i galdeblæren eller galdevejene ved sygdom klassificeret andetsteds		1	
DK871;Sygdom i pancreas ved sygdom klassificeret andetsteds		1	
DK90;Nedsat optagelse af næringsstoffer fra tarmen		1	
DK900;Cøliaki		1	
DK900B;Ikke-tropisk steatoré		1	
DK901;Tropisk sprue		1	
DK901A;Sprue UNS		1	
DK901B;Tropisk steatoré		1	
DK902;Blind loop-syndrom IKA		1	
DK903;Pankreatisk steatoré		1	
DK904;Malabsorption ved intolerans IKS		1	
DK904D;Malabsorption ved fedtintolerans		1	
DK904E;Malabsorption ved kulhydratintolerans		1	
DK904F;Malabsorption ved proteinintolerans		1	
DK908;Anden form for malabsorption		1	
DK908A;Whipples sygdom		1	
DK908B;Galdesyrediaré		1	
DK909;Malabsorption UNS		1	
DK91;Forstyrrelser i fordøjelsessystemets funktion efter indgreb IKA		1	
DK910;Opkastning efter gastrointestinal kirurgi		1	
DK911;Postgastrektomisyndrom		1	
DK911A;Postvagotomisyndrom		1	
DK912;Malabsorption efter gastrointestinal kirurgi IKA		1	
DK912B;Korttarmssyndrom		1	
DK912C;Postoperativt blind loop-syndrom		1	
DK913;Postoperativ tarmobstruktion		1	
DK913A;Tarmstenose efter kirurgi		1	
DK913B;Stenose ved gastrojejunostomi		1	
DK913C;Stenose ved jejunojejunostomi		1	
DK914;Dårligt fungerende kolostomi eller enterostomi		1	
DK914A;Dårligt fungerende enterostomi		1	
DK914B;Dårligt fungerende kolostomi		1	

Supplement 2 Nielsen et al. Mortality after paediatric emergency calls for patients with and without pre-existing comorbidity (2023)

DK914C;Postoperativ stominekrose		1	
DK914D;Postoperativ stomikomplikation IKA		1	
DK915;Postkolecystektomisyndrom		1	
DK918;Anden forstyrrelse i fordøjelsessystemet efter kirurgisk eller medicinsk behandling IKA		1	
DK918A;Postoperativt afferent loop syndrome		1	
DK918B;Nekrose af gastrisk pouch		1	
DK919;Forstyrrelse i fordøjelsessystemet UNS efter kirurgisk eller medicinsk behandling		1	
DK92;Andre sygdommme i fordøjelsessystemet		1	
DK920;Hæmatemese		1	
DK921;Melæna		1	
DK922;Gastrointestinal blødning UNS		1	
DK928;Anden sygdom i fordøjelsessystemet		1	
DK929;Sygdom i fordøjelsessystemet UNS		1	
DK93;Sygdomme i andre fordøjelsesorganer ved sygdom klassificeret andetsteds		1	
DK930;Tuberkulose i tarmen, mesenteriet eller mesenterielle lymfeknuder		1	
DK931;Megacolon ved Chagas' sygdom		1	
DK938;Sygdom i andet fordøjelsesorgan ved sygdom klassificeret andetsteds		1	
DL00;Eksfoliativ dermatitis forårsaget af stafylokokker		1	
DL009;Dermatitis exfoliativa staphylococcica		1	
DL009A;Ritters sygdom		1	
DL009B;Pemphigus neonatorum		1	
DL009C;Staphylococcal scalded skin syndrome		1	
DL01;Børnesår		1	
DL010;Impetigo UNS		1	
DL010A;Distal dactylitis forårsaget af hæmolytiske streptokokker, gruppe A		1	
DL010B;Impetigo bullosa		1	
DL010C;Impetigo palpebrae		1	
DL011;Dermatose med impetiginisering		1	
DL02;Bylder i huden		1	
DL020;Absces, furunkel eller karbunkel i huden i ansigtet		1	
DL020A;Furunkel i ansigtet		1	
DL020B;Karbunkel i ansigtet		1	

DL020C;Absces i ansigtet		1	
DL021;Absces, furunkel eller karbunkel i huden på halsen		1	
DL021A;Furunkel på halsen		1	
DL021B;Karbunkel på halsen		1	
DL021C;Absces på halsen		1	
DL022;Absces, furunkel eller karbunkel i huden på kroppen		1	
DL022A;Furunkel på brystvæggen		1	
DL022B;Furunkel i perineum		1	
DL022C;Furunkel på bugvæggen		1	
DL022D;Furunkel i lysken		1	
DL022E;Furunkel i navlen		1	
DL022F;Furunkel på ryggen		1	
DL022G;Karbunkel på ryggen		1	
DL022H;Karbunkel i lysken		1	
DL022I;Karbunkel på bugvæggen		1	
DL022J;Karbunkel på brystvæggen		1	
DL022K;Karbunkel i perineum		1	
DL022L;Karbunkel på kroppen UNS		1	
DL022M;Karbunkel i navlen		1	
DL022N;Absces på ryggen		1	
DL022O;Absces i navlen		1	
DL022P;Absces på kroppen UNS		1	
DL022Q;Absces i perineum		1	
DL022R;Absces på bugvæggen		1	
DL022S;Absces på brystvæggen		1	
DL022T;Absces i lysken		1	
DL023;Absces, furunkel eller karbunkel i huden i sæderegionen		1	
DL023A;Furunkel i sæderegionen		1	
DL023B;Karbunkel i sæderegionen		1	
DL023C;Absces i sæderegionen		1	
DL024;Absces, furunkel eller karbunkel i huden på ekstremitet		1	
DL024A;Absces på storetå		1	

DL024B;Absces på lår		1	
DL024C;Absces på arm		1	
DL024D;Absces i armhule		1	
DL024E;Absces på hånd		1	
DL024F;Absces på skinneben		1	
DL024G;Absces på finger UNS		1	
DL024H;Absces på overekstremitet UNS		1	
DL024I;Absces på underekstremitet UNS		1	
DL024J;Absces på tå UNS		1	
DL024K;Absces på underarm		1	
DL024L;Absces på tommelfinger		1	
DL024M;Absces på fod		1	
DL028;Absces, furunkel eller karbunkel i huden med anden lokalisation		1	
DL028A;Furunkel på hovedet		1	
DL028B;Karbunkel på hovedet		1	
DL028C;Absces på hovedet		1	
DL029;Absces, furunkel eller karbunkel i huden UNS		1	
DL029A;Furunkel UNS		1	
DL029B;Karbunkel UNS		1	
DL029C;Hudabsces UNS		1	
DL03;Flegmone		1	
DL030;Flegmone i finger eller tå		1	
DL030A;Panaritium tendinosum		1	
DL030B;Panaritium subunguale		1	
DL030C;Paronychion		1	
DL030D;Panaritium periunguale		1	
DL030E;Onychia		1	
DL030F;Panaritium articulare		1	
DL030G;Panaritium digiti		1	
DL030H;Flegmone i tå UNS		1	
DL030I;Flegmone i tommelfinger		1	
DL030J;Flegmone i storetå		1	

DL030K;Flegmone i finger UNS		1	
DL031;Flegmone med anden lokalisation på ekstremitet		1	
DL031A;Flegmone i lår		1	
DL031B;Flegmone i hånd UNS		1	
DL031C;Flegmone i overekstremitet UNS		1	
DL031D;Flegmone i fod UNS		1	
DL031E;Flegmone i skinneben		1	
DL031F;Flegmone i armhule		1	
DL031G;Flegmone i underarm		1	
DL031H;Flegmone i arm UNS		1	
DL031I;Flegmone i underekstremitet UNS		1	
DL032;Flegmone i ansigtet		1	
DL033;Flegmone på kroppen		1	
DL033A;Flegmone på ryggen		1	
DL033B;Flegmone i lysken		1	
DL033C;Flegmone i navlen		1	
DL033D;Flegmone i perineum		1	
DL033E;Flegmone i brystvæggen		1	
DL033F;Flegmone i bugvæggen		1	
DL038;Flegmone med anden lokalisation		1	
DL038A;Flegmone på hovedet		1	
DL038B;Flegmone på halsen		1	
DL039;Flegmone UNS		1	
DL039A;Cellulitis non spec. (findes ikke i SKS)		1	
DL04;Akut lymfadenitis		1	
DL040;Akut lymfadenitis i ansigtet, på hovedet og halsen		1	
DL040A;Akut lymfadenitis på hovedet		1	
DL040B;Akut lymfadenitis på halsen		1	
DL040C;Akut lymfadenitis i ansigtet		1	
DL041;Akut lymfadenitis på kroppen		1	
DL042;Akut lymfadenitis på overekstremitet		1	
DL042A;Akut lymfadenitis i armhule		1	

Supplement 2 Nielsen et al. Mortality after paediatric emergency calls for patients with and without pre-existing comorbidity (2023)

DL043;Akut lymfadenitis på underekstremitet		1	
DL048;Akut lymfadenitis med anden lokalisation		1	
DL049;Akut lymfadenitis UNS		1	
DL05;Hårrede over halebenet		1	
DL050;Pilonidalcyste med absces		1	
DL059;Pilonidalcyste uden absces		1	
DL08;Andre lokale infektioner i hud og underhud		1	
DL080;Pyoderma		1	
DL080A;Pyodermia staphylococcica		1	
DL080B;Ecthyma		1	
DL080C;Ecthyma gangraenosum		1	
DL081;Erytrasma		1	
DL088;Anden lokal infektion i hud eller underhud		1	
DL088C;Pyoderma vegetans (Hallopeau)		1	
DL088D;Perianal streptokokinfektion		1	
DL089;Lokal infektion i hud eller underhud UNS		1	
DL10;Blæreudslæt		1	
DL100;Pemphigus vulgaris		1	
DL101;Pemphigus vegetans		1	
DL102;Pemphigus foliaceus		1	
DL103;Brasiliansk pemfigus		1	
DL104;Pemphigus erythematosus		1	
DL105;Pemfigus forårsaget af lægemiddel		1	
DL108;Anden form for pemfigus		1	
DL108A;IgA-pemfigus		1	
DL109;Pemfigus UNS		1	
DL11;Andre former for akantolyse		1	
DL110;Erhvervet follikulær keratose		1	
DL111;Transitorisk akantolytisk dermatose		1	
DL118;Anden form for akantolyse		1	
DL119;Akantolyse UNS		1	
DL12;Pemfigoid		1	

DL120;Bulløs pemfigoid		1	
DL121;Pemphigoides cicatricialis		1	
DL121A;Pemphigoides mucosa benigna		1	
DL122;Dermatitis bullosa chronica infantilis		1	
DL122A;Dermatitis herpetiformis infantilis		1	
DL123;Epidermolysis bullosa acquisita		1	
DL128;Anden form for pemfigoid		1	
DL129;Pemfigoid UNS		1	
DL13;Andre former for blæreudslæt		1	
DL130;Dermatitis herpetiformis		1	
DL131;Subcorneal pustuløs dermatose		1	
DL138;Anden bulløs dermatitis		1	
DL138A;Lineær IgA-dermatose		1	
DL138B;Intraepidermal neutrofil IgA-dermatose		1	
DL138C;Infantil akropustulose		1	
DL139;Blæreudslæt UNS		1	
DL14;Blæreudslæt ved sygdom klassificeret andetsteds		1	
DL149;Bulløs dermatitis ved sygdom klassificeret andetsteds		1	
DL20;Atopisk eksem		1	
DL200;Prurigo Besnier		1	
DL208;Anden form for atopisk dermatitis		1	
DL208A;Dermatitis atopica (findes ikke i SKS)		1	
DL208B;Dermatitis plantaris sicca		1	
DL208C;Keratosis pilaris		1	
DL208D;Atopisk håndeksem		1	
DL209;Atopisk dermatitis UNS		1	
DL21;Seborroisk dermatitis		1	
DL210;Dermatitis seborrhoica capitis		1	
DL210B;Pityriasis capitis		1	
DL210C;Pityriasis simplex		1	
DL210D;Seborroisk dermatitis i ansigtet		1	
DL210E;Seborroisk dermatitis i hovedbunden		1	

DL211;Dermatitis seborrhoica infantilis		1	
DL211A;Leiners sygdom		1	
DL218;Anden form for seborroisk dermatitis		1	
DL218A;Eczema seborrhoicum		1	
DL218B;Pityriasis simplex eczematisata		1	
DL218D;Pityriasis steatodes figurata		1	
DL218E;Seborrhoea sicca		1	
DL218F;Generaliseret seborroisk dermatitis		1	
DL219;Seborroisk dermatitis UNS		1	
DL22;Eksem i bleregionen		1	
DL229;Bledermatitis		1	
DL229B;Jadassohns psoriasiforme bledermatitis		1	
DL23;Allergisk kontaktdermatitis		1	
DL230;Allergisk kontaktdermatitis forårsaget af metal		1	
DL230A;Erhvervsbetinget allergisk kontaktdermatitis forårsaget af metal UNS		1	
DL230B;Allergisk kontaktdermatitis forårsaget af krom		1	
DL230C;Erhvervsbetinget allergisk kontaktdermatitis forårsaget af krom		1	
DL230D;Allergisk kontaktdermatitis forårsaget af nikkel		1	
DL230E;Erhvervsbetinget allergisk kontaktdermatitis forårsaget af nikkel		1	
DL231;Allergisk kontaktdermatitis forårsaget af klæbestof		1	
DL231A;Erhvervsbetinget allergisk kontaktdermatitis forårsaget af klæbestof		1	
DL232;Allergisk kontaktdermatitis forårsaget af kosmetik		1	
DL232A;Erhvervsbetinget allergisk kontaktdermatitis forårsaget af kosmetik		1	
DL232B;Allergisk kontaktdermatitis forårsaget af parfume		1	
DL232C;Erhvervsbetinget allergisk kontaktdermatitis forårsaget af parfume		1	
DL232D;Allergisk kontaktdermatitis forårsaget af konserveringsmiddel i kosmetik		1	
DL232E;Erhvervsbetinget allergisk kontaktdermatitis forårsaget af konserveringsmiddel i kosmetik		1	
DL233;Allergisk dermatitis forårsaget af hudkontakt med lægemiddel		1	
DL233A;Erhvervsbetinget allergisk dermatitis forårsaget af hudkontakt med lægemiddel		1	
DL234;Allergisk kontaktdermatitis forårsaget af farvestof		1	
DL234A;Erhvervsbetinget allergisk kontaktdermatitis forårsaget af farvestof		1	
DL235;Allergisk kontaktdermatitis forårsaget af andet kemikalie		1	

DL235A;Erhvervsbetinget allergisk kontaktdermatitis forårsaget af andet kemikalie		1	
DL235B;Allergisk kontaktdermatitis forårsaget af plastik		1	
DL235C;Erhvervsbetinget allergisk kontaktdermatitis forårsaget af plastik		1	
DL235D;Allergisk kontaktdermatitis forårsaget af sprøjtemiddel		1	
DL235E;Erhvervsbetinget allergisk kontaktdermatitis forårsaget af sprøjtemiddel		1	
DL235F;Allergisk kontaktdermatitis forårsaget af latex		1	
DL235G;Erhvervsbetinget allergisk kontaktdermatitis forårsaget af latex		1	
DL235H;Allergisk kontaktdermatitis forårsaget af anden form for gummi		1	
DL235I;Erhvervsbetinget allergisk kontaktdermatitis forårsaget af anden form for gummi		1	
DL236;Allergisk dermatitis forårsaget af hudkontakt med fødeemne		1	
DL236A;Erhvervsbetinget allergisk dermatitis forårsaget af hudkontakt med fødeemne		1	
DL237;Allergisk kontaktdermatitis forårsaget af hudkontakt med plante		1	
DL237A;Erhvervsbetinget allergisk dermatitis forårsaget af hudkontakt med plante		1	
DL238;Allergisk kontaktdermatitis forårsaget af anden substans		1	
DL238A;Erhvervsbetinget allergisk kontaktdermatitis forårsaget af anden substans		1	
DL238B;Allergisk kontaktdermatitis forårsaget af skimmelsvampe		1	
DL238C;Allergisk kontaktdermatitis forårsaget af støvmider		1	
DL239;Allergisk kontaktdermatitis UNS		1	
DL239A;Erhvervsbetinget allergisk kontaktdermatitis UNS		1	
DL24;Toksisk kontaktdermatitis		1	
DL240;Toksisk kontaktdermatitis forårsaget af vaskemiddel		1	
DL240A;Erhvervsbetinget toksisk kontaktdermatitis forårsaget af vaskemiddel		1	
DL241;Toksisk kontaktdermatitis forårsaget af olie eller smøremiddel		1	
DL241A;Erhvervsbetinget toksisk kontaktdermatitis forårsaget af olie eller smøremiddel		1	
DL242;Toksisk kontaktdermatitis forårsaget af opløsningsmiddel		1	
DL242A;Erhvervsbetinget toksisk kontaktdermatitis forårsaget af opløsningsmiddel		1	
DL243;Toksisk kontaktdermatitis forårsaget af kosmetik		1	
DL243A;Erhvervsbetinget toksisk kontaktdermatitis forårsaget af kosmetik		1	
DL244;Toksisk dermatitis forårsaget af hudkontakt med lægemiddel		1	
DL244A;Erhvervsbetinget toksisk dermatitis forårsaget af hudkontakt med lægemiddel		1	
DL245;Toksisk kontaktdermatitis forårsaget af andet kemikalie		1	
DL245A;Erhvervsbetinget toksisk kontaktdermatitis forårsaget af andet kemikalie		1	

DL245B;Toksisk kontaktdermatitis forårsaget af cement		1	
DL245C;Erhvervsbetinget toksisk kontaktdermatitis forårsaget af cement		1	
DL245D;Toksisk kontaktdermatitis forårsaget af insekticid		1	
DL245E;Erhvervsbetinget toksisk kontaktdermatitis forårsaget af insekticid		1	
DL246;Toksisk dermatitis forårsaget af hudkontakt med fødeemne		1	
DL246A;Erhvervsbetinget toksisk dermatitis forårsaget af hudkontakt med fødeemne		1	
DL247;Toksisk kontaktdermatitis forårsaget af hudkontakt med plante		1	
DL247A;Erhvervsbetinget toksisk kontaktdermatitis forårsaget af hudkontakt med plante		1	
DL248;Toksisk kontaktdermatitis forårsaget af anden substans		1	
DL248A;Erhvervsbetinget toksisk kontaktdermatitis forårsaget af enden substans		1	
DL249;Toksisk kontaktdermatitis UNS		1	
DL249A;Erhvervsbetinget toksisk kontaktdermatitis UNS		1	
DL25;Ikke nærmere specificeret kontaktdermatitis		1	
DL250;Kontaktdermatitis UNS forårsaget af kosmetik		1	
DL251;Dermatitis UNS forårsaget af hudkontakt med lægemiddel		1	
DL252;Kontaktdermatitis UNS forårsaget af farvestof		1	
DL253;Kontaktdermatitis UNS forårsaget af andre kemikalier		1	
DL253A;Kontaktdermatitis UNS forårsaget af cement		1	
DL253B;Kontaktdermatitis UNS forårsaget af insekticid		1	
DL254;Dermatitis UNS forårsaget af hudkontakt med fødeemne		1	
DL255;Kontaktdermatitis UNS forårsaget af plante		1	
DL258;Kontaktdermatitis UNS forårsaget af anden substans		1	
DL259;Kontaktdermatitis UNS uden specificeret årsag		1	
DL259A;Erhvervsbetinget kontaktdermatitis UNS uden specificeret årsag		1	
DL26;Eksfoliativ dermatitis		1	
DL269;Dermatitis exfoliativa		1	
DL269B;Keratolysis plantare sulcatum		1	
DL27;Dermatitis forårsaget af indtaget substans		1	
DL270;Generaliseret dermatitis forårsaget af indtaget lægemiddel		1	
DL270A;Exanthema medicamentale		1	
DL270B;Akut generaliseret eksantematøs pustulose (AGEP) forårsaget af indtaget lægemiddel		1	
DL271;Lokaliseret dermatitis forårsaget af indtaget lægemiddel		1	

Supplement 2 Nielsen et al. Mortality after paediatric emergency calls for patients with and without pre-existing comorbidity (2023)

DL271A;Autoimmun progesterondermatitis		1	
DL271B;Diuretikabetinget bulla		1	
DL271C;Erythema fixum forårsaget af lægemiddel		1	
DL272;Dermatitis forårsaget af indtaget fødeemne		1	
DL278;Dermatitis forårsaget af anden indtaget substans		1	
DL278A;Alkoholdermatitis		1	
DL278B;Iododerma		1	
DL279;Dermatitis forårsaget af indtaget substans UNS		1	
DL28;Lichenisering og prurigo		1	
DL280;Lichen simplex chronicus		1	
DL280A;Lichen UNS		1	
DL280B;Lichenisering UNS		1	
DL280C;Neurodermatitis circumscripta		1	
DL281;Prurigo nodularis		1	
DL282;Anden form for prurigo		1	
DL282A;Urticaria papulosa		1	
DL282B;Prurigo UNS		1	
DL282C;Prurigo simplex acuta		1	
DL282D;Prurigo mitis		1	
DL29;Kløe		1	
DL290;Analkløe		1	
DL291;Kløe på scrotum		1	
DL292;Kløe på vulva		1	
DL293;Anogenital kløe UNS		1	
DL298;Anden form for kløe		1	
DL298A;Aquagen kløe		1	
DL298B;Kløe ved intern sygdom		1	
DL298C;Uræmisk kløe		1	
DL298D;Senil kløe		1	
DL299;Kløe UNS		1	
DL30;Andre former for dermatitis		1	
DL300;Dermatitis nummularis		1	

Supplement 2 Nielsen et al. Mortality after paediatric emergency calls for patients with and without pre-existing comorbidity (2023)

DL301;Dyshidrose		1	
DL301A;Dermatitis dyshidrotica		1	
DL301B;Fokal palmar peeling		1	
DL301C;Vesikulose		1	
DL302;Kutan autosensibilisering		1	
DL302A;Dermatofytid		1	
DL303;Infektiøs dermatitis		1	
DL304;Intertrigo		1	
DL305;Pityriasis alba		1	
DL305A;Pityriasis streptogenes		1	
DL308;Anden form for dermatitis		1	
DL308A;Dermatitis acuta UNS		1	
DL308B;Dermatitis chronica UNS		1	
DL308C;Eksem (findes ikke i SKS)		1	
DL308D;Dermatitis perianalis UNS		1	
DL308F;Mikrobielt eksem		1	
DL308G;Eczema keratoticum		1	
DL308H;Håndeksem UNS		1	
DL308I;Friktionsdermatitis		1	
DL309;Dermatitis UNS		1	
DL40;Psoriasis		1	
DL400;Psoriasis vulgaris		1	
DL400B;Psoriasis nummularis		1	
DL400C;Psoriasis en plaque		1	
DL400D;Psoriasis discoides		1	
DL401;Psoriasis pustulosa generalisata		1	
DL401B;Impetigo herpetiformis		1	
DL402;Acrodermatitis continua		1	
DL402B;Acrodermatitis continua maligna		1	
DL403;Pustulosis palmaris et plantaris		1	
DL403A;Pustulosis palmaris		1	
DL403B;Pustulosis plantaris		1	

Supplement 2 Nielsen et al. Mortality after paediatric emergency calls for patients with and without pre-existing comorbidity (2023)

DL404;Guttat psoriasis		1	
DL405;Psoriasis artropati		1	
DL408;Anden form for psoriasis		1	
DL408A;Psoriasis inversa		1	
DL408B;Erythrodermia psoriatica		1	
DL408C;Psoriasis på hænder og fødder		1	
DL408D;Neglepsoriasis		1	
DL408E;Pityriasis amiantacea		1	
DL409;Psoriasis UNS		1	
DL41;Parapsoriasis		1	
DL410;Pityriasis lichenoides et varioliformis acuta		1	
DL411;Pityriasis lichenoides chronica		1	
DL413;Small plaque-parapsoriasis		1	
DL414;Large plaque-parapsoriasis		1	
DL415;Parapsoriasis retiformis		1	
DL418;Anden form for parapsoriasis		1	
DL418A;Parapsoriasis variegata		1	
DL419;Parapsoriasis UNS		1	
DL42;Pityriasis rosea		1	
DL429;Pityriasis rosea UNS		1	
DL429A;Pityriasis circinata		1	
DL429B;Pityriasis maculata		1	
DL43;Lichen ruber planus		1	
DL430;Hypertrofisk lichen ruber planus		1	
DL431;Bulløs lichen ruber planus		1	
DL432;Lichenoid lægemiddelreaktion		1	
DL433;Subakut lichen ruber planus		1	
DL438;Anden form for lichen ruber planus		1	
DL438A;Lichen ruber actinicus		1	
DL438B;Lichen ruber erosiva		1	
DL438C;Lichen planus pemphigoides		1	
DL438D;Lichenoid dermatitis		1	

DL438E;Keratosis lichenoides chronica		1		
DL439;Lichen ruber planus UNS		1		
DL44;Andre papuloskvamøse sygdomme		1		
DL440;Pityriasis rubra pilaris		1		
DL441;Lichen nitidus		1		
DL442;Lichen striatus		1		
DL443;Lichen ruber moniliformis		1		
DL444;Acrodermatitis papulosa infantum		1		
DL444A;Asymmetrical periflexural exanthem of childhood (APEC)		1		
DL448;Anden papuloskvamøs sygdom		1		
DL448A;Parakeratosis pustulosa		1		
DL448B;Erythromelanosis follicularis faciei et colli		1		
DL448C;Papuloerythroderma		1		
DL448D;Inflammatorisk lineært verrukøst epidermalt nævus (ILVEN)		1		
DL449;Papuloskvamøs sygdom UNS		1		
DL45;Papuloskvamøse tilstande ved sygdomme klassificeret andetsteds		1		
DL459;Papuloskvamøs tilstand ved sygdom klassificeret andetsteds		1		
DL50;Nældefeber		1		
DL500;Allergisk urticaria		1		
DL501;Idiopatisk urticaria		1		
DL502;Urticaria forårsaget af kulde eller varme		1		
DL502A;Varmeurticaria		1		
DL502B;Kuldeurticaria		1		
DL503;Dermografisme		1		
DL504;Urticaria vibratoria		1		
DL505;Urticaria cholinergica		1		
DL506;Kontakturticaria		1		
DL506A;Erhvervsbetinget kontakturticaria		1		
DL508;Anden form for nældefeber		1		
DL508A;Urticaria chronica		1		
DL508B;Urticaria recidivans		1		
DL508C;Urticaria aquagenica		1		

Supplement 2 Nielsen et al. Mortality after paediatric emergency calls for patients with and without pre-existing comorbidity (2023)

DL508D;Urticaria acuta		1	
DL508E;Urticaria forårsaget af tryk		1	
DL509;Nældefeber UNS		1	
DL51;Erythema multiforme		1	
DL510;Erythema multiforme non bullosum		1	
DL511;Erythema multiforme bullosum		1	
DL511A;Stevens-Johnsons syndrom		1	
DL512;Necrolysis epidermalis toxica		1	
DL518;Anden form for erythema multiforme		1	
DL518A;Erythema multiforme majus		1	
DL519;Erythema multiforme UNS		1	
DL52;Knuderosen		1	
DL529;Erythema nodosum		1	
DL53;Andre erytematøse tilstande		1	
DL530;Erythema toxicum		1	
DL531;Erythema annulare centrifugum		1	
DL532;Erythema marginatum		1	
DL533;Anden form for kronisk erythema figuratum		1	
DL538;Anden erytematøs tilstand		1	
DL538A;Erythema gyratum repens		1	
DL538B;Erythema palmare		1	
DL538C;Erythrodermia exfoliativa		1	
DL539;Erytem eller erytrodermi UNS		1	
DL539A;Erytrodermi UNS		1	
DL539B;Erytem UNS		1	
DL54;Erytem ved sygdomme klassificeret andetsteds		1	
DL540;Erythema marginatum ved gigtfeber		1	
DL548;Erytem ved sygdom klassificeret andetsteds		1	
DL55;Solskoldning		1	
DL550;Solskoldning med 1. grads forbrænding		1	
DL551;Solskoldning med 2. grads forbrænding		1	
DL552;Solskoldning med 3. grads forbrænding		1	

DL558;Anden form for solskoldning		1	
DL559;Solskoldning UNS		1	
DL56;Andre akutte hudforandringer forårsaget af ultraviolet lys		1	
DL560;Fototoksisk lægemiddelreaktion		1	
DL561;Fotoallergisk lægemiddelreaktion		1	
DL562;Fototoksisk eller fotoallergisk kontaktdermatitis		1	
DL562B;Fototoksisk kontaktdermatitis		1	
DL562C;Fotoallergisk kontaktdermatitis		1	
DL562D;Dermatitis ex herbis		1	
DL563;Urticaria solaris		1	
DL564;Polymorft lysudslæt		1	
DL568;Anden akut hudforandring forårsaget af ultraviolet lys		1	
DL568A;Hydroa vacciniformia		1	
DL568B;Elastose med cyster og komedoner		1	
DL568C;Aktinisk keilitis		1	
DL569;Hudforandring UNS forårsaget af ultraviolet lys		1	
DL57;Hudforandringer ved langvarig eksponering for ikke-joniserende stråling		1	
DL570;Aktinisk keratose		1	
DL570A;Keratosis actinica solaris		1	
DL570B;Keratosis actinica senilis		1	
DL570C;Keratosis UNS		1	
DL571;Aktinisk retikuloid		1	
DL572;Cutis rhomboidalis nuchae		1	
DL573;Civattes poikiloderma		1	
DL574;Cutis laxa senilis		1	
DL574B;Solar elastose		1	
DL575;Aktinisk granulom		1	
DL578;Anden hudforandring ved langvarig ikke-joniserende stråling		1	
DL578A;Dermatitis solaris		1	
DL578B;Sømandshud		1	
DL578C;Landmandshud		1	
DL579;Hudforandring UNS ved langvarig ikke-joniserende stråling		1	

Supplement 2 Nielsen et al. Mortality after paediatric emergency calls for patients with and without pre-existing comorbidity (2023)

DL58;Stråledermatitis		1		
DL580;Akut stråledermatitis		1		
DL581;Kronisk stråledermatitis		1		
DL589;Stråledermatitis UNS		1		
DL59;Andre strålebetingede sygdomme i hud og underhud		1		
DL590;Erythema ab igne		1		
DL598;Anden strålebetinget sygdom i hud og underhud		1		
DL598A;Fibrose og ardannelse i hud og underhud forårsaget af stråling		1		
DL599;Strålebetinget sygdom i hud og underhud UNS		1		
DL60;Sygdomme i negle		1		
DL600;Nedgroet negl		1		
DL601;Negleløsning		1		
DL602;Klonegl		1		
DL603;Negledystrofi		1		
DL603A;Dystrophia mediana canaliformis unguium		1		
DL603B;Onychoschisis		1		
DL604;Beaus neglefurer		1		
DL605;Yellow nail-syndrom		1		
DL608;Anden neglesygdom		1		
DL608A;Atrophia unguinis		1		
DL608B;Anonychia acquisita		1		
DL608C;Deformatio unguinis		1		
DL608D;Leukonychia		1		
DL608E;Pincer nails		1		
DL608F;Koilonychia		1		
DL608G;Hypertrophia unguium		1		
DL608H;Terry nails		1		
DL608I;Twenty nail-dystrofi		1		
DL609;Neglesygdom UNS		1		
DL62;Neglelidelser ved sygdomme klassificeret andetsteds		1		
DL620;Urglasnegle ved pachydermoperiostosis		1		
DL628;Neglelidelse ved sygdom klassificeret andetsteds		1		

Supplement 2 Nielsen et al. Mortality after paediatric emergency calls for patients with and without pre-existing comorbidity (2023)

DL628A;Urglasnegle		1	
DL63;Alopecia areata		1	
DL630;Alopecia capitis totalis		1	
DL631;Alopecia universalis		1	
DL632;Ophiasis		1	
DL638;Anden form for alopecia areata		1	
DL638A;Alopecia areata localisata		1	
DL639;Alopecia areata UNS		1	
DL64;Hårtab forårsaget af mandligt kønshormon		1	
DL640;Androgen alopeci forårsaget af lægemiddel		1	
DL648;Anden form for androgen alopeci		1	
DL649;Androgen alopeci UNS		1	
DL65;Andre former for hårtab uden ardannelse		1	
DL650;Effluvium capillorum telogenes		1	
DL651;Effluvium capillorum anagenes		1	
DL652;Mucinøs alopeci		1	
DL658;Anden form for alopeci uden ardannelse		1	
DL658B;Alopeci forårsaget af lægemiddel		1	
DL658C;Loose anagen hair syndrome		1	
DL659;Alopeci uden ardannelse UNS		1	
DL66;Hårtab med ardannelse		1	
DL660;Pseudopelade		1	
DL661;Lichen planopilaris		1	
DL662;Folliculitis decalvans		1	
DL663;Perifolliculitis capitis abscedens		1	
DL664;Folliculitis ulerythematosa reticulata		1	
DL668;Anden form for alopeci med ardannelse		1	
DL668A;Alopecia cicatricialis lupuides		1	
DL669;Alopeci med ardannelse UNS		1	
DL67;Abnorm hårfarve og hårform		1	
DL670;Trichorrhexis nodosa		1	
DL671;Variation i hårfarve		1	

DL671A;Canities pilorum		1	
DL671B;Præmatur gråhårethed		1	
DL671C;Poliosis circumscripta acquisita		1	
DL671D;Heterochromia pilorum		1	
DL678;Anden abnorm hårfarve eller hårform		1	
DL678A;Abnorm hårform IKA		1	
DL678B;Trichothiodystrofi		1	
DL678C;Bamboo-hair		1	
DL678D;Uncombable hair-syndrom		1	
DL679;Abnorm hårfarve eller hårform UNS		1	
DL68;Forøget hårvækst		1	
DL680;Hirsutisme		1	
DL681;Hypertrichosis lanuginosa acquisita		1	
DL682;Lokaliseret hypertrikose		1	
DL683;Polytrichia		1	
DL688;Anden form for hypertrikose		1	
DL689;Hypertrikose UNS		1	
DL70;Akne		1	
DL700;Acne vulgaris		1	
DL701;Acne conglobata		1	
DL702;Acne varioliformis		1	
DL702A;Acne necrotica miliaris		1	
DL703;Acne tropica		1	
DL704;Acne infantilis		1	
DL705;Acné excoriée des jeunes filles		1	
DL708;Anden form for akne		1	
DL708A;Komedon		1	
DL708B;Dermatitis papillaris capillitii		1	
DL708C;Acne fulminans		1	
DL708D;Acne mechanica		1	
DL708E;Acne agminata		1	
DL708F;Akne forårsaget af lægemiddel		1	

DL708G;Acne venenata		1		
DL708H;Klorakne		1		
DL708I;Acne cosmetica		1		
DL709;Akne UNS		1		
DL71;Rosacea		1		
DL710;Perioral dermatitis		1		
DL711;Rhinophyma		1		
DL718;Anden form for rosacea		1		
DL718A;Acne rosacea		1		
DL718B;Lupus miliaris disseminatus faciei		1		
DL718D;Rosacea-lignende dermatitis efter steroidbehandling		1		
DL718E;Rosacea microlupoides sive lupoides		1		
DL719;Rosacea UNS		1		
DL72;Follikulære cyster i hud og underhud		1		
DL720;Epidermal cyste		1		
DL721;Cystis trichodermalis		1		
DL721A;Cystis pilaris		1		
DL721B;Cystis sebacea		1		
DL722;Steatocystoma multiplex		1		
DL728;Anden follikulær cyste i hud og underhud		1		
DL729;Follikulær cyste i hud og underhud UNS		1		
DL73;Andre follikulære sygdomme i hud og underhud		1		
DL730;Aknekeloid		1		
DL731;Pili retenti		1		
DL732;Suppurerende hidrosadenitis		1		
DL738;Anden follikulær sygdom i hud og underhud		1		
DL738A;Folliculitis UNS		1		
DL738B;Folliculitis barbae		1		
DL738C;Psilosis non sprue		1		
DL738D;Pseudosycosis barbae		1		
DL738E;Sycosis barbae		1		
DL738F;Eosinofil pustuløs folliculitis (Ofuji)		1		

DL738G;Infundibulofollicullitis		1	
DL738H;Bakteriel folliculitis		1	
DL739;Follikulær sygdom i hud og underhud UNS		1	
DL74;Forstyrrelser i svedsekretionen		1	
DL740;Miliaria rubra		1	
DL741;Miliaria crystallina		1	
DL742;Miliaria profunda		1	
DL742A;Miliaria tropicalis		1	
DL743;Miliaria UNS		1	
DL743A;Exanthema caloris		1	
DL743B;Sudamina		1	
DL744;Anhidrosis		1	
DL744A;Hypohidrosis		1	
DL748;Anden forstyrrelse i svedsekretionen		1	
DL748A;Neutrofil ekkrin hidradenitis		1	
DL749;Forstyrrelse i svedsekretionen UNS		1	
DL75;Forstyrrelser i apokrine svedkirtler		1	
DL750;Bromhidrosis		1	
DL751;Chromhidrosis		1	
DL752;Miliaria i apokrine svedkirtler		1	
DL752A;Fox-Fordyces sygdom		1	
DL758;Anden forstyrrelse i apokrine svedkirtler		1	
DL759;Forstyrrelse i apokrine svedkirtler UNS		1	
DL80;Vitiligo		1	
DL809;Vitiligo		1	
DL81;Andre forstyrrelser i hudens pigmentering		1	
DL810;Postinflammatorisk hyperpigmentering		1	
DL811;Kloasma		1	
DL811A;Chloasma symptomaticum		1	
DL812;Fregner		1	
DL813;Café au lait-pletter		1	
DL814;Anden form for melaninhyperpigmentering		1	

Supplement 2 Nielsen et al. Mortality after paediatric emergency calls for patients with and without pre-existing comorbidity (2023)

DL814A;Hypermelanosis UNS		1	
DL814B;Melanosis cutis		1	
DL814C;Nævoid hypermelanose		1	
DL814D;Linear and whorled hypermelanosis		1	
DL814E;Lentigo		1	
DL814EA;Lentigo labialis		1	
DL814EB;Lentigo fremkaldt af PUVA		1	
DL814EC;Lentigo solaris		1	
DL814F;Lentiginosis		1	
DL814G;Melanoderma		1	
DL815;Leukoderma IKA		1	
DL815A;Suttons sygdom		1	
DL816;Anden form for nedsat melanindannelse		1	
DL816A;Depigmentatio cutis		1	
DL816B;Guttat hypomelanosis		1	
DL816C;Leukoderma UNS		1	
DL817;Pigmenteret purpura		1	
DL817A;Purpura annularis teleangiectoides		1	
DL817B;Schambergs sygdom		1	
DL817C;Dermatosis pigmentosa progressiva		1	
DL817E;Gougerot-Blums sygdom		1	
DL817F;Angioma serpiginosum		1	
DL817G;Lichen aureus		1	
DL818;Anden forstyrrelse i hudens pigmentering IKA		1	
DL818B;Jernpigmentering af huden		1	
DL818D;Dermatitis maculosa nigra		1	
DL818E;Tatovatio		1	
DL818G;Argyria		1	
DL818H;Erythema dyschromicum perstans		1	
DL818I;Chrysiasis		1	
DL818M;Xanthochromia		1	
DL819;Forstyrrelse i hudens pigmentering UNS		1	

Supplement 2 Nielsen et al. Mortality after paediatric emergency calls for patients with and without pre-existing comorbidity (2023)

DL82;Seborroisk keratose		1	
DL829;Keratosis seborrhoica		1	
DL829A;Dermatosis papulosa nigra		1	
DL829B;Leser-Trélats sygdom		1	
DL83;Acanthosis nigricans		1	
DL839;Acanthosis nigricans UNS		1	
DL839A;Acanthosis benigna		1	
DL839B;Acanthosis endocrinologica		1	
DL839C;Acanthosis paraneoplastica		1	
DL839D;Reticular and confluent papillomatosis		1	
DL84;Hård hud og ligtorne		1	
DL849;Callositas et clavus		1	
DL849A;Hård hud		1	
DL849B;Ligtorn		1	
DL85;Andre former for fortykkelse af hud		1	
DL850;Erhvervet ichthyosis		1	
DL851;Keratoderma palmare et plantare acquisitum		1	
DL852;Keratosis punctata		1	
DL853;Xerosis cutis		1	
DL853A;Dermatitis asteatoticum		1	
DL858;Anden form for epidermal fortykkelse		1	
DL858A;Kutane horn		1	
DL858B;Acanthoma fissuratum		1	
DL858C;Acrokeratosis paraneoplastica		1	
DL858D;Keratosis arsenicalis		1	
DL858E;Erythrokeratodermia progressiva symmetrica		1	
DL858F;Erythrokeratodermia variabilis		1	
DL858G;Hyperkeratosis lenticularis perstans		1	
DL858H;Granuloma fissuratum		1	
DL858I;Greithers syndrom		1	
DL858J;Hyperkeratosis aquisita palmaris et plantaris		1	
DL858K;Keratoderma palmaris et plantaris UNS		1	

DL858L;Keratoderma palmaris et plantaris Vörner		1	
DL858M;Keratoderma palmaris et plantaris Unna-Thost		1	
DL858N;Keratoderma climactericum		1	
DL858O;Stucco-keratose		1	
DL858P;Hyperkeratosis follicularis		1	
DL858Q;Keratoderma blenorrhagicum		1	
DL859;Epidermale fortykkelser UNS		1	
DL86;Keratoderma ved sygdomme klassificeret andetsteds		1	
DL869;Keratoderma ved sygdom klassificeret andetsteds		1	
DL87;Forstyrrelser i den transepidermale elimination		1	
DL870;Hyperkeratosis follicularis et parafollicularis penetrans		1	
DL871;Collagenosis perforans reactiva		1	
DL872;Elastosis perforans serpiginosa		1	
DL878;Anden form for forstyrrelse i den transepidermale elimination		1	
DL878A;Folliculitis perforans		1	
DL879;Forstyrrelse i den transepidermale elimination UNS		1	
DL88;Pusdannelse i huden		1	
DL889;Pyoderma gangraenosum		1	
DL889C;Pyodermia phagedenica		1	
DL89;Tryksår		1	
DL890;Decubitus grad I		1	
DL890A;Decubitus grad I ved neurologiske udfald		1	
DL891;Decubitus grad II		1	
DL891A;Decubitus grad II ved neurologiske udfald		1	
DL892;Decubitus grad III		1	
DL892A;Decubitus grad III ved neurologiske udfald		1	
DL893;Decubitus grad IV		1	
DL893A;Decubitus grad IV ved neurologiske udfald		1	
DL899;Decubitus UNS		1	
DL90;Atrofiske forstyrrelser i hud		1	
DL900;Sklerotisk eller atrofisk lichen		1	
DL900A;Lichen atrophicus		1	

Supplement 2 Nielsen et al. Mortality after paediatric emergency calls for patients with and without pre-existing comorbidity (2023)

DL900B;Lichen sclerosus		1		
DL901;Schweninger-Buzzis anetoderma		1		
DL902;Jadassohn-Pellizzaris anetoderma		1		
DL903;Pasini-Pierinis atrophoderma		1		
DL904;Acrodermatitis chronica atrophicans		1		
DL905;Ardannelse eller fibrose i hud		1		
DL905A;Kosmetisk generende ardannelse		1		
DL905B;Hudfibrose UNS		1		
DL905C;Adhærence i hud		1		
DL905D;Ar i hud UNS		1		
DL906;Atrofiske striae		1		
DL906A;Atrofiske striae forårsaget af steroidbehandling		1		
DL906B;Striae atrophicae adolescentium		1		
DL906C;Striae gravidarum		1		
DL908;Anden atrofisk forstyrrelse i hud		1		
DL908B;Atrophia maculosa cutis		1		
DL908C;Atrophoderma follicularis		1		
DL908D;Elastolysis		1		
DL908E;Keratoelastoidosis Costa		1		
DL908F;Lipoatrofi i hud		1		
DL908G;Lipodermatosklerose		1		
DL908H;Sklerotisk panatrofi i hud		1		
DL908I;Gowers panatrofi		1		
DL908J;Hudatrofi UNS forårsaget af steroidbehandling		1		
DL908K;Atrophia cutis senilis		1		
DL909;Atrofisk forstyrrelse i hud UNS		1		
DL91;Hypertrofiske forstyrrelser i hud		1		
DL910;Keloidt ar		1		
DL910A;Hypertrofisk ar		1		
DL918;Anden hypertrofisk forstyrrelse i hud		1		
DL918A;Acrokeratoelastoidosis		1		
DL918B;Osteoma cutis		1		

Supplement 2 Nielsen et al. Mortality after paediatric emergency calls for patients with and without pre-existing comorbidity (2023)

DL919;Hypertrofisk forstyrrelse i hud UNS		1		
DL92;Granulomatøse sygdomme i hud og underhud		1		
DL920;Annulært granulom		1		
DL920A;Granuloma annulare diffusum		1		
DL920B;Granuloma annulare perforans		1		
DL920C;Granuloma annulare subcutaneum		1		
DL921;Necrobiosis lipoidica IKA		1		
DL921A;Granuloma disciforme Miescher		1		
DL922;Granuloma faciale		1		
DL923;Fremmedlegemegranulom i hud eller underhud		1		
DL923A;Fremmedlegemegranulom i hud		1		
DL923B;Fremmedlegemegranulom i underhud		1		
DL928;Anden granulomatøse sygdom i hud eller underhud		1		
DL928A;Granuloma gluteale infantum		1		
DL928B;Reticulohistiocytært granulom		1		
DL928C;Kviksølvsgranulom		1		
DL928D;Granulomatøs keilitis		1		
DL928E;Lipogranuloma		1		
DL928F;Granuloma multiforme		1		
DL928G;Kronisk granulomatøs sygdom		1		
DL929;Granulomatøs sygdom i hud eller underhud UNS		1		
DL929A;Granulomatosis subcutis		1		
DL929B;Granulomatosis cutis		1		
DL93;Lupus erythematosus		1		
DL930;Diskoid lupus erythematosus		1		
DL930A;Lupus erythematosus UNS		1		
DL931;Lupus erythematosus cutaneus subacutus		1		
DL932;Anden form for lokaliseret lupus erythematosus		1		
DL932A;Lupus erythematosus profundus		1		
DL932B;Lupuspanniculitis		1		
DL932C;Lupus erythematosus tumidus		1		
DL94;Andre lokaliserede bindevævssygdomme		1		

Supplement 2 Nielsen et al. Mortality after paediatric emergency calls for patients with and without pre-existing comorbidity (2023)

DL940;Lokaliseret sklerodermi		1	
DL940B;Sclerodermia circumscripta		1	
DL941;Scleroderma lineare		1	
DL942;Calcinosis cutis		1	
DL942B;Calciphylaxis		1	
DL943;Sklerodaktyli		1	
DL944;Gottrons papler		1	
DL945;Poikiloderma vasculare atrophicans		1	
DL946;Ainhum		1	
DL948;Anden form for lokaliseret bindevævssygdom		1	
DL949;Lokaliseret bindevævssygdom UNS		1	
DL95;Vaskulitis begrænset til huden IKA		1	
DL950;Vasculitis livedoides		1	
DL950A;Capillaritis alba		1	
DL951;Erythema elevatum diutinum		1	
DL958;Anden vaskulitis begrænset til huden		1	
DL958A;Leukocytoklastisk vaskulitis		1	
DL958B;Urtikariel vaskulitis		1	
DL959;Vaskulitis begrænset til huden UNS		1	
DL97;Sår på ben IKA		1	
DL979;Ulcus på ben IKA		1	
DL979A;Neurogent ulcus på ben		1	
DL979B;Ulcus cruris arterioscleroticum		1	
DL979C;Ulcus perforans pedis		1	
DL979E;Ulcus pedis arterioscleroticum		1	
DL98;Andre sygdomme i hud og underhud IKA		1	
DL980;Pyogent granulom		1	
DL981;Dermatitis factitia		1	
DL981A;Excoriatio neurotica		1	
DL981B;Purpura factitia		1	
DL982;Dermatosis neutrophilica febrilis		1	
DL983;Cellulitis eosinophilica		1	

Supplement 2 Nielsen et al. Mortality after paediatric emergency calls for patients with and without pre-existing comorbidity (2023)

DL984;Kronisk ulcus i huden IKA		1	
DL984A;Tropisk ulcus UNS		1	
DL984B;Hudfissur		1	
DL984C;Ulcus i huden UNS		1	
DL984D;Hudfistel		1	
DL984E;Rhagade		1	
DL985;Mucinosis cutis		1	
DL985A;Scleromyxoedema		1	
DL985B;Lichen myxoedematosus		1	
DL985C;Fokal mucinose		1	
DL985D;Myxoedema praetibiale		1	
DL985E;Mucinosis follicularis		1	
DL985F;Reticulat erytematøs mucimose		1	
DL985G;Mucinosis papulosa		1	
DL985H;Scleroedema Buschke		1	
DL986;Anden infiltrativ sygdom i hud eller underhud		1	
DL987;Overskydende og overflødig hud og subkutant væv	0		
DL987A;Overskydende og overflødig hud og subkutant væv efter vægttab	0		
DL987B;Overskydende og overflødig hud og subkutant væv UNS	0		
DL988;Anden sygdom i hud og underhud	0		
DL988A;Lymphocytoma benigna cutis	0		
DL988B;Jessners benigne lymfocyt-infiltration i huden	0		
DL988D;Trykvable på fod	0		
DL988E;Øjenbrynsptose	0		
DL989;Sygdom i hud eller underhud UNS		1	
DL99;Andre sygdomme i hud og underhud ved sygdomme klassificeret andetsteds		1	
DL990;Amyloidosis cutis UNS		1	
DL990A;Lichen amyloidosis		1	
DL990B;Amyloidosis macularis		1	
DL990C;Amyloidosis nodularis		1	
DL998;Anden forandring i hud og underhud ved sygdom klassificeret andetsteds	0		
DL998A;Syfilitisk leukoderma	0		

DL998B;Syfilitisk alopeci	0		
DM00;Purulent ledbetændelse	0		
DM000;Artritis eller polyartritis forårsaget af stafylokokker	0		
DM000A;Artritis forårsaget af stafylokokker	0		
DM000B;Polyartritis forårsaget af stafylokokker	0		
DM001;Artritis eller polyartritis forårsaget af pneumokokker	0		
DM001A;Artritis forårsaget af pneumokokker	0		
DM001B;Polyartritis forårsaget af pneumokokker	0		
DM002;Artritis eller polyartritis forårsaget af streptokokker	0		
DM002A;Artritis forårsaget af streptokokker	0		
DM002B;Polyartritis forårsaget af streptokokker	0		
DM008;Purulent artritis eller polyartritis forårsaget af anden bakterie	0		
DM009;Purulent artritis UNS	0		
DM01;Artritis ved infektiøse og parasitære sygdommme klassificeret andetsteds	0		
DM010;Artritis ved meningokok-sygdom	0		
DM011;Artritis ved tuberkulose	0		
DM012;Artritis ved borrelia-sygdom	0		
DM013;Artritis ved anden bakteriel sygdom klassificeret andetsteds	0		
DM014;Artritis ved røde hunde	0		
DM015;Artritis ved viral sygdom klassificeret andetsteds	0		
DM016;Artritis forårsaget af svampeinfektion	0		
DM018;Artritis ved anden infektiøs eller parasitær sygdom klassificeret andetsteds	0		
DM02;Reaktive artritter	0		
DM020;Artritis efter intestinal bypassoperation	0		
DM021;Artritis efter dysenteri	0		
DM022;Artritis efter vaccination	0		
DM023;Reiters sygdom	0		
DM028;Anden reaktiv artritis	0		
DM029;Reaktiv artritis UNS	0		
DM03;Postinfektiøse og reaktive artritter ved sygdomme klassificeret andetsteds	0		
DM030;Postmeningokokartritis	0		
DM031;Postinfektiøs artritis ved syfilis	0		

Supplement 2 Nielsen et al. Mortality after paediatric emergency calls for patients with and without pre-existing comorbidity (2023)

DM031A;Cluttons led	0		
DM032;Anden postinfektiøs artritis ved sygdom klassificeret andetsteds	0		
DM032A;Postinfektiøs artritis efter virushepatitis-infektion	0		
DM032B;Postinfektiøs artritis efter Yersinia enterocolitica-infektion	0		
DM036;Reaktiv artritis ved anden sygdom klassificeret andetsteds	0		
DM05;Seropositiv leddegigt		1	
DM050;Feltys syndrom		1	
DM051;Reumatoid artritis med lungemanifestationer		1	
DM051A;Reumatoid artritis med Caplans syndrom		1	
DM051B;Reumatoid artritis med pleural effusion		1	
DM051C;Reumatoid artritis med interstitiel diffus lungefibrose		1	
DM051D;Reumatoid artritis med pleuritis		1	
DM051E;Reumatoid artritis med noduli rheumatici pulmonis		1	
DM051F;Reumatoid artritis med granulomer i lungerne		1	
DM052;Reumatoid vaskulitis		1	
DM053;Reumatoid artritis med involvering af andre organsystemer		1	
DM058;Anden form for seropositiv reumatoid artritis		1	
DM059;Seropositiv reumatoid artritis UNS		1	
DM06;Andre former for leddegigt		1	
DM060;Seronegativ reumatoid artritis		1	
DM061;Stills sygdom med debut efter det fyldte 16. år		1	
DM062;Bursitis rheumatoides		1	
DM063;Noduli rheumatici		1	
DM064;Polyarthritis inflammatorica		1	
DM068;Anden form for reumatoid artritis		1	
DM069;Reumatoid artritis UNS		1	
DM07;Psoriatisk artropati og artropati ved tarmlidelse		1	
DM070;Psoriatisk artritis i distale interfalangealled		1	
DM071;Arthritis psoriatica mutilans		1	
DM072;Psoriatisk spondylitis		1	
DM073;Anden form for psoriatisk artropati		1	
DM073A;Psoriasisartritis UNS		1	

Supplement 2 Nielsen et al. Mortality after paediatric emergency calls for patients with and without pre-existing comorbidity (2023)

DM073B;Anden psoriatisk artropati		1	
DM074;Artropati ved Crohns sygdom		1	
DM075;Artropati ved colitis ulcerosa		1	
DM076;Anden form for artropati ved anden tarmsygdom		1	
DM08;Leddegigt hos børn		1	
DM080;Juvenil reumatoid artritis		1	
DM080A;Juvenil reumatoid artritis med rheumafaktor		1	
DM080B;Juvenil reumatoid artritis uden rheumafaktor		1	
DM081;Juvenil ankyloserende spondylitis		1	
DM082;Juvenil artritis med ekstraartikulære manifestationer		1	
DM082A;Stills sygdom med debut før det fyldte 16 år		1	
DM082B;Stills sygdom UNS		1	
DM083;Juvenil seronegativ polyartritis		1	
DM084;Pauciartikulær juvenil artritis		1	
DM088;Anden form for juvenil artritis		1	
DM088A;Entesitis-relateret artritis		1	
DM089;Juvenil artritis UNS		1	
DM09;Leddegigt hos børn ved sygdomme klassificeret andetsteds		1	
DM090;Juvenil artritis ved psoriasis		1	
DM091;Juvenil artritis ved Crohns sygdom		1	
DM092;Juvenil artritis ved ulcerøs colitis		1	
DM098;Juvenil artritis ved anden sygdom klassificeret andetsteds		1	
DM10;Urinsur gigt		1	
DM100;Idiopatisk urinsur gigt		1	
DM100A;Podagra		1	
DM100B;Tophi urici		1	
DM100C;Bursitis urica		1	
DM100D;Tophi urici cordis		1	
DM101;Urinsur gigt ved blyforgiftning		1	
DM102;Urinsur gigt forårsaget af lægemiddel		1	
DM103;Urinsur gigt ved nefropati		1	
DM104;Anden form for sekundær urinsur gigt		1	

Supplement 2 Nielsen et al. Mortality after paediatric emergency calls for patients with and without pre-existing comorbidity (2023)

DM109;Urinsur gigt UNS		1	
DM11;Andre former for krystalartropati		1	
DM110;Hydroxyapatitartritis		1	
DM111;Familiær kondrokalcinose		1	
DM112;Anden form for kondrokalcinose		1	
DM112A;Kondrokalcinose UNS		1	
DM118;Anden form for krystalartropati		1	
DM119;Krystalartropati UNS		1	
DM12;Andre artropatier		1	
DM120;Kronisk postreumatisk artropati		1	
DM121;Kaschin-Becks sygdom		1	
DM122;Synovitis pigmentosa villonodularis		1	
DM123;Palindromisk reumatisme		1	
DM124;Intermitterende hydrartrose		1	
DM125;Traumatisk artropati		1	
DM128;Anden artropati IKA		1	
DM128A;Arthropathia transitoria		1	
DM13;Andre artritter		1	
DM130;Polyartritis UNS		1	
DM131;Monoartritis IKA	0		
DM131A;Coxitis simplex	0		
DM138;Anden artritis		1	
DM138A;Allergisk artritis		1	
DM139;Artritis UNS		1	
DM14;Artropatier ved andre sygdomme klassificeret andetsteds		1	
DM140;Urinsur gigt ved enzymdefekt eller anden arvelig sygdom klassificeret andetsteds		1	
DM141;Krystalartropati ved anden metabolisk sygdom		1	
DM142;Diabetisk artropati		1	
DM143;Dermatoarthritis lipoidica		1	
DM144;Artropati ved amyloidose		1	
DM145;Artropati ved anden endokrin, ernæringsbetinget eller metabolisk sygdom		1	
DM146;Neuropatisk artropati		1	

Supplement 2 Nielsen et al. Mortality after paediatric emergency calls for patients with and without pre-existing comorbidity (2023)

DM148;Artropati ved anden sygdom klassificeret andetsteds		1	
DM15;Slidgigt i flere led		1	
DM150;Primær generaliseret artrose		1	
DM151;Heberdens knuder ved artrose		1	
DM152;Bouchards knuder ved artrose		1	
DM153;Sekundær multipel artrose		1	
DM153A;Posttraumatisk polyartrose		1	
DM154;Erosiv artrose		1	
DM158;Anden polyartrose		1	
DM159;Polyartrose UNS		1	
DM16;Slidgigt i hofte		1	
DM160;Primær dobbeltsidig hofteledsartrose		1	
DM161;Primær enkeltsidig hofteledsartrose		1	
DM161A;Primær hofteledsartrose UNS		1	
DM162;Dysplastisk dobbeltsidig hofteledsartrose		1	
DM163;Dysplastisk enkeltsidig hofteledsartrose		1	
DM163A;Dysplastisk hofteledsartrose UNS		1	
DM164;Posttraumatisk dobbeltsidig hofteledsartrose		1	
DM165;Posttraumatisk enkeltsidig hofteledsartrose		1	
DM165A;Posttraumatisk hofteledsartrose UNS		1	
DM166;Anden form for sekundær dobbeltsidig hofteledsartrose		1	
DM167;Anden form for sekundær enkeltsidig hofteledsartrose		1	
DM167A;Sekundær hofteledsartrose UNS		1	
DM169;Hofteledsartrose UNS		1	
DM17;Slidgigt i knæ		1	
DM170;Primær dobbeltsidig knæledsartrose		1	
DM171;Primær enkeltsidig knæledsartrose		1	
DM171B;Primær knæledsartrose UNS		1	
DM172;Posttraumatisk dobbeltsidig knæledsartrose		1	
DM173;Posttraumatisk enkeltsidig knæledsartrose		1	
DM173A;Posttraumatisk knæledsartrose UNS		1	
DM174;Anden form for sekundær bilateral knæledsartrose		1	

DM175;Anden form for sekundær enkeltsidig knæledsartrose		1	
DM175A;Sekundær knæledsartrose UNS		1	
DM179;Knæledsartrose UNS		1	
DM18;Slidgigt i tommelfingers rodled		1	
DM180;Primær dobbeltsidig artrose i tommelfingers rodled		1	
DM181;Primær enkeltsidig artrose i tommelfingers rodled		1	
DM181A;Primær artrose i tommelfingers rodled UNS		1	
DM182;Posttraumatisk dobbeltsidig artrose i tommelfingers rodled		1	
DM183;Posttraumatisk enkeltsidig artrose i tommelfingers rodled		1	
DM183A;Posttraumatisk artrose i tommelfingers rodled UNS		1	
DM184;Anden form for sekundær bilateral artrose i tommelfingers rodled		1	
DM185;Anden form for sekundær enkeltsidig artrose i tommelfingers rodled		1	
DM185A;Sekundær artrose i tommelfingers rodled UNS		1	
DM189;Artrose i tommelfingers rodled UNS		1	
DM19;Andre former for slidgigt		1	
DM190;Primær artrose i andet (andre) led		1	
DM190A;Primær artrose UNS		1	
DM191;Posttraumatisk artrose i andet (andre) led		1	
DM191A;Posttraumatisk artrose UNS		1	
DM192;Anden sekundær artrose i andet (andre) led		1	
DM192A;Anden sekundær artrose UNS		1	
DM198;Anden artrose		1	
DM198A;Slidgigt i skulderled UNS		1	
DM199;Artrose UNS		1	
DM20;Erhvervede deformiteter i fingre og tæer	0		
DM200;Deformitet af fingre	0		
DM200A;Digitus varus manus	0		
DM200B;Digitus valgus manus	0		
DM200C;Knaphulsdeformitet	0		
DM200E;Digitus rigidus manus	0		
DM200F;Svanehalsdeformitet	0		
DM201;Erhvervet hallux valgus	0		

DM201A;Bunio	0			
DM202;Hallux rigidus	0			
DM203;Anden erhvervet deformitet af storetå	0			
DM203A;Hallux varus	0			
DM203B;Hammertå på storetå	0			
DM203C;Hallux flexus acquisitus	0			
DM204;Hammertå UNS	0			
DM205;Anden erhvervet deformitet af tå	0			
DM205A;Digitus varus acquisitus pedis, dig. II-V	0			
DM205B;Digitus valgus acquisitus pedis, dig. II-V	0			
DM205C;Digitus rigidus acquisitus pedis, dig. II-V	0			
DM205E;Digitus superponens pedis	0			
DM206;Erhvervet tådeformitet UNS	0			
DM21;Andre erhvervede deformiteter af arme og ben	0			
DM210;Valgusdeformitet IKA	0			
DM211;Varusdeformitet IKA	0			
DM212;Fleksionsdeformitet	0			
DM213;Erhvervet dropfod	0			
DM214;Erhvervet platfod	0			
DM215;Erhvervet klohånd, klofod, klumphånd eller klumpfod	0			
DM215A;Erhvervet klumphånd	0			
DM215D;Pes equino-varus acquisitus	0			
DM215E;Erhvervet klohånd	0			
DM215F;Erhvervet klofod	0			
DM215G;Erhvervet klumpfod	0			
DM216;Anden erhvervet deformitet af ankel eller fod	0			
DM216H;Pes calcaneo-valgus acquisitus	0			
DM216I;Pes cavus acquisitus	0			
DM216J;Pes calcaneus acquisitus	0			
DM216K;Pes transverso-planus	0			
DM216L;Pes metatarso-varus acquisitus	0			
DM216M;Pes plano-valgus acquisitus	0			

DM216N;Pes excavatus acquisitus	0			
DM216O;Pes valgus acquisitus	0			
DM216P;Pes equino-valgus acquisitus	0			
DM217;Erhvervet anisomeli	0			
DM218;Anden erhvervet ekstremitetsdeformitet	0			
DM218A;Cubitus valgus acquisitus	0			
DM218B;Cubitus varus acquisitus	0			
DM218C;Genu valgum acquisitum	0			
DM218D;Brevitas acquisita extremitatis	0			
DM218E;Genu varum acquisitum	0			
DM218F;Coxa vara acquisita	0			
DM218G;Genu recurvatum acquisitum	0			
DM218H;Coxa valga acquisita	0			
DM218J;Erhvervet drophånd	0			
DM219;Erhvervet ekstremitetsdeformitet UNS	0			
DM22;Sygdomme i knæskal	0			
DM220;Habituel patellaluksation	0			
DM221;Habituel patellasubluksation	0			
DM222;Patellofemoral lidelse	0			
DM222A;Patellofemoralt malalignmentsyndrom	0			
DM223;Anden forskydning af patella	0			
DM224;Kondromalaci i patella	0			
DM228;Anden sygdom i patella	0			
DM228A;Deformatio patellae UNS	0			
DM229;Sygdom i patella UNS	0			
DM23;Lidelser i knæled	0			
DM230;Meniskcyste	0			
DM231;Medfødt diskoid menisk	0			
DM232;Gammel menisklæsion	0			
DM233;Anden menisklidelse	0			
DM233A;Meniskforkalkning	0			
DM233B;Meniskdegeneration	0			

DM234;Mus i knæled	0			
DM235;Kronisk instabilitet i knæled	0			
DM236;Anden spontan ruptur af knæledsligament	0			
DM236A;Spontan ruptur af bageste korsbånd i knæ	0			
DM236B;Spontan ruptur af kollateralligament i knæ	0			
DM236C;Spontan ruptur af knæledsligament UNS	0			
DM236D;Spontan ruptur af forreste korsbånd i knæ	0			
DM238;Anden lidelse i knæled	0			
DM238A;Slaphed i knæledsligament	0			
DM238B;Knæledsklik	0			
DM239;Knæledslidelse UNS	0			
DM24;Andre ledlidelser	0			
DM240;Ledmus UNS	0			
DM241;Anden lidelse i ledbrusk	0			
DM242;Lidelse i ligament	0			
DM242A;Ledinstabilitet efter tidligere ligamentskade	0			
DM242B;Ligamentslaphed UNS	0			
DM243;Luksation eller subluksation i led IKA	0			
DM243A;Luksation af led IKA	0			
DM243B;Subluksation af led IKA	0			
DM244;Habituelle luksationer eller subluksationer	0			
DM244A;Habituel luksation af led UNS	0			
DM244B;Habituel subluksation af led UNS	0			
DM244C;Habituel luksation af albueled	0			
DM244D;Spontan luksation af skulderled	0			
DM244E;Habituel luksation af skulderled	0			
DM244E1;Anterior skulderinstabilitet	0			
DM244E2;Posterior skulderinstabilitet	0			
DM245;Ledkontraktur	0			
DM246;Ankylose	0			
DM246A;Ankylosis manus	0			
DM246B;Ankylosis multiplex	0			

DM246C;Ankylosis olecrani	0		
DM246D;Ankylose UNS	0		
DM246E;Ankylosis humeri	0		
DM246F;Ankylosis pedis	0		
DM246G;Ankylosis genus	0		
DM246H;Ankylosis cubiti	0		
DM246I;Ankylosis digitorum manus	0		
DM246J;Ankylosis coxae	0		
DM246K;Ankylosis carpi	0		
DM246L;Ankylosis talocruralis	0		
DM246M;Ankylosis humeroscapularis	0		
DM246N;Ankylosis radiocarpi	0		
DM246O;Ankylosis tarsi	0		
DM247;Protrusio acetabuli	0		
DM248;Anden ledlidelse IKA	0		
DM248A;Coxa irritabilis	0		
DM249;Ledsygdom UNS	0		
DM25;Andre ledlidelser IKA	0		
DM250;Hæmartrose	0		
DM251;Ledfistel	0		
DM252;Ustabilt led	0		
DM253;Anden ledinstabilitet	0		
DM254;Væskeansamling i led	0		
DM255;Ledsmerter	0		
DM256;Ledstivhed IKA	0		
DM256A;Ledstivhed uden ankylose	0		
DM257;Osteofyt	0		
DM258;Anden ledlidelse	0		
DM258A;Coxitis simplex (findes ikke i SKS)	0		
DM258B;Scapula crepitans (Snapping scapula)	0		
DM259;Ledlidelse UNS	0		
DM30;Polyarteritis nodosa og beslægtede sygdomme		1	

Supplement 2 Nielsen et al. Mortality after paediatric emergency calls for patients with and without pre-existing comorbidity (2023)

DM300;Polyarteritis nodosa		1	
DM301;Polyarteritis med asthma bronchiale		1	
DM301A;Angiitis granulomatosa allergica		1	
DM302;Polyarteritis juvenilis		1	
DM303;Mukokutant lymfeknudesyndrom (Kawasaki)		1	
DM308;Anden sygdom beslægtet med polyarteritis nodosa		1	
DM308A;Polyangiitis blandingssyndrom		1	
DM31;Andre nekrotiserende vaskuliter		1	
DM310;Angiitis hypersensitiva		1	
DM310A;Goodpastures syndrom		1	
DM310B;Angiitis hypersensitiva ved Schönlein-Henochs purpura		1	
DM311;Microangiopathia thrombotica		1	
DM311A;Trombotisk trombocytopenisk purpura		1	
DM311B;Buergers sygdom		1	
DM312;Letalt midtlinjegranulom		1	
DM313;Wegeners granulomatose		1	
DM313A;Nekrotiserende respiratorisk granulomatose		1	
DM314;Arcus aortae-syndrom		1	
DM315;Kæmpecellearteritis med reumatisk polymyalgi		1	
DM315A;Arteritis temporalis med reumatisk polymyalgi		1	
DM316;Anden kæmpecellearteritis		1	
DM316A;Arteritis temporalis uden reumatisk polymyalgi		1	
DM317;Mikroskopisk polyangiitis		1	
DM318;Anden nekrotiserende vaskulitis		1	
DM318A;Vaskulitis med nedsat komplement		1	
DM319;Nekrotiserende vaskulitis UNS		1	
DM32;Systemisk lupus erythematosus		1	
DM320;Systemisk lupus erythematosus forårsaget af lægemiddel		1	
DM321;Systemisk lupus erythematosus med organinvolvering		1	
DM328;Anden form for systemisk lupus erythematosus		1	
DM329;Systemisk lupus erythematosus UNS		1	
DM33;Dermatopolymyositis		1	

DM330;Dermatomyositis juvenilis		1	
DM331;Anden dermatomyositis		1	
DM332;Polymyositis		1	
DM339;Dermatopolymyositis UNS		1	
DM34;Systemisk sklerodermi		1	
DM340;Progressiv systemisk sklerodermi		1	
DM341;CREST-syndrom		1	
DM342;Systemisk sklerodermi forårsaget af lægemiddel eller kemikalie		1	
DM342A;Systemisk sklerodermi forårsaget af kemikalie		1	
DM342B;Systemisk sklerodermi forårsaget af lægemiddel		1	
DM348;Anden form for systemisk sklerodermi		1	
DM348B;Systemisk sklerodermi med akrosklerose		1	
DM349;Systemisk sklerodermi UNS		1	
DM35;Andre generaliserede bindevævssygdomme		1	
DM350;Sjögrens syndrom		1	
DM350A;Keratokonjunktivitis sicca ved Sjögrens syndrom		1	
DM350B;Interstitiel pneumonitis ved Sjögrens syndrom		1	
DM350C;Xerostomi ved Sjögrens syndrom		1	
DM350D;Myopati ved Sjögrens syndrom		1	
DM350E;Tubulointerstiel nefropati ved Sjögrens syndrom		1	
DM351;Andet blandingssyndrom ved generaliseret bindevævssygdom		1	
DM351A;Mixed connective tissue disease		1	
DM351B;VEXAS syndrom		1	
DM352;Behçets sygdom		1	
DM353;Reumatisk polymyalgi		1	
DM354;Eosinofil fasciitis		1	
DM355;Fibrosclerosis multifocalis		1	
DM356;Recidiverende pannikulitis		1	
DM357;Hypermobilitetssyndrom		1	
DM358;Anden generaliseret bindevævssygdom		1	
DM359;Generaliseret bindevævssygdom UNS		1	
DM36;Generaliserede bindevævssygdomme ved sygdomme klassificeret andetsteds		1	

Supplement 2 Nielsen et al. Mortality after paediatric emergency calls for patients with and without pre-existing comorbidity (2023)

DM360;Dermato(poly)myositis ved neoplastisk sygdom klassificeret andetsteds		1	
DM361;Artropati ved neoplastisk sygdom klassificeret andetsteds		1	
DM362;Artropati ved hæmofili		1	
DM363;Artropati ved anden blodsygdom klassificeret andetsteds		1	
DM364;Artropati ved hypersensitivitetsreaktioner klassificeret andetsteds		1	
DM368;Generaliseret bindevævssygdom ved sygdom klassificeret andetsteds		1	
DM40;Kyfose og lordose		1	
DM400;Postural kyfose		1	
DM401;Anden form for sekundær kyfose		1	
DM401A;Sekundær kyfose UNS		1	
DM402;Anden eller ikke specificeret kyfose		1	
DM402A;Kyfose UNS		1	
DM403;Columna recta		1	
DM404;Anden form for lordose		1	
DM404A;Erhvervet lordose		1	
DM404B;Postural lordose		1	
DM404C;Hyperlordosis		1	
DM405;Lordose UNS		1	
DM41;Skoliose		1	
DM410;Idiopatisk skoliose hos barn		1	
DM410A;Idiopatisk kyfoskoliose hos barn		1	
DM411;Idiopatisk skoliose hos ung		1	
DM411A;Idiopatisk kyfoskoliose hos ung		1	
DM412;Anden form for idiopatisk skoliose		1	
DM413;Torakogen skoliose		1	
DM413A;Torakogen kyfoskoliose		1	
DM414;Neuromuskulær skoliose		1	
DM414A;Sekundær skoliose ved Friedreichs ataksi		1	
DM414B;Sekundær skoliose ved cerebral parese		1	
DM414C;Neuromuskulær kyfoskoliose		1	
DM414D;Sekundær kyfoskoliose ved cerebral parese		1	
DM414E;Sekundær kyfoskoliose ved Friedreichs ataksi		1	

DM414F;Sekundær kyfoskoliose ved poliomyelitis		1	
DM415;Anden form for sekundær skoliose		1	
DM415A;Sekundær skoliose UNS		1	
DM415B;Sekundær kyfoskoliose UNS		1	
DM418;Anden form for skoliose		1	
DM419;Skoliose UNS		1	
DM42;Osteokondrose i rygsøjlen		1	
DM420;Juvenil osteokondrose i rygsøjlen		1	
DM421;Adult osteokondrose i rygsøjlen		1	
DM429;Osteokondrose i rygsøjlen UNS		1	
DM43;Andre deformerende rygsygdomme		1	
DM430;Spondylolyse		1	
DM431;Spondylolistese		1	
DM432;Blokhvirveldannelse i rygsøjlen		1	
DM432A;Ankylose i rygsøjlen		1	
DM433;Habituel atlantoaksial subluksation med myelopati		1	
DM434;Anden habituel atlantoaksial subluksation		1	
DM434A;Habituel atlantoaksial subluksation uden myelopati		1	
DM435;Anden form for habituel subluksation i rygsøjlen		1	
DM436;Torticollis		1	
DM438;Anden deformerende ryglidelse		1	
DM439;Deformerende ryglidelse UNS		1	
DM45;Spondylitis ankylopoietica		1	
DM459;Ankyloserende spondylitis		1	
DM46;Andre inflammatoriske spondylopatier		1	
DM460;Entesopati i rygsøjlen		1	
DM461;Sakroiliitis IKA		1	
DM462;Osteomyelitis i ryghvirvel		1	
DM463;Infektiøs diskitis i rygsøjlen		1	
DM463A;Infektiøs discitis intervertebralis postoperativa		1	
DM464;Diskitis UNS		1	
DM465;Anden form for infektiøs spondylopati		1	

DM465A;Infektiøs spondylitis UNS		1	
DM468;Anden form for inflammatorisk spondylopati		1	
DM468A;Seronegativ spondylartritis		1	
DM469;Inflammatorisk spondylopati UNS		1	
DM47;Spondylose		1	
DM470;Kompressionssyndrom ved forsnævring omkring arteria spinalis anterior eller arteria vertebralis		1	
DM470A;Arteria spinalis anterior-syndrom		1	
DM471;Anden spondylose med myelopati		1	
DM471A;Spondylose med myelopati		1	
DM471B;Kompression af rygmarven forårsaget af spondylose		1	
DM471C;Spondylose i halshvirvelsøjlen med myelopati og tetraplegi			2
DM472;Anden spondylose med radikulopati		1	
DM472A;Spondylose med radikulopati		1	
DM472B;Spondylose med neuropati		1	
DM478;Anden spondylose		1	
DM478A;Torakal spondylose		1	
DM478B;Total spondylose		1	
DM478C;Cervikal spondylose		1	
DM478D;Lumbosakral spondylose		1	
DM478E;Lumbal spondylose		1	
DM479;Spondylose UNS		1	
DM48;Andre sygdomme i rygsøjlen		1	
DM480;Spinalstenose		1	
DM481;Hyperostosis ankylotica		1	
DM481A;Hyperostosis skeletalis idiopathica diffusa		1	
DM482;Arthrosis processus spinosi vertebrarum lumbalium		1	
DM483;Traumatisk spondylopati		1	
DM484;Spontanfraktur af ryghvirvel		1	
DM485;Sammenfald af ryghvirvel IKA		1	
DM485A;Kileformet sammenfald af ryghvirvel		1	
DM488;Anden form for spondylopati		1	
DM488A;Ossificatio ligamenti longitudinalis posterioris		1	

DM489;Spondylopati UNS		1	
DM49;Sygdomme i rygsøjlen ved sygdomme klassificeret andetsteds		1	
DM490;Tuberkuløs spondylitis		1	
DM491;Spondylitis ved brucellose		1	
DM492;Enterobakteriel spondylitis		1	
DM493;Spondylitis ved anden infektiøs eller parasitær sygdom klassificeret andetsted		1	
DM494;Neuropatisk spondylopati		1	
DM495;Sammenfald af ryghvirvel ved sygdom klassificeret andetsteds		1	
DM498;Spondylopati ved anden sygdom klassificeret andetsteds		1	
DM50;Sygdomme i halshvirvelsøjlens båndskiver		1	
DM500;Cervikal diskusprolaps med myelopati		1	
DM500A;Cervikal diskusprolaps II/III med myelopati		1	
DM500B;Cervikal diskusprolaps III/IV med myelopati		1	
DM500C;Cervikal diskusprolaps IV/V med myelopati		1	
DM500D;Cervikal diskusprolaps V/VI med myelopati		1	
DM500E;Cervikal diskusprolaps VI/VII med myelopati		1	
DM500F;Cervicotorakal diskusprolaps med myelopati		1	
DM501;Cervikal diskusprolaps med radikulopati		1	
DM501A;Cervikal diskusprolaps II/III med radikulopati		1	
DM501B;Cervikal diskusprolaps III/IV med radikulopati		1	
DM501C;Cervikal diskusprolaps IV/V med radikulopati		1	
DM501D;Cervikal diskusprolaps V/VI med radikulopati		1	
DM501E;Cervikal diskusprolaps VI/VII med radikulopati		1	
DM501F;Cervicotorakal diskusprolaps med radikulopati		1	
DM502;Anden form for cervikal diskusprolaps		1	
DM503;Anden form for cervikal diskusdegeneration		1	
DM508;Anden sygdom i cervikal båndskive		1	
DM509;Sygdom i cervikal båndskive UNS		1	
DM51;Sygdomme i lumbale og torakale båndskiver		1	
DM510;Lumbal eller torakal diskusprolaps med myelopati		1	
DM510A;Lumbal diskusprolaps I/II med myelopati		1	
DM510G;Torakal diskusprolaps med myelopati		1	

DM511;Lumbal eller torakal diskusprolaps med radikulopati		1	
DM511A;Lumbal diskusprolaps I/II med radikulopati		1	
DM511B;Lumbal diskusprolaps II/III med radikulopati		1	
DM511C;Lumbal diskusprolaps III/IV med radikulopati		1	
DM511D;Lumbal diskusprolaps IV/V med radikulopati		1	
DM511E;Lumbosakral diskusprolaps med radikulopati		1	
DM511F;Lumbal diskusprolaps UNS med radikulopati		1	
DM511H;Torakal diskusprolaps med radikulopati		1	
DM511I;Torakolumbal diskusprolaps med radikulopati		1	
DM512;Anden form for torakolumbal diskusprolaps		1	
DM512A;Lumbal diskusprolaps I/II UNS		1	
DM512B;Lumbal diskusprolaps II/III UNS		1	
DM512C;Lumbal diskusprolaps III/IV UNS		1	
DM512D;Lumbal diskusprolaps IV/V UNS		1	
DM512E;Lumbosakral diskusprolaps UNS		1	
DM512F;Lumbal diskusprolaps UNS		1	
DM512G;Torakal diskusprolaps UNS		1	
DM513;Anden torakal eller lumbal diskusdegeneration		1	
DM513A;Lumbal diskusdegeneration UNS		1	
DM513B;Torakal diskusdegeneration UNS		1	
DM513C;Diskusforkalkning		1	
DM514;Schmorlsk impression		1	
DM518;Anden sygdom i lumbal eller torakal båndskive		1	
DM519;Sygdom i lumbal eller torakal båndskive UNS		1	
DM53;Andre ryglidelser IKA		1	
DM530;Cervikokranialt syndrom		1	
DM530A;Posteriort cervikalt sympatikussyndrom		1	
DM531;Cervikobrakialt syndrom		1	
DM532;Instabilitet i rygsøjlen		1	
DM532A;Luxatio spontanea sacroiliaca		1	
DM533;Sygdom i sakro-coccygeale region IKA		1	
DM533A;Coccygodynia		1	

DM533B;Sacrocoxalgia		1	
DM538;Anden ryglidelse		1	
DM539;Ryglidelse UNS		1	
DM54;Rygsmerter		1	
DM540;Pannikulitis i nakken eller ryggen		1	
DM540A;Pannikulitis i nakken		1	
DM540B;Pannikulitis i ryggen		1	
DM541;Radikulopati UNS		1	
DM541A;Radiculitis lumbosacralis		1	
DM541B;Radiculitis lumbalis		1	
DM541C;Radiculitis brachialis		1	
DM541D;Radiculitis thoracalis		1	
DM542;Cervikale rygsmerter		1	
DM543;Ischias		1	
DM544;Lændesmerter med ischias		1	
DM545;Lændesmerter UNS		1	
DM546;Torakale rygsmerter		1	
DM548;Andre rygsmerter		1	
DM549;Rygsmerter UNS		1	
DM60;Myositis		1	
DM600;Infektiøs myositis		1	
DM600A;Pyomyositis tropica		1	
DM601;Interstitiel myositis		1	
DM602;Fremmedlegemegranulom i bløddelsvæv IKA		1	
DM608;Anden form for myositis		1	
DM608A;Absces i muskulatur		1	
DM608A1;Psoasabsces		1	
DM609;Myositis UNS		1	
DM61;Forkalkning og forbening af muskel		1	
DM610;Myositis ossificans traumatica		1	
DM611;Myositis ossificans progressiva		1	
DM612;Forkalkning eller forbening af muskel ved lammelse			2

DM613;Forkalkning eller forbening af muskel ved forbrænding		1	
DM614;Anden form for forkalkning af muskelvæv		1	
DM615;Anden form for forbening af muskelvæv		1	
DM619;Forkalkning eller forbening af muskel UNS		1	
DM62;Andre muskelsygdomme		1	
DM620;Muskeldiastase		1	
DM620A;Rectusdiastase		1	
DM621;Anden ikke-traumatisk muskelruptur		1	
DM621A;Ikke-traumatisk muskelruptur UNS		1	
DM622;Iskæmisk muskelinfarkt		1	
DM622A;Ikke-traumatisk kompartmentsyndrom		1	
DM623;Immobilitetssyndrom			2
DM624;Muskelkontraktur			2
DM625;Muskelatrofi IKA		1	
DM625A;Muskelatrofi forårsaget af inaktivitet IKA		1	
DM626;Muskelspændinger		1	
DM626A;Muskeloveranstrengelse UNS		1	
DM628;Anden muskelsygdom		1	
DM628A;Muskelhernie		1	
DM628B;Idiopatisk rhabdomyolyse		1	
DM629;Muskelsygdom UNS		1	
DM63;Muskelsygdomme ved sygdomme klassificeret andetsteds		1	
DM630;Myositis ved bakteriel sygdom klassificeret andetsteds		1	
DM631;Myositis ved protozoisk eller parasitær sygdom klassificeret andetsteds		1	
DM632;Myositis ved anden infektiøs sygdom klassificeret andetsteds		1	
DM633;Myositis ved sarkoidose		1	
DM638;Anden muskelsygdom ved sygdom klassificeret andetsteds		1	
DM65;Betændelse i ledkapselhinde og seneskede		1	
DM650;Purulent tenosynovitis		1	
DM651;Anden infektiøs tenosynovitis		1	
DM652;Tendinitis med forkalkning		1	
DM653;Springfinger		1	

DM654;Tenovaginitis styloideae radii		1	
DM658;Anden form for synovitis eller tenosynovitis		1	
DM658A;Anden form for tenosynovitis		1	
DM658B;Anden form for synovitis		1	
DM659;Synovitis eller tenosynovitis UNS		1	
DM659A;Tenosynovitis UNS		1	
DM659B;Synovitis UNS		1	
DM66;Spontanruptur af ledkapselhinder og sener		1	
DM660;Ruptur af Baker-cyste		1	
DM661;Spontan synovialisruptur		1	
DM661A;Ruptura cystis synovialis UNS		1	
DM662;Spontan ekstensorseneruptur		1	
DM663;Spontan fleksorseneruptur		1	
DM664;Anden spontan seneruptur		1	
DM665;Spontan seneruptur UNS		1	
DM67;Andre sygdomme i ledkapselhinder og sener		1	
DM670;Erhvervet forkortelse af akillessene		1	
DM671;Anden senekontraktur		1	
DM672;Synovialishypertrofi IKA		1	
DM673;Forbigående synovitis		1	
DM673A;Toksisk synovitis		1	
DM674;Ganglion		1	
DM678;Anden sygdom i ledkapselhinde eller sene		1	
DM679;Sygdom i ledkapselhinde eller sene UNS		1	
DM68;Sygdomme i ledkapselhinder og sener ved sygdomme klassificeret andetsteds		1	
DM680;Betændelse i ledkapselhinde eller seneskedehinde ved bakteriel sygdom klassificeret andetsteds		1	
DM680G;Betændelse i ledkapselhinde ved bakteriel sygdom klassificeret andetsteds		1	
DM680H;Betændelse i seneskedehinde ved bakteriel sygdom klassificeret andetsteds		1	
DM688;Anden sygdom i ledkapselhinde eller sene ved sygdom klassificeret andetsteds		1	
DM70;Bløddelsgigt opstået ved belastning, overbelastning eller tryk		1	
DM700;Kronisk krepiterende synovitis i hånd eller håndled		1	
DM700A;Kronisk krepiterende synovitis i håndled		1	

DM700B;Kronisk krepiterende synovitis i hånd		1	
DM701;Bursitis i hånd		1	
DM702;Bursitis olecrani		1	
DM703;Anden bursitis i albue		1	
DM704;Bursitis i slimsækken over knæskallen		1	
DM705;Anden bursitis i knæ		1	
DM705A;Pes anserinus-bursitis		1	
DM706;Bursitis trochanterica		1	
DM706A;Tendinitis trochanterica		1	
DM707;Anden bursitis i hofte		1	
DM707A;Bursitis ischiadica		1	
DM708;Anden bløddelsgigt opstået ved belastning, overbelastning eller tryk		1	
DM709;Bløddelsgigt UNS opstået ved belastning, overbelastning eller tryk		1	
DM71;Anden sygdom i slimsæk		1	
DM710;Purulent bursitis		1	
DM711;Anden infektiøs bursitis		1	
DM712;Synovialcyste i knæhase		1	
DM713;Anden bursacyste		1	
DM713A;Synovialcyste UNS		1	
DM713B;Cervikal synovialcyste		1	
DM713B1;Cervikal synovialcyste med myelopati		1	
DM713B2;Cervikal synovialcyste med radikulopati		1	
DM713C;Torakal synovialcyste		1	
DM713C1;Torakal synovialcyste med myelopati		1	
DM713C2;Torakal synovialcyste med radikulopati		1	
DM713D;Lumbal synovialcyste		1	
DM713D1;Lumbal synovialcyste med myelopati		1	
DM713D2;Lumbal synovialcyste med radikulopati		1	
DM714;Bursitis med forkalkning		1	
DM715;Anden bursitis IKA		1	
DM718;Anden sygdom i slimsæk		1	
DM719;Sygdom i slimsæk UNS		1	

DM719A;Bursitis UNS		1	
DM72;Fibroblastsygdomme		1	
DM720;Dupuytrens kontraktur		1	
DM720A;Fasciitis palmaris (Dupuytren), recidiv		1	
DM721;Knofortykkelse		1	
DM722;Contractura aponeuroseos plantaris		1	
DM724;Pseudosarkomatøs fibromatose		1	
DM724A;Nodulær fasciitis		1	
DM726;Nekrotiserende bløddelsinfektion		1	
DM728;Anden fibroblastsygdom		1	
DM728A;Infantil aggressiv fibromatose		1	
DM728B;Infantil myofibromatose		1	
DM728C;Infantil digital fibromatose		1	
DM728D;Retroperitoneal fibrose uden hydronefrose		1	
DM729;Fibroblastsygdom UNS		1	
DM729A;Fasciitis UNS		1	
DM729B;Fibromatosis UNS		1	
DM73;Bløddelsreumatisme ved sygdomme klassificeret andetsteds		1	
DM730;Gonoroisk bursitis		1	
DM731;Syfilitisk bursitis		1	
DM738;Anden bløddelsreumatisme ved sygdom klassificeret andetsteds		1	
DM75;Skulderlidelser		1	
DM750;Periarthrosis humeroscapularis		1	
DM751;Rotator cuff-syndrom		1	
DM751A;Supraspinatussyndrom		1	
DM751B;Tendinitis supraspinata		1	
DM751C;Ruptura non traumatica tendinis supraspinati		1	
DM752;Bicepstendinitis		1	
DM753;Tendinitis med forkalkning i skulderen		1	
DM753A;Bursitis med forkalkning i skulderen		1	
DM754;Afklemningssyndrom i skulder		1	
DM755;Bursitis i skulder		1	

DM755A;Subakromial bursitis		1	
DM758;Anden skulderlidelse		1	
DM759;Skulderlidelse UNS		1	
DM76;Entesopatier på ben		1	
DM760;Tendinitis glutealis		1	
DM761;Tendinitis iliopsoas		1	
DM762;Calcar cristae iliacae		1	
DM763;Pes anserinus-syndrom		1	
DM763A;Bursitis sartoria		1	
DM763B;Bursitis anserina		1	
DM764;Tibial kollateral bursitis		1	
DM765;Tendinitis patellaris		1	
DM766;Achilles tendinitis		1	
DM766A;Achilles bursitis		1	
DM767;Tendinitis peronealis		1	
DM768;Anden entesopati på underekstremitet		1	
DM768A;Tibialis anterior-syndrom		1	
DM768B;Tibialis posterior tendinitis		1	
DM769;Entesopati på underekstremitet UNS		1	
DM77;Andre entesopatier		1	
DM770;Epicondylitis medialis		1	
DM771;Epicondylitis lateralis		1	
DM772;Periartritis i håndled		1	
DM773;Hælspore		1	
DM774;Metatarsalgia		1	
DM775;Anden entesopati på fod		1	
DM778;Anden entesopati IKA		1	
DM779;Entesopati UNS		1	
DM779A;Tendinitis UNS		1	
DM79;Anden bløddelsreumatisme IKA		1	
DM790;Reumatisme UNS		1	
DM791;Myalgi		1	

DM792;Neuralgi eller neuritis UNS		1	
DM792A;Neuralgi UNS		1	
DM792B;Neuritis UNS		1	
DM793;Pannikulitis UNS		1	
DM793A;Pannikulitis ved antitrypsinmangel		1	
DM793B;Pannikulitis ved pankreatitis		1	
DM793C;Pannikulitis forårsaget af kulde		1	
DM793D;Panniculitis migratorica nodularis		1	
DM793E;Panniculitis factitia		1	
DM794;Hypertrophia corporis adiposi infrapatellaris		1	
DM795;Retineret fremmedlegeme i bløddel		1	
DM796;Ekstremitetssmerter		1	
DM797;Fibromyalgi		1	
DM797A;Fibromyositis		1	
DM798;Anden bløddelsreumatisme		1	
DM798A;Fibrositis nodularis chronica rheumatoides		1	
DM798B;Noduli Jaccoud		1	
DM798C;Bækkenløsning efter tidligere graviditet		1	
DM798D;Bækkenløsning UNS		1	
DM799;Bløddelsreumatisme UNS		1	
DM80;Osteoporose med patologisk fraktur		1	
DM800;Postmenopausal osteoporose med patologisk fraktur		1	
DM801;Osteoporose efter ooforektomi med patologisk fraktur		1	
DM802;Immobilisationsosteoporose med patologisk fraktur		1	
DM803;Osteoporose med patologisk fraktur forårsaget af malabsorption efter operation		1	
DM804;Osteoporose med patologisk fraktur forårsaget af lægemiddel		1	
DM805;Idiopatisk osteoporose med patologisk fraktur		1	
DM808;Anden form for osteoporose med patologisk fraktur		1	
DM809;Osteoporose UNS med patologisk fraktur		1	
DM809A;Osteoporose UNS med patologisk håndledsfraktur		1	
DM809B;Osteoporose UNS med patologisk hoftefraktur		1	
DM809C;Osteoporose UNS med patologisk fraktur i rygsøjlen		1	

DM81;Osteoporose uden patologisk fraktur		1	
DM810;Postmenopausal osteoporose		1	
DM811;Osteoporose efter ooforektomi		1	
DM812;Immobilisationsosteoporose			2
DM813;Osteoporose forårsaget af malabsorption efter operation		1	
DM814;Osteoporose forårsaget af lægemiddel		1	
DM815;Idiopatisk osteoporose		1	
DM816;Lokaliseret osteoporose		1	
DM818;Anden osteoporose		1	
DM818A;Senil osteoporose		1	
DM819;Osteoporose UNS		1	
DM82;Osteoporose ved sygdom klassificeret andetsteds		1	
DM820;Osteoporose ved myelomatose		1	
DM821;Osteoporose ved endokrin sygdom		1	
DM828;Osteoporose ved andre sygdomme klassificeret andetsteds		1	
DM83;Osteomalaci hos voksne		1	
DM830;Puerperal osteomalaci		1	
DM831;Senil osteomalaci		1	
DM832;Osteomalaci hos voksen forårsaget af malabsorption		1	
DM832A;Osteomalaci hos voksen forårsaget af malabsorption efter operation		1	
DM833;Osteomalaci hos voksen forårsaget af fejlernæring		1	
DM834;Aluminium-knoglesygdom		1	
DM835;Osteomalaci forårsaget af lægemiddel		1	
DM838;Anden form for osteomalaci hos voksen		1	
DM839;Osteomalaci hos voksen UNS		1	
DM84;Forstyrrelser i knoglekontinuitet		1	
DM840;Fraktur med ufuldstændig heling		1	
DM841;Pseudartrose		1	
DM842;Fraktur med forsinket heling		1	
DM843;Stressfraktur IKA		1	
DM844;Patologisk fraktur IKA		1	
DM844A;Patologisk fraktur UNS		1	

Supplement 2 Nielsen et al. Mortality after paediatric emergency calls for patients with and without pre-existing comorbidity (2023)

DM845;Fraktur helet i fejlstilling		1	
DM848;Anden forstyrrelse i knoglekontinuitet		1	
DM849;Forstyrrelse i knoglekontinuitet UNS		1	
DM85;Andre forstyrrelser i knogletæthed og knoglestruktur		1	
DM850;Fibrøs monostotisk dysplasi		1	
DM851;Knoglefluorose		1	
DM852;Hyperostosis cranii		1	
DM852A;Hyperostosis frontalis interna		1	
DM853;Osteitis condensans		1	
DM854;Solitær knoglecyste		1	
DM855;Knoglecyste forårsaget af aneurisme		1	
DM856;Anden knoglecyste		1	
DM858;Anden forstyrrelse i knogletæthed eller knoglestruktur		1	
DM858A;Osteopeni		1	
DM859;Forstyrrelse i knogletæthed eller knoglestruktur UNS		1	
DM86;Knoglemarvsbetændelse		1	
DM860;Akut hæmatogen osteomyelitis		1	
DM861;Anden akut osteomyelitis		1	
DM862;Subakut osteomyelitis		1	
DM863;Kronisk multifokal osteomyelitis		1	
DM864;Kronisk osteomyelitis med fistel		1	
DM865;Anden kronisk hæmatogen osteomyelitis		1	
DM865A;Kronisk hæmatogen osteomyelitis UNS		1	
DM866;Anden kronisk osteomyelitis		1	
DM868;Anden osteomyelitis		1	
DM868A;Brodies absces		1	
DM869;Osteomyelitis UNS		1	
DM869A;Periostal absces UNS		1	
DM869B;Periostitis UNS		1	
DM869C;Ostitis UNS		1	
DM87;Knoglenekrose		1	
DM870;Idiopatisk aseptisk knoglenekrose		1	

DM871;Knoglenekrose forårsaget af lægemiddel		1	
DM872;Posttraumatisk knoglenekrose		1	
DM873;Anden form for sekundær knoglenekrose		1	
DM878;Anden form for knoglenekrose		1	
DM879;Knoglenekrose UNS		1	
DM88;Pagets knoglesygdom		1	
DM880;Pagets sygdom i kranieknogle		1	
DM888;Pagets sygdom i anden knogle		1	
DM889;Pagets knoglesygdom UNS		1	
DM89;Andre knoglesygdomme		1	
DM890;Refleksdystrofi		1	
DM891;Patologisk epifyselukning		1	
DM892;Anden vækst- eller udviklingsforstyrrelse i knogle		1	
DM893;Knoglehypertrofi		1	
DM894;Anden hypertrofisk osteoartropati		1	
DM894A;Osteoarthropathia hypertrophica pulmonalis		1	
DM894B;Pachydermoperiostosis		1	
DM894C;Hypertrofisk osteoartropati		1	
DM895;Osteolysis		1	
DM896;Osteopati efter poliomyelitis		1	
DM898;Anden knoglesygdom		1	
DM898A;Infantil kortikal hyperostose		1	
DM898B;Posttraumatisk subperiostal ossifikation		1	
DM899;Knoglesygdom UNS		1	
DM90;Knoglelidelser ved sygdommme klassificeret andetsteds		1	
DM900;Knogletuberkulose		1	
DM901;Periostitis ved anden infektiøs sygdom klassificeret andetsteds		1	
DM902;Osteopati ved anden infektiøs sygdom klassificeret andetsteds		1	
DM903;Osteonekrose ved dykkersyge		1	
DM904;Osteonekrose ved hæmoglobinopati		1	
DM905;Osteonekrose ved anden sygdom klassificeret andetsteds		1	
DM906;Ostitis deformans ved neoplastisk klassificeret andetsteds		1	

DM907;Patologisk fraktur ved neoplastisk sygdom klassificeret andetsteds		1	
DM908;Osteopati ved anden sygdom klassificeret andetsteds		1	
DM91;Brusklidelse i hofte og bækken hos unge		1	
DM910;Juvenil bækkenosteokondrose		1	
DM910A;Osteochondrosis juvenilis symphysis pubis		1	
DM910B;Osteochondrosis juvenilis cristae iliacae		1	
DM910C;Osteochondrosis juvenilis synchondrosis ischiopubica		1	
DM910D;Osteochondrosis juvenilis acetabuli		1	
DM911;Juvenil deformerende hofteosteokondrose		1	
DM912;Coxa plana		1	
DM913;Pseudocoxalgia		1	
DM918;Anden form for juvenil brusklidelse i hofte eller bækken		1	
DM918A;Juvenil osteokondrose efter behandling af kongenit hofteluksation		1	
DM919;Juvenil brusklidelse i hofte eller bækken UNS		1	
DM92;Andre brusklidelser hos unge		1	
DM920;Juvenil osteokondrose i overarm		1	
DM920A;Osteochondrosis juvenilis capitis humeri		1	
DM920B;Osteochondrosis juvenilis capituli humeri		1	
DM921;Juvenil osteokondrose i underarm		1	
DM921A;Osteochondrosis juvenilis ulnae		1	
DM921B;Osteochondrosis juvenilis capitis ulnae		1	
DM921C;Osteochondrosis juvenilis radii		1	
DM921D;Osteochondrosis juvenilis capitis radii		1	
DM922;Juvenil osteokondrose i hånd		1	
DM922A;Osteochondrosis capitis metacarpi		1	
DM922B;Osteochondrosis corporis lunati		1	
DM922C;Osteochondrosis ossis navicularis manus		1	
DM923;Anden juvenil osteokondrose i overekstremitet		1	
DM924;Juvenil osteokondrose i knæskal		1	
DM925;Juvenil osteokondrose i underben		1	
DM925A;Osteochondrosis tuberositatis tibiae		1	
DM925B;Osteochondrosis juvenilis fibulae		1	

DM926;Juvenil osteokondrose i fodrod		1	
DM926B;Osteochondrosis juvenilis ossis navicularis pedis		1	
DM926C;Osteochondrosis juvenilis tali		1	
DM926D;Osteochondrosis juvenilis calcanei		1	
DM927;Juvenil osteokondrose i mellemfod		1	
DM927A;Osteochondrosis capituli metatarsi II		1	
DM928;Anden juvenil brusklidelse		1	
DM928A;Apophysitis calcanei		1	
DM929;Juvenil brusklidelse UNS		1	
DM93;Andre osteokondropatier		1	
DM930;Juvenil ikke-traumatisk hofteepifysiolyse		1	
DM931;Osteokondrose i os lunatum hos voksen		1	
DM932;Osteochondritis dissecans		1	
DM932A;Osteochondritis dissecans genus		1	
DM932B;Osteochondritis dissecans cubiti		1	
DM938;Anden osteokondropati		1	
DM939;Osteokondropati UNS		1	
DM94;Andre brusksygdomme		1	
DM940;Tietzes syndrom		1	
DM941;Recidiverende polykondritis		1	
DM942;Kondromalaci		1	
DM943;Kondrolyse		1	
DM948;Anden brusksygdom		1	
DM948A;Ruptura cartilaginis articuli		1	
DM948B;Ruptura cartilaginis articuli genus		1	
DM948D;Ruptura cartilaginis articuli cubiti		1	
DM948E;Degeneratio cartilaginis articuli cubiti		1	
DM948F;Degeneratio cartilaginis articuli genus		1	
DM948G;Degeneratio cartilaginis articuli humeri		1	
DM949;Brusksygdom UNS		1	
DM95;Andre erhvervede deformiteter af muskler og knogler		1	
DM950;Erhvervet deformitet af næsen		1	

Supplement 2 Nielsen et al. Mortality after paediatric emergency calls for patients with and without pre-existing comorbidity (2023)

DM951;Sequelae efter othæmatom		1	
DM952;Anden erhvervet deformitet af hovedet		1	
DM952A;Erhvervet deformitet af kraniet		1	
DM952B;Lejebetinget kraniedeformitet hos spædbarn		1	
DM953;Erhvervet deformitet af halsen		1	
DM954;Erhvervet deformitet af thorax eller ribben		1	
DM954A;Erhvervet deformitet af ribben		1	
DM954B;Erhvervet deformitet af thorax		1	
DM955;Erhvervet deformitet af bækkenet		1	
DM958;Anden erhvervet deformitet af muskler eller knogler		1	
DM959;Erhvervet deformitet af muskler eller knogler UNS		1	
DM96;Sygdomme i knogler og muskler efter kirurgiske og medicinske indgreb IKA		1	
DM960;Pseudoartrose efter fusion eller artrodese		1	
DM960A;Pseudoartrose efter fusion		1	
DM960B;Pseudoartrose efter artrodese		1	
DM961;Postlaminektomi syndrom IKA		1	
DM962;Kyfose forårsaget af bestråling		1	
DM963;Postlaminektomikyfose		1	
DM964;Lordose efter kirurgisk indgreb		1	
DM965;Skoliose forårsaget af bestråling		1	
DM966;Fraktur efter indsættelse af ortopædisk implantat		1	
DM968;Anden forstyrrelse i muskler eller knogler efter kirurgisk eller medicinsk indgreb		1	
DM968A;Sekundær instabilitet efter fjernelse af ledprotese		1	
DM969;Forstyrrelse i muskler eller knogler efter kirurgisk eller medicinsk indgreb UNS		1	
DM99;Biomekaniske dysfunktioner IKA		1	
DM990;Segmentær eller somatisk dysfunktion		1	
DM990A;Somatisk dysfunktion UNS		1	
DM990B;Segmentær dysfunktion UNS		1	
DM991;Subluksation i rygsøjlen		1	
DM992;Subluksationsstenose af rygmarvskanalen		1	
DM993;Ossøs stenose af rygmarvskanalen		1	
DM994;Bløddelsstenose af rygmarvskanalen		1	

DM995;Diskusstenose af rygmarvskanalen		1	
DM996;Stenose af foramen intervertebralis forårsaget af knoglevæv eller subluksation		1	
DM997;Stenose af foramen intervertebralis forårsaget af bløddele eller diskus		1	
DM998;Anden biomekanisk dysfunktion		1	
DM999;Biomekanisk dysfunktion UNS		1	
DN00;Akut nyrebetændelse		1	
DN000;Akut glomerulonefritis med minimale glomerulære forandringer		1	
DN001;Akut glomerulonefritis med fokale eller segmentære glomerulære forandringer		1	
DN002;Akut glomerulonefritis med diffus membranøs morfologi		1	
DN003;Akut glomerulonefritis med diffus mesangial proliferation		1	
DN004;Akut glomerulonefritis med diffus endokapillær proliferation		1	
DN005;Akut glomerulonefritis med membranoproliferativ morfologi type 1 og 3 eller UNS		1	
DN006;Akut glomerulonefritis med membranoproliferativ morfologi type 2		1	
DN007;Akut glomerulonefritis med ekstrakapillær morfologi		1	
DN008;Akut glomerulonefritis med anden morfologi		1	
DN008A;Akut glomerulonefritis med proliferativ morfologi UNS		1	
DN009;Akut glomerulonefritis UNS		1	
DN01;Akut progredierende nyrebetændelse		1	
DN010;Akut progredierende glomerulonefritis med minimale glomerulære forandringer		1	
DN011;Akut progredierende glomerulonefritis med fokale eller segmentære glomerulære forandringer		1	
DN012;Akut progredierende glomerulonefritis med diffus membranøs morfologi		1	
DN013;Akut progredierende glomerulonefritis med diffus mesangial proliferation		1	
DN014;Akut progredierende glomerulonefritis med diffus endokapillær proliferation		1	
DN015;Akut progredierende glomerulonefritis med membranoproliferativ morfologi type 1 og 3 eller UNS		1	
DN016;Akut progredierende glomerulonefritis med membranoproliferativ morfologi type 2		1	
DN017;Akut progredierende glomerulonefritis med ekstrakapillær morfologi		1	
DN018;Akut progredierende glomerulonefritis med anden morfologi		1	
DN018A;Akut progredierende glomerulonefritis med proliferativ morfologi UNS		1	
DN019;Akut progredierende glomerulonefritis UNS		1	
DN02;Tilbagevendende og vedvarende blod i urinen		1	
DN020;Recidiverende eller vedvarende hæmaturi med minimale glomerulære forandringer		1	
DN021;Recidiverende eller vedvarende hæmaturi med fokale eller segmentære glomerulære forandringer		1	

Supplement 2 Nielsen et al. Mortality after paediatric emergency calls for patients with and without pre-existing comorbidity (2023)

DN022;Recidiverende eller vedvarende hæmaturi med diffus membranøs morfologi		1	
DN023;Recidiverende eller vedvarende hæmaturi med diffus mesangial proliferation		1	
DN024;Recidiverende eller vedvarende hæmaturi med diffus endokapillær proliferation		1	
DN025;Recidiverende eller vedvarende hæmaturi med membranoproliferativ morfologi type 1 og 3 eller UNS		1	
DN026;Recidiverende eller vedvarende hæmaturi med membranoproliferativ morfologi type 2		1	
DN027;Recidiverende eller vedvarende hæmaturi med ekstrakapillær morfologi		1	
DN028;Recidiverende eller vedvarende hæmaturi med anden morfologi		1	
DN028C;Recidiverende eller vedvarende hæmaturi med proliferativ morfologi UNS		1	
DN029;Recidiverende eller vedvarende hæmaturi UNS		1	
DN029A;Vedvarende hæmaturi UNS		1	
DN029B;Recidiverende hæmaturi UNS		1	
DN03;Kronisk nyrebetændelse		1	
DN030;Kronisk glomerulonefritis med minimale glomerulære forandringer		1	
DN031;Kronisk glomerulonefritis med fokale eller segmentære glomerulære forandringer		1	
DN032;Kronisk glomerulonefritis med diffus membranøs morfologi		1	
DN033;Kronisk glomerulonefritis med diffus mesangial proliferation		1	
DN034;Kronisk glomerulonefritis med diffus endokapillær proliferation		1	
DN035;Kronisk glomerulonefritis med membranoproliferativ morfologi type 1 og 3 eller UNS		1	
DN036;Kronisk glomerulonefritis med membranoproliferativ morfologi type 2		1	
DN037;Kronisk glomerulonefritis med ekstrakapillær morfologi		1	
DN038;Kronisk glomerulonefritis med anden morfologi		1	
DN038A;Kronisk glomerulonefritis med proliferativ morfologi UNS		1	
DN039;Kronisk glomerulonefritis UNS		1	
DN04;Nefrotisk syndrom		1	
DN040;Nefrose med minimale glomerulære forandringer		1	
DN041;Nefrose med fokale eller segmentære glomerulære forandringer		1	
DN042;Nefrose med diffus membranøs morfologi		1	
DN043;Nefrose med diffus mesangial proliferation		1	
DN044;Nefrose med diffus endokapillær proliferation		1	
DN045;Nefrose med membranoproliferativ morfologi type 1 og 3 eller UNS		1	
DN046;Nefrose med membranoproliferativ morfologi type 2		1	
DN047;Nefrose med ekstrakapillær morfologi		1	

DN048;Nefrose med anden morfologi		1	
DN048A;Nefrose med proliferativ morfologi UNS		1	
DN049;Nefrose UNS		1	
DN05;Nyrebetændelse UNS		1	
DN050;Glomerulonefritis UNS med minimale glomerulære forandringer		1	
DN051;Glomerulonefritis UNS med fokale eller segmentære glomerulære forandringer		1	
DN052;Glomerulonefritis UNS med diffus membranøs morfologi		1	
DN053;Glomerulonefritis UNS med diffus mesangial proliferation		1	
DN054;Glomerulonefritis UNS med diffus endokapillær proliferation		1	
DN055;Glomerulonefritis UNS med membranoproliferativ morfologi type 1 og 3 eller UNS		1	
DN056;Glomerulonefritis UNS med membranoproliferativ morfologi type 2		1	
DN057;Glomerulonefritis UNS med ekstrakapillær morfologi		1	
DN058;Glomerulonefritis UNS med anden morfologi		1	
DN058A;Glomerulonefritis UNS med proliferativ morfologi UNS		1	
DN059;Glomerulonefritis UNS		1	
DN06;Isoleret protein i urinen med forandringer i nyrevævet		1	
DN060;Monosymptomatisk proteinuri med minimale glomerulære forandringer		1	
DN061;Monosymptomatisk proteinuri med fokale eller segmentære glomerulære forandringer		1	
DN062;Monosymptomatisk proteinuri med diffus membranøs morfologi		1	
DN063;Monosymptomatisk proteinuri med diffus mesangial proliferation		1	
DN064;Monosymptomatisk proteinuri med diffus endokapillær proliferation		1	
DN065;Monosymptomatisk proteinuri med membranoproliferativ morfologi type 1 og 3 eller UNS		1	
DN066;Monosymptomatisk proteinuri med membranoproliferativ morfologi type 2		1	
DN067;Monosymptomatisk proteinuri med ekstrakapillær morfologi		1	
DN068;Monosymptomatisk proteinuri med anden morfologi		1	
DN068A;Monosymptomatisk proteinuri med proliferativ morfologi UNS		1	
DN069;Monosymptomatisk proteinuri med forandringer i nyrevævet UNS		1	
DN07;Arvelig nyresygdom IKA		1	
DN070;Arvelig nefropati med minimale glomerulære forandringer		1	
DN071;Arvelig nefropati med fokale eller segmentære glomerulære forandringer		1	
DN072;Arvelig nefropati med diffus membranøs morfologi		1	
DN073;Arvelig nefropati med diffus mesangial proliferation		1	

DN074;Arvelig nefropati med diffus endokapillær proliferation		1	
DN075;Arvelig nefropati med membranoproliferativ morfologi type 1 og 3 eller UNS		1	
DN076;Arvelig nefropati med membranoproliferativ morfologi type 2		1	
DN077;Arvelig nefropati med ekstrakapillær morfologi		1	
DN078;Arvelig nefropati med anden morfologi		1	
DN078A;Arvelig nefropati med proliferativ morfologi UNS		1	
DN079;Arvelig nefropati UNS		1	
DN08;Glomerulære sygdomme ved sygdomme klassificeret andetsteds		1	
DN080;Glomerulonefropati ved infektiøs eller parasitær sygdom klassificeret andetsteds		1	
DN081;Glomerulonefropati ved kræftsygdom klassificeret andetsteds		1	
DN082;Glomerulonefropati ved hæmatologisk eller immunologisk sygdom klassificeret andetsteds		1	
DN083;Glomerulonefropati ved diabetes mellitus		1	
DN084;Glomerulonefropati ved endokrin, ernæringsbetinget eller metabolisk sygdom klassificeret andetsteds		1	
DN085;Glomerulonefropati ved generaliseret bindevævssygdom klassifieret andetsteds		1	
DN088;Glomerulonefropati ved anden sygdom klassificeret andetsteds		1	
DN10;Akut tubulointerstitiel nefritis	0		
DN109;Akut tubulointerstitiel nefritis UNS	0		
DN109A;Akut infektiøs interstitiel nefritis	0		
DN109B;Akut pyelitis	0		
DN109C;Akut pyelonefritis	0		
DN11;Kronisk tubulointerstitiel nefritis		1	
DN110;Ikke-obstruktiv kronisk pyelonefritis ved vesikoureteral refluks		1	
DN110A;Vesikoureteral refluks med kronisk pyelonefritis		1	
DN111;Kronisk obstruktiv pyelonefritis		1	
DN111A;Kronisk pyelonefritis ved ureterovesikal stenose		1	
DN111B;Kronisk pyelonefritis ved ureteropelvin stenose		1	
DN112;Pyelonephritis recidivans (findes ikke i SKS)		1	
DN118;Anden form for kronisk tubulointerstitiel nyresygdom		1	
DN118A;Ikke-obstruktiv kronisk pyelonefritis UNS		1	
DN118B;Recidiverende pyelonefritis		1	
DN118C;Kronisk interstitiel nefritis		1	
DN118D;Kronisk pyelitis		1	

DN119;Kronisk pyelonefritis UNS		1	
DN12;Tubulo-interstitiel nefritis UNS		1	
DN129;Pyelonefritis UNS		1	
DN13;Urinvejslidelser ved afløbshindring og tilbageløb i urinvejene		1	
DN130;Hydronefrose ved ureteropelvin obstruktion		1	
DN131;Hydronefrose ved ureterstriktur IKA		1	
DN131A;Retroperitoneal fibrose med hydronefrose		1	
DN132;Hydronefrose med obstruktion forårsaget af sten i nyrebækken eller ureter		1	
DN132A;Hydronefrose med obstruktion forårsaget af sten i nyrebækken		1	
DN132B;Hydronefrose med obstruktion forårsaget af sten i ureter		1	
DN133;Anden eller ikke nærmere specificeret hydronefrose		1	
DN133A;Hydronefrose UNS		1	
DN134;Hydroureter		1	
DN135;Striktur eller stenose i ureter uden hydronefrose		1	
DN135A;Stenose i ureter uden hydronefrose		1	
DN135B;Striktur i ureter uden hydronefrose		1	
DN136;Pyonefrose		1	
DN136A;Hydronefrose med infektion		1	
DN136B;Hydroureter med infektion		1	
DN136C;Ureterstenose med infektion		1	
DN136D;Ureterstriktur med infektion		1	
DN136E;Obstruktiv nyrelidelse med infektion UNS		1	
DN137;Uropati ved vesikoureteral refluks		1	
DN138;Anden uropati ved obstruktion eller refluks		1	
DN138A;Nefropati ved refluks forårsaget af infravesikal obstruktion		1	
DN139;Nyrelidelse UNS ved obstruktion eller refluks		1	
DN139A;Nyrelidelse UNS ved obstruktion		1	
DN139B;Nyrelidelse UNS ved refluks		1	
DN139C;Obstruktion i urinvejene UNS		1	
DN14;Tubulointerstitielle nyresygdomme forårsaget af lægemidler og tungmetaller		1	
DN140;Nefropati forårsaget af analgetika		1	
DN140A;Nefropati forårsaget af fenacetin		1	

Supplement 2 Nielsen et al. Mortality after paediatric emergency calls for patients with and without pre-existing comorbidity (2023)

DN141;Nefropati forårsaget af anden biologisk substans eller lægemiddel		1	
DN142;Nefropati forårsaget af ikke specificeret biologisk substans eller lægemiddel		1	
DN143;Nefropati forårsaget af tungmetal		1	
DN144;Toksisk nefropati IKA		1	
DN15;Andre tubulointerstitielle nyresygdomme		1	
DN150;Balkan-nefropati		1	
DN151;Renal eller perirenal absces		1	
DN151A;Perirenal absces		1	
DN151B;Renal absces		1	
DN158;Anden tubulointerstitiel nyresygdom		1	
DN159;Tubulointerstitiel nyresygdom UNS		1	
DN16;Tubulointerstitielle nyresygdomme ved sygdomme klassificeret andetsteds		1	
DN160;Nefropati ved infektiøs eller parasitær sygdom klassificeret andetsteds		1	
DN161;Nefropati ved neoplastisk sygdom		1	
DN162;Nefropati ved hæmatologisk eller immunologisk sygdom klassificeret andetsteds		1	
DN163;Nefropati ved metabolisk sygdom		1	
DN164;Nefropati ved systemisk bindevævssygdom		1	
DN165;Nefropati ved afstødning af transplantat		1	
DN168;Nefropati ved anden sygdom klassificeret andetsteds		1	
DN17;Akut nyreinsufficiens		1	
DN170;Akut nyreinsufficiens med tubulær nekrose		1	
DN171;Akut nyreinsufficiens med akut kortikal nekrose		1	
DN172;Akut nyreinsufficiens med medullær nekrose		1	
DN178;Anden form for akut nyreinsufficiens		1	
DN179;Akut nyreinsufficiens UNS		1	
DN18;Kronisk nyreinsufficiens		1	
DN181;Kronisk nyreinsufficiens uden funktionsnedsættelse, stadie 1		1	
DN182;Kronisk nyreinsufficiens, stadie 2		1	
DN183;Kronisk nyreinsufficiens, stadie 3		1	
DN184;Kronisk nyreinsufficiens, stadie 4		1	
DN185;Kronisk nyreinsufficiens, terminal stadie 5			2
DN189;Kronisk nyreinsufficiens UNS		1	

DN19;Nyreinsufficiens UNS		1	
DN199;Nyreinsufficiens UNS		1	
DN20;Sten i nyre og urinleder		1	
DN200;Nyresten UNS		1	
DN200A;Anatomisk nyresten		1	
DN200F;Funktionel nyresten		1	
DN200I;Infektiøs nyresten		1	
DN200M;Metabolisk nyresten		1	
DN200S;Simpel idiopatisk kalciumnefrolitiasis		1	
DN200X;Kompliceret idiopatisk kalciumnefrolitiasis		1	
DN200X1;Kompliceret idiopatisk kalciumnefrolitiasis uden hyperkalcæmi eller hypocitraturi		1	
DN200X2;Kompliceret idiopatisk kalciumnefrolitiasis, døgnanalyse ikke udført		1	
DN200Y;Anden nyresten		1	
DN200Z;Nyresten, der ikke kan klassificeres		1	
DN201;Uretersten UNS		1	
DN201A;Anatomisk uretersten		1	
DN201F;Funktionel uretersten		1	
DN201I;Infektiøs uretersten		1	
DN201M;Metabolisk uretersten		1	
DN201S;Simpel idiopatisk kalciumureterolitiasis		1	
DN201X;Kompliceret idiopatisk kalciumureterolitiasis		1	
DN201X1;Kompliceret idiopatisk kalciumureterolitiasis uden hyperkalcæmi eller hypocitraturi		1	
DN201X2;Kompliceret idiopatisk kalciumureterolitiasis, døgnanalyse ikke udført		1	
DN201Y;Anden uretersten		1	
DN201Z;Uretersten, der ikke kan klassificeres		1	
DN202;Nyresten med uretersten UNS		1	
DN202A;Anatomisk nyresten med uretersten		1	
DN202F;Funktionel nyresten med uretersten		1	
DN202I;Infektiøs nyresten med uretersten		1	
DN202M;Metabolisk nyresten med uretersten		1	
DN202S;Simpel idiopatisk kalciumnefroureterolitiasis		1	
DN202X;Kompliceret idiopatisk kalciumnefroureterolitiasis		1	

Supplement 2 Nielsen et al. Mortality after paediatric emergency calls for patients with and without pre-existing comorbidity (2023)

DN202X1;Kompliceret idiopatisk kalciumnefroureterolitiasis uden hyperkalcæmi eller hypocitraturi		1	
DN202X2;Kompliceret idiopatisk kalciumnefroureterolitiasis, døgnanalyse ikke udført		1	
DN202Y;Anden nyresten med uretersten		1	
DN202Z;Nyresten med uretersten, der ikke kan klassificeres		1	
DN209;Urinvejssten UNS		1	
DN209A;Nyrebækkenbetændelse med nyresten		1	
DN21;Sten i nedre urinveje		1	
DN210;Urinblæresten		1	
DN210A;Sten i urinblæredivertikel		1	
DN211;Uretrasten		1	
DN218;Anden form for sten i nedre urinveje		1	
DN219;Sten i nedre urinveje UNS		1	
DN22;Urinvejssten ved sygdomme klassificeret andetsteds		1	
DN220;Urinvejssten ved skistosomiasis		1	
DN228;Urinvejssten ved anden sygdom klassificeret andetsteds		1	
DN23;Nyrekolik UNS		1	
DN239;Colica renalis UNS		1	
DN25;Sygdomme ved nedsat tubulær nyrefunktion		1	
DN250;Nefrogen osteodystrofi		1	
DN250B;Nefrogen dværgvækst		1	
DN251;Nefrogen diabetes insipidus		1	
DN258;Anden sygdom ved nedsat tubulær nyrefunktion		1	
DN258A;Tubulær renal acidose UNS		1	
DN258B;Sekundær renal hyperparathyroidisme		1	
DN258C;Lightwood-Albrights syndrom		1	
DN259;Nedsat tubulær nyrefunktion UNS		1	
DN26;Skrumpenyrer UNS		1	
DN269;Skrumpenyre UNS		1	
DN27;Små nyrer af ukendt årsag		1	
DN270;Enkeltsidig nyreatrofi		1	
DN271;Dobbeltsidig nyreatrofi		1	
DN279;Nyreatrofi UNS		1	

Supplement 2 Nielsen et al. Mortality after paediatric emergency calls for patients with and without pre-existing comorbidity (2023)

DN28;Andre sygdomme i nyre og urinleder IKA		1	
DN280;Iskæmi eller infarkt i nyre		1	
DN280A;Emboli i nyrearterie		1	
DN280B;Nyreinfarkt		1	
DN280C;Nyreiskæmi		1	
DN280D;Trombose i nyrearterie		1	
DN281;Erhvervet nyrecyste		1	
DN288;Anden sygdom i nyre eller urinleder		1	
DN288A;Hypertrophia renis		1	
DN288B;Megaloureter		1	
DN288C;Nephroptosis		1	
DN288D;Pyelitis cystica		1	
DN288E;Pyeloureteritis cystica		1	
DN288F;Ureteritis cystica		1	
DN288G;Ureterocele		1	
DN288H;Ren mobilis		1	
DN288J;Forkalkninger i nyreparenkymet		1	
DN289;Sygdom i nyre eller urinleder UNS		1	
DN289A;Nefropati UNS		1	
DN29;Andre sygdomme i nyre og urinleder ved sygdomme klassificeret andetsteds		1	
DN290;Sensyfilitisk nyresygdom UNS		1	
DN291;Sygdom i nyre eller urinleder ved infektiøs eller parasitær sygdom klassificeret andetsteds		1	
DN298;Anden sygdom i nyre eller urinleder ved sygdom klassificeret andetsteds		1	
DN30;Blærebetændelse		1	
DN300;Akut blærebetændelse		1	
DN301;Kronisk interstitiel cystitis		1	
DN302;Anden kronisk cystitis		1	
DN303;Trigonitis		1	
DN303A;Urethrotrigonitis		1	
DN304;Cystitis forårsaget af bestråling		1	
DN308;Anden cystitis		1	
DN308A;Empyema vesicae urinariae		1	

DN308B;Hæmoragisk cystitis		1	
DN308C;Cystitis recidivans		1	
DN308D;Eosinofil cystitis		1	
DN308E;Cystitis cystica		1	
DN308F;Cystitis glandularis		1	
DN308G;Cystitis planocellularis		1	
DN308H;Nefrogent adenom		1	
DN308J;Skistosomiasiscystitis		1	
DN308K;Cystitis follicularis		1	
DN309;Cystitis UNS		1	
DN31;Neuromuskulære funktionsforstyrrelser i urinblæren IKA		1	
DN310;Spastisk neurogen urinblære IKA		1	
DN311;Reflektorisk neurogen urinblære IKA		1	
DN312;Atonisk neurogen urinblære IKA		1	
DN318;Anden neuromuskulær funktionsforstyrrelse i urinblæren		1	
DN318A;Detrusor-sphincter dyssynergi		1	
DN319;Neuromuskulær funktionsforstyrrelse i urinblæren UNS		1	
DN32;Andre sygdomme i urinblæren		1	
DN320;Urinblærehalsstenose		1	
DN320A;Urinblærehalssklerose		1	
DN320B;Urinblærehalsdyssynergi		1	
DN321;Vesikointestinal fistel		1	
DN321A;Vesikorektal fistel		1	
DN322;Urinblærefistel IKA		1	
DN323;Urinblæredivertikel		1	
DN324;Ikke-traumatisk urinblæreruptur		1	
DN328;Anden sygdom i urinblæren		1	
DN328A;Calcificatio vesicae urinariae		1	
DN328B;Contractus vesicae urinariae		1	
DN328C;Hypertrophia vesicae urinariae		1	
DN328D;Leukoplakia vesicae urinariae		1	
DN328E;Vesica urinaria irritabilis		1	

DN328F;Dysplasia vesicae urinariae		1	
DN329;Sygdom i urinblæren UNS		1	
DN33;Sygdomme i urinblæren ved sygdomme klassificeret andetsteds		1	
DN330;Tuberkuløs cystitis		1	
DN338;Sygdom i urinblæren ved anden sygdom klassificeret andetsteds		1	
DN34;Urinrørsbetændelse og uretrasyndrom		1	
DN340;Uretralabsces		1	
DN340B;Abscessus periurethralis		1	
DN340C;Carbunculus urethrae		1	
DN341;Infektiøs uretritis UNS		1	
DN342;Anden form for uretritis		1	
DN342A;Meatitis urethrae		1	
DN342B;Periuretritis		1	
DN342C;Ulcus urethrae		1	
DN342D;Allergisk uretritis		1	
DN342E;Postmenopausal uretritis		1	
DN342F;Urethritis recidivans		1	
DN342G;Uretritis UNS		1	
DN343;Uretralt syndrom UNS		1	
DN35;Urinrørsforsnævring		1	
DN350;Posttraumatisk uretrastriktur		1	
DN350A;Posttraumatisk uretrastriktur efter fødsel		1	
DN351;Postinfektiøs uretrastriktur IKA		1	
DN358;Anden form for uretrastriktur		1	
DN358A;Stenosis meatus urethrae		1	
DN359;Uretrastriktur UNS		1	
DN36;Andre sygdomme i urinrøret		1	
DN360;Uretrafistel		1	
DN360A;Periuretral fistel		1	
DN360B;Uretrorektal fistel		1	
DN360C;Uretroperineal fistel		1	
DN361;Uretradivertikel		1	

DN362;Caruncula urethrae		1	
DN363;Uretralprolaps		1	
DN363A;Uretrocele hos mand		1	
DN368;Anden sygdom i uretra		1	
DN368A;Haemorrhagia urethrae		1	
DN368B;Leukoplakia urethrae		1	
DN368D;Via falsa paraprostatica		1	
DN368E;Via falsa paraurethralis		1	
DN369;Sygdom i uretra UNS		1	
DN37;Sygdomme i urinrøret ved sygdom klassificeret andetsteds		1	
DN370;Uretritis ved sygdom klassificeret andetsteds		1	
DN378;Anden sygdom i urethra ved sygdom klassificeret andetsteds		1	
DN39;Andre sygdomme i urinvejene		1	
DN390;Urinvejsinfektion uden angivelse af lokalisation		1	
DN390A;Asymptomatisk bakteriuri		1	
DN390B;Pyuria		1	
DN391;Vedvarende proteinuri UNS		1	
DN392;Ortostatisk proteinuri UNS		1	
DN393;Stressinkontinens		1	
DN394;Anden form for urininkontinens		1	
DN394A;Overløbsurininkontinens		1	
DN394B;Refleksurininkontinens		1	
DN394C;Urge-urininkontinens		1	
DN398;Anden sygdom i urinvejene		1	
DN398A;Forandringer i urinvejene UNS forårsaget af bestråling		1	
DN399;Sygdom i urinvejene UNS		1	
DN399A;Funktionsforstyrrelse i urinvejene UNS		1	
DN40;Forstørret blærehalskirtel		1	
DN409;Prostatahypertrofi		1	
DN409B;Cyste i prostata		1	
DN409E;Prostatafibrose		1	
DN409G;Adenomatøs hyperplasi i prostata		1	

Supplement 2 Nielsen et al. Mortality after paediatric emergency calls for patients with and without pre-existing comorbidity (2023)

DN41;Betændelse i blærehalskirtlen		1	
DN410;Akut prostatistis		1	
DN411;Kronisk prostatitis		1	
DN412;Prostataabsces		1	
DN412A;Periprostatisk absces		1	
DN413;Prostatocystitis		1	
DN413A;Prostatovesiculitis		1	
DN418;Anden form for prostatitis		1	
DN419;Prostatitis UNS		1	
DN42;Andre sygdomme i blærehalskirtlen		1	
DN420;Prostatasten		1	
DN421;Prostatablødning		1	
DN422;Prostataatrofi		1	
DN423;Dysplasi i prostata		1	
DN428;Anden sygdom i prostata		1	
DN428A;Prostatafistel		1	
DN429;Sygdom i prostata UNS		1	
DN43;Vandbrok og sædbrok		1	
DN430;Cystisk hydrocele		1	
DN431;Inficeret hydrocele		1	
DN432;Anden form for hydrocele		1	
DN433;Hydrocele UNS		1	
DN434;Spermatocele		1	
DN44;Torsio testis		1	
DN449;Testistorsion UNS		1	
DN449A;Torsio epididymidis		1	
DN449B;Torsio funiculi spermatici		1	
DN449C;Testistorsion med gangræn		1	
DN449D;Torsio appendicis testis		1	
DN449E;Torsio appendicis epididymis		1	
DN45;Betændelse i testikel og bitestikel		1	
DN450;Orkitis, epididymitis eller epididymoorkitis med absces		1	

Supplement 2 Nielsen et al. Mortality after paediatric emergency calls for patients with and without pre-existing comorbidity (2023)

DN450A;Absces i bitestikel		1	
DN450B;Testikelabsces		1	
DN450C;Epididymoorkitis med absces		1	
DN459;Orkitis, epididymitis eller epididymoorkitis uden absces		1	
DN459A;Akut epididymitis UNS		1	
DN459B;Kronisk epididymitis UNS		1	
DN459C;Epididymoorkitis UNS		1	
DN459D;Akut orkitis UNS		1	
DN459E;Recidiverende orkitis UNS		1	
DN46;Mandlig sterilitet		1	
DN469;Mandlig sterilitet UNS		1	
DN469A;Aspermi		1	
DN469B;Azoospermi		1	
DN469C;Oligospermi		1	
DN469D;Oligo-teratozoospermi		1	
DN469E;Mandlig sterilitet efter sterilisation	0		
DN469W;Mandlig sterilitet af anden årsag		1	
DN47;Sygdomme i forhuden	0		
DN479;Sygdomme i forhuden UNS	0		
DN479A;Agglutinatio preputii	0		
DN479B;Frenulum breve preputii	0		
DN479C;Paraphimosis	0		
DN479D;Phimosis	0		
DN479E;Preputium redundans	0		
DN48;Andre sygdomme i penis	0		
DN480;Leukoplaki på penis	0		
DN480A;Erytroplaki på penis	0		
DN480B;Kraurosis penis	0		
DN481;Balanoposthitis	0		
DN481A;Balanitis UNS	0		
DN481B;Balanitis erosiva circinata	0		
DN481C;Gangrænøs balanitis	0		

Supplement 2 Nielsen et al. Mortality after paediatric emergency calls for patients with and without pre-existing comorbidity (2023)

DN481D;Plasmacellebalanitis	0		
DN482;Anden betændelsestilstand i penis	0		
DN482A;Abscessus corporis cavernosi penis	0		
DN482B;Abscessus pelvis viri	0		
DN482C;Abscessus penis	0		
DN482D;Carbunculus corporis cavernosi penis	0		
DN482E;Carbunculus penis	0		
DN482F;Cavernitis penis	0		
DN482G;Furunculus penis	0		
DN482H;Phlegmone corporis cavernosi penis	0		
DN482I;Phlegmone penis	0		
DN483;Priapismus	0		
DN484;Organisk impotens	0		
DN485;Ulcus på penis	0		
DN485A;Ulcus corporis cavernosi penis	0		
DN485B;Ulcus cutis penis chronicum	0		
DN486;Induratio penis plastica	0		
DN486A;Balanitis xerotica obliterans	0		
DN488;Anden sygdom i penis	0		
DN488A;Atrophia corporis cavernosi penis	0		
DN488B;Atrophia penis	0		
DN488C;Cystis penis	0		
DN488D;Haematoma corporis cavernosi penis	0		
DN488E;Haematoma penis	0		
DN488F;Hypertrophia corporis cavernosi penis	0		
DN488G;Thrombosis corporis cavernosi penis	0		
DN488H;Penis arcuatus aquisitus	0		
DN488J;Pearly penile papules	0		
DN488K;Fistel i preputium	0		
DN489;Sygdom i penis UNS	0		
DN49;Andre betændelsestilstande i de mandlige kønsorganer IKA		1	
DN490;Vesiculitis seminalis		1	

DN491;Betændelsestilstand i funiculus spermaticus, tunica vaginalis eller ductus deferens		1	
DN491A;Infectio ductus deferentis		1	
DN491B;Infectio tunicae vaginalis		1	
DN491C;Inflammatio ductus spermatici		1	
DN491D;Inflammatio tunicae vaginalis		1	
DN492;Betændelsestilstand i scrotum		1	
DN492A;Abscessus scroti		1	
DN492B;Carbunculus scroti		1	
DN492C;Furunculus scroti		1	
DN492D;Infectio scroti		1	
DN498;Betændelsestilstand i anden del af de mandlige kønsorganer		1	
DN498A;Infectio multiplex genitalium viri		1	
DN498B;Inflammatio multiplex genitalium viri		1	
DN498C;Fourniers gangræn		1	
DN498M;Mikrolithiasis testis		1	
DN499;Betændelsestilstand i de mandlige kønsorganer UNS		1	
DN50;Andre sygdomme i de mandlige kønsorganer		1	
DN500;Testisatrofi		1	
DN501;Vaskulær sygdom i de mandlige kønsorganer		1	
DN501A;Haematocele testis		1	
DN501B;Haematoma funiculi spermatici		1	
DN501C;Haematoma scroti		1	
DN501D;Haematoma testis		1	
DN501E;Haematoma tunicae vaginalis		1	
DN501F;Thrombosis genitalium viri		1	
DN502;Ejakulatorisk dysfunktion		1	
DN502A;Retrograd ejakulation		1	
DN502B;Ejaculatio tarda		1	
DN502C;Anejakulation		1	
DN508;Anden sygdom i de mandlige kønsorganer		1	
DN508A;Adhaesiones epididymidis		1	
DN508B;Atrophia funiculi spermatici		1	

DN508C;Atrophia scroti		1	
DN508D;Atrophia vesiculae seminalis		1	
DN508E;Chylocele tunicae vaginalis UNS		1	
DN508F;Cystis epididymidis		1	
DN508G;Degeneratio testis postinfectiosa		1	
DN508H;Fibrosis testis		1	
DN508I;Fistula epididymidis		1	
DN508J;Fistula scroti		1	
DN508K;Fistula urethroscrotalis		1	
DN508L;Hypertrophia funiculi spermatici		1	
DN508M;Hypertrophia scroti		1	
DN508N;Hypertrophia testis		1	
DN508O;Hypertrophia vesiculae seminalis		1	
DN508P;Keratosis genitalium viri		1	
DN508Q;Oedema scroti		1	
DN508R;Spermatorrhoea		1	
DN508S;Strictura funiculi spermatici		1	
DN508T;Strictura tunicae vaginalis		1	
DN508U;Strictura vasis deferentis		1	
DN508V;Ulcus scroti		1	
DN508X;Hæmospermi		1	
DN508Y;Testodynia		1	
DN509;Sygdom i de mandlige kønsorganer UNS		1	
DN51;Sygdomme i mandlige kønsorganer ved sygdomme klassificeret andetsteds		1	
DN510;Sygdom i prostata ved sygdom klassificeret andetsteds		1	
DN511;Sygdom i testikel eller epididymis ved sygdom klassificeret andetsteds		1	
DN511K;Epididymitis ved sygdom klassificeret andetsteds		1	
DN511L;Orkitis ved sygdom klassificeret andetsteds		1	
DN512;Balanitis ved sygdom klassificeret andetsteds		1	
DN518;Anden sygdom i de mandlige kønsorganer ved sygdom klassificeret andetsteds		1	
DN60;Godartet dysplasi i brystkirtel		1	
DN600;Solitær mammacyste		1	

DN600A;Cyste i brystvorte		1	
DN601;Multiple mammacyster		1	
DN602;Fibroadenomatose i brystkirtel		1	
DN603;Fibrosklerose i brystkirtel		1	
DN603A;Atypisk duktal hyperplasi		1	
DN603B;Atypisk lobulær hyperplasi		1	
DN604;Ectasia ductus mammae		1	
DN608;Anden form for godartet mammadysplasi		1	
DN608A;Mikroforkalkninger i mamma		1	
DN609;Godartet mammadysplasi UNS		1	
DN61;Ikke-puerperal betændelse i brystkirtel		1	
DN619;Ikke-puerperal betændelse i mamma UNS		1	
DN619A;Ikke-puerperal absces i areola mammae		1	
DN619B;Ikke-puerperal mammaabsces		1	
DN619E;Ikke-puerperal infektiøs mastitis		1	
DN62;Store bryster		1	
DN629;Mammahypertrofi		1	
DN629A;Gynækomasti		1	
DN629B;Hypertrophia areolae mammae		1	
DN629D;Dobbeltsidig mammahypertrofi		1	
DN629E;Enkeltsidig mammahypertrofi		1	
DN63;Knude i bryst UNS		1	
DN639;Mammatumor UNS		1	
DN64;Andre sygdomme i brystkirtel		1	
DN640;Fissur eller fistel i brystvorte		1	
DN640A;Fissur i brystvorte		1	
DN640B;Fistel i brystvorte		1	
DN641;Liponekrose i mamma		1	
DN642;Mammaatrofi		1	
DN643;Ikke-puerpural galaktorré		1	
DN644;Smerter i mamma		1	
DN644A;Cykliske mammasmerter		1	

Supplement 2 Nielsen et al. Mortality after paediatric emergency calls for patients with and without pre-existing comorbidity (2023)

DN644B;Ikke-cykliske mammasmerter		1	
DN645;Andet symptom eller fund i mamma		1	
DN645A;Fluor papillae mammae		1	
DN645B;Induratio mammae		1	
DN645C;Inversio papillae mammae		1	
DN645D;Papilla mammae retracta		1	
DN648;Anden sygdom i mamma		1	
DN648A;Deformatio mammae		1	
DN648D;Galaktocele		1	
DN648E;Subinvolutio mammae		1	
DN648F;Ulcus mammae		1	
DN648G;Fremmedlegeme i mamma		1	
DN648H;Ptosis mammae		1	
DN649;Sygdom i mamma UNS		1	
DN70;Betændelse i æggeleder og æggestok		1	
DN700;Akut salpingitis eller ooforitis		1	
DN700A;Akut ovarieabsces		1	
DN700B;Akut æggelederabsces		1	
DN700C;Akut ooforitis		1	
DN700D;Pyosalpinx acuta		1	
DN700E;Pyovarium acutum		1	
DN700F;Akut salpingitis		1	
DN700G;Akut salpingooophoritis		1	
DN701;Kronisk salpingitis eller ooforitis		1	
DN701A;Kronisk ovarieabsces		1	
DN701B;Kronisk æggelederabsces		1	
DN701C;Hydrosalpinx		1	
DN701D;Kronisk ooforitis		1	
DN701E;Pyosalpinx chronica		1	
DN701F;Pyovarium chronicum		1	
DN701G;Kronisk salpingitis		1	
DN701H;Kronisk salpingooophoritis		1	

DN709;Salpingitis eller ooforitis UNS		1		
DN709A;Ooforitis UNS		1		
DN709B;Salpingitis UNS		1		
DN709C;Salpingooooforitis UNS		1		
DN71;Betændelsestilstande i livmoderen, undtagen livmoderhalsen		1		
DN710;Akut endometritis		1		
DN710A;Akut absces i livmoderen		1		
DN710C;Akut purulent endometritis		1		
DN710D;Akut endomyometritis		1		
DN710E;Akut myometritis		1		
DN710F;Akut pyometra		1		
DN711;Kronisk endometritis		1		
DN711A;Kronisk absces i livmoderen		1		
DN711B;Kronisk purulent endometritis		1		
DN711D;Kronisk atrofisk endometritis		1		
DN711E;Kronisk myometritis		1		
DN711F;Kronisk pyometra		1		
DN719;Endometritis UNS		1		
DN72;Betændelsestilstande i livmoderhalsen		1		
DN729;Cervicitis uteri UNS		1		
DN729A;Cervicitis uteri acuta		1		
DN729B;Cervicitis uteri chronica		1		
DN729C;Endocervicitis UNS		1		
DN729D;Endocervicitis acuta		1		
DN729E;Endocervicitis chronica		1		
DN729F;Endometritis cervicis uteri		1		
DN73;Anden form for underlivsbetændelse		1		
DN730;Akut parametritis eller flegmone i det kvindelige bækken		1		
DN730A;Akut absces i ligamentum latum		1		
DN730B;Akut absces i parametriet		1		
DN730C;Akut parametritis		1		
DN730D;Akut flegmone i ligamentum latum		1		

DN730E;Akut flegmone i det kvindelige bækken		1	
DN731;Kronisk parametritis eller flegmone i det kvindelige bækken		1	
DN731A;Kronisk absces i ligamentum latum		1	
DN731B;Kronisk absces i parametriet		1	
DN731C;Kronisk parametritis		1	
DN731D;Kronisk flegmone i ligamentum latum		1	
DN731E;Kronisk flegmone i det kvindelige bækken		1	
DN732;Parametritis eller flegmone i det kvindelige bækken UNS		1	
DN732A;Absces UNS i ligamentum latum		1	
DN732B;Absces UNS i parametriet		1	
DN732C;Parametritis UNS		1	
DN732D;Flegmone UNS i ligamentum latum		1	
DN732E;Flegmone UNS i det kvindelige bækken		1	
DN733;Akut kvindelig pelveoperitonitis		1	
DN733A;Akut absces i fossa Douglasi		1	
DN734;Kronisk kvindelig pelveoperitonitis		1	
DN734A;Kronisk absces i fossa Douglasi		1	
DN735;Kvindelig pelveoperitonitis UNS		1	
DN735A;Absces i fossa Douglasi UNS		1	
DN736;Adhærencer i det kvindelige bækken		1	
DN736A;Ovarieadhærencer		1	
DN736B;Tubo-ovariale adhærencer		1	
DN738;Anden infektion i det kvindelige bækken		1	
DN738A;Akut absces i det kvindelige bækken		1	
DN738B;Kronisk absces i det kvindelige bækken		1	
DN738C;Absces i det kvindelige bækken UNS		1	
DN739;Infektion i det kvindelige bækken UNS		1	
DN74;Underlivsbetændelse ved sygdom klassificeret andetsteds		1	
DN740;Tuberkuløs cervicit		1	
DN741;Tuberkulose i kvindelige kønsorganer IKA		1	
DN741A;Tubekuløs endometritis		1	
DN742;Syfilitisk underlivsbetændelse		1	

DN743;Gonoroisk underlivsbetændelse		1	
DN744;Underlivsbetændelse forårsaget af klamydia		1	
DN748;Kvindelig pelveoperitonitis ved sygdom klassificeret andetsteds		1	
DN75;Sygdom i Bartholins kirtler		1	
DN750;Cyste i Bartholins kirtel		1	
DN751;Absces i Bartholins kirtel		1	
DN758;Anden sygdom i Bartholins kirtler		1	
DN758A;Akut bartholinitis		1	
DN758B;Kronisk bartholinitis		1	
DN758C;Bartholinitis UNS		1	
DN759;Sygdom i Bartholins kirtler UNS		1	
DN76;Andre betændelsessygdomme i skede og ydre kvindelige kønsdele		1	
DN760;Akut vaginitis		1	
DN760B;Pyokolpos		1	
DN760C;Vaginitis UNS		1	
DN760D;Akut vulvovaginitis		1	
DN760E;Vulvovaginitis UNS		1	
DN761;Subakut eller kronisk vaginitis		1	
DN761A;Kronisk vaginitis		1	
DN761B;Subakut vaginitis		1	
DN761C;Kronisk vulvovaginitis		1	
DN761D;Subakut vulvovaginitis		1	
DN761E;Plasmacellevulvit		1	
DN762;Akut vulvitis		1	
DN762A;Vulvitis UNS		1	
DN763;Subakut eller kronisk vulvitis		1	
DN763A;Kronisk vulvitis		1	
DN763B;Subakut vulvitis		1	
DN764;Absces i vulva		1	
DN764A;Abscessus labii majoris		1	
DN764B;Abscessus labii minoris		1	
DN764C;Furunculus labii majoris		1	

DN764D;Furunculus labii minoris		1	
DN764E;Furunculus vulvae		1	
DN765;Ulcus i vagina		1	
DN766;Ulcus i vulva		1	
DN766A;Noma vulvae		1	
DN768;Anden betændelsessygdom i vagina eller vulva		1	
DN768A;Vaginal absces		1	
DN768B;Bakteriel vaginitis UNS		1	
DN768C;Bakteriel vaginose UNS		1	
DN77;Sår og betændelse i skede og ydre kvindelige kønsdele ved sygdomme klassificeret andetsteds		1	
DN770;Ulcus i vulva ved infektiøs eller parasitær sygdom klassificeret andetsteds		1	
DN771;Vaginitis, vulvitis eller vulvovaginitis ved infektiøs eller parasitær sygdom klassificeret andetsteds		1	
DN771P;Vaginitis ved infektiøs eller parasitær sygdom klassificeret andetsteds		1	
DN771Q;Vulvitis ved infektiøs eller parasitær sygdom klassificeret andetsteds		1	
DN771R;Vulvovaginitis ved infektiøs eller parasitær sygdom klassificeret andetsteds		1	
DN778;Vulvovaginalt ulcus eller inflammation ved anden sygdom klassificeret andetsteds		1	
DN80;Endometriose		1	
DN800;Endometriose i livmoderen		1	
DN801;Endometriose i ovarie		1	
DN802;Endometriose i æggeleder		1	
DN803;Endometriose i peritoneum		1	
DN804;Endometriose i septum rectovaginalis eller vagina		1	
DN804A;Endometriose i septum rectovaginalis		1	
DN804B;Endometriose i vagina		1	
DN805;Endometriose i mave-tarm-kanalen		1	
DN805A;Endometriose i tyktarmen		1	
DN805B;Endometriose i tyndtarmen		1	
DN805C;Endometriosis recto-sigmoidalis		1	
DN806;Endometriose i arvæv i huden		1	
DN808;Anden form for endometriose		1	
DN808A;Endometriose i flere lokalisationer		1	
DN808B;Endometriose i parametriet		1	

DN808C;Endometriosis umbilicalis		1	
DN808D;Endometriose i urinblæren		1	
DN809;Endometriose UNS		1	
DN81;Nedsynkning og fremfald af kvindelige kønsdele		1	
DN810;Uretrocele hos kvinde		1	
DN811;Cystocele hos kvinde		1	
DN812;Livmoderprolaps uden vaginalprolaps		1	
DN813;Inkomplet uterovaginal prolaps		1	
DN813A;Prolaps af livmoderhalsen		1	
DN814;Komplet uterovaginal prolaps		1	
DN815;Uterovaginal prolaps UNS		1	
DN815A;Livmoderprolaps UNS		1	
DN816;Vaginalt enterocele		1	
DN816A;Hernia vaginalis		1	
DN817;Rektocele		1	
DN818;Anden form for kvindelig genitalprolaps		1	
DN818A;Relaxatio perinei		1	
DN818B;Relaxatio vaginae		1	
DN818C;Ruptura perinei vetus		1	
DN818D;Lateraldefekt i vagina		1	
DN819;Kvindelig genitalprolaps UNS		1	
DN82;Fistler i kvindelige kønsorganer		1	
DN820;Vesikovaginal fistel		1	
DN821;Anden fistel mellem urinveje og kvindelige kønsorganer		1	
DN821A;Cervikovesikal fistel		1	
DN821B;Ureterovaginal fistel		1	
DN821C;Uretrovaginal fistel		1	
DN821D;Uterouereterisk fistel		1	
DN821E;Uterovesikal fistel		1	
DN822;Intestinovaginal fistel		1	
DN823;Kolovaginal fistel		1	
DN823A;Rektovaginal fistel		1	

DN824;Anden fistel mellem kvindelige kønsorganer og tarm		1	
DN824A;Intestinouterin fistel		1	
DN824B;Rektouterin fistel		1	
DN825;Fistel mellem kvindelige kønsorganer og hud		1	
DN825A;Fistula cutaneo-genitalis feminae		1	
DN825B;Fistel fra Bartholins kirtel		1	
DN825C;Fistula labii majoris vulvae		1	
DN825D;Fistula labii minoris vulvae		1	
DN825E;Uteroabdominal fistel		1	
DN825F;Vaginoperineal fistel		1	
DN828;Anden fistel i kvindelige kønsorganer		1	
DN828A;Fistula cervicis uteri UNS		1	
DN828B;Fistula fossae Douglasi		1	
DN828C;Fistula tubae uterinae		1	
DN828D;Fistula uteri UNS		1	
DN828E;Fistula uterovaginalis		1	
DN828F;Fistula vaginae UNS		1	
DN829;Fistel i kvindelige kønsorganer UNS		1	
DN83;Ikke-inflammatoriske sygdomme i æggestokke, æggeledere og parametrie		1	
DN830;Follikulær ovariecyste		1	
DN830A;Blødning i follikulær ovariecyste		1	
DN831;Corpus luteum-cyste		1	
DN831A;Corpus luteum-hæmatom		1	
DN831B;Blødning i corpus luteum-cyste		1	
DN832;Anden eller ikke specificeret ovariecyste		1	
DN832A;Cystis corporis albicantis		1	
DN832B;Retentionscyste i ovarie		1	
DN832C;Simpel ovariecyste		1	
DN832D;Cystisk ovariedegeneration		1	
DN832X;Ovariecyste UNS		1	
DN833;Erhvervet atrofi af ovarie eller æggeleder		1	
DN833A;Erhvervet atrofi af ovarie		1	

DN833B;Erhvervet atrofi af æggeleder		1	
DN834;Prolaps eller hernie af ovarie eller æggeleder		1	
DN834A;Ovariehernie		1	
DN834B;Ovarieprolaps		1	
DN834C;Æggelederprolaps		1	
DN835;Torsion af ovarie, æggeleder eller uterine adnekser		1	
DN835A;Torsion af uterine adnekser		1	
DN835B;Torsion af ovariecyste		1	
DN835C;Torsio cystis retentionis ovarii		1	
DN835D;Ovarietorsion		1	
DN835E;Torsio tubae uterinae		1	
DN836;Hæmatosalpinx		1	
DN837;Hæmatom i parametriet		1	
DN838;Anden ikke-inflammatorisk sygdom i ovarie, æggeleder og parametrie		1	
DN838A;Atresi af æggeleder		1	
DN838B;Anovulatorisk cyklus		1	
DN838C;Ovariedegeneration		1	
DN838D;Retrouterint hæmatocele		1	
DN838E;Ovariehæmatom		1	
DN838F;Ovarieblødning		1	
DN838G;Hydrocele i ligamentum teres		1	
DN838H;Allen-Masters syndrom		1	
DN838I;Tillukning af æggeleder		1	
DN838J;Ruptur af corpus luteum		1	
DN838K;Ruptur af æggeleder		1	
DN839;Ikke-inflammatorisk sygdom i ovarie, æggeleder eller parametrie UNS		1	
DN84;Polypper i kvindelige kønsorganer		1	
DN840;Polyp i corpus uteri		1	
DN840A;Endometriepolyp i livmoderen		1	
DN840B;Polyp i livmoderen UNS		1	
DN841;Polyp i livmoderhalsen		1	
DN841A;Mukosapolyp i livmoderhalsen		1	

DN842;Vaginal polyp		1	
DN843;Polyp i vulva		1	
DN843A;Polypus labii majoris		1	
DN843B;Polypus labii minoris		1	
DN848;Polyp i anden del af de kvindelige kønsorganer		1	
DN849;Polyp i kvindelige kønsorganer UNS		1	
DN85;Andre ikke-inflammatoriske sygdomme i livmoderen		1	
DN850;Endometriehyperplasi uden atypi		1	
DN850A;Simpel endometriehyperplasi uden atypi		1	
DN850B;Kompleks endometriehyperplasi uden atypi		1	
DN851;Adenomatøs endometriehyperplasi		1	
DN852;Uterushypertrofi		1	
DN852A;Myometriehypertrofi		1	
DN853;Subinvolutio uteri		1	
DN854;Malpositio uteri		1	
DN854A;Anteflexio uteri		1	
DN854B;Lateroflexio uteri		1	
DN854C;Retroversio uteri		1	
DN855;Inversio uteri		1	
DN856;Sammenvoksninger i livmoderen		1	
DN857;Hæmatometra		1	
DN857A;Hæmatosalpinx med hæmatometra		1	
DN858;Anden ikke-inflammatorisk sygdom i livmoderen		1	
DN858A;Erhvervet atresi af æggeleder		1	
DN858B;Erhvervet atresi af livmoder		1	
DN858C;Endometrieatrofi		1	
DN858E;Myometrieatrofi		1	
DN858F;Erhvervet atrofi af livmoderen		1	
DN858G;Uterusdegeneration		1	
DN858H;Endometritis hyperplastica		1	
DN858I;Uterusfibrose		1	
DN858J;Hydrometra		1	

Supplement 2 Nielsen et al. Mortality after paediatric emergency calls for patients with and without pre-existing comorbidity (2023)

DN858K;Incarceratio uteri		1		
DN858L;Leukoplakia uteri		1		
DN858M;Metropathia haemorrhagica		1		
DN858N;Mucometra		1		
DN858O;Ulcus uteri		1		
DN859;Ikke-inflammatorisk sygdom i livmoderen UNS		1		
DN86;Sår og ektropi på livmoderhalsen		1		
DN869;Sår eller ektropi på livmoderhalsen UNS		1		
DN869A;Ektropi på livmoderhalsen		1		
DN869B;Erosion på livmoderhalsen		1		
DN869C;Ulcus på livmoderhalsen		1		
DN869D;Kronisk ulcus på livmoderhalsen		1		
DN869E;Erytroplaki på portio		1		
DN87;Celleforandringer i slimhinde på livmoderhalsen		1		
DN870;Let dysplasi på livmoderhalsen		1		
DN871;Moderat dysplasi på livmoderhalsen		1		
DN872;Svær dysplasi på livmoderhalsen		1		
DN879;Dysplasi på livmoderhalsen UNS		1		
DN88;Andre ikke-inflammatoriske sygdomme i livmoderhalsen		1		
DN880;Leukoplaki på livmoderhalsen		1		
DN881;Følger efter livmoderhalsruptur		1		
DN881A;Adhærencer i livmoderhalsen		1		
DN881B;Cervikovaginale adhærencer		1		
DN881C;Ardannelse i livmoderhalsen		1		
DN882;Striktur eller stenose i livmoderhalsen		1		
DN882A;Stenose i livmoderhalsen		1		
DN882B;Striktur i livmoderhalsen		1		
DN883;Insufficiens af cervix uteri hos ikke-gravid kvinde		1		
DN884;Hypertrofisk forlængelse af livmoderhalsen		1		
DN888;Anden ikke-inflammatorisk sygdom i livmoderhalsen		1		
DN888A;Livmoderhalsatrofi		1		
DN888B;Endometrieatrofi i livmoderhalsen		1		

DN888C;Deformering af livmoderhalsen		1	
DN888D;Degeneration af livmoderhalsen		1	
DN888E;Hyperplastisk endocervicitis		1	
DN888F;Hyperkeratose på livmoderhalsen		1	
DN888G;Endometriehypertrofi i livmoderhalsen		1	
DN888H;Hypertrophia glandularis cystica cervicis		1	
DN889;Ikke-inflammatorisk sygdom i livmoderhalsen UNS		1	
DN89;Andre ikke-inflammatoriske sygdomme i vagina		1	
DN890;Let dysplasi i vagina		1	
DN891;Moderat dysplasi i vagina		1	
DN892;Svær dysplasi i vagina		1	
DN893;Dysplasi i vagina UNS		1	
DN894;Leukoplaki i vagina		1	
DN895;Striktur eller atresi af vagina		1	
DN895A;Adhærencer i vagina		1	
DN895B;Vaginal atresi		1	
DN895C;Vaginal stenose		1	
DN895D;Vaginal striktur		1	
DN895E;Snæver vagina		1	
DN896;Hymen rigidum		1	
DN896A;Snæver hymen		1	
DN896B;Snæver vaginalåbning		1	
DN897;Hæmatokolpos		1	
DN897A;Hæmatokolpos med hæmatometra		1	
DN897B;Hæmatokolpos med hæmatosalpinx		1	
DN898;Anden ikke-inflammatorisk sygdom i vagina		1	
DN898A;Agglutinatio hymenis		1	
DN898B;Vaginal atrofi		1	
DN898C;Caruncula vaginae		1	
DN898D;Ardannelse i vagina		1	
DN898E;Fluor vaginalis UNS		1	
DN898F;Vaginal hypertrofi		1	

DN898G;Kraurosis vaginae		1	
DN898H;Sår i vagina forårsaget af pessar		1	
DN898J;Vaginalcyste		1	
DN899;Ikke-inflammatorisk sygdom i vagina UNS		1	
DN90;Andre ikke-inflammatoriske sygdomme i kvindens ydre kønsdele		1	
DN900;Let dysplasi i vulva		1	
DN900A;LSIL (Low grade Squamous Intraepithelial Lesion) i vulva		1	
DN901;Moderat dysplasi i vulva		1	
DN902;Svær dysplasi i vulva		1	
DN902A;HSIL (High grade Squamous Intraepithelial Lesion) i vulva		1	
DN902B;Vulvær intraepitelial neoplasi, differentieret (dVIN)		1	
DN903;Dysplasi i vulva UNS		1	
DN904;Leukoplaki i vulva		1	
DN904A;Kraurosis vulvae		1	
DN905;Vulvaatrofi		1	
DN905A;Vulvastenose		1	
DN906;Vulvahypertrofi		1	
DN907;Cyste i vulva		1	
DN908;Anden ikke-inflammatorisk sygdom i vulva eller perineum		1	
DN908A;Adhærencer i vulva		1	
DN908B;Caruncula labii majoris		1	
DN908C;Caruncula labii minoris		1	
DN908D;Hæmatom i vulva		1	
DN908E;Kvindeligt hydrocele		1	
DN908F;Hyperkeratose i vulva		1	
DN908G;Klitorishypertrofi		1	
DN909;Ikke-inflammatorisk sygdom i vulva eller perineum UNS		1	
DN909A;Ikke-inflammatorisk sygdom i perineum UNS		1	
DN909B;Ikke-inflammatorisk sygdom i vulva UNS		1	
DN91;Manglende, sparsom og sjælden menstruation		1	
DN910;Primær amenoré		1	
DN911;Sekundær amenoré		1	

DN912;Amenoré UNS		1	
DN913;Primær oligomenoré		1	
DN913A;Primær hypomenoré		1	
DN913C;Primær oligohypomenoré		1	
DN914;Sekundær oligomenoré		1	
DN914A;Sekundær hypomenoré		1	
DN914C;Sekundær oligohypomenoré		1	
DN915;Oligomenoré UNS		1	
DN915A;Hypomenoré UNS		1	
DN915C;Oligohypomenoré UNS		1	
DN92;Kraftig, hyppig og uregelmæssig menstruation		1	
DN920;Menoragi eller polymenoré		1	
DN920A;Hypermenoré		1	
DN920B;Menoragi		1	
DN920C;Polymenoré		1	
DN921;Metroragi		1	
DN921A;Haemorrhagia intermenstrualis		1	
DN921B;Menometroragi		1	
DN922;Juvenil menoragi		1	
DN922A;Juvenil metroragi		1	
DN923;Ovulationsblødning		1	
DN924;Præmenopausal menoragi eller metroragi		1	
DN924A;Menopausal metroragi		1	
DN924B;Præmenopausal metroragi		1	
DN925;Anden uregelmæssig menstruation		1	
DN925A;Anovulatorisk metroragi		1	
DN925B;Metroragi forårsaget af hormonbehandling		1	
DN926;Uregelmæssig menstruation UNS		1	
DN93;Anden abnorm blødning fra livmoderen og vagina		1	
DN930;Livmoderblødning efter coitus eller kontaktblødning		1	
DN930A;Livmoderblødning efter coitus		1	
DN930B;Kontaktblødning		1	

Supplement 2 Nielsen et al. Mortality after paediatric emergency calls for patients with and without pre-existing comorbidity (2023)

DN938;Anden form for abnorm blødning fra livmoderen eller vagina		1	
DN938A;Anden form for abnorm blødning fra livmoderen		1	
DN938B;Anden form for abnorm blødning fra vagina		1	
DN938C;Abnorm blødning fra livmoderen efter fødsel		1	
DN939;Abnorm blødning fra livmoderen eller vagina UNS		1	
DN939A;Abnorm blødning fra vagina UNS		1	
DN939B;Abnorm blødning fra livmoderen UNS		1	
DN94;Smerter og andre tilstande forbundet med kvindelige kønsorganer og menstruation		1	
DN940;Ovulationssmerter		1	
DN941;Dyspareuni		1	
DN942;Vaginismus		1	
DN943;Præmenstruelt syndrom		1	
DN944;Primære menstruationssmerter		1	
DN945;Sekundære menstruationssmerter		1	
DN946;Dysmenoré UNS		1	
DN948;Anden tilstand forbundet med kvindelige kønsorganer eller menstruation		1	
DN948A;Vulvodynia		1	
DN949;Tilstand forbundet med kvindelige kønsorganer eller menstruation UNS		1	
DN95;Klimakterielle tilstande		1	
DN950;Postmenopausal metroragi		1	
DN951;Klimakterielle symptomer		1	
DN951A;Klimakteriel hovedpine		1	
DN951B;Klimakteriel hedetur		1	
DN951C;Klimakteriel søvnløshed		1	
DN951D;Neurosis climacterica		1	
DN951E;Klimakteriel svimmelhed		1	
DN952;Senil atrofisk vaginitis		1	
DN952A;Postmenopausal atrofisk vaginitis		1	
DN953;Tilstande ved kunstigt fremkaldt menopause		1	
DN958;Anden klimakteriel tilstand		1	
DN959;Klimakteriel tilstand UNS		1	
DN96;Gentagne spontane aborter		1	

DN969;Habituelle aborter		1	
DN97;Kvindelig infertilitet		1	
DN970;Kvindelig infertilitet ved anovulation		1	
DN971;Kvindelig infertilitet ved tilstande i æggelederne		1	
DN972;Kvindelig infertilitet ved tilstande i livmoderen		1	
DN973;Kvindelig infertilitet ved tilstande i livmoderhalsen		1	
DN974;Kvindelig infertilitet ved tilstande hos manden		1	
DN978;Kvindelig infertilitet forårsaget af andre forhold		1	
DN978A;Sekundær kvindelig infertilitet		1	
DN978B;Kvindelig infertilitet ved tilstande i ovarierne		1	
DN978B1;Kvindelig infertilitet efter tilbagelægning af kryopræserveret ovarievæv		1	
DN978C;Kvindelig infertilitet ved multiple årsager		1	
DN978D;Primær kvindelig sterilitet		1	
DN978E;Kvindelig infertilitet ved tilstande i hypofysen		1	
DN978F;Kvindelig infertilitet ved tilstande i hypotalamus		1	
DN979;Kvindelig infertilitet UNS		1	
DN98;Komplikationer til kunstig befrugtning		1	
DN980;Infektion opstået ved kunstig befrugtning		1	
DN981;Ovarielt hyperstimulationssyndrom		1	
DN981A;Ovarielt hyperstimulationssyndrom, mild		1	
DN981B;Ovarielt hyperstimulationssyndrom, moderat		1	
DN981C;Ovarielt hyperstimulationssyndrom, svær		1	
DN981D;Ovarielt hyperstimulationssyndrom, kritisk		1	
DN982;Komplikation ved implantering af in vitro-fertiliseret æg		1	
DN983;Komplikation ved forsøg på oplægning af embryo		1	
DN984;Abdominalsmerter som komplikation til kunstig befrugtning uden tegn på hyperstimulation		1	
DN985;Intraperitoneal infektion efter ægaspiration		1	
DN986;Blødning i forbindelse med ægaspiration		1	
DN988;Anden komplikation til kunstig befrugtning		1	
DN989;Komplikation til kunstig befrugtning UNS		1	
DN99;Tilstande i urin- og kønsorganer efter kirurgiske og medicinske indgreb IKA		1	
DN990;Uræmi efter kirurgisk eller medicinsk indgreb		1	

Supplement 2 Nielsen et al. Mortality after paediatric emergency calls for patients with and without pre-existing comorbidity (2023)

DN991;Uretrastriktur efter kirurgisk indgreb eller kateterisation		1	
DN991A;Uretrastriktur efter kirurgisk indgreb		1	
DN991B;Uretrastriktur efter kateterisation		1	
DN992;Vaginaladhærencer efter kirurgisk indgreb		1	
DN993;Vaginal prolaps efter hysterektomi		1	
DN994;Peritoneale adhærencer i bækkenet efter kirurgisk eller medicinsk indgreb		1	
DN995;Dysfunktion af udvendigt urinvejsstoma		1	
DN998;Anden tilstand i urin- eller kønsorgan efter kirurgisk eller medicinsk indgreb		1	
DN998A;Vaginaltopsruptur efter hysterektomi		1	
DN998A1;Vaginaltopsruptur efter hysterektomi, delvis defekt		1	
DN998A2;Vaginaltopsruptur efter hysterektomi, fuldvægsdefekt		1	
DN999;Tilstand UNS i urin- eller kønsorgan efter kirurgisk eller medicinsk indgreb		1	
DO00;Graviditet uden for livmoderen		1	
DO000;Ektopisk graviditet i bughulen		1	
DO001;Ektopisk graviditet i æggeleder		1	
DO002;Ektopisk graviditet i æggestok		1	
DO003;Heterotop graviditet		1	
DO003A;Heterotop graviditet ved spontan graviditet		1	
DO003B;Heterotop graviditet ved IUI/IVF/ICSI		1	
DO008;Anden form for ektopisk graviditet		1	
DO008A;Ektopisk graviditet i livmoderhalsen		1	
DO008B;Ektopisk graviditet i cornu uteri		1	
DO008C;Ektopisk graviditet i livmoderens ligamenter		1	
DO008D;Ektopisk graviditet i livmodervæggen		1	
DO008E;Graviditet i cicatrice efter kejsersnit		1	
DO009;Ektopisk graviditet UNS		1	
DO01;Molagraviditet		1	
DO010;Komplet mola		1	
DO011;Partiel mola		1	
DO019;Mola UNS		1	
DO019A;Ektopisk mola		1	
DO02;Andre unormale graviditetsprodukter		1	

DO020;Graviditet uden erkendt foster (blighted ovum)	1		
DO021;Graviditet med dødt retineret foster (missed abortion)	1		
DO022;Afsluttet graviditet med ukendt lokalisation (PUL)	1		
DO028;Andet abnormt graviditetsprodukt	1		
DO029;Abnormt graviditetsprodukt UNS	1		
DO03;Spontan abort	1		
DO030;Inkomplet spontan abort kompliceret med underlivsinfektion	1		
DO031;Inkomplet spontan abort kompliceret med sen eller excessiv blødning	1		
DO032;Inkomplet spontan abort kompliceret med emboli	1		
DO033;Inkomplet spontan abort med anden eller ikke specificeret komplikation	1		
DO034;Inkomplet spontan abort uden komplikation	1		
DO035;Komplet eller ikke specificeret spontan abort kompliceret med underlivsinfektion	1		
DO036;Komplet eller ikke specificeret spontan abort kompliceret med sen eller excessiv blødning	1		
DO037;Komplet eller ikke specificeret spontan abort kompliceret med emboli	1		
DO038;Komplet eller ikke specificeret spontan abort kompliceret med anden komplikation	1		
DO039;Komplet eller ikke specificeret spontan abort uden komplikation	1		
DO04;Provokeret abort før udgangen af 12. graviditetsuge	1		
DO043;Prov. abort før 12 grav-uger, inkomplet, m an. komplikation (findes ikke i SKS)	1		
DO044;Prov. abort før 12 grav-uger, inkomplet u komplikation (findes ikke i SKS)	1		
DO049;Provokeret abort før udgangen af 12. graviditetsuge UNS	1		
DO05;Provokeret abort efter udgangen af 12. graviditetsuge	1		
DO050;Provokeret abort efter udgangen af 12. graviditetsuge uden samrådstilladelse ved fare for kvindens liv eller helbred	1		
DO056;Prov. abort eft. 12 grav-uger, samråd v ung alder/umodenhed (findes ikke i SKS)	1		
DO059;Provokeret abort efter udgangen af 12. graviditetsuge med samrådstilladelse	1		
DO06;Anden provokeret abort med samrådstilladelse	1		
DO069;Provokeret abort før udgangen af 12. graviditetsuge med samrådstilladelse	1		
DO07;Mislykket fremkaldelse af abort	1		
DO079;Fortsat levende graviditet efter forsøg på provokeret abort	1		
DO08;Komplikation efter abort, ektopisk graviditet og mola	1		
DO080;Underlivsinfektion efter abort, ektopisk graviditet eller mola	1		
DO080B;Endometritis efter abort	1		
DO080O;Salpingitis efter abort	1		

Supplement 2 Nielsen et al. Mortality after paediatric emergency calls for patients with and without pre-existing comorbidity (2023)

DO080U;Sepsis efter abort	1		
DO081;Sen eller excessiv blødning efter abort, ektopisk graviditet eller mola	1		
DO081G;Blødning efter abort	1		
DO082;Emboli efter abort, ektopisk graviditet eller mola	1		
DO083;Shock efter abort, ektopisk graviditet eller mola	1		
DO084;Nyresvigt efter abort, ektopisk graviditet eller mola	1		
DO085;Metabolisk sygdom efter abort, ektopisk graviditet eller mola	1		
DO086;Beskadigelse af organ eller væv i bækkenet efter abort, ektopisk graviditet eller mola	1		
DO086R;Beskadigelse af livmoderen efter abort	1		
DO087;Anden venøs komplikation efter abort, ektopisk graviditet eller mola	1		
DO088;Andre komplikationer efter abort, ektopisk graviditet eller mola	1		
DO088L;Retineret væv efter abort	1		
DO089;Komplikation efter abort, ektopisk graviditet eller mola UNS	1		
DO10;Graviditet, fødsel og barsel med forud bestående komplicerende blodtryksforhøjelse	1		
DO100;Graviditet, fødsel eller barsel med forud bestående komplicerende blodtryksforhøjelse	1		
DO101;Graviditet, fødsel eller barsel med forud bestående komplicerende hypertensiv hjertesygdom	1		
DO102;Graviditet, fødsel eller barsel med forud bestående komplicerende hypertensiv nyresygdom	1		
DO103;Graviditet, fødsel eller barsel med forud bestående komplicerende hypertensiv hjertesygdom og nyresygdom	1		
DO104;Graviditet, fødsel eller barsel med forud bestående komplicerende sekundær blodtryksforhøjelse	1		
DO109;Graviditet, fødsel eller barsel med forud bestående komplicerende blodtryksforhøjelse UNS	1		
DO11;Præeklampsi ved forud bestående kronisk hypertension	1		
DO119;Graviditet, fødsel eller barsel med forud bestående komplicerende blodtryksforhøjelse og proteinuri	1		
DO119A;Graviditet med forud bestående komplicerende blodtryksforhøjelse og proteinuri	1		
DO119B;Fødsel med forud bestående komplicerende blodtryksforhøjelse og proteinuri	1		
DO119C;Barsel med forud bestående komplicerende blodtryksforhøjelse og proteinuri	1		
DO12;Graviditetsbetinget ødem og proteiner i urinen uden blodtryksforhøjelse	1		
DO120;Graviditetsødem	1		
DO121;Proteinuri i graviditeten	1		
DO122;Ødem og proteinuri i graviditeten	1		
DO13;Graviditetsbetinget blodtryksforhøjelse uden proteiner i urinen	1		
DO139;Graviditetsbetinget hypertension uden proteinuri	1		
DO14;Præeklampsi	1		

Supplement 2 Nielsen et al. Mortality after paediatric emergency calls for patients with and without pre-existing comorbidity (2023)

DO140;Let til moderat præeklampsi		1	
DO141;Svær præeklampsi		1	
DO142;HELLP-syndrom		1	
DO149;Præeklampsi UNS		1	
DO15;Fødselskrampe		1	
DO150;Eklampsi		1	
DO151;Eklampsi under fødsel		1	
DO152;Eklampsi i barselsperiode		1	
DO159;Eklampsi UNS		1	
DO16;Forhøjet blodtryk uden nærmere specificering under graviditet		1	
DO169;Blodtryksforhøjelse under graviditet UNS		1	
DO20;Blødning tidligt i graviditeten		1	
DO200;Truende abort		1	
DO208;Anden blødning før 22 graviditetsuger		1	
DO208A;Accidentel blødning tidligt i graviditeten		1	
DO208B;Blødning ved fibrinolyse tidligt i graviditeten		1	
DO208C;Blødning ved koagulationsdefekt tidligt i graviditeten		1	
DO209;Blødning tidligt i graviditeten UNS		1	
DO21;Opkastninger i graviditeten		1	
DO210;Graviditetsopkastninger i lettere grad		1	
DO211;Svære graviditetsopkastninger med metaboliske forstyrrelser		1	
DO212;Opkastninger efter 22 graviditetsuger		1	
DO218;Opkastninger under graviditeten ved sygdom klassificeret andetsteds		1	
DO219;Opkastninger i graviditeten UNS		1	
DO22;Venøse komplikationer og hæmorider i graviditeten		1	
DO220;Varicer på underekstremitet i graviditeten		1	
DO221;Varicer på kønsorganer i graviditeten		1	
DO221A;Varicer i perineum i graviditeten		1	
DO221B;Varicer i vagina i graviditeten		1	
DO221C;Varicer i vulva i graviditeten		1	
DO222;Overfladisk tromboflebitis i graviditeten		1	
DO223;Dyb tromboflebitis i graviditeten		1	

Supplement 2 Nielsen et al. Mortality after paediatric emergency calls for patients with and without pre-existing comorbidity (2023)

DO224;Hæmorider i graviditeten		1	
DO225;Venøs trombose i hjernen i graviditeten		1	
DO225A;Trombose i sinus venosi cerebri i graviditeten		1	
DO228;Anden venøs komplikation i graviditeten		1	
DO229;Venøs komplikation i graviditeten UNS		1	
DO23;Underlivs- og urinvejsinfektioner i graviditeten		1	
DO230;Pyelonefritis i graviditeten		1	
DO231;Cystitis i graviditeten		1	
DO232;Uretritis i graviditeten		1	
DO233;Anden urinvejsinfektion i graviditeten		1	
DO234;Urinvejsinfektion i graviditeten UNS		1	
DO235;Underlivsinfektion i graviditeten		1	
DO235A;Gruppe B streptokok-syndrom i graviditeten		1	
DO239;Anden eller ikke specificeret underlivs- eller urinvejsinfektion i graviditeten		1	
DO24;Diabetes under graviditet, fødsel og barsel		1	
DO240;Graviditet, fødsel eller barsel med forud bestående type 1-diabetes		1	
DO240A;Graviditet med forud bestående type 1-diabetes		1	
DO240B;Fødsel med forud bestående type 1-diabetes		1	
DO240C;Barsel med forud bestående type 1-diabetes		1	
DO241;Graviditet, fødsel eller barsel med forud bestående type 2-diabetes		1	
DO241A;Graviditet med forud bestående type 2-diabetes		1	
DO241B;Fødsel med forud bestående type 2-diabetes		1	
DO241C;Barsel med forud bestående type 2-diabetes		1	
DO242;Graviditet, fødsel eller barsel med forud bestående diabetes forårsaget af underernæring		1	
DO242A;Graviditet med forud bestående diabetes forårsaget af underernæring		1	
DO242B;Fødsel med forud bestående diabetes forårsaget af underernæring		1	
DO242C;Barsel med forud bestående diabetes forårsaget af underernæring		1	
DO243;Graviditet, fødsel eller barsel med forud bestående diabetes UNS		1	
DO243A;Graviditet med forud bestående diabetes UNS		1	
DO243B;Fødsel med forud bestående diabetes UNS		1	
DO243C;Barsel med forud bestående diabetes UNS		1	
DO244;Graviditet, fødsel eller barsel med gestationel diabetes		1	

DO244B;Fødsel med gestationel diabetes		1	
DO244C;Barsel med gestationel diabetes		1	
DO244D;Graviditet med gestationel diabetes		1	
DO244E;Graviditet med insulinbehandlet gestationel diabetes		1	
DO245;Nyopdaget manifest diabetes i graviditeten		1	
DO249;Graviditet, fødsel eller barsel med diabetes UNS		1	
DO249A;Graviditet med diabetes UNS		1	
DO249B;Fødsel med diabetes UNS		1	
DO249C;Barsel med diabetes UNS		1	
DO25;Fejlernæring under graviditet, fødsel og barselsperiode		1	
DO259;Fejlernæring under graviditet, fødsel eller barselsperiode UNS		1	
DO26;Andre sygdomme overvejende relateret til graviditeten		1	
DO260;Overdreven vægtøgning i graviditeten		1	
DO261;For lille vægtøgning i graviditeten		1	
DO262;Graviditet efter tidligere habituel abort		1	
DO263;Graviditet med spiral (IUD) in situ		1	
DO264;Herpes gestationis		1	
DO265;Hypotensio arterialis gestationis		1	
DO266;Leversygdom ved graviditet, fødsel eller barsel		1	
DO266A;Leversygdom i graviditeten		1	
DO266B;Leversygdom i forbindelse med fødsel		1	
DO266C;Leversygdom i barselsperioden		1	
DO266D;Prurigo gravidarum		1	
DO266F;Akut gul leveratrofi i graviditeten		1	
DO266G;Graviditetskløe med leverpåvirkning		1	
DO267;Bækkenløsning i graviditeten		1	
DO267A;Bækkeninsufficiens under graviditeten		1	
DO267B;Bækkenløsning under graviditeten		1	
DO268;Anden sygdom relateret til graviditeten		1	
DO268A;Graviditetstræthed		1	
DO268B;Molimina gravidarum		1	
DO268C;Nefropati i graviditeten		1	

DO268D;Perifer neuritis i graviditet		1	
DO268E;Papuløst udslæt i graviditet		1	
DO268F;Graviditetskløe		1	
DO268G;Polymorft udslæt i graviditet		1	
DO268H;PUPPP (pruritic urticarial papules and plaques of pregnancy)		1	
DO268J;Graviditetsdermatose		1	
DO269;Sygdom relateret til graviditeten UNS		1	
DO28;Abnorme fund ved graviditet		1	
DO280;Abnormt hæmatologisk fund ved graviditet		1	
DO281;Abnormt biokemisk fund ved graviditet		1	
DO282;Abnormt cytologisk fund ved graviditet		1	
DO283;Abnormt ultralydsfund hos moderen ved graviditet		1	
DO283A;Abnormt Doppler-fund hos moderen ved graviditet		1	
DO283A1;Abnormt flow i a. uterina ved graviditet		1	
DO284;Abnorm radiologisk undersøgelse ved graviditet		1	
DO285;Abnormt fund i kromosomer eller gener hos moder ved graviditet		1	
DO285A;Abnormt fund i kromosomer hos moder ved graviditet		1	
DO285B;Abnormt fund i gener hos moder ved graviditet		1	
DO288;Andet abnormt fund hos moder ved graviditet		1	
DO289;Abnormt fund hos moder ved graviditet UNS		1	
DO29;Komplikationer til anæstesi i graviditeten		1	
DO290;Lungekomplikation til anæstesi under graviditeten		1	
DO290A;Aspirationspneumoni efter anæstesi under graviditeten		1	
DO290B;Atelektase efter anæstesi under graviditeten		1	
DO290C;Postaspirationssyndrom efter anæstesi under graviditeten		1	
DO291;Hjertekomplikation til anæstesi under graviditeten		1	
DO291A;Hjertesvigt ved anæstesi under graviditeten		1	
DO291B;Hjertestop ved anæstesi under graviditeten		1	
DO292;Komplikation i centralnervesystemet ved anæstesi under graviditeten		1	
DO292A;Cerebral anoksi ved anæstesi under graviditeten		1	
DO293;Toksisk reaktion på lokalanæstesi under graviditeten		1	
DO294;Hovedpine efter spinal eller epidural anæstesi under graviditeten		1	

DO295;Anden komplikation efter spinal eller epidural anæstesi under graviditeten		1	
DO296;Vanskelig eller mislykket trakeal intubation under graviditeten		1	
DO298;Anden komplikation til anæstesi under graviditeten		1	
DO299;Komplikation til anæstesi under graviditeten UNS		1	
DO30;Flerfoldsgraviditet		1	
DO300;Tvillingegraviditet		1	
DO300C;Dikoriske, diamnioniske tvillinger		1	
DO300D;Monokoriske, diamnioniske tvillinger		1	
DO300E;Monoamnioniske tvillinger		1	
DO301;Trillingegraviditet		1	
DO302;Firlingegraviditet		1	
DO308;Anden flerfoldsgraviditet		1	
DO308A;Fortsat graviditet efter fosterreduktion af et eller flere fostre i flerfoldsgraviditet		1	
DO308A1;Fortsat graviditet efter fosterreduktion af et eller flere fostre i flerfoldsgraviditet før 22 graviditetssuger		1	
DO308A2;Fortsat graviditet efter fosterreduktion af et eller flere fostre i flerfoldsgraviditet efter 22 graviditetssuger		1	
DO309;Flerfoldsgraviditet UNS		1	
DO31;Komplikationer, som er specifikke for flerfoldsgraviditet		1	
DO310;Flerfoldsgraviditet med et foetus papyraceus		1	
DO311;Fortsat graviditet efter spontan abort af et eller flere fostre i flerfoldsgraviditet		1	
DO312;Fortsat graviditet efter intrauterin død af et eller flere fostre i flerfoldsgraviditet		1	
DO312C;Flerfoldsgraviditet med intrauterin død af foster før 22 graviditetsuger		1	
DO312D;Flerfoldsgraviditet med intrauterin død af foster efter 22 graviditetsuger		1	
DO318;Anden komplikation til flerfoldsgraviditet		1	
DO32;Graviditet med erkendt eller mistænkt abnorm fosterstilling		1	
DO320;Ustabil fosterstilling		1	
DO321;Underkropsstilling		1	
DO322;Tværleje eller skråleje		1	
DO322A;Tværleje		1	
DO322B;Skråleje		1	
DO323;Pande- eller ansigtsstilling		1	
DO323A;Pandestilling		1	
DO323B;Ansigtsstilling		1	

DO324;Højtstående foster ved termin		1	
DO325;Flerfoldsgraviditet med malposition af et eller flere fostre		1	
DO326;Fremfald af hånd eller fod		1	
DO328;Anden abnorm fosterstilling		1	
DO329;Abnorm fosterstilling UNS		1	
DO33;Graviditet med erkendt eller mistænkt mekanisk misforhold		1	
DO330;Mekanisk misforhold på grund af bækkendeformitet		1	
DO331;Mekanisk misforhold på grund af generelt forsnævret bækken		1	
DO332;Mekanisk misforhold på grund af forsnævret bækkenindgang		1	
DO333;Mekanisk misforhold på grund af tragtbækken		1	
DO334;Mekanisk misforhold på grund af tilstande både hos moder og foster		1	
DO335;Mekanisk misforhold på grund af for stort foster		1	
DO336;Mekanisk misforhold på grund af foster med hydrocefalus		1	
DO337;Mekanisk misforhold på grund af anden misdannelse af foster		1	
DO337A;Mekanisk misforhold på grund af sakralt teratom hos foster		1	
DO337B;Mekanisk misforhold på grund af sammenvoksede tvillinger		1	
DO337C;Mekanisk misforhold på grund af tumor hos foster		1	
DO337D;Mekanisk misforhold på grund af ascites hos foster		1	
DO337E;Mekanisk misforhold på grund af hydrops foetalis		1	
DO337F;Mekanisk misforhold på grund af meningomyelocele hos foster		1	
DO338;Mekanisk misforhold af anden årsag		1	
DO339;Mekanisk misforhold UNS		1	
DO339A;Mekanisk misforhold mellem fosterhoved og bækken UNS		1	
DO339B;Mekanisk misforhold mellem foster og bækken UNS		1	
DO34;Graviditet med erkendt eller mistænkt abnorm tilstand i den bløde fødselsvej		1	
DO340;Graviditet med misdannelse i livmoderen		1	
DO340A;Graviditet i bikorn livmoder		1	
DO341;Graviditet med fibromyom i livmoderen		1	
DO342;Graviditet med cicatrice i livmoderen efter tidligere indgreb		1	
DO343;Graviditet med cervixinsufficiens		1	
DO343A;Klinisk cervixinsufficiens		1	
DO343B;Ultralydpåvist cervixinsufficiens		1	

DO344;Graviditet med anden abnorm tilstand i livmoderhalsen		1	
DO344A;Graviditet med cicatrice i livmoderhalsen efter tidligere indgreb		1	
DO344B;Graviditet efter tidligere konisation af livmoderhalsen		1	
DO344C;Graviditet med polyp på livmoderhalsen		1	
DO344D;Graviditet med stenose af livmoderhalsen		1	
DO344E;Graviditet med striktur af livmoderhalsen		1	
DO344F;Graviditet med tumor i livmoderhalsen		1	
DO345;Graviditet med anden abnorm tilstand i livmoderen		1	
DO345A;Graviditet med nedsynkning af livmoderen		1	
DO345B;Graviditet med inkarceration af livmoderen		1	
DO345C;Graviditet med prolaps af livmoderen		1	
DO345D;Graviditet med retroversion af livmoderen		1	
DO346;Graviditet med abnorm tilstand i vagina		1	
DO346A;Graviditet med cicatrice i vagina efter tidligere indgreb		1	
DO346B;Graviditet med septum i vagina		1	
DO346C;Graviditet med stenose i vagina		1	
DO346D;Graviditet med striktur i vagina		1	
DO346E;Graviditet med tumor i vagina		1	
DO347;Graviditet med abnorm tilstand i ydre kønsdele eller perineum		1	
DO347A;Graviditet med abnorm tilstand i perineum		1	
DO347B;Graviditet med abnorm tilstand i ydre kønsdele		1	
DO347C;Graviditet med cicatrice efter tidligere indgreb i perineum		1	
DO347D;Graviditet med cicatrice efter tidligere indgreb i ydre kønsdele		1	
DO347E;Graviditet med perineum rigidum		1	
DO347F;Graviditet med tumor i ydre kønsdele		1	
DO348;Graviditet med anden abnorm tilstand i den bløde fødselsvej		1	
DO348A;Graviditet med abdomen pendens		1	
DO348B;Graviditet med cicatrice i bækkenbunden efter tidligere indgreb		1	
DO348C;Graviditet med cystocele		1	
DO348D;Graviditet med rektocele		1	
DO348G;Graviditet med kort cervix (<25 mm)		1	
DO349;Graviditet med abnorm tilstand i den bløde fødselsvej UNS		1	

Supplement 2 Nielsen et al. Mortality after paediatric emergency calls for patients with and without pre-existing comorbidity (2023)

DO35;Graviditet med erkendt eller mistænkt unormalt eller beskadiget foster		1	
DO350;Graviditet med neuralrørsdefekt hos foster		1	
DO350A;Graviditet med anencefalt foster		1	
DO350B;Graviditet med spina bifida hos foster		1	
DO351;Graviditet med kromosomanomali hos foster		1	
DO352;Graviditet med arvelig sygdom hos foster		1	
DO353;Graviditet med fosterskade efter maternel virusinfektion		1	
DO353A;Graviditet med fosterskade efter maternel rubellainfektion		1	
DO353B;Graviditet med fosterskade efter maternel cytomegalovirusinfektion		1	
DO354;Graviditet med alkoholisk fosterskade		1	
DO355;Graviditet med fosterskade forårsaget af lægemiddel		1	
DO355A;Graviditet med fosterskade forårsaget af stofmisbrug		1	
DO356;Graviditet med fosterskade efter stråling		1	
DO357;Graviditet med fosterskade efter indgreb eller anden behandling		1	
DO357A;Graviditet med fosterskade efter intrauterint indgreb på foster		1	
DO357B;Graviditet med fosterskade efter prænatalt intrauterint indgreb		1	
DO357C;Graviditet med fosterskade efter indgreb på moder		1	
DO357D;Graviditet med fosterskade efter hæmatologisk undersøgelse		1	
DO357E;Graviditet med fosterskade efter biopsi		1	
DO357F;Graviditet med fosterskade efter amniocentese		1	
DO357G;Graviditet med fosterskade forårsaget af spiral		1	
DO358;Graviditet med anden abnorm tilstand eller skade hos foster		1	
DO358A;Graviditet med fosterskade ved listeriose		1	
DO358B;Graviditet med fosterskade ved toksoplasmose		1	
DO359;Graviditet med anden abnorm tilstand eller skade hos foster UNS		1	
DO359A;Graviditet med misdannet foster fundet ved UL UNS		1	
DO359B;Graviditet med andet abnormt ultralydsfund hos fosteret UNS		1	
DO36;Graviditet med andre abnorme tilstande hos foster		1	
DO360;Graviditet med rhesusimmunisering		1	
DO361;Graviditet med anden immunisering		1	
DO361A;Graviditet med AB0-immunisering		1	
DO361B;Graviditet med trombocytimmunisering		1	

DO362;Graviditet med hydrops foetalis		1	
DO362A;Graviditet med ascites hos foster		1	
DO362B;Graviditet med perikardieekssudat hos foster		1	
DO362C;Graviditet med pleuraekssudat hos foster		1	
DO362D;Graviditet med generaliseret subkutant ødem hos foster		1	
DO363;Graviditet med truende hypoxi hos foster		1	
DO364;Graviditet med intrauterin fosterdød		1	
DO365;Graviditet med estimeret fostervægt mindre end forventet ved gestationsalder (SGA)		1	
DO365A;Graviditet med placentainsufficiens		1	
DO365B;Graviditet med abnormt flow (>2SD), grad I i a. umbilicalis		1	
DO365C;Graviditet med abnormt flow (>3SD), grad II i a. umbilicalis		1	
DO365D;Graviditet med abnormt flow, grad III i a. umbilicalis		1	
DO366;Graviditet med stort foster		1	
DO367;Levende foster ved abdominal graviditet		1	
DO368;Graviditet med anden abnorm tilstand hos foster		1	
DO368A;Graviditet med hjertearytmi hos foster		1	
DO368B;Graviditet med abnormt flow over trikuspidalklappen hos foster		1	
DO368D;Graviditet med abnormt flow i ductus arteriosus hos foster		1	
DO368E;Graviditet med abnormt flow i a. cerebri media hos foster		1	
DO368E1;Graviditet med abnormt flow i a. cerebri media hos foster, føtal redistribuering		1	
DO368E2;Graviditet med abnormt flow i a. cerebri media hos foster, PSV>1,5 MoM		1	
DO368F;Graviditet med abnormt flow i ductus venosus hos foster		1	
DO368G;Graviditet med abnormt flow i v. umbilicalis hos foster		1	
DO368H;Graviditet med abnormt flow i andre vener hos foster		1	
DO368K;Graviditet med parvovirus infektion hos foster		1	
DO368L;Graviditet med CMV infektion hos foster		1	
DO368M;Graviditet med toksoplasmoseinfektion hos foster		1	
DO368N;Graviditet med anden vrificeret infektion hos foster		1	
DO368P;Graviditet med anæmi hos foster		1	
DO368Q;Graviditet med øget nakkefold hos foster i 11-14 uger		1	
DO368Q1;Graviditet med øget nakkefold hos foster i 11-14 uger, 95-99 percentil		1	
DO368Q2;Graviditet med øget nakkefold hos foster i 11-14 uger, >99 percentil		1	

DO368R;Graviditet med ekkogent fokus i fosterhjerte		1	
DO368S;Graviditet med hyperekkogen tarm hos foster		1	
DO368T;Graviditet med nakkeødem hos foster i 2. trimester		1	
DO369;Graviditet med abnorm tilstand hos foster UNS		1	
DO40;Graviditet med for meget fostervand		1	
DO409;Hydramnion		1	
DO41;Andre sygdomme i amnionvæske og fosterhinder		1	
DO410;Oligohydramnion		1	
DO411;Infektion i amnionhule og fosterhinder		1	
DO411A;Amnionitis		1	
DO411B;Chorioamnionitis		1	
DO411C;Membranitis amnii		1	
DO411D;Placentitis		1	
DO418;Anden sygdom i amnionvæske eller fosterhinder		1	
DO418A;Amnionbånd		1	
DO418B;Amniondetachment		1	
DO418C;Chorionbånd		1	
DO419;Sygdom i amnionvæske eller fosterhinder UNS		1	
DO42;Vandafgang		1	
DO420;Vandafgang før 37. graviditetsuge med veer indenfor 24 timer (PPROM)		1	
DO422;For tidlig vandafgang før 37. graviditetsuge uden regelmæssige veer (PPROM)		1	
DO423;Vandafgang efter 37. graviditetsuge uden regelmæssige veer (PROM)		1	
DO424;Vandafgang før 37. graviditetsuge med medicinsk vehæmning (PPROM)		1	
DO429;Vandafgang UNS		1	
DO43;Sygdomme i moderkagen		1	
DO430;Placentatransfusionssyndrom		1	
DO430C;Tvilling-til-tvilling transfusion		1	
DO430D;Føtomaternel transfusion		1	
DO430E;Maternoføtal transfusion		1	
DO431;Placenta-anomali		1	
DO431A;Circumvallat placenta		1	
DO431C;Placenta med biplacenta		1	

Supplement 2 Nielsen et al. Mortality after paediatric emergency calls for patients with and without pre-existing comorbidity (2023)

DO431E;Vasa Prævia		1		
DO431F;Velamentøs insertion af navlesnoren		1		
DO438;Anden sygdom i placenta		1		
DO438A;Placentadegeneration		1		
DO438B;Placentafibrose		1		
DO438C;Placentainfarkt		1		
DO438D;Placentavaricer		1		
DO438E;Subchorisk hæmatom		1		
DO439;Sygdom i moderkagen UNS		1		
DO44;Forliggende moderkage		1		
DO440;Forliggende moderkage uden blødning		1		
DO440A;Totalt forliggende moderkage uden blødning		1		
DO440B;Partielt forliggende moderkage uden blødning		1		
DO441;Forliggende moderkage med blødning		1		
DO441A;Totalt forliggende moderkage med blødning		1		
DO441B;Partielt forliggende moderkage med blødning		1		
DO449;Forliggende moderkage UNS		1		
DO45;For tidlig løsning af moderkagen		1		
DO450;For tidlig løsning af moderkagen med koagulationsdefekt		1		
DO458;Anden form for for tidlig løsning af moderkagen		1		
DO459;For tidlig løsning af moderkagen UNS		1		
DO46;Vaginalblødning før fødsel IKA		1		
DO460;Blødning før fødsel med koagulationsdefekt		1		
DO468;Anden form for blødning før fødsel		1		
DO468A;Blødning i graviditet efter 22 graviditetsuger		1		
DO469;Blødning før fødsel UNS		1		
DO47;Plukkeveer og truende for tidlig fødsel		1		
DO470;Plukkeveer før 37 graviditetsuger		1		
DO471;Plukkeveer efter 37 graviditetsuger		1		
DO472;Truende for tidlig fødsel		1		
DO479;Plukkeveer UNS		1		
DO48;Overbåren graviditet		1		

DO489;Overbåren graviditet UNS		1	
DO49;Graviditetslængde som hovedindikation for igangsættelse af fødsel		1	
DO499;Graviditetslængde som hovedindikation for igangsættelse af fødsel		1	
DO60;Veer før termin		1	
DO600;Veer før termin uden fødsel		1	
DO601;Veer før termin med for tidlig fødsel		1	
DO602;Veer før termin med fødsel til termin		1	
DO603;For tidlig fødsel uden spontane veer		1	
DO609;For tidlig fødsel UNS		1	
DO61;Mislykket igangsættelse af fødsel		1	
DO610;Mislykket medikamentel igangsættelse af fødsel		1	
DO610A;Mislykket igangsættelse af fødsel med prostaglandin		1	
DO610B;Mislykket igangsættelse af fødsel med oxytocin		1	
DO611;Mislykket instrumentel igangsættelse af fødsel		1	
DO618;Mislykket igangsættelse af fødsel med anden metode		1	
DO619;Mislykket igangsættelse af fødsel UNS		1	
DO62;Veabnormiteter		1	
DO620;Primær vesvækkelse		1	
DO620A;Primær vesvækkelse ved cervix uteri rigida		1	
DO620B;Primær vesvækkelse ved collum uteri rigidum		1	
DO620C;Primær vesvækkelse ved orificium uteri rigidum		1	
DO620F;Vesvækkelse i udvidelsesperioden		1	
DO621;Sekundær vesvækkelse		1	
DO621A;Svækkelse af presseveer		1	
DO622;Anden form for vesvækkelse		1	
DO622A;Vesvækkelse UNS		1	
DO623;Styrtfødsel		1	
DO624;Hypertone og ukoordinerede veer		1	
DO624A;Bandls ring		1	
DO624B;Contractiones uteri prolongatae		1	
DO624C;Uterusdystoci UNS		1	
DO624D;Uterus hypertoni		1	

Supplement 2 Nielsen et al. Mortality after paediatric emergency calls for patients with and without pre-existing comorbidity (2023)

DO628;Anden veabnormitet		1	
DO629;Veabnormitet UNS		1	
DO63;Langvarig fødsel		1	
DO630;Fødsel med forlænget udvidelsesfase		1	
DO630A;Fødsel med forlænget latensfase		1	
DO631;Fødsel med forlænget uddrivelsesfase		1	
DO632;Forsinket fødsel af efterfølgende barn i flerfoldsfødsel		1	
DO632A;Forsinket fødsel af tvilling B		1	
DO639;Langvarig fødsel UNS		1	
DO64;Fødsel kompliceret af abnorm fosterstilling		1	
DO640;Fødsel kompliceret af ufuldstændig rotation af fosterets hoved		1	
DO640A;Fødsel kompliceret af dyb tværstand		1	
DO640B;Fødsel kompliceret af positio occipitotransversa persistens		1	
DO640C;Fødsel kompliceret af positio occipitosacralis persistens		1	
DO640D;Fødsel kompliceret af positio occipitoiliaca persistens		1	
DO640E;Fødsel kompliceret af positio occipitoposterior persistens		1	
DO640F;Fødsel kompliceret af uregelmæssig hovedpræsentation UNS		1	
DO640G;Fødsel kompliceret af høj lige stand		1	
DO640H;Fødsel kompliceret af asynklitisk hovedpræsentation		1	
DO641;Fødsel kompliceret af underkropspræsentation		1	
DO641A;Fødsel kompliceret af uerkendt underkropspræsentation		1	
DO642;Fødsel kompliceret af ansigtspræsentation		1	
DO643;Fødsel kompliceret af pandepræsentation		1	
DO644;Fødsel kompliceret af skulderpræsentation		1	
DO645;Fødsel kompliceret af fremfald af hånd eller fod		1	
DO648;Fødsel kompliceret af anden abnorm fosterstilling		1	
DO649;Fødsel kompliceret af abnorm fosterstilling UNS		1	
DO65;Fødsel kompliceret af abnormt bækken		1	
DO650;Fødsel kompliceret af bækkendeformitet		1	
DO651;Fødsel kompliceret af snævert bækken		1	
DO652;Fødsel kompliceret af forsnævret bækkenindgang		1	
DO653;Fødsel kompliceret af tragtbækken		1	

Supplement 2 Nielsen et al. Mortality after paediatric emergency calls for patients with and without pre-existing comorbidity (2023)

DO654;Fødsel kompliceret af føtopelvint misforhold uden specifkation		1	
DO655;Fødsel kompliceret af abnormitet i den bløde fødselsvej		1	
DO656;Fødsel kompliceret af perineum rigidum		1	
DO658;Fødsel kompliceret af anden abnormitet i det kvindelige bækken		1	
DO659;Fødsel kompliceret af abnormitet i det kvindelige bækken UNS		1	
DO66;Kompliceret fødsel af anden årsag		1	
DO660;Fødsel kompliceret af skulderdystoci		1	
DO661;Fødsel kompliceret af tvillingekollision		1	
DO662;Fødsel kompliceret af stort foster		1	
DO663;Fødsel kompliceret af abnormitet hos foster		1	
DO663A;Fødsel kompliceret af hydrocefalus hos foster		1	
DO663B;Fødsel kompliceret af lille foster		1	
DO663C;Fødsel kompliceret af tumor hos foster		1	
DO663D;Fødsel kompliceret af sammenvoksede tvillinger		1	
DO663E;Fødsel kompliceret af anencefali hos foster		1	
DO663F;Fødsel kompliceret af sakralt teratom hos foster		1	
DO663G;Fødsel kompliceret af meningomyelocele hos foster		1	
DO663H;Fødsel kompliceret af ascites hos foster		1	
DO663I;Fødsel kompliceret af hydrops hos foster		1	
DO664;Fødsel kompliceret af mislykket forsøg på vaginal fødsel		1	
DO665;Fødsel kompliceret af mislykket forsøg på fødsel med vakuumekstraktor eller tang		1	
DO665A;Fødsel kompliceret af mislykket forsøg på fødsel med vakuumekstraktor		1	
DO665B;Fødsel kompliceret af mislykket forsøg på fødsel med tang		1	
DO668;Fødsel kompliceret af anden årsag		1	
DO668A;Fødsel kompliceret af tidligere omskæring		1	
DO669;Kompliceret fødsel UNS		1	
DO67;Fødsel kompliceret af blødning IKA		1	
DO670;Fødsel kompliceret af blødning med koagulationsdefekt		1	
DO670B;Fødsel kompliceret af blødning med hypofibrinogenæmi		1	
DO670C;Fødsel kompliceret af blødning med afibrinogenæmi		1	
DO670D;Fødsel kompliceret af blødning med dissemineret intravaskulær koagulation		1	
DO670E;Fødsel kompliceret af blødning med hyperfibrinolyse		1	

Supplement 2 Nielsen et al. Mortality after paediatric emergency calls for patients with and without pre-existing comorbidity (2023)

DO678;Fødsel kompliceret af anden form for blødning		1	
DO678A;Fødsel kompliceret af excessiv blødning		1	
DO679;Fødsel kompliceret af blødning UNS		1	
DO68;Fødsel kompliceret af mistanke om asfyksi hos foster		1	
DO680;Fødsel kompliceret af påvirket hjertelyd hos foster		1	
DO680A;Fødsel kompliceret af takykardi hos foster		1	
DO680C;Fødsel kompliceret af bradykardi hos foster		1	
DO680D;Fødsel kompliceret af truende fosterdød		1	
DO680F;Fødsel kompliceret af påvirket hjertelyd hos foster med CTG-overvågning		1	
DO681;Fødsel kompliceret af mekoniumfarvet fostervand		1	
DO681A;Fødsel kompliceret af tyk mekonium		1	
DO682;Fødsel kompliceret af påvirket hjertelyd og mekoniumfarvet fostervand		1	
DO683;Fødsel kompliceret af biokemiske tegn til fosteracidose		1	
DO688;Fødsel kompliceret af andre tegn på asfyksi		1	
DO688A;Fødsel kompliceret af mistanke om asfyksi påvist ved ultralydsundersøgelse		1	
DO688B;Fødsel kompliceret af mistanke om asfyksi påvist ved STAN		1	
DO689;Fødsel kompliceret af mistanke om asfyksi UNS		1	
DO69;Fødsel med navlesnorskomplikation		1	
DO690;Fødsel kompliceret af fremfald af navlesnoren		1	
DO691;Fødsel kompliceret af navlesnoren om barnets hals		1	
DO692;Fødsel kompliceret af anden kompression af navlesnoren		1	
DO692A;Fødsel kompliceret af navlesnorstorsion		1	
DO692B;Fødsel kompliceret af navlesnorsstrangulation		1	
DO693;Fødsel kompliceret af for kort navlesnor		1	
DO694;Fødsel kompliceret af forliggende fosterhindekar		1	
DO694A;Fødsel kompliceret af blødning fra forliggende fosterhindekar		1	
DO695;Fødsel kompliceret af læsion af navlesnorens kar		1	
DO695A;Fødsel kompliceret af trombose i navlesnorens kar		1	
DO695B;Fødsel kompliceret af hæmatom i navlesnoren		1	
DO698;Fødsel kompliceret af anden navlesnorskomplikation		1	
DO698A;Fødsel kompliceret af ruptur i navlesnoren		1	
DO699;Fødsel kompliceret af navlesnorskomplikation UNS		1	

DO70;Bristning af mellemkød og slimhinder ved fødsel		1	
DO700;Fødselslæsion grad 1		1	
DO700A;Fødselslæsion med læsion af vulva, grad 1		1	
DO700B;Fødselslæsion med læsion af vagina, grad 1		1	
DO700C;Fødselslæsion med læsion af perineal hud, grad 1		1	
DO700D;Fødselslæsion med læsion af kønslæber, grad 1		1	
DO701;Fødselslæsion grad 2		1	
DO701B;Fødselslæsion med læsion af septum rectovaginalis		1	
DO702;Fødselslæsion grad 3		1	
DO702D;Fødselslæsion med delvis ruptur af eksterne analsfinkter (<50 pct), grad 3a		1	
DO702E;Fødselslæsion med total ruptur af eksterne analsfinkter (>50 pct), grad 3b		1	
DO702G;Fødselslæsion med ruptur af eksterne og interne analsfinkter, grad 3c		1	
DO703;Fødselslæsion grad 4		1	
DO704;Isoleret læsion af rektal- og analslimhinden ved fødsel		1	
DO709;Fødselslæsion i mellemkød og slimhinder UNS		1	
DO71;Andre fødselslæsioner		1	
DO710;Uterusruptur før fødslen		1	
DO710A;Partiel uterusruptur før fødslen		1	
DO710B;Total uterusruptur før fødslen		1	
DO711;Uterusruptur under eller efter fødslen		1	
DO711A;Uterusruptur under fødsel		1	
DO711AA;Partiel uterusruptur under fødsel		1	
DO711AB;Total uterusruptur under fødsel		1	
DO711B;Uterusruptur efter fødslen		1	
DO712;Uterusinversion efter fødslen		1	
DO713;Fødselslæsion i livmoderhalsen		1	
DO714;Fødsel med høj vaginalæsion uden læsion i perineum		1	
DO715;Anden fødselslæsion i den bløde fødselsvej		1	
DO715A;Fødselslæsion i urinrøret		1	
DO715B;Fødselslæsion i urinblæren		1	
DO715C;Fødselslæsion med perforation af bækkenorgan		1	
DO715D;Fødselslæsion med ruptur af bækkenorgan		1	

Supplement 2 Nielsen et al. Mortality after paediatric emergency calls for patients with and without pre-existing comorbidity (2023)

DO716;Fødselslæsion i bækkenets led eller ligamenter		1	
DO716A;Fødselslæsion i halebenet		1	
DO716B;Fødselslæsion i bækkenets led		1	
DO716C;Fødselslæsion i bækkenets ligamenter		1	
DO716D;Fødselslæsion med bækkensymfysiolyse		1	
DO717;Hæmatom i fødselsvejen		1	
DO718;Anden fødselslæsion		1	
DO719;Anden fødselslæsion UNS		1	
DO72;Blødning i efterbyrdsperioden		1	
DO720;Fødselsblødning registreret ved afslutning på operations- eller fødestue		1	
DO722;Sen fødselsblødning efter 24 timer efter fødsel		1	
DO73;Fastsiddende moderkage og fosterhinder		1	
DO730;Fastsiddende placenta		1	
DO730A;Placenta accreta UNS		1	
DO730A1;Placenta accreta vera		1	
DO730A2;Placenta increta		1	
DO730A3;Placenta percreta		1	
DO731;Fastsiddende kotyledoner eller fosterhinder		1	
DO731A;Fastsiddende kotyledoner		1	
DO731B;Fastsiddende fosterhinder		1	
DO74;Komplikationer til anæstesi ved fødsel		1	
DO740;Aspirationspneumoni under anæstesi ved fødsel		1	
DO740A;Postaspirationssyndrom efter anæstesi ved fødsel		1	
DO741;Anden lungekomplikation til anæstesi ved fødsel		1	
DO741A;Atelektase under anæstesi ved fødsel		1	
DO742;Hjertekomplikation til anæstesi ved fødsel		1	
DO742A;Hjertesvigt ved anæstesi under fødsel		1	
DO742B;Hjertestop ved anæstesi under fødsel		1	
DO743;Komplikation i centralnervesystemet til anæstesi ved fødsel		1	
DO743A;Hjerneanoksi ved anæstesi under fødsel		1	
DO744;Toksisk reaktion på lokalanæstesi ved fødsel		1	
DO745;Hovedpine efter spinal eller epidural anæstesi ved fødsel		1	

Supplement 2 Nielsen et al. Mortality after paediatric emergency calls for patients with and without pre-existing comorbidity (2023)

DO745A;Hovedpine efter spinal anæstesi ved fødsel		1	
DO745B;Hovedpine efter epidural anæstesi ved fødsel		1	
DO746;Anden komplikation efter spinal eller epidural anæstesi ved fødsel		1	
DO747;Mislykket eller vanskelig trakeal intubation ved fødsel		1	
DO748;Anden komplikation til anæstesi ved fødsel		1	
DO748A;Død under anæstesi ved fødsel		1	
DO749;Komplikation til anæstesi ved fødsel UNS		1	
DO75;Andre fødselskomplikationer IKA		1	
DO750;Udmattelse af moder under fødsel		1	
DO751;Shock under eller efter fødslen		1	
DO752;Temperaturforhøjelse under fødsel IKA		1	
DO753;Anden infektion under fødsel		1	
DO753A;Sepsis under fødsel		1	
DO754;Anden komplikation til obstetrisk indgreb		1	
DO754A;Hjerneanoksi efter kejsersnit		1	
DO754B;Hjerneanoksi efter obstetrisk indgreb UNS		1	
DO754C;Hjertesvigt efter kejsersnit		1	
DO754D;Hjertesvigt efter obstetrisk indgreb UNS		1	
DO754E;Hjertestop efter kejsersnit		1	
DO754F;Hjertestop efter obstetrisk indgreb UNS		1	
DO755;Langvarig fødsel efter hindesprængning		1	
DO756;Langvarig fødsel efter spontan eller ikke specificeret vandafgang		1	
DO756A;Langvarig fødsel efter spontan vandafgang		1	
DO756B;Langvarig fødsel efter vandafgang UNS		1	
DO757;Vaginal fødsel efter tidligere kejsersnit		1	
DO758;Anden fødselskomplikation		1	
DO758A;Truende uterusruptur		1	
DO759;Fødselskomplikation UNS		1	
DO80;Spontan enkeltfødsel		1	
DO800;Spontan enkeltfødsel med hovedpræsentation (findes ikke i SKS)		1	
DO809;Spontan enkeltfødsel		1	
DO81;Instrumentel enkeltfødsel		1	

DO814;Enkeltfødsel med forløsning med vakuumekstraktor (findes ikke i SKS)		1	
DO819;Enkeltfødsel med instrumentel forløsning		1	
DO82;Enkeltfødsel ved kejsersnit		1	
DO820;Enkeltfødsel ved elektivt kejsersnit (findes ikke i SKS)		1	
DO821A;Enkeltfødsel ved akut kejsersnit før fødsel (findes ikke i SKS)		1	
DO821C;Enkeltfødsel ved akut kejsersnit u. fødsel pga. fødselskomplikation (findes ikke i SKS)		1	
DO829;Enkeltfødsel ved kejsersnit		1	
DO83;Andre former for enkeltfødsel		1	
DO836;Enkeltfødsel efter abortprocedure		1	
DO836G;Enkeltfødsel efter abortproc., samråd v. ung alder/umodenhed (findes ikke i SKS)		1	
DO837;Enkeltfødsel efter igangsættelse		1	
DO838;Anden form for enkeltfødsel		1	
DO838A;Enkeltfødsel efter igangsættelse (findes ikke i SKS)		1	
DO839;Enkeltfødsel UNS		1	
DO84;Flerfoldsfødsel		1	
DO840;Spontan flerfoldsfødsel		1	
DO841;Flerfoldsfødsel med instrumentel forløsning		1	
DO842;Flerfoldsfødsel ved kejsersnit		1	
DO842A;Flerfoldsfødsel med vaginal fødsel af A og akut kejsersnit af B		1	
DO846;Flerfoldsfødsel efter abortprocedure		1	
DO847;Flerfoldsfødsel efter igangsættelse		1	
DO848;Anden form for flerfoldsfødsel		1	
DO849;Flerfoldsfødsel UNS		1	
DO85;Barselsfeber		1	
DO859;Sepsis i barselsperioden		1	
DO859A;Endometritis i barselsperioden		1	
DO859B;Peritonitis i barselsperioden		1	
DO86;Andre infektioner i barselsperioden		1	
DO860;Sårinfektion efter obstetrisk indgreb		1	
DO860A;Sårinfektion efter kejsersnit		1	
DO860B;Sårinfektion efter sutur i perineum		1	
DO860C;Overfladisk sårinfektion efter obstetrisk operation UNS		1	

DO860D;Dyb sårinfektion efter obstetrisk operation UNS		1	
DO861;Anden infektion i fødselsvejen efter fødsel		1	
DO861A;Cervicitis i barselsperioden		1	
DO861B;Vaginitis i barselsperioden		1	
DO861C;Sårinfektion i fødselsvejen efter fødsel UNS		1	
DO862;Urinvejsinfektion i barselsperioden		1	
DO862A;Cystitis i barselsperioden		1	
DO862B;Pyelonefritis i barselsperioden		1	
DO862C;Uretritis i barselsperioden		1	
DO863;Anden urogenital infektion i barselsperioden		1	
DO863A;Urogenital infektion i barselsperioden UNS		1	
DO863B;Underlivsinfektion i barselsperioden		1	
DO864;Feber i barselsperioden uden kendt årsag		1	
DO868;Anden infektion i barselsperioden		1	
DO87;Venøse komplikationer og hæmorider under barselsperioden		1	
DO870;Overfladisk tromboflebitis i barselsperioden		1	
DO871;Dyb tromboflebitis i barselsperioden		1	
DO871A;Tromboflebitis i bækkenet i barselsperioden		1	
DO872;Hæmorider i barselsperioden		1	
DO873;Trombose i hjernevener i barselsperioden		1	
DO873A;Thrombosis sinuum venosum cerebri puerperalis		1	
DO878;Anden venøs komplikation i barselsperioden		1	
DO879;Venøs komplikation i barselsperioden UNS		1	
DO88;Obstetrisk emboli		1	
DO880;Obstetrisk luftemboli		1	
DO881;Amnionvæskeemboli		1	
DO882;Obstetrisk emboli forårsaget af blodprop		1	
DO882A;Emboli i barselsperioden		1	
DO882B;Lungeinfarkt i barselsperioden		1	
DO882C;Lungetrombose i barselsperioden		1	
DO882D;Lungeemboli i barselsperioden		1	
DO882E;Lungeemboli opstået under graviditeten		1	

DO883;Obstetrisk septisk emboli		1	
DO888;Anden obstetrisk emboli		1	
DO888A;Obstetrisk fedtemboli		1	
DO89;Komplikationer til anæstesi i barselsperioden		1	
DO890;Lungekomplikation til anæstesi i barselsperioden		1	
DO890A;Aspirationspneumoni efter anæstesi i barselsperioden		1	
DO890B;Atelektase efter anæstesi i barselsperioden		1	
DO890C;Postaspirationssyndrom efter anæstesi i barselsperioden		1	
DO891;Hjertekomplikation til anæstesi i barselsperioden		1	
DO891A;Hjertesvigt ved anæstesi i barselsperioden		1	
DO891B;Hjertestop ved anæstesi i barselsperioden		1	
DO892;Komplikation i centralnervesystemet til anæstesi i barselsperioden		1	
DO892A;Cerebral anoksi ved anæstesi i barselsperioden		1	
DO893;Toksisk reaktion ved lokalanæstesi i barselsperioden		1	
DO894;Hovedpine efter spinal eller epidural anæstesi i barselsperioden		1	
DO895;Anden komplikation til spinal eller epidural anæstesi i barselsperioden		1	
DO896;Vanskelig eller mislykket trakeal intubation i barselsperioden		1	
DO898;Anden komplikation til anæstesi i barselsperioden		1	
DO899;Komplikation til anæstesi i barselsperioden UNS		1	
DO90;Komplikationer i barselsperioden IKA		1	
DO900;Sårruptur efter kejsersnit		1	
DO901;Sårruptur efter sutur af perineum		1	
DO901A;Sårruptur efter episiotomi		1	
DO902;Hæmatom i obstetrisk operationssår		1	
DO903;Kardiomyopati i barselsperioden		1	
DO904;Akut nyresvigt i barselsperioden		1	
DO904A;Hepatorenalt syndrom efter fødslen		1	
DO905;Thyroiditis efter fødslen		1	
DO908;Anden komplikation i barselsperioden		1	
DO908A;Placentapolyp		1	
DO908B;Sårruptur i fødselsvejen UNS		1	
DO908C;Urinretention efter fødsel		1	

DO908D;Retineret placentarvæv eller fosterhinder efter fødsel		1	
DO909;Komplikation i barselsperioden UNS		1	
DO91;Infektioner i brystkirtel og brystvorte i forbindelse med fødslen		1	
DO910;Infektion i brystvorte i forbindelse med fødslen		1	
DO910A;Absces i brystvorte i forbindelse med amning		1	
DO910B;Absces i brystvorte i barselsperioden		1	
DO910C;Absces i brystvorte i graviditeten		1	
DO910D;Infektion i brystvorte i barselsperioden		1	
DO910E;Infektion i brystvorte i forbindelse med amning		1	
DO910F;Infektion i brystvorte i graviditeten		1	
DO911;Absces i bryst i forbindelse med fødslen		1	
DO911A;Absces i areola mammae i barselsperioden		1	
DO911B;Absces i areola mammae i forbindelse med amning		1	
DO911C;Absces i areola mammae i graviditeten		1	
DO911D;Absces i brystkirtel i barselsperioden		1	
DO911E;Absces i brystkirtel i graviditeten		1	
DO911F;Absces i brystkirtel i forbindelse med amning		1	
DO911G;Purulent mastitis i barselsperioden		1	
DO911H;Purulent mastitis i graviditeten		1	
DO911I;Purulent mastitis i forbindelse med amning		1	
DO911J;Flegmone i brystkirtel i barselsperioden		1	
DO911K;Flegmone i brystkirtel i graviditeten		1	
DO911L;Flegmone i brystkirtel i forbindelse med amning		1	
DO912;Ikke-purulent mastitis i forbindelse med fødslen		1	
DO912A;Galactophoritis puerperalis		1	
DO912B;Lymfangitis i bryst i barselsperioden		1	
DO912C;Lymfangitis i bryst i graviditeten		1	
DO912D;Lymfangitis i bryst i forbindelse med amning		1	
DO912E;Mastitis i graviditeten UNS		1	
DO912F;Ikke-purulent mastitis i forbindelse med amning		1	
DO912G;Ikke-purulent mastitis i barselsperioden		1	
DO912H;Ikke-purulent mastitis i graviditeten		1	

Supplement 2 Nielsen et al. Mortality after paediatric emergency calls for patients with and without pre-existing comorbidity (2023)

DO912I;Mastitis i barselsperioden UNS		1		
DO92;Andre forstyrrelser i brystkirtel og amning i barselsperioden		1		
DO920;Inverteret brystvorte efter fødslen		1		
DO920A;Kompliceret amning ved inverteret brystvorte		1		
DO921;Fissur i brystvorte efter fødslen		1		
DO922;Anden eller ikke specificeret sygdom i bryst i barselsperioden		1		
DO923;Udeblevet mælkeproduktion		1		
DO924;Nedsat mælkeproduktion		1		
DO925;Ophævet mælkeproduktion		1		
DO926;Galaktoré i barselsperioden		1		
DO927;Anden eller ikke nærmere specificeret forstyrrelse i amning		1		
DO927A;Brystatrofi i barselsperioden		1		
DO927B;Galaktocele i barselsperioden		1		
DO927C;Laktationsinsufficiens		1		
DO927D;Galaktostase		1		
DO98;Infektioner og parasitære sygdomme hos moderen, som klassificeres i andre kapitler, men som komplicerer graviditet		1		
DO980;Tuberkulose som komplicerer graviditet, fødsel eller barselsperiode		1		
DO981;Syfilis som komplicerer graviditet, fødsel eller barselsperiode		1		
DO982;Gonore som komplicerer graviditet, fødsel eller barselsperiode		1		
DO983;Anden venerisk sygdom som komplicerer graviditet, fødsel eller barselsperiode		1		
DO984;Viral hepatitis som komplicerer graviditet, fødsel eller barselsperiode		1		
DO985;Anden viral sygdom som komplicerer graviditet, fødsel eller barselsperiode		1		
DO985A;Influenza som komplicerer graviditet, fødsel eller barselsperiode		1		
DO986;Protozosygdom som komplicerer graviditet, fødsel eller barselsperiode		1		
DO987;HIV sygdom som komplicerer graviditet, fødsel eller barselsperiode		1		
DO988;Anden infektiøs eller parasitær sygdom som komplicerer graviditet, fødsel eller barselsperiode		1		
DO988A;Cytomegalovirusinfektion som komplicerer graviditet, fødsel eller barselsperiode		1		
DO988B;Herpes simplex-infektion som komplicerer graviditet, fødsel eller barselsperiode		1		
DO988C;Listeriose som komplicerer graviditet, fødsel eller barselsperiode		1		
DO988D;Parvovirus B19-infektion som komplicerer graviditet, fødsel eller barselsperiode		1		
DO988E;Skoldkopper som komplicerer graviditet, fødsel eller barselsperiode		1		
DO988F;Q-feber som komplicerer graviditet, fødsel eller barselsperiode		1		

DO988G;Toksoplasmose som komplicerer graviditet, fødsel eller barselsperiode	1		
DO988H;MRSA som komplicerer graviditet, fødsel eller barselsperiode	1		
DO989;Infektiøs eller parasitær sygdom komplicerer graviditet, fødsel eller barselsperiode UNS	1		
DO99;Andre sygdomme hos moderen, som klassificeres i andre kapitler, men som komplicerer graviditet, fødsel og barsels	1		
DO990;Anæmi som komplicerer graviditet, fødsel eller barselsperiode	1		
DO990A;Anæmi som komplicerer barselsperioden	1		
DO990B;Anæmi som komplicerer graviditeten	1		
DO990C;Anæmi som komplicerer fødslen	1		
DO991;Anden sygdom i blod, bloddannende organer eller visse immunsygdomme som komplicerer graviditet, fødsel eller ba	1		
DO991B;Trombocytopeni som komplicerer graviditet, fødsel eller barselsperiode	1		
DO991C;Reumatoid artritis som komplicerer graviditet, fødsel eller barselsperiode	1		
DO991D;Systemisk lupus erythematosus som komplicerer graviditet, fødsel eller barselsperiode	1		
DO992;Endokrin, ernæringsbetinget eller metabolisk sygdom som komplicerer graviditet, fødsel eller barselsperiode	1		
DO992B;Hypothyroidisme som komplicerer graviditet, fødsel eller barselsperiode	1		
DO992C;Thyrotoksikose som komplicerer graviditet, fødsel eller barselsperiode	1		
DO993;Psykisk eller neurologisk sygdom som komplicerer graviditet, fødsel eller barselsperiode	1		
DO993A;Neurologisk sygdom som komplicerer graviditet, fødsel eller barselsperiode	1		
DO993A1;Epilepsi som komplicerer graviditet, fødsel eller barselsperiode	1		
DO993A2;Migræne som komplicerer graviditet, fødsel eller barselsperiode	1		
DO993A3;Dissemineret sklerose som komplicerer graviditet, fødsel eller barselsperiode	1		
DO993B;Psykisk sygdom som komplicerer graviditet, fødsel eller barselsperiode	1		
DO993B1;Skizofreni som komplicerer graviditet, fødsel eller barselsperiode	1		
DO993B2;Depression som komplicerer graviditet, fødsel eller barselsperiode	1		
DO993B3;Angsttilstand som komplicerer graviditet, fødsel eller barselsperiode	1		
DO993B4;Spiseforstyrrelse som komplicerer graviditet, fødsel eller barselsperiode	1		
DO993B5;Hyperkinetisk forstyrrelse som komplicerer graviditet, fødsel eller barselsperiode	1		
DO994;Kredsløbssygdom som komplicerer graviditet, fødsel eller barselsperiode	1		
DO995;Luftvejssygdom som komplicerer graviditet, fødsel eller barselsperiode	1		
DO995A;Astma som komplicerer graviditet, fødsel eller barselsperiode	1		
DO995B;Bronkitis som komplicerer graviditet, fødsel eller barselsperiode	1		
DO996;Sygdom i fordøjelseskanal som komplicerer graviditet, fødsel eller barselsperiode	1		
DO996A;Crohns sygdom som komplicerer graviditet, fødsel eller barselsperiode	1		

Supplement 2 Nielsen et al. Mortality after paediatric emergency calls for patients with and without pre-existing comorbidity (2023)

DO996B;Ulcerøs colitis som komplicerer graviditet, fødsel eller barselsperiode		1	
DO996C;Akut pseudoobstruktion af tyktarm som komplicerer graviditet, fødsel eller barselsperiode		1	
DO997;Sygdom i hud eller underhud som komplicerer graviditet, fødsel eller barselsperiode		1	
DO997A;Psoriasis som komplicerer graviditet, fødsel eller barselsperiode		1	
DO998;Anden sygdom som komplicerer graviditet, fødsel eller barselsperiode		1	
DO998A;Hydronefrose under graviditet		1	
DO998B;Spontan hæmoperitoneum som komplicerer graviditet, fødsel eller barselsperiode		1	
DP00;Følger for nyfødt forårsaget af forhold hos moder uden forbindelse med det aktuelle graviditet		1	
DP000;Forhøjet blodtryk hos moder med følger for nyfødt		1	
DP001;Urinvejssygdom hos moder med følger for nyfødt		1	
DP001A;Nefropati hos moder med følger for nyfødt		1	
DP002;Infektiøs eller parasitær sygdom hos moder med følger for nyfødt		1	
DP003;Anden sygdom i luftveje eller kredsløb hos moder med følger for nyfødt		1	
DP004;Ernæringssygdom hos moder med følger for nyfødt		1	
DP005;Moders tilskadekomst med følger for nyfødt		1	
DP006;Kirurgisk indgreb på moder med følger for nyfødt		1	
DP007;Andet indgreb eller behandling på moder med følger for nyfødt		1	
DP008;Anden tilstand hos moder med følger for nyfødt		1	
DP009;Sygdom UNS hos moder med følger for nyfødt		1	
DP01;Følger for nyfødt som følge af komplikationer til det aktuelle graviditet		1	
DP010;Livmoderhalsinsufficiens med følger for nyfødt		1	
DP011;For tidlig vandafgang med følger for nyfødt		1	
DP012;Oligohydramnion med følger for nyfødt		1	
DP013;Hydramnion med følger for nyfødt		1	
DP014;Ektopisk graviditet med følger for nyfødt		1	
DP015;Flerfoldsgraviditet med følger for nyfødt		1	
DP015A;Tvillingegraviditet med følger for nyfødt		1	
DP016;Moders død med følger for nyfødt		1	
DP017;Abnorm fosterstilling med følger for nyfødt		1	
DP018;Anden graviditetskomplikation med følger for nyfødt		1	
DP019;Graviditetskomplikation UNS med følger for nyfødt		1	
DP02;Følger for nyfødt forårsaget af abnormitet ved placenta, navlesnor og fosterhinder		1	

DP020;Forliggende placenta med følger for nyfødt		1	
DP021;Anden blødning med følger for nyfødt		1	
DP021D;Beskadigelse af placenta ved indgreb i graviditeten med følger for nyfødt		1	
DP022;Anden eller ikke specificeret abnormitet ved placenta med følger for nyfødt		1	
DP022A;Placentainfarkt med følger for nyfødt		1	
DP022B;Placentainsufficiens med følger for nyfødt		1	
DP023;Placentatransfusionssyndrom med følger for nyfødt		1	
DP024;Fremfald af navlesnor med følger for nyfødt		1	
DP025;Anden afklemning af navlesnor med følger for nyfødt		1	
DP025A;Navlesnorsstrangulation med følger for nyfødt		1	
DP025B;Navlesnorstorsion med følger for nyfødt		1	
DP026;Anden abnormitet i navlesnor med følger for nyfødt		1	
DP027;Chorioamnionitis med følger for nyfødt		1	
DP028;Anden abnormitet i chorion eller amnion med følger for nyfødt		1	
DP028A;Anden abnormitet i amnion med følger for nyfødt		1	
DP028B;Anden abnormitet i chorion med følger for nyfødt		1	
DP029;Abnormitet i chorion eller amnion UNS med følger for nyfødt		1	
DP03;Følger for nyfødt ved andre fødselskomplikationer		1	
DP030;Underkropsfødsel med følger for nyfødt		1	
DP031;Fødsel med anden abnorm fosterstilling eller mekanisk misforhold med følger for nyfødt		1	
DP031A;Fødsel med anden abnorm fosterstilling med følger for nyfødt		1	
DP031B;Fødsel med anden mekanisk misforhold med følger for nyfødt		1	
DP032;Tangforløsning med følger for nyfødt		1	
DP033;Forløsning med vakuumekstraktor med følger for nyfødt		1	
DP034;Kejsersnit med følger for nyfødt		1	
DP035;Styrtfødsel med følger for nyfødt		1	
DP036;Abnorme veer med følger for nyfødt		1	
DP036A;Hypertone veer med følger for nyfødt		1	
DP036B;Vesvækkelse med følger for nyfødt		1	
DP038;Anden fødselskomplikation med følger for nyfødt		1	
DP039;Fødselskomplikation UNS med følger for nyfødt		1	
DP04;Følger for nyfødt ved toksiske stoffer overført gennem placenta eller modermælk		1	

Supplement 2 Nielsen et al. Mortality after paediatric emergency calls for patients with and without pre-existing comorbidity (2023)

DP040;Anæstesi eller analgesi givet til moder i graviditet, fødsel eller barselsperiode med følger for nyfødt		1	
DP040K;Abstinenssymptomer hos nyfødt forårsaget af prænatal eksponering af anæstesi/analgetika		1	
DP041;Medicinsk behandling af moder med følger for nyfødt		1	
DP042;Tobaksbrug hos moder med følger for nyfødt		1	
DP043;Alkoholbrug hos moder med følger for nyfødt		1	
DP044;Stofmisbrug hos moder med følger for nyfødt		1	
DP045;Fødemiddel påvirkning af moder med følger for nyfødt		1	
DP046;Kemisk miljøpåvirkning af moder med følger for nyfødt		1	
DP048;Anden skadelig påvirkning af moder med følger for nyfødt		1	
DP049;Skadelig påvirkning af moder UNS med følger for nyfødt		1	
DP05;Langsom vækst og underernæring af fosteret		1	
DP050;Lav vægt i forhold til gestationsalderen		1	
DP051;Lille i forhold til gestationsalderen		1	
DP052;Dysmaturitet		1	
DP059;Langsom fostervækst UNS		1	
DP07;Tilstande hos nyfødt ved tidlig fødsel og lav vægt IKA		1	
DP070;Ekstremt lav fødselsvægt		1	
DP071;Lav fødselsvægt		1	
DP071A;Lav fødselsvægt (1000-1499 gram)		1	
DP071B;Lav fødselsvægt (1500-2499 gram)		1	
DP072;Immaturitet		1	
DP073;Præmaturitet		1	
DP08;Tilstande hos nyfødt ved forlænget graviditet og høj fødselsvægt		1	
DP080;Meget høj fødselsvægt		1	
DP081;Barn med høj fødselsvægt for gestationsalder af anden årsag		1	
DP082;Postmaturitet		1	
DP10;Fødselslæsioner med intrakranielle skader og blødninger		1	
DP100;Subdural blødning hos nyfødt forårsaget af fødselstraume		1	
DP100A;Subduralt hygrom hos nyfødt forårsaget af fødselstraume		1	
DP101;Hjerneblødning hos nyfødt forårsaget af fødselstraume		1	
DP102;Intraventrikulær blødning hos nyfødt forårsaget af fødselstraume		1	
DP103;Subaraknoidal blødning hos nyfødt forårsaget af fødselstraume		1	

DP104;Ruptur af tentorium cerebelli hos nyfødt forårsaget af fødselstraume		1	
DP108;Anden intrakraniel læsion forårsaget af fødselstraume		1	
DP109;Intrakraniel læsion eller blødning UNS forårsaget af fødselstraume		1	
DP11;Andre fødselslæsioner i centralnervesystemet		1	
DP110;Hjerneødem hos nyfødt forårsaget af fødselstraume		1	
DP111;Anden hjerneskade hos nyfødt forårsaget af fødselstraume		1	
DP112;Hjerneskade UNS hos nyfødt forårsaget af fødselstraume		1	
DP113;Læsion af nervus facialis hos nyfødt forårsaget af fødselstraume		1	
DP114;Læsion af anden hjernenerve hos nyfødt forårsaget af fødselstraume		1	
DP115;Fødselslæsion af rygsøjlen eller rygmarven		1	
DP115A;Fødselslæsion af rygsøjlen		1	
DP115B;Blødning i rygmarven hos nyfødt forårsaget af fødselstraume		1	
DP115C;Fødselslæsion af rygmarven		1	
DP119;Fødselslæsion i centralnervesystemet UNS		1	
DP12;Fødselslæsioner af skalp		1	
DP120;Kefalhæmatom hos nyfødt forårsaget af fødselstraume		1	
DP121;Fødselssvulst		1	
DP122;Epikraniel subaponeurotisk blødning forårsaget af fødselstraume		1	
DP123;Overfladisk læsion på hovedet forårsaget af fødselstraume		1	
DP124;Sår på skalp forårsaget af prøvetagning eller monitorering		1	
DP128;Anden fødselslæsion af skalp		1	
DP129;Fødselslæsion af skalp UNS		1	
DP13;Fødselslæsioner af skelettet		1	
DP130;Kraniefraktur forårsaget af fødselstraume		1	
DP131;Anden fødselslæsion af kraniet		1	
DP132;Femurfraktur forårsaget af fødselstraume		1	
DP133;Fødselslæsion af anden rørknogle		1	
DP134;Claviculafraktur forårsaget af fødselstraume		1	
DP138;Anden fødselslæsion af skelettet		1	
DP139;Fødselslæsion af skelettet UNS		1	
DP14;Fødselslæsioner af det perifere nervesystem		1	
DP140;Erb-Duchennes paralyse		1	

Supplement 2 Nielsen et al. Mortality after paediatric emergency calls for patients with and without pre-existing comorbidity (2023)

DP141;Klumpkes paralyse		1	
DP142;Paralyse af nervus phrenicus forårsaget af fødselstraume		1	
DP143;Anden fødselslæsion af plexus brachialis		1	
DP148;Anden fødselslæsion af det perifere nervesystem		1	
DP149;Fødselslæsion af det perifere nervesystem UNS		1	
DP15;Andre fødselslæsioner		1	
DP150;Fødselslæsion af leveren		1	
DP151;Fødselslæsion af milten		1	
DP152;Fødselslæsion af musculus sternocleidomastideus		1	
DP153;Fødselslæsion af øje		1	
DP153A;Traumatisk glaukom hos nyfødt forårsaget af fødselstraume		1	
DP153B;Subkonjunktivalt hæmatom hos nyfødt forårsaget af fødselstraume		1	
DP153C;Luksation af øjeæble hos nyfødt forårsaget af fødselstraume		1	
DP154;Fødselslæsion af ansigtet		1	
DP154A;Luksation af næseskillevæggen hos nyfødt forårsaget af fødselstraume		1	
DP155;Fødselslæsion af ydre kønsdele		1	
DP156;Subkutan fedtnekrose hos nyfødt forårsaget af fødselstraume		1	
DP158;Anden fødselslæsion		1	
DP158A;Torticollis hos nyfødt forårsaget af fødselstraume		1	
DP159;Fødselslæsion af nyfødt UNS		1	
DP20;Iltmangel hos foster		1	
DP200;Intrauterin hypoxi observeret før veernes begyndelse		1	
DP200A;Fosteracidose observeret før veernes begyndelse		1	
DP200B;Fosterbradykardi observeret før veernes begyndelse		1	
DP200C;Føtalt distress-syndrom observeret før veernes begyndelse		1	
DP200D;Mekoniumafgang intrauterint observeret før veernes begyndelse		1	
DP200E;Fostertakykardi observeret før veernes begyndelse		1	
DP201;Intrauterin hypoxi observeret efter veernes begyndelse		1	
DP201A;Fosteracidose observeret efter veernes begyndelse		1	
DP201B;Fosterbradykardi observeret efter veernes begyndelse		1	
DP201C;Føtalt distress-syndrom observeret efter veernes begyndelse		1	
DP201D;Mekoniumafgang intrauterint observeret efter veernes begyndelse		1	

DP201E;Fostertakykardi observeret efter veernes begyndelse		1	
DP209;Intrauterin hypoxi UNS		1	
DP21;Iltmangel ved fødsel		1	
DP210;Svær neonatal asfyksi		1	
DP210A;Svær neonatal asfyksi med Apgar-score 0 ved 1 min		1	
DP210B;Svær neonatal asfyksi med Apgar-score 1 ved 1 min		1	
DP210C;Svær neonatal asfyksi med Apgar-score 2 ved 1 min		1	
DP210D;Svær neonatal asfyksi med Apgar-score 3 ved 1 min		1	
DP211;Let neonatal asfyksi		1	
DP211A;Let neonatal asfyksi med Apgar-score 4 ved 1 min		1	
DP211B;Let neonatal asfyksi med Apgar-score 5 ved 1 min		1	
DP211C;Let neonatal asfyksi med Apgar-score 6 ved 1 min		1	
DP211D;Let neonatal asfyksi med Apgar-score 7 ved 1 min		1	
DP219;Neonatal asfyksi UNS		1	
DP22;Respiratorisk distress hos nyfødt		1	
DP220;Idiopatisk respiratorisk distress-syndrom hos nyfødt		1	
DP220A;Hyaline membraner		1	
DP221;Transitorisk takypnoe hos nyfødt		1	
DP228;Anden respiratorisk distress hos nyfødt		1	
DP229;Respiratorisk distress hos nyfødt UNS		1	
DP23;Medfødt lungebetændelse		1	
DP230;Medfødt viruspneumoni		1	
DP231;Medfødt klamydiapneumoni		1	
DP232;Medfødt stafylokokpneumoni		1	
DP233;Medfødt streptokok gruppe B pneumoni		1	
DP234;Medfødt pneumoni forårsaget af Escherichia coli		1	
DP235;Medfødt pneumoni forårsaget af Pseudomonas		1	
DP236;Anden bakteriel medfødt pneumoni		1	
DP236A;Medfødt pneumoni forårsaget af Streptococcus pneumoniae		1	
DP236B;Medfødt pneumoni forårsaget af Haemophilus influenzae		1	
DP236C;Medfødt pneumoni forårsaget af Klebsiella pneumoniae		1	
DP236D;Medfødt pneumoni forårsaget af Mycoplasma		1	

Supplement 2 Nielsen et al. Mortality after paediatric emergency calls for patients with and without pre-existing comorbidity (2023)

DP238;Anden medfødt infektiøs pneumoni		1	
DP239;Medfødt pneumoni UNS		1	
DP24;Tilstande hos nyfødt forårsaget af aspiration		1	
DP240;Aspiration af mekonium hos nyfødt		1	
DP241;Aspiration af amnionvæske hos nyfødt		1	
DP242;Aspiration af blod hos nyfødt		1	
DP243;Aspiration af mælk og opgylpet maveindhold hos nyfødt		1	
DP248;Anden aspiration hos nyfødt		1	
DP249;Aspiration hos nyfødt UNS		1	
DP25;Interstitielt emfysem og beslægtede tilstande opstået i perinatalperioden		1	
DP250;Interstitielt emfysem opstået i perinatalperioden		1	
DP251;Pneumothorax opstået i perinatalperioden		1	
DP252;Pneumomediastinum opstået i perinatalperioden		1	
DP253;Pneumoperikardium opstået i perinatalperioden		1	
DP258;Anden tilstand forbundet med interstitielt emfysem i perinatalperioden		1	
DP26;Blødning i lungerne opstået i perinatalperioden		1	
DP260;Trakeobronkial blødning opstået i perinatalperioden		1	
DP261;Kraftig lungeblødning opstået i perinatalperioden		1	
DP268;Anden form for perinatal lungeblødning		1	
DP269;Perinatal lungeblødning UNS		1	
DP27;Kroniske luftvejssygdomme opstået i perinatalperioden		1	
DP270;Wilson-Mikitys syndrom			2
DP271;Bronkopulmonal dysplasi opstået i perinatalperioden		1	
DP271A;Bronkopulmonal dysplasi opstået i perinatalperioden, moderat grad		1	
DP271B;Bronkopulmonal dysplasi opstået i perinatalperioden, svær grad		1	
DP278;Anden kronisk luftvejssygdom opstået i perinatalperioden		1	
DP278A;Respiratorlunge opstået i perinatalperioden		1	
DP278B;Medfødt lungefibrose		1	
DP279;Kronisk luftvejssygdom opstået i perinatalperioden UNS		1	
DP28;Andre luftvejssygdomme opstået i perinatalperioden		1	
DP280;Primær atelektase hos nyfødt		1	
DP280A;Lungehypoplasi ved for tidlig fødsel		1	

Supplement 2 Nielsen et al. Mortality after paediatric emergency calls for patients with and without pre-existing comorbidity (2023)

DP280B;Lungeimmaturitet hos nyfødt UNS		1	
DP281;Anden atelektase hos nyfødt		1	
DP281A;Medfødt partiel atelektase hos nyfødt		1	
DP281B;Medfødt sekundær atelektase hos nyfødt		1	
DP281C;Medfødt atelektase hos nyfødt UNS		1	
DP282;Paroksysmatisk cyanose hos nyfødt		1	
DP283;Primær central søvnapnø hos nyfødt		1	
DP284;Anden form for apnø hos nyfødt		1	
DP285;Lungeinsufficiens hos nyfødt		1	
DP288;Anden luftvejssygdom hos nyfødt		1	
DP288A;Snue hos nyfødt		1	
DP288B;Medfødt laryngeal stridor		1	
DP289;Perinatal lungesygdom UNS		1	
DP29;Kredsløbssygdomme opstået i perinatalperioden		1	
DP290;Hjertesvigt hos nyfødt		1	
DP291;Hjertearytmi hos nyfødt		1	
DP291A;Bradykardi hos nyfødt (primær)(idiopatisk)		1	
DP292;Hypertension hos nyfødt		1	
DP293;Persisterende føtalt kredsløb			2
DP293A;Primær pulmonal hypertension hos nyfødt			2
DP293B;Forsinket lukning af ductus arteriosus		1	
DP294;Transitorisk myokardieiskæmi hos nyfødt		1	
DP298;Anden kredsløbssygdom opstået i perinatalperioden		1	
DP299;Perinatal kredsløbssygdom UNS		1	
DP35;Medfødt virusinfektion		1	
DP350;Medfødt rubellasyndrom		1	
DP351;Medfødt cytomegalovirusinfektion		1	
DP352;Medfødt herpes simplex infektion		1	
DP352A;Medfødt herpes encephalitis		1	
DP353;Medfødt virushepatitis		1	
DP354;Medfødt Zika virussygdom		1	
DP358;Anden medfødt virusinfektion		1	

Supplement 2 Nielsen et al. Mortality after paediatric emergency calls for patients with and without pre-existing comorbidity (2023)

DP358A;Medfødt varicella infektion		1		
DP359;Medfødt virusinfektion UNS		1		
DP36;Bakteriel sepsis hos nyfødt		1		
DP360;Sepsis hos nyfødt forårsaget af gruppe B streptokokker		1		
DP361;Sepsis hos nyfødt forårsaget af streptokokker UNS		1		
DP362;Sepsis hos nyfødt forårsaget af Staphylococcus aureus		1		
DP363;Sepsis hos nyfødt forårsaget af stafylokokker UNS		1		
DP364;Sepsis hos nyfødt forårsaget af Escherichia coli		1		
DP365;Sepsis hos nyfødt forårsaget af anaerobe bakterier		1		
DP368;Sepsis hos nyfødt forårsaget af anden bakterie		1		
DP369;Bakteriel sepsis hos nyfødt UNS		1		
DP37;Andre medfødte infektiøse og parasitære sygdomme		1		
DP370;Medfødt tuberkulose		1		
DP371;Medfødt toksoplasmose		1		
DP371A;Hydrocefalus ved medfødt toksoplasmose		1		
DP372;Dissemineret listeriose hos nyfødt		1		
DP373;Medfødt malaria falciparum		1		
DP374;Anden form for medfødt malaria		1		
DP375;Candidiasis hos nyfødt		1		
DP378;Anden medfødt infektiøs eller parasitær sygdom		1		
DP379;Medfødt infektiøs eller parasitær sygdom UNS		1		
DP38;Betændelse i navle hos nyfødt		1		
DP389;Navlebetændelse hos nyfødt med eller uden let blødning		1		
DP389B;Omphalitis neonati uden blødning (findes ikke i SKS)		1		
DP389E;Navlegranulom		1		
DP39;Andre infektioner specifikke for perinatalperioden		1		
DP390;Infektiøs mastitis hos nyfødt		1		
DP390A;Mamma-absces hos nyfødt		1		
DP391;Konjunktivitis eller dakryocystitis hos nyfødt		1		
DP391A;Konjunktivitis hos nyfødt		1		
DP391B;Dakryocystitis hos nyfødt		1		
DP391C;Oftalmi hos nyfødt UNS		1		

DP392;Intra-amniotisk infektion af foster IKA		1	
DP393;Urinvejsinfektion hos nyfødt		1	
DP394;Hudinfektion hos nyfødt		1	
DP398;Anden infektion specifik for perinatalperioden		1	
DP399;Infektion specifik for perinatalperioden UNS		1	
DP50;Blodtab opstået før eller under fødsel		1	
DP500;Blodtab hos foster ved blødning fra forliggende fosterhindekar		1	
DP501;Blodtab hos foster ved blødning fra ruptur af navlesnoren		1	
DP502;Blodtab hos foster ved blødning fra placenta		1	
DP503;Blodtab hos foster ved blødning til tvilling ved flerfoldsgraviditet		1	
DP504;Blodtab hos foster ved blødning til moderens cirkulation		1	
DP505;Blodtab hos foster ved blødning fra tvillings navlesnor		1	
DP508;Andet blodtab hos foster		1	
DP509;Blodtab hos foster UNS		1	
DP51;Navleblødning hos nyfødt		1	
DP510;Kraftig navleblødning hos nyfødt		1	
DP518;Anden form for navleblødning hos nyfødt		1	
DP518A;Navleblødning hos nyfødt ved suturskred		1	
DP519;Navleblødning hos nyfødt UNS		1	
DP52;Ikke-traumatisk hjerneblødning hos nyfødt		1	
DP520;Ikke-traumatisk intraventrikulær hjerneblødning hos nyfødt, grad 1		1	
DP521;Ikke-traumatisk intraventrikulær hjerneblødning hos nyfødt, grad 2		1	
DP522;Ikke-traumatisk intraventrikulær hjerneblødning hos nyfødt, grad 3		1	
DP523;Ikke-traumatisk intraventrikulær hjerneblødning hos nyfødt UNS		1	
DP524;Ikke-traumatisk intracerebral hjerneblødning hos nyfødt		1	
DP525;Ikke-traumatisk subaraknoidal blødning hos nyfødt		1	
DP526;Ikke-traumatisk blødning i lillehjernen eller fossa posterior hos nyfødt		1	
DP528;Anden form for ikke-traumatisk intrakraniel blødning hos nyfødt		1	
DP529;Ikke-traumatisk intrakraniel blødning hos nyfødt UNS		1	
DP53;Blødningstendens hos nyfødt		1	
DP539;Hæmoragisk sygdom hos nyfødt		1	
DP539A;Anæmi hos nyfødt forårsaget af K-vitaminmangel		1	

Supplement 2 Nielsen et al. Mortality after paediatric emergency calls for patients with and without pre-existing comorbidity (2023)

DP54;Andre former for blødning hos nyfødt		1	
DP540;Hæmatemese hos nyfødt		1	
DP541;Melæna hos nyfødt		1	
DP542;Rektalblødning hos nyfødt		1	
DP543;Anden blødning i mave-tarm-kanalen hos nyfødt		1	
DP544;Binyreblødning hos nyfødt		1	
DP545;Blødning i huden hos nyfødt		1	
DP545A;Petecchiae neonati		1	
DP545B;Purpura neonati		1	
DP546;Vaginal blødning hos nyfødt		1	
DP548;Anden blødning hos nyfødt		1	
DP549;Blødning hos nyfødt UNS		1	
DP55;Hæmolytisk sygdom hos nyfødt		1	
DP550;Rhesus-immunisering hos nyfødt		1	
DP551;AB0-immunisering hos nyfødt		1	
DP558;Anden hæmolytisk sygdom hos nyfødt		1	
DP559;Hæmolytisk sygdom hos nyfødt UNS		1	
DP56;Universel væskeansamling hos foster ved hæmolytisk sygdom		1	
DP560;Hydrops foetalis ved isoimmunisering		1	
DP569;Hydrops foetalis hos foster ved anden eller ikke specificeret hæmolytisk sygdom		1	
DP57;Kernikterus		1	
DP570;Kernikterus ved isoimmunisering		1	
DP578;Anden form for kernikterus		1	
DP579;Kernikterus UNS		1	
DP58;Gulsot hos nyfødt ved anden svær hæmolyse		1	
DP580;Ikterus hos nyfødt forårsaget af traume		1	
DP581;Ikterus hos nyfødt forårsaget af blødning		1	
DP582;Ikterus hos nyfødt forårsaget af infektion		1	
DP583;Ikterus hos nyfødt forårsaget af polycytæmi		1	
DP584;Ikterus hos nyfødt forårsaget af lægemiddel eller toksin ved indgift eller ved overførsel fra moder		1	
DP585;Ikterus hos nyfødt ved synkning af blod fra moder		1	
DP588;Ikterus hos nyfødt ved anden svær hæmolyse		1	

Supplement 2 Nielsen et al. Mortality after paediatric emergency calls for patients with and without pre-existing comorbidity (2023)

DP589;Ikterus hos nyfødt ved svær hæmolyse UNS		1	
DP59;Gulsot hos nyfødt af andre og ikke specificerede årsager		1	
DP590;Ikterus hos nyfødt ved præmaturitet		1	
DP591;Galdepropsyndrom		1	
DP592;Ikterus hos nyfødt ved anden eller ikke specificeret levercelleskade		1	
DP593;Ikterus hos nyfødt forårsaget af inhibitor fra modermælk		1	
DP598;Ikterus hos nyfødt af anden årsag		1	
DP599;Ikterus hos nyfødt UNS		1	
DP60;Udbredt intravaskulær koagulation hos nyfødt		1	
DP609;Dissemineret intravaskulær koagulation hos nyfødt		1	
DP61;Andre perinatale blodsygdomme		1	
DP610;Forbigående trombocytopeni hos nyfødt		1	
DP610B;Forbigående trombocytopeni hos nyfødt ved isoimmunisering		1	
DP610C;Forbigående trombocytopeni hos nyfødt ved udskiftningstransfusion		1	
DP610D;Forbigående trombocytopeni hos nyfødt ved idiopatisk trombocytopeni hos moder		1	
DP611;Polycytæmi hos nyfødt		1	
DP612;Anæmi ved præmaturitet		1	
DP613;Medfødt anæmi ved blodtab i fostertilstanden		1	
DP614;Anæmi hos nyfødt IKA		1	
DP614A;Anæmi hos nyfødt UNS		1	
DP615;Forbigående neutropeni hos nyfødt		1	
DP616;Anden forbigående koagulationsforstyrrelse hos nyfødt		1	
DP618;Anden perinatal blodsygdom		1	
DP619;Perinatal blodsygdom UNS		1	
DP70;Forbigående forstyrrelse i kulhydratomsætningen hos nyfødt		1	
DP700;Syndrom hos barn af moder med gestationel diabetes		1	
DP701;Syndrom hos barn af moder med diabetes mellitus		1	
DP702;Diabetes mellitus hos nyfødt		1	
DP703;Hypoglykæmi hos nyfødt som følge af behandling		1	
DP704;Anden form for hypoglykæmi hos nyfødt		1	
DP708;Anden form for forbigående forstyrrelse i kulhydratomsætningen hos nyfødt		1	
DP709;Forbigående forstyrrelse i kulhydratomsætningen hos nyfødt UNS		1	

Supplement 2 Nielsen et al. Mortality after paediatric emergency calls for patients with and without pre-existing comorbidity (2023)

DP71;Forbigående forstyrrelse i kalcium- og magnesiumomsætningen hos nyfødt		1	
DP710;Komælkshypokalcæmi hos nyfødt		1	
DP711;Anden form for hypokalcæmi hos nyfødt		1	
DP712;Hypomagnesiæmi hos nyfødt		1	
DP713;Tetani hos nyfødt uden kendt hypokalcæmi eller hypomagnesiæmi		1	
DP713A;Tetani hos nyfødt UNS		1	
DP714;Forbigående hypoparathyroidisme hos nyfødt		1	
DP718;Anden forbigående forstyrrelse i kalcium- eller magnesiumomsætningen hos nyfødt		1	
DP718A;Hyperkalcæmi hos nyfødt		1	
DP718B;Hypermagnesiæmi hos nyfødt		1	
DP719;Forbigående forstyrrelse i kalcium- eller magnesiumomsætningen hos nyfødt UNS		1	
DP72;Andre forbigående hormonforstyrrelser hos nyfødt		1	
DP720;Struma hos nyfødt IKA		1	
DP720A;Forbigående struma hos nyfødt med normal funktion		1	
DP720B;Struma hos nyfødt UNS		1	
DP721;Forbigående thyrotoksikose hos nyfødt		1	
DP721A;Thyrotoksikose hos nyfødt UNS		1	
DP722;Anden forbigående forstyrrelse i skjoldbrukskirtlens funktion hos nyfødt IKA		1	
DP722A;Forbigående myxødem hos nyfødt		1	
DP728;Anden forbigående hormonforstyrrelser hos nyfødt		1	
DP729;Forbigående hormonforstyrrelse hos nyfødt UNS		1	
DP74;Andre forbigående elektrolyt- og stofskifteforstyrrelser hos nyfødt		1	
DP740;Sen metabolisk acidose hos nyfødt		1	
DP741;Dehydrering hos nyfødt		1	
DP742;Forstyrrelser i natriumbalancen hos nyfødt		1	
DP742A;Hypernatriæmi hos nyfødt		1	
DP742B;Hyponatriæmi hos nyfødt		1	
DP743;Forstyrrelser i kaliumbalancen hos nyfødt		1	
DP743A;Hyperkaliæmi hos nyfødt		1	
DP743B;Hypokaliæmi hos nyfødt		1	
DP744;Anden forbigående elektrolytforstyrrelse hos nyfødt		1	
DP744A;Hyperkloræmi hos nyfødt		1	

Supplement 2 Nielsen et al. Mortality after paediatric emergency calls for patients with and without pre-existing comorbidity (2023)

DP744B;Hypokloræmi hos nyfødt		1		
DP745;Forbigående tyrosinæmi hos nyfødt		1		
DP748;Anden forbigående metabolisk forstyrrelse hos nyfødt		1		
DP749;Forbigående metabolisk forstyrrelse hos nyfødt UNS		1		
DP75;Mekoniumileus ved cystisk fibrose		1		
DP759;Mekoniumileus ved cystisk fibrose		1		
DP76;Anden form for tarmobstruktion hos nyfødt		1		
DP760;Mekoniumprop		1		
DP761;Forbigående ileus hos nyfødt		1		
DP762;Tarmobstruktion hos nyfødt forårsaget af mælkeprop		1		
DP768;Anden form for tarmobstruktion hos nyfødt		1		
DP769;Tarmobstruktion hos nyfødt UNS		1		
DP77;Nekrotiserende tarmbetændelse hos nyfødt		1		
DP779;Nekrotiserende enterocolitis hos nyfødt		1		
DP78;Andre perinatale sygdomme i fordøjelsessystemet		1		
DP780;Tarmperforation hos nyfødt		1		
DP780A;Mekoniumperitonitis		1		
DP781;Anden eller ikke specificeret form for peritonitis hos nyfødt		1		
DP782;Hæmatemese eller melæna hos nyfødt ved synkning af blod fra moder		1		
DP783;Ikke-infektiøs diaré hos nyfødt		1		
DP783A;Diaré hos nyfødt UNS		1		
DP788;Anden perinatal sygdom i fordøjelsessystemet		1		
DP788A;Medfødt levercirrose		1		
DP788B;Peptisk ulcus hos nyfødt		1		
DP789;Perinatal sygdom i fordøjelsessystemet UNS		1		
DP80;Nedsat legemstemperatur hos nyfødt		1		
DP800;Cold injury-syndrom		1		
DP808;Anden form for hypotermi hos nyfødt		1		
DP809;Hypotermi hos nyfødt UNS		1		
DP81;Andre forstyrrelser i regulation af legemstemperaturen hos nyfødt		1		
DP810;Hypertermi hos nyfødt på grund af omgivelserne		1		
DP818;Anden forstyrrelse i temperaturregulationen hos nyfødt		1		

DP818A;Feber forårsaget af dehydrering hos nyfødt		1	
DP819;Forstyrrelse i legemstemperatur hos nyfødt UNS		1	
DP83;Andre tilstande i huden hos nyfødt		1	
DP830;Sclerema neonatorum		1	
DP831;Toksisk erytem hos nyfødt		1	
DP832;Hydrops foetalis hos foster UNS		1	
DP833;Andet eller ikke specificeret ødem hos nyfødt		1	
DP834;Mammahypertrofi hos nyfødt		1	
DP834A;Ikke-infektiøs mastitis hos nyfødt		1	
DP835;Medfødt hydrocele		1	
DP835A;Hydrocele funiculi spermatici congenita		1	
DP835B;Hydrocele testis congenita		1	
DP836;Navlepolyp hos nyfødt		1	
DP838;Anden hudsygdom hos nyfødt		1	
DP838A;Bronze baby-syndrom		1	
DP838D;Neonatal sklerodermi		1	
DP838E;Neonatal urtikaria		1	
DP839;Neonatal hudsygdom eller tilstand i hud hos nyfødt UNS		1	
DP90;Kramper hos nyfødt		1	
DP909;Kramper hos nyfødt UNS		1	
DP91;Andre cerebrale forstyrrelser hos nyfødt		1	
DP910;Hjerneiskæmi hos nyfødt		1	
DP911;Erhvervede periventrikulære cyster hos nyfødt		1	
DP912;Cerebral leukomalaci hos nyfødt		1	
DP912A;Periventrikulær cerebral leukomalaci hos nyfødt		1	
DP912B;Subkortikal cerebral leukomalaci hos nyfødt		1	
DP913;Cerebral irritabilitet hos nyfødt		1	
DP914;Depressio cerebri neonati		1	
DP915;Coma neonati		1	
DP916;Neonatal hypoxisk iskæmisk encefalopati		1	
DP917;Erhvervet hydrocefalus hos nyfødt		1	
DP918;Anden neonatal cerebral forstyrrelse		1	

Supplement 2 Nielsen et al. Mortality after paediatric emergency calls for patients with and without pre-existing comorbidity (2023)

DP918A;Hjerneatrofi hos nyfødt		1		
DP918B;Ikke-traumatisk hjerneødem hos nyfødt		1		
DP919;Cerebral forstyrrelse hos nyfødt UNS		1		
DP92;Ernæringsproblemer hos nyfødt		1		
DP920;Opkastning hos nyfødt		1		
DP921;Tilbageløb og opgylpning af føde hos nyfødt		1		
DP921A;Tilbageløb af føde hos nyfødt		1		
DP921B;Opgylpning af føde hos nyfødt		1		
DP922;Langsom fødeindtagelse hos nyfødt		1		
DP923;Underernæring hos nyfødt		1		
DP923A;Fejlernæring hos nyfødt		1		
DP924;Overernæring hos nyfødt		1		
DP924A;Fedme hos nyfødt		1		
DP925;Ammeproblemer hos nyfødt		1		
DP928;Andet problem med fødeindtagelsen hos nyfødt		1		
DP929;Problem med fødeindtagelsen hos nyfødt UNS		1		
DP93;Utilsigtet virkning af lægemidler givet til nyfødt		1		
DP939;Reaktioner og forgiftninger hos nyfødt forårsaget af lægemiddel		1		
DP939A;Grey syndrome efter kloramfenikol hos nyfødt		1		
DP939B;Lægemiddelforgiftning hos foster		1		
DP939C;Lægemiddelforgiftning hos nyfødt		1		
DP939D;Utilsigtet reaktion på lægemiddel hos foster		1		
DP939E;Utilsigtet reaktion på lægemiddel hos nyfødt		1		
DP94;Forstyrrelser i muskeltonus hos nyfødt		1		
DP940;Forbigående myasthenia gravis hos nyfødt		1		
DP941;Medfødt muskulær hypertoni		1		
DP942;Medfødt muskulær hypotoni		1		
DP942A;Floppy baby syndrom UNS		1		
DP948;Anden forstyrrelse i muskeltonus hos nyfødt		1		
DP949;Forstyrrelse i muskeltonus hos nyfødt UNS		1		
DP95;Fosterdød og dødfødt barn uden kendt årsag		1		
DP950;Dødfødt barn		1		

DP959;Foetus mortuus ante partum		1	
DP96;Andre sygdomme i perinatalperioden		1	
DP960;Medfødt uræmi		1	
DP961;Abstinenssymptomer hos nyfødt forårsaget af prænatal eksponering af afhængighedsskabende stoffer		1	
DP962;Abstinenser hos nyfødt ved terapeutisk brug af lægemiddel		1	
DP963;Suturdiastase hos nyfødt		1	
DP963A;Craniotabes neonati		1	
DP964;Abortindgreb med følger for nyfødt		1	
DP965;Intrauterint indgreb med følger for nyfødt		1	
DP968;Anden sygdom opstået i perinatalperioden		1	
DP968A;Forbigående pustulose hos nyfødt		1	
DP969;Sygdom opstået i perinatalperioden UNS		1	
DQ00;Mangel på hjerne og lignende misdannelser			2
DQ000;Anencefali			2
DQ000A;Akrani			2
DQ000B;Amyelencefali			2
DQ000D;Hemianencefali			2
DQ001;Craniorhachischisis			2
DQ002;Iniencefali			2
DQ01;Hjernebrok			2
DQ010;Encephalocele frontalis			2
DQ011;Encephalocele nasofrontalis			2
DQ012;Encephalocele occipitalis			2
DQ018;Encefalocele med anden lokalisation			2
DQ019;Encefalocele UNS			2
DQ02;Mikrocefali			2
DQ029;Mikrocefali UNS			2
DQ029A;Hydromikrocefali			2
DQ03;Medfødt hydrocefalus			2
DQ030;Misdannelse af aquaeductus cerebri			2
DQ030A;Atresia aquaeducti cerebri			2
DQ030B;Stenosis congenita aquaeducti cerebri			2

Supplement 2 Nielsen et al. Mortality after paediatric emergency calls for patients with and without pre-existing comorbidity (2023)

DQ031;Atresia aperturae medianae et lateralis ventriculi quarti		1	
DQ031C;Dandy-Walkers syndrom		1	
DQ038;Anden form for medfødt hydrocefalus			2
DQ038A;Kommunicerende medfødt hydrocefalus			2
DQ038B;Ekstern medfødt hydrocefalus			2
DQ038C;Intern medfødt hydrocefalus			2
DQ039;Medfødt hydrocefalus UNS			2
DQ04;Andre medfødte misdannelser af hjernen			2
DQ040;Medfødt misdannelse af corpus callosum			2
DQ040A;Agenesia corporis callosi			2
DQ041;Arhinencefali			2
DQ042;Holoprosencefali			2
DQ043;Anden cerebral hypoplasi			2
DQ043A;Agyria			2
DQ043B;Hydranencefali			2
DQ043C;Hypoplasia congenita cerebri partialis			2
DQ043F;Lissencefali			2
DQ043G;Mikrogyri			2
DQ043H;Pakygyri			2
DQ044;Septo-optisk dysplasi		1	
DQ045;Megalencephalus			2
DQ046;Medfødte cerebrale cyster		1	
DQ046A;Medfødt cystisk hjernedegeneration		1	
DQ046B;Porencefali		1	
DQ046C;Schizencefali		1	
DQ048;Anden medfødt misdannelse i hjernen			2
DQ048A;Aplasia cerebelli			2
DQ048B;Makrogyri			2
DQ048C;Cerebellar hypoplasi			2
DQ048D;Fossa posterior cyste		1	
DQ048E;Ventrikulomegali		1	
DQ049;Medfødt misdannelse i hjernen UNS			2

DQ05;Spina bifida			2
DQ050;Cervikal spina bifida med hydrocefalus			2
DQ050A;Åben cervikal spina bifida med hydrocefalus			2
DQ051;Torakal spina bifida med hydrocefalus			2
DQ051A;Åben torakal spina bifida med hydrocefalus			2
DQ051B;Torakolumbal spina bifida med hydrocefalus			2
DQ051C;Åben torakolumbal spina bifida med hydrocefalus			2
DQ052;Lumbal spina bifida med hydrocefalus			2
DQ052A;Åben lumbal spina bifida med hydrocefalus			2
DQ052B;Lumbosakral spina bifida med hydrocefalus			2
DQ052C;Åben lumbosakral spina bifida med hydrocefalus			2
DQ053;Sakral spina bifida med hydrocefalus			2
DQ053A;Åben sakral spina bifida med hydrocefalus			2
DQ054;Spina bifida UNS med hydrocefalus			2
DQ055;Cervikal spina bifida uden hydrocefalus		1	
DQ055A;Åben cervikal spina bifida uden hydrocefalus		1	
DQ056;Torakal spina bifida uden hydrocefalus		1	
DQ056A;Åben torakal spina bifida uden hydrocefalus		1	
DQ056B;Torakolumbal spina bifida uden hydrocefalus		1	
DQ056C;Åben torakolumbal spina bifida uden hydrocefalus		1	
DQ057;Lumbal spina bifida uden hydrocefalus		1	
DQ057A;Åben lumbal spina bifida uden hydrocefalus		1	
DQ057B;Lumbosakral spina bifida uden hydrocefalus		1	
DQ057C;Åben lumbosakral spina bifida uden hydrocefalus		1	
DQ058;Sakral spina bifida uden hydrocefalus		1	
DQ058A;Åben sakral spina bifida uden hydrocefalus		1	
DQ059;Spina bifida UNS			2
DQ059A;Åben spina bifida UNS			2
DQ06;Andre medfødte misdannelser i rygmarv			2
DQ060;Amyeli			2
DQ061;Hypoplasi eller dysplasi af rygmarven		1	
DQ061A;Hypoplasi af rygmarven		1	

DQ061B;Dysplasi af rygmarven		1		
DQ061C;Atelomyeli		1		
DQ061D;Myelateli		1		
DQ062;Diastematomyeli		1		
DQ063;Anden medfødt misdannelse af cauda equina		1		
DQ064;Hydromyeli		1		
DQ064A;Hydrorachis		1		
DQ068;Anden medfødt misdannelse i rygmarv		1		
DQ068A;Medfødt rygmarvscyste		1		
DQ068B;Duplikation af rygmarven		1		
DQ069;Medfødt misdannelse i rygmarven UNS		1		
DQ07;Andre medfødte misdannelser i nervesystemet		1		
DQ070;Arnold-Chiaris syndrom		1		
DQ078;Anden medfødt misdannelse i nervesystemet		1		
DQ078A;Aplasi af perifer nerve		1		
DQ078B;Dislocatio congenita plexus brachialis		1		
DQ078C;Medfødt encefalopati		1		
DQ078E;Medfødt nervemisdannelse		1		
DQ078F;Malformatio congenita plexus brachialis		1		
DQ078G;Marcus Gunns syndrom		1		
DQ079;Medfødt misdannelse i nervesystemet UNS		1		
DQ10;Medfødte misdannelser af øjenlåg, tåreapparat og øjenhule		1		
DQ100;Medfødt blefaroptose		1		
DQ101;Medfødt ektropion af øjenlåg		1		
DQ102;Medfødt entropion af øjenlåg		1		
DQ103;Anden medfødt misdannelse af øjenlåg		1		
DQ103A;Ablefari		1		
DQ103B;Medfødt sammenvoksning af øjenlåg		1		
DQ103C;Agenesia ciliae		1		
DQ103D;Ankyloblepharon filiforme congenitum		1		
DQ103E;Blepharochalasis congenita		1		
DQ103F;Blepharophimosis congenita		1		

Supplement 2 Nielsen et al. Mortality after paediatric emergency calls for patients with and without pre-existing comorbidity (2023)

DQ103H;Kolobom i øjenlåg		1	
DQ103I;Medfødt dermoidcyste i øjenlåg		1	
DQ103J;Epiblepharon		1	
DQ103K;Medfødt misdannelse af øjenlåg UNS		1	
DQ103M;Accessorisk muskel i øjenlåg		1	
DQ103O;Symblepharon congenitum		1	
DQ103P;Epicanthus		1	
DQ104;Agenesi af tåreapparatet		1	
DQ104A;Aplasia puncti lacrimalis		1	
DQ105;Medfødt stenose eller striktur af tårekanal		1	
DQ105A;Medfødt stenose af tårekanal		1	
DQ105B;Medfødt stenose af ductus nasolacrimalis		1	
DQ105C;Medfødt striktur af tårekanal		1	
DQ105D;Medfødt striktur af ductus nasolacrimalis		1	
DQ106;Anden medfødt misdannelse i tåreapparatet		1	
DQ107;Medfødt misdannelse af øjenhule		1	
DQ11;Manglende, lille og stort øjeæble		1	
DQ110;Cystisk øjeæble		1	
DQ111;Anden form for manglende øjeæble		1	
DQ111A;Anoftalmi UNS		1	
DQ111B;Aplasi af øjeæble		1	
DQ112;Mikroftalmi		1	
DQ112A;Arvelig atrofi af øjeæble		1	
DQ112B;Kryptoftalmi UNS		1	
DQ112C;Medfødt hypoplasi af øjeæble		1	
DQ113;Makroftalmi		1	
DQ12;Medfødte misdannelser i øjets linse		1	
DQ120;Medfødt grå stær		1	
DQ120A;Cataracta coerulea		1	
DQ120B;Cataracta coralliformis		1	
DQ120C;Cataracta coronaria congenita		1	
DQ120E;Cataracta membranacea congenita		1	

DQ120F;Cataracta punctata		1	
DQ120G;Cataracta zonularis		1	
DQ121;Medfødt linseluksation		1	
DQ121A;Linseektopi		1	
DQ122;Kolobom i linse		1	
DQ123;Medfødt afaki		1	
DQ124;Sfærofaki		1	
DQ128;Anden medfødt misdannelse i øjets linse		1	
DQ128A;Medfødt deformation af linse		1	
DQ129;Medfødt misdannelse i øjets linse UNS		1	
DQ13;Medfødte misdannelser i forreste del af øje		1	
DQ130;Iriskolobom		1	
DQ130A;Kolobom UNS		1	
DQ131;Irisagenesi		1	
DQ132;Anden medfødt irismisdannelse		1	
DQ132A;Medfødt anisokori		1	
DQ132B;Pupilatresi		1	
DQ132C;Korektopi		1	
DQ132D;Heterochromia iridis congenita		1	
DQ132E;Medfødt irishypoplasi		1	
DQ133;Medfødt corneaopacitet		1	
DQ134;Anden medfødt misdannelse i cornea		1	
DQ134A;Cornea plana		1	
DQ134B;Keratoconus congenitus		1	
DQ134C;Macrocornea		1	
DQ134D;Melanosis corneae congenita		1	
DQ134E;Microcornea		1	
DQ134F;Peters anomali		1	
DQ134G;Embryotoxon posterius		1	
DQ134H;Embryotoxon anterius		1	
DQ135;Blå sklera		1	
DQ138;Anden medfødt misdannelse i forreste del af øje		1	

DQ138A;Membrana pupillaris persistens		1	
DQ138B;Riegers anomali		1	
DQ139;Medfødt misdannelse i forreste del af øje UNS		1	
DQ14;Medfødte misdannelser i bageste del af øje		1	
DQ140;Medfødt misdannelse i corpus vitreum		1	
DQ140A;Arteria hyaloidea persistens		1	
DQ140B;Corpus vitreum primarium persistens		1	
DQ140C;Medfødt opacitet i corpus vitreum		1	
DQ141;Medfødt misdannelse i nethinden		1	
DQ141A;Medfødt aneurisme i nethinden		1	
DQ141B;Degeneratio retinae pigmentosa		1	
DQ141C;Degeneratio tapetoretinalis		1	
DQ141D;Fibrae medullae retinae		1	
DQ141E;Retinitis punctata albescens		1	
DQ141F;Medfødt retinoskise		1	
DQ141G;Tortuositas vasorum retinae		1	
DQ142;Medfødt misdannelse i synsnervepapillen		1	
DQ142A;Coloboma discus nervi optici		1	
DQ143;Medfødt misdannelse i choroidea		1	
DQ143A;Coloboma choroideae		1	
DQ143B;Coloboma uveae		1	
DQ148;Anden medfødt misdannelse i bageste del af øje		1	
DQ148A;Coloboma fundi oculi		1	
DQ148B;Conus congenitus		1	
DQ148C;Conus nasalis		1	
DQ148D;Conus peripapillaris		1	
DQ148E;Conus temporalis		1	
DQ149;Medfødt misdannelse i bageste del af øje UNS		1	
DQ15;Andre medfødte misdannelser i øje		1	
DQ150;Medfødt glaukom		1	
DQ150A;Buftalmus		1	
DQ150C;Keratoglobus congenitus		1	

DQ150D;Makroftalmus ved medfødt glaukom		1	
DQ150E;Megalocornea		1	
DQ158;Anden medfødt misdannelse i øje		1	
DQ158A;Exophthalmus congenitus		1	
DQ158B;Melanosis oculi congenita		1	
DQ158C;Pigmentatio abnormis naevoides oculi		1	
DQ158D;Pseudoalbinismus		1	
DQ158E;Accessorisk øjenmuskel		1	
DQ159;Medfødt misdannelse i øje UNS		1	
DQ16;Medfødte misdannelser i øre med nedsat hørelse		1	
DQ160;Agenesi af ydre øre		1	
DQ161;Agenesi, atresi eller striktur af ydre øregang		1	
DQ161A;Agenesi af ydre øregang		1	
DQ161B;Aplasi af ydre øregang		1	
DQ161C;Atresi af ydre øregang		1	
DQ161D;Striktur af ydre øregang		1	
DQ162;Agenesi af tuba auditiva		1	
DQ163;Medfødt misdannelse af små høreknogler		1	
DQ163A;Medfødt sammenvoksning af små høreknogler		1	
DQ164;Anden medfødt misdannelse i mellemøret		1	
DQ164A;Medfødt kolesteatom i mellemøret		1	
DQ165;Medfødt misdannelse i indre øre		1	
DQ165A;Anomalia congenita ducti cochlearis		1	
DQ165B;Anomalia congenita labyrinthi membranacei		1	
DQ165C;Aplasia labyrinthi		1	
DQ169;Medfødt misdannelse i øre UNS med nedsat hørelse		1	
DQ169A;Monoti		1	
DQ169B;Anoti		1	
DQ17;Andre medfødte misdannelser i øre		1	
DQ170;Accessorisk ydre øre		1	
DQ170A;Polyotia		1	
DQ170B;Tragus accessorius		1	

Supplement 2 Nielsen et al. Mortality after paediatric emergency calls for patients with and without pre-existing comorbidity (2023)

DQ171;Makroti	0		
DQ172;Mikroti	0		
DQ173;Anden form for misdannet øre	0		
DQ173A;Antenneøre	0		
DQ174;Øre med atypisk placering	0		
DQ174A;Lavtsiddende øre	0		
DQ175;Aures alatae	0		
DQ178;Anden medfødt misdannelse i øre	0		
DQ178A;Agenesia lobuli auriculae	0		
DQ179;Medfødt misdannelse i øre UNS	0		
DQ18;Andre medfødte misdannelser i ansigt og hals		1	
DQ180;Brankialfistel eller brankialcyste		1	
DQ180A;Brankialcyste		1	
DQ180B;Brankialfistel		1	
DQ180C;Brankialfistel i svælget		1	
DQ181;Præaurikulær fistel eller cyste		1	
DQ181A;Præaurikulær cyste		1	
DQ181B;Medfødt fistel i ydre øre		1	
DQ181C;Medfødt cervikoaurikulær fistel		1	
DQ181D;Præaurikulær fistel		1	
DQ182;Anden brankiogen misdannelse		1	
DQ182A;Auricula cervicalis		1	
DQ182B;Otocefali		1	
DQ183;Pterygium colli		1	
DQ184;Makrostomi		1	
DQ185;Mikrostomi		1	
DQ186;Makrokeili		1	
DQ187;Mikrokeili		1	
DQ188;Anden medfødt misdannelse i ansigt eller hals		1	
DQ188A;Cystis congenita cervicalis medialis		1	
DQ188B;Cystis congenita facialis medialis		1	
DQ188C;Fistula congenita cervicalis medialis		1	

Supplement 2 Nielsen et al. Mortality after paediatric emergency calls for patients with and without pre-existing comorbidity (2023)

DQ188D;Fistula congenita facialis medialis		1	
DQ188F;Nasal hypoplasi hos foster i 11-14 uger		1	
DQ188G;Prænasalt ødem		1	
DQ189;Medfødt misdannelse i ansigt eller hals UNS		1	
DQ20;Medfødte misdannelser af hjertekamre			2
DQ200;Truncus arteriosus communis			2
DQ201;Transpositio vasorum incompleta i højre ventrikel			2
DQ202;Transpositio vasorum incompleta i venstre ventrikel			2
DQ203;Transpositio vasorum completa			2
DQ203A;Dextrotranspositio aortae			2
DQ204;Ventriculus cordis communis			2
DQ205;Inversio ventriculorum cordis			2
DQ205A;Levotranspositio			2
DQ205B;Transpositio vasorum correcta			2
DQ206;Isomerismus auricularum atriorum med aspleni eller polyspleni			2
DQ208;Anden medfødt misdannelse af hjertekamre			2
DQ208A;Cor biloculare			2
DQ208B;Cor triloculare			2
DQ208C;Cor triloculare biventriculare			2
DQ208S;Cor uniloculare			2
DQ209;Medfødt misdannelse af hjertekamre UNS			2
DQ21;Medfødte misdannelser af hjerteskillevæg		1	
DQ210;Ventrikelseptumdefekt		1	
DQ211;Atrieseptumdefekt		1	
DQ211A;Defectus sinus coronarii cordis		1	
DQ211B;Defectus sinus venosi cordis		1	
DQ211C;Åbentstående foramen ovale		1	
DQ212;Atrioventrikulær septumdefekt			2
DQ213;Steno-Fallots tetralogi		1	
DQ213A;Steno-Fallots tetralogi med pulmonalklapatresi			2
DQ214;Aortopulmonal septumdefekt		1	
DQ218;Anden medfødt misdannelse af hjerteskillevæg		1	

Supplement 2 Nielsen et al. Mortality after paediatric emergency calls for patients with and without pre-existing comorbidity (2023)

DQ218A;Eisenmengers defekt		1	
DQ218B;Ostium atrioventriculare commune		1	
DQ218C;Pentalogia Fallot		1	
DQ219;Medfødt misdannelse af hjerteskillevæg UNS		1	
DQ22;Medfødte misdannelser af pulmonal- og trikuspidalklapper		1	
DQ220;Pulmonalklapatresi			2
DQ220A;Absent pulmonary valve-syndrom			2
DQ221;Medfødt pulmonalklapstenose		1	
DQ222;Medfødt pulmonalklapinsufficiens			2
DQ223;Anden medfødt misdannelse af pulmonalklap			2
DQ224;Medfødt trikuspidalklapstenose			2
DQ225;Ebsteins anomali			2
DQ226;Hypoplastisk højre hjerte-syndrom			2
DQ228;Anden medfødt misdannelse af trikuspidalklap			2
DQ229;Medfødt misdannelse af trikuspidalklap UNS			2
DQ23;Medfødte misdannelser af aorta- og mitralklapperne		1	
DQ230;Medfødt aortaklapstenose		1	
DQ231;Medfødt aortaklapinsufficiens		1	
DQ231A;Valvula aortae bicuspidalis		1	
DQ232;Medfødt mitralklapstenose			2
DQ233;Medfødt mitralklapinsufficiens		1	
DQ234;Hypoplastisk venstre hjerte-syndrom			2
DQ238;Anden medfødt misdannelse af aorta- eller mitralklap			2
DQ239;Medfødt misdannelse af aorta- eller mitralklap UNS			2
DQ24;Andre medfødte misdannelser i hjerte			2
DQ240;Dekstrokardi		1	
DQ241;Levokardi			2
DQ242;Cor triatriatum			2
DQ243;Infundibulær pulmonalstenose		1	
DQ244;Medfødt subaortastenose		1	
DQ245;Medfødt misdannelse af koronararterie		1	
DQ245A;Medfødt aneurisme på koronararterie		1	

Supplement 2 Nielsen et al. Mortality after paediatric emergency calls for patients with and without pre-existing comorbidity (2023)

DQ245B;Aplasi af koronararterie		1	
DQ245C;Atresi af koronararterie		1	
DQ246;Medfødt hjerteblok		1	
DQ248;Anden medfødt hjertemisdannelse			2
DQ248A;Akardi			2
DQ248C;Hjerteatresi			2
DQ248D;Atresi af hjerteklapper UNS			2
DQ248E;Medfødt kardiomegali		1	
DQ248F;Medfødt divertikel på venstre hjertekammer		1	
DQ248G;Hjerteektopi			2
DQ248H;Misdannelse i myokardiet		1	
DQ248I;Misdannelse i perikardiet		1	
DQ248J;Malpositio congenita cordis			2
DQ248K;Medfødt stenose af hjerteklapper UNS		1	
DQ248L;Uhls sygdom			2
DQ249;Medfødt hjertemisdannelse UNS			2
DQ25;Medfødte misdannelser i de store arterier		1	
DQ250;Persisterende ductus arteriosus		1	
DQ251;Coarctatio aortae		1	
DQ251A;Medfødt stenose i isthmus aortae		1	
DQ252;Aortaatresi		1	
DQ253;Medfødt aortastenose		1	
DQ254;Anden medfødt misdannelse i aorta		1	
DQ254A;Aortaagenesi		1	
DQ254B;Medfødt aortaaneurisme		1	
DQ254C;Aneurysma congenitum sinus coronarii		1	
DQ254D;Aortaaplasi		1	
DQ254E;Arcus aortae dexter persistens		1	
DQ254F;Convolutio arcus aortae persistens		1	
DQ254G;Medfødt aortadilatation		1	
DQ254H;Aortahypoplasi		1	
DQ254I;Vaskulær ring af aorta		1	

Supplement 2 Nielsen et al. Mortality after paediatric emergency calls for patients with and without pre-existing comorbidity (2023)

DQ255;Pulmonalarterieatresi			2
DQ256;Medfødt pulmonalarteriestenose			2
DQ257;Anden medfødt misdannelse af pulmonalarterie			2
DQ257A;Agenesi af pulmonalarterie			2
DQ257B;Medfødt aneurisme på pulmonalarterie			2
DQ257C;Medfødt arteriovenøst pulmonalt aneurisme			2
DQ257D;Aplasi af pulmonalarterie			2
DQ257E;Arteria pulmonalis aberrans			2
DQ257F;Medfødt hypoplasia af pulmonalarterie			2
DQ257G;Medfødt misdannelse i pulmonalarterie UNS			2
DQ258;Anden medfødt misdannelse i de store arterier		1	
DQ259;Medfødt misdannelse i de store arterier UNS		1	
DQ26;Medfødte misdannelser i de store vener		1	
DQ260;Medfødt stenose i vena cava		1	
DQ261;Vena cava superior sinistra persistens		1	
DQ262;Total anomali af lungevenerne			2
DQ263;Partiel anomali af lungevenerne		1	
DQ264;Anomali af lungevenerne UNS		1	
DQ265;Anomalia venae portae		1	
DQ266;Fistula arteriovenosa hepaticoporta congenita		1	
DQ268;Anden medfødt misdannelse i de store vener		1	
DQ268A;Agenesia venae cavae		1	
DQ268B;Scimitar syndrom		1	
DQ268C;Vena cardinalis posterior sinistra persistens		1	
DQ268D;Vena cava inferior med atypisk indløb af vena azygos		1	
DQ269;Medfødt misdannelse i de store vener UNS		1	
DQ27;Andre medfødte misdannelser i det perifere kredsløb		1	
DQ270;Atresi eller hypoplasi af arteria umbilicalis		1	
DQ270A;Arteria umbilicalis anomalis		1	
DQ270B;Atresi af arteria umbilicalis		1	
DQ270C;Hypoplasi af arteria umbilicalis		1	
DQ271;Medfødt stenose af nyrearterie		1	

Supplement 2 Nielsen et al. Mortality after paediatric emergency calls for patients with and without pre-existing comorbidity (2023)

DQ272;Anden medfødt misdannelse i nyrearterie		1	
DQ272A;Arteria accessoria renis		1	
DQ272B;Arteriae renales multiplex		1	
DQ273;Medfødt perifer arteriovenøs malformation		1	
DQ273A;Medfødt spinal arteriovenøs malformation		1	
DQ274;Medfødt flebektasi		1	
DQ278;Anden medfødt misdannelse i det perifere kredsløb		1	
DQ278A;Agenesi af arterie IKA		1	
DQ278B;Agenesi af vene IKA		1	
DQ278C;Arteria subclavia aberrans		1	
DQ278D;Atresi af arterie IKA		1	
DQ278E;Atresi af vene IKA		1	
DQ278F;Medfødt hypoplasi af arterie IKA		1	
DQ278G;Medfødt hypoplasi af vene IKA		1	
DQ278H;Medfødt stenose af arterie IKA		1	
DQ278I;Medfødt striktur af arterie IKA		1	
DQ278J;Medfødte varicer		1	
DQ278K;Vas aberrans UNS		1	
DQ278L;Persisterende marginal vene UNS		1	
DQ279;Medfødt misdannelse i det perifere kredsløb UNS		1	
DQ28;Andre medfødte misdannelser i kredsløbsorganerne		1	
DQ280;Arteriovenøs misdannelse i præcerebralt kar		1	
DQ281;Anden misdannelse i præcerebralt kar		1	
DQ282;Arteriovenøs misdannelse i cerebralt kar		1	
DQ283;Anden misdannelse i cerebralt kar		1	
DQ283A;Aneurysma congenitum circulus Willisii		1	
DQ283B;Medfødt cerebralt aneurisme uden ruptur		1	
DQ283C;Atresi af cerebral arterie		1	
DQ283D;Cerebrovaskulær atresi		1	
DQ283E;Medfødt cerebral arteriel misdannelse UNS		1	
DQ288;Anden medfødt misdannelse i kredsløbsorganerne		1	
DQ289;Medfødt misdannelse i kredsløbsorganerne UNS		1	

Supplement 2 Nielsen et al. Mortality after paediatric emergency calls for patients with and without pre-existing comorbidity (2023)

DQ30;Medfødte misdannelser i næsen		1	
DQ300;Koanal atresi		1	
DQ300A;Atresi af næsebor		1	
DQ300B;Medfødt koanal stenose		1	
DQ301;Agenesi af næsen		1	
DQ302;Medfødt fissur i næsen		1	
DQ303;Medfødt perforation af næseskillevæg		1	
DQ308;Anden medfødt misdannelse i næsen		1	
DQ308A;Nasus accessorius		1	
DQ309;Medfødt misdannelse i næsen UNS		1	
DQ31;Medfødte misdannelser i strubehovedet		1	
DQ310;Medfødt larynxmembran		1	
DQ310A;Membrana laryngis glottica		1	
DQ310B;Membrana laryngis subglottica		1	
DQ311;Medfødt subglottisk stenose		1	
DQ312;Hypoplasi af larynx		1	
DQ313;Laryngocele		1	
DQ318;Anden medfødt misdannelse i strubehovedet		1	
DQ318A;Agenesi af epiglottis		1	
DQ318B;Agenesi af glottis		1	
DQ318C;Agenesi af larynx		1	
DQ318D;Atresi af epiglottis		1	
DQ318E;Atresi af glottis		1	
DQ318F;Atresi af larynx		1	
DQ318G;Kondromalaci i epiglottis		1	
DQ318H;Kondromalaci i larynx		1	
DQ318I;Medfødt fissur i epiglottis		1	
DQ318J;Medfødt fissur i larynx		1	
DQ318K;Medfødt misdannelse i epiglottis UNS		1	
DQ318L;Medfødt stenose i larynx IKA		1	
DQ319;Medfødt misdannelse i strubehovedet UNS		1	
DQ32;Medfødte misdannelser i luftrør og bronkier		1	

Supplement 2 Nielsen et al. Mortality after paediatric emergency calls for patients with and without pre-existing comorbidity (2023)

DQ320;Tracheomalacia congenita		1	
DQ321;Anden medfødt misdannelse i trakea		1	
DQ321A;Atresi af trakea		1	
DQ321B;Kondromalaci i trakea		1	
DQ321C;Medfødt dilatation af trakea		1	
DQ321D;Medfødt divertikel i trakea		1	
DQ321E;Medfødt fistel i trakea		1	
DQ321F;Medfødt stenose i trakea		1	
DQ321G;Tracheocele congenita		1	
DQ322;Bronchomalacia congenita		1	
DQ323;Medfødt bronkiestenose		1	
DQ324;Anden medfødt bronkiemisdannelse		1	
DQ324A;Bronkieagenesi		1	
DQ324B;Bronkieaplasi		1	
DQ324C;Bronkieatresi		1	
DQ324D;Medfødt bronkiedivertikel		1	
DQ324E;Medfødt bronkiemisdannelse UNS		1	
DQ33;Medfødte lungemisdannelser		1	
DQ330;Cystelunge		1	
DQ330A;Medfødt bronkogen cyste		1	
DQ331;Accessorisk lungelap		1	
DQ332;Sequestrum pulmonum		1	
DQ333;Agenesi af lunge		1	
DQ333A;Agenesi af lungelap		1	
DQ333B;Aplasi af lunge		1	
DQ334;Medfødt bronkiektasi		1	
DQ335;Ektopisk væv i lunge		1	
DQ335A;Hamartoma pulmonis		1	
DQ336;Hypoplasi eller dysplasi af lunge		1	
DQ336A;Lungedysplasi		1	
DQ336B;Medfødt lungehypoplasi		1	
DQ338;Anden medfødt lungemisdannelse		1	

DQ339;Medfødt lungemisdannelse UNS		1	
DQ34;Andre medfødte misdannelser i åndedrætsorganer		1	
DQ340;Medfødt misdannelse af lungehinde		1	
DQ341;Medfødt mediastinal cyste		1	
DQ348;Anden medfødt misdannelse i åndedrætsorganer		1	
DQ348A;Nasofaryngeal atresi		1	
DQ349;Medfødt misdannelse i åndedrætsorganer UNS		1	
DQ35;Ganespalte		1	
DQ353;Ganespalte i den bløde gane		1	
DQ355;Ganespalte i både den hårde og den bløde gane		1	
DQ357;Submukøs ganespalte		1	
DQ359;Ganespalte UNS		1	
DQ36;Læbespalte		1	
DQ360;Bilateral læbespalte		1	
DQ361;Median læbespalte		1	
DQ369;Unilateral læbespalte		1	
DQ37;Læbe-gumme-ganespalte		1	
DQ370;Dobbeltsidig læbe-gummespalte		1	
DQ371;Enkeltsidig læbe-gummespalte		1	
DQ372;Dobbeltsidig læbe-gummespalte med ganespalte i den bløde gane		1	
DQ373;Enkeltsidig læbe-gummespalte med ganespalte i den bløde gane		1	
DQ374;Dobbeltsidig læbe-gumme-ganespalte		1	
DQ375;Enkeltsidig læbe-gumme-ganespalte		1	
DQ378;Ganespalte med dobbeltsidig læbespalte UNS		1	
DQ379;Ganespalte med enkeltsidig læbespalte UNS		1	
DQ38;Andre medfødte misdannelser i tunge, mund og svælg		1	
DQ380;Medfødt misdannelse i læbe IKA		1	
DQ380A;Medfødt læbefistel		1	
DQ380B;Frenulum anomale labii		1	
DQ380C;Van der Woudes syndrom		1	
DQ381;Ankyloglossi	0		
DQ381A;Frenulum breve linguae (findes ikke i SKS)	0		

DQ382;Makroglossi		1	
DQ383;Anden medfødt misdannelse i tungen		1	
DQ383A;Aglossi		1	
DQ383B;Medfødt tungefistel		1	
DQ383C;Medfødt tungehypoplasi		1	
DQ383D;Lingua bifida		1	
DQ383E;Mikroglossi		1	
DQ383F;Ranula congenita		1	
DQ384;Medfødt misdannelse i spytkirtel eller udførselsgang		1	
DQ384A;Agenesi af udførselsgang fra spytkirtel		1	
DQ384B;Agenesi af spytkirtel		1	
DQ384C;Atresi af udførselsgang fra spytkirtel		1	
DQ384D;Atresi af spytkirtel		1	
DQ384E;Ductus salivarius accessorius		1	
DQ384F;Medfødt spytkirtelfistel		1	
DQ384G;Glandula salivaria accessoria		1	
DQ384H;Medfødt hypoplasi af udførselsgang fra spytkirtel		1	
DQ384I;Medfødt misdannelse af udførselsgang fra spytkirtel		1	
DQ384J;Medfødt misdannelse af spytkirtel IKA		1	
DQ385;Medfødt misdannelse i ganen IKA		1	
DQ385A;Agenesi af uvula		1	
DQ385B;Høj hvælvet gane		1	
DQ386;Anden medfødt misdannelse i mundhule		1	
DQ386A;Duplikation af munden		1	
DQ386B;Medfødt hypoplasi af mundhule		1	
DQ386C;Medfødt misdannelse af tandkød		1	
DQ386D;Medfødt misdannelse i mund UNS		1	
DQ387;Medfødt divertikel i pharynx		1	
DQ388;Anden medfødt misdannelse i pharynx		1	
DQ388A;Atresi af pharynx		1	
DQ388B;Medfødt misdannelse i pharynx UNS		1	
DQ388C;Pharynx imperforatus		1	

DQ39;Medfødte misdannelser i spiserør		1	
DQ390;Øsofagusatresi uden fistel		1	
DQ390A;Øsofagusatresi UNS		1	
DQ391;Øsofagusatresi med medfødt trakeoøsofageal fistel		1	
DQ391A;Øsofagusatresi med medfødt bronkoøsofageal fistel		1	
DQ392;Medfødt trakeoøsofageal fistel uden øsofagusatresi		1	
DQ392A;Medfødt øsofagobronkial fistel uden øsofagusatresi		1	
DQ393;Medfødt stenose eller striktur i øsofagus		1	
DQ394;Membrana oesophagi		1	
DQ395;Medfødt øsofagusdilatation		1	
DQ395A;Medfødt kardiospasme		1	
DQ396;Øsofagusdivertikel		1	
DQ398;Anden medfødt misdannelse i øsofagus		1	
DQ398A;Øsofagusagenesi		1	
DQ398B;Brachyøsofagus		1	
DQ398C;Dislocatio congenita oesophagi		1	
DQ398D;Duplikation af øsofagus		1	
DQ398E;Medfødt øsofagusfistel		1	
DQ398F;Medfødt øsofagushypoplasi		1	
DQ398G;Megaloøsofagus congenitus		1	
DQ399;Medfødt misdannelse i øsofagus UNS		1	
DQ40;Andre medfødte misdannelser i øvre fordøjelsesorganer		1	
DQ400;Medfødt pylerospasme		1	
DQ401;Medfødt hiatushernie		1	
DQ402;Anden medfødt misdannelse i mavesæk		1	
DQ402B;Dislocatio congenita ventriculi		1	
DQ402C;Medfødt ventrikeldivertikel		1	
DQ402D;Duplikation af mavesæk		1	
DQ402E;Medfødt ventrikelfistel		1	
DQ402F;Megalogastria		1	
DQ402G;Microgastria		1	
DQ402H;Transpositio ventriculi		1	

Supplement 2 Nielsen et al. Mortality after paediatric emergency calls for patients with and without pre-existing comorbidity (2023)

DQ403;Medfødt misdannelse i mavesæk UNS		1	
DQ408;Anden medfødt misdannelse i øvre fordøjelsesorganer		1	
DQ409;Medfødt misdannelse i øvre fordøjelsesorganer UNS		1	
DQ41;Agenesi, atresi og medfødt stenose af tyndtarm		1	
DQ410;Agenesi, atresi eller medfødt stenose af duodenum		1	
DQ410A;Agenesi af duodenum		1	
DQ410B;Atresi af duodenum		1	
DQ410C;Medfødt stenose af duodenum		1	
DQ411;Agenesi, atresi eller medfødt stenose af jejunum		1	
DQ411A;Agenesi af jejunum		1	
DQ411B;Atresi af jejunum		1	
DQ411C;Imperforatio jejuni		1	
DQ411D;Medfødt stenose af jejunum		1	
DQ412;Agenesi, atresi eller medfødt stenose af ileum		1	
DQ412A;Agenesi af ileum		1	
DQ412B;Atresi af ileum		1	
DQ412C;Medfødt stenose af ileum		1	
DQ418;Agenesi, atresi eller medfødt stenose af anden del af tyndtarm		1	
DQ419;Agenesi, atresi eller medfødt stenose af tyndtarm UNS		1	
DQ42;Agenesi, atresi og medfødt stenose af tyktarm, endetarm og analkanal		1	
DQ420;Agenesi, atresi eller medfødt stenose af rektum med fistel		1	
DQ420A;Agenesi af rektum med fistel		1	
DQ420B;Atresi af rektum med fistel		1	
DQ420C;Medfødt stenose af rektum med fistel		1	
DQ421;Agenesi, atresi eller medfødt stenose af rektum uden fistel		1	
DQ421A;Agenesi af rektum uden fistel		1	
DQ421B;Atresi af rektum uden fistel		1	
DQ421C;Imperforatio recti		1	
DQ421D;Medfødt stenose af rektum uden fistel		1	
DQ422;Agenesi, atresi eller medfødt stenose af anus med fistel		1	
DQ422A;Agenesi af anus med fistel		1	
DQ422B;Atresi af anus med fistel		1	

DQ422C;Medfødt stenose af anus med fistel		1	
DQ423;Agenesi, atresi eller medfødt stenose af anus uden fistel		1	
DQ423A;Agenesi af anus uden fistel		1	
DQ423B;Atresi af anus uden fistel		1	
DQ423C;Imperforatio ani		1	
DQ423D;Medfødt stenose af anus uden fistel		1	
DQ428;Agenesi, atresi eller medfødt stenose af anden del af tyktarm		1	
DQ429;Agenesi, atresi eller medfødt stenose af tyktarm UNS		1	
DQ43;Andre medfødte misdannelser i mave-tarm-kanalen		1	
DQ430;Meckels divertikel		1	
DQ430A;Ductus omphalomesentericus persistens		1	
DQ431;Medfødt megacolon		1	
DQ431A;Megasigmoideum congenitum		1	
DQ432;Anden medfødt forstyrrelse i tyktarmens funktion		1	
DQ432A;Medfødt colondilatation		1	
DQ433;Medfødt misdannelse i mesenterie, oment eller peritoneum		1	
DQ433A;Medfødte anormale sammenvoksninger i oment		1	
DQ433B;Medfødte sammenvoksninger i peritoneum		1	
DQ433C;Jacksons membran		1	
DQ433D;Malrotation af caecum		1	
DQ433E;Malrotation af colon		1	
DQ433F;Malrotation af tyndtarm		1	
DQ433G;Mesenterium ileo-colicum commune		1	
DQ433H;Mesenterium universale		1	
DQ433I;Mesocolon commune		1	
DQ434;Duplikation af tarm		1	
DQ434A;Duplikation af caecum		1	
DQ435;Ektopisk anus		1	
DQ435A;Anus perinealis		1	
DQ435B;Anus vestibularis		1	
DQ436;Medfødt anal eller rektal fistel		1	
DQ436A;Medfødt anal fistel		1	

DQ436B;Medfødt rektal fistel		1	
DQ437;Cloaca persistens		1	
DQ437A;Cloaca UNS		1	
DQ438;Anden medfødt misdannelse i mave-tarm-kanalen		1	
DQ438A;Medfødt blind loop-syndrom		1	
DQ438B;Caecum mobile		1	
DQ438C;Medfødt colondivertikulitis		1	
DQ438D;Medfødt colondivertikel		1	
DQ438E;Medfødt divertikel i duodenum		1	
DQ438F;Medfødt divertikel i tyndtarm		1	
DQ438G;Colon elongatum		1	
DQ438H;Duplikation af anus		1	
DQ438I;Duplikation af appendiks		1	
DQ438J;Medfødt hypoplasi af tarm UNS		1	
DQ438K;Medfødt anorektal misdannelse UNS		1	
DQ438L;Medfødt misdannelse af appendiks UNS		1	
DQ438M;Medfødt misdannelse af tyktarm UNS		1	
DQ438N;Medfødt misdannelse af rektum UNS		1	
DQ438O;Megaloappendiks		1	
DQ438P;Megaloduodenum		1	
DQ438Q;Mikrocolon		1	
DQ438R;Transpositio appendicis		1	
DQ438S;Transpositio coli		1	
DQ438T;Transpositio intestini tenuis		1	
DQ439;Medfødt misdannelse i mave-tarm-kanalen UNS		1	
DQ44;Medfødte misdannelser i galdeblære, galdegange og lever		1	
DQ440;Agenesi, aplasi eller hypoplasi af galdeblære		1	
DQ440A;Agenesi af galdeblære		1	
DQ440B;Aplasi af galdeblære		1	
DQ440C;Hypoplasi af galdeblære		1	
DQ441;Anden medfødt misdannelse i galdeblære		1	
DQ441A;Duplikation af galdeblære		1	

DQ441B;Intrahepatisk galdeblære		1	
DQ441C;Medfødt misdannelse i galdeblære UNS		1	
DQ442;Atresi af galdegang		1	
DQ442A;Aplasi af galdegang		1	
DQ443;Medfødt stenose eller striktur af galdegang		1	
DQ443A;Medfødt tillukning af galdeveje UNS		1	
DQ443B;Medfødt stenose af galdegang		1	
DQ443C;Medfødt stenose af ductus choledochus		1	
DQ443D;Medfødt stenose af galdeveje UNS		1	
DQ443E;Medfødt striktur af galdegang		1	
DQ444;Koledokuscyste		1	
DQ444A;Carolis sygdom		1	
DQ445;Anden medfødt misdannelse i galdegang		1	
DQ445A;Medfødt deformitet i galdegang		1	
DQ445B;Ductus hepaticus accessorius		1	
DQ445C;Duplikation af galdegang		1	
DQ445D;Duplikation af ductus cysticus		1	
DQ445E;Medfødt misdannelse i galdegang UNS		1	
DQ445F;Medfødt misdannelse af ductus hepaticus		1	
DQ446;Medfødt cystelever		1	
DQ447;Anden medfødt misdannelse i lever		1	
DQ447A;Agenesi af lever		1	
DQ447B;Alagilles syndrom		1	
DQ447C;Duplikation af lever		1	
DQ447D;Hepar accessorium		1	
DQ447E;Medfødt leverforstørrelse		1	
DQ447F;Medfødt hypoplasi af lever		1	
DQ447G;Medfødt misdannelse i lever UNS		1	
DQ45;Andre medfødte misdannelser i fordøjelsesorganer		1	
DQ450;Agenesi, aplasi og hypoplasi af pancreas		1	
DQ450A;Agenesi af pancreas		1	
DQ450B;Aplasi af pancreas		1	

DQ450C;Medfødt hypoplasi af pancreas		1	
DQ451;Pancreas annulare		1	
DQ452;Medfødt pancreascyste		1	
DQ453;Anden medfødt misdannelse af pancreas eller ductus pancreatis		1	
DQ453A;Duplikation af pancreas		1	
DQ453B;Medfødt misdannelse i ductus pancreatis UNS		1	
DQ453C;Medfødt misdannelse i pancreas UNS		1	
DQ453D;Pancreas accessorium		1	
DQ458;Anden medfødt misdannelse i fordøjelsesorganer		1	
DQ458A;Agenesi i fordøjelseskanal UNS		1	
DQ458B;Medfødt omentcyste		1	
DQ458C;Duplicatio organorum digestionis UNS		1	
DQ458D;Medfødt malposition i fordøjelseskanal UNS		1	
DQ459;Medfødt misdannelse i fordøjelsessystem UNS		1	
DQ50;Medfødte misdannelser i æggestokke, æggeledere og livmoderens ligamenter		1	
DQ500;Agenesi af æggestok		1	
DQ500A;Aplasi af æggestok		1	
DQ501;Medfødt ovariecyste		1	
DQ502;Medfødt ovarietorsion		1	
DQ503;Anden medfødt misdannelse af æggestok		1	
DQ503A;Medfødt hypoplasi af æggestok		1	
DQ503B;Ovarium accessorium		1	
DQ503C;Medfødt misdannelse af æggestok UNS		1	
DQ504;Medfødt cyste i æggeleder		1	
DQ505;Parovarialcyste		1	
DQ505A;Torsio cystis parovarialis		1	
DQ506;Anden medfødt misdannelse af æggeleder eller ligamentum latum uteri		1	
DQ506A;Agenesi af æggeleder		1	
DQ506B;Aplasi af æggeleder		1	
DQ506C;Medfødt atresi af æggeleder		1	
DQ506D;Duplicatio salpingis		1	
DQ506E;Medfødt hypoplasi af æggeleder		1	

DQ506F;Medfødt misdannelse af ligamentum latum uteri		1	
DQ506G;Medfødt misdannelse af æggeleder UNS		1	
DQ51;Medfødte misdannelser i livmoder og livmoderhals		1	
DQ510;Agenesi eller aplasi af livmoder		1	
DQ510A;Agenesi af livmoder		1	
DQ510B;Aplasi af livmoder		1	
DQ511;Duplikation af livmoder med duplikation af livmoderhals og vagina		1	
DQ512;Anden duplikation af livmoder		1	
DQ512A;Uterus accessorius		1	
DQ512B;Uterus bipartitus		1	
DQ513;Uterus bicornis		1	
DQ514;Uterus unicornis		1	
DQ515;Agenesi eller aplasi af livmoderhals		1	
DQ515A;Agenesi af livmoderhals		1	
DQ515B;Aplasi af livmoderhals		1	
DQ516;Medfødt cyste i livmoderhals		1	
DQ517;Medfødt uterointestinal eller uterourinal fistel		1	
DQ517A;Medfødt rektouterin fistel		1	
DQ517B;Medfødt uteroabdominal fistel		1	
DQ517C;Medfødt uterointestinal fistel		1	
DQ517D;Medfødt vesikouterin fistel		1	
DQ518;Anden medfødt misdannelse i livmoder eller livmoderhals		1	
DQ518A;Medfødte cervikovaginale sammenvoksninger		1	
DQ518B;Atresi af livmoderhals		1	
DQ518C;Atresi af livmoder		1	
DQ518D;Cervix uteri imperforata		1	
DQ518F;Elongatio cervicis uteri		1	
DQ518G;Medfødt hypoplasi af livmoderhals		1	
DQ518H;Medfødt hypoplasi af livmoder		1	
DQ518J;Medfødt prolaps af livmoderhals		1	
DQ518K;Uterus septus		1	
DQ519;Medfødt misdannelse i livmoder eller livmoderhals UNS		1	

DQ52;Andre medfødte misdannelser i kvindelige kønsorganer		1	
DQ520;Agenesi af vagina		1	
DQ520A;Aplasi af vagina		1	
DQ521;Duplikation af vagina		1	
DQ522;Medfødt rektovaginal fistel		1	
DQ523;Hymen imperforatum		1	
DQ524;Anden medfødt misdannelse i vagina		1	
DQ524A;Medfødt cyste i vagina		1	
DQ524B;Medfødt enterovaginal fistel		1	
DQ524C;Medfødt hypoplasi af vagina		1	
DQ524D;Medfødt misdannelse i vagina UNS		1	
DQ524E;Medfødt stenose i vagina		1	
DQ524F;Vagina imperforata		1	
DQ525;Fusio labiarum vulvae		1	
DQ526;Medfødt misdannelse af klitoris		1	
DQ526A;Aplasi af klitoris		1	
DQ526B;Medfødt hypertrofi af klitoris		1	
DQ526C;Medfødt hypotrofi af klitoris		1	
DQ527;Anden medfødt misdannelse af vulva		1	
DQ527A;Agenesi af vulva		1	
DQ527B;Medfødt cyste i vulva		1	
DQ527C;Medfødt vulvorektal fistel		1	
DQ527D;Medfødt misdannelse i vulva UNS		1	
DQ528;Anden medfødt misdannelse i kvindeligt kønsorgan		1	
DQ528A;Sinus urogenitalis hos kvinde		1	
DQ529;Medfødt misdannelse i kvindeligt kønsorgan UNS		1	
DQ53;Manglende nedsynkning af testikler		1	
DQ530;Ektopisk testikel		1	
DQ530A;Dobbeltsidig ektopisk testikel		1	
DQ530B;Enkeltsidig ektopisk testikel		1	
DQ531;Enkeltsidig testikelretention		1	
DQ531A;Enkeltsidig ikke-palpabel testikel		1	

DQ532;Dobbeltsidig testikelretention		1	
DQ532A;Dobbeltsidig ikke-palpabel testikel		1	
DQ539;Testikelretention UNS		1	
DQ54;Hypospadi		1	
DQ540;Hypospadi på glans penis		1	
DQ540A;Hypospadia coronalis		1	
DQ541;Hypospadi på corpus penis		1	
DQ542;Penoskrotal hypospadi		1	
DQ543;Perineal hypospadi		1	
DQ544;Penis arcuatus		1	
DQ548;Anden hypospadi		1	
DQ549;Hypospadi UNS		1	
DQ55;Andre medfødte misdannelser i mandlige kønsorganer		1	
DQ550;Agenesi eller aplasi af testikel		1	
DQ550A;Agenesi af testikel		1	
DQ550B;Aplasi af testikel		1	
DQ550C;Monorkisme		1	
DQ551;Hypoplasi af testikel eller scrotum		1	
DQ551A;Fusio testis		1	
DQ551B;Hypoplasi af scrotum		1	
DQ551C;Hypoplasi af testikel		1	
DQ552;Anden medfødt misdannelse af testikel eller scrotum		1	
DQ552A;Testisinversion		1	
DQ552B;Medfødt misdannelse af scrotum UNS		1	
DQ552C;Medfødt misdannelse af testikel UNS		1	
DQ552D;Polyorkisme		1	
DQ552E;Testis migrans		1	
DQ552F;Retraktil testis		1	
DQ553;Atresi af ductus deferens		1	
DQ554;Anden medfødt misdannelse af ductus deferens, epididymis, vesicula seminalis eller prostata		1	
DQ554A;Agenesi af funiculus spermaticus		1	
DQ554B;Agenesi af prostata		1	

DQ554C;Aplasi af funiculus spermaticus		1	
DQ554D;Aplasi af prostata		1	
DQ554E;Aplasi af ductus deferens		1	
DQ554F;Aplasi af vesicula seminalis		1	
DQ554G;Duplikation af ductus deferens		1	
DQ554H;Medfødt misdannelse af epididymidis UNS		1	
DQ554I;Medfødt misdannelse af prostata UNS		1	
DQ554J;Medfødt misdannelse af ductus deferens UNS		1	
DQ554K;Medfødt misdannelse af vesicula seminalis UNS		1	
DQ555;Agenesi eller aplasi af penis		1	
DQ555A;Agenesi af penis		1	
DQ555B;Aplasi af penis		1	
DQ556;Anden medfødt misdannelse af penis		1	
DQ556A;Atresi af penis		1	
DQ556B;Duplikation af penis		1	
DQ556C;Medfødt hypoplasi af penis		1	
DQ556D;Penis occultus		1	
DQ556E;Penoskrotal fusion eller transposition		1	
DQ558;Anden medfødt misdannelse af mandligt kønsorgan		1	
DQ558A;Sinus urogenitalis hos mand		1	
DQ559;Medfødt misdannelse i mandligt kønsorgan UNS		1	
DQ56;Interseksualitet og pseudohermafroditisme		1	
DQ560;Hermafroditisme IKA		1	
DQ560A;Ovotestis		1	
DQ561;Mandlig pseudohermafroditisme IKA		1	
DQ561A;Mandlig pseudohermafroditisme UNS		1	
DQ562;Kvindelig pseudohermafroditisme IKA		1	
DQ562B;Kvindelig pseudohermafroditisme UNS		1	
DQ563;Pseudohermafroditisme UNS		1	
DQ564;Ubestemmeligt køn UNS		1	
DQ60;Helt og delvist manglende nyre			2
DQ600;Enkeltsidig nyreagenesi		1	

Supplement 2 Nielsen et al. Mortality after paediatric emergency calls for patients with and without pre-existing comorbidity (2023)

DQ600A;Enkeltsidig nyreaplasi		1	
DQ601;Dobbeltsidig nyreagenesi			2
DQ601A;Dobbeltsidig nyreaplasi			2
DQ602;Nyreagenesi UNS		1	
DQ602A;Nyreaplasi UNS		1	
DQ603;Enkeltsidig nyrehypoplasi		1	
DQ604;Dobbeltsidig nyrehypoplasi		1	
DQ605;Nyrehypoplasi UNS		1	
DQ606;Potters syndrom			2
DQ61;Cystenyre		1	
DQ610;Medfødt nyrecyste		1	
DQ610A;Enkeltsidig nyrecyste		1	
DQ610B;Dobbeltsidig nyrecyste		1	
DQ611;Polycystisk nyresygdom med autosomal recessiv arvegang		1	
DQ612;Polycystisk nyresygdom med autosomal dominant arvegang		1	
DQ613;Polycystisk nyresygdom UNS		1	
DQ614;Cystisk renal dysplasi		1	
DQ615;Medullær svampenyre		1	
DQ618;Anden form for cystenyre		1	
DQ618A;Polycystisk lever- og nyresygdom		1	
DQ618B;Fibrocystisk nyre		1	
DQ619;Cystenyre UNS		1	
DQ619A;Meckel-Grubers syndrom		1	
DQ62;Medfødte afløbshindringer i nyrebækken og medfødte misdannelser i urinleder		1	
DQ620;Medfødt hydronefrose		1	
DQ620A;Enkeltsidig medfødt hydronefrose		1	
DQ620B;Dobbeltsidig medfødt hydronefrose		1	
DQ621;Medfødt atresi eller stenose af urinleder		1	
DQ621A;Atresi af urinleder		1	
DQ621B;Medfødt stenose af urinleder		1	
DQ621C;Stenosis congenita ureteris juxtapelvina		1	
DQ621D;Stenosis congenita ureteris juxtavesicalis		1	

DQ622;Medfødt dilatation af urinleder		1	
DQ623;Anden medfødt afløbshindring i nyrebækken eller urinleder		1	
DQ623A;Medfødt ureterocele		1	
DQ623B;Valvula ureteris		1	
DQ624;Agenesi af urinleder		1	
DQ624A;Aplasi af urinleder		1	
DQ625;Duplikation af urinleder		1	
DQ625A;Ureter accessorius		1	
DQ626;Malpositio ureteris		1	
DQ626A;Ektopisk urinleder		1	
DQ627;Medfødt vesikoureterorenal refluks		1	
DQ628;Anden medfødt misdannelse i urinleder		1	
DQ628A;Medfødt ureterdivertikel		1	
DQ628B;Medfødt ureterovaginal fistel		1	
DQ628C;Medfødt misdannelse i urinleder UNS		1	
DQ63;Andre medfødte misdannelser i nyre		1	
DQ630;Ren accessorius		1	
DQ631;Lobuleret nyre, kagenyre eller hesteskonyre		1	
DQ631A;Hesteskonyre		1	
DQ631B;Lobuleret nyre		1	
DQ632;Ektopisk nyre		1	
DQ632A;Malrotatio renis		1	
DQ633;Nyrehyperplasi		1	
DQ638;Anden medfødt nyremisdannelse		1	
DQ638A;Medfødt nyresten		1	
DQ638B;Dobbelt nyrebækken		1	
DQ638C;Ptosis renis congenita		1	
DQ638D;Medfødt calyxdivertikel		1	
DQ638E;Ren mobilis congenita		1	
DQ639;Medfødt misdannelse i nyre UNS		1	
DQ64;Andre medfødte misdannelser i urinveje		1	
DQ640;Epispadi		1	

DQ641;Ektropisk urinblære		1	
DQ642;Valvula urethrae posterior congenita		1	
DQ643;Anden atresi eller medfødt stenose i urinrør eller blærehals		1	
DQ643A;Blærehalsatresi		1	
DQ643B;Atresi af urinrør		1	
DQ643C;Medfødt stenose i blærehals		1	
DQ643D;Stenosis congenita meatus urethrae		1	
DQ643E;Stenosis congenita orificii vesicourethralis		1	
DQ643F;Medfødt stenose af urinrør		1	
DQ644;Medfødt misdannelse af urachus		1	
DQ644A;Medfødt urachuscyste		1	
DQ644B;Medfødt urachusfistel		1	
DQ644C;Urachusprolaps		1	
DQ644D;Urachus persistens		1	
DQ645;Agenesi eller aplasi af urinblære og urinrør		1	
DQ645A;Agenesi af urinrør		1	
DQ645B;Agenesi af urinblære		1	
DQ645C;Aplasi af urinrør		1	
DQ645D;Aplasi af urinblære		1	
DQ646;Medfødt blæredivertikel		1	
DQ647;Anden medfødt misdannelse af urinblære og urinrør		1	
DQ647A;Medfødt uretraldivertikel		1	
DQ647B;Duplikation af urinblære		1	
DQ647C;Medfødt rektouretral fistel		1	
DQ647D;Medfødt uretrovaginal fistel		1	
DQ647E;Medfødt uretralfistel		1	
DQ647G;Medfødt vesikorektal fistel		1	
DQ647H;Hernia vesicae urinariae congenita		1	
DQ647I;Kvindelig hypospadi		1	
DQ647J;Medfødt misdannelse af urinrør UNS		1	
DQ647K;Medfødt misdannelse af urinblære UNS		1	
DQ647L;Medfødt uretralprolaps		1	

DQ647M;Medfødt prolaps af urinblære		1	
DQ647N;Dobbelt urinrør		1	
DQ648;Anden medfødt misdannelse i urinveje		1	
DQ648A;Medfødt anuri		1	
DQ648B;Medfødt hydroureter		1	
DQ649;Medfødt misdannelse i urinveje UNS		1	
DQ65;Medfødte misdannelser i hofte		1	
DQ650;Medfødt enkeltsidig hofteluksation		1	
DQ651;Medfødt dobbeltsidig hofteluksation		1	
DQ652;Medfødt hofteluksation UNS		1	
DQ653;Medfødt enkeltsidig subluksation i hofte		1	
DQ654;Medfødt dobbeltsidig subluksation i hofterne		1	
DQ655;Medfødt subluksation i hofte UNS		1	
DQ656;Instabilt hofteled		1	
DQ658;Anden medfødt misdannelse i hofte		1	
DQ658A;Medfødt anteversion af collum femoris		1	
DQ658B;Coxa valga congenita		1	
DQ658C;Coxa vara congenita		1	
DQ658D;Medfødt dislokation af hofteled		1	
DQ658E;Dysplasia acetabuli congenita		1	
DQ658F;Hofteledsdysplasi		1	
DQ659;Medfødt misdannelse i hofte UNS		1	
DQ66;Medfødte misdannelser i fod		1	
DQ660;Talipes equinovarus		1	
DQ661;Talipes calcaneovarus		1	
DQ662;Metatarsus varus		1	
DQ663;Anden medfødt varusdeformitet i fod		1	
DQ663A;Hallux varus congenitus		1	
DQ663B;Klumpfod UNS		1	
DQ664;Talipes calcaneovalgus		1	
DQ665;Medfødt platfod		1	
DQ666;Anden medfødt valgusdeformitet i fod		1	

DQ666A;Hallux valgus congenitus		1	
DQ666B;Metatarsus valgus		1	
DQ666C;Pes equinovalgus		1	
DQ666D;Pes planovalgus		1	
DQ666E;Platfod UNS		1	
DQ667;Hulfod		1	
DQ668;Anden medfødt misdannelse i fod		1	
DQ668A;Coalitio tarsalis		1	
DQ668B;Medfødt hammertå		1	
DQ668C;Hallux flexus congenitus		1	
DQ668D;Hallux rigidus congenitus		1	
DQ668E;Megalodactylia digiti pedis		1	
DQ668F;Pes calcaneus		1	
DQ668G;Talipes asymmetricus		1	
DQ668H;Hælfod UNS		1	
DQ668I;Talus verticalis		1	
DQ669;Medfødt misdannelse i fod UNS		1	
DQ67;Medfødte misdannelser i knogler og muskler i hoved, ansigt, rygsøjle og brystkasse		1	
DQ670;Asymmetrisk ansigt		1	
DQ671;Compressio faciei		1	
DQ672;Dolikocefali		1	
DQ673;Plagiocefali		1	
DQ674;Anden medfødt misdannelse i kranie, ansigt eller kæbe		1	
DQ674A;Atrophia hemifaciei		1	
DQ674B;Depressio cranii congenita		1	
DQ674C;Medfødt deviation af næseseptum		1	
DQ674D;Halvsidig hypertrofi af kranie		1	
DQ674E;Halvsidig hypertrofi af ansigt		1	
DQ674F;Skafocefali		1	
DQ675;Medfødt deformitet af rygsøjle		1	
DQ675A;Medfødt kyfoskoliose UNS		1	
DQ675B;Medfødt postural skoliose		1	

DQ675C;Medfødt skoliose UNS		1	
DQ676;Tragtbryst		1	
DQ677;Fuglebryst		1	
DQ678;Anden medfødt misdannelse i brystkassens muskler eller skelet		1	
DQ678A;Medfødt misdannelse i brystkasse UNS		1	
DQ68;Andre medfødte misdannelser i muskler og knogler		1	
DQ680;Medfødt misdannelse af musculus sternocleidomastoideus		1	
DQ680A;Medfødt tortikollis		1	
DQ681;Medfødt misdannelse i hånd		1	
DQ681A;Medfødt springfinger		1	
DQ681B;Manus valga		1	
DQ681C;Manus vara		1	
DQ681D;Medfødt spadelignende hånd		1	
DQ681E;Medfødte trommestikfingre		1	
DQ682;Medfødt knædeformitet		1	
DQ682A;Genu recurvatum congenitum		1	
DQ682B;Medfødt knæluksation		1	
DQ683;Curvatura corporis femoris congenita		1	
DQ684;Curvatura tibiae et fibulae congenita		1	
DQ684A;Curvatura fibulae congenita		1	
DQ684B;Curvatura tibiae congenita		1	
DQ685;Medfødt abnorm krumning af lang rørknogle i underekstremitet UNS		1	
DQ688;Anden medfødt misdannelse i muskel eller knogle		1	
DQ688A;Cubitus valgus congenitus		1	
DQ688B;Cubitus varus congenitus		1	
DQ688C;Elevatio scapulae congenita		1	
DQ688D;Medfødt luksation i albueled		1	
DQ688E;Medfødt luksation i skulderled		1	
DQ688F;Medfødt misdannelse i underarm		1	
DQ688G;Medfødt misdannelse i clavicula		1	
DQ688H;Medfødt misdannelse i albueled		1	
DQ688I;Medfødt misdannelse i skulderblad		1	

Supplement 2 Nielsen et al. Mortality after paediatric emergency calls for patients with and without pre-existing comorbidity (2023)

DQ688J;Scapula alata congenita		1	
DQ69;Overtallige fingre og tæer		1	
DQ690;Overtallig finger		1	
DQ691;Overtallig tommelfinger		1	
DQ692;Overtallig tå		1	
DQ692A;Overtallig storetå		1	
DQ699;Polydaktyli UNS		1	
DQ70;Sammenvoksning af fingre og tæer		1	
DQ700;Komplet syndaktyli i hånd med synostose		1	
DQ701;Simpel syndaktyli i hånd uden synostose		1	
DQ702;Komplet syndaktyli i fod med synostose		1	
DQ703;Simpel syndaktyli i fod uden synostose		1	
DQ704;Polysyndaktyli		1	
DQ704A;Polysyndaktyli på hånd		1	
DQ704B;Polysyndaktyli på fod		1	
DQ709;Syndaktyli UNS		1	
DQ71;Mangelfuld udvikling af overekstremitet		1	
DQ710;Ameli af overekstremitet		1	
DQ710A;Amputatio congenita extremitatis superioris		1	
DQ710B;Aplasi af overekstremitet		1	
DQ711;Fokomeli af overekstremitet		1	
DQ712;Aplasi af både underarm og hånd		1	
DQ713;Aplasi af hånd eller fingre		1	
DQ713A;Aplasi af fingre		1	
DQ713A1;Aplasi af tommelfinger		1	
DQ713B;Aplasi af hånd		1	
DQ714;Longitudinel reduktionsdefekt af radius		1	
DQ715;Longitudinel reduktionsdefekt af ulna		1	
DQ716;Medfødt klohånd		1	
DQ718;Anden medfødt mangelfuld udvikling af overekstremitet		1	
DQ718A;Anisomelia congenita extremitatis superioris		1	
DQ718D;Aplasi af radius		1	

DQ718E;Aplasi af ulna		1		
DQ718F;Medfødt forkortning af overekstremitet		1		
DQ718G;Ektrodaktyli på overekstremitet		1		
DQ718H;Medfødt hypoplasi af radius		1		
DQ718I;Medfødt hypoplasi af ulna		1		
DQ718J;Micromelia extremitatis superioris		1		
DQ718K;Aplasi af underarm UNS		1		
DQ718L;Reductio humero		1		
DQ719;Medfødt mangelfuld udvikling af overekstremitet UNS		1		
DQ72;Mangelfuld udvikling af underekstremitet		1		
DQ720;Ameli af underekstremitet		1		
DQ720A;Amputatio congenita extremitatis inferioris		1		
DQ720B;Aplasi af underekstremitet		1		
DQ721;Fokomeli af underekstremitet		1		
DQ722;Aplasi af både underben og fod		1		
DQ723;Aplasi af fod eller tæer		1		
DQ723A;Aplasi af fod		1		
DQ724;Longitudinel reduktionsdefekt af femur		1		
DQ725;Longitudinel reduktionsdefekt af tibia		1		
DQ726;Longitudinel reduktionsdefekt af fibula		1		
DQ727;Splitfod		1		
DQ728;Anden medfødt mangelfuld udvikling af underekstremitet		1		
DQ728A;Anisomelia congenita extremitatis inferioris		1		
DQ728B;Aplasi af fibula		1		
DQ728C;Aplasi af storetå		1		
DQ728D;Aplasi af tåknogle		1		
DQ728E;Aplasi af tibia		1		
DQ728F;Medfødt forkortelse af underekstremitet		1		
DQ728G;Ektrodaktyli på underekstremitet		1		
DQ728H;Medfødt hypoplasi af fibula		1		
DQ728I;Medfødt hypoplasi af tibia		1		
DQ728J;Micromelia extremitatis inferioris		1		

Supplement 2 Nielsen et al. Mortality after paediatric emergency calls for patients with and without pre-existing comorbidity (2023)

DQ728K;Aplasi af underben		1	
DQ729;Medfødt mangelfuld udvikling af underekstremitet UNS		1	
DQ74;Andre medfødte misdannelser af arme og ben		1	
DQ740;Anden medfødt misdannelse af overekstremitet		1	
DQ740A;Deformatio congenita radii		1	
DQ740B;Dysostosis cleidocranialis		1	
DQ740D;Macrodactylia digiti manus		1	
DQ740G;Os carpi accessorium		1	
DQ740H;Medfødt pseudartrose i clavicula		1	
DQ740I;Dysostosis cleidocranialis		1	
DQ740J;Synostosis radio-ulnaris		1	
DQ740K;Triphalangismus digiti I manus		1	
DQ741;Medfødt misdannelse i knæ		1	
DQ741A;Aplasi af knæskal		1	
DQ741B;Medfødt valgusknæ		1	
DQ741C;Medfødt varusknæ		1	
DQ741D;Medfødt hypertrofi af femurkondyl		1	
DQ741E;Medfødt patellaluksation		1	
DQ741F;Medfødt misdannelse i knæ UNS		1	
DQ741G;Medfødt misdannelse af knæskal UNS		1	
DQ741H;Patella bipartita		1	
DQ741I;Patella rudimentaria		1	
DQ741J;Patella tripartita		1	
DQ742;Anden medfødt misdannelse i underekstremitet		1	
DQ742A;Ankylosis congenita sacroiliaca		1	
DQ742B;Macrodactylia digiti pedis		1	
DQ742C;Medfødt misdannelse i sakroiliakaled UNS		1	
DQ742D;Medfødt misdannelse i fodrodsben UNS		1	
DQ743;Arthrogryposis multiplex congenita		1	
DQ748;Anden medfødt misdannelse af ekstremitet(er)		1	
DQ748A;Anisomelia congenita		1	
DQ748B;Brachydactylia		1	

DQ748C;Ectrodactylia		1	
DQ748D;Gigantomelia		1	
DQ748E;Medfødt misdannelse i ekstremitetsknogle UNS		1	
DQ748E1;Medfødt kort ekstremitetsknogle		1	
DQ748F;Makrodaktyli UNS		1	
DQ749;Medfødt misdannelse af ekstremitet(er) UNS		1	
DQ75;Andre medfødte misdannelser af knogler i kranie og ansigt		1	
DQ750;Kraniosynostose		1	
DQ750C;Oxycefali		1	
DQ750D;Trigonocefali		1	
DQ751;Kraniofacial dysostose		1	
DQ751A;Cranioschisis		1	
DQ751B;Craniostenosis		1	
DQ751C;Crouzon's sygdom (findes ikke i SKS)		1	
DQ751D;Diastasis ossis cranialis congenita		1	
DQ752;Hypertelorisme		1	
DQ753;Makrocefali		1	
DQ754;Mandibulofacial dysostose		1	
DQ755;Okulomandibulær dysostose		1	
DQ758;Anden medfødt misdannelse af knogle i kranie eller ansigt		1	
DQ758A;Aplasi af kranieknogle		1	
DQ758B;Duplikation af processus frontonasalis		1	
DQ758C;Medfødt misdannelse af os frontale UNS		1	
DQ758D;Basilær invagination		1	
DQ758E;Mikrognati		1	
DQ759;Medfødt misdannelse af knogle i kranie eller ansigt UNS		1	
DQ76;Medfødte misdannelser i rygsøjle og brystkasse		1	
DQ760;Spina bifida occulta		1	
DQ761;Klippel-Feils syndrom		1	
DQ762;Medfødt spondylolistese		1	
DQ762A;Medfødt spondylolyse		1	
DQ763;Medfødt skoliose forårsaget af knoglemisdannelse		1	

DQ763A;Medfødt kyfoskoliose forårsaget af knoglemisdannelse		1	
DQ764;Anden medfødt misdannelse af rygsøjle uden samtidig skoliose		1	
DQ764A;Agenesi af haleben		1	
DQ764B;Agenesi af korsben		1	
DQ764C;Agenesi af ryghvirvel		1	
DQ764D;Aplasi af haleben		1	
DQ764E;Aplasi af korsben		1	
DQ764F;Aplasi af ryghvirvel		1	
DQ764G;Hemilumbalisatio ossis sacri		1	
DQ764H;Hemisacralisatio vertebrae lumbalis		1	
DQ764I;Hemispondylia congenita		1	
DQ764J;Hemivertebra		1	
DQ764K;Medfødt kyfose		1	
DQ764L;Medfødt lordose		1	
DQ764M;Lumbalisering		1	
DQ764N;Sakralisering		1	
DQ764O;Symphysis lumbosacralis abnormis		1	
DQ764P;Synostosis columnae vertebralis		1	
DQ764Q;Overtallig ryghvirvel		1	
DQ765;Halsribben		1	
DQ765A;Scalenus anterior-syndrom med halsribben		1	
DQ766;Anden medfødt misdannelse af ribben		1	
DQ766A;Agenesi af ribben		1	
DQ766B;Aplasi af ribben		1	
DQ766C;Costa accessoria		1	
DQ766D;Synostosis costae		1	
DQ767;Medfødt misdannelse af brystben		1	
DQ767A;Agenesi af brystben		1	
DQ767B;Aplasi af brystben		1	
DQ767C;Sternum bifidum		1	
DQ767D;Sternum recurvatum		1	
DQ768;Anden medfødt misdannelse i brystkassens knogler		1	

Supplement 2 Nielsen et al. Mortality after paediatric emergency calls for patients with and without pre-existing comorbidity (2023)

DQ769;Medfødt misdannelse i brystkassens knogler UNS		1	
DQ77;Mangelfuld vækst af knoglevæv og brusk i rørknogler og rygsøjle		1	
DQ770;Akondrogenese		1	
DQ770A;Hypokondrogenese		1	
DQ771;Tanatofor dværgvækst		1	
DQ772;Costa brevis-syndrom		1	
DQ772A;Dysplasia thoracica asphyxica		1	
DQ773;Chondrodysplasia punctata		1	
DQ773A;Dyschondroplasia cum haemangiomata		1	
DQ774;Akondroplasi		1	
DQ774A;Hypokondroplasi		1	
DQ774B;Medfødt osteosclerose		1	
DQ775;Dystrofisk dværgvækst		1	
DQ776;Kondroektodermal dysplasi		1	
DQ777;Spondyloepifyseal dysplasi		1	
DQ778;Anden form for osteokondrodysplasi i rørknogle eller rygsøjle		1	
DQ778A;Kondrodystrofisk dværgvækst		1	
DQ779;Osteokondrodysplasi i rørknogle eller rygsøjle UNS		1	
DQ78;Andre former for mangelfuld udvikling af knogle og brusk		1	
DQ780;Osteogenesis imperfecta		1	
DQ781;Polyostotisk fibrøs dysplasi		1	
DQ781A;McCune-Albright syndrom		1	
DQ782;Osteopetrose		1	
DQ782A;Osteopetrosis tarda		1	
DQ783;Progressiv diafyseal dysplasi		1	
DQ784;Enkondromatose		1	
DQ784A;Maffuccis syndrom		1	
DQ784B;Olliers sygdom		1	
DQ785;Metafyseal dysplasi		1	
DQ786;Medfødte multiple eksostoser		1	
DQ786A;Aclasis diaphysealis		1	
DQ788;Anden form for osteokondrodysplasi		1	

DQ788A;Caffeys syndrom		1	
DQ788B;Chondrodystrophia punctata		1	
DQ788C;Epifyseal dysplasi		1	
DQ788D;Exostosis congenita cartilaginis multiplex		1	
DQ788E;Hyperostosis congenita		1	
DQ788F;Osteochondritis multiplex		1	
DQ788G;Osteochondroma multiplex congenitum		1	
DQ788H;Osteopoikilose		1	
DQ788I;Generaliseret osteosklerose		1	
DQ788J;Rhachitis foetalis		1	
DQ788K;Osteodermatopoikilosis		1	
DQ788L;Buschke-Ollendorfs syndrom		1	
DQ789;Osteokondrodysplasi UNS		1	
DQ79;Medfødte misdannelser i muskler og knogler IKA		1	
DQ790;Medfødt diafragmahernie		1	
DQ791;Anden medfødt misdannelse af diafragma		1	
DQ791A;Aplasi af diafragma		1	
DQ791B;Eventratio diaphragmatica congenita		1	
DQ792;Omfalocele		1	
DQ793;Gastroskise		1	
DQ793A;Torakogastroskise		1	
DQ794;Sveskemavesyndrom		1	
DQ794A;Aplasi af bugmuskulatur		1	
DQ795;Anden medfødt misdannelse af bugvæg		1	
DQ796;Ehlers-Danlos' syndrom		1	
DQ798;Anden medfødt misdannelse i muskler og knogler		1	
DQ798A;Muskelagenesi UNS		1	
DQ798B;Knogleagenesi UNS		1	
DQ798C;Seneagenesi UNS		1	
DQ798D;Medfødt amyotrofi		1	
DQ798E;Ledaplasi UNS		1	
DQ798F;Muskelaplasi UNS		1	

DQ798G;Aplasi af musculus pectoralis		1	
DQ798H;Knogleaplasi UNS		1	
DQ798I;Seneaplasi UNS		1	
DQ798J;Medfødt ledbåndskontraktur		1	
DQ798K;Medfødt navlecyste		1	
DQ798L;Medfødt leddeformitet UNS		1	
DQ798M;Medfødt leddislokation UNS		1	
DQ798N;Elongatio ligamentum patellae		1	
DQ798O;Medfødt navlefistel		1	
DQ798P;Medfødt muskelhypoplasi UNS		1	
DQ798Q;Medfødt knoglehypoplasi UNS		1	
DQ798R;Musculus accessorius		1	
DQ798S;Polands syndrom		1	
DQ798T;Medfødt atlanto-occipital synostose		1	
DQ799;Medfødt misdannelse i muskel eller knogle UNS		1	
DQ80;Medfødt iktyose		1	
DQ800;Ichthyosis vulgaris		1	
DQ801;Kønsbunden ichthyosis		1	
DQ802;Ichthyosis lamellaris		1	
DQ803;Medfødt bulløs iktyosiform erytrodermi		1	
DQ804;Harlekinfoster		1	
DQ808;Anden form for medfødt iktyose		1	
DQ808A;Sjögren-Larssons syndrom		1	
DQ808B;Ichthyosis hystrix		1	
DQ808C;Ichthyosis circumflexa		1	
DQ808D;Keratitis ichtyosis deafness syndrom (KIDS)		1	
DQ809;Medfødt iktyosis UNS		1	
DQ81;Epidermolysis bullosa		1	
DQ810;Epidermolysis bullosa simplex		1	
DQ811;Epidermolysis bullosa letalis		1	
DQ812;Epidermolysis bullosa dystrophica		1	
DQ818;Anden form for epidermolysis bullosa		1	

Supplement 2 Nielsen et al. Mortality after paediatric emergency calls for patients with and without pre-existing comorbidity (2023)

DQ819;Epidermolysis bullosa UNS		1	
DQ82;Andre medfødte misdannelser af hud		1	
DQ820;Arveligt lymfødem		1	
DQ820A;Medfødt lymfødem i benene		1	
DQ820B;Milroys sygdom		1	
DQ821;Xeroderma pigmentosum		1	
DQ822;Mastocytose		1	
DQ822A;Urticaria pigmentosa		1	
DQ822B;Telangiectasia macularis eruptiva perstans		1	
DQ823;Incontinentia pigmenti		1	
DQ824;Ektodermal dysplasi		1	
DQ824A;Anhidrotisk ektodermal dysplasi		1	
DQ824B;Hidrotisk ektodermal dysplasi		1	
DQ824C;Hypohidrotisk ektodermal dysplasi		1	
DQ825;Medfødt ikke-neoplastisk nævus	0		
DQ825A;Medfødt godartet melanom	0		
DQ825B;Modermærke UNS	0		
DQ825E;Naevus flammeus	0		
DQ825F;Naevus sanguineus	0		
DQ825G;Vaskulært nævus UNS	0		
DQ825H;Naevus verrucosus	0		
DQ825I;Naevus systemicus	0		
DQ825K;Naevus sebaceus	0		
DQ825L;Naevus comedonicus	0		
DQ825M;Becker nævus	0		
DQ825N;Lineært epidermalt nævus	0		
DQ825P;Dysplastisk nævus syndrom	0		
DQ825Q;Jordbærmærke	0		
DQ828;Anden medfødt misdannelse af huden	0		
DQ8280;Medfødt misdannelse i lymfekarsystemet		1	
DQ8281;Periungvalt fibrom ved tuberøs sklerose		1	
DQ8282;Porokeratosis actinica superficialis		1	

DQ8283;Sebaceøst adenom ved tuberøs sklerose		1	
DQ8284;Keratosis palmoplantaris mutilans		1	
DQ8285;Keratosis follicularis spinolosus decalvans		1	
DQ8286;Medfødt poikiloderma vasculare atrophicans		1	
DQ8288;Michelinbaby		1	
DQ8289;Aquagenic wrinkling of the palms		1	
DQ828A;Medfødt akantose		1	
DQ828B;Angiokeratom		1	
DQ828C;Diffus hæmangiomatose hos nyfødt		1	
DQ828D;Cutis laxa hyperelastica		1	
DQ828E;Cutis verticis gyrata		1	
DQ828F;Dyskeratosis follicularis		1	
DQ828G;Elastom		1	
DQ828H;Medfødt elefantiasis		1	
DQ828I;Medfødt hyperkeratose		1	
DQ828J;Medfødt hyperkeratosis follicularis		1	
DQ828K;Keratoderma hereditarium		1	
DQ828L;Keratoderma palmare		1	
DQ828M;Keratoderma plantare		1	
DQ828N;Keratolysis exfoliativa		1	
DQ828P;Keratosis palmaris		1	
DQ828Q;Medfødt keratosis pilaris		1	
DQ828R;Angiokeratoma circumscriptum		1	
DQ828S;Lichen pilaris		1	
DQ828T;Lichen spinulosus		1	
DQ828U;Macula mongolica		1	
DQ828V;Mibellis angiokeratom		1	
DQ828W;Basalcellenævussyndrom		1	
DQ828X;Pemphigus benignus chronicus familiaris		1	
DQ828Y;Poikiloderma congenitum		1	
DQ828Z;Pseudoxanthoma elasticum		1	
DQ829;Medfødt misdannelse af huden UNS		1	

Supplement 2 Nielsen et al. Mortality after paediatric emergency calls for patients with and without pre-existing comorbidity (2023)

DQ829A;Rothmund-Thomsons syndrom		1	
DQ829B;Pigment mosaicisme		1	
DQ83;Medfødte misdannelser af mamma		1	
DQ830;Aplasi af både mamma og brystvorte		1	
DQ831;Mamma accessoria		1	
DQ832;Aplasi af brystvorte		1	
DQ833;Papilla mammae accessoria		1	
DQ838;Anden medfødt misdannelse af mamma		1	
DQ838A;Medfødt hypoplasi af mamma		1	
DQ838B;Tuberøse mammæ		1	
DQ839;Medfødt misdannelse af mamma UNS		1	
DQ84;Andre medfødte misdannelser af huddækket		1	
DQ840;Alopecia congenita		1	
DQ841;Medfødt morfologisk forstyrrelse i hårvæksten IKA		1	
DQ841A;Medfødt hypotrikose		1	
DQ841B;Monilethrix		1	
DQ841C;Pili annulati		1	
DQ841D;Pili torti		1	
DQ841E;Wooly hair		1	
DQ842;Anden medfødt misdannelse af hår		1	
DQ842A;Medfødt hypertrikose		1	
DQ842B;Lanugo persistens		1	
DQ843;Manglende negledannelse		1	
DQ844;Medfødt leukonyki		1	
DQ845;Hyperplasi eller hypertrofi af negle		1	
DQ845A;Neglehyperplasi		1	
DQ845B;Medfødt neglehypertrofi		1	
DQ845C;Onychauxis congenita		1	
DQ845D;Pachyonychia		1	
DQ846;Anden medfødt misdannelse af negle		1	
DQ846E;Medfødt negleatrofi		1	
DQ846F;Medfødt neglehypoplasi		1	

DQ846G;Medfødte skenegle		1	
DQ846H;Medfødt misdannelse af negle UNS		1	
DQ848;Anden medfødt misdannelse af huddækket		1	
DQ848A;Medfødt hudaplasi		1	
DQ849;Medfødt misdannelse af huddækket UNS		1	
DQ85;Tumoragtige dannelser på nervesystemet		1	
DQ850;Ikke-malign neurofibromatose		1	
DQ851;Tuberøs sklerose		1	
DQ858;Anden fakomatose IKA		1	
DQ858B;Peutz-Jeghers' syndrom		1	
DQ858C;Sturge-Webers-Krabbes syndrom		1	
DQ858D;von Hippel-Lindaus syndrom		1	
DQ859;Fakomatose UNS		1	
DQ86;Medfødte misdannelsessyndromer med kendte årsager IKA		1	
DQ860;Føtalt alkoholsyndrom		1	
DQ861;Føtalt hydantoinsyndrom		1	
DQ862;Dysmorfisme forårsaget af warfarin		1	
DQ863;Fetal alcohol spectrum disorder		1	
DQ868;Andet medfødt misdannelsessyndrom med kendt årsag		1	
DQ87;Andre specificerede medfødte misdannelsessyndromer i flere organsystemer		1	
DQ870;Syndromer med medfødte misdannelser overvejende i ansigtet		1	
DQ870A;Akrocefalopolysyndaktyli		1	
DQ870B;Akrocefalosyndaktyli type 1		1	
DQ870C;Kryptoftalmi		1	
DQ870D;Kyklopi		1	
DQ870F;Okulo-aurikulo-vertebral dysplasi		1	
DQ870G;Möbius' syndrom		1	
DQ870H;Oro-facio-digitalt syndrom		1	
DQ870I;Pierre Robins syndrom		1	
DQ870L;Treacher-Collin’s syndrom (findes ikke i SKS)		1	
DQ870M;Waardenburgs syndrom		1	
DQ870P;Oculodentodigital dysplasi		1	

Supplement 2 Nielsen et al. Mortality after paediatric emergency calls for patients with and without pre-existing comorbidity (2023)

DQ871;Medfødt misdannelsessyndrom med dværgvækst		1	
DQ871A;Cockaynes syndrom		1	
DQ871B;Cornelia de Langes syndrom		1	
DQ871C;Dubowitz' syndrom		1	
DQ871D;Noonans syndrom		1	
DQ871E;Prader-Willis syndrom		1	
DQ871F;Robinow-Silverman-Smiths syndrom		1	
DQ871G;Russell-Silvers syndrom		1	
DQ871H;Seckels syndrom		1	
DQ871I;Smith-Lemli-Opitz' syndrom		1	
DQ871J;Faciogenital dysplasi		1	
DQ872;Syndromer med medfødte misdannelser overvejende i ekstremiteter		1	
DQ872A;Atriodigital syndrom		1	
DQ872B;Klippel-Trenaunay-Webers syndrom		1	
DQ872C;Negl-patella-syndrom		1	
DQ872D;Rubinstein-Taybis syndrom		1	
DQ872E;Sirenomeli		1	
DQ872F;Trombocytopeni med aplasi af radius		1	
DQ872G;VACTERL syndrom		1	
DQ873;Syndromer med medfødte misdannelser med tidlig højdevækst		1	
DQ873A;Beckwith-Wiedemanns syndrom		1	
DQ873B;Sotos' syndrom		1	
DQ873C;Weavers syndrom		1	
DQ874;Marfans syndrom		1	
DQ874A;Araknodaktyli		1	
DQ875;Andet medfødt misdannelsessyndrom med andre skeletforandringer		1	
DQ878;Andet medfødt misdannelsessyndrom IKA		1	
DQ878A;Alports syndrom		1	
DQ878B;Laurence-Moon-Biedl-Bardets syndrom		1	
DQ878C;Zellwegers syndrom		1	
DQ878D;Cowdens sygdom		1	
DQ878E;Goltz syndrom		1	

DQ878H;Keratose palmoplantaris med parodontitis		1	
DQ878J;LEOPARD syndrom		1	
DQ878K;Birt-Hogg-Dube syndrom		1	
DQ89;Andre medfødte misdannelser IKA		1	
DQ890;Medfødt misdannelse i milten		1	
DQ890A;Medfødt manglende milt		1	
DQ890B;Lien supernumerarius		1	
DQ890C;Medfødt miltforstørrelse		1	
DQ891;Medfødt misdannelse i binyre		1	
DQ891A;Medfødt binyrecyste		1	
DQ892;Medfødt misdannelse i anden endokrin kirtel		1	
DQ892A;Thymusaplasi		1	
DQ892B;Cystis congenita ductus thyroglossalis		1	
DQ892C;Ductus thyroglossalis persistens		1	
DQ892D;Fistula congenita colli thyroglossalis		1	
DQ892E;Medfødt misdannelse i endokrin kirtel UNS		1	
DQ892F;Medfødt misdannelse i biskjoldbruskkirtel		1	
DQ892G;Medfødt misdannelse i hypofyse		1	
DQ892H;Medfødt misdannelse i skjoldbruskkirtel		1	
DQ892I;Medfødt misdannelse i thymus		1	
DQ893;Situs inversus		1	
DQ893A;Situs inversus abdominis		1	
DQ893B;Situs inversus cordis		1	
DQ893C;Situs inversus organorum partialis		1	
DQ893D;Situs inversus organorum totalis		1	
DQ893E;Situs inversus thoracalis		1	
DQ893F;Kartageners syndrom		1	
DQ894;Siamesiske tvillinger		1	
DQ894A;Kraniopagi		1	
DQ894B;Dicefali		1	
DQ894C;Diplopagi		1	
DQ894D;Pygopagi		1	

DQ894E;Thoracopagi		1	
DQ894F;Xiphopagi		1	
DQ897;Multiple medfødte misdannelser IKA			2
DQ897A;Multiple medfødte misdannelser UNS			2
DQ898;Anden medfødt misdannelse		1	
DQ898A;Hemigigantismus congenitus		1	
DQ898B;Hemihypertrophia congenita		1	
DQ898T;Tumor hos foster UNS		1	
DQ898T1;Sakralt teratom hos foster		1	
DQ898T2;Teratom hos foster		1	
DQ898T3;Lymfangiom hos foster		1	
DQ898T4;Nakkehygrom hos foster		1	
DQ899;Medfødt misdannelse UNS		1	
DQ90;Downs syndrom		1	
DQ900;Trisomi 21, meiotisk nondisjunktion		1	
DQ901;Trisomi 21, mosaik, mitotisk nondisjunktion		1	
DQ901A;Trisomi 21-22, mosaik, mitotisk nondisjunktion		1	
DQ902;Trisomi 21, translokation		1	
DQ902A;Trisomi 21-22, translokation		1	
DQ909;Downs syndrom UNS		1	
DQ909A;Trisomi 13-21, translokation			2
DQ91;Edwards og Pataus syndromer			2
DQ910;Trisomi 18, meiotisk nondisjunktion			2
DQ910A;Trisomi 17-18, meiotisk nondisjunktion			2
DQ911;Trisomi 18, mosaik mitotisk nondisjunktion			2
DQ911A;Trisomi 17-18, mosaik mitotisk nondisjunktion			2
DQ912;Trisomi 18, translokation			2
DQ912A;Trisomi 17-18, translokation			2
DQ913;Edwards' syndrom UNS			2
DQ913A;Trisomi 17-18 UNS			2
DQ914;Trisomi 13, meiotisk nondisjunktion			2
DQ914A;Trisomi 13-15, meiotisk nondisjunktion			2

Supplement 2 Nielsen et al. Mortality after paediatric emergency calls for patients with and without pre-existing comorbidity (2023)

DQ915;Trisomi 13, mosaik mitotisk nondisjunktion			2
DQ915A;Trisomi 13-15, mosaik mitotisk nondisjunktion			2
DQ916;Trisomi 13, translokation			2
DQ916A;Trisomi 13-15, translokation			2
DQ917;Pataus syndrom UNS			2
DQ917A;Trisomi 13-15 UNS			2
DQ917B;Trisomi 14		1	
DQ92;Andre trisomier og partielle trisomier i autosomale kromosomer IKA		1	
DQ920;Autosomal trisomi, meiotisk nondisjunktion		1	
DQ921;Autosomal trisomi, mosaik mitotisk nondisjunktion		1	
DQ922;Større partiel autosomal trisomi		1	
DQ923;Lille partiel autosomal trisomi		1	
DQ924;Kromosomduplikation kun synlig i prometafase		1	
DQ924A;Trisomia autosomalis, duplikation kun synlig i prometafase		1	
DQ925;Autosomal kromosomduplikation med anden kompleks ombytning		1	
DQ926;Ekstra markørkromosom		1	
DQ927;Triploidi eller polyploidi			2
DQ927A;Polyploidi			2
DQ927B;Triploidi			2
DQ928;Anden hel eller partiel autosomal trisomi			2
DQ929;Hel eller partiel autosomal trisomi UNS			2
DQ93;Autosomale deletioner og monosomier IKA			2
DQ930;Autosomal monosomi, meiotisk nondisjunktion			2
DQ931;Autosomal monosomi, mosaik mitotisk nondisjunktion			2
DQ932;Kromosom erstattet af ring eller dicentrisk kromosom			2
DQ932A;Dicentrisk kromosom, monosomi			2
DQ932B;Kromosom erstattet af ring, monosomi			2
DQ933;Deletion af kromosom 4, kort arm			2
DQ934;Deletion af kromosom 5, kort arm			2
DQ934A;Cri-du-chat syndrom (findes ikke i SKS)			2
DQ935;Anden partiel deletion af et kromosom			2
DQ935B;Partiel translokation 4-5			2

Supplement 2 Nielsen et al. Mortality after paediatric emergency calls for patients with and without pre-existing comorbidity (2023)

DQ935C;Angelmans syndrom			2
DQ936;Deletion kun synlig i prometafase		1	
DQ937;Deletion med anden kompleks ombytning		1	
DQ938;Anden autosomal kromosomdeletion		1	
DQ938A;Williams' syndrom		1	
DQ939;Autosomal kromosomdeletion UNS		1	
DQ95;Balancerede ombytninger i kromosomer og kromosommarkører IKA		1	
DQ950;Balanceret translokation eller insertion med normal fænotype		1	
DQ950A;Balanceret insertion med normal fænotype		1	
DQ950B;Balanceret translokation med normal fænotype		1	
DQ951;Inversion med normal fænotype		1	
DQ952;Balanceret ombytning i autosomale kromosomer med abnorm fænotype		1	
DQ953;Balanceret ombytning i kønskromosomer eller autosomale kromosomer med abnorm fænotype		1	
DQ954;Heterokromatinmarkør		1	
DQ955;Autosomalt kromosom med fragilt sted		1	
DQ958;Anden balanceret ombytning i kromosom eller kromosommarkør		1	
DQ959;Balanceret ombytning i kromosom eller kromosommarkør UNS		1	
DQ96;Turners syndrom		1	
DQ960;Turners syndrom karyotype 45,X		1	
DQ961;Turners syndrom karyotype 46,X iso (Xq)		1	
DQ962;Turners syndrom med andet abnormt kønskromosom		1	
DQ963;Turners syndrom mosaik, 45,X/46,XX eller XY		1	
DQ963A;Turners syndrom mosaik, 45,X/46,XX		1	
DQ963B;Turners syndrom mosaik, 45,X/46,XY		1	
DQ964;Turners syndrom mosaik, 45,X/anden cellelinje(r) med abnormt kønskromosom		1	
DQ968;Anden form for Turners syndrom		1	
DQ969;Turners syndrom UNS		1	
DQ97;Andre kønskromosomanomalier med kvindelig fænotype IKA		1	
DQ970;Kønskromosomanomali karyotype 47,XXX		1	
DQ971;Kvinde med flere end tre X-kromosomer		1	
DQ972;Kønskromosomanomali med mosaik, cellelinjer med forskelligt antal X-kromosomer		1	
DQ973;Kønskromosomanomali karyotype 46,XY med kvindelig fænotype		1	

Supplement 2 Nielsen et al. Mortality after paediatric emergency calls for patients with and without pre-existing comorbidity (2023)

DQ978;Anden kønskromosomanomali med kvindelig fænotype		1	
DQ979;Kønskromosomanomali med kvindelig fænotype UNS		1	
DQ98;Andre kønskromosomanomalier med mandlig fænotype IKA		1	
DQ980;Klinefelters syndrom, karyotype 47,XXY		1	
DQ981;Klinefelters syndrom hos mand med flere end to X-kromosomer		1	
DQ982;Klinefelters syndrom hos mand med karyotype 46,XX		1	
DQ983;Mand med karyotype 46,XX uden Klinefelters syndrom		1	
DQ984;Klinefelters syndrom UNS		1	
DQ985;Kønskromosomanomali, karyotype 47,XYY		1	
DQ986;Mand med strukturelt abnormt kønskromosom		1	
DQ987;Mand med kønskromosommosaik		1	
DQ988;Anden kønskromosomanomali med mandlig fænotype		1	
DQ988A;Mand med YO-syndrom		1	
DQ988B;Mand med YY-syndrom		1	
DQ989;Kønskromosomanomali med mandlig fænotype UNS		1	
DQ99;Andre kønskromosomanomalier IKA		1	
DQ990;Kimære, karyotype 46,XX/46,XY		1	
DQ991;Ægte hermafrodit, karyotype 46,XX		1	
DQ991A;Ægte gonadedysgenesi		1	
DQ991C;Ægte hermafrodit, karyotype 46,XY		1	
DQ992;Fragilt X-kromosom		1	
DQ998;Anden kromosomanomali		1	
DQ999;Kromosomanomali UNS		1	
DR00;Abnorm hjerterytme	0		
DR000;Takykardi UNS	0		
DR001;Bradykardi UNS	0		
DR002;Hjertebanken	0		
DR008;Anden eller ikke specificeret abnorm hjerterytme	0		
DR008A;Hjertearytmi UNS	0		
DR01;Mislyd ved hjertet	0		
DR010;Funktionel mislyd ved hjertet	0		
DR011;Mislyd ved hjertet UNS	0		

Supplement 2 Nielsen et al. Mortality after paediatric emergency calls for patients with and without pre-existing comorbidity (2023)

DR012;Anden hjertelyd	0			
DR012A;Perikardiel gnidningslyd	0			
DR012B;Øget hjertedæmpning	0			
DR02;Koldbrand IKA	0			
DR029;Gangræn IKA	0			
DR03;Abnormt blodtryk uden diagnose	0			
DR030;Forhøjet blodtryk uden verificeret hypertension	0			
DR030A;Forbigående blodtryksforhøjelse	0			
DR030B;White coat-hypertension	0			
DR031;Lavt blodtryk UNS	0			
DR04;Blødning fra luftveje	0			
DR040;Næseblod	0			
DR040A;Epistaxis anterior	0			
DR040B;Epistaxis posterior	0			
DR040C;Epistaxis recidivans	0			
DR041;Blødning fra svælg	0			
DR042;Hæmoptyse	0			
DR048;Blødning fra anden lokalisation i luftveje	0			
DR048A;Blødning fra lunger	0			
DR049;Blødning fra luftvejene UNS	0			
DR05;Hoste	0			
DR057;Uforklaret eller refraktær kronisk hoste	0			
DR059;Hoste UNS	0			
DR06;Abnorm vejrtrækning	0			
DR060;Dyspnø	0			
DR060A;Ortopnø	0			
DR061;Stridor	0			
DR062;Piben på lungerne	0			
DR063;Cheyne-Stokes respiration	0			
DR064;Hyperventilation	0			
DR065;Mundånding	0			
DR065A;Snorken	0			

DR066;Hikke	0			
DR067;Nysen	0			
DR068;Anden eller ikke specificeret abnorm vejrtrækning	0			
DR068A;Respiratio paradoxa	0			
DR068B;Sukken	0			
DR068C;Kvælningsfornemmelse	0			
DR068D;Apnø UNS	0			
DR068E;Gispen	0			
DR07;Smerter i hals og bryst	0			
DR070;Smerter i svælg	0			
DR071;Smerter i thorax ved vejrtrækning	0			
DR072;Prækordialsmerter	0			
DR073;Andre brystsmerter	0			
DR074;Brystsmerter UNS	0			
DR079;Brystsmerter uden specifikation (findes ikke i SKS)	0			
DR09;Andre symptomer og fund i kredsløbs- og åndedrætsorganer	0			
DR090;Asfyksi	0			
DR091;Lungehindebetændelse IKA	0			
DR092;Respirationsstop	0			
DR092A;Hjerte-lunge-svigt UNS	0			
DR093;Abnormt opspyt	0			
DR093A;Sputum excessivum	0			
DR093B;Purulent opspyt	0			
DR098;Andet specificeret symptom eller fund i kredsløbs- og åndedrætsorganerne	0			
DR098A;Abnorm perkussion af thorax	0			
DR098B;Krepitation ved lungestetoskopi	0			
DR098C;Pulsus parvus	0			
DR098D;Stenosemislyd over arterie	0			
DR098E;Tympanitisk perkussionslyd over thorax	0			
DR098F;Kredsløbssymptom UNS	0			
DR10;Smerter i bughule og bækken	0			
DR100;Akutte mavesmerter	0			

Supplement 2 Nielsen et al. Mortality after paediatric emergency calls for patients with and without pre-existing comorbidity (2023)

DR100A;Defense ved akut abdomen	0			
DR101;Mavesmerter lokaliseret til øvre abdomen	0			
DR102;Mavesmerter lokaliseret til bækken og bækkenbund	0			
DR102A;Mavesmerter lokaliseret til perineum	0			
DR102B;Mavesmerter lokaliseret til bækken	0			
DR102C;Mavesmerter lokaliseret til nedre abdomen	0			
DR103;Mavesmerter lokaliseret til anden del af nedre abdomen	0			
DR103A;Defækationssmerter	0			
DR104;Andre eller ikke specificerede mavesmerter	0			
DR104A;Spædbarnskolik	0			
DR104B;Mavekolik UNS	0			
DR108;Abdominalia, anden og ikke specificeret (findes ikke i SKS)	0			
DR11;Kvalme og opkastning	0			
DR119;Kvalme og opkastning	0			
DR119B;Kvalme	0			
DR119C;Opkastning	0			
DR12;Halsbrand	0			
DR129;Halsbrand UNS	0			
DR13;Synkebesvær	0			
DR131;Søvnrelateret synken, hosten eller laryngospasme	0			
DR131A;Søvnrelateret synkebesvær	0			
DR131B;Søvnrelateret laryngospasme	0			
DR139;Synkebesvær UNS	0			
DR14;Øget tarmluft og lignende tilstande	0			
DR149;Flatulens eller lignende tilstand UNS	0			
DR149A;Flatulens	0			
DR15;Ufrivillig afgang af afføring	0			
DR159;Afføringsinkontinens	0			
DR16;Forstørret lever og milt IKA	0			
DR160;Forstørret lever IKA	0			
DR160A;Hepatomegali UNS	0			
DR161;Forstørret milt IKA	0			

Supplement 2 Nielsen et al. Mortality after paediatric emergency calls for patients with and without pre-existing comorbidity (2023)

DR161A;Splenomegali UNS	0			
DR162;Hepatomegali med splenomegali IKA	0			
DR162B;Hepatosplenomegali UNS	0			
DR17;Hyperbilirubinæmi med eller uden gulsot IKA	0			
DR170;Hyperbilirubinæmi med ikterus IKA	0			
DR179;Hyperbilirubinæmi uden ikterus IKA	0			
DR18;Ascites	0			
DR189;Ascites UNS	0			
DR19;Andre symptomer og abnorme fund i fordøjelsessystem og abdomen	0			
DR190;Udfyldning i abdomen eller bækken UNS	0			
DR190A;Udfyldning i bækken UNS	0			
DR190B;Navleudfyldning UNS	0			
DR190C;Udfyldning i abdomen UNS	0			
DR191;Abnorme tarmlyde	0			
DR191B;Tyst abdomen	0			
DR192;Tarmrejsning	0			
DR193;Défense i bugvæg UNS	0			
DR194;Ændret afføringsmønster	0			
DR195;Abnorm afføring UNS	0			
DR196;Dårlig ånde	0			
DR196A;Foetor ex ore (findes ikke i SKS)	0			
DR198;Andet symptom eller abnormt fund i fordøjelsessystem eller abdomen	0			
DR198A;Opstød	0			
DR198C;Meteorismus	0			
DR198D;Ruminatio	0			
DR198E;Tenesmi rectalis	0			
DR198F;Tenesmi UNS	0			
DR198G;Burning mouth syndrom	0			
DR20;Føleforstyrrelser i huden	0			
DR200;Manglende følelse i huden	0			
DR201;Nedsat følelse i huden	0			
DR202;Unormal følefornemmelse i huden	0			

Supplement 2 Nielsen et al. Mortality after paediatric emergency calls for patients with and without pre-existing comorbidity (2023)

DR203;Hyperæstesi i huden	0		
DR208;Anden eller ikke specificeret føleforstyrrelse i huden	0		
DR208A;Æstesiodermi UNS	0		
DR21;Ikke nærmere specificeret udslæt på huden	0		
DR219;Hududslæt UNS	0		
DR219A;Eruptio cutanea	0		
DR22;Lokaliserede hævelser, udfyldninger og knuder i hud og underhud	0		
DR220;Hævelse, udfyldning eller knude i hud eller underhud på hoved	0		
DR221;Hævelse, udfyldning eller knude i hud eller underhud på hals	0		
DR222;Hævelse, udfyldning eller knude i hud eller underhud på kroppen	0		
DR223;Hævelse, udfyldning eller knude i hud eller underhud på overekstremitet	0		
DR224;Hævelse, udfyldning eller knude i hud eller underhud på underekstremitet	0		
DR227;Hævelse, udfyldning eller knude i hud eller underhud på flere lokalisationer	0		
DR229;Hævelse, udfyldning eller knude i hud eller underhud UNS	0		
DR23;Andre hudforandringer	0		
DR230;Cyanose	0		
DR231;Bleghed	0		
DR232;Rødmen	0		
DR233;Spontane ekkymoser	0		
DR233A;Spontane petekkier	0		
DR234;Ændringer i hudstrukturen	0		
DR234A;Induration af huden	0		
DR234B;Skællende hud	0		
DR238;Anden eller ikke specificeret hudforandring	0		
DR238A;Hudforandring UNS	0		
DR238B;Cutis marmorata	0		
DR25;Abnorme ufrivillige bevægelser	0		
DR250;Abnorme hovedbevægelser	0		
DR251;Tremor UNS	0		
DR252;Kramper eller spasmer	0		
DR252A;Kramper UNS	0		
DR252B;Spasmer UNS	0		

Supplement 2 Nielsen et al. Mortality after paediatric emergency calls for patients with and without pre-existing comorbidity (2023)

DR252C;Muskelspasmer UNS	0			
DR253;Fascikulationer	0			
DR258;Anden eller ikke specificeret abnorm ufrivillig bevægelse	0			
DR258A;Myokloni	0			
DR258B;Klonus	0			
DR258C;Indsovningsmyoklonier	0			
DR258D;Benign neonatal myokloni	0			
DR258E;Søvnrelateret fodtremor	0			
DR258F;Søvnrelateret propriospinal myokloni	0			
DR258G;Søvnrelateret fragmenteret myokloni	0			
DR26;Gangbesvær og mobilitetsforstyrrelser	0			
DR260;Ataktisk gang	0			
DR261;Paralytisk gang	0			
DR262;Gangbesvær IKA	0			
DR268;Andet eller ikke specificeret gangbesvær eller mobilitetsforstyrrelse	0			
DR268B;Grovmotorisk dyspraksi	0			
DR268C;Andet gangbesvær	0			
DR268D;Usikkerhed på benene UNS	0			
DR268E;Mobilitetsforstyrrelse UNS	0			
DR268F;Gangbesvær UNS	0			
DR27;Andre koordinationsforstyrrelser	0			
DR270;Ataksi UNS	0			
DR278;Anden eller ikke specificeret koordinationsforstyrrelse	0			
DR278A;Koordinationsforstyrrelse UNS	0			
DR29;Andre symptomer og abnorme fund i nervesystemet og bevægeapparat	0			
DR290;Tetani	0			
DR290A;Karpopedalspasme	0			
DR291;Meningisme	0			
DR292;Abnorme reflekser	0			
DR293;Abnorm kropsholdning	0			
DR294;Hofteklik	0			
DR296;Faldtendens IKA	0			

Supplement 2 Nielsen et al. Mortality after paediatric emergency calls for patients with and without pre-existing comorbidity (2023)

DR298;Andet eller ikke specificeret symptom eller abnormt fund i nerve- og bevægelsessystemet	0			
DR298A;Symptom i nervesystemet UNS	0			
DR298B;Søvnrelaterede hallucinationer	0			
DR298C;Talen i søvne	0			
DR298D;Lang søvntid	0			
DR298E;Kort søvntid	0			
DR30;Smerter ved vandladning	0			
DR300;Dysuri	0			
DR301;Tenesmi vesicalis	0			
DR309;Smerter ved vandladning UNS	0			
DR31;Blod i urinen uden nærmere specificering	0			
DR319;Hæmaturi UNS	0			
DR319A;Makroskopisk hæmaturi UNS	0			
DR319B;Mikroskopisk hæmaturi UNS	0			
DR32;Urininkontinens uden nærmere specificering	0			
DR329;Urininkontinens UNS	0			
DR329A;Enuresis nocturna UNS	0			
DR329B;Enuresis diurna UNS	0			
DR33;Urinretention	0			
DR339;Urinretention UNS	0			
DR339A;Ischuria paradoxa	0			
DR339B;Akut urinretention	0			
DR339C;Kronisk urinretention	0			
DR339D;Residualurin	0			
DR34;Sparsom og ophævet diurese	0			
DR349;Anuri eller oliguri	0			
DR349A;Anuri	0			
DR349B;Oligouri	0			
DR35;Stor diurese	0			
DR359;Polyuri UNS	0			
DR359A;Nokturn polyuri	0			
DR359B;Pollakisuri	0			

Supplement 2 Nielsen et al. Mortality after paediatric emergency calls for patients with and without pre-existing comorbidity (2023)

DR36;Udflåd fra urinrøret	0			
DR369;Udflåd fra urinrøret UNS	0			
DR39;Andre symptomer og abnorme fund fra urinvejene	0			
DR390;Extravasatio urinae	0			
DR391;Vandladningsbesvær UNS	0			
DR391A;Igangsætningsbesvær ved vandladning	0			
DR391B;Slap stråle ved vandladning	0			
DR391C;Spredt stråle ved vandladning	0			
DR391D;Imperiøs vandladning	0			
DR391E;Nykturi (findes ikke i SKS)	0			
DR392;Ekstrarenal uræmi	0			
DR392A;Prærenal uræmi	0			
DR392B;Postrenal uræmi	0			
DR398;Andet eller ikke specificeret symptom eller abnormt fund i urinvejene	0			
DR398A;Efterdryp	0			
DR398B;Infravesikal obstruktion	0			
DR398C;Prostatodyni	0			
DR398D;Pneumaturi	0			
DR398E;Abnormt prostata palpationsfund	0			
DR40;Bevidsthedspåvirkning	0			
DR400;Somnolens	0			
DR401;Stupor	0			
DR402;Koma UNS	0			
DR403;Persisterende vegetativ tilstand	0			
DR41;Andre symptomer og abnorme fund vedrørende erkendelsesevne	0			
DR410;Konfusion UNS	0			
DR411;Anterograd amnesi	0			
DR412;Retrograd amnesi	0			
DR413;Anden amnesi	0			
DR413A;Amnesi UNS	0			
DR418;Andet eller ikke specificeret symptom eller abnormt fund vedrørende erkendelsesevne	0			
DR42;Svimmelhed	0			

DR429;Vertigo UNS	0			
DR43;Lugte- og smagsforstyrrelser	0			
DR430;Manglende lugtesans	0			
DR431;Parosmi	0			
DR432;Forandret smagsoplevelse	0			
DR438;Anden eller ikke specificeret lugte- eller smagsforstyrrelse	0			
DR438A;Manglende smagssans	0			
DR438B;Smagsforstyrrelse UNS	0			
DR438C;Lugteforstyrrelse UNS	0			
DR44;Andre symptomer og abnorme fund vedrørende opfattelsesevnen	0			
DR440;Hørehallucinationer	0			
DR441;Synshallucinationer	0			
DR442;Andre hallucinationer	0			
DR443;Hallucinationer UNS	0			
DR448;Andet symptom eller abnormt fund vedrørende opfattelsesevnen	0			
DR45;Symptomer og abnorme fund vedrørende stemningslejet	0			
DR450;Nervøsitet	0			
DR451;Rastløs eller urolig	0			
DR451A;Rastløs	0			
DR451B;Urolig	0			
DR452;Ulykkelig eller bekymret	0			
DR452A;Ulykkelig	0			
DR452B;Bekymret	0			
DR453;Apatisk eller demoraliseret	0			
DR453A;Apatisk	0			
DR453B;Demoraliseret	0			
DR454;Irritabel eller vredladen	0			
DR454A;Irritabel	0			
DR454B;Vredladen	0			
DR455;Fjendtlig	0			
DR456;Voldelig	0			
DR457;Følelsesmæssig shocktilstand UNS	0			

Supplement 2 Nielsen et al. Mortality after paediatric emergency calls for patients with and without pre-existing comorbidity (2023)

DR458;Andet symptom eller abnormt fund vedrørende stemningslejet	0		
DR458A;Patologisk sorgreaktion	0		
DR46;Symptomer og abnorme fund vedrørende udseende og opførsel	0		
DR460;Dårlig personlig hygiejne	0		
DR461;Bizart udseende	0		
DR462;Mærkværdig eller uforklarlig opførsel	0		
DR463;Hyperaktiv	0		
DR464;Sløv med nedsat reaktion	0		
DR465;Mistænksom eller undvigende	0		
DR465A;Mistænksom	0		
DR465B;Undvigende	0		
DR466;Overdreven bekymring eller optagethed af belastende oplevelser	0		
DR467;Usædvanlig snakkesalig eller omstændelig uden at komme til sagen	0		
DR468;Andet symptom eller abnormt fund vedrørendse udseende og opførsel	0		
DR47;Taleforstyrrelser IKA	0		
DR470;Dysfasi eller afasi	0		
DR470A;Dysfasi	0		
DR470B;Afasi	0		
DR471;Dysartri eller anartri	0		
DR471A;Dysartri	0		
DR471B;Anartri	0		
DR478;Anden eller ikke specificeret taleforstyrrelse	0		
DR48;Ordblindhed og andre indlæringsforstyrrelser	0		
DR480;Dysleksia eller aleksi	0		
DR480A;Ordblindhed	0		
DR480C;Aleksi	0		
DR481;Agnosi	0		
DR482;Apraksi	0		
DR488;Anden eller ikke specificeret indlæringsforstyrrelse	0		
DR488A;Akalkuli IKA	0		
DR488B;Agrafi IKA	0		
DR488C;Astereognose	0		

DR488D;Dysgrafi	0			
DR49;Forstyrrelser i stemmeføring	0			
DR490;Dysfoni	0			
DR490A;Hæshed	0			
DR491;Afoni	0			
DR492;Snøvlen	0			
DR498;Anden eller ikke specificeret forstyrrelse i stemmeføring	0			
DR498A;Fonasteni	0			
DR498B;Stemmeændring UNS	0			
DR50;Feber af anden og ukendt årsag	0			
DR502;Feber forårsaget af lægemiddel	0			
DR508;Anden feber	0			
DR508A;Feber med kulderystelser	0			
DR508B;Vedvarende feber af ukendt årsag	0			
DR509;Feber UNS	0			
DR51;Hovedpine	0			
DR519;Hovedpine UNS	0			
DR52;Smerter IKA	0			
DR520;Akutte smerter	0			
DR521;Maligne smerter	0			
DR521A;Simple maligne smerter	0			
DR521B;Komplekse maligne smerter	0			
DR522;Andre kroniske smerter	0			
DR522A;Kroniske smerter UNS	0			
DR522B;Smerter i kønsorganer	0			
DR522C;Burning vulva-penis-scrotum-anus syndrom	0			
DR522D;Simple langvarige eller kroniske ikke-maligne smerter	0			
DR522E;Komplekse langvarige eller kroniske ikke-maligne smerter	0			
DR529;Smerter UNS	0			
DR53;Utilpashed og træthed	0			
DR539;Utilpashed eller udmattelse UNS	0			
DR539A;Udmattelse	0			

DR539C;Letargi	0		
DR539E;Sygdomsfølelse	0		
DR539F;Utilpashed	0		
DR54;Alderdomssvækkelse	0		
DR549;Senilitet	0		
DR549A;Senil debilitet	0		
DR549B;Senil asteni	0		
DR55;Besvimelse og kollaps	0		
DR559;Besvimelse eller kollaps	0		
DR559A;Vasovagalt anfald	0		
DR559B;Lipotymi UNS	0		
DR559C;Infantilt kollaps	0		
DR559D;Kollaps UNS	0		
DR56;Konvulsioner IKA	0		
DR560;Feberkramper	0		
DR560A;Tetani ved feber	0		
DR568;Andre eller ikke specificerede konvulsioner	0		
DR568C;Affektkramper	0		
DR568D;Konvulsioner UNS	0		
DR568E;Førstegangs uprovokeret generaliseret tonisk-klonisk anfald (GTCS)	0		
DR568F;Oligoepilepsi	0		
DR568G;Non-epileptiske anfald	0		
DR57;Shock IKA	0		
DR570;Kardiogent shock	0		
DR571;Hypovolæmisk shock	0		
DR572;Septisk shock	0		
DR578;Anden form for shock	0		
DR578A;Endotoksisk shock	0		
DR579;Shock UNS	0		
DR58;Blødning IKA	0		
DR589;Blødning UNS	0		
DR59;Forstørrede lymfeknuder	0		

DR590;Lokaliseret lymfeknudeforstørrelse	0		
DR591;Universel lymfeknudeforstørrelse	0		
DR599;Lymfeknudeforstørrelse UNS	0		
DR60;Ødem IKA	0		
DR600;Lokaliseret ødem	0		
DR601;Universelt ødem	0		
DR609;Ødem UNS	0		
DR61;Øget svedsekretion	0		
DR610;Lokaliseret hyperhidrose	0		
DR610A;Pitted keratolyse	0		
DR611;Universel hyperhidrose	0		
DR619;Hyperhidrose UNS	0		
DR619A;Nattesved	0		
DR62;Forsinket fysiologisk udvikling	0		
DR620;Retarderet psykomotorisk udvikling	0		
DR620A;Taleretarderet	0		
DR620B;Sprogretarderet	0		
DR620C;Motorisk retarderet	0		
DR628;Anden form for retarderet fysiologisk udvikling	0		
DR628A;Dårlig trivsel	0		
DR628B;Infantilisme UNS	0		
DR628C;Vækstretarderet	0		
DR629;Retarderet fysiologisk udvikling UNS	0		
DR63;Symptomer og abnorme fund vedrørende føde- og væskeindtagelse	0		
DR630;Appetitløshed	0		
DR631;Polydipsia	0		
DR632;Polyfagi	0		
DR633;Anden form for spisevanskelighed	0		
DR633A;Spisevanskelighed UNS	0		
DR634;Abnormt vægttab	0		
DR635;Abnorm vægtstigning	0		
DR638;Symptomer eller abnorme fund vedrørende føde- og væskeindtagelse UNS	0		

DR64;Kakeksi	0		
DR649;Kakeksi UNS	0		
DR67;Fund ved vurdering af generel funktionsevne	0		
DR670;Selvhjulpen eller lettere afhængig	0		
DR670A;Selvhjulpen eller lettere afhængig, Barthel indeks 100: 80-100 point	0		
DR670AA;Selvhjulpen, Barthel indeks 100: 100 point	0		
DR670AB;Lettere afhængig, Barthel indeks 100: 80-99 point	0		
DR670B;Selvhjulpen eller lettere afhængig, Barthel indeks 20: 15-20 point	0		
DR670BA;Selvhjulpen, Barthel indeks 20: 20 point	0		
DR670BB;Lettere afhængig, Barthel indeks 20: 15-19 point	0		
DR671;Moderat afhængig	0		
DR671A;Moderat afhængig, Barthel indeks 100: 50-79 point	0		
DR671B;Moderat afhængig, Barthel indeks 20: 10-14 point	0		
DR672;Væsentlig afhængig	0		
DR672A;Væsentlig afhængig, Barthel indeks 100: 25-49 point	0		
DR672B;Væsentlig afhængig, Barthel indeks 20: 5-9 point	0		
DR673;Fuldstændig afhængig	0		
DR673A;Fuldstændig afhængig, Barthel indeks 100: 0-24 point	0		
DR673B;Fuldstændig afhængig, Barthel indeks 20: 0-4 point	0		
DR679;Generelt nedsat funktionsevne UNS	0		
DR68;Andre generelle symptomer og abnorme fund	0		
DR680;Hypotermi, som ikke skyldes kolde omgivelser	0		
DR681;Uspecifikke symptomer karakteristiske for børn	0		
DR681A;Irritabelt barn	0		
DR681B;Kolikbarn	0		
DR682;Mundtørhed UNS	0		
DR683;Trommestikfingre	0		
DR688;Andet generelt symptom eller abnormt fund	0		
DR688A;Medicinsk uforklaret symptom	0		
DR688A1;Symptomer relateret til dufte og kemiske stoffer	0		
DR688A9;Funktionel lidelse IKA	0		
DR688A9A;Funktionel lidelse, multiorgan	0		

DR688A9B;Funktionel lidelse, enkelt organ	0			
DR688A9B1;Funktionel lidelse, almen/træthed	0			
DR688A9B2;Funktionel lidelse, gastrointestinal	0			
DR688A9B3;Funktionel lidelse, muskuloskeletal	0			
DR688A9B4;Funktionel lidelse, kardiopulmonal	0			
DR688A9B5;Funktionel lidelse, neurologisk	0			
DR688A9B6;Funktionel lidelse, urogenital	0			
DR688A9B9;Anden funktionel lidelse, enkelt organ	0			
DR688A9C;Funktionel lidelse, enkelt symptom	0			
DR69;Ukendte og ikke specificerede årsager til sygdom	0			
DR699;Sygdom uden kendt eller specificeret årsag	0			
DR699A;Sygdom relateret til inhalering af aerosol	0			
DR70;Forhøjet sænkning og abnorm plasmaviskositet	0			
DR700;Forhøjet sænkning	0			
DR701;Abnorm plasmaviskositet	0			
DR71;Abnorme røde blodlegemer	0			
DR719;Erythrocytabnormitet	0			
DR719A;Anisocytose	0			
DR719B;Poikilocytose	0			
DR72;Abnorme hvide blodlegemer IKA	0			
DR728;Andet abnormt fund ved hvide blodlegemer IKA	0			
DR73;Forhøjet blodsukker	0			
DR730;Nedsat glukosetolerance	0			
DR730A;Latent sukkersyge	0			
DR739;Hyperglykæmi UNS	0			
DR74;Abnorme koncentrationer af serumenzymer	0			
DR740;Transaminase- og laktatdehydrogenaseforhøjelse	0			
DR740A;Laktatdehydrogenaseforhøjelse i serum	0			
DR740B;Transaminaseforhøjelse i serum	0			
DR748;Anden abnorm serumenzymkoncentration	0			
DR748A;Abnorm serumamylase	0			
DR748B;Abnorm alkalisk fosfatase-koncentration i serum	0			

DR748D;Abnorm serumlipase	0		
DR748E;Abnorm sur fosfatase-koncentration i serum	0		
DR749;Abnorm koncentration af serumenzym UNS	0		
DR76;Andre abnorme immunologiske fund i serum	0		
DR760;Forhøjet antistoftiter	0		
DR761;Abnorm tuberkulinreaktion	0		
DR762;Falsk positiv reaktion for syfilis	0		
DR768;Andet abnormt immunologisk fund	0		
DR768A;Forhøjede serumimmunglobuliner UNS	0		
DR768D;Forhøjet antifosfolipidantistof-titer	0		
DR769;Abnormt immunologisk fund i serum UNS	0		
DR77;Andre abnorme fund i plasmaproteiner	0		
DR770;Abnormt plasma-albumin	0		
DR770A;Hypoalbuminæmi UNS	0		
DR771;Abnormt plasma-globulin	0		
DR771A;Hyperglobulinæmi UNS	0		
DR772;Abnormt plasma-alfaføtoprotein	0		
DR778;Andet abnormt fund i plasmaproteiner	0		
DR779;Abnorme fund i plasmaproteiner UNS	0		
DR78;Påvisning af lægemidler og andre stoffer, som normalt ikke forekommer i blod	0		
DR780;Alkohol i blodet	0		
DR781;Morfika i blodet	0		
DR782;Kokain i blodet	0		
DR783;Hallucinogener i blodet	0		
DR784;Andre narkotika i blodet	0		
DR785;Psykofarmaka i blodet	0		
DR786;Steroider i blodet	0		
DR787;Øget mængde tungmetal i blodet	0		
DR788;Påvisning af andet fremmed stof i blodet	0		
DR788A;Øget mængde litium i blodet	0		
DR789;Påvisning af ikke specificeret stof, der normalt ikke findes i blodet	0		
DR79;Andre abnorme kemiske fund i blodprøver	0		

DR790;Abnorm mængde mineral i blodet	0			
DR790A;Abnorm mængde zink i blodet	0			
DR790B;Abnorm mængde magnesium i blodet	0			
DR790C;Abnorm mængde jern i blodet	0			
DR790D;Abnorm mængde kobber i blodet	0			
DR798;Anden abnorm blodprøve	0			
DR798B;Abnormt blodgasniveau	0			
DR798C;Hyperkapni	0			
DR798D;Hypokapni	0			
DR798E;Hypoxia	0			
DR799;Abnormt kemisk fund i blodprøve UNS	0			
DR80;Æggehvidestof i urinen	0			
DR809;Proteinuri UNS	0			
DR809A;Bence Jones-proteinuri	0			
DR809B;Albuminuri ved feber	0			
DR809C;Monosymptomatisk proteinuri	0			
DR809D;Albuminuri UNS	0			
DR809E;Forbigående albuminuri	0			
DR81;Sukker i urinen	0			
DR819;Glukosuri	0			
DR82;Andre abnorme fund i urinen	0			
DR820;Kyluri	0			
DR821;Myoglobinuri	0			
DR822;Biliuri	0			
DR823;Hæmoglobinuri	0			
DR823A;Methæmoglobinuri	0			
DR824;Ketonuri	0			
DR825;Øget mængde af lægemiddel eller biologisk substans i urinen	0			
DR825A;Katekolaminuri	0			
DR825B;Lipuri	0			
DR825C;Øget mængde af lægemiddel i urinen UNS	0			
DR825D;Øget mængde steroid i urinen	0			

DR826;Øget mængde af ikke-medikamentelt stof i urinen	0			
DR826A;Øget mængde tungmetal i urinen	0			
DR827;Abnormt fund ved mikrobiologisk undersøgelse af urinen	0			
DR827A;Coliuri	0			
DR827B;Positiv urindyrkning	0			
DR828;Abnorm urincytologi og urinhistologi	0			
DR829;Andet eller ikke specificeret abnormt fund ved urinundersøgelse	0			
DR829A;Cylindre i urinen	0			
DR829B;Melanuri	0			
DR829C;Krystaluri	0			
DR829D;Celler i urinen	0			
DR83;Abnorme fund i cerebrospinalvæske	0			
DR830;Cerebrospinalvæske med abnorm enzymkoncentration	0			
DR831;Cerebrospinalvæske med abnorm hormonkoncentration	0			
DR832;Cerebrospinalvæske med abnormt indhold af lægemiddel eller biologisk substans	0			
DR833;Cerebrospinalvæske med abnormt indhold af ikke-medikamentelt stof	0			
DR834;Cerebrospinalvæske med abnormt immunologisk fund	0			
DR835;Cerebrospinalvæske med abnormt mikrobiologiske fund	0			
DR836;Cerebrospinalvæske med abnorm cytologisk fund	0			
DR838;Cerebrospinalvæske med andet abnormt fund	0			
DR838A;Cerebrospinalvæske med kromosomanomali	0			
DR839;Cerebrospinalvæske med abnormt fund UNS	0			
DR84;Abnorme fund i prøver fra luftveje og thorax	0			
DR840;Prøve fra luftveje eller thorax med abnorm enzymkoncentration	0			
DR841;Prøve fra luftveje eller thorax med abnorm hormonkoncentration	0			
DR842;Prøve fra luftveje eller thorax med abnormt indhold af lægemiddel eller biologisk substans	0			
DR843;Prøve fra luftveje eller thorax med abnormt indhold af ikke-medikamentelt stof	0			
DR844;Prøve fra luftveje eller thorax med abnormt immunologisk fund	0			
DR845;Prøve fra luftveje eller thorax med abnormt mikrobiologisk fund	0			
DR846;Prøve fra luftveje eller thorax med abnormt cytologisk fund	0			
DR847;Prøve fra luftveje eller thorax med abnormt histologisk fund	0			
DR848;Prøve fra luftveje eller thorax med andet abnormt fund	0			

DR849;Prøve fra luftveje eller thorax med abnormt fund UNS	0			
DR85;Abnorme fund i prøver fra fordøjelsesorganer og bughule	0			
DR850;Prøve fra fordøjelsesorgan eller bughulen med abnorm enzymkoncentration	0			
DR851;Prøve fra fordøjelsesorgan eller bughulen med abnorm hormonkoncentration	0			
DR852;Prøve fra fordøjelsesorgan eller bughulen med abnormt indhold af lægemiddel eller biolog	0			
DR853;Prøve fra fordøjelsesorgan eller bughulen med abnormt indhold af ikke-medikamentelt sto	0			
DR854;Prøve fra fordøjelsesorgan eller bughulen med abnormt immunologisk fund	0			
DR855;Prøve fra fordøjelsesorgan eller bughulen med abnormt mikrobiologisk fund	0			
DR856;Prøve fra fordøjelsesorgan eller bughulen med abnormt cytologisk fund	0			
DR857;Prøve fra fordøjelsesorgan eller bughulen med abnormt histologisk fund	0			
DR858;Prøve fra fordøjelsesorgan eller bughulen med andet abnormt fund	0			
DR859;Prøve fra fordøjelsesorgan eller bughulen med abnormt fund UNS	0			
DR86;Abnorme fund i prøver fra mandlige kønsorganer	0			
DR860;Prøve fra mandligt kønsorgan med abnorm enzymkoncentration	0			
DR861;Prøve fra mandligt kønsorgan med abnorm hormonkoncentration	0			
DR862;Prøve fra mandligt kønsorgan med abnormt indhold af lægemiddel eller biologisk substans	0			
DR863;Prøve fra mandligt kønsorgan med abnormt indhold af ikke-medikamentelt stof	0			
DR864;Prøve fra mandligt kønsorgan med abnormt immunologisk fund	0			
DR865;Prøve fra mandligt kønsorgan med abnormt mikrobiologisk fund	0			
DR866;Prøve fra mandligt kønsorgan med abnormt cytologisk fund	0			
DR867;Prøve fra mandligt kønsorgan med abnormt histologisk fund	0			
DR868;Prøve fra mandligt kønsorgan med andet abnormt fund	0			
DR869;Prøve fra mandligt kønsorgan med abnormt fund UNS	0			
DR87;Abnorme fund i prøver fra kvindelige kønsorganer	0			
DR870;Prøve fra kvindeligt kønsorgan med abnorm enzymkoncentration	0			
DR871;Prøve fra kvindeligt kønsorgan med abnormt hormonkoncentration	0			
DR872;Prøve fra kvindeligt kønsorgan med abnormt indhold af lægemiddel eller biologisk substar	0			
DR873;Prøve fra kvindeligt kønsorgan med abnormt indhold af ikke-medikamentelt stof	0			
DR874;Prøve fra kvindeligt kønsorgan med abnormt immunologisk fund	0			
DR875;Prøve fra kvindeligt kønsorgan med abnormt mikrobiologisk fund	0			
DR876;Prøve fra kvindeligt kønsorgan med abnormt cytologisk fund	0			
DR876A;Cervikalsekret med abnormt cytologisk fund	0			

DR877;Prøve fra kvindeligt kønsorgan med abnormt histologisk fund	0			
DR878;Prøve fra kvindeligt kønsorgan med andet abnormt fund	0			
DR879;Prøve fra kvindeligt kønsorgan med abnormt fund UNS	0			
DR89;Abnorme fund i prøver fra andre organer og væv	0			
DR890;Prøve fra andet organ eller væv med abnorm enzymkoncentration	0			
DR891;Prøve fra andet organ eller væv med abnorm hormonkoncentration	0			
DR892;Prøve fra andet organ eller væv med abnormt indhold af lægemiddel eller biologisk substa	0			
DR893;Prøve fra andet organ eller væv med abnormt indhold af ikke medikamentelt stof	0			
DR894;Prøve fra andet organ eller væv med abnormt immunologisk fund	0			
DR895;Prøve fra andet organ eller væv med abnormt mikrobiologisk fund	0			
DR896;Prøve fra andet organ eller væv med abnormt cytologisk fund	0			
DR897;Prøve fra andet organ eller væv med abnormt histologisk fund	0			
DR898;Prøve fra andet organ eller væv med andet abnormt fund	0			
DR899;Prøve fra andet organ eller væv med abnormt fund UNS	0			
DR90;Abnorme fund ved billeddiagnostisk undersøgelse af centralnervesystemet	0			
DR900;Intrakraniel rumopfyldende proces	0			
DR908;Andet abnormt fund ved billeddiagnostisk undersøgelse af centralnervesystemet	0			
DR91;Abnorme fund ved billeddiagnostisk undersøgelse af lungerne	0			
DR919;Abnormt fund ved billeddiagnostisk undersøgelse af lunger	0			
DR92;Abnorme fund ved billeddiagnostisk undersøgelse af mammae	0			
DR929;Abnormt fund ved billeddiagnostisk undersøgelse af mamma	0			
DR93;Abnorme fund ved billeddiagnostisk undersøgelse af anden region	0			
DR930;Abnormt fund ved billeddiagnostisk undersøgelse af kranie IKA	0			
DR931;Abnormt fund ved billeddiagnostisk undersøgelse af hjerte eller koronarkar	0			
DR932;Abnormt fund ved billeddiagnostisk undersøgelse af lever og galdeveje	0			
DR932C;Manglende (kontrast)udskillelse i galdeblæren	0			
DR933;Abnormt fund ved billeddiagnostisk undersøgelse af anden del af fordøjelsesorganerne	0			
DR934;Abnormt fund ved billeddiagnostisk undersøgelse af urinveje	0			
DR935;Abnormt fund ved billeddiagnostisk undersøgelse af anden struktur i abdomen eller retrop	0			
DR936;Abnormt fund ved billeddiagnostisk undersøgelse af ekstremitet	0			
DR937;Abnormt fund ved billeddiagnostisk undersøgelse af anden del af bevægeapparatet	0			
DR938;Abnormt fund ved billeddiagnostisk undersøgelse af anden struktur	0			

Supplement 2 Nielsen et al. Mortality after paediatric emergency calls for patients with and without pre-existing comorbidity (2023)

DR939;Abnormt fund ved billeddiagnostik UNS	0			
DR94;Abnorme fund ved funktionsundersøgelser	0			
DR940;Abnormt fund ved funktionsundersøgelse af centralnervesystemet	0			
DR940A;Elektroencefalografi, abnorm	0			
DR940B;Uden reaktioner og uden sedationsbehov, dvs. mistanke om hjernedød	0			
DR941;Abnormt fund ved funktionsundersøgelse af det perifere nervesystem og sanseorganer	0			
DR942;Abnormt fund ved lungefunktionsundersøgelse	0			
DR943;Abnormt fund ved kardiovaskulær funktionsundersøgelse	0			
DR944;Abnormt fund ved nyrefunktionsundersøgelse	0			
DR945;Abnormt fund ved leverfunktionsundersøgelse	0			
DR946;Abnormt fund ved thyroideafunktionsundersøgelse	0			
DR947;Abnormt fund ved anden endokrin funktionsundersøgelse	0			
DR948;Abnormt fund ved funktionsundersøgelse af andet organ eller organsystem	0			
DR949;Abnorm funktionsundersøgelse UNS	0			
DR97;Indbragt uden livstegn	0			
DR979;Indbragt uden livstegn UNS	0			
DR99;Særlige forhold vedrørende død	0			
DR991;Hjernedød i henhold til sundhedsloven § 176	0			
DR992;Hjertedød i henhold til sundhedsloven § 176	0			
DS00;Overfladisk læsion af hovedet	0			
DS000;Overfladisk læsion af skalpen	0			
DS001;Kontusion af øjenlåg og øjenomgivelser	0			
DS001A;Kontusion af øjenlåg	0			
DS001B;Kontusion af øjenomgivelser	0			
DS002;Anden overfladisk læsion af øjenlåg og øjenomgivelser	0			
DS002A;Overfladisk læsion af øjenlåg UNS	0			
DS002B;Overfladisk læsion af øjenomgivelser UNS	0			
DS003;Overfladisk læsion af næsen	0			
DS003A;Hæmatom i næseskillevæggen	0			
DS004;Overfladisk læsion af øre	0			
DS005;Overfladisk læsion af læbe eller i mundhulen	0			
DS005A;Overfladisk læsion i mundhulen	0			

Supplement 2 Nielsen et al. Mortality after paediatric emergency calls for patients with and without pre-existing comorbidity (2023)

DS005B;Overfladisk læsion af læbe	0			
DS007;Multiple overfladiske læsioner af hovedet	0			
DS008;Anden overfladisk læsion af hovedet	0			
DS009;Overfladisk læsion af hovedet UNS	0			
DS01;Åbent sår på hoved	0			
DS010;Åbent sår på skalpen	0			
DS011;Åbent sår på øjenlåg eller øjenomgivelser med eller uden læsion af tårekanal	0			
DS011A;Åbent sår på øjenlåg	0			
DS011B;Åbent sår i øjenomgivelser	0			
DS011C;Åbent sår på øjenhule	0			
DS012;Åbent sår på næsen	0			
DS013;Åbent sår på øre	0			
DS014;Åbent sår på hagen eller i kæbe-tindingeregion	0			
DS014A;Åbent sår på hagen	0			
DS014B;Åbent sår i kæbe-tindingeregion	0			
DS015;Åbent sår på læbe eller i mundhulen	0			
DS015A;Åbent sår i mundhulen	0			
DS015B;Åbent sår på læbe	0			
DS017;Multiple åbne sår på hovedet	0			
DS018;Åbent sår i anden region på hovedet	0			
DS019;Åbent sår på hovedet UNS	0			
DS02;Kraniebrud og brud af ansigtsknogler	0			
DS020;Fraktur af kraniekalotten	0			
DS020A;Fraktur af pandeben	0			
DS020B;Fraktur af tindingeben	0			
DS021;Fraktur i kraniebasis	0			
DS021A;Fractura fossae cranii anterioris	0			
DS021B;Fractura fossae cranii mediae	0			
DS021C;Fractura fossae cranii posterioris	0			
DS021D;Fraktur i loftet af øjenhule	0			
DS021E;Fractura ossis occipitalis	0			
DS021F;Fractura ossis sphenoidalis	0			

DS021G;Fractura ossis temporalis	0		
DS021GA;Fractura partis tympanicae ossis temporalis	0		
DS021H;Fractura sinus ethmoidalis	0		
DS021I;Fractura sinus frontalis	0		
DS022;Fraktur af næsen	0		
DS022A;Fraktur af næseben	0		
DS022B;Fraktur af næseskillevæggen	0		
DS023;Fraktur i bunden af øjenhule	0		
DS024;Fraktur af kindben eller i overkæbe	0		
DS024A;Fraktur i overkæbe	0		
DS024B;Fraktur af kindben	0		
DS024C;Fractura processus alveolaris maxillae (findes ikke i SKS)	0		
DS025;Fraktur af tand	0		
DS025A;Fractura coronae dentis non complicata	0		
DS025B;Fractura coronae dentis complicata	0		
DS025C;Fractura coronae et radicis dentis non complicata	0		
DS025D;Fractura coronae et radicis dentis complicata	0		
DS025E;Fraktur af tandrod	0		
DS026;Fraktur af underkæbe	0		
DS026A;Fractura colli mandibulae	0		
DS026B;Fractura corporis mandibulae	0		
DS026C;Fractura processus alveolaris mandibulae	0		
DS026D;Fractura processus coronoidei mandibulae	0		
DS026E;Fractura rami mandibulae	0		
DS027;Multiple frakturer i kranie og ansigtsknogler	0		
DS027A;Multiple frakturer i kraniet	0		
DS027B;Multiple frakturer i ansigtsknogler	0		
DS028;Anden fraktur i kranie og ansigtsknogler	0		
DS028A;Fractura orbitae pars lateralis	0		
DS028B;Fractura orbitae pars medialis	0		
DS028C;Fraktur i øjenhule UNS	0		
DS028D;Fractura palati	0		

Supplement 2 Nielsen et al. Mortality after paediatric emergency calls for patients with and without pre-existing comorbidity (2023)

DS028F;Fractura processus alveolaris maxillae	0			
DS029;Kraniebrud eller brud af ansigtsknogle UNS	0			
DS029A;Fraktur i ansigtsknogle UNS	0			
DS03;Ledskred og forvridning af ledbånd på hovedet	0			
DS030;Luksation af kæbeled	0			
DS031;Luksation af brusk i næseskillevæggen	0			
DS032;Luksation af tand	0			
DS033;Luksation af anden eller ikke specificeret del af hoved	0			
DS034;Distorsion af underkæben	0			
DS034A;Distorsion af kæbeled	0			
DS034B;Distorsion af ledbånd i kæbeled	0			
DS035;Forvridning af andet eller ikke specificeret led eller ledbånd på hovedet	0			
DS04;Læsioner af hjernenerve	0			
DS040;Læsion af synsnerve og tractus opticus	0			
DS040A;Laesio traumatica chiasmatis optici	0			
DS040B;Laesio traumatica corticis visualis cerebri	0			
DS040C;Læsion af synsnerve	0			
DS040D;Læsion af tractus opticus	0			
DS041;Læsion af nervus oculomotorius	0			
DS042;Læsion af nervus trochlearis	0			
DS043;Læsion af nervus trigeminus	0			
DS044;Læsion af nervus abducens	0			
DS045;Læsion af nervus facialis	0			
DS046;Læsion af nervus acusticus	0			
DS046A;Nedsat hørelse efter læsion af nervus acusticus	0			
DS047;Læsion af nervus accessorius	0			
DS048;Læsion af anden hjernenerve	0			
DS048A;Læsion af nervus olfactorius	0			
DS048B;Læsion af nervus glossopharyngeus	0			
DS048C;Læsion af nervus vagus	0			
DS048D;Læsion af nervus hypoglossus	0			
DS049;Læsion af hjernenerve UNS	0			

Supplement 2 Nielsen et al. Mortality after paediatric emergency calls for patients with and without pre-existing comorbidity (2023)

DS05;Læsion af øje og øjenhule	0			
DS050;Læsion af konjunktiva eller abrasio på cornea uden fremmedlegeme	0			
DS050A;Abrasio på cornea uden fremmedlegeme	0			
DS050B;Læsion af konjunktiva	0			
DS051;Kontusion af øjeæble eller væv i øjenhule	0			
DS051A;Kontusion af øjeæble	0			
DS051B;Kontusion af væv i øjenhule	0			
DS051C;Traumatisk hyfæma	0			
DS051D;Traumatisk linseluksation	0			
DS051E;Traumatisk irisdefekt	0			
DS051F;Traumatisk kammervinkelskade	0			
DS051G;Traumatisk cyklodialyse	0			
DS051H;Traumatisk nethinderuptur	0			
DS051J;Traumatisk årehinderuptur	0			
DS051K;Traumatisk glaslegemeblødning	0			
DS051L;Partiel traumatisk defekt i cornea	0			
DS051M;Partiel traumatisk defekt i sklera	0			
DS051N;Traumatisk orbitalt kompartmentsyndrom	0			
DS052;Læsion af øjeæble med prolaps eller tab af intraokulært væv	0			
DS053;Læsion af øjeæble uden prolaps eller tab af intraokulært væv	0			
DS053A;Læsion af øjeæble UNS	0			
DS054;Penetrerende sår i øjenhule med eller uden fremmedlegeme	0			
DS055;Penetrerende sår i øjeæble med fremmedlegeme	0			
DS055A;Perforerende sår i øjeæble med fremmedlegeme	0			
DS056;Penetrerende sår i øjeæble uden fremmedlegeme	0			
DS056A;Perforerende sår i øjeæble uden fremmedlegeme	0			
DS057;Traumatisk udrivning af øje	0			
DS058;Anden læsion af øje og øjenhule	0			
DS058A;Traumatisk ruptur af tårekanal	0			
DS058B;Åbent sår på tårekanal	0			
DS059;Læsion af øje eller øjenhule UNS	0			
DS06;Intrakranielle læsioner	0			

Supplement 2 Nielsen et al. Mortality after paediatric emergency calls for patients with and without pre-existing comorbidity (2023)

DS060;Hjernerystelse	0		
DS061;Traumatisk hjerneødem	0		
DS062;Diffus traumatisk hjernelæsion	0		
DS062A;Knusningslæsion af hjernen UNS	0		
DS062B;Hjernekontusion UNS	0		
DS063;Fokal traumatisk hjernelæsion	0		
DS063A;Fokal knusningslæsion i hjernen	0		
DS063B;Fokal hjernekontusion	0		
DS063C;Fokal traumatisk blødning i hjernen	0		
DS064;Traumatisk epidural blødning	0		
DS064A;Traumatisk epiduralt hæmatom	0		
DS065;Traumatisk subdural blødning	0		
DS065A;Traumatisk subduralt hæmatom UNS	0		
DS065B;Akut traumatisk subduralt hæmatom	0		
DS065C;Kronisk traumatisk subduralt hæmatom		1	
DS066;Traumatisk subaraknoidal blødning	0		
DS067;Traumatisk intrakraniel læsion med protraheret coma		1	
DS068;Anden intrakraniel læsion	0		
DS068A;Traumatisk hjernehæmatom	0		
DS068B;Traumatisk blødning i lillehjernen	0		
DS068C;Traumatisk blødning i hjernen UNS	0		
DS068D;Traumatisk intrakraniel blødning UNS	0		
DS068E;Traumatisk blødning i tentorium cerebelli	0		
DS068F;Traumatisk intrakraniel karlæsion	0		
DS069;Intrakraniel læsion UNS	0		
DS07;Knusningslæsion på hovedet	0		
DS070;Knusningslæsion i ansigtet	0		
DS071;Knusningslæsion på kraniet	0		
DS078;Knusningslæsion på anden del af hovedet	0		
DS079;Knusningslæsion på hovedet UNS	0		
DS08;Traumatisk amputation af dele af hovedet	0		
DS080;Traumatisk amputation af skalpen	0		

DS080A;Traumatisk amputation af hovedhår	0		
DS081;Traumatisk amputation af øre	0		
DS088;Traumatisk amputation af anden del af hovedet	0		
DS088A;Traumatisk amputation af næsen	0		
DS089;Traumatisk amputation af del af hovedet UNS	0		
DS09;Anden og ikke specificeret læsion i hovedet	0		
DS090;Læsion af kar i hovedet IKA	0		
DS091;Læsion af muskel eller sene på hovedet	0		
DS091A;Læsion af muskel på hovedet	0		
DS091B;Læsion af sene på hovedet	0		
DS092;Traumatisk ruptur af trommehinde	0		
DS097;Multiple læsioner i hovedet	0		
DS098;Anden læsion i hoved	0		
DS098A;Traumatisk blødning i trommehule	0		
DS098B;Tandskade UNS	0		
DS099;Læsion i hovedet UNS	0		
DS10;Overfladisk læsion på halsen	0		
DS100;Kontusion på halsens forreste del	0		
DS100A;Kontusion af larynx	0		
DS100B;Kontusion af øsofagus på halsen	0		
DS100C;Kontusion af trakea	0		
DS101;Anden eller ikke specificeret overfladisk læsion på halsen	0		
DS101A;Overfladisk læsion på larynx UNS	0		
DS101B;Overfladisk læsion på øsofagus i halsregionen UNS	0		
DS101C;Overfladisk læsion i svælget UNS	0		
DS101D;Overfladisk læsion på luftrøret UNS	0		
DS107;Multiple overfladiske læsioner på halsen	0		
DS108;Overfladisk læsion på anden del af halsen	0		
DS109;Overfladisk læsion på halsen UNS	0		
DS11;Åbent sår på halsen	0		
DS110;Åbent sår, der involverer larynx eller trakea	0		
DS110A;Åbent sår, der involverer larynx	0		

DS110B;Åbent sår, der involverer trakea på halsen	0		
DS110C;Åbent sår på trakea UNS	0		
DS111;Åbent sår, der involverer skjoldbruskkirtlen	0		
DS112;Åbent sår, der involverer pharynx eller øsofagus i halsregionen	0		
DS112A;Åbent sår, der involverer øsofagus i halsregionen	0		
DS112B;Åbent sår i svælget	0		
DS117;Multiple åbne sår på halsen	0		
DS118;Åbent sår på anden del af halsen	0		
DS119;Åbent sår på halsen UNS	0		
DS12;Fraktur i halsregionen	0		
DS120;Fraktur af 1. halshvirvel	0		
DS121;Fraktur af 2. halshvirvel	0		
DS121C;Fractura dens axis	0		
DS122;Fraktur af anden del af cervikale rygsøjle	0		
DS122A;Fraktur af 3. halshvirvel	0		
DS122B;Fraktur af 4. halshvirvel	0		
DS122C;Fraktur af 5. halshvirvel	0		
DS122D;Fraktur af 6. halshvirvel	0		
DS122E;Fraktur af 7. halshvirvel	0		
DS127;Multiple frakturer i cervikale rygsøjle	0		
DS128;Fraktur af anden struktur i halsen	0		
DS128A;Fraktur af cartilago thyroidea	0		
DS128B;Fraktur af larynx	0		
DS128C;Fraktur af tungebenet	0		
DS128D;Fraktur af luftrøret	0		
DS129;Fraktur i halsregionen UNS	0		
DS13;Luksation og distorsion af led og ligamenter på halsen	0		
DS130;Traumatisk cervikal diskusruptur	0		
DS131;Luksation af halshvirvel	0		
DS131A;Luxatio articuli columnae cervicalis	0		
DS131B;Luxatio atlanto-occipitalis	0		
DS132;Luksation af andet eller ikke specificeret led i halsen	0		

DS132A;Luksation af led i halsen UNS	0		
DS132B;Luxatio articuli crico-arytaenoidei	0		
DS133;Multiple luksationer af led i halsen	0		
DS134;Distorsion i cervikale rygsøjle	0		
DS134A;Distorsio ligamenti longitudinalis anterioris cervicalis	0		
DS134B;Traumatisk torticollis	0		
DS134C;Whiplash-syndrom	0		
DS134D;Distorsio articuli columnae vertebralis cervicalis	0		
DS134E;Distorsio atlanto-axialis	0		
DS134F;Distorsio atlanto-occipitalis	0		
DS134G;Cervikalt facetsyndrom	0		
DS135;Distorsion i strubehovedet	0		
DS136;Luksation eller distorsion af anden eller ikke specificeret del af halsen	0		
DS14;Læsion af nerver og rygmarv i halsregionen	0		
DS140;Concussio et oedema traumaticum medullae spinalis cervicalis	0		
DS140A;Concussio medullae spinalis cervicalis	0		
DS140B;Oedema traumaticum medullae spinalis cervicalis	0		
DS141;Anden eller ikke specificeret læsion af den cervikale rygmarv	0		
DS141A;Commotio medullae spinalis cervicalis	0		
DS141B;Knusningslæsion af den cervikale rygmarv	0		
DS141C;Epiduralt traumatisk hæmatom i den cervikale rygmarv	0		
DS141D;Intramedullært traumatisk hæmatom i den cervikale rygmarv	0		
DS141E;Traumatisk hæmatomyeli i den cervikale rygmarv	0		
DS142;Læsion af spinal nerverod i halsen	0		
DS143;Læsion af plexus brachialis	0		
DS144;Læsion af perifer nerve i halsen	0		
DS145;Læsion af autonom nerve i halsen	0		
DS145A;Traumatisk Horner-syndrom	0		
DS146;Anden eller ikke specificeret nervelæsion i halsen	0		
DS15;Læsion af blodkar i halsregionen	0		
DS150;Læsion af arteria carotis	0		
DS150A;Læsion af arteria carotis communis	0		

DS150B;Læsion af arteria carotis externa	0		
DS150C;Læsion af arteria carotis interna	0		
DS151;Læsion af arteria vertebralis	0		
DS152;Læsion af vena jugularis externa	0		
DS153;Læsion af vena jugularis interna	0		
DS157;Multiple karlæsioner i halsen	0		
DS158;Læsion af andet blodkar i halsen	0		
DS159;Læsion af blodkar i halsen UNS	0		
DS16;Læsion af muskler og sener i halsregionen	0		
DS169;Læsion af muskel eller sene på halsen	0		
DS169A;Læsion af muskel i halsen	0		
DS169B;Læsion af sene i halsen	0		
DS17;Knusningslæsion på halsen	0		
DS170;Knusningslæsion af larynx eller trakea	0		
DS170A;Knusningslæsion af larynx	0		
DS170B;Knusningslæsion af trakea	0		
DS178;Knusningslæsion af anden struktur i halsen	0		
DS179;Knusningslæsion på halsen UNS	0		
DS18;Traumatisk amputation af hovedet			2
DS189;Dekapitering			2
DS19;Andre og ikke specificerede læsioner på halsen	0		
DS197;Multiple læsioner i halsregionen	0		
DS198;Anden læsion på halsen	0		
DS199;Læsion på halsen UNS	0		
DS20;Overfladisk læsion af thorax	0		
DS200;Kontusion af mamma	0		
DS201;Anden eller ikke specificeret overfladisk læsion af mamma	0		
DS202;Kontusion af thorax	0		
DS203;Anden overfladisk læsion af forsiden af thorax	0		
DS204;Anden overfladisk læsion af bagsiden af thorax	0		
DS207;Multiple overfladiske thoraxlæsioner	0		
DS208;Overfladisk læsion af anden eller ikke specificeret del af thorax	0		

Supplement 2 Nielsen et al. Mortality after paediatric emergency calls for patients with and without pre-existing comorbidity (2023)

DS21;Åbent sår på thorax	0			
DS210;Åbent sår på mamma	0			
DS211;Åbent sår på forsiden af thorax	0			
DS212;Åbent sår på bagsiden af thorax	0			
DS217;Multiple åbne sår på thorax	0			
DS218;Åbent sår på anden del af thorax	0			
DS219;Åbent sår på thorax UNS	0			
DS22;Fraktur af ribben, brystbenet og torakale rygsøjle	0			
DS220;Fraktur af torakale rygsøjle	0			
DS220A;Fractura columnae vertebralis I thoracalis	0			
DS220B;Fractura columnae vertebralis II thoracalis	0			
DS220C;Fractura columnae vertebralis III thoracalis	0			
DS220D;Fractura columnae vertebralis IV thoracalis	0			
DS220E;Fractura columnae vertebralis V thoracalis	0			
DS220F;Fractura columnae vertebralis VI thoracalis	0			
DS220G;Fractura columnae vertebralis VII thoracalis	0			
DS220H;Fractura columnae vertebralis VIII thoracalis	0			
DS220I;Fractura columnae vertebralis IX thoracalis	0			
DS220J;Fractura columnae vertebralis X thoracalis	0			
DS220K;Fractura columnae vertebralis XI thoracalis	0			
DS220L;Fractura columnae vertebralis XII thoracalis	0			
DS221;Multiple frakturer i torakale rygsøjle	0			
DS222;Fraktur af brystbenet	0			
DS223;Fraktur af ribben	0			
DS224;Multiple ribbensfrakturer	0			
DS225;Ustabil thoraxvæg ved multiple ribbensfrakturer	0			
DS228;Fraktur af anden del af thoraxskelettet	0			
DS229;Fraktur af thoraxskelettet UNS	0			
DS23;Luksation og distorsion af led og ligamenter i thoraxskelettet	0			
DS230;Traumatisk torakal diskusruptur	0			
DS231;Luksation af brysthvirvel	0			
DS232;Luksation i anden eller ikke specificeret del af thorax	0			

DS232A;Luksation af led i torakale rygsøjle UNS	0			
DS233;Distorsion i torakale rygsøjle	0			
DS233A;Distorsion af led i torakale rygsøjle	0			
DS233B;Torakalt facetsyndrom	0			
DS234;Distorsion af ribben eller brystbenet	0			
DS234A;Distorsion af ribben	0			
DS234B;Distorsion af brystbenet	0			
DS235;Distorsion i anden eller ikke specificeret del af thorax	0			
DS235A;Distorsion i thorax UNS	0			
DS24;Læsion af nerver og rygmarv i brystregionen	0			
DS240;Concussio et oedema traumaticum medullae spinalis thoracalis	0			
DS240A;Concussio medullae spinalis thoracalis	0			
DS240B;Oedema traumaticum medullae spinalis thoracalis	0			
DS241;Anden eller ikke specificeret læsion af den torakale rygmarv	0			
DS241A;Commotio medullae spinalis thoracalis	0			
DS241B;Knusningslæsion af den torakale rygmarv	0			
DS241C;Kontusion af den torakale rygmarv	0			
DS241D;Epiduralt traumatisk hæmatom i den torakale rygmarv	0			
DS241E;Intramedullært traumatisk hæmatom i den torakale rygmarv	0			
DS241F;Traumatisk hæmatomyeli i den torakale rygmarv	0			
DS242;Læsion af torakal spinal nerverod	0			
DS243;Læsion af perifer nerve i thorax	0			
DS244;Læsion af autonom nerve i thorax	0			
DS245;Læsion af anden nerve i thorax	0			
DS246;Læsion af nerve i thorax UNS	0			
DS25;Læsion af blodkar i thorax	0			
DS250;Læsion af aorta thoracalis	0			
DS250B;Læsion af aorta UNS	0			
DS251;Læsion af truncus brachiocephalicus eller arteria subclavia	0			
DS252;Læsion af vena cava superior	0			
DS252A;Læsion af vena cava UNS	0			
DS253;Læsion af vena brachiocephalica eller vena subclavia	0			

DS254;Læsion af blodkar i lunge	0			
DS255;Læsion af interkostale blodkar	0			
DS257;Multiple karlæsioner i thorax	0			
DS258;Læsion af andet blodkar i thorax	0			
DS259;Læsion af blodkar i thorax UNS	0			
DS259A;Traumatisk hæmatom i mediastinum	0			
DS26;Læsion af hjertet	0			
DS260;Læsion af hjertet med hæmoperikardium	0			
DS268;Anden læsion af hjertet	0			
DS268A;Læsion af hjertet uden hæmoperikardium	0			
DS269;Læsion af hjertet UNS	0			
DS27;Læsion af andre og ikke specificerede organer i thorax	0			
DS270;Traumatisk pneumothorax	0			
DS271;Traumatisk hæmothorax	0			
DS272;Traumatisk pneumohæmothorax	0			
DS273;Anden lungelæsion	0			
DS274;Bronkielæsion	0			
DS275;Trakealæsion i thorax	0			
DS276;Pleuralæsion	0			
DS277;Multiple læsioner af intratorakale organer	0			
DS278;Anden læsion af organ i thorax	0			
DS278A;Læsion af diafragma	0			
DS278B;Traumatisk læsion af ductus thoracicus	0			
DS278C;Læsion af torakale øsofagus	0			
DS278D;Læsion af thymus	0			
DS278E;Traumatisk ruptur af diafragma	0			
DS279;Læsion af organ i thorax UNS	0			
DS28;Knusningslæsion af thorax og traumatisk amputation af dele af thorax	0			
DS280;Knusningslæsion af thorax	0			
DS281;Traumatisk amputation af dele af thorax	0			
DS29;Andre og ikke specificerede læsioner af thorax	0			
DS290;Læsion af muskel eller sene i thorax	0			

Supplement 2 Nielsen et al. Mortality after paediatric emergency calls for patients with and without pre-existing comorbidity (2023)

DS290A;Læsion af muskel i thorax	0		
DS290B;Læsion af sene i thorax	0		
DS297;Multiple læsioner i thorax IKA	0		
DS298;Anden læsion af thorax	0		
DS299;Læsion af thorax UNS	0		
DS30;Overfladisk læsion af abdomen, lænden og bækkenet	0		
DS300;Kontusion af lænden eller bækkenet	0		
DS300A;Kontusion af sædet	0		
DS300B;Kontusion af lænden	0		
DS300C;Kontusion af bækkenet	0		
DS301;Kontusion af bugvæggen	0		
DS302;Kontusion af ydre kønsorganer	0		
DS302A;Kontusion af store kønslæber	0		
DS302B;Kontusion af penis	0		
DS302C;Kontusion af perineum	0		
DS302D;Kontusion af scrotum	0		
DS302E;Kontusion af testikel	0		
DS302F;Kontusion af vagina	0		
DS302G;Kontusion af ydre kvindelige kønsorganer	0		
DS302P;Fraktur af penis	0		
DS307;Multiple overfladiske læsioner af abdomen, lænden og bækkenet	0		
DS308;Anden overfladisk læsion af abdomen, lænden og bækkenet	0		
DS309;Overfladisk læsion af abdomen, lænden og bækkenet UNS	0		
DS31;Åbent sår på abdomen, lænden og bækkenet	0		
DS310;Åbent sår på lænden eller bækkenet	0		
DS310A;Åbent sår på sædet	0		
DS310B;Åbent sår på lænden	0		
DS310C;Åbent sår på bækkenet	0		
DS311;Åbent sår på bugvæggen	0		
DS312;Åbent sår på penis	0		
DS313;Åbent sår på scrotum eller testikel	0		
DS313A;Åbent sår på scrotum	0		

DS313B;Åbent sår på testikel	0			
DS314;Åbent sår i vagina eller på ydre kvindelige kønsorganer	0			
DS314A;Åbent sår i vagina	0			
DS314B;Åbent sår på ydre kvindelige kønsorganer	0			
DS315;Åbent sår på anden eller ikke specificeret del af ydre kønsorganer	0			
DS317;Multiple åbne sår på abdomen, lænden og bækkenet	0			
DS317A;Multiple åbne sår på abdomen	0			
DS317B;Multiple åbne sår på lænden	0			
DS317C;Multiple åbne sår på bækkenet	0			
DS318;Åbent sår på anden eller ikke specificeret del af abdomen, lænden eller bækkenet	0			
DS318A;Åbent sår på abdomen UNS	0			
DS318B;Åbent sår på lænd UNS	0			
DS318C;Åbent sår på bækkenregion UNS	0			
DS32;Fraktur af lumbale rygsøjle og bækkenet	0			
DS320;Fraktur af lumbale rygsøjle	0			
DS320A;Fraktur af 1. lændehvirvel	0			
DS320B;Fraktur af 2. lændehvirvel	0			
DS320C;Fraktur af 3. lændehvirvel	0			
DS320D;Fraktur af 4. lændehvirvel	0			
DS320E;Fraktur af 5. lændehvirvel	0			
DS321;Fraktur af os sacrum	0			
DS322;Fraktur af halebenet	0			
DS323;Fraktur af os ilium	0			
DS324;Fraktur af acetabulum	0			
DS325;Fraktur af os pubis	0			
DS327;Multiple frakturer i lumbale rygsøjle og bækkenet	0			
DS327A;Multiple frakturer i lumbale rygsøjle	0			
DS327B;Multiple frakturer i bækkenet	0			
DS328;Fraktur af anden eller ikke specificeret del af lumbale rygsøjle eller bækkenet	0			
DS328A;Fraktur af lumbale rygsøjle UNS	0			
DS328B;Fraktur af os ischii	0			
DS328C;Bækkenfraktur UNS	0			

Supplement 2 Nielsen et al. Mortality after paediatric emergency calls for patients with and without pre-existing comorbidity (2023)

DS33;Luksation og distorsion af led og ligamenter i lumbale rygsøjle og bækkenet	0			
DS330;Traumatisk diskusruptur i lænden	0			
DS331;Luksation af lændehvirvel	0			
DS331A;Luxatio articuli columnae lumbalis	0			
DS332;Luksation af sakroiliakaled eller sakrococcygealled	0			
DS332A;Luksation af sakrococcygealled	0			
DS332B;Luksation af sakroiliakaled	0			
DS333;Luksation i anden eller ikke specificeret del af lumbale rygsøjle eller bækkenet	0			
DS333A;Luksation i lumbale rygsøjle UNS	0			
DS333B;Luksation i bækkenet UNS	0			
DS334;Traumatisk ruptur af symfysen	0			
DS335;Distorsion i lumbale rygsøjle	0			
DS335A;Lumbalt facetsyndrom	0			
DS336;Distorsion af sakroiliakaled	0			
DS336A;Sakroiliakasyndrom	0			
DS337;Distorsion i anden eller ikke specificeret del af lumbale rygsøjle eller bækkenet	0			
DS337A;Distorsion af bækkenled UNS	0			
DS337B;Distorsion af symfysen	0			
DS34;Læsion af nerver og rygmarv i lænde- og bækkenregionen	0			
DS340;Concussio et oedema medullae spinalis lumbalis	0			
DS340A;Concussio medullae spinalis lumbalis	0			
DS340B;Oedema medullae spinalis lumbalis	0			
DS341;Anden eller ikke specificeret læsion af den lumbale rygmarv	0			
DS341A;Commotio medullae spinalis lumbalis	0			
DS341B;Knusningslæsion af den lumbale rygmarv	0			
DS341C;Kontusion af den lumbale rygmarv	0			
DS341D;Epiduralt traumatisk hæmatom i den lumbale rygmarv	0			
DS341E;Intramedullært traumatisk hæmatom i den lumbale rygmarv	0			
DS341F;Traumatisk hæmatomyeli i den lumbale rygmarv	0			
DS342;Læsion af lumbal eller sakral spinal nerverod	0			
DS342A;Læsion af lumbal spinal nerverod	0			
DS342B;Læsion af sakral spinal nerverod	0			

DS343;Læsion af cauda equina	0			
DS344;Læsion af plexus lumbosacralis	0			
DS345;Læsion af lumbale eller sakrale sympatiske nerver	0			
DS346;Læsion af perifer nerve i abdomen, lænden eller bækkenet	0			
DS348;Læsion af anden eller ikke specificeret nerve i abdomen, lænden eller bækkenet	0			
DS35;Læsion af blodkar i abdomen, lænden og bækkenet	0			
DS350;Læsion af aorta i abdomen	0			
DS351;Læsion af vena cava inferior	0			
DS351A;Læsion af vena hepatica	0			
DS352;Læsion af truncus coeliacus eller arteria mesenterica	0			
DS353;Læsion af vena porta eller vena mesenterica	0			
DS354;Læsion af nyrekar	0			
DS355;Læsion af blodkar i bækkenet	0			
DS357;Multiple læsioner af blodkar i abdomen, lænden eller bækkenet	0			
DS358;Læsion af andet blodkar i abdomen, lænden eller bækkenet	0			
DS359;Læsion af blodkar i abdomen, lænden eller bækkenet UNS	0			
DS36;Læsion af organer i bughulen	0			
DS360;Læsion af milten	0			
DS361;Læsion af leveren eller galdeblæren	0			
DS361A;Traumatisk subkapsulært leverhæmatom	0			
DS361B;Læsion af galdeblæren	0			
DS361C;Læsion af galdegang	0			
DS361D;Læsion af leveren	0			
DS362;Læsion af pancreas	0			
DS363;Læsion af mavesækken	0			
DS364;Læsion af tyndtarmen	0			
DS364A;Læsion af tolvfingertarmen	0			
DS365;Læsion af tyktarmen	0			
DS365A;Læsion af appendix	0			
DS366;Læsion af rektum	0			
DS367;Multiple læsioner af intraabdominale organer	0			
DS368;Læsion af andet intraabdominalt organ	0			

DS368A;Traumatisk hæmatom i mesenteriet	0			
DS368B;Traumatisk retroperitonalt hæmatom	0			
DS368C;Traumatisk hæmoperitoneum	0			
DS368D;Traumatisk retroperitonal blødning	0			
DS368E;Læsion af mave-tarm-kanalen UNS	0			
DS368F;Læsion af mesenteriet	0			
DS368G;Læsion af omentet	0			
DS368H;Læsion af peritoneum	0			
DS369;Læsion af intraabdominalt organ UNS	0			
DS37;Læsion af bækkenorganer	0			
DS370;Læsion af nyre	0			
DS371;Læsion af urinleder	0			
DS372;Læsion af urinblæren	0			
DS373;Læsion af urinrøret	0			
DS374;Læsion af ovarie	0			
DS375;Læsion af æggeleder	0			
DS376;Læsion af livmoderen	0			
DS377;Multiple læsioner af bækkenorganer	0			
DS378;Læsion af andet bækkenorgan	0			
DS378A;Traumatisk hæmatom i sædstreng	0			
DS378B;Læsion af binyre	0			
DS378C;Læsion af prostata	0			
DS378D;Læsion af ductus deferens	0			
DS378E;Læsion af vesicula seminalis	0			
DS379;Læsion af bækkenorgan UNS	0			
DS38;Knusningslæsion og traumatisk amputation af dele af abdomen, lænden og bækkenet	0			
DS380;Knusningslæsion af ydre kønsorgan	0			
DS381;Knusningslæsion af anden del af abdomen, lænden eller bækkenet	0			
DS381A;Knusningslæsion i abdomen UNS	0			
DS381B;Knusningslæsion i lænden UNS	0			
DS381C;Knusningslæsion i bækkenet UNS	0			
DS382;Traumatisk amputation af ydre kønsorgan	0			

DS382A;Traumatisk amputation af stor kønslæbe	0			
DS382B;Traumatisk amputation af lille kønslæbe	0			
DS382C;Traumatisk amputation af penis	0			
DS382D;Traumatisk amputation af testikel	0			
DS382E;Traumatisk amputation af ydre kvindelige kønsorganer	0			
DS383;Traumatisk amputation af anden del af abdomen, lænden eller bækkenet	0			
DS383A;Partiel traumatisk amputation af abdomen UNS	0			
DS383B;Partiel traumatisk amputation af lænden UNS	0			
DS383C;Partiel traumatisk amputation af bækkenet UNS	0			
DS39;Andre og ikke specificerede læsioner af abdomen, lænden og bækkenet	0			
DS390;Læsion af muskel eller sene i abdomen, lænden eller bækkenet	0			
DS390A;Læsion af muskel i abdomen	0			
DS390B;Læsion af muskel i lænden	0			
DS390C;Læsion af muskel i bækkenet	0			
DS390D;Læsion af sene i abdomen	0			
DS390E;Læsion af sene i lænden	0			
DS390F;Læsion af sene i bækkenet	0			
DS396;Læsion af organer både i abdomen og i bækkenet	0			
DS397;Multiple læsioner i abdomen, lænden eller bækkenet	0			
DS397A;Multiple læsioner i abdomen	0			
DS397B;Multiple læsioner i lænden	0			
DS397C;Multiple læsioner i bækkenet	0			
DS398;Anden læsion af abdomen, lænden eller bækkenet	0			
DS399;Læsion af abdomen, lænden eller bækkenet UNS	0			
DS40;Overfladisk læsion af skulder og overarm	0			
DS400;Kontusion af skulder eller overarm	0			
DS400A;Kontusion af overarm	0			
DS400B;Kontusion af skulder	0			
DS407;Multiple overfladiske læsioner af skulder og overarm	0			
DS407A;Multiple overfladiske læsioner af overarm	0			
DS407B;Multiple overfladiske læsioner af skulder	0			
DS408;Anden overfladisk læsion af skulder eller overarm	0			

DS409;Overfladisk læsion af skulder eller overarm UNS	0		
DS41;Åbent sår på skulder og overarm	0		
DS410;Åbent sår på skulder	0		
DS411;Åbent sår på overarm	0		
DS417;Multiple åbne sår på skulder eller overarm	0		
DS417A;Multiple åbne sår på overarm	0		
DS417B;Multiple åbne sår på skulder	0		
DS418;Åbent sår på anden eller ikke specificeret del af skulder eller overarm	0		
DS42;Fraktur af skulder og overarm	0		
DS420;Fraktur af clavicula	0		
DS420A;Fraktur af clavicula, medialt	0		
DS420B;Fraktur af clavicula, midtskaft	0		
DS420C;Fraktur af clavicula, lateral	0		
DS421;Fraktur af skulderblad	0		
DS422;Fraktur af proksimale del af overarmsknogle	0		
DS422A;Fraktur af ledhoved på overarmsknogle	0		
DS422B;Fraktur af collum chirurgicum på overarmsknogle	0		
DS422C;Fraktur af tuberculum majus på overarmsknogle	0		
DS423;Fraktur af skaftet på overarmsknogle	0		
DS423A;Fraktur på overarmsknogle UNS	0		
DS424;Fraktur af nederste del af overarmsknogle	0		
DS424A;Fraktur af kondyl på overarmsknogle	0		
DS424B;Fraktur af epikondyl på overarmsknogle	0		
DS424C;Suprakondylær fraktur på overarmsknogle	0		
DS427;Multiple frakturer af clavicula, skulderblad eller overarmsknogle	0		
DS427A;Multiple frakturer af clavicula	0		
DS427B;Multiple frakturer af overarmsknogle	0		
DS427C;Multiple frakturer af skulderblad	0		
DS428;Fraktur af anden del af skulder eller overarm	0		
DS429;Fraktur i skulder og overarm UNS	0		
DS43;Luksation og distorsion af led og ligamenter i skulderregion	0		
DS430;Luksation i skulderled	0		

DS431;Luksation i akromioclaviculærled	0			
DS432;Luksation i sternoclaviculærled	0			
DS433;Luksation af anden eller ikke specificeret del af skulderregion	0			
DS434;Distorsion af skulderled	0			
DS434A;Distorsion af ligamentum coracohumerale	0			
DS435;Distorsion af akromioclaviculærled	0			
DS435A;Distorsion af ligamentum acromioclaviculare	0			
DS436;Distorsion af sternoclaviculærled	0			
DS437;Distorsion af anden eller ikke specificeret del af skulderregion	0			
DS44;Læsion af nerver i skulderregion og overarm	0			
DS440;Læsion af nervus ulnaris på overarm	0			
DS441;Læsion af nervus medianus på overarm	0			
DS442;Læsion af nervus radialis på overarm	0			
DS443;Læsion af nervus axillaris	0			
DS444;Læsion af nervus musculocutaneus	0			
DS445;Læsion af sensorisk nerve i skulderregion eller overarm	0			
DS447;Mulitiple nervelæsioner i skulderregion eller overarm	0			
DS448;Læsion af anden nerve i skulderregion eller overarm	0			
DS449;Læsion af nerve i skulderregion eller overarm UNS	0			
DS45;Læsion af blodkar i skulderregion og overarm	0			
DS450;Læsion af arteria axillaris	0			
DS451;Læsion af arteria brachialis	0			
DS452;Læsion af vena axillaris eller vena brachialis	0			
DS453;Læsion af overfladisk vene i skulderregion eller overarm	0			
DS457;Multiple karlæsioner i skulderregion eller overarm	0			
DS458;Læsion af andet blodkar i skulderregion eller overarm	0			
DS459;Læsion af blodkar i skulderregion eller overarm UNS	0			
DS46;Læsion af muskler og sener i skulder og overarm	0			
DS460;Læsion af rotatormanchet	0			
DS460A;Læsion af muskel i rotatormanchet	0			
DS460A1;Subskapularis muskellæsion	0			
DS460A2;Supraspinatus muskellæsion	0			

Supplement 2 Nielsen et al. Mortality after paediatric emergency calls for patients with and without pre-existing comorbidity (2023)

DS460A3;Infraspinatus muskellæsion	0			
DS460B;Læsion af sene i rotatormanchet	0			
DS460B1;Subskapularis senelæsion	0			
DS460B2;Supraspinatus senelæsion	0			
DS460B3;Infraspinatus senelæsion	0			
DS461;Læsion af muskel og sene af caput longum, musculus biceps brachii	0			
DS461A;Muskellæsion af caput longum, musculus biceps brachii	0			
DS461B;Senelæsion af caput longum, musculus biceps brachii	0			
DS461C;Læsion af SLAP (superior labrum anterior posterior)	0			
DS461D;Læsion af caput breve, musculus biceps brachii	0			
DS462;Anden muskel- eller senelæsion i musculus biceps brachii	0			
DS462A;Muskellæsion i musculus biceps brachii UNS	0			
DS462B;Senelæsion i musculus biceps brachii UNS	0			
DS462C;Anden senelæsion af musculus biceps brachii (distal læsion)	0			
DS463;Læsion af muskel og sene i musculus triceps brachii	0			
DS463A;Muskellæsion i musculus triceps brachii	0			
DS463B;Senelæsion i musculus triceps brachii	0			
DS467;Multiple læsioner af muskler eller sener i skulder eller overarm	0			
DS467A;Multiple muskellæsioner i overarm	0			
DS467B;Multiple muskellæsioner i skulderregion	0			
DS467B1;Multiple muskellæsioner af rotatormanchet	0			
DS467C;Multiple senelæsioner i overarm	0			
DS467D;Multiple senelæsioner i skulderregion	0			
DS467D1;Multiple senelæsioner i rotatormanchet	0			
DS468;Læsion af anden muskel eller sene i skulderregion eller overarm	0			
DS469;Læsion af muskel eller sene i skulderregion eller overarm UNS	0			
DS47;Knusningslæsion af skulderregion og overarm	0			
DS479;Knusningslæsion af skulderregion eller overarm	0			
DS479A;Knusningslæsion af overarm	0			
DS479B;Knusningslæsion af skulderregion	0			
DS48;Traumatisk amputation af skulder og overarm	0			
DS480;Traumatisk amputation i skulderled	0			

DS481;Traumatisk amputation på overarm	0		
DS489;Traumatisk amputation af skulder eller overarm UNS	0		
DS49;Andre og ikke specificerede læsioner af skulderregion og overarm	0		
DS497;Multiple læsioner af skulderregion eller overarm	0		
DS498;Anden læsion af skulderregion eller overarm	0		
DS499;Læsion af skulderregion eller overarm UNS	0		
DS50;Overfladisk læsion af albueregion og underarm	0		
DS500;Kontusion af albue	0		
DS501;Kontusion af anden eller ikke specificeret del af underarm	0		
DS507;Multiple overfladiske læsioner af albueregion eller underarm	0		
DS508;Anden overfladisk læsion af albueregion eller underarm	0		
DS509;Overfladisk læsion af albueregion eller underarm UNS	0		
DS51;Åbent sår på albueregion og underarm	0		
DS510;Åbent sår på albue	0		
DS517;Multiple åbne sår på albueregion eller underarm	0		
DS518;Åbent sår på anden del af albueregion eller underarm	0		
DS519;Åbent sår på albueregion eller underarm UNS	0		
DS52;Fraktur af albue og underarm	0		
DS520;Fraktur af proksimale ende af ulna	0		
DS520A;Fraktur af albue UNS	0		
DS520B;Fraktur af olecranon	0		
DS520C;Fraktur af processus coronoideus ulnae	0		
DS520D;Monteggia-fraktur	0		
DS521;Fraktur af proksimale ende af radius	0		
DS521A;Fraktur af caput radii	0		
DS521B;Fraktur af collum radii	0		
DS522;Fraktur af corpus ulnae	0		
DS523;Fraktur af corpus radii	0		
DS524;Fraktur af både corpus ulnae og corpus radii	0		
DS525;Fraktur af distale ende af radius	0		
DS525A;Bartons fraktur	0		
DS525B;Colles' fraktur	0		

DS525C;Smiths fraktur	0			
DS526;Fraktur af distale ende af både ulna og radius	0			
DS527;Multiple frakturer af underarmknogle	0			
DS528;Fraktur af anden del af underarm	0			
DS528A;Fraktur af caput ulnae	0			
DS528B;Fraktur af processus styloideus ulnae	0			
DS528C;Fraktur af distale ende af ulna	0			
DS529;Fraktur i underarm UNS	0			
DS53;Luksation og distorsion af led og ligamenter i albueregion	0			
DS530;Luksation af caput radii	0			
DS531;Luksation i albueled UNS	0			
DS531A;Luksation i humero-ulnarled	0			
DS532;Traumatisk ruptur af radialt kollateralligament	0			
DS533;Traumatisk ruptur af ulnart kollateralligament	0			
DS534;Distorsion af albueled	0			
DS54;Læsion af nerver i albueregion og underarm	0			
DS540;Læsion af nervus ulnaris i albueregion eller underarm	0			
DS540A;Læsion af nervus ulnaris UNS	0			
DS541;Læsion af nervus medianus i albueregion eller underarm	0			
DS541A;Læsion af nervus medianus UNS	0			
DS542;Læsion af nervus radialis i albueregion eller underarm	0			
DS542A;Læsion af nervus radialis UNS	0			
DS543;Læsion af kutan sensorisk hudnerve på underarm	0			
DS547;Multiple nervelæsioner i albueregion eller underarm	0			
DS548;Læsion af anden nerve i albueregion eller underarm	0			
DS549;Læsion af nerve i albueregion eller underarm UNS	0			
DS55;Læsion af blodkar i albueregion og underarm	0			
DS550;Læsion af arteria ulnaris i albueregion eller underarm	0			
DS551;Læsion af arteria radialis i albueregion eller underarm	0			
DS552;Læsion af vene i albueregion eller underarm	0			
DS557;Multiple karlæsioner i albueregion eller underarm	0			
DS558;Læsion af andet blodkar i albueregion eller underarm	0			

DS559;Læsion af blodkar i albueregion eller underarm UNS	0			
DS56;Læsion af muskler og sener i underarm	0			
DS560;Læsion af muskel eller sene af fleksor til tommelfinger på underarm	0			
DS561;Læsion af muskel eller sene af lang fleksor til anden specificeret finger på underarm	0			
DS562;Læsion af muskel eller sene af anden fleksor på underarm	0			
DS563;Læsion af muskel eller sene af ekstensor eller abduktor til tommelfinger på underarm	0			
DS564;Læsion af muskel eller sene af ekstensor eller abduktor til anden specificeret finger på und	0			
DS565;Læsion af muskel eller sene af anden ekstensor på underarm	0			
DS567;Multiple læsioner af muskler eller sener i albueregion eller underarm	0			
DS567A;Multiple muskellæsioner på underarm	0			
DS567B;Multiple senelæsioner i albueregion eller underarm	0			
DS568;Læsion af anden eller ikke specificeret muskel eller sene i albueregion eller underarm	0			
DS57;Knusningslæsion af albue og underarm	0			
DS570;Knusningslæsion af albue	0			
DS578;Anden knusningslæsion af underarm	0			
DS579;Knusningslæsion af underarm UNS	0			
DS58;Traumatisk amputation af albue og underarm	0			
DS580;Traumatisk amputation i albue	0			
DS581;Traumatisk amputation på underarm	0			
DS589;Traumatisk amputation i albue eller på underarm UNS	0			
DS59;Andre og ikke specificerede læsioner af albue og underarm	0			
DS597;Multiple læsioner af albueregion eller underarm	0			
DS598;Anden læsion af albueregion eller underarm	0			
DS599;Læsion af albueregion eller underarm UNS	0			
DS60;Overfladisk læsion af håndled og hånd	0			
DS600;Kontusion af finger uden beskadigelse af negl	0			
DS600A;Kontusion af finger UNS	0			
DS601;Kontusion af finger med beskadigelse af negl	0			
DS601A;Subungvalt hæmatom på finger	0			
DS601B;Skade på negleleje på finger	0			
DS602;Kontusion af håndled eller hånd	0			
DS602A;Kontusion af håndled	0			

DS602B;Kontusion af hånd UNS	0		
DS607;Multiple overfladiske læsioner af håndled eller hånd	0		
DS607A;Multiple overfladiske læsioner af håndled	0		
DS607B;Multiple overfladiske læsioner af hånd	0		
DS608;Anden overfladisk læsion af håndled eller hånd	0		
DS609;Overfladisk læsion af håndled eller hånd UNS	0		
DS61;Åbent sår på håndled og hånd	0		
DS610;Åbent sår på finger uden beskadigelse af negl	0		
DS610A;Åbent sår på finger UNS	0		
DS611;Åbent sår på finger med beskadigelse af negl	0		
DS617;Multiple åbne sår på håndled eller hånd	0		
DS617A;Multiple åbne sår på håndled	0		
DS617B;Multiple åbne sår på hånd	0		
DS618;Åbent sår på anden del af håndled eller hånd	0		
DS619;Åbent sår på håndled eller hånd UNS	0		
DS62;Fraktur af håndled og hånd	0		
DS620;Fraktur af os scaphoideum	0		
DS621;Fraktur af anden håndrodsknogle	0		
DS621A;Fraktur af os hamatum	0		
DS621B;Fraktur af os lunatum	0		
DS621C;Fraktur af os pisiformis	0		
DS621D;Fraktur af os trapezoideum	0		
DS621E;Fraktur af os triquetum	0		
DS622;Fraktur af 1. metakarpalknogle	0		
DS622A;Bennetts fraktur	0		
DS623;Fraktur af anden specificeret metakarpalknogle	0		
DS623A;Fraktur af 2. metakarpalknogle	0		
DS623B;Fraktur af 3. metakarpalknogle	0		
DS623C;Fraktur af 4. metakarpalknogle	0		
DS623D;Fraktur af 5. metakarpalknogle	0		
DS624;Multiple frakturer i metakarpalknogler	0		
DS625;Fraktur af tommelfinger	0		

Supplement 2 Nielsen et al. Mortality after paediatric emergency calls for patients with and without pre-existing comorbidity (2023)

DS625A;Fraktur af tommelfingers proksimale phalanx	0			
DS625B;Fraktur af tommelfingers distale phalanx	0			
DS626;Fraktur af anden specificeret finger	0			
DS626C;Fractura digiti I phal III manus (findes ikke i SKS)	0			
DS626D;Fraktur af 2. fingers proksimale phalanx	0			
DS626E;Fraktur af 2. fingers midterste phalanx	0			
DS626F;Fraktur af 2. fingers distale phalanx	0			
DS626G;Fraktur af 3. fingers proksimale phalanx	0			
DS626H;Fraktur af 3. fingers midterste phalanx	0			
DS626I;Fraktur af 3. fingers distale phalanx	0			
DS626J;Fraktur af 4. fingers proksimale phalanx	0			
DS626K;Fraktur af 4. fingers midterste phalanx	0			
DS626L;Fraktur af 4. fingers distale phalanx	0			
DS626M;Fraktur af 5. fingers proksimale phalanx	0			
DS626N;Fraktur af 5. fingers midterste phalanx	0			
DS626O;Fraktur af 5. fingers distale phalanx	0			
DS627;Multiple fingerfrakturer	0			
DS628;Fraktur af anden eller ikke specificeret del af håndled eller hånd	0			
DS628A;Fraktur af håndled UNS	0			
DS628B;Fraktur af hånd UNS	0			
DS63;Luksation og distorsion af led og ligamenter i håndled og hånd	0			
DS630;Luksation i håndled UNS	0			
DS630B;Luksation i karpometakarpalled UNS	0			
DS630C;Luksation i tommelfingers karpometakarpalled	0			
DS630D;Luksation i radiokarpalled	0			
DS630E;Luksation af håndrodsknogle UNS	0			
DS630F;Luksation af os lunatum	0			
DS630G;Luksation af os scaphoideum	0			
DS630H;Distal radius-luksation	0			
DS630I;Distal ulna-luksation	0			
DS631;Luksation i fingerled	0			
DS631A;Luksation i tommelfingers interfalangealled	0			

Supplement 2 Nielsen et al. Mortality after paediatric emergency calls for patients with and without pre-existing comorbidity (2023)

DS631B;Luksation i 2. fingers distale interfalangealled	0			
DS631C;Luksation i 3. fingers distale interfalangealled	0			
DS631D;Luksation i 4. fingers distale interfalangealled	0			
DS631E;Luksation i 5. fingers distale interfalangealled	0			
DS631F;Luksation i 2. fingers proksimale interfalangealled	0			
DS631G;Luksation i 3. fingers proksimale interfalangealled	0			
DS631H;Luksation i 4. fingers proksimale interfalangealled	0			
DS631I;Luksation i 5. fingers proksimale interfalangealled	0			
DS631J;Luksation i tommelfingers metakarpofalangealled	0			
DS631K;Luksation i 2. fingers metakarpofalangealled	0			
DS631L;Luksation i 3. fingers metakarpofalangealled	0			
DS631M;Luksation i 4. fingers metakarpofalangealled	0			
DS631N;Luksation i 5. fingers metakarpofalangealled	0			
DS632;Multiple fingerluksationer	0			
DS633;Traumatisk ruptur af ligament i håndled eller hånd	0			
DS633A;Traumatisk ruptur af radiokarpalligament	0			
DS633B;Traumatisk ruptur af ulnokarpalligament	0			
DS634;Traumatisk ruptur af metakarpofalangealligament eller interfalangealligament	0			
DS634A;Traumatisk ruptur af interfalangealligament i tommelfinger	0			
DS634B;Traumatisk ruptur af interfalangealligament i 2. finger	0			
DS634C;Traumatisk ruptur af interfalangealligament i 3. finger	0			
DS634D;Traumatisk ruptur af interfalangealligament i 4. finger	0			
DS634E;Traumatisk ruptur af interfalangealligament i 5. finger	0			
DS634F;Traumatisk ruptur af metakarpofalangealligament i tommelfinger	0			
DS634G;Traumatisk ruptur af metakarpofalangealligament i 2. finger	0			
DS634H;Traumatisk ruptur af metakarpofalangealligament i 3. finger	0			
DS634I;Traumatisk ruptur af metakarpofalangealligament i 4. finger	0			
DS634J;Traumatisk ruptur af metakarpofalangealligament i 5. finger	0			
DS635;Distorsion af håndled	0			
DS636;Distorsion af fingerled	0			
DS636A;Distorsion af interfalangealled i tommelfinger	0			
DS636B;Distorsion af interfalangealled i 2. finger	0			

DS636C;Distorsion af interfalangealled i 3. finger	0			
DS636D;Distorsion af interfalangealled i 4. finger	0			
DS636E;Distorsion af interfalangealled i 5. finger	0			
DS636F;Distorsion af metakarpofalangealled i tommelfinger	0			
DS636G;Distorsion af metakarpofalangealled i 2. finger	0			
DS636H;Distorsion af metakarpofalangealled i 3. finger	0			
DS636I;Distorsion af metakarpofalangealled i 4. finger	0			
DS636J;Distorsion af metakarpofalangealled i 5. finger	0			
DS636K;Distorsion af tommelfinger UNS	0			
DS637;Distorsion af anden eller ikke specificeret del af håndled eller hånd	0			
DS64;Læsion af nerver i håndled og hånd	0			
DS640;Læsion af nervus ulnaris i håndled eller hånd	0			
DS641;Læsion af nervus medianus i håndled eller hånd	0			
DS642;Læsion af nervus radialis i håndled eller hånd	0			
DS643;Læsion af nerve i tommelfinger	0			
DS644;Læsion af nerve i anden specificeret finger	0			
DS644A;Læsion af nerve i 2. finger	0			
DS644B;Læsion af nerve i 3. finger	0			
DS644C;Læsion af nerve i 4. finger	0			
DS644D;Læsion af nerve i 5. finger	0			
DS647;Multiple nervelæsioner i håndled eller hånd	0			
DS648;Læsion af anden nerve i håndled eller hånd	0			
DS649;Læsion af nerve i håndled eller hånd UNS	0			
DS65;Læsion af blodkar i håndled og hånd	0			
DS650;Læsion af arteria ulnaris i håndled eller hånd	0			
DS651;Læsion af arteria radialis i håndled eller hånd	0			
DS652;Læsion af arcus palmaris superficialis	0			
DS653;Læsion af arcus palmaris profundus	0			
DS654;Læsion af blodkar i tommelfinger	0			
DS655;Læsion af blodkar i anden specificeret finger	0			
DS657;Multiple karlæsioner på håndled eller hånd	0			
DS658;Læsion af andet blodkar i håndled eller hånd	0			

DS659;Læsion af blodkar i håndled eller hånd UNS	0		
DS66;Læsion af muskler og sener i håndled og hånd	0		
DS660;Læsion af lang fleksorsene til tommelfinger i håndled eller hånd	0		
DS661;Læsion af fleksorsene til anden specificeret finger i håndled eller hånd	0		
DS662;Læsion af ekstensorsene til tommelfinger i håndled eller hånd	0		
DS663;Læsion af ekstensorsene til anden specificeret finger i håndled eller hånd	0		
DS664;Læsion af kort muskel eller sene til tommelfinger	0		
DS665;Læsion af kort muskel eller sene til anden specificeret finger	0		
DS666;Multiple læsioner af fleksormuskler eller sener i håndled eller hånd	0		
DS667;Multiple læsioner af ekstensormuskler eller sener i håndled eller hånd	0		
DS668;Læsion af anden muskel eller sene i håndled eller hånd	0		
DS669;Læsion af muskel eller sene i håndled eller hånd UNS	0		
DS67;Knusningslæsion af håndled og hånd	0		
DS670;Knusningslæsion af finger	0		
DS670A;Knusningslæsion af tommelfinger	0		
DS670B;Knusningslæsion af 2. finger	0		
DS670C;Knusningslæsion af 3. finger	0		
DS670D;Knusningslæsion af 4. finger	0		
DS670E;Knusningslæsion af 5. finger	0		
DS678;Anden eller ikke specificeret knusningslæsion i håndled eller hånd	0		
DS678A;Knusningslæsion af håndled UNS	0		
DS678B;Knusningslæsion af hånd UNS	0		
DS68;Traumatisk amputation af håndled og hånd	0		
DS680;Traumatisk amputation af tommelfinger	0		
DS681;Traumatisk amputation af anden specificeret finger	0		
DS681A;Traumatisk amputation af 2. finger	0		
DS681B;Traumatisk amputation af 3. finger	0		
DS681C;Traumatisk amputation af 4. finger	0		
DS681D;Traumatisk amputation af 5. finger	0		
DS682;Traumatisk amputation af flere fingre	0		
DS683;Traumatisk amputation af flere fingre og dele af hånd eller håndled	0		
DS684;Traumatisk amputation af hånd	0		

DS688;Traumatisk amputation af anden del af håndled eller hånd	0			
DS689;Traumatisk amputation af håndled eller hånd UNS	0			
DS69;Andre og ikke specificerede læsioner af håndled og hånd	0			
DS697;Multiple læsioner af hånd eller håndled UNS	0			
DS697A;Multiple læsioner af håndled UNS	0			
DS697B;Multiple læsioner af hånd UNS	0			
DS698;Anden læsion af håndled eller hånd	0			
DS699;Læsion af håndled eller hånd UNS	0			
DS70;Overfladisk læsion af hofteregion og lår	0			
DS700;Kontusion af hofte	0			
DS701;Kontusion af lår	0			
DS707;Multiple overfladiske læsioner af hofteregion eller lår	0			
DS707A;Multiple overfladiske læsioner af hofteregion	0			
DS707B;Multiple overfladiske læsioner af lår	0			
DS708;Anden overfladisk læsion af hofteregion eller lår	0			
DS709;Overfladisk læsion af hofteregion eller lår UNS	0			
DS71;Åbent sår på hofteregion og lår	0			
DS710;Åbent sår på hofteregion	0			
DS711;Åbent sår på lår	0			
DS717;Multiple åbne sår på hofteregion eller lår	0			
DS717A;Multiple åbne sår på hofteregion	0			
DS717B;Multiple åbne sår på lår	0			
DS718;Åbent sår på anden eller ikke specificeret del af hofteregion eller lår	0			
DS72;Fraktur af femur	0			
DS720;Fraktur af lårbenshals	0			
DS721;Pertrokantær femurfraktur	0			
DS721A;Intertrokantær femurfraktur	0			
DS721B;Trokantær femurfraktur	0			
DS722;Subtrokantær femurfraktur	0			
DS723;Fraktur af femurskaft	0			
DS724;Fraktur af distale del af femur	0			
DS724A;Fraktur af femurkondyl	0			

DS724B;Interkondylær femurfraktur	0		
DS724C;Suprakondylær femurfraktur	0		
DS727;Multiple frakturer af femur	0		
DS728;Fraktur af anden del af femur	0		
DS728A;Fraktur af femurhoved	0		
DS729;Fraktur af femur UNS	0		
DS73;Luksation og distorsion af hofte	0		
DS730;Luksation i hofte	0		
DS731;Distorsion af hofte	0		
DS74;Læsion af nerver i hofteregion og i lår	0		
DS740;Læsion af nervus ischiadicus i hofteregion eller lår	0		
DS741;Læsion af nervus femoralis i hofteregion eller lår	0		
DS742;Læsion af sensorisk nerve i hofteregion eller lår	0		
DS747;Multiple nervelæsioner i hofteregion eller lår	0		
DS748;Læsion af anden nerve i hofteregion eller lår	0		
DS749;Læsion af nerve i hofteregion eller lår UNS	0		
DS75;Læsion af blodkar i hofteregion og lår	0		
DS750;Læsion af arteria femoralis	0		
DS751;Læsion af vena femoralis på lårniveau	0		
DS752;Læsion af vena saphena magna på lårniveau	0		
DS757;Multiple karlæsioner i hofteregion og lår	0		
DS758;Læsion af andet blodkar i hofteregion eller lår	0		
DS759;Læsion af blodkar i hofteregion eller lår UNS	0		
DS76;Læsion af muskler og sener i hofteregion og lår	0		
DS760;Læsion af muskel eller sene i hofteregion	0		
DS760A;Læsion af muskel i hofteregion	0		
DS760B;Læsion af sene i hofteregion	0		
DS761;Læsion af muskel og sene af musculus quadriceps femoris	0		
DS761A;Læsion af musculus quadriceps femoris	0		
DS761B;Læsion af ligamentum patellae	0		
DS762;Læsion af muskel og sene af musculus adductor femoris	0		
DS762A;Læsion af musculus adductor femoris	0		

DS762B;Læsion af sene af musculus adductor femoris	0		
DS763;Læsion af muskel eller sene på bagsiden af lår	0		
DS763A;Læsion af muskel på bagsiden af lår	0		
DS763B;Læsion af sene på bagsiden af lår	0		
DS764;Læsion af anden eller ikke specificeret muskel eller sene på lår	0		
DS767;Multiple læsioner af muskler eller sener i hofteregion eller lår	0		
DS77;Knusningslæsion af hofteregion og lår	0		
DS770;Knusningslæsion af hofteregion	0		
DS771;Knusningslæsion af lår	0		
DS772;Knusningslæsion af både hofteregion og lår	0		
DS78;Traumatisk amputation af hofte og lår	0		
DS780;Traumatisk amputation i hofteled	0		
DS781;Traumatisk amputation mellem hofte og knæ	0		
DS789;Traumatisk amputation i hofteregion eller på lår UNS	0		
DS79;Andre og ikke specificerede læsioner af hofteregion og lår	0		
DS797;Multiple læsioner af hofteregion eller lår UNS	0		
DS797A;Multiple læsioner af hofteregion	0		
DS797B;Multiple læsioner af lår	0		
DS798;Anden læsion af hofte eller lår	0		
DS799;Læsion af hofte eller lår UNS	0		
DS80;Overfladisk læsion af knæ og underben	0		
DS800;Kontusion af knæ	0		
DS801;Kontusion af underben	0		
DS801A;Contusio cruris (findes ikke i SKS)	0		
DS807;Multiple overfladiske læsioner af knæ eller underben	0		
DS808;Anden overfladisk læsion af knæ eller underben	0		
DS809;Overfladisk læsion af knæ eller underben UNS	0		
DS81;Åbent sår på knæ og underben	0		
DS810;Åbent sår på knæ	0		
DS817;Multiple åbne sår på knæ eller underben	0		
DS818;Åbent sår på anden del af underben	0		
DS819;Åbent sår på underben UNS	0		

DS82;Fraktur af knæ, underben og ankel	0			
DS820;Fraktur af knæskal	0			
DS821;Fraktur af proksimale ende af tibia	0			
DS821A;Fraktur af begge tibiakondyler	0			
DS821B;Fraktur af laterale tibiakondyl	0			
DS821C;Fraktur af mediale tibiakondyl	0			
DS821D;Fraktur af eminentia intercondyloidea tibiae	0			
DS822;Fraktur af tibiaskaftet	0			
DS823;Fraktur af distale ende af tibia	0			
DS823A;Fraktur af tibias bagkant	0			
DS823C;Potts fraktur	0			
DS824;Fraktur af fibulaskaftet	0			
DS825;Fraktur af mediale malleol	0			
DS826;Fraktur af laterale malleol	0			
DS827;Multiple frakturer i knæ eller underben	0			
DS827A;Bimalleolær fraktur	0			
DS827B;Trimalleolær fraktur	0			
DS828;Fraktur af anden del af underben	0			
DS828A;Fractura bimalleolaris (findes ikke i SKS)	0			
DS828B;Malleolfraktur UNS	0			
DS828D;Ankelfraktur UNS	0			
DS829;Fraktur af underben UNS	0			
DS83;Luksation og distorsion af led og ligamenter i knæ	0			
DS830;Luksation af patella	0			
DS831;Luksation i knæled	0			
DS831A;Luksation i tibiofibularled	0			
DS832;Traumatisk ruptur af menisk i knæled	0			
DS832A;Traumatisk ruptur af ligamentum meniscofemorale posterius	0			
DS832B;Traumatisk ruptur af ligamentum meniscofemorale anterius	0			
DS833;Fraktur af ledbrusk i knæled	0			
DS834;Læsion af kollateralligament i knæ	0			
DS834A;Læsion af lateralt kollateralligament i knæ	0			

DS834B;Læsion af medialt kollateralligament i knæ	0			
DS834C;Læsion af både lateralt og medialt kollateralligament i knæ	0			
DS835;Læsion af korsbånd i knæled	0			
DS835A;Læsion af begge korsbånd i knæled	0			
DS835B;Læsion af forreste korsbånd i knæled	0			
DS835C;Læsion af bageste korsbånd i knæled	0			
DS835D;Traumatisk ruptur af begge korsbånd i knæled	0			
DS835E;Traumatisk ruptur af forreste korsbånd i knæled	0			
DS835F;Traumatisk ruptur af bageste korsbånd i knæled	0			
DS836;Distorsion af anden eller ikke specificeret struktur i knæled	0			
DS836A;Distorsion af knæled med ligamentlæsion	0			
DS836B;Distorsion af articulatio tibiofibularis superioris	0			
DS836C;Distorsion af ligamentum patellae	0			
DS836D;Distorsion af ligamentum tibiofibularis superior	0			
DS837;Multiple læsioner af strukturer i knæled	0			
DS837B;Multiple læsioner af ligamenter i knæled	0			
DS837C;Multiple læsioner af ligamenter og menisker i knæled	0			
DS84;Læsion af nerver i knæregion og underben	0			
DS840;Læsion af nervus tibialis i knæregion eller underben	0			
DS841;Læsion af nervus peroneus i knæregion eller underben	0			
DS842;Læsion af sensorisk nerve i knæregion eller underben	0			
DS847;Multiple nervelæsioner i knæregion eller underben	0			
DS848;Læsion af anden nerve i knæregion eller underben	0			
DS849;Læsion af nerve i knæregion eller underben UNS	0			
DS85;Læsion af blodkar i knæregion og underben	0			
DS850;Læsion af arteria poplitea	0			
DS851;Læsion af arteria tibialis anterior eller posterior	0			
DS851A;Læsion af arteria tibialis anterior	0			
DS851B;Læsion af arteria tibialis posterior	0			
DS852;Læsion af arteria peronea	0			
DS853;Læsion af vena saphena magna i knæregion eller underben	0			
DS854;Læsion på crus af vena saphena parva i knæregion eller underben	0			

DS855;Læsion af vena poplitea	0			
DS857;Multiple karlæsioner i knæregion eller underben	0			
DS858;Læsion af andet blodkar i knæregion eller underben	0			
DS859;Læsion af blodkar i knæregion eller underben UNS	0			
DS86;Beskadigelse af muskler og sener i knæregion eller underben	0			
DS860;Læsion af akillessene	0			
DS860A;Traumatisk ruptur af akillessene	0			
DS861;Læsion af anden muskel eller sene på bagsiden af underben	0			
DS861A;Læsion af musculus flexor hallucis longus	0			
DS861B;Læsion af muskel på bagsiden af underben	0			
DS861C;Læsion af sene på bagsiden af underben	0			
DS861D;Læsion af tendo musculis flexoris hallucis longus	0			
DS862;Læsion af muskel eller sene på forsiden af underben	0			
DS862A;Læsion af musculus extensor digiti longi pedis	0			
DS862B;Læsion af musculus extensor hallucis longi	0			
DS862C;Læsion af muskel på forsiden af underben UNS	0			
DS862D;Læsion af musculus tibialis anterior	0			
DS862E;Læsion af sene til musculus extensor digiti longi pedis	0			
DS862F;Læsion af sene til musculus extensor hallucis longi	0			
DS862G;Læsion af sene til musculus tibialis anterior	0			
DS862H;Læsion af sene på forsiden af underben UNS	0			
DS863;Læsion af peroneus muskel eller sene	0			
DS863A;Læsion af musculus peroneus	0			
DS863B;Læsion af sene til musculus peroneus	0			
DS867;Multiple læsioner af muskler eller sener i knæregion eller underben	0			
DS868;Læsion af anden muskel eller sene i knæregion eller underben	0			
DS868A;Læsion af muskel eller sene i knæhase	0			
DS869;Læsion af muskel eller sene i knæregion eller underben UNS	0			
DS87;Knusningslæsion af knæ og crus	0			
DS870;Knusningslæsion af knæ	0			
DS878;Knusningslæsion af anden eller ikke specificeret del af underben	0			
DS88;Traumatisk amputation i knæregion eller underben	0			

DS880;Traumatisk amputation i knæled	0			
DS881;Traumatisk amputation på underben	0			
DS889;Traumatisk amputation i knæregion eller på underben UNS	0			
DS89;Andre og ikke specificerede læsioner af knæregion og underben	0			
DS897;Multiple læsioner i knæregion eller på underben	0			
DS898;Anden læsion i knæregion eller på underben	0			
DS898A;Traumatisk ruptur af membrana interossea cruris	0			
DS899;Læsion i knæregion eller på underben UNS	0			
DS90;Overfladisk læsion af ankel og fod	0			
DS900;Kontusion af ankel	0			
DS901;Kontusion af tå uden beskadigelse af negl	0			
DS901A;Kontusion af tå UNS	0			
DS902;Kontusion af tå med beskadigelse af negl	0			
DS902A;Subungvalt hæmatom på tå	0			
DS902B;Skade på negleleje på tå	0			
DS903;Kontusion af anden eller ikke specificeret del af fod	0			
DS903A;Kontusion af fod UNS	0			
DS903B;Contusio pedis uden specifikation (findes ikke i SKS)	0			
DS907;Multiple overfladiske læsioner af ankel eller fod	0			
DS907A;Multiple overfladiske læsioner af fod	0			
DS907B;Multiple overfladiske læsioner af ankel	0			
DS908;Anden overfladisk læsion af ankel eller fod	0			
DS909;Overfladisk læsion af ankel eller fod UNS	0			
DS91;Åbent sår på ankel og fod	0			
DS910;Åbent sår på ankel	0			
DS911;Åbent sår på tå uden beskadigelse af negl	0			
DS911A;Åbent sår på tå UNS	0			
DS912;Åbent sår på tå med beskadigelse af negl	0			
DS913;Åbent sår på anden del af fod	0			
DS913A;Åbent sår på fod UNS	0			
DS917;Multiple åbne sår på ankel og fod	0			
DS917A;Multiple åbne sår på fod	0			

DS917B;Multiple åbne sår på ankel	0		
DS918;Åbent sår på andre dele af ankel og fod (findes ikke i SKS)	0		
DS92;Fraktur af fod	0		
DS920;Fraktur af hælben	0		
DS920A;Fraktur af tuber calcanei	0		
DS921;Fraktur af talus	0		
DS922;Fraktur af anden fodrodsknogle	0		
DS922A;Fraktur af os cuboideum	0		
DS922B;Fraktur af os cuneiforme mediale	0		
DS922C;Fraktur af os naviculare pedis	0		
DS922D;Fraktur af os cuneiforme intermedium	0		
DS922E;Fraktur af os cuneiforme laterale	0		
DS923;Fraktur af metatarsalknogle UNS	0		
DS923A;Fraktur af 1. metatarsalknogle	0		
DS923B;Fraktur af 2. metatarsalknogle	0		
DS923C;Fraktur af 3. metatarsalknogle	0		
DS923D;Fraktur af 4. metatarsalknogle	0		
DS923E;Fraktur af 5. metatarsalknogle	0		
DS924;Fraktur af storetå	0		
DS925;Fraktur af anden specificeret tå	0		
DS925A;Fraktur af 2. tå	0		
DS925B;Fraktur af 3. tå	0		
DS925C;Fraktur af 4. tå	0		
DS925D;Fraktur af 5. tå	0		
DS926;Lisfranc læsion	0		
DS926A;Lisfranc læsion uden luksation	0		
DS926B;Lisfranc læsion med luksation	0		
DS927;Multiple frakturer af fod	0		
DS929;Fraktur af fod UNS	0		
DS93;Luksation og distorsion af led og ligamenter i ankel og fod	0		
DS930;Luksation i ankelled	0		
DS930B;Luksation af distale ende af fibula	0		

Supplement 2 Nielsen et al. Mortality after paediatric emergency calls for patients with and without pre-existing comorbidity (2023)

DS930C;Luksation af distale ende af tibia	0			
DS931;Luksation i tåled	0			
DS931A;Luksation i interfalangealled i 2. tå	0			
DS931B;Luksation i interfalangealled i 3. tå	0			
DS931C;Luksation i interfalangealled i 4. tå	0			
DS931D;Luksation i interfalangealled i 5. tå	0			
DS931E;Luksation i metatarsofalangealled i 2. tå	0			
DS931F;Luksation i metatarsofalangealled i 3. tå	0			
DS931G;Luksation i metatarsofalangealled i 4. tå	0			
DS931H;Luksation i metatarsofalangealled i 5. tå	0			
DS931I;Luksation i interfalangealled i storetå	0			
DS931J;Luksation i metatarsofalangealled i storetå	0			
DS932;Traumatisk ruptur af ligament i ankel eller fod	0			
DS932A;Traumatisk ruptur af ligamentum deltoideum pedis	0			
DS932B;Traumatisk ruptur af ligament i fod UNS	0			
DS932C;Traumatisk ruptur af ligament i ankel UNS	0			
DS932D;Traumatisk ruptur af ligamentum talocalcaneofibulare	0			
DS932E;Traumatisk ruptur af ligamentum tarsi	0			
DS932F;Traumatisk ruptur af ligamentum tibiofibularis distalis	0			
DS933;Luksation af anden eller ikke specificeret struktur i fod	0			
DS933B;Luksation i tarsometatarsalled (findes ikke i SKS)	0			
DS933C;Luksation i os naviculare pedis	0			
DS934;Distorsion af ankel UNS	0			
DS934A;Distorsion af ligamentum calcaneofibulare	0			
DS934B;Distorsion af ligamentum deltoideum	0			
DS934C;Distorsion af ligamentum talofibulare anterior	0			
DS934D;Distorsion af ligamentum talofibulare posterior	0			
DS934E;Fodrodsdistorsion	0			
DS935;Distorsion af tå	0			
DS935A;Distorsion af interfalangealled i storetå	0			
DS935B;Distorsion af interfalangealled i 2. tå	0			
DS935C;Distorsion af interfalangealled i 3. tå	0			

DS935D;Distorsion af interfalangealled i 4. tå	0		
DS935E;Distorsion af interfalangealled i 5. tå	0		
DS935F;Distorsion af metatarsofalangealled i storetå	0		
DS935G;Distorsion af metatarsofalangealled i 2. tå	0		
DS935H;Distorsion af metatarsofalangealled i 3. tå	0		
DS935I;Distorsion af metatarsofalangealled i 4. tå	0		
DS935J;Distorsion af metatarsofalangealled i 5. tå	0		
DS936;Distorsion af anden eller ikke specificeret struktur i fod	0		
DS936A;Distorsion af ligamentum talocalcaneum laterale	0		
DS936B;Distorsion af ligamentum talocalcaneum mediale	0		
DS936C;Distorsion af ligameni tarsi dorsalia	0		
DS936D;Distorsion af ligamentum tarsometatarsalis	0		
DS94;Læsion af nerver i ankel og fod	0		
DS940;Læsion af nervus plantaris lateralis	0		
DS941;Læsion af nervus plantaris medialis	0		
DS942;Læsion af nervus peroneus profundus i ankel eller fod	0		
DS943;Læsion af sensorisk nerve i ankel eller fod	0		
DS947;Multiple nervelæsioner i ankel eller fod	0		
DS948;Læsion af anden nerve i ankel eller fod	0		
DS949;Læsion af nerve i ankel eller fod UNS	0		
DS95;Beskadigelse af blodkar i ankel og fod	0		
DS950;Læsion af dorsal fodarterie	0		
DS951;Læsion af plantar fodarterie	0		
DS952;Læsion af dorsal fodvene	0		
DS957;Multiple karlæsioner i ankel eller fod	0		
DS958;Læsion af andet blodkar i ankel eller fod	0		
DS959;Læsion af blodkar i ankel eller fod UNS	0		
DS96;Læsion af muskler og sener i ankel og fod	0		
DS960;Læsion af lang fleksorsene i ankel eller fod	0		
DS960A;Læsion af lang fleksorsene i fod	0		
DS960B;Læsion af lang fleksorsene i ankel	0		
DS961;Læsion af lang ekstensorsene i ankel eller fod	0		

DS961A;Læsion af lang ekstensorsene i fod	0			
DS961B;Læsion af lang ekstensorsene i ankel	0			
DS962;Læsion af kort muskel eller sene i fod	0			
DS967;Multiple læsioner af muskler eller sener i ankel eller fod	0			
DS968;Læsion af anden muskel eller sene i ankel eller fod	0			
DS969;Læsion af muskel eller sene i ankel eller fod UNS	0			
DS97;Knusningslæsion af ankel og fod	0			
DS970;Knusningslæsion af ankel	0			
DS971;Knusningslæsion af tå	0			
DS978;Anden eller ikke specificeret knusningslæsion i ankel eller fod	0			
DS978A;Knusningslæsion af fod UNS	0			
DS98;Traumatisk amputation af ankel og fod	0			
DS980;Traumatisk amputation på ankelniveau	0			
DS981;Traumatisk amputation af en enkelt tå	0			
DS981A;Traumatisk amputation af storetå	0			
DS981B;Traumatisk amputation af 2. tå	0			
DS981C;Traumatisk amputation af 3. tå	0			
DS981D;Traumatisk amputation af 4. tå	0			
DS981E;Traumatisk amputation af 5. tå	0			
DS982;Traumatisk amputation af flere tæer	0			
DS983;Traumatisk amputation af anden del af fod	0			
DS984;Traumatisk amputation af fod UNS	0			
DS99;Andre og ikke specificerede læsioner af ankel og fod	0			
DS997;Multiple læsioner af ankel og fod	0			
DS998;Anden læsion af ankel eller fod	0			
DS999;Læsion af ankel eller fod UNS	0			
DT00;Overfladiske læsioner af flere legemsregioner	0			
DT000;Overfladiske læsioner af både hovedet og hals	0			
DT001;Overfladiske læsioner af både thorax, abdomen, lænden og bækkenet	0			
DT002;Overfladiske læsioner af flere regioner på overekstremitet	0			
DT003;Overfladiske læsioner af flere regioner på underekstremitet	0			
DT006;Overfladiske læsioner af både arm(e) og ben	0			

Supplement 2 Nielsen et al. Mortality after paediatric emergency calls for patients with and without pre-existing comorbidity (2023)

DT008;Overfladiske læsioner af andre kombinationer af legemsregioner	0			
DT009;Multiple overfladiske læsioner UNS	0			
DT009A;Mygstik (findes ikke i SKS)	0			
DT01;Åbne sår på flere legemsregioner	0			
DT010;Åbne sår på både hovedet og halsen	0			
DT011;Åbne sår på både thorax, abdomen, lænden og bækkenet	0			
DT012;Åbne sår på flere regioner på overekstremitet	0			
DT013;Åbne sår på flere regioner på underekstremitet	0			
DT016;Åbne sår på både arm(e) og ben	0			
DT018;Åbne sår på andre kombinationer af legemsregioner	0			
DT019;Multiple åbne sår UNS	0			
DT02;Frakturer i flere legemsregioner	0			
DT020;Frakturer i både hovedet og halsen	0			
DT021;Frakturer i både thorax, lænden og bækkenet	0			
DT022;Multiple frakturer i flere regioner på samme overekstremitet	0			
DT023;Multiple frakturer i flere regioner på samme underekstremitet	0			
DT024;Dobbeltsidige frakturer på overekstremiteter	0			
DT025;Dobbeltsidige frakturer på underekstremiteter	0			
DT026;Frakturer af både arm(e) og ben	0			
DT027;Frakturer både i thorax, lænden, bækkenet, arm(e) og ben	0			
DT028;Frakturer i andre kombinationer af legemsregioner	0			
DT029;Multiple frakturer UNS	0			
DT03;Luksation og distorsion af led og ligamenter i flere legemsregioner	0			
DT030;Luksationer eller distorsioner i både hovedet og halsen	0			
DT030A;Distorsioner i både hovedet og hals	0			
DT030B;Luksationer i både hovedet og hals	0			
DT031;Luksationer og distorsioner i både thorax, lænden og bækkenet	0			
DT032;Multiple luksationer eller distorsioner i flere regioner i overekstremitet(er)	0			
DT033;Multiple luksationer eller distorsioner i flere regioner i underekstremitet(er)	0			
DT034;Luksationer eller distorsioner i både arm(e) og ben	0			
DT038;Luksationer eller distorsioner i andre kombinationer af legemsregioner	0			
DT039;Multiple luksationer og distorsioner UNS	0			

DT04;Knusningslæsioner i flere legemsregioner	0			
DT040;Knusningslæsioner på både hovedet og halsen	0			
DT041;Knusningslæsioner i både thorax og abdomen, lænden og bækkenet	0			
DT042;Multiple knusningslæsioner på overekstremitet(er)	0			
DT043;Multiple knusningslæsioner på underekstremitet(er)	0			
DT044;Knusningslæsioner på både arm(e) og ben	0			
DT047;Knusningslæsioner på thorax og abdomen, lænden og bækkenet og ekstremiteter	0			
DT048;Knusningslæsioner på andre kombinationer af legemsregioner	0			
DT049;Multiple knusningslæsioner UNS	0			
DT05;Traumatisk amputation af flere legemsdele	0			
DT050;Traumatisk amputation af begge hænder	0			
DT051;Traumatisk amputation af hånd og modsidige arm	0			
DT052;Traumatisk amputation af begge arme	0			
DT053;Traumatisk amputation af begge fødder	0			
DT054;Traumatisk amputation af fod og modsidige ben	0			
DT055;Traumatisk amputation af begge ben	0			
DT056;Traumatisk amputation både af arm(e) og ben	0			
DT058;Traumatisk amputation med andre kombinationer af legemsregioner	0			
DT058A;Transectio abdominis	0			
DT058B;Transectio thoracis	0			
DT059;Traumatisk amputation af flere legemsdele UNS	0			
DT06;Andre læsioner af flere legemsregioner IKA	0			
DT060;Læsioner af både hjerne og hjernenerver samt rygmarven og nerver på halsniveau	0			
DT061;Læsioner af nerver og rygmarven som involverer flere legemsregioner	0			
DT062;Læsioner af nerver i flere legemsregioner	0			
DT062A;Multiple nervelæsioner UNS	0			
DT063;Læsioner af blodkar i flere legemsregioner	0			
DT064;Læsioner af muskler og sener i flere legemsregioner	0			
DT065;Læsioner af intratorakale, intraabdominale organer og bækkenorganer	0			
DT068;Andre specificerede læsioner i flere legemsregioner	0			
DT07;Multiple læsioner uden nærmere specificeringer	0			
DT079;Multiple læsioner UNS	0			

DT08;Fraktur af rygsøjlen uden angivelse af niveau	0			
DT089;Fraktur af rygsøjlen UNS	0			
DT09;Andre læsioner af rygsøjle og krop uden angivelse af lokalisation	0			
DT090;Overfladisk læsion af kroppen UNS	0			
DT091;Åbent sår på kroppen UNS	0			
DT092;Luksation eller distorsion i kroppen UNS	0			
DT093;Læsion af rygmarven UNS	0			
DT094;Læsion af nerve, spinalnerve eller plexus i kroppen UNS	0			
DT095;Læsion af muskel eller sene i kroppen UNS	0			
DT096;Traumatisk amputation på kroppen uden angivelse af lokalisation	0			
DT098;Anden læsion på kroppen UNS	0			
DT099;Læsion på kroppen UNS	0			
DT10;Fraktur af overekstremitet uden angivelse af lokalisation	0			
DT109;Fraktur af arm UNS	0			
DT11;Andre læsioner af overekstremitet uden angivelse af lokalisation	0			
DT110;Overfladisk læsion af arm UNS	0			
DT111;Åbent sår på arm UNS	0			
DT112;Luksation eller distorsion på arm UNS	0			
DT112A;Distorsion på arm UNS	0			
DT112B;Luksation på arm UNS	0			
DT113;Læsion af nerve på arm UNS	0			
DT114;Læsion af blodkar i arm UNS	0			
DT115;Læsion af muskel eller sene i arm UNS	0			
DT115A;Muskellæsion på arm UNS	0			
DT115B;Senelæsion af sene på arm UNS	0			
DT116;Traumatisk amputation på arm UNS	0			
DT118;Anden læsion på arm UNS	0			
DT119;Læsion på arm UNS	0			
DT12;Fraktur af underekstremitet uden angivelse af lokalisation	0			
DT129;Fraktur af ben UNS	0			
DT13;Andre læsioner af underekstremitet uden angivelse af lokalisation	0			
DT130;Overfladisk læsion af ben UNS	0			

DT131;Åbent sår på ben UNS	0		
DT132;Luksation eller distorsion på ben UNS	0		
DT132A;Distorsion på ben UNS	0		
DT132B;Luksation på ben UNS	0		
DT133;Læsion af nerve på ben UNS	0		
DT134;Læsion af blodkar i ben UNS	0		
DT135;Læsion af muskel eller sene i ben UNS	0		
DT135A;Muskellæsion på ben UNS	0		
DT135B;Senelæsion på ben UNS	0		
DT136;Traumatisk amputation på ben UNS	0		
DT138;Anden læsion på ben UNS	0		
DT139;Læsion på ben UNS	0		
DT14;Læsion uden angivelse af legemsregion	0		
DT140;Overfladisk læsion uden angivelse af legemsregion	0		
DT140A;Vabel UNS	0		
DT140B;Kontusion UNS	0		
DT140C;Hudafskrabning UNS	0		
DT140D;Hæmatom UNS	0		
DT140E;Insektbid, ikke giftigt UNS	0		
DT140F;Blodudtrædning UNS	0		
DT140G;Fremmedlegeme i hud UNS	0		
DT140H;Insektstik (findes ikke i SKS)	0		
DT140J;Negleskade UNS	0		
DT140K;Subungvalt hæmatom UNS	0		
DT140L;Flåtbid UNS	0		
DT140M;Myggestik UNS	0		
DT141;Åbent sår uden angivelse af legemsregion	0		
DT141A;Laceration UNS	0		
DT141B;Bidsår UNS	0		
DT141C;Snitsår UNS	0		
DT142;Fraktur uden angivelse af legemsregion	0		
DT143;Luksation eller distorsion uden angivelse af legemsregion	0		

Supplement 2 Nielsen et al. Mortality after paediatric emergency calls for patients with and without pre-existing comorbidity (2023)

DT143A;Distorsion af led UNS	0		
DT143B;Distorsion af ligament UNS	0		
DT143C;Traumatisk hæmartrose UNS	0		
DT143D;Luksation i led UNS	0		
DT143E;Traumatisk ruptur af ligament UNS	0		
DT144;Læsion af nerve uden angivelse af legemsregion	0		
DT144A;Hæmatomyeli UNS	0		
DT144B;Traumatisk lammelse UNS	0		
DT144C;Traumatisk paraplegi UNS		1	
DT144D;Traumatisk tetraplegi UNS			2
DT145;Læsion af blodkar uden angivelse af legemsregion	0		
DT145A;Traumatisk aneurisme UNS	0		
DT146;Læsion af muskel eller sene uden angivelse af legemsregion	0		
DT146A;Læsion af muskel UNS	0		
DT146B;Læsion af sene UNS	0		
DT146C;Traumatisk ruptur af muskel UNS	0		
DT146D;Traumatisk ruptur af sene UNS	0		
DT147;Knusningslæsion eller traumatisk amputation uden angivelse af legemsregion	0		
DT148;Anden læsion uden angivelse af legemsregion	0		
DT149;Læsion uden angivelse af legemsregion	0		
DT15;Fremmedlegeme på øjets ydre overflade	0		
DT150;Fremmedlegeme i cornea	0		
DT151;Fremmedlegeme i konjunktiva	0		
DT158;Fremmedlegeme andet sted på øjes ydre overflade	0		
DT158A;Fremmedlegeme i punctum lacrimale	0		
DT159;Fremmedlegeme på øjets ydre overflade UNS	0		
DT16;Fremmedlegeme i øre	0		
DT169;Fremmedlegeme i øre UNS	0		
DT169A;Fremmedlegeme i ydre øre	0		
DT169B;Fremmedlegeme i indre øre	0		
DT169C;Fremmedlegeme i mellemøre	0		
DT169D;Fremmedlegeme i tuba auditiva	0		

Supplement 2 Nielsen et al. Mortality after paediatric emergency calls for patients with and without pre-existing comorbidity (2023)

DT17;Fremmedlegeme i luftvejene	0			
DT170;Fremmedlegeme i bihule	0			
DT171;Fremmedlegeme i næsen	0			
DT172;Fremmedlegeme i svælget	0			
DT173;Fremmedlegeme i larynx	0			
DT174;Fremmedlegeme i luftrøret	0			
DT175;Fremmedlegeme i bronkie	0			
DT178;Fremmedlegeme i anden lokalisation i luftvejene	0			
DT178A;Fremmedlegeme i lunge	0			
DT179;Fremmedlegeme i luftvejene UNS	0			
DT179A;Asfyksi forårsaget af fremmedlegeme i luftvejene	0			
DT18;Fremmedlegeme i fordøjelseskanalen	0			
DT180;Fremmedlegeme i mundhulen	0			
DT181;Fremmedlegeme i spiserøret	0			
DT182;Fremmedlegeme i mavesækken	0			
DT182A;Bezoar i mavesækken	0			
DT183;Fremmedlegeme i tyndtarmen	0			
DT183A;Bezoar i tyndtarmen	0			
DT184;Fremmedlegeme i tyktarmen	0			
DT184A;Fremmedlegeme i colon sigmoideum	0			
DT185;Fremmedlegeme i endetarm eller endetarmsåbning	0			
DT185A;Fremmedlegeme i endetarmsåbning	0			
DT185B;Fremmedlegeme i endetarm	0			
DT188;Fremmedlegeme i anden lokalistion i fordøjelseskanalen	0			
DT189;Fremmedlegeme i fordøjelseskanalen UNS	0			
DT19;Fremmedlegeme i urin- og kønsorganerne	0			
DT190;Fremmedlegeme i urinrøret	0			
DT191;Fremmedlegeme i urinblæren	0			
DT192;Fremmedlegeme i vulva eller vagina	0			
DT192A;Fremmedlegeme i vagina	0			
DT192B;Fremmedlegeme i vulva	0			
DT193;Fremmedlegeme i livmoderen	0			

DT198;Fremmedlegeme i anden lokalisation i urin- eller kønsorganer	0		
DT199;Fremmedlegeme i urin- eller kønsorganer UNS	0		
DT20;Forbrænding og ætsning på hovedet og halsen	0		
DT200;Forbrænding UNS på hovedet og halsen	0		
DT200A;Forbrænding UNS på hovedet	0		
DT200B;Forbrænding UNS på halsen	0		
DT201;1° forbrænding på hovedet og halsen	0		
DT201A;1° forbrænding på hovedet	0		
DT201B;1° forbrænding på halsen	0		
DT202;2° forbrænding på hovedet og halsen	0		
DT202A;2° forbrænding på hovedet	0		
DT202B;2° forbrænding på halsen	0		
DT203;3° forbrænding på hovedet og halsen		1	
DT203A;3° forbrænding på hovedet		1	
DT203B;3° forbrænding på halsen		1	
DT204;Ætsning UNS på hovedet og halsen	0		
DT204A;Ætsning UNS på hovedet	0		
DT204B;Ætsning UNS på halsen	0		
DT205;1° ætsning på hovedet og halsen	0		
DT205A;1° ætsning på hovedet	0		
DT205B;1° ætsning på halsen	0		
DT206;2° ætsning på hovedet og halsen	0		
DT206A;2° ætsning på hovedet	0		
DT206B;2° ætsning på halsen	0		
DT207;3° ætsning på hovedet og halsen		1	
DT207A;3° ætsning på hovedet		1	
DT207B;3° ætsning på halsen		1	
DT21;Forbrænding og ætsning på kroppen	0		
DT210;Forbrænding UNS på kroppen	0		
DT211;1° forbrænding på kroppen	0		
DT212;2° forbrænding på kroppen	0		
DT213;3° forbrænding på kroppen		1	

DT214;Ætsning UNS på kroppen	0		
DT215;1° ætsning på kroppen	0		
DT216;2° ætsning på kroppen	0		
DT217;3° ætsning på kroppen		1	
DT22;Forbrænding og ætsning på skulder og arm	0		
DT220;Forbrænding UNS på overekstremitet	0		
DT220A;Forbrænding UNS på skulder	0		
DT220B;Forbrænding UNS på arm	0		
DT221;1° forbrænding på overekstremitet	0		
DT221A;1° forbrænding på skulder	0		
DT221B;1° forbrænding på arm	0		
DT222;2° forbrænding på overekstremitet	0		
DT222A;2° forbrænding på skulder	0		
DT222B;2° forbrænding på arm	0		
DT223;3° forbrænding på overekstremitet		1	
DT223A;3° forbrænding på skulder		1	
DT223B;3° forbrænding på arm		1	
DT224;Ætsning UNS på overekstremitet	0		
DT224A;Ætsning UNS på skulder	0		
DT224B;Ætsning UNS på arm	0		
DT225;1° ætsning på overekstremitet	0		
DT225A;1° ætsning på skulder	0		
DT225B;1° ætsning på arm	0		
DT226;2° ætsning på overekstremitet	0		
DT226A;2° ætsning på skulder	0		
DT226B;2° ætsning på arm	0		
DT227;3° ætsning på overekstremitet		1	
DT227A;3° ætsning på skulder		1	
DT227B;3° ætsning på arm		1	
DT23;Forbrænding og ætsning på håndled og hånd	0		
DT230;Forbrænding UNS på håndled og hånd	0		
DT230A;Forbrænding UNS på håndled	0		

Supplement 2 Nielsen et al. Mortality after paediatric emergency calls for patients with and without pre-existing comorbidity (2023)

DT230B;Forbrænding UNS på hånd	0		
DT231;1° forbrænding på håndled og hånd	0		
DT231A;1° forbrænding på håndled	0		
DT231B;1° forbrænding på hånd	0		
DT232;2° forbrænding på håndled og hånd	0		
DT232A;2° forbrænding på håndled	0		
DT232B;2° forbrænding på hånd	0		
DT233;3° forbrænding på håndled og hånd		1	
DT233A;3° forbrænding på håndled		1	
DT233B;3° forbrænding på hånd		1	
DT234;Ætsning UNS på håndled og hånd	0		
DT234A;Ætsning UNS på håndled	0		
DT234B;Ætsning UNS på hånd	0		
DT235;1° ætsning på håndled og hånd	0		
DT235A;1° ætsning på håndled	0		
DT235B;1° ætsning på hånd	0		
DT236;2° ætsning på håndled og hånd	0		
DT236A;2° ætsning på håndled	0		
DT236B;2° ætsning på hånd	0		
DT237;3° ætsning på håndled og hånd		1	
DT237A;3° ætsning på håndled		1	
DT237B;3° ætsning på hånd		1	
DT24;Forbrænding og ætsning på hofte og ben	0		
DT240;Forbrænding UNS på underekstremitet	0		
DT240A;Forbrænding UNS på hofte	0		
DT240B;Forbrænding UNS på ben	0		
DT241;1° forbrænding på underekstremitet	0		
DT241A;1° forbrænding på hofte	0		
DT241B;1° forbrænding på ben	0		
DT242;2° forbrænding på underekstremitet	0		
DT242A;2° forbrænding på hofte	0		
DT242B;2° forbrænding på ben	0		

Supplement 2 Nielsen et al. Mortality after paediatric emergency calls for patients with and without pre-existing comorbidity (2023)

DT243;3° forbrænding på underekstremitet		1	
DT243A;3° forbrænding på hofte		1	
DT243B;3° forbrænding på ben		1	
DT244;Ætsning UNS på underekstremitet	0		
DT244A;Ætsning UNS på hofte	0		
DT244B;Ætsning UNS på ben	0		
DT245;1° ætsning på underekstremitet	0		
DT245A;1° ætsning på hofte	0		
DT245B;1° ætsning på ben	0		
DT246;2° ætsning på underekstremitet	0		
DT246A;2° ætsning på hofte	0		
DT246B;2° ætsning på ben	0		
DT247;3° ætsning på underekstremitet		1	
DT247A;3° ætsning på hofte		1	
DT247B;3° ætsning på ben		1	
DT25;Forbrænding og ætsning på ankel og fod	0		
DT250;Forbrænding UNS på ankel og fod	0		
DT250A;Forbrænding UNS på fod	0		
DT250B;Forbrænding UNS på ankel	0		
DT251;1° forbrænding på ankel og fod	0		
DT251A;1° forbrænding på fod	0		
DT251B;1° forbrænding på ankel	0		
DT252;2° forbrænding på ankel og fod	0		
DT252A;2° forbrænding på fod	0		
DT252B;2° forbrænding på ankel	0		
DT253;3° forbrænding på ankel og fod		1	
DT253A;3° forbrænding på fod		1	
DT253B;3° forbrænding på ankel		1	
DT254;Ætsning UNS på ankel og fod	0		
DT254A;Ætsning UNS på fod	0		
DT254B;Ætsning UNS på ankel	0		
DT255;1° ætsning på ankel og fod	0		

Supplement 2 Nielsen et al. Mortality after paediatric emergency calls for patients with and without pre-existing comorbidity (2023)

DT255A;1° ætsning på fod	0		
DT255B;1° ætsning på ankel	0		
DT256;2° ætsning på ankel og fod	0		
DT256A;2° ætsning på fod	0		
DT256B;2° ætsning på ankel	0		
DT257;3° ætsning på ankel og fod		1	
DT257A;3° ætsning på fod		1	
DT257B;3° ætsning på ankel		1	
DT26;Forbrænding og ætsning af øje og øjenregion	0		
DT260;Forbrænding af øjenlåg og øjenregion	0		
DT260A;Forbrænding af øjenlåg	0		
DT260B;Forbrænding af øjenregion	0		
DT261;Forbrænding af cornea og konjunktiva	0		
DT261A;Forbrænding af konjunktiva	0		
DT261B;Forbrænding af cornea	0		
DT262;Forbrænding af øje med ruptur eller destruktion af øjeæble	0		
DT263;Forbrænding af anden del af øje eller øjenregion	0		
DT264;Forbrænding af øje eller øjenregion UNS	0		
DT265;Ætsning af øjenlåg og øjenregion	0		
DT265A;Ætsning af øjenlåg	0		
DT265B;Ætsning af øjenregion	0		
DT266;Ætsning af cornea eller konjunktiva	0		
DT266A;Ætsning af konjunktiva	0		
DT266B;Ætsning af cornea	0		
DT267;Ætsning af øje med ruptur eller destruktion af øjeæble	0		
DT268;Ætsning af anden del af øje eller øjenregion	0		
DT269;Ætsning af øje eller øjenregion UNS	0		
DT27;Forbrænding og ætsning af luftveje		1	
DT270;Forbrænding i larynx eller luftrøret		1	
DT271;Forbrænding både i larynx eller luftrøret og lunger		1	
DT272;Forbrænding af anden del af luftvejene		1	
DT273;Forbrænding af luftvejene UNS		1	

Supplement 2 Nielsen et al. Mortality after paediatric emergency calls for patients with and without pre-existing comorbidity (2023)

DT274;Ætsning i larynx eller luftrøret		1	
DT275;Ætsning både i larynx eller luftrøret og lunger		1	
DT276;Ætsning af anden del af luftvejene		1	
DT277;Ætsning af luftvejene UNS		1	
DT28;Forbrænding og ætsning af andre indre organer		1	
DT280;Forbrænding i mundhulen eller svælget		1	
DT280A;Forbrænding i mundhulen		1	
DT280B;Forbrænding i svælget		1	
DT281;Forbrænding i spiserøret		1	
DT282;Forbrænding i anden del af fordøjelseskanalen		1	
DT283;Forbrænding i indre urin- eller kønsorganer		1	
DT284;Forbrænding i andet eller ikke specificeret indre organ		1	
DT284A;Forbrænding af indre organer UNS		1	
DT285;Ætsning i mundhulen eller svælget		1	
DT285A;Ætsning i mundhulen		1	
DT285B;Ætsning i svælget		1	
DT286;Ætsning i spiserøret		1	
DT287;Ætsning i anden del af fordøjelseskanalen		1	
DT288;Ætsning i indre urin- eller kønsorganer		1	
DT289;Ætsning i andet eller ikke specificeret indre organ		1	
DT29;Forbrænding og ætsning af flere legemsregioner	0		
DT290;Multiple forbrændinger UNS af flere legemsregioner	0		
DT291;Multiple 1° forbrændinger uden 2° og 3° forbrændinger	0		
DT292;Multiple 1° og 2° forbrændinger uden 3° forbrændinger	0		
DT293;Multiple forbrændinger med mindst en 3° forbrænding		1	
DT294;Multiple ætsninger UNS af flere legemsregioner	0		
DT295;Multiple 1° ætsninger uden 2° og 3° ætsninger	0		
DT296;Multiple 1° og 2° ætsninger uden 3° ætsninger	0		
DT297;Multiple ætsninger med mindst en 3° ætsning		1	
DT30;Forbrænding og ætsning uden angivelse af legemsregion	0		
DT300;Forbrænding UNS	0		
DT301;1° forbrænding UNS	0		

DT302;2° forbrænding UNS	0		
DT303;3° forbrænding UNS		1	
DT304;Ætsning UNS	0		
DT305;1° ætsning UNS	0		
DT306;2° ætsning UNS	0		
DT307;3° ætsning UNS		1	
DT31;Forbrænding i procent af legemsoverfladen	0		
DT310;Forbrænding af mindre end 10% af legemsoverfladen	0		
DT310A;Forbrænding, mindre end 5% af legemsoverfladen	0		
DT310B;Forbrænding, 5-9 % af legemsoverfladen	0		
DT311;Forbrænding af 10-19% af legemsoverfladen	0		
DT312;Forbrænding af 20-29% af legemsoverfladen		1	
DT313;Forbrænding af 30-39% af legemsoverfladen		1	
DT314;Forbrænding af 40-49% af legemsoverfladen		1	
DT315;Forbrænding af 50-59% af legemsoverfladen		1	
DT316;Forbrænding af 60-69% af legemsoverfladen		1	
DT317;Forbrænding af 70-79% af legemsoverfladen		1	
DT318;Forbrænding af 80-89% af legemsoverfladen		1	
DT319;Forbrænding af 90% eller mere af legemsoverfladen		1	
DT32;Ætsning i procent af legemsoverfladen	0		
DT320;Ætsning af mindre end 10% af legemsoverfladen	0		
DT320A;Ætsning, mindre end 5% af legemsoverfladen	0		
DT320B;Ætsning, 5-9 % af legemsoverfladen	0		
DT321;Ætsning af 10-19% af legemsoverfladen	0		
DT322;Ætsning af 20-29% af legemsoverfladen		1	
DT323;Ætsning af 30-39% af legemsoverfladen		1	
DT324;Ætsning af 40-49% af legemsoverfladen		1	
DT325;Ætsning af 50-59% af legemsoverfladen		1	
DT326;Ætsning af 60-69% af legemsoverfladen		1	
DT327;Ætsning af 70-79% af legemsoverfladen		1	
DT328;Ætsning af 80-89% af legemsoverfladen		1	
DT329;Ætsning af 90% eller mere af legemsoverfladen		1	

DT33;Overfladisk forfrysning	0		
DT330;Overfladisk forfrysning på hovedet	0		
DT331;Overfladisk forfrysning på halsen	0		
DT332;Overfladisk forfrysning på thorax	0		
DT333;Overfladisk forfrysning på abdomen, lænden eller bækkenet	0		
DT334;Overfladisk forfrysning på overekstremitet	0		
DT335;Overfladisk forfrysning på håndled eller hånd	0		
DT336;Overfladisk forfrysning på hofte eller lår	0		
DT337;Overfladisk forfrysning på knæ eller underben	0		
DT338;Overfladisk forfrysning på ankel eller fod	0		
DT339;Overfladisk forfrysning UNS	0		
DT34;Forfrysning med nekrose	0		
DT340;Forfrysning med nekrose på hovedet	0		
DT341;Forfrysning med nekrose på halsen	0		
DT342;Forfrysning med nekrose på thorax	0		
DT343;Forfrysning med nekrose på abdomen, lænden og bækkenet	0		
DT344;Forfrysning med nekrose på overekstremitet	0		
DT345;Forfrysning med nekrose på håndled eller hånd	0		
DT346;Forfrysning med nekrose på hofte eller lår	0		
DT347;Forfrysning med nekrose på knæ eller underben	0		
DT348;Forfrysning med nekrose på ankel eller fod	0		
DT349;Forfrysning med nekrose med anden eller ikke specificeret lokalisation	0		
DT35;Forfrysninger i flere legemsregioner og ikke specificeret forfrysning	0		
DT350;Multiple overfladiske forfrysninger i flere legemsregioner	0		
DT351;Multiple forfrysninger med nekroser i flere legemsregioner		1	
DT352;Forfrysninger på hovedet eller halsen UNS	0		
DT353;Forfrysninger på thorax, abdomen, lænden eller bækkenet UNS	0		
DT354;Forfrysninger på overekstremitet UNS	0		
DT355;Forfrysninger på underekstremitet UNS	0		
DT356;Multiple forfrysninger UNS	0		
DT357;Forfrysning UNS	0		
DT36;Forgiftning med antibiotika til systemisk brug	0		

DT360;Forgiftning med antibiotikum af kendt art	0		
DT369;Forgiftning med antibiotikum UNS	0		
DT37;Forgiftning med andre antibakterielle og antiparasitære midler	0		
DT370;Forgiftning med andet kendt antibakterielt eller antiparasitært middel	0		
DT379;Forgiftning med andet antibakterielt eller antiparasitært middel UNS	0		
DT38;Forgiftning med hormoner og syntetiske substitutter og antagonister IKA	0		
DT380;Forgiftning med hormon, syntetisk substitut eller antagonist af kendt art IKA	0		
DT383;Forgiftning med insulin eller andet antidiabetika	0		
DT383A;Insulinshock		1	
DT389;Forgiftning med hormon, syntetisk substitut eller antagonist UNS	0		
DT39;Forgiftning med svage smertestillende midler	0		
DT390;Forgiftning med svagt smertestillende middel af kendt art	0		
DT390A;Forgiftning med paracetamol (findes ikke i SKS)	0		
DT398;Forgiftning med andet svagt smertestillende middel	0		
DT398A;Forgiftning med paracetamol	0		
DT399;Forgiftning med svagt smertestillende middel UNS	0		
DT40;Forgiftninger med opioider, kokain og hallucinogener	0		
DT400;Forgiftning med opium	0		
DT401;Forgiftning med heroin	0		
DT402;Forgiftning med andet opioid	0		
DT402A;Forgiftning med kodein	0		
DT402B;Forgiftning med morfin	0		
DT403;Forgiftning med metadon	0		
DT404;Forgiftning med andet syntetisk opioid	0		
DT404A;Forgiftning med petidin	0		
DT405;Forgiftning med kokain	0		
DT406;Forgiftning med andet eller ikke specificeret opioid	0		
DT406W;Forgiftning med opioid UNS	0		
DT407;Forgiftning med cannabis	0		
DT408;Forgiftning med lysergsyrediethylamid (LSD)	0		
DT409;Forgiftning med andet eller ikke specificeret hallucinogen	0		
DT409A;Forgiftning med euforiserende svampe	0		

DT409B;Forgiftning med meskalin	0		
DT409C;Forgiftning med psilocin	0		
DT409D;Forgiftning med psilocybin	0		
DT409X;Forgiftning ved blandingsmisbrug med hallucinogener	0		
DT409Z;Forgiftning med hallucinogen UNS	0		
DT41;Forgiftninger med bedøvelsesmidler og terapeutiske gasarter	0		
DT410;Forgiftning med inhaleret bedøvelsesmiddel	0		
DT411;Forgiftning med intravenøst bedøvelsesmiddel	0		
DT411A;Forgiftning med thiobarbiturat	0		
DT412;Forgiftning med andet eller ikke specificeret middel til generel anæstesi	0		
DT413;Forgiftning med lokalt anæstetisk middel	0		
DT414;Forgiftning med andet eller ikke specificeret anæstetisk middel	0		
DT415;Forgiftning med terapeutisk gasart	0		
DT415A;Forgiftning med kuldioxid	0		
DT415B;Forgiftning med oxygen	0		
DT42;Forgiftninger med epilepsi-, sove- og antiparkinsonmidler	0		
DT420;Forgiftning med hydantoinderivat	0		
DT421;Forgiftning med carboxamidderivat	0		
DT422;Forgiftning med succinimiderivat	0		
DT423;Forgiftning med barbituratderivat	0		
DT424;Forgiftning med benzodiazepinderivat	0		
DT425;Forgiftning med blanding af antiepileptika, IKA	0		
DT426;Forgiftning andet antiepileptikum, sovemiddel eller beroligende middel	0		
DT427;Forgiftning med antiepileptikum, sovemiddel eller beroligende middel UNS	0		
DT428;Forgiftning med antiparkinsonmiddel eller andet centralt virkende muskeltonusnedsættend	0		
DT429;Forgiftning med epilepsi-, sove- eller antiparkinsonmiddel UNS	0		
DT43;Forgiftninger med andre psykotrope midler, IKA	0		
DT430;Forgiftning med cykliske antidepressive midler	0		
DT431;Forgiftning med MAO-hæmmer	0		
DT432;Forgiftning med andet antidepressivum	0		
DT433;Forgiftning med fenotiazin	0		
DT434;Forgiftning med butyrofenoner eller thioxantener	0		

Supplement 2 Nielsen et al. Mortality after paediatric emergency calls for patients with and without pre-existing comorbidity (2023)

DT435;Forgiftning med andet antipsykotisk eller neuroleptisk lægemiddel	0		
DT436;Forgiftning med psykostimulerende stoffer med misbrugspotentiale	0		
DT436A;Forgiftning med amfetamin	0		
DT436B;Forgiftning med ecstasy	0		
DT436C;Forgiftning med khat	0		
DT438;Forgiftning med andet psykotropt middel	0		
DT438A;Forgiftning med designer drug	0		
DT439;Forgiftning med psykotropt middel UNS	0		
DT44;Forgiftning med lægemidler med virkning på det autonome nervesystem	0		
DT440;Forgiftning med lægemiddel af kendt art med virkning på det autonome nervesystem	0		
DT449;Forgiftning med lægemiddel UNS med virkning på autonome nerver	0		
DT45;Forgiftning med lægemidler med systemisk og hæmatologisk virkning IKA	0		
DT450;Forgiftning med lægemiddel af kendt art med systemisk eller hæmatologisk virkning IKA	0		
DT450A;Forgiftning med lægemiddel af kendt art med virkning på koagulation IKA	0		
DT459;Forgiftning med lægemiddel UNS med systemisk eller hæmatologisk virkning IKA	0		
DT459A;Forgiftning med lægemiddel UNS med virkning på koagulation IKA	0		
DT46;Forgiftning med lægemidler med virkning primært på hjerte og kredsløb	0		
DT460;Forgiftning med lægemiddel af kendt art med virkning primært på hjerte og kredsløb	0		
DT469;Forgiftning med lægemiddel UNS med virkning primært på hjerte og kredsløb	0		
DT47;Forgiftning med lægemidler med virkning primært på fordøjelsessystemet	0		
DT470;Forgiftning med lægemiddel med virkning primært på fordøjelsessystemet	0		
DT479;Forgiftning med lægemiddel UNS med virkning primært på fordøjelsessystemet	0		
DT48;Forgiftning med lægemidler med virkning primært på glat muskulatur, bevægeapparat og år	0		
DT480;Forgiftning med lægemiddel af kendt art med virkning primært på glat muskulatur, bevæge	0		
DT489;Forgiftning med lægemiddel UNS med virkning primært på glat muskulatur, bevægeappara	0		
DT49;Forgiftning med lokalt virkende lægemidler til hud, slimhinder, øjne, ører, næse, hals og tær	0		
DT490;Forgiftning med lokalt virkende lægemiddel af kendt art til hud, slimhinder, øjne, ører, næs	0		
DT499;Forgiftning med lokalt virkende lægemiddel UNS til hud, slimhinder, øjne, ører, næse, hals	0		
DT50;Forgiftning med diuretika og andre og ikke specificerede lægemidler og biologiske stoffer	0		
DT500;Forgiftning med diuretikum eller andet lægemiddel eller biologisk stof af kendt art	0		
DT500A;Antabus-alkohol reaktion		1	
DT509;Forgiftning med lægemiddel eller biologisk stof UNS	0		

DT509A;Forgiftning med lægemiddel UNS	0		
DT509B;Forgiftning med biologisk stof UNS	0		
DT51;Forgiftning med alkohol	0		
DT510;Ethanolforgiftning	0		
DT511;Metanolforgiftning	0		
DT512;Isopropanolforgiftning	0		
DT513;Fuselolieforgiftning	0		
DT513A;Butylalkoholforgiftning	0		
DT513B;Propylalkoholforgiftning	0		
DT518;Forgiftning med anden type alkohol	0		
DT519;Alkoholforgiftning UNS	0		
DT52;Forgiftning med organiske opløsningsmidler	0		
DT520;Petroleumsforgiftning	0		
DT520A;Benzinforgiftning	0		
DT521;Benzenforgiftning	0		
DT522;Forgiftning med benzenhomolog	0		
DT522A;Toluenforgiftning	0		
DT522B;Xylenforgiftning	0		
DT523;Glycolforgiftning	0		
DT523A;Ethylenglycolforgiftning	0		
DT524;Ketonforgiftning	0		
DT528;Forgiftning med andet organisk opløsningsmiddel	0		
DT528A;Forgiftning med gamma-butyrolactone (GBL)	0		
DT529;Forgiftning med organisk opløsningsmiddel UNS	0		
DT53;Forgiftning med halogenderivater af alifatiske og aromatiske hydrocarboner	0		
DT530;Tetraklorkulstofforgiftning	0		
DT531;Kloroformforgiftning	0		
DT532;Triklorætylenforgiftning	0		
DT533;Tetraklorætylenforgiftning	0		
DT533A;Perklorætylenforgiftning	0		
DT533B;Tetraklorætenforgiftning	0		
DT534;Diklormetanforgiftning	0		

DT534A;Metylenkloridforgiftning	0			
DT535;Klorfluorkarbonforgiftning	0			
DT535A;Freonforgiftning	0			
DT536;Forgiftning med andet halogenderivat af alifatisk hydrocarbon	0			
DT537;Forgiftning med andet halogenderivat af aromatisk hydrocarbon	0			
DT539;Forgiftning med halogenderivat af alifatisk eller aromatisk hydrocarbon UNS	0			
DT54;Forgiftning med ætsende substanser	0			
DT540;Forgiftning med fenol eller fenolhomologer	0			
DT540A;Fenolforgiftning	0			
DT540B;Fenolhomologforgiftning	0			
DT541;Forgiftning med anden ætsende organisk substans	0			
DT542;Forgiftning med ætsende syre eller syrelignende substans	0			
DT542A;Saltsyreforgiftning	0			
DT542B;Svovlsyreforgiftning	0			
DT543;Forgiftning med ætsende base eller baselignende substans	0			
DT543A;Kaliumhydroxidforgiftning	0			
DT543B;Natriumhydroxidforgiftning	0			
DT549;Forgiftning med ætsende substans UNS	0			
DT55;Forgiftning med sæbe og rengøringsmidler	0			
DT559;Forgiftning med sæbe eller detergentium	0			
DT559A;Detergentiumforgiftning	0			
DT559B;Sæbeforgiftning	0			
DT56;Forgiftning med metaller	0			
DT560;Blyforgiftning	0			
DT561;Kviksølvforgiftning	0			
DT562;Kromforgiftning	0			
DT563;Kadmiumforgiftning	0			
DT564;Kobberforgiftning	0			
DT565;Zinkforgiftning	0			
DT566;Tinforgiftning	0			
DT567;Berylliumforgiftning	0			
DT568;Forgiftning med andet metal	0			

DT568A;Talliumforgiftning	0		
DT569;Forgiftning med metal UNS	0		
DT57;Forgiftning med andre uorganiske substanser	0		
DT570;Arsenikforgiftning	0		
DT571;Fosforforgiftning	0		
DT572;Manganforgiftning	0		
DT573;Blåsyreforgiftning	0		
DT578;Forgiftning med anden uorganisk substans	0		
DT579;Forgiftning med uorganisk substans UNS	0		
DT58;Forgiftning med kulilte	0		
DT589;Kulilteforgiftning	0		
DT59;Forgiftning med andre gasarter, røg og dampe	0		
DT590;Nitrogenoxidforgiftning	0		
DT591;Svovldioxidforgiftning	0		
DT592;Formaldehydforgiftning	0		
DT593;Tåregasforgiftning	0		
DT594;Klorgasforgiftning	0		
DT595;Fluorgas- eller hydrogenfluorgasforgiftning	0		
DT596;Hydrogensulfidforgiftning	0		
DT597;Kultveilteforgiftning	0		
DT598;Forgiftning med anden gasart, røg eller damp	0		
DT599;Forgiftning med gasart, røg eller damp UNS	0		
DT599B;Røgforgiftning UNS	0		
DT60;Forgiftning med pesticider	0		
DT600;Forgiftning med organisk fosfat- eller karbamatholdigt insekticid	0		
DT601;Forgiftning med halogeneret insekticid	0		
DT602;Forgiftning med andet insekticid	0		
DT602A;Insekticidforgiftning UNS	0		
DT603;Forgiftning med herbicid eller fungicid	0		
DT604;Rodenticidforgiftning	0		
DT608;Forgiftning med andet pesticid	0		
DT609;Pesticidforgiftning UNS	0		

Supplement 2 Nielsen et al. Mortality after paediatric emergency calls for patients with and without pre-existing comorbidity (2023)

DT61;Forgiftning med giftige bestanddele i madvarer fra havet	0		
DT610;Ciguaterafiskeforgiftning	0		
DT611;Makrelforgiftning	0		
DT612;Forgiftning med anden fisk eller skaldyr	0		
DT612A;Fiskeforgiftning UNS	0		
DT612B;Skaldyrforgiftning	0		
DT618;Forgiftning med anden madvare fra havet	0		
DT619;Forgiftning med fisk, skaldyr eller madvare fra havet UNS	0		
DT62;Forgiftning med andre giftige bestanddele i madvarer	0		
DT620;Svampeforgiftning	0		
DT621;Bærforgiftning	0		
DT622;Forgiftning med anden indtaget plantedel	0		
DT622A;Planteforgiftning UNS	0		
DT628;Forgiftning med anden indtaget specificeret bestanddel i maden	0		
DT629;Madforgiftning UNS	0		
DT63;Skadelig virkning ved kontakt med giftige dyr	0		
DT630;Giftigt slangebid	0		
DT631;Giftigt krybdyrsbid	0		
DT632;Giftigt skorpionbid	0		
DT633;Giftigt edderkoppebid	0		
DT634;Skadelig virkning ved kontakt med andet giftigt leddyr	0		
DT634A;Bistik	0		
DT634B;Hvepsestik	0		
DT634C;Giftigt insektbid eller insektstik UNS	0		
DT634E;Kraftig lokalreaktion efter insektbid eller insektstik	0		
DT634F;Anafylaktisk shock efter insektbid eller insektstik		1	
DT634G;Systemisk allergisk reaktion efter insektbid eller insektstik		1	
DT635;Skadelig virkning forårsaget af kontakt med fisk	0		
DT636;Skadelig virkning forårsaget af kontakt med andet havdyr	0		
DT636A;Skadelig virkning forårsaget af kontakt med brandmand	0		
DT636B;Skadelig virkning forårsaget af kontakt med havdyr UNS	0		
DT636C;Skadelig virkning forårsaget af kontakt med musling	0		

Supplement 2 Nielsen et al. Mortality after paediatric emergency calls for patients with and without pre-existing comorbidity (2023)

DT636D;Skadelig virkning forårsaget af kontakt med gople	0			
DT638;Skadelig virkning forårsaget af kontakt med andet giftigt dyr	0			
DT639;Skadelig virkning forårsaget af kontakt med giftigt dyr UNS	0			
DT64;Skadelig virkning af aflatoksin og andre svampegifte i madvarer	0			
DT641;Alimentær aflatoksinforgiftning	0			
DT641A;Intoksikation med aflatoksin efter inhalation	0			
DT649;Skadelig virkning af andet mykotoksin i madvarer	0			
DT65;Forgiftning med andre og ikke specificerede substanser	0			
DT650;Cyanidforgiftning	0			
DT651;Strykninforgiftning	0			
DT652;Forgiftning med tobak eller nikotin	0			
DT652A;Nikotinforgiftning	0			
DT653;Forgiftning med nitro- eller aminoderivater af benzen eller benzenhomologer	0			
DT653C;Anilinforgiftning	0			
DT653D;Nitrobenzenforgiftning	0			
DT654;Kulstofdisulfidforgiftning	0			
DT655;Nitroglycerinforgiftning	0			
DT656;Forgiftning med maling eller farver IKA	0			
DT658;Forgiftning med anden substans	0			
DT658A;Forgiftning med ricintoksin	0			
DT658C;Styrenforgiftning	0			
DT659;Forgiftning UNS	0			
DT66;Skade forårsaget af stråling	0			
DT669;Stråleskade UNS	0			
DT669A;Fibrose eller ardannelse i kar, binde- eller støttevæv som følge af stråling	0			
DT669B;Nekrose i kar, binde- eller støttevæv som følge af stråling	0			
DT67;Skade forårsaget af varme og lys	0			
DT670;Hedeslag og solstik	0			
DT670A;Hedeslag	0			
DT670B;Solstik	0			
DT671;Besvimelse forårsaget af varme	0			
DT672;Krampe forårsaget af varme	0			

DT673;Varmeudmattelse forårsaget af væsketab uden salttab	0		
DT674;Varmeudmattelse forårsaget af salttab	0		
DT675;Udmattelse ved varme UNS	0		
DT676;Forbigående udmattelse forårsaget af varme	0		
DT677;Ødem forårsaget af varme	0		
DT678;Anden påvirkning af varme og lys	0		
DT679;Påvirkning af varme og lys UNS	0		
DT68;Påvirkning af nedsat temperatur	0		
DT689;Accidentel hypotermi	0		
DT69;Andre virkninger af nedsat temperatur	0		
DT690;Nedkøling af hånd eller fod	0		
DT691;Pernio	0		
DT698;Anden virkning af nedsat temperatur	0		
DT699;Virkning af nedsat temperatur UNS	0		
DT70;Skadelig virkning af lufttryk og vandtryk	0		
DT700;Tryklæsion af øre UNS	0		
DT7001;Tryklæsion i ydre øre	0		
DT7002;Tryklæsion i mellemøre	0		
DT70020;Tryklæsion i mellemøre TEED 0	0		
DT70021;Tryklæsion i mellemøre TEED 1	0		
DT70022;Tryklæsion i mellemøre TEED 2	0		
DT70023;Tryklæsion i mellemøre TEED 3	0		
DT70024;Tryklæsion i mellemøre TEED 4	0		
DT70025;Tryklæsion i mellemøre TEED 5	0		
DT7003;Tryklæsion i indre øre	0		
DT701;Tryklæsion i bihule	0		
DT702;Anden eller ikke specificeret virkning af ophold i stor højde	0		
DT703;Dykkersyge	0		
DT7031;Dykkersyge, udvikling ikke specificeret	0		
DT7031A;Langsomt progredierende dykkersyge	0		
DT7031B;Hurtigt progredierende dykkersyge	0		
DT7031C;Statisk dykkersyge	0		

DT7031D;Dykkersyge med spontan bedring	0		
DT7031E;Dykkersyge med tilbagefald før behandling	0		
DT7031F;Dykkersyge med tilbagefald under behandling	0		
DT7031G;Dykkersyge med tilbagefald efter behandling	0		
DT7032;Dykkersyge med specificerede symtomer	0		
DT7032A;Dykkersyge med artikulære smerter	0		
DT7032B;Dykkersyge med limb-girdle-smerter	0		
DT7032C;Dykkersyge med neurologiske symptomer	0		
DT7032D;Dykkersyge med audiovestibulære symptomer	0		
DT7032E;Dykkersyge med pulmonale symptomer	0		
DT7032F;Dykkersyge med kutane symptomer	0		
DT7032G;Dykkersyge med med lymfatiske symptomer	0		
DT7032H;Dykkersyge med konstitutionelle symptomer	0		
DT7032I;Dykkersyge med multiorgansymptomer	0		
DT7033;Dykkersyge med oplysning om forløb	0		
DT7033A;Dykkersyge med symptomdebut inden 30 minutter	0		
DT7033B;Dykkersyge med symptomdebut efter ½ til 6 timer	0		
DT7033C;Dykkersyge med symptomdebut efter mere end 6 timer	0		
DT7033D;Dykkersyge med tegn på pulmonalt barotraume	0		
DT7034;Dykkersyge med beskrevet behandlingsresultat	0		
DT7034A;Dykkersyge med symptomer svundet på behandling	0		
DT7034B;Dykkersyge i regression på behandling	0		
DT7034C;Dykkersyge, uændret trods behandling	0		
DT7035;Dykkersyge uden angivelse af undersøgelsesresultater	0		
DT7039;Følger efter dykkersyge		1	
DT7039A;Dykkersyge med subjektive restsymptomer i regression		1	
DT7039B;Dykkersyge med objektive restsymptomer i regression		1	
DT7039C;Følger efter dykkersyge med stationære restsymptomer		1	
DT704;Påvirkning fra højt vandtryk	0		
DT708;Anden virkning fra lufttryk eller vandtryk	0		
DT708A;Eksplosionslæsion	0		
DT709;Virkning af lufttryk eller vandtryk UNS	0		

Supplement 2 Nielsen et al. Mortality after paediatric emergency calls for patients with and without pre-existing comorbidity (2023)

DT71;Kvælning		1	
DT719;Asfyksi		1	
DT719A;Asfyksi forårsaget af strangulation ved hængning		1	
DT719B;Asfyksi forårsaget af strangulation ved omsnøring		1	
DT719C;Asfyksi som følge af mekanisk kvælning		1	
DT73;Skadevirkning af andre mangelsituationer	0		
DT730;Sult	0		
DT731;Tørst	0		
DT732;Udmattelse efter ydre påvirkning	0		
DT733;Udmattelse efter fysisk overanstrengelse	0		
DT738;Anden skadevirkning af mangelsituation	0		
DT739;Skadevirkning af mangelsituation UNS	0		
DT74;Mishandlingssyndromer		1	
DT740;Omsorgssvigt		1	
DT740A;Misrøgt af barn		1	
DT741;Fysisk mishandling		1	
DT741A;Battered child-syndrom		1	
DT741B;Battered spouse-syndrom		1	
DT742;Seksuelt overgreb		1	
DT743;Psykisk mishandling		1	
DT748;Andet mishandlingssyndrom		1	
DT748A;Münchhausen by proxy		1	
DT749;Mishandlingssyndrom UNS		1	
DT75;Andre ydre påvirkninger	0		
DT750;Skadevirkning af lynnedslag	0		
DT751;Drukning eller nær-drukning	0		
DT751A;Asfyksi som følge af nær-drukning	0		
DT752;Vibrationsskade	0		
DT752A;Traumatisk vasospastisk syndrom	0		
DT753;Transportsyge	0		
DT754;Skadevirkning af elektrisk strøm	0		
DT754A;Shock ved elektisk stød	0		

Supplement 2 Nielsen et al. Mortality after paediatric emergency calls for patients with and without pre-existing comorbidity (2023)

DT758;Anden skade af ydre påvirkning	0		
DT758A;Gravitationsskade	0		
DT758B;Vægtløshedsskade	0		
DT759;Skade ved ydre påvirkning UNS	0		
DT78;Visse bivirkninger IKA	0		
DT780;Anafylaktisk shock forårsaget af fødevareallergi eller -intolerans		1	
DT780A;Anafylaktisk shock forårsaget af fødevareallergi		1	
DT780B;Anafylaktisk shock forårsaget af fødevarentolerans		1	
DT781;Anden fødevarereaktion IKA	0		
DT781A;Ikke-allergisk fødevarereaktion UNS	0		
DT781B;Allergisk fødevarereaktion UNS	0		
DT781C;Fødevareintolerance UNS	0		
DT782;Anafylaktisk shock UNS		1	
DT782A;Anstrengelsesudløst anafylaktisk shock		1	
DT783;Quinkes ødem	0		
DT784;Allergi UNS	0		
DT788;Anden bivirkning IKA	0		
DT788A;Anstrengelsesudløst anafylaksi	0		
DT789;Bivirkning UNS	0		
DT79;Visse tidlige komplikationer til skade ved ydre påvirkning IKA	0		
DT790;Traumatisk luftemboli	0		
DT791;Traumatisk fedtemboli	0		
DT792;Sekundær eller recidiverende traumatisk blødning	0		
DT792A;Recidiverende traumatisk blødning	0		
DT792B;Sekundær traumatisk blødning	0		
DT793;Posttraumatisk sårinfektion IKA	0		
DT794;Traumatisk shock	0		
DT795;Posttraumatisk anuri	0		
DT795B;Kompressionssyndrom	0		
DT796;Traumatisk muskeliskæmi	0		
DT796A;Volkmanns kontraktur	0		
DT796B;Posttraumatisk dystrofi	0		

Supplement 2 Nielsen et al. Mortality after paediatric emergency calls for patients with and without pre-existing comorbidity (2023)

DT796C;Paralysis ischaemica Volkmann	0		
DT796D;Traumatisk rhabdomyolyse	0		
DT796E;Traumatisk kompartmentssyndrom	0		
DT797;Traumatisk subkutant emfysem	0		
DT798;Anden tidlig komplikation til skade ved ydre påvirkning	0		
DT799;Posttraumatisk komplikation UNS	0		
DT80;Komplikationer efter infusion, transfusion og injektion		1	
DT800;Luftemboli efter infusion, transfusion eller injektion		1	
DT801;Karkomplikation efter infusion, transfusion eller injektion		1	
DT802;Infektion efter infusion, transfusion eller injektion		1	
DT802A;Infektion efter infusion, transfusion eller injektion UNS		1	
DT802D;Sepsis efter infusion, transfusion eller injektion		1	
DT802D1;CVK-relateret sepsis		1	
DT802D2;CVK-relateret bakteriæmi (CLABSI)		1	
DT802G;CVK-relateret infektion		1	
DT803;AB0-uforligelighedsreaktion ved transfusion		1	
DT804;Rhesusuforligelighedsreaktion ved transfusion		1	
DT805;Anafylaktisk shock ved indgift af serum		1	
DT806;Anden reaktion på serum		1	
DT808;Anden komplikation efter infusion, transfusion eller injektion		1	
DT808A;Hæmoglobinæmi efter transfusion		1	
DT808B;Hæmosiderose efter transfusion		1	
DT808C;Komplikation efter spinal eller epidural anæstesi UNS		1	
DT808E;Ekstravasation af kemoterapeutikum		1	
DT809;Komplikation efter infusion, transfusion eller injektion UNS		1	
DT81;Komplikationer til indgreb IKA		1	
DT810;Postoperativ blødning eller hæmatom IKA		1	
DT810A;Hæmatom i cicatrice		1	
DT810B;Overfladisk hæmatom eller blødning i operationssår		1	
DT810C;Dybt hæmatom eller blødning i operationssår		1	
DT810E;Postoperativ intraperitonal blødning eller hæmatom		1	
DT810F;Postoperativ supravaginal blødning eller hæmatom		1	

DT810G;Postoperativ blødning UNS		1		
DT810H;Postoperativt hæmatom UNS		1		
DT810I;Blæretamponade		1		
DT810J;Blødning som følge af endoskopi		1		
DT810J1;Blødning som følge af koloskopi		1		
DT810K;Blødning fra biopsisted		1		
DT811;Shock ved indgreb IKA		1		
DT811A;Postoperativ kollaps UNS		1		
DT811B;Hypovolæmisk kirurgisk shock		1		
DT812;Utilsigtet peroperativ punktur eller læsion IKA		1		
DT812A;Utilsigtet peroperativ læsion IKA		1		
DT812B;Utilsigtet peroperativ punktur IKA		1		
DT812G;Utilsigtet peroperativ punktur eller læsion af gastrointestinalkanalen		1		
DT812G1;Utilsigtet peroperativ punktur eller læsion af tarmen ved koloskopi		1		
DT812H;Utilsigtet peroperativ punktur eller læsion af kvindeligt kønsorgan		1		
DT812H1;Utilsigtet peroperativ perforation af uterus ved resektion		1		
DT812K;Utilsigtet peroperativ punktur eller læsion af kar eller lymfesystem		1		
DT812N;Utilsigtet peroperativ læsion af nerve		1		
DT812O;Utilsigtet peroperativ læsion af led eller bløddele		1		
DT812T;Utilsigtet pneumothorax ved punktur		1		
DT812U;Utilsigtet peroperativ punktur eller læsion af urinveje		1		
DT812UA;Utilsigtet peroperativ punktur eller læsion af nyre		1		
DT812UB;Utilsigtet peroperativ punktur eller læsion af nyrebækken		1		
DT812UC;Utilsigtet peroperativ punktur eller læsion af urinleder		1		
DT812UD;Utilsigtet peroperativ punktur eller læsion af urinblære		1		
DT812UE;Utilsigtet peroperativ punktur eller læsion af urinrør		1		
DT812V;Utilsigtet peroperativ læsion af organ med samtidig intervention		1		
DT812W;Utilsigtet peroperativ læsion af organ uden samtidig intervention		1		
DT812X;Utilsigtet peroperativ læsion af retina		1		
DT813;Postoperativ sårruptur IKA		1		
DT813A;Anastomoselækage		1		
DT813A1;Anastomoselækage, anastomose bevaret		1		

DT813A2;Anastomoselækage, anastomose nedbrudt		1	
DT813C;Overfladisk bristning eller nekrose af operationssår		1	
DT813D;Dyb bristning af operationssår		1	
DT813G;Postoperativ komplikation ved anastomose eller stoma i gastrointestinalkanalen		1	
DT813O;Frakturskred eller pseudoartrose efter reposition med eller uden fiksation		1	
DT813O1;Frakturskred efter reposition uden fiksation		1	
DT813O2;Frakturskred efter reposition med fiksation		1	
DT813S;Sutur sprunget op		1	
DT813T;Postoperativ komplikation ved operation på thorax eller lunger		1	
DT813U;Postoperativ komplikation ved anastomose eller stoma i urinvejene		1	
DT814;Infektion efter indgreb IKA		1	
DT814A;Absces i operationscicatrice		1	
DT814B;Postoperativ intraabdominal absces		1	
DT814C;Postoperativ subfrenisk absces		1	
DT814D;Postoperativ sepsis		1	
DT814E;Postoperativt sårgranulom		1	
DT814F;Postoperativ sårinfektion		1	
DT814G;Postoperativ overfladisk sårinfektion		1	
DT814H;Postoperativ dyb sårinfektion		1	
DT814I;Postoperativ intraabdominal infektion UNS		1	
DT814J;Postoperativ retroperitoneal infektion		1	
DT814P;Postoperativ pneumoni		1	
DT814P1;Postoperativ aspirationspneumoni		1	
DT814U;Postoperativ urinvejsinfektion		1	
DT814X;Anden postoperativ infektion		1	
DT815;Fremmedlegeme utilsigtet efterladt i operationsfelt		1	
DT816;Reaktion mod fremmedlegeme utilsigtet efterladt i operationsfelt		1	
DT816A;Peritonitis (aseptisk, kemisk) forårsaget af fremmedlegeme utilsigtet efterladt i operationsfelt		1	
DT817;Karkomplikation efter indgreb IKA		1	
DT817A;Postoperativ luftemboli IKA		1	
DT817B;Tromboembolisk komplikation IKA		1	
DT817B1;Postoperativ arteriel emboli eller trombose		1	

DT817C;Postoperativ dyb venetrombose		1	
DT817D;Postoperativ lungeemboli		1	
DT817E;Postoperativ lymphocele		1	
DT817E1;Lymphocele efter nyretransplantation		1	
DT817E2;Lymphocele efter karoperation		1	
DT817E3;Lymphocele efter eksstirpation af lymfeknude		1	
DT817K;Trombose, emboli eller nekrose efter karoperation		1	
DT817X;Anden kirurgisk komplikation til indgreb IKA		1	
DT817Y;Anden medicinsk komplikation til indgreb IKA		1	
DT817Y1;Postoperativ apoplexia cerebri		1	
DT817Y2;Postoperativt akut myokardieinfarkt		1	
DT817Y3;Postoperativ hjerteinsufficiens		1	
DT817Y4;Postoperativ nyreinsufficiens		1	
DT818;Anden komplikation til indgreb IKA		1	
DT818A;Postoperativt subkutant emfysem		1	
DT818B;Fistel i cicatrice		1	
DT818C;Permanent postoperativ fistel		1	
DT818D;Siven af fostervand efter amniocentese		1	
DT818E;Postoperativ temperaturforhøjelse		1	
DT818F;Peroperativ blødning UNS		1	
DT818G;Postoperative sammenvoksninger i næsehulen		1	
DT818H;Tabt linsemateriale		1	
DT818J;Postoperative kroniske smerter		1	
DT818J1;Postoperative kroniske smerter efter gynækologisk operation		1	
DT819;Komplikation til indgreb UNS		1	
DT82;Komplikationer til proteser, implantater og transplantater i hjerte og kar		1	
DT820;Mekanisk komplikation til hjerteklapprotese		1	
DT820A;Insufficiens af hjerteklapprotese		1	
DT820B;Luksation af hjerteklapprotese		1	
DT820C;Stenose i hjerteklapprotese		1	
DT821;Mekanisk komplikation til pacemaker		1	
DT822;Mekanisk komplikation til koronar bypass		1	

Supplement 2 Nielsen et al. Mortality after paediatric emergency calls for patients with and without pre-existing comorbidity (2023)

DT822A;Stenose i koronar bypass		1		
DT823;Mekanisk komplikation til anden karprotese eller -transplantat		1		
DT823A;Mekanisk komplikation til aortaprotese eller -transplantat		1		
DT823B;Mekanisk komplikation til carotisprotese eller -transplantat		1		
DT823C;Mekanisk komplikation til arteria femoralis-protese eller -transplantat		1		
DT823D;Akut koronar stent-trombose		1		
DT823E;Koronar stent-restenose		1		
DT824;Kateterkomplikation ved hæmodialyse		1		
DT825;Mekanisk komplikation til andet implantat eller transplantat i hjerte eller kar		1		
DT825A;Malfunktion af a-v fistel		1		
DT826;Infektion eller inflammation omkring hjerteklapprotese		1		
DT826A;Infektion omkring hjerteklapprotese		1		
DT827;Infektion eller inflammation omkring andet implantat eller transplantat i hjerte eller kar		1		
DT827A;Infektion omkring hjerte-kar-protese UNS		1		
DT827B;Infektion omkring hjerte-kar-transplantat UNS		1		
DT827I;Infektion ved implanteret kardioverter-defibrillator		1		
DT827P;Infektion ved implanteret pacemaker		1		
DT828;Anden komplikation til protese, implantat eller transplantat i hjerte eller kar		1		
DT828A;Trombose i a-v fistel		1		
DT828P;Funktionsproblemer ved pacemakerbehandling		1		
DT828PA;Manglende output (ingen pacespike)		1		
DT828PB;Manglende capture (exit blok) ved pacemaker		1		
DT828PC;Manglende capture i atriet ved pacemaker		1		
DT828PD;Manglende capture i ventriklen ved pacemaker		1		
DT828PE;Mangelfuld sensing ved pacemaker		1		
DT828PF;Oversensing ved pacemaker		1		
DT828PG;Oversensing i atriet ved pacemaker		1		
DT828PH;Oversensing i ventriklen ved pacemaker		1		
DT828PJ;Undersensing ved pacemaker		1		
DT828PK;Undersensing i atriet ved pacemaker		1		
DT828PL;Undersensing i ventriklen ved pacemaker		1		
DT828PM;Pacemakermedieret takykardi		1		

Supplement 2 Nielsen et al. Mortality after paediatric emergency calls for patients with and without pre-existing comorbidity (2023)

DT828PN;Uhensigtsmæssigt DC-stød ved pacemaker		1	
DT829;Komplikation til protese, implantat eller transplantat i hjerte eller kar UNS		1	
DT83;Komplikationer til urogenitale proteser, implantater og transplanter		1	
DT830;Mekanisk komplikation til urinvejskateterisering		1	
DT831;Mekanisk komplikation til andet utensilie eller implantat i urinvejene		1	
DT832;Mekanisk komplikation til transplantat i urinvejene		1	
DT833;Mekanisk komplikation til intrauterint utensilie		1	
DT833A;Uterusperforation forårsaget af intrauterint kontraceptivt hjælpemiddel		1	
DT833A1;Uterusperforation, IUD beliggende intraperitonealt		1	
DT834;Mekanisk komplikation til anden protese, implantat eller transplantat i kønsorganerne		1	
DT835;Infektion eller inflammation omkring protese, implantat eller transplantat i urinvejene		1	
DT835A;Infektion ved implantat i urinvejene		1	
DT835B;Infektion ved protese i urinvejene		1	
DT835C;Infektion ved transplantat i urinvejene		1	
DT836;Infektion eller inflammation omkring protese, implantat eller transplantat i kønsorganerne		1	
DT836A;Infektion ved implantat i kønsorganerne		1	
DT836B;Infektion ved protese i kønsorganerne		1	
DT836C;Infektion ved transplantat i kønsorganerne		1	
DT838;Anden komplikation til urogenital protese, implantat eller transplantat		1	
DT838A;Banalt mekanisk kateterproblem		1	
DT838B;Nedbrydning af vaginalt implantat		1	
DT839;Komplikation til urogenital protese, implantat eller transplantat UNS		1	
DT84;Komplikationer til interne ortopædiske proteser, implantater og transplantater		1	
DT840;Mekanisk komplikation til ledprotese		1	
DT840A;Mekanisk komplikation til hofteledsprotese		1	
DT840B;Mekanisk komplikation til knæledsprotese		1	
DT841;Mekanisk komplikation til intern fiksation af ekstremitetsknogle		1	
DT842;Mekanisk komplikation til intern fiksation af anden knogle		1	
DT843;Mekanisk komplikation til anden knogleprotese, -implantat eller -transplantat		1	
DT844;Mekanisk komplikation til anden ortopædisk protese, implantat eller transplantat		1	
DT845;Infektion eller inflammation omkring ledprotese		1	
DT845A;Infektion omkring ledprotese		1	

DT846;Infektion eller inflammation omkring internt fiksationsmateriale		1	
DT846A;Infektion omkring internt fiksationsmateriale		1	
DT847;Infektion eller inflammation omkring anden ortopædisk protese, implantat eller transplantat		1	
DT848;Anden komplikation til intern ortopædisk protese, implantat eller transplantat		1	
DT849;Komplikation til intern ortopædisk protese, implantat eller transplantat UNS		1	
DT85;Komplikationer til andre interne proteser, implantater og transplantater		1	
DT850;Mekanisk komplikation til ventrikulær intrakraniel shunt		1	
DT851;Mekanisk komplikation til elektronisk nervestimulator		1	
DT851A;Mekanisk komplikation til intradural stimulator		1	
DT852;Mekanisk komplikation til intraokulær kunstig linse		1	
DT852A;Luksation af intraokulær kunstig linse		1	
DT853;Mekanisk komplikation til anden protese, implantat eller transplantat i øje		1	
DT853A;Mekanisk komplikation til øjenprotese		1	
DT853B;Mekanisk komplikation til andet implantat i øje		1	
DT853C;Mekanisk komplikation til corneatransplantat		1	
DT854;Mekanisk komplikation til mammaprotese		1	
DT855;Mekanisk komplikation til gastrointestinal protese, implantat eller transplantat		1	
DT855A;Mekanisk komplikation til gastrointestinalt implantat		1	
DT855A1;Malfunktion af peritonealdialysekateter		1	
DT855B;Mekanisk komplikation til gastrointestinal protese		1	
DT855C;Mekanisk komplikation til gastrointestinalt transplantat		1	
DT856;Mekanisk komplikation til anden intern protese, implantat eller transplantat		1	
DT856A;Mekanisk komplikation til epiduralt infusionkateter		1	
DT856B;Mekanisk komplikation til subduralt infusionkateter		1	
DT856C;Mekanisk komplikation til intraperitonealt dialysekateter		1	
DT856D;Suturgranulom		1	
DT857;Infektion eller inflammation omkring anden intern protese, implantat eller transplantat		1	
DT857A;Komplikation til intraduralt implantat		1	
DT858;Anden komplikation til intern protese, implantat eller transplantat		1	
DT858A;Excessiv periprostetisk fibrose		1	
DT858B;Lapnekrose		1	
DT859;Komplikation til intern protese, implantat eller transplantat UNS		1	

Supplement 2 Nielsen et al. Mortality after paediatric emergency calls for patients with and without pre-existing comorbidity (2023)

DT86;Svigt og afstødning af transplanterede organer og væv		1	
DT860;Afstødning af knoglemarvstransplantat		1	
DT860A;Graft-versus-host reaktion		1	
DT861;Svigt eller afstødning af transplanteret nyre		1	
DT861A;Svigt af nyretransplantat		1	
DT861B;Afstødning af nyretransplantat		1	
DT862;Svigt eller afstødning af transplanteret hjerte		1	
DT862A;Svigt af hjertetransplantat		1	
DT862B;Afstødning af hjertetransplantat		1	
DT863;Svigt eller afstødning af hjerte-lunge-transplantat		1	
DT863A;Svigt af hjerte-lunge-transplantat		1	
DT863B;Afstødning af hjerte-lunge-transplantat		1	
DT864;Svigt eller afstødning af transplanteret lever		1	
DT864A;Svigt af levertransplantat		1	
DT864B;Afstødning af levertransplantat		1	
DT868;Svigt eller afstødning af andet transplanteret organ eller væv		1	
DT868A;Svigt af transplanteret hud		1	
DT868B;Svigt af transplanteret tyndtarm		1	
DT868C;Svigt af transplanteret knogle		1	
DT868D;Svigt af transplanteret pancreas		1	
DT868E;Svigt af transplanteret lunge		1	
DT868F;Afstødning af transplanteret hud		1	
DT868G;Afstødning af transplanteret tyndtarm		1	
DT868H;Afstødning af transplanteret knogle		1	
DT868I;Afstødning af transplanteret pancreas		1	
DT868J;Afstødning af transplanteret lunge		1	
DT869;Svigt eller afstødning af transplanteret organ eller væv UNS		1	
DT87;Komplikationer til påsætning og amputation		1	
DT870;Komplikation til påsætning af (del af) arm		1	
DT871;Komplikation til påsætning af (del af) ben		1	
DT872;Komplikation til påsætning af anden legemsdel		1	
DT873;Neurom i amputationsstump		1	

DT874;Infektion i amputationsstump		1	
DT875;Nekrose i amputationsstump		1	
DT876;Anden eller ikke specificeret komplikation i amputationstump		1	
DT876A;Komplikation efter amputation UNS		1	
DT876B;Kontraktur efter amputation		1	
DT876C;Residualsmerter efter amputation		1	
DT876D;Hæmatom i amputationssår		1	
DT876E;Ødem efter amputation		1	
DT876F;Stumpsmerter		1	
DT88;Andre komplikationer til kirurgisk og medicinsk behandling IKA		1	
DT880;Infektion efter vaccination		1	
DT880A;Sepsis efter vaccination		1	
DT881;Anden komplikation til vaccination eller immunisering		1	
DT881A;Eksantem efter vaccination		1	
DT881B;Vaccinationsgranulom		1	
DT881C;Komplikation til vaccination eller immunisering UNS		1	
DT882;Shock ved anæstesi		1	
DT883;Malign hypertermi ved anæstesi		1	
DT884;Mislykket eller vanskelig intubation		1	
DT884A;Tandskader efter intubation		1	
DT884B;Skade på kæbe eller kæbeled efter intubation		1	
DT884C;Fejlplaceret tube under anæstesi		1	
DT885;Anden komplikation til anæstesi		1	
DT885A;Hypotermi efter anæstesi		1	
DT885E;Komplikation til epidural analgesi		1	
DT885G;Komplikation til generel anæstesi		1	
DT885S;Komplikation til spinal analgesi		1	
DT886;Anafylaktisk shock ved korrekt administration af lægemiddel		1	
DT887;Lægemiddelbivirkning UNS		1	
DT887A;Lægemiddelallergi UNS		1	
DT887B;Lægemiddelhypersensitivitet UNS		1	
DT887C;Lægemiddelidiosynkrasi UNS		1	

DT887D;Lægemiddelreaktion UNS		1	
DT888;Anden komplikation til kirurgisk eller medicinsk behandling IKA		1	
DT888K;Dysreguleret AK-behandling		1	
DT888L;Post-polypektomi syndrom		1	
DT888N;Neutropen feber ved cytostatisk behandling		1	
DT888P;Gips for stram	0		
DT888Q;Gips for løs	0		
DT888R;Problem med forbinding eller gips UNS	0		
DT888S;Peroperativ absorption af skyllevæske		1	
DT888S1;Peroperativ absorption af NaCl-skyllevæske		1	
DT888S2;Peroperativ absorption af natriumcitratskyllevæske		1	
DT888S3;Peroperativ absorption af magnesiumcitratskyllevæske		1	
DT888S4;Peroperativ absorption af natriumbicarbonatskyllevæske		1	
DT888S5;Peroperativ absorption af glukoseskyllevæske		1	
DT888S6;Peroperativ absorption af sorbitolskyllevæske		1	
DT888S7;Peroperativ absorption af glycinskyllevæske		1	
DT888S8;Peroperativ absorption af mannitolskyllevæske		1	
DT888T;TUR-P syndrom		1	
DT888U;Anden komplikation til endoskopi		1	
DT888U1;Medicinsk komplikation til koloskopi		1	
DT888X;Symptom eller klage vedrørende briller		1	
DT888Y;Symptom eller klage vedrørende kontaktlinser		1	
DT889;Komplikation til kirurgisk eller medicinsk behandling UNS		1	
DT89;Infektioner opstået i forbindelse med sygehusbehandling		1	
DT899;Nosokomiel infektion		1	
DT90;Følgetilstande efter læsion af hovedet		1	
DT900;Følgetilstand efter overfladisk læsion af hovedet		1	
DT901;Følgetilstand efter åbent sår på hovedet		1	
DT902;Følgetilstand efter fraktur af kraniet eller ansigtet		1	
DT902A;Følgetilstand efter fraktur i kraniet		1	
DT902B;Følgetilstand efter fraktur i ansigtet		1	
DT903;Følgetilstand efter læsion af kranienerve		1	

DT904;Følgetilstand efter læsion af øje eller øjenhule		1	
DT904B;Følgetilstand efter læsion af øje		1	
DT904C;Følgetilstand efter læsion af øjenhule		1	
DT905;Følgetilstand efter intrakraniel læsion		1	
DT908;Følgetilstand efter anden læsion af hovedet		1	
DT908A;Følgetilstand efter læsion af øre		1	
DT909;Følgetilstand efter læsion af hovedet UNS		1	
DT91;Følgetilstand efter læsion af halsen og kroppen		1	
DT910;Følgetilstand efter overfladisk læsion eller åbent sår på halsen eller kroppen		1	
DT911;Følgetilstand efter fraktur i rygsøjlen		1	
DT912;Følgetilstand efter anden fraktur i thorax eller bækkenet		1	
DT912A;Følgetilstand efter fraktur i bækkenet		1	
DT912B;Følgetilstand efter fraktur i thorax		1	
DT913;Følgetilstand efter rygmarvslæsion		1	
DT914;Følgetilstand efter læsion af intratorakalt organ		1	
DT915;Følgetilstand efter læsion af organ i abdomen eller bækkenet		1	
DT915A;Følgetilstand efter læsion af organ i abdomen		1	
DT915B;Følgetilstand efter læsion af organ i bækkenet		1	
DT918;Følgetilstand efter anden læsion af halsen eller kroppen		1	
DT919;Følgetilstand efter læsion af halsen eller kroppen UNS		1	
DT92;Følgetilstande efter læsion af overekstremitet		1	
DT920;Følgetilstand efter sårlæsion på overekstremitet		1	
DT921;Følgetilstand efter fraktur i arm		1	
DT922;Følgetilstand efter fraktur i håndled eller hånd		1	
DT922A;Følgetilstand efter fraktur i håndled		1	
DT922B;Følgetilstand efter fraktur i hånd		1	
DT923;Følgetilstand efter luksation eller distorsion i overekstremitet		1	
DT923A;Følgetilstand efter distorsion i overekstremitet		1	
DT923B;Følgetilstand efter luksation i overekstremitet		1	
DT924;Følgetilstand efter nervelæsion i overekstremitet		1	
DT925;Følgetilstand efter muskel- eller senelæsion i overekstremitet		1	
DT925A;Følgetilstand efter muskellæsion i overekstremitet		1	

DT925B;Følgetilstand efter senelæsion i overekstremitet		1	
DT926;Følgetilstand efter knusningslæsion eller traumatisk amputation på overekstremitet		1	
DT926A;Følgetilstand efter traumatisk amputation på overekstremitet		1	
DT926B;Følgetilstand efter knusningslæsion på overekstremitet		1	
DT928;Følgetilstand efter anden læsion af overekstremitet		1	
DT929;Følgetilstand efter læsion af overekstremitet UNS		1	
DT93;Følgetilstande efter læsion af underekstremitet		1	
DT930;Følgetilstand efter sårlæsion på underekstremitet		1	
DT931;Følgetilstand efter fraktur af lårben		1	
DT932;Følgetilstand efter anden fraktur i underekstremitet		1	
DT933;Følgetilstand efter luksation eller distorsion i underekstremitet		1	
DT933A;Følgetilstand efter distorsion i underekstremitet		1	
DT933B;Følgetilstand efter luksation i underekstremitet		1	
DT934;Følgetilstand efter nervelæsion i underekstremitet		1	
DT935;Følgetilstand efter muskel- eller senelæsion i underekstremitet		1	
DT935A;Følgetilstand efter muskellæsion i underekstremitet		1	
DT935B;Følgetilstand efter senelæsion i underekstremitet		1	
DT936;Følgetilstand efter knusningslæsion eller traumatisk amputation på underekstremitet		1	
DT936A;Følgetilstand efter knusningslæsion på underekstremitet		1	
DT936B;Følgetilstand efter traumatisk amputation på underekstremitet		1	
DT938;Følgetilstand efter anden læsion af underekstremitet		1	
DT939;Følgetilstand efter læsion af underekstremitet UNS		1	
DT94;Følgetilstande efter læsion i flere eller ikke specificerede legemsregioner		1	
DT940;Følgetilstand efter multiple læsioner i flere legemsregioner		1	
DT941;Følgetilstand efter læsioner uden angivelse af legemsregion		1	
DT95;Følgetilstande efter forbrændinger, ætsninger og forfrysninger		1	
DT950;Følgetilstand efter forbrænding, ætsning eller forfrysning på hovedet eller halsen		1	
DT950A;Følgetilstand efter forbrænding på hovedet		1	
DT950B;Følgetilstand efter forbrænding på halsen		1	
DT950C;Følgetilstand efter forfrysning på hovedet		1	
DT950D;Følgetilstand efter forfrysning på halsen		1	
DT950E;Følgetilstand efter ætsning på hovedet		1	

DT950F;Følgetilstand efter ætsning på halsen		1	
DT951;Følgetilstand efter forbrænding, ætsning eller forfrysning på kroppen		1	
DT951A;Følgetilstand efter forbrænding på kroppen		1	
DT951B;Følgetilstand efter forfrysning på kroppen		1	
DT951C;Følgetilstand efter ætsning på kroppen		1	
DT952;Følgetilstand efter forbrænding, ætsning eller forfrysning på overekstremitet		1	
DT952A;Følgetilstand efter forbrænding på overekstremitet		1	
DT952B;Følgetilstand efter forfrysning på overekstremitet		1	
DT952C;Følgetilstand efter ætsning på overekstremitet		1	
DT953;Følgetilstand efter forbrænding, ætsning eller forfrysning på underekstremitet		1	
DT953A;Følgetilstand efter forbrænding på underekstremitet		1	
DT953B;Følgetilstand efter forfrysning på underekstremitet		1	
DT953C;Følgetilstand efter ætsning på underekstremitet		1	
DT954;Følgetilstand efter forbrænding eller ætsning, som kun er klassificeret efter udbredelse		1	
DT954A;Følgetilstand efter forbrænding, som kun er klassificeret efter udbredelse		1	
DT954B;Følgetilstand efter ætsning, som kun er klassificeret efter udbredelse		1	
DT958;Følgetilstand efter anden forbrænding, ætsning eller forfrysning		1	
DT958A;Følgetilstand efter forbrænding, ætsning eller forfrysning, der involverer flere regioner		1	
DT959;Følgetilstand efter forbrænding, ætsning eller forfrysning UNS		1	
DT96;Følgetilstande efter forgiftning med lægemidler og biologiske substanser		1	
DT969;Følgetilstand efter forgiftning med lægemiddel eller biologisk substans		1	
DT969A;Følgetilstand efter forgiftning med lægemiddel		1	
DT969B;Følgetilstand efter forgiftning med biologisk substans		1	
DT97;Følgetilstande efter forgiftning med ikke-medicinske substanser		1	
DT979;Følgetilstand efter forgiftning med ikke-medicinsk substans		1	
DT98;Følgetilstande efter andre eller ikke specificerede virkninger af ydre påvirkninger		1	
DT980;Følgetilstand efter fremmedlegeme trængt ind gennem naturlig legemsåbning		1	
DT981;Følgetilstand efter anden eller ikke specificeret ydre påvirkning		1	
DT982;Følgetilstand efter visse tidlige komplikationer til læsion		1	
DT983;Følgetilstand efter lægelig behandling IKA		1	
DT983A;Følgetilstand efter epilepsikirurgi		1	
DT983B;Følgetilstand efter fedmekirurgi		1	

Supplement 2 Nielsen et al. Mortality after paediatric emergency calls for patients with and without pre-existing comorbidity (2023)

DT983C;Postoperativ seksuel dysfunktion		1	
DT983D;Følgetilstande efter kræftbehandling		1	
DT983D1;Følgetilstand med tarmdysfunktion efter kræftbehandling		1	
DT983D2;Følgetilstand med urinvejsdysfunktion efter kræftbehandling		1	
DT983D3;Følgetilstand med seksuel dysfunktion efter kræftbehandling		1	
DT983D4;Følgetilstand med kroniske smerter efter kræftbehandling		1	
DT983D5;Følgetilstand med træthed efter kræftbehandling		1	
DT983D6;Følgetilstand med hormonforstyrrelse efter kræftbehandling		1	
DT983D7;Følgetilstand med hjerte-kar-påvirkning efter kræftbehandling		1	
DT983D8;Følgetilstand med kognitiv forstyrrelse efter kræftbehandling		1	
DT983D9;Følgetilstand med skade i luftveje efter kræftbehandling		1	
DT983DA;Følgetilstand med lymfødem efter kræftbehandling		1	
DT983DB;Følgetilstand med symptomer fra muskler, skelet og led efter kræftbehandling		1	
DT983DC;Følgetilstand med symptomer fra mundhulen og svælget efter kræftbehandling		1	
DT983DD;Følgetilstand med neuropati efter kræftbehandling		1	
DT983DE;Følgetilstand med psykisk tilstand efter kræftbehandling		1	
DT983DF;Følgetilstand med søvnforstyrrelser efter kræftbehandling		1	
DT983DG;Følgetilstand med knogleskørhed efter kræftbehandling		1	
DT983DH;Følgetilstand med høretab og tinnitus efter kræftbehandling		1	
DT983DI;Følgetilstand med nedsat immunforsvar efter kræftbehandling		1	
DT983DJ;Følgetilstand med synsnedsættelse og synsforstyrrelse efter kræftbehandling		1	
DX60;Forsætlig selvbeskadigelse med ikke-opioide analgetika o.l.		1	
DX6001;Selvmord med ikke-opioidt analgetikum o.l. under patientkontakt		1	
DX6010;Selvmordsforsøg med ikke-opioidt analgetikum o.l. før patientkontakt		1	
DX6011;Selvmordsforsøg med ikke-opioidt analgetikum o.l. under patientkontakt		1	
DX6020;Selvbeskadigelse med ikke-opioidt analgetikum o.l. før patientkontakt		1	
DX6021;Selvbeskadigelse med ikke-opioidt analgetikum o.l. under patientkontakt		1	
DX61;Forsætlig selvbeskadigelse med psykofarmaka, antiepileptika og antiparkinsonmidler		1	
DX6101;Selvmord med psykofarmakum, antiepileptikum eller antiparkinsonmiddel under patientkontakt		1	
DX6110;Selvmordsforsøg med psykofarmakum, antiepileptikum eller antiparkinsonmiddel før patientkontakt		1	
DX6111;Selvmordsforsøg med psykofarmakum, antiepileptikum eller antiparkinsonmiddel under patientkontakt		1	
DX6120;Selvbeskadigelse med psykofarmakum, antiepileptikum eller antiparkinsonmiddel før patientkontakt		1	

Supplement 2 Nielsen et al. Mortality after paediatric emergency calls for patients with and without pre-existing comorbidity (2023)

DX6121;Selvbeskadigelse med psykofarmakum, antiepileptikum eller antiparkinsonmiddel under patientkontakt		1	
DX62;Forsætlig selvbeskadigelse med narkotika og psykodysleptika		1	
DX6201;Selvmord med narkotikum eller psykodysleptikum under patientkontakt		1	
DX6210;Selvmordsforsøg med narkotikum eller psykodysleptikum før patientkontakt		1	
DX6211;Selvmordsforsøg med narkotikum eller psykodysleptikum under patientkontakt		1	
DX6220;Selvbeskadigelse med narkotikum eller psykodysleptikum før patientkontakt		1	
DX6221;Selvbeskadigelse med narkotikum eller psykodysleptikum under patientkontakt		1	
DX63;Forsætlig selvbeskadigelse med midler med virkning på det autonome nervesystem		1	
DX6301;Selvmord med middel med virkning på autonome nervesystem under patientkontakt		1	
DX6310;Selvmordsforsøg med middel med virkning på autonome nervesystem før patientkontakt		1	
DX6311;Selvmordsforsøg med middel med virkning på autonome nervesystem under patientkontakt		1	
DX6320;Selvbeskadigelse med middel med virkning på autonome nervesystem før patientkontakt		1	
DX6321;Selvbeskadigelse med middel med virkning på autonome nervesystem under patientkontakt		1	
DX64;Forsætlig selvbeskadigelse med ikke specificerede lægemidler og biologiske stoffer		1	
DX6401;Selvmord med andet eller ikke specificeret lægemiddel eller biologisk stof under patientkontakt		1	
DX6410;Selvmordsforsøg med andet eller ikke specificeret lægemiddel eller biologisk stof før patientkontakt		1	
DX6411;Selvmordsforsøg med andet eller ikke specificeret lægemiddel eller biologisk stof under patientkontakt		1	
DX6420;Selvbeskadigelse med andet eller ikke specificeret lægemiddel eller biologisk stof før patientkontakt		1	
DX6421;Selvbeskadigelse med andet eller ikke specificeret lægemiddel eller biologisk stof under patientkontakt		1	
DX65;Forsætlig selvbeskadigelse med alkohol		1	
DX6501;Selvmord med alkohol under patientkontakt		1	
DX6510;Selvmordsforsøg med alkohol før patientkontakt		1	
DX6511;Selvmordsforsøg med alkohol under patientkontakt		1	
DX6520;Selvbeskadigelse med alkohol før patientkontakt		1	
DX6521;Selvbeskadigelse med alkohol under patientkontakt		1	
DX66;Forsætlig selvbeskadigelse med organiske opløsningsmidler og halogen-kulbrinter		1	
DX6601;Selvmord med organisk opløsningsmiddel eller halogen-kulbrinte under patientkontakt		1	
DX6610;Selvmordsforsøg med organisk opløsningsmiddel eller halogen-kulbrinte før patientkontakt		1	
DX6611;Selvmordsforsøg med organisk opløsningsmiddel eller halogen-kulbrinte under patientkontakt		1	
DX6620;Selvbeskadigelse med organisk opløsningsmiddel eller halogen-kulbrinte før patientkontakt		1	
DX6621;Selvbeskadigelse med organisk opløsningsmiddel eller halogen-kulbrinte under patientkontakt		1	
DX67;Forsætlig selvbeskadigelse med kulilte og andre gasarter og dampe		1	

Supplement 2 Nielsen et al. Mortality after paediatric emergency calls for patients with and without pre-existing comorbidity (2023)

DX6701;Selvmord med kulilte eller anden gasart og damp under patientkontakt		1	
DX6710;Selvmordsforsøg med kulilte eller anden gasart og damp før patientkontakt		1	
DX6711;Selvmordsforsøg med kulilte eller anden gasart og damp under patientkontakt		1	
DX6720;Selvbeskadigelse med kulilte eller anden gasart og damp før patientkontakt		1	
DX6721;Selvbeskadigelse med kulilte eller anden gasart og damp under patientkontakt		1	
DX68;Forsætlig selvbeskadigelse med bekæmpelsesmidler		1	
DX6801;Selvmord med bekæmpelsesmiddel under patientkontakt		1	
DX6810;Selvmordsforsøg med bekæmpelsesmiddel før patientkontakt		1	
DX6811;Selvmordsforsøg med bekæmpelsesmiddel under patientkontakt		1	
DX6820;Selvbeskadigelse med bekæmpelsesmiddel før patientkontakt		1	
DX6821;Selvbeskadigelse med bekæmpelsesmiddel under patientkontakt		1	
DX69;Forsætlig selvbeskadigelse med andre kemiske og toksiske stoffer		1	
DX6901;Selvmord med andet eller ikke specificeret kemisk eller toksisk stof under patientkontakt		1	
DX6910;Selvmordsforsøg med andet eller ikke specificeret kemisk eller toksisk stof før patientkontakt		1	
DX6911;Selvmordsforsøg med andet eller ikke specificeret kemisk eller toksisk stof under patientkontakt		1	
DX6920;Selvbeskadigelse med andet eller ikke specificeret kemisk eller toksisk stof før patientkontakt		1	
DX6921;Selvbeskadigelse med andet eller ikke specificeret kemisk eller toksisk stof under patientkontakt		1	
DX70;Forsætlig selvbeskadigelse ved hængning, strangulation og kvælning		1	
DX7001;Selvmord ved hængning, strangulation eller kvælning under patientkontakt		1	
DX7010;Selvmordsforsøg ved hængning, strangulation eller kvælning før patientkontakt		1	
DX7011;Selvmordsforsøg ved hængning, strangulation eller kvælning under patientkontakt		1	
DX7020;Selvbeskadigelse ved hængning, strangulation eller kvælning før patientkontakt		1	
DX7021;Selvbeskadigelse ved hængning, strangulation eller kvælning under patientkontakt		1	
DX71;Forsætlig selvbeskadigelse ved drukning og nedsænken i vand		1	
DX7101;Selvmord ved drukning under patientkontakt		1	
DX7110;Selvmordsforsøg ved drukning før patientkontakt		1	
DX7111;Selvmordsforsøg ved drukning under patientkontakt		1	
DX7120;Selvbeskadigelse ved drukning før patientkontakt		1	
DX7121;Selvbeskadigelse ved drukning under patientkontakt		1	
DX72;Forsætlig selvbeskadigelse ved skud fra håndvåben		1	
DX7201;Selvmord ved skud fra håndvåben under patientkontakt		1	
DX7210;Selvmordsforsøg ved skud fra håndvåben før patientkontakt		1	

DX7211;Selvmordsforsøg ved skud fra håndvåben under patientkontakt		1	
DX7220;Selvbeskadigelse ved skud fra håndvåben før patientkontakt		1	
DX7221;Selvbeskadigelse ved skud fra håndvåben under patientkontakt		1	
DX73;Forsætlig selvbeskadigelse ved skud fra gevær og andre større skydevåben		1	
DX7301;Selvmord ved skud fra gevær eller andet større skydevåben under patientkontakt		1	
DX7310;Selvmordsforsøg ved skud fra gevær eller andet større skydevåben før patientkontakt		1	
DX7311;Selvmordsforsøg ved skud fra gevær eller andet større skydevåben under patientkontakt		1	
DX7320;Selvbeskadigelse ved skud fra gevær eller andet større skydevåben før patientkontakt		1	
DX7321;Selvbeskadigelse ved skud fra gevær eller andet større skydevåben under patientkontakt		1	
DX74;Forsætlig selvbeskadigelse ved skud fra andre og ikke specificerede skydevåben		1	
DX7401;Selvmord ved skud fra andet eller ikke specificeret skydevåben under patientkontakt		1	
DX7410;Selvmordsforsøg ved skud fra andet eller ikke specificeret skydevåben før patientkontakt		1	
DX7411;Selvmordsforsøg ved skud fra andet eller ikke specificeret skydevåben under patientkontakt		1	
DX7420;Selvbeskadigelse ved skud fra andet eller ikke specificeret skydevåben før patientkontakt		1	
DX7421;Selvbeskadigelse ved skud fra andet eller ikke specificeret skydevåben under patientkontakt		1	
DX75;Forsætlig selvbeskadigelse ved eksplosive materialer		1	
DX7501;Selvmord ved eksplosivt materiale under patientkontakt		1	
DX7510;Selvmordsforsøg ved eksplosivt materiale før patientkontakt		1	
DX7511;Selvmordsforsøg ved eksplosivt materiale under patientkontakt		1	
DX7520;Selvbeskadigelse ved eksplosivt materiale før patientkontakt		1	
DX7521;Selvbeskadigelse ved eksplosivt materiale under patientkontakt		1	
DX76;Forsætlig selvbeskadigelse ved røg, ild og flammer		1	
DX7601;Selvmord ved røg, ild eller flammer under patientkontakt		1	
DX7610;Selvmordsforsøg ved røg, ild eller flammer før patientkontakt		1	
DX7611;Selvmordsforsøg ved røg, ild eller flammer under patientkontakt		1	
DX7620;Selvbeskadigelse ved røg, ild eller flammer før patientkontakt		1	
DX7621;Selvbeskadigelse ved røg, ild eller flammer under patientkontakt		1	
DX77;Forsætlig selvbeskadigelse ved varme dampe og varme genstande		1	
DX7701;Selvmord ved varm damp eller varm genstand under patientkontakt		1	
DX7710;Selvmordsforsøg ved varm damp eller varm genstand før patientkontakt		1	
DX7711;Selvmordsforsøg ved varm damp eller varm genstand under patientkontakt		1	
DX7720;Selvbeskadigelse ved varm damp eller varm genstand før patientkontakt		1	

DX7721;Selvbeskadigelse ved varm damp eller varm genstand under patientkontakt		1	
DX78;Forsætlig selvbeskadigelse med skarpe genstande		1	
DX7801;Selvmord med skarp genstand under patientkontakt		1	
DX7810;Selvmordsforsøg med skarp genstand før patientkontakt		1	
DX7811;Selvmordsforsøg med skarp genstand under patientkontakt		1	
DX7820;Selvbeskadigelse med skarp genstand før patientkontakt		1	
DX7821;Selvbeskadigelse med skarp genstand under patientkontakt		1	
DX79;Forsætlig selvbeskadigelse med stumpe genstande		1	
DX7901;Selvmord med stump genstand under patientkontakt		1	
DX7910;Selvmordsforsøg med stump genstand før patientkontakt		1	
DX7911;Selvmordsforsøg med stump genstand under patientkontakt		1	
DX7920;Selvbeskadigelse med stump genstand før patientkontakt		1	
DX7921;Selvbeskadigelse med stump genstand under patientkontakt		1	
DX80;Forsætlig selvbeskadigelse ved spring fra højde		1	
DX8001;Selvmord ved spring fra højde under patientkontakt		1	
DX8010;Selvmordsforsøg ved spring fra højde før patientkontakt		1	
DX8011;Selvmordsforsøg ved spring fra højde under patientkontakt		1	
DX8020;Selvbeskadigelse ved spring fra højde før patientkontakt		1	
DX8021;Selvbeskadigelse ved spring fra højde under patientkontakt		1	
DX81;Forsætlig selvbeskadigelse under køretøjer og genstande i bevægelse		1	
DX8101;Selvmord under køretøj eller genstand i bevægelse under patientkontakt		1	
DX8110;Selvmordsforsøg under køretøj eller genstand i bevægelse før patientkontakt		1	
DX8111;Selvmordsforsøg under køretøj eller genstand i bevægelse under patientkontakt		1	
DX8120;Selvbeskadigelse under køretøj eller genstand i bevægelse før patientkontakt		1	
DX8121;Selvbeskadigelse under køretøj eller genstand i bevægelse under patientkontakt		1	
DX82;Forsætlig selvbeskadigelse ved kollision med motorkøretøj		1	
DX8201;Selvmord ved kollision med motorkøretøj under patientkontakt		1	
DX8210;Selvmordsforsøg ved kollision med motorkøretøj før patientkontakt		1	
DX8211;Selvmordsforsøg ved kollision med motorkøretøj under patientkontakt		1	
DX8220;Selvbeskadigelse ved kollision med motorkøretøj før patientkontakt		1	
DX8221;Selvbeskadigelse ved kollision med motorkøretøj under patientkontakt		1	
DX83;Forsætlig selvbeskadigelse med andre specificerede metoder		1	

DX8301;Selvmord med anden specificeret metode under patientkontakt		1	
DX8310;Selvmordsforsøg med anden specificeret metode før patientkontakt		1	
DX8311;Selvmordsforsøg med anden specificeret metode under patientkontakt		1	
DX8320;Selvbeskadigelse med anden specificeret metode før patientkontakt		1	
DX8321;Selvbeskadigelse med anden specificeret metode under patientkontakt		1	
DX84;Forsætlig selvbeskadigelse med ikke specificerede metoder		1	
DX8401;Selvmord med ikke specificeret metode under patientkontakt		1	
DX8410;Selvmordsforsøg med ikke specificeret metode før patientkontakt		1	
DX8411;Selvmordsforsøg med ikke specificeret metode under patientkontakt		1	
DX8420;Selvbeskadigelse med ikke specificeret metode før patientkontakt		1	
DX8421;Selvbeskadigelse med ikke specificeret metode under patientkontakt		1	
DX85;Overgreb ved brug af lægemidler og biologiske stoffer		1	
DX859;Overgreb ved brug af lægemiddel eller biologisk stof		1	
DX86;Overgreb ved brug af ætsende stoffer		1	
DX869;Overgreb ved brug af ætsende stof		1	
DX87;Overgreb ved brug af bekæmpelsesmidler		1	
DX879;Overgreb ved brug af bekæmpelsesmiddel		1	
DX88;Overgreb ved brug af kulilte og andre gasarter og dampe		1	
DX889;Overgreb ved brug af kulilte eller anden gasart eller damp		1	
DX89;Overgreb ved brug af andre kemiske og toksiske stoffer		1	
DX899;Overgreb ved brug af andet kemisk eller toksisk stof		1	
DX90;Overgreb ved brug af ikke specificerede kemiske og giftige stoffer		1	
DX909;Overgreb ved brug af ikke specificeret kemisk eller giftigt stof		1	
DX91;Overgreb ved hængning, strangulation og kvælning		1	
DX919;Overgreb ved hængning, strangulation eller kvælning		1	
DX92;Overgreb ved drukning		1	
DX929;Overgreb ved drukning		1	
DX93;Overgreb ved skud fra håndskydevåben		1	
DX939;Overgreb ved skud fra håndskydevåben		1	
DX94;Overgreb ved skud fra geværer og andre større skydevåben		1	
DX949;Overgreb ved skud fra gevær eller andet større skydevåben		1	
DX95;Overgreb ved skud fra andre og ikke specificerede skydevåben		1	

DX959;Overgreb ved skud fra andet eller ikke specificeret skydevåben		1	
DX96;Overgreb ved brug af eksplosive materialer		1	
DX969;Overgreb ved brug af eksplosivt materiale		1	
DX97;Overgreb ved brug af røg, ild og flammer		1	
DX979;Overgreb ved brug af røg, ild eller flammer		1	
DX98;Overgreb ved brug af varme dampe og varme genstande		1	
DX989;Overgreb ved brug af varm damp eller varm genstand		1	
DX99;Overgreb ved brug af skarpe genstande		1	
DX999;Overgreb ved brug af skarp genstand		1	
DY00;Overgreb ved brug af stumpe genstande		1	
DY009;Overgreb ved brug af stump genstand		1	
DY01;Overgreb ved skub fra højde		1	
DY019;Overgreb ved skub fra højde		1	
DY02;Overgreb ved skub foran genstand i bevægelse		1	
DY029;Overgreb ved skub foran genstand i bevægelse		1	
DY03;Overgreb ved påkørsel med motorkøretøj		1	
DY039;Overgreb ved påkørsel med motorkøretøj		1	
DY04;Overgreb ved korporlig vold		1	
DY049;Overgreb ved korporlig vold		1	
DY05;Overgreb ved seksuel korporlig vold		1	
DY059;Overgreb ved seksuel korporlig vold		1	
DY06;Omsorgssvigt		1	
DY060;Omsorgssvigt fra ægtefælle/partner		1	
DY061;Omsorgssvigt fra forælder (forældre)		1	
DY062;Omsorgssvigt fra bekendt eller ven		1	
DY068;Omsorgssvigt fra anden person		1	
DY069;Omsorgssvigt fra ikke specificeret person		1	
DY07;Mishandling		1	
DY070;Mishandling fra ægtefælle eller partner		1	
DY071;Mishandling fra forælder (forældre)		1	
DY072;Mishandling fra bekendt eller ven		1	
DY073;Mishandling fra myndighed		1	

DY078;Mishandling fra anden person		1	
DY079;Mishandling fra ikke specificeret person		1	
DY08;Overgreb ved andre specificerede metoder		1	
DY089;Overgreb ved anden metode		1	
DY09;Overgreb ved ikke specificerede metoder		1	
DY099;Overgreb ved ikke specificeret metode		1	
DZ00;Undersøgelse af personer uden klager eller diagnoser	0		
DZ000;Kontakt mhp. generel helbredsundersøgelse	0		
DZ001;Kontakt mhp. planlagt generel børneundersøgelse	0		
DZ002;Kontakt mhp. undersøgelse af barn i periode med hurtig vækst	0		
DZ003;Kontakt mhp. undersøgelse af udviklingsstatus i puberteten	0		
DZ004;Kontakt mhp. psykiatrisk undersøgelse IKA	0		
DZ005;Kontakt mhp. undersøgelse af potentiel organ- eller vævsdonor	0		
DZ006;Kontakt mhp. undersøgelse som led i et klinisk forskningsprogram	0		
DZ008;Kontakt mhp. anden generel undersøgelse	0		
DZ008A;Kontakt mhp. generel undersøgelse mhp. visum	0		
DZ008B;Kontakt mhp. generel undersøgelse af asylansøger	0		
DZ01;Andre særlige undersøgelser af personer uden klager eller diagnoser	0		
DZ010;Kontakt mhp. undersøgelse af øjne eller syn	0		
DZ011;Kontakt mhp. undersøgelse af ører eller hørelse	0		
DZ012;Kontakt mhp. undersøgelse af tænder	0		
DZ013;Kontakt mhp. undersøgelse af blodtryk	0		
DZ014;Kontakt mhp. gynækologisk undersøgelse	0		
DZ015;Kontakt mhp. diagnostisk hud- eller allergitestning	0		
DZ016;Kontakt mhp. radiologisk undersøgelse	0		
DZ016M;Kontakt mhp. rutinemammografi	0		
DZ016U;Kontakt mhp. ultralydsundersøgelse	0		
DZ017;Kontakt mhp. laboratorieundersøgelse	0		
DZ018;Kontakt mhp. anden særlig undersøgelse	0		
DZ018A;Kontakt som følge af positiv screening for tyk- og endetarmskræft (det nationale screenir	0		
DZ018B;Adenomkontrolprogram, tidligere fund af mellem- eller højrisikoadenomer	0		
DZ018C;Kontakt pga. positiv høj-risiko HPV-test (HR-HPV)	0		

DZ019;Kontakt mhp. særlig undersøgelse UNS	0		
DZ02;Undersøgelse og fremmøde af administrative årsager	0		
DZ020;Kontakt mhp. undersøgelse før optagelse på undervisningsinstitution	0		
DZ021;Kontakt mhp. undersøgelse før erhvervsmæssig ansættelse	0		
DZ021A;Kontakt ifm. attestudstedelse til erhvervsdykker	0		
DZ021B;Kontakt ifm. attestudstedelse til pilot	0		
DZ022;Kontakt mhp. undersøgelse før anbringelse på beboelsesinstitution	0		
DZ023;Kontakt mhp. undersøgelse før rekruttering til militæret	0		
DZ024;Kontakt mhp. undersøgelse før erhvervelse af kørekort	0		
DZ025;Kontakt mhp. undersøgelse før deltagelse i sport	0		
DZ025A;Kontakt ifm. attestudstedelse til sportsdykker	0		
DZ026;Kontakt mhp. undersøgelse før forsikringstegning	0		
DZ026A;Kontakt mhp. undersøgelse ifm. pensionsforsikring	0		
DZ027;Kontakt mhp. udfyldelse af lægeattest	0		
DZ027A;Kontakt mhp. udfyldelse af dødsattest for anden person	0		
DZ028;Kontakt mhp. anden undersøgelse med administrativt formål	0		
DZ028A;Undersøgelse af projektpatient med administrativt formål	0		
DZ028B;Kontakt mhp. arbejdsmedicinsk undersøgelse ved graviditet	0		
DZ029;Kontakt mhp. undersøgelse af administrativ årsag UNS	0		
DZ03;Lægelig observation for og vurdering af personer mistænkt for sygdom	0		
DZ030;Observation pga. mistanke om tuberkulose	0		
DZ031;Observation pga. mistanke om kræft	0		
DZ031A;Observation pga. mistanke om kræft i centralnervesystemet	0		
DZ031AR;Observation pga. mistanke om recidiv af kræft i centralnervesystemet	0		
DZ031B;Observation pga. mistanke om kræft i lunge	0		
DZ031BR;Observation pga. mistanke om recidiv af kræft i lunge	0		
DZ031C;Observation pga. mistanke om kræft i spiserøret eller mavesækken	0		
DZ031C1;Observation pga. mistanke om kræft i spiserøret	0		
DZ031C1R;Observation pga. mistanke om recidiv af kræft i spiserøret	0		
DZ031C2;Observation pga. mistanke om kræft i cardia	0		
DZ031C2R;Observation pga. mistanke om recidiv af kræft i cardia	0		
DZ031C3;Observation pga. mistanke om kræft i mavesækken	0		

DZ031C3R;Observation pga. mistanke om recidiv af kræft i mavesækken	0		
DZ031CR;Observation pga. mistanke om recidiv af kræft i spiserøret eller mavesækken	0		
DZ031D;Observation pga. mistanke om kræft i tyktarmen eller endetarmen	0		
DZ031DA;Observation pga. mistanke om kræft i tyktarmen	0		
DZ031DAR;Observation pga. mistanke om recidiv af kræft i tyktarmen	0		
DZ031DB;Observation pga. mistanke om kræft i endetarmen	0		
DZ031DBR;Observation pga. mistanke om recidiv af kræft i endetarmen	0		
DZ031DC;Observation pga. mistanke om kræft i endetarmsåbningen	0		
DZ031DCR;Observation pga. mistanke om recidiv af kræft i endetarmsåbningen	0		
DZ031DR;Observation pga. mistanke om recidiv af kræft i tyktarmen eller endetarmen	0		
DZ031E;Observation pga. mistanke om kræft i leveren	0		
DZ031ER;Observation pga. mistanke om recidiv af kræft i leveren	0		
DZ031F;Observation pga. mistanke om kræft i pancreas	0		
DZ031FR;Observation pga. mistanke om recidiv af kræft i pancreas	0		
DZ031H;Observation pga. mistanke om kræft i urinveje	0		
DZ031H1;Observation pga. mistanke om kræft i nyre	0		
DZ031H1R;Observation pga. mistanke om recidiv af kræft i nyre	0		
DZ031H2;Observation pga. mistanke om kræft i urinblæren	0		
DZ031H2R;Observation pga. mistanke om recidiv af kræft i urinblæren	0		
DZ031H3;Observation pga. mistanke om kræft i urinlederen	0		
DZ031H3R;Observation pga. mistanke om recidiv af kræft i urinlederen	0		
DZ031H4;Observation pga. mistanke om kræft i urinrøret	0		
DZ031H4R;Observation pga. mistanke om recidiv af kræft i urinrøret	0		
DZ031HR;Observation pga. mistanke om recidiv af kræft i urinveje	0		
DZ031J;Observation pga. mistanke om kræft i prostata	0		
DZ031JR;Observation pga. mistanke om recidiv af kræft i prostata	0		
DZ031K;Observation pga. mistanke om kræft i kvindeligt kønsorgan	0		
DZ031K1;Observation pga. mistanke om kræft i æggestok	0		
DZ031K1R;Observation pga. mistanke om recidiv af kræft i æggestok	0		
DZ031K2;Observation pga. mistanke om kræft i livmoderen	0		
DZ031K2R;Observation pga. mistanke om recidiv af kræft i livmoderen	0		
DZ031K3;Observation pga. mistanke om kræft i livmoderhalsen	0		

DZ031K3R;Observation pga. mistanke om recidiv af kræft i livmoderhalsen	0			
DZ031K4;Observation pga. mistanke om kræft i vagina	0			
DZ031K4R;Observation pga. mistanke om recidiv af kræft i vagina	0			
DZ031K5;Observation pga. mistanke om kræft i vulva	0			
DZ031K5R;Observation pga. mistanke om recidiv af kræft i vulva	0			
DZ031KR;Observation pga. mistanke om recidiv af kræft i kvindeligt kønsorgan	0			
DZ031L;Observation pga. mistanke om kræft i galdegang	0			
DZ031LR;Observation pga. mistanke om recidiv af kræft i galdegang	0			
DZ031N;Observation pga. mistanke om kræft i knogle	0			
DZ031NR;Observation pga. mistanke om recidiv af kræft i knogle	0			
DZ031O;Observation pga. mistanke om kræft i muskel	0			
DZ031OR;Observation pga. mistanke om recidiv af kræft i muskel	0			
DZ031P;Observation pga. mistanke om kræft i hud	0			
DZ031PR;Observation pga. mistanke om recidiv af kræft i hud	0			
DZ031R;Observation pga. mistanke om kræft i bryst	0			
DZ031RR;Observation pga. mistanke om recidiv af kræft i bryst	0			
DZ031S;Observation pga. mistanke om kræft i penis	0			
DZ031SR;Observation pga. mistanke om recidiv af kræft i penis	0			
DZ031T;Observation pga. mistanke om kræft i testis	0			
DZ031TR;Observation pga. mistanke om recidiv af kræft i testis	0			
DZ031W;Observation pga. mistanke om kræft i binyre	0			
DZ031WR;Observation pga. mistanke om recidiv af kræft i binyre	0			
DZ031X;Observation pga. mistanke om kræft i hovedet eller halsen	0			
DZ031XA;Observation pga. mistanke om kræft i mundhulen	0			
DZ031XAR;Observation pga. mistanke om recidiv af kræft i mundhulen	0			
DZ031XB;Observation pga. mistanke om kræft i næsen eller bihule	0			
DZ031XBR;Observation pga. mistanke om recidiv af kræft i næsen eller bihule	0			
DZ031XC;Observation pga. mistanke om kræft i spytkirtel	0			
DZ031XCR;Observation pga. mistanke om recidiv af kræft i spytkirtlen	0			
DZ031XD;Observation pga. mistanke om kræft i skjoldbruskkirtlen	0			
DZ031XDR;Observation pga. mistanke om recidiv af kræft i skjoldbruskkirtlen	0			
DZ031XE;Observation pga. mistanke om kræft i struben eller svælget	0			

Supplement 2 Nielsen et al. Mortality after paediatric emergency calls for patients with and without pre-existing comorbidity (2023)

DZ031XER;Observation pga. mistanke om recidiv af kræft i struben eller svælget	0		
DZ031XF;Observation pga. mistanke om kræft i lymfeknude i halsen	0		
DZ031XFR;Observation pga. mistanke om recidiv af kræft i lymfeknude i halsen	0		
DZ031XR;Observation pga. mistanke om recidiv af kræft i hovedet eller halsen	0		
DZ031Y;Observation pga. mistanke om kræft i blod eller bloddannende organ	0		
DZ031YA;Observation pga. mistanke om lymfekræft eller kronisk lymfatisk leukæmi	0		
DZ031YAR;Observation pga. mistanke om recidiv af lymfekræft eller lymfatisk leukæmi	0		
DZ031YB;Observation pga. mistanke om kronisk myeloid sygdom	0		
DZ031YBR;Observation pga. mistanke om recidiv af myeloid sygdom	0		
DZ031YC;Observation pga. mistanke om akut leukæmi	0		
DZ031YCR;Observation pga. mistanke om recidiv af akut leukæmi	0		
DZ031YD;Observation pga. mistanke om myelomatose	0		
DZ031YDR;Observation pga. mistanke om recidiv af myelomatose	0		
DZ031YR;Observation pga. mistanke om recidiv af kræft i blod eller bloddannende organ	0		
DZ031Z;Observation pga. mistanke om kræft i øje eller øjenomgivelser	0		
DZ031ZR;Observation pga. mistanke om recidiv af kræft i øje eller øjenomgivelser	0		
DZ032;Observation pga. mistanke om psykisk lidelse eller adfærdsforstyrrelse	0		
DZ032A;Observation pga. mistanke om hyperkinetisk forstyrrelse	0		
DZ032X;Psykisk lidelse eller adfærdsforstyrrelse afkræftet	0		
DZ033;Observation pga. mistanke om sygdom i nervesystem	0		
DZ033A;Observation pga. mistanke om epilepsi	0		
DZ033B;Observation pga. mistanke om migræne	0		
DZ033C;Observation pga. mistanke om dissemineret sklerose	0		
DZ033D;Observation pga. mistanke om hjernerystelse	0		
DZ033E;Observation pga. mistanke om apopleksi	0		
DZ033F;Observation pga. mistanke om følge af hjerneskade	0		
DZ034;Observation pga. mistanke om myokardieinfarkt	0		
DZ035;Observation pga. mistanke om anden hjerte-kar-lidelse	0		
DZ035A;Observation pga. mistanke om arytmi	0		
DZ035B;Observation pga. mistanke om blodtryksforhøjelse	0		
DZ035C;Observation pga. mistanke om stabil angina pectoris	0		
DZ035D;Observation pga. mistanke om klaplidelse eller endokarditis	0		

DZ035E;Observation pga. mistanke om hjertesvigt	0			
DZ036;Observation pga. mistanke om toksisk effekt af indtaget stof	0			
DZ037;Observation pga. mistanke om medfødt eller perinatal sygdom	0			
DZ037A;Observation pga. mistanke om medfødt høretab	0			
DZ038;Observation pga. mistanke om anden sygdom eller tilstand	0			
DZ038A;Observation pga. mistanke om urinvejsinfektion	0			
DZ038B;Observation pga. mistanke om urinvejssygdom UNS	0			
DZ038C;Observation pga. mistanke om allergisk lidelse	0			
DZ038D;Observation pga. mistanke om blodsygdom	0			
DZ038E;Observation pga. mistanke om tumor UNS	0			
DZ038F;Observation pga. mistanke om underernæring	0			
DZ038G;Observation pga. mistanke om fysisk overgreb/mishandling	0			
DZ038H;Observation pga. mistanke om gigt- eller bindevævssygdom	0			
DZ038J;Observation pga. mistanke om røgforgiftning	0			
DZ038K;Observation pga. mistanke om sygdom i fordøjelsesorganer	0			
DZ038KA;Observation pga. mistanke om gastrointestinal perforation	0			
DZ038M;Observation pga. mistanke om prænatal rusmiddeleksposition	0			
DZ038O;Observation pga. mistanke om sygdom i forbindelse med graviditet, fødsel eller barsel	0			
DZ038P;Observation pga. mistanke om infektion	0			
DZ038PA;Observation pga. mistanke om virusinfektion	0			
DZ038PA1;Observation pga. mistanke om COVID-19-infektion	0			
DZ038Q;Observation pga. mistanke om følger efter COVID-19	0			
DZ038R;Observation pga. mistanke om lungesygdom	0			
DZ038RA;Observation pga. mistanke om kronisk obstruktiv lungesygdom	0			
DZ038S;Observation pga. mistanke om vaccineinduceret immun trombotisk trombocytopeni (VITT	0			
DZ038T;Observation pga. mistanke om komplikation til behandling	0			
DZ038TA;Observation pga. mistanke om komplikation til kirurgisk indgreb	0			
DZ038U;Observation pga. mistanke om medfødt misdannelse eller kromosomanomali	0			
DZ039;Observation pga. mistanke om sygdom eller tilstand UNS	0			
DZ04;Undersøgelse og observation af andre årsager	0			
DZ040;Kontakt mhp. undersøgelse for alkohol eller lægemidler i blodet	0			
DZ040A;Kontakt mhp. undersøgelse for alkohol i blodet	0			

DZ040B;Kontakt mhp. undersøgelse for lægemidler i blodet	0		
DZ041;Kontakt mhp. undersøgelse og observation efter færdselsulykke	0		
DZ042;Kontakt mhp. undersøgelse og observation efter arbejdsulykke	0		
DZ043;Kontakt mhp. undersøgelse og observation efter andet ulykkestilfælde	0		
DZ044;Kontakt mhp. undersøgelse og observation efter mistænkt voldtægt	0		
DZ045;Kontakt mhp. undersøgelse og observation efter anden påført skade	0		
DZ046;Kontakt mhp. psykiatrisk undersøgelse begæret af myndigheder	0		
DZ048;Kontakt mhp. undersøgelse og observation af anden årsag	0		
DZ048A;Kontakt mhp. vurdering af kosmetisk generende hudforandring	0		
DZ048B;Kontakt mhp. arbejdsmedicinsk udredning	0		
DZ048C;Kontakt mhp. retsmedicinsk bestemmelse af knoglealder	0		
DZ048D;Kontakt mhp. retsmedicinsk undersøgelse for ulovligt tarmindhold	0		
DZ048E;Kontakt mhp. erhvervsevnevurdering	0		
DZ048F;Kontakt mhp. miljømedicinsk udredning	0		
DZ048G;Kontakt mhp. DNA-undersøgelse af blodet	0		
DZ048H;Kontakt mhp. undersøgelse af helbredsrisiko	0		
DZ048S;Kontakt mhp. socialmedicinsk udredning	0		
DZ048T;Kontakt mhp. medicingennemgang	0		
DZ048W;Kontakt mhp. retsmedicinsk undersøgelse UNS	0		
DZ049;Kontakt mhp. undersøgelse og observation UNS	0		
DZ06;Undersøgelsesresultater vedrørende helbredstilstand	0		
DZ061;Undersøgelsesresultater vedrørende fjernmetastaser til kræftsygdom	0		
DZ061A;Ingen fjernmetastaser påvist	0		
DZ061B;Metastase status ikke afklaret	0		
DZ062;Undersøgelsesresultater vedrørende lymfeknudemetastaser til kræftsygdom	0		
DZ062A;Ingen lymfeknudemetastaser påvist	0		
DZ062B;Lymfeknude status ikke afklaret	0		
DZ063;Undersøgelsesresultater vedrørende lokalrecidiv ved kræftsygdom	0		
DZ063A;Intet lokalrecidiv påvist	0		
DZ063B;Lokalrecidiv status ikke afklaret	0		
DZ063C;Lokalrecidiv status ikke relevant	0		
DZ07;Sundhedsfagligt vurderet risiko for sygdom eller tilstand	0		

Supplement 2 Nielsen et al. Mortality after paediatric emergency calls for patients with and without pre-existing comorbidity (2023)

DZ071;Prænatal risikofaktor	0		
DZ071A;Prænatal eksposition for alkohol	0		
DZ071B;Prænatal eksposition for anden psykoaktiv substans	0		
DZ071B1;Prænatal eksposition for opioid	0		
DZ071B1B;Prænatal eksposition for buprenorfin	0		
DZ071B1H;Prænatal eksposition for heroin	0		
DZ071B1M;Prænatal eksposition for metadon	0		
DZ071B1P;Prænatal eksposition for andet opioat	0		
DZ071B2;Prænatal eksposition for cannabis	0		
DZ071B3;Prænatal eksposition for benzodiazepin	0		
DZ071B4;Prænatal eksposition for barbiturat	0		
DZ071B5;Prænatal eksposition for kokain	0		
DZ071B6;Prænatal eksposition for amfetamin	0		
DZ071B7;Prænatal eksposition for anden centralstimulerende el. psykoaktiv substans	0		
DZ071B8;Prænatal eksposition for nikotin	0		
DZ079;Risiko for sygdom eller tilstand UNS	0		
DZ08;Kontrolundersøgelse efter behandling af kræft			2
DZ080;Kontrolundersøgelse efter operation af kræft			2
DZ080A;Kontrolundersøgelse efter operation af kræft i centralnervesystemet			2
DZ080B;Kontrolundersøgelse efter operation af kræft i lunge			2
DZ080C;Kontrolundersøgelse efter operation af kræft i mavesæk			2
DZ080D;Kontrolundersøgelse efter operation af kræft i tyktarm			2
DZ080E;Kontrolundersøgelse efter operation af kræft i lever			2
DZ080F;Kontrolundersøgelse efter operation af kræft i pancreas			2
DZ080G;Kontrolundersøgelse efter operation af kræft i endetarm			2
DZ080H;Kontrolundersøgelse efter operation af kræft i nyre			2
DZ080I;Kontrolundersøgelse efter operation af kræft i urinblære			2
DZ080J;Kontrolundersøgelse efter operation af kræft i prostata			2
DZ080K;Kontrolundersøgelse efter operation af kræft i æggestok			2
DZ080L;Kontrolundersøgelse efter operation af kræft i livmoder			2
DZ080M;Kontrolundersøgelse efter operation af kræft i livmoderhals			2
DZ080N;Kontrolundersøgelse efter operation af kræft i knogle			2

DZ080O;Kontrolundersøgelse efter operation af kræft i muskel			2
DZ080P;Kontrolundersøgelse efter operation af kræft i hud			2
DZ080Q;Kontrolundersøgelse efter operation af hoved- og halskræft			2
DZ080R;Kontrolundersøgelse efter operation af kræft i bryst			2
DZ080S;Kontrolundersøgelse efter operation af kræft i penis			2
DZ080T;Kontrolundersøgelse efter operation af kræft i testis			2
DZ080U;Kontrolundersøgelse efter operation af kræft i urinleder			2
DZ080V;Kontrolundersøgelse efter operation af kræft i urinrør			2
DZ080W;Kontrolundersøgelse efter operation af kræft i lungehinde			2
DZ080X;Kontrolundersøgelse efter operation af kræft i vagina			2
DZ080Y;Kontrolundersøgelse efter operation af kræft i vulva			2
DZ080Z;Kontrolundersøgelse efter operation af kræft i andre lokalisationer			2
DZ080ZA;Kontrolundersøgelse efter operation af kræft i æggeleder			2
DZ081;Kontrolundersøgelse efter strålebehandling af kræft			2
DZ081A;Kontrolundersøgelse efter strålebehandling af kræft i centralnervesystemet			2
DZ081B;Kontrolundersøgelse efter strålebehandling af kræft i lunge			2
DZ081C;Kontrolundersøgelse efter strålebehandling af kræft i mavesæk			2
DZ081D;Kontrolundersøgelse efter strålebehandling af kræft i tyktarm			2
DZ081E;Kontrolundersøgelse efter strålebehandling af kræft i lever			2
DZ081F;Kontrolundersøgelse efter strålebehandling af kræft i pancreas			2
DZ081G;Kontrolundersøgelse efter strålebehandling af kræft i endetarm			2
DZ081H;Kontrolundersøgelse efter strålebehandling af kræft i nyre			2
DZ081I;Kontrolundersøgelse efter strålebehandling af kræft i urinblære			2
DZ081J;Kontrolundersøgelse efter strålebehandling af kræft i prostata			2
DZ081K;Kontrolundersøgelse efter strålebehandling af kræft i æggestok			2
DZ081L;Kontrolundersøgelse efter strålebehandling af kræft i livmoder			2
DZ081M;Kontrolundersøgelse efter strålebehandling af kræft i livmoderhals			2
DZ081N;Kontrolundersøgelse efter strålebehandling af kræft i knogle			2
DZ081O;Kontrolundersøgelse efter strålebehandling af kræft i muskel			2
DZ081P;Kontrolundersøgelse efter strålebehandling af kræft i hud			2
DZ081Q;Kontrolundersøgelse efter strålebehandling af hoved- og halskræft			2
DZ081R;Kontrolundersøgelse efter strålebehandling af kræft i bryst			2

DZ081S;Kontrolundersøgelse efter strålebehandling af kræft i penis			2
DZ081T;Kontrolundersøgelse efter strålebehandling af kræft i testis			2
DZ081U;Kontrolundersøgelse efter strålebehandling af kræft i urinleder			2
DZ081V;Kontrolundersøgelse efter strålebehandling af kræft i urinrør			2
DZ081W;Kontrolundersøgelse efter strålebehandling af kræft i lungehinde			2
DZ081X;Kontrolundersøgelse efter strålebehandling af kræft i vagina			2
DZ081Y;Kontrolundersøgelse efter strålebehandling af kræft i vulva			2
DZ081Z;Kontrolundersøgelse efter strålebehandling af kræft i andre lokalisationer			2
DZ081ZA;Kontrolundersøgelse efter strålebehandling af kræft i æggeleder			2
DZ082;Kontrolundersøgelse efter kemoterapi af kræft			2
DZ082A;Kontrolundersøgelse efter kemoterapi af kræft i centralnervesystemet			2
DZ082B;Kontrolundersøgelse efter kemoterapi af kræft i lunge			2
DZ082C;Kontrolundersøgelse efter kemoterapi af kræft i mavesæk			2
DZ082D;Kontrolundersøgelse efter kemoterapi af kræft i tyktarm			2
DZ082E;Kontrolundersøgelse efter kemoterapi af kræft i lever			2
DZ082F;Kontrolundersøgelse efter kemoterapi af kræft i pancreas			2
DZ082G;Kontrolundersøgelse efter kemoterapi af kræft i endetarm			2
DZ082H;Kontrolundersøgelse efter kemoterapi af kræft i nyre			2
DZ082I;Kontrolundersøgelse efter kemoterapi af kræft i urinblære			2
DZ082J;Kontrolundersøgelse efter kemoterapi af kræft i prostata			2
DZ082K;Kontrolundersøgelse efter kemoterapi af kræft i æggestok			2
DZ082L;Kontrolundersøgelse efter kemoterapi af kræft i livmoder			2
DZ082M;Kontrolundersøgelse efter kemoterapi af kræft i livmoderhals			2
DZ082N;Kontrolundersøgelse efter kemoterapi af kræft i knogle			2
DZ082O;Kontrolundersøgelse efter kemoterapi af kræft i muskel			2
DZ082P;Kontrolundersøgelse efter kemoterapi af kræft i huden			2
DZ082Q;Kontrolundersøgelse efter kemoterapi af hoved- og halskræft			2
DZ082R;Kontrolundersøgelse efter kemoterapi af kræft i bryst			2
DZ082S;Kontrolundersøgelse efter kemoterapi af kræft i penis			2
DZ082T;Kontrolundersøgelse efter kemoterapi af kræft i testis			2
DZ082U;Kontrolundersøgelse efter kemoterapi af kræft i urinleder			2
DZ082V;Kontrolundersøgelse efter kemoterapi af kræft i urinrør			2

DZ082W;Kontrolundersøgelse efter kemoterapi af kræft i lungehinde			2
DZ082X;Kontrolundersøgelse efter kemoterapi af kræft i vagina			2
DZ082Y;Kontrolundersøgelse efter kemoterapi af kræft i vulva			2
DZ082Z;Kontrolundersøgelse efter kemoterapi af kræft i andre lokalisationer			2
DZ082ZA;Kontrolundersøgelse efter kemoterapi af kræft i æggeleder			2
DZ087;Kontrolundersøgelse efter kombineret behandling af kræft			2
DZ087A;Kontrolundersøgelse efter kombineret behandling af kræft i centralnervesystemet			2
DZ087B;Kontrolundersøgelse efter kombineret behandling af kræft i lunge			2
DZ087C;Kontrolundersøgelse efter kombineret behandling af kræft i mavesæk			2
DZ087D;Kontrolundersøgelse efter kombineret behandling af kræft i tyktarm			2
DZ087E;Kontrolundersøgelse efter kombineret behandling af kræft i lever			2
DZ087F;Kontrolundersøgelse efter kombineret behandling af kræft i pancreas			2
DZ087G;Kontrolundersøgelse efter kombineret behandling af kræft i endetarm			2
DZ087H;Kontrolundersøgelse efter kombineret behandling af kræft i nyre			2
DZ087I;Kontrolundersøgelse efter kombineret behandling af kræft i urinblære			2
DZ087J;Kontrolundersøgelse efter kombineret behandling af kræft i prostata			2
DZ087K;Kontrolundersøgelse efter kombineret behandling af kræft i æggestok			2
DZ087L;Kontrolundersøgelse efter kombineret behandling af kræft i livmoder			2
DZ087M;Kontrolundersøgelse efter kombineret behandling af kræft i livmoderhals			2
DZ087N;Kontrolundersøgelse efter kombineret behandling af kræft i knogle			2
DZ087O;Kontrolundersøgelse efter kombineret behandling af kræft i muskel			2
DZ087P;Kontrolundersøgelse efter kombineret behandling af kræft i huden			2
DZ087Q;Kontrolundersøgelse efter kombineret behandling af hoved- og halskræft			2
DZ087R;Kontrolundersøgelse efter kombineret behandling af kræft i bryst			2
DZ087S;Kontrolundersøgelse efter kombineret behandling af kræft i penis			2
DZ087T;Kontrolundersøgelse efter kombineret behandling af kræft i testis			2
DZ087U;Kontrolundersøgelse efter kombineret behandling af kræft i urinleder			2
DZ087V;Kontrolundersøgelse efter kombineret behandling af kræft i urinrør			2
DZ087W;Kontrolundersøgelse efter kombineret behandling af kræft i lungehinde			2
DZ087X;Kontrolundersøgelse efter kombineret behandling af kræft i vagina			2
DZ087Y;Kontrolundersøgelse efter kombineret behandling af kræft i vulva			2
DZ087Z;Kontrolundersøgelse efter kombineret behandling af kræft i andre lokalisationer			2

Supplement 2 Nielsen et al. Mortality after paediatric emergency calls for patients with and without pre-existing comorbidity (2023)

DZ087ZA;Kontrolundersøgelse efter kombineret behandling af kræft i æggeleder			2
DZ088;Kontrolundersøgelse efter anden behandling af kræft			2
DZ089;Kontrolundersøgelse efter behandling af kræft UNS			2
DZ09;Kontrolundersøgelse efter behandling af andre sygdomme end kræft	0		
DZ090;Kontrolundersøgelse efter operation af anden tilstand	0		
DZ091;Kontrolundersøgelse efter strålebehandling af anden tilstand	0		
DZ092;Kontrolundersøgelse efter kemoterapi af anden tilstand	0		
DZ093;Kontrolundersøgelse efter psykoterapi	0		
DZ094;Kontrolundersøgelse efter behandling af fraktur	0		
DZ094A;Kontrolundersøgelse efter behandling af collum chirurgicum-fraktur	0		
DZ094B;Kontrolundersøgelse efter behandling af anden humerus-fraktur	0		
DZ094C;Kontrolundersøgelse efter behandling af Colles-fraktur	0		
DZ094D;Kontrolundersøgelse efter behandling af fingerfraktur	0		
DZ094E;Kontrolundersøgelse efter behandling af columnafraktur	0		
DZ094F;Kontrolundersøgelse efter behandling af bækkenfraktur	0		
DZ094G;Kontrolundersøgelse efter behandling af collum femoris-fraktur	0		
DZ094H;Kontrolundersøgelse efter behandling af femurfraktur	0		
DZ094I;Kontrolundersøgelse efter behandling af knæfraktur	0		
DZ094J;Kontrolundersøgelse efter behandling af crusfraktur	0		
DZ094K;Kontrolundersøgelse efter behandling af malleolfraktur	0		
DZ094L;Kontrolundersøgelse efter behandling af calcaneus-fraktur	0		
DZ095;Kontrolundersøgelse efter behandling af medicinsk sygdom	0		
DZ095A;Kontrolundersøgelse efter behandling af hjertesygdom	0		
DZ097;Kontrolundersøgelse efter kombineret behandling af anden tilstand	0		
DZ098;Kontrolundersøgelse efter anden behandling af anden tilstand	0		
DZ098A;Kontrolundersøgelse efter medicinsk tidlig abort	0		
DZ098B;Kontrolundersøgelse efter behandling af ikke-ondartet tumor	0		
DZ098C;Kontrolundersøgelse efter abort IKA	0		
DZ098D;Kontrolundersøgelse efter ECMO-behandling	0		
DZ098N;Kontrolundersøgelse efter neurorehabilitering af hjerneskade	0		
DZ098P;Kontrolundersøgelse efter psykiatrisk behandling	0		
DZ099;Kontrolundersøgelse efter behandling af anden tilstand UNS	0		

DZ099X;Kontrol efter behandling af sygdom UNS	0			
DZ10;Rutinemæssig helbredsundersøgelse af definerede befolkningsgrupper	0			
DZ100;Kontakt som led i arbejdsmedicinsk helbredsundersøgelse	0			
DZ100A;Kontakt som led i arbejdsmedicinsk helbredsundersøgelse af gravid	0			
DZ101;Kontakt som led i rutinemæssig helbredsundersøgelse af institutionsbeboere	0			
DZ102;Kontakt som led i rutinemæssig helbredsundersøgelse af militærpersonale	0			
DZ103;Kontakt som led i rutinemæssig helbredsundersøgelse af sportshold	0			
DZ104;Hjemtagningsundersøgelse efter udsendelse i international mission	0			
DZ108;Kontakt som led i rutinemæssig helbredsundersøgelse af anden defineret befolkningsgrup	0			
DZ109;Kontakt som led i rutinemæssig helbredsundersøgelse af befolkning UNS	0			
DZ11;Screening for infektiøse og parasitære sygdomme	0			
DZ110;Screening for infektiøse tarmsygdomme	0			
DZ111;Screening for tuberkulose i luftvejene	0			
DZ112;Screening for anden bakteriel sygdom	0			
DZ113;Screening for overvejende seksuelt overført infektion	0			
DZ113A;Screening for gonoré	0			
DZ113B;Screening for syflis	0			
DZ114;Screening for HIV-infektion	0			
DZ114A;HIV screening ved 1. graviditetsundersøgelse	0			
DZ114B;HIV screening ved gentagelse	0			
DZ115;Screening for anden virussygdom	0			
DZ115A;Screening for COVID-19	0			
DZ116;Screening for anden protozosygdom eller ormesygdom	0			
DZ116A;Screening for ormesygdom	0			
DZ116B;Screening for protozosygdom	0			
DZ118;Screening for anden infektiøs eller parasitær sygdom	0			
DZ119;Screening for infektiøs eller parasitær sygdom UNS	0			
DZ119A;Screening for infektiøs sygdom UNS	0			
DZ119B;Screening for parasitær sygdom UNS	0			
DZ12;Screening for kræft	0			
DZ120;Screening for kræft i mavesæk	0			
DZ121;Screening for kræft i mave-tarm-kanalen	0			

Supplement 2 Nielsen et al. Mortality after paediatric emergency calls for patients with and without pre-existing comorbidity (2023)

DZ122;Screening for kræft i åndedrætsorganer	0			
DZ123;Screening for kræft i bryst	0			
DZ123A;Mammografiscreening	0			
DZ123AA;Mammografiscreening i henhold til sundhedsloven § 85	0			
DZ124;Screening for kræft i livmoderhals	0			
DZ124A;Screening ved prøvetagning (smear) for kræft i livmoderhals	0			
DZ125;Screening for kræft i prostata	0			
DZ125A;Screening ved PSA bestemmelse for kræft i prostata	0			
DZ126;Screening for kræft i urinblære	0			
DZ127;Screening for kræft i æggestok	0			
DZ128;Screening for kræft med anden lokalisation	0			
DZ128A;Screening for kræft i pancreas	0			
DZ129;Screening for kræft UNS	0			
DZ13;Screening for andre sygdomme og tilstande	0			
DZ130;Screening for blodsygdom, sygdom i bloddannende organ eller forstyrrelse i immunforsvar	0			
DZ131;Screening for diabetes	0			
DZ132;Screening for ernæringsforstyrrelse	0			
DZ133;Screening for mental eller adfærdsmæssig forstyrrelse	0			
DZ134;Screening for udviklingsforstyrrelse i barnealderen	0			
DZ135;Screening for øjen- eller ørelidelse	0			
DZ135A;Screening for øjenlidelse	0			
DZ135A1;Screening for diabetisk retinopati	0			
DZ135A2;Screening for glaukom	0			
DZ135A3;Screening for degeneratio maculae luteae	0			
DZ135B;Screening for ørelidelse	0			
DZ135C;Screening for medfødt høretab	0			
DZ136;Screening for hjerte-kar-sygdom	0			
DZ137;Screening for medfødt misdannelse eller kromosomanomali	0			
DZ138;Screening for anden sygdom eller tilstand	0			
DZ139;Screening for sygdom eller tilstand UNS	0			
DZ20;Kontakt med eller udsat for smitte med overførbare sygdomme	0			
DZ200;Kontakt med eller udsat for smitte med infektiøs tarmsygdom	0			

DZ201;Kontakt med eller udsat for smitte med tuberkulose	0			
DZ202;Kontakt med eller udsat for smitte med infektion med overvejende seksuel overførsel	0			
DZ203;Kontakt med eller udsat for smitte med hundegalskab	0			
DZ204;Kontakt med eller udsat for smitte med røde hunde	0			
DZ205;Kontakt med eller udsat for smitte med viral hepatitis	0			
DZ206;Kontakt med eller udsat for smitte med HIV	0			
DZ207;Kontakt med eller udsat for smitte med lus, mider eller andre ektoparasitter	0			
DZ208;Kontakt med eller udsat for smitte med anden infektionssygdom	0			
DZ209;Kontakt med eller udsat for smitte med infektionssygdom UNS	0			
DZ21;Asymptomatisk HIV-infektion	0			
DZ219;Asymptomatisk HIV-infektion UNS	0			
DZ22;Asymptomatisk bærer af infektiøs sygdom	0			
DZ220;Tyfusbacilbærer	0			
DZ221;Bærer af anden infektiøs tarmsygdom	0			
DZ222;Difteribacilbærer	0			
DZ223;Bærer af anden bakteriel sygdom	0			
DZ223A;Meningokok bærertilstand	0			
DZ224;Bærer af infektion, som hovedsagelig er seksuelt overført	0			
DZ226;Bærer af HTLV-1 virus	0			
DZ227;Latent tuberkulose	0			
DZ228;Bærer af anden infektiøs sygdom	0			
DZ229;Bærer af infektiøs sygdom UNS	0			
DZ23;Kontakt mhp. vaccination mod en enkelt bakteriel sygdom	0			
DZ230;Kontakt mhp. vaccination mod kolera	0			
DZ231;Kontakt mhp. vaccination mod tyfus	0			
DZ232;Kontakt mhp. vaccination mod tuberkulose (BCG)	0			
DZ233;Kontakt mhp. vaccination mod pest	0			
DZ234;Kontakt mhp. vaccination mod tularæmi	0			
DZ235;Kontakt mhp. vaccination mod stivkrampe	0			
DZ236;Kontakt mhp. vaccination mod difteri	0			
DZ237;Kontakt mhp. vaccination mod kighoste	0			
DZ238;Kontakt mhp. vaccination mod anden bakteriel sygdom	0			

DZ24;Kontakt mhp. vaccination mod visse virale sygdomme	0			
DZ240;Kontakt mhp. vaccination mod polio	0			
DZ241;Kontakt mhp. vaccination mod leddyrsbåren hjernebetændelse	0			
DZ242;Kontakt mhp. vaccination mod hundegalskab	0			
DZ243;Kontakt mhp. vaccination mod gul feber	0			
DZ244;Kontakt mhp. vaccination mod mæslinger	0			
DZ245;Kontakt mhp. vaccination mod røde hunde	0			
DZ246;Kontakt mhp. vaccination mod leverbetændelse	0			
DZ25;Kontakt mhp. vaccination mod andre virale sygdomme	0			
DZ250;Kontakt mhp. vaccination mod fåresyge	0			
DZ251;Kontakt mhp. vaccination mod influenza	0			
DZ252;Kontakt mhp. vaccination mod COVID-19	0			
DZ258;Kontakt mhp. vaccination mod anden viral sygdom	0			
DZ26;Kontakt mhp. vaccination mod andre infektionssygdomme	0			
DZ260;Kontakt mhp. vaccination mod leishmaniasis	0			
DZ268;Kontakt mhp. vaccination mod anden infektionssygdom	0			
DZ269;Kontakt mhp. vaccination mod infektionssygdom UNS	0			
DZ27;Kontakt mhp. kombinerede vaccinationer mod infektionssygdomme	0			
DZ270;Kontakt mhp. vaccination mod kolera og tyfus (kolera+TAB)	0			
DZ271;Kontakt mhp. vaccination mod difteri-stivkrampe-kighoste (DTP)	0			
DZ272;Kontakt mhp. vaccination mod DTP+kolera og tyfus	0			
DZ273;Kontakt mhp. vaccination mod DTP+polio	0			
DZ274;Kontakt mhp. vaccination mod mæslinger, fåresyge og røde hunde (MFR)	0			
DZ278;Kontakt mhp. vaccination mod anden kombination af infektionssygdomme	0			
DZ279;Kontakt mhp. vaccination mod kombination af sygdomme UNS	0			
DZ29;Kontakt mhp. andre forebyggende foranstaltninger	0			
DZ290;Kontakt mhp. profylaktisk isolationsindlæggelse	0			
DZ291;Kontakt mhp. profylaktisk immunoterapi	0			
DZ292;Kontakt mhp. anden profylaktisk kemoterapi	0			
DZ292A;Kontakt mhp. profylaktisk antibiotikabehandling	0			
DZ298;Kontakt mhp. anden forebyggende foranstaltning	0			
DZ299;Kontakt mhp. forebyggende foranstaltning UNS	0			

DZ30;Kontakter ifm. graviditetsforebyggelse	0		
DZ300;Kontakt mhp. almen rådgivning og vejledning om graviditetsforebyggelse	0		
DZ301;Kontakt mhp. indsættelse af intrauterin antikonception	0		
DZ302;Kontakt mhp. sterilisation	0		
DZ303;Kontakt mhp. postkoital graviditetsforebyggelse	0		
DZ304;Kontakt mhp. kontrol af antikonception med lægemiddel	0		
DZ304A;Kontakt mhp. kontrol af antikonception med P-piller	0		
DZ304B;Kontakt mhp. kontrol af antikonception med P-stav	0		
DZ305;Kontakt mhp. kontrol af intrauterin antikonception med spiral	0		
DZ308;Kontakt mhp. anden graviditetsforebyggelse	0		
DZ308A;Kontakt mhp. kontrol af antikonception med pessar	0		
DZ308P;Kontakt pga. problemer med graviditetsforebyggelse	0		
DZ309;Kontakt mhp. graviditetsforebyggelse UNS	0		
DZ31;Kontakter ifm. rådgivning og behandling vedrørende graviditet og barnløshed	0		
DZ310;Kontakt mhp. kirurgisk refertilisering	0		
DZ310A;Kontakt mhp. tuboplastik efter tidligere sterilisation	0		
DZ310B;Kontakt mhp. vasoplastik efter tidligere sterilisation	0		
DZ311;Kontakt mhp. kunstig insemination	0		
DZ312;Kontakt mhp. in vitro-befrugtning	0		
DZ313;Kontakt mhp. anden assisteret befrugtning	0		
DZ314;Kontakt mhp. fertilitetsundersøgelse	0		
DZ315;Kontakt mhp. genetisk rådgivning	0		
DZ316;Kontakt mhp. almindelig rådgivning ved barnløshed	0		
DZ317;Prækonceptionel rådgivning	0		
DZ318;Kontakt mhp. anden fertilitetsbehandling	0		
DZ318A;Kontakt mhp. fertilitetsbehandling hvor partner har HIV eller hepatitis	0		
DZ319;Kontakt mhp. fertilitetsbehandling UNS	0		
DZ32;Graviditetsundersøgelse	0		
DZ320;Ubekræftet graviditet	0		
DZ321;Bekræftet graviditet	0		
DZ3211;Graviditet bekræftet ved påvisning af foster	0		
DZ321A;Graviditet bekræftet ved u-hCG	0		

Supplement 2 Nielsen et al. Mortality after paediatric emergency calls for patients with and without pre-existing comorbidity (2023)

DZ321B;Graviditet bekræftet ved s-hCG	0			
DZ321C;Intrauterin graviditet bekræftet ved ultralyd	0			
DZ321E;Bekræftet graviditet efter oplægning af optøede embryoner	0			
DZ321F;Bekræftet graviditet efter mikroinsemination	0			
DZ321G;Bekræftet graviditet efter IUI	0			
DZ321H;Bekræftet graviditet efter IVF	0			
DZ321J;Bekræftet spontan graviditet	0			
DZ321K;Bekræftet graviditet efter TESE	0			
DZ321L;Bekræftet graviditet med ukendt lokalisation (PUL)	0			
DZ322;Undersøgelse forud for provokeret abort	0			
DZ323;Graviditet som tilfældigt fund	0			
DZ324;Uønsket graviditet	0			
DZ33;Henvist til fødsel	0			
DZ338;Planlagt fødsel	0			
DZ34;Normal graviditet	0			
DZ340;Graviditet, førstegangsfødende	0			
DZ348;Anden graviditet	0			
DZ348A;Graviditet, flergangsfødende	0			
DZ348B;Graviditet, paritet ukendt	0			
DZ349;Normal graviditet UNS	0			
DZ35;Højrisikograviditet	0			
DZ350;Graviditet efter tidligere infertilitet	0			
DZ351;Graviditet efter tidligere abort	0			
DZ351A;Graviditet efter tidligere spontan senabort (GA 12+0 - 21+6)	0			
DZ351B;Graviditet efter tidligere provokeret senabort (GA 12+0 - 21+6)	0			
DZ352;Graviditet efter tidligere peri- eller neonatalt dødsfald	0			
DZ353;Graviditet efter tidligere insufficient moderomsorg	0			
DZ354;Graviditet hos mangegangsfødende kvinde	0			
DZ355;Graviditet hos ældre førstegangsfødende (>= 40 år)	0			
DZ356;Graviditet hos meget ung førstegangsfødende	0			
DZ357;Graviditet med sociale problemer	0			
DZ358;Anden højrisikograviditet	0			

Supplement 2 Nielsen et al. Mortality after paediatric emergency calls for patients with and without pre-existing comorbidity (2023)

DZ358A;Graviditet efter tidligere intrauterin væksthæmning (IUGR)	0			
DZ358B;Graviditet efter tidligere præterm fødsel	0			
DZ358C;Graviditet efter tidligere fødsel af misdannet barn	0			
DZ358D;Graviditet efter tidligere kompliceret fødsel	0			
DZ358D1;Graviditet efter tidligere sfinkterruptur	0			
DZ358E;Graviditet efter tidligere kejsersnit	0			
DZ358F;IVF-graviditet	0			
DZ358H;Graviditet efter hormonstimulation	0			
DZ358J;Graviditet ved mindre liv end normalt	0			
DZ358K;Graviditet efter ægdonation	0			
DZ358L;Graviditet med traume	0			
DZ358M;Graviditet ved rusmiddelproblematik	0			
DZ358M1;Rusmiddelforbrug i aktuel graviditet	0			
DZ358M10;Alkoholforbrug i aktuel graviditet	0			
DZ358M11;Opioidforbrug i aktuel graviditet	0			
DZ358M11B;Forbrug af buprenorfin i aktuel graviditet	0			
DZ358M11H;Forbrug af heroin i aktuel graviditet	0			
DZ358M11M;Forbrug af metadon i aktuel graviditet	0			
DZ358M11P;Forbrug af andet opiat i aktuel graviditet	0			
DZ358M12;Cannabisforbrug i aktuel graviditet	0			
DZ358M13;Benzodiazepinforbrug i aktuel graviditet	0			
DZ358M14;Barbituratforbrug i aktuel graviditet	0			
DZ358M15;Kokainforbrug i aktuel graviditet	0			
DZ358M16;Amfetaminforbrug i aktuel graviditet	0			
DZ358M17;Forbrug af andet centralstimulerende stof eller psykoaktivt middel i aktuel graviditet	0			
DZ358M18;Tobaksforbrug i aktuel graviditet	0			
DZ358M2;Rusmiddelforbrug før aktuel graviditet	0			
DZ358M20;Graviditet med tidligere forbrug af alkohol	0			
DZ358M21;Graviditet med tidligere forbrug af opioid	0			
DZ358M21B;Graviditet med tidligere forbrug af buprenorfin	0			
DZ358M21H;Graviditet med tidligere forbrug af heroin	0			
DZ358M21M;Graviditet med tidligere forbrug af metadon	0			

DZ358M21P;Graviditet med tidligere forbrug af andet opiat	0			
DZ358M22;Graviditet med tidligere forbrug af cannabis	0			
DZ358M23;Graviditet med tidligere forbrug af benzodiazepin	0			
DZ358M24;Graviditet med tidligere forbrug af barbiturat	0			
DZ358M25;Graviditet med tidligere forbrug af kokain	0			
DZ358M26;Graviditet med tidligere forbrug af amfetamin	0			
DZ358M27;Graviditet med tidligere forbrug af andet centralstimulerende stof eller psykoaktivt mid	0			
DZ358M3;Graviditet med rusmiddelforbrug hos relateret person	0			
DZ358N;Graviditet med øget risiko for genetisk afvigelse	0			
DZ358O;Graviditet efter tidligere graviditet med abnormt foster	0			
DZ358O1;Graviditet efter tidligere graviditet med misdannet foster	0			
DZ358O2;Graviditet efter tidligere graviditet med foster med kromosomsygdom	0			
DZ358O3;Graviditet efter tidligere graviditet med foster med arvelig sygdom	0			
DZ358P;Graviditet hos ældre fødende	0			
DZ358Q;Graviditet med tidligere svær præeklampsi/HELLP	0			
DZ358R;Graviditet med tidligere spiseforstyrrelse	0			
DZ358S;Graviditet med tidligere fødsel med komplicerende blødning > 1000 ml	0			
DZ359;Højrisikograviditet UNS	0			
DZ36;Prænatal screening	0			
DZ360;Prænatal screening for kromosomale abnormiteter	0			
DZ361;Prænatal screening for forhøjet alfaføtoprotein	0			
DZ362;Anden prænatal screening baseret på amniocentese	0			
DZ363;Prænatal screening for medfødte misdannelser ved ultralyd eller anden fysisk metode	0			
DZ364;Prænatal screening for intrauterin væksthæmning ved ultralyd eller anden fysisk metode	0			
DZ365;Prænatal screening for autoimmunisation	0			
DZ368;Anden prænatal screening	0			
DZ368U;Prænatal ultralydsundersøgelse med normalt undersøgelsesresultat	0			
DZ368U1;Prænatal ultralydsundersøgelse normalt undersøgelsesresultat vedr. foster	0			
DZ368U2;Prænatal ultralydsundersøgelse normalt undersøgelsesresultat vedr. moder	0			
DZ369;Prænatal screening UNS	0			
DZ38;Levendefødt barn efter fødested	0			
DZ380;Levendefødt barn, født på sygehus (findes ikke i SKS)	0			

Supplement 2 Nielsen et al. Mortality after paediatric emergency calls for patients with and without pre-existing comorbidity (2023)

DZ380Q;Levendefødt barn født på sygehus overf. u planlagt hjemmefødsel (findes ikke i SKS)	0			
DZ380R;Lev.født barn født på sygehus overf. u planlagt fødsel på fødeklinik (findes ikke i SKS)	0			
DZ381;Levendefødt barn, født uden for sygehus (findes ikke i SKS)	0			
DZ381A;Levendefødt barn, født på vej til sygehus (findes ikke i SKS)	0			
DZ381B;Levendefødt barn, hjemmefødt (findes ikke i SKS)	0			
DZ381B1;Levendefødt barn, hjemmefødt, planlagt (findes ikke i SKS)	0			
DZ381B2;Levendefødt barn, hjemmefødt, ikke planlagt (findes ikke i SKS)	0			
DZ382;Levendefødt barn uden angivelse af fødested (findes ikke i SKS)	0			
DZ383;Tvilling, født på sygehus (findes ikke i SKS)	0			
DZ384;Tvilling, født uden for sygehus (findes ikke i SKS)	0			
DZ384A;Tvilling, født på vej til sygehus (findes ikke i SKS)	0			
DZ384B;Tvilling, hjemmefødt (findes ikke i SKS)	0			
DZ386;Levendefødt ved anden flerfødsel, født på sygehus (findes ikke i SKS)	0			
DZ389;Levendefødt barn	0			
DZ39;Undersøgelse og pleje efter fødsel	0			
DZ390;Pleje og undersøgelse efter fødsel	0			
DZ391;Care and examination of lactating mother (findes ikke i SKS)	0			
DZ392;Rutineundersøgelse efter fødsel	0			
DZ393;Postpartum undersøgelse begrundet i rusmiddelproblematik	0			
DZ3931;Postpartum undersøgelse pga rusmiddelforbrug i graviditet	0			
DZ39310;Postpartum undersøgelse ved forbrug af alkohol i graviditet	0			
DZ39311;Postpartum undersøgelse ved forbrug af opioid i graviditet	0			
DZ39311B;Postpartum undersøgelse ved forbrug af buprenorfin i graviditet	0			
DZ39311H;Postpartum undersøgelse ved forbrug af heroin i graviditet	0			
DZ39311M;Postpartum undersøgelse ved forbrug af metadon i graviditet	0			
DZ39311P;Postpartum undersøgelse ved forbrug af andet opiat i graviditet	0			
DZ39312;Postpartum undersøgelse ved forbrug af cannabis i graviditet	0			
DZ39313;Postpartum undersøgelse ved forbrug af benzodiazepin i graviditet	0			
DZ39314;Postpartum undersøgelse ved forbrug af barbiturat i graviditet	0			
DZ39315;Postpartum undersøgelse ved forbrug af kokain i graviditet	0			
DZ39316;Postpartum undersøgelse ved forbrug af amfetamin i graviditet	0			
DZ39317;Postpartum undersøgelse ved forbrug af andet centralstimulerende eller psykoaktivt mid	0			

DZ39318;Postpartum undersøgelse ved forbrug af tobak i graviditet	0		
DZ3932;Postpartum undersøgelse pga rusmiddelforbrug før graviditet	0		
DZ39320;Postpartum undersøgelse pga forbrug af alkohol før graviditet	0		
DZ39321;Postpartum undersøgelse pga forbrug af opioid før graviditet	0		
DZ39321B;Postpartum undersøgelse pga forbrug af buprenorfin før graviditet	0		
DZ39321H;Postpartum undersøgelse pga forbrug af heroin før graviditet	0		
DZ39321M;Postpartum undersøgelse pga forbrug af metadon før graviditet	0		
DZ39321P;Postpartum undersøgelse pga forbrug af andet opiat før graviditet	0		
DZ39322;Postpartum undersøgelse pga forbrug af cannabis før graviditet	0		
DZ39323;Postpartum undersøgelse pga forbrug af benzodiazepin før graviditet	0		
DZ39324;Postpartum undersøgelse pga forbrug af barbiturat før graviditet	0		
DZ39325;Postpartum undersøgelse pga forbrug af kokain før graviditet	0		
DZ39326;Postpartum undersøgelse pga forbrug af amfetamin før graviditet	0		
DZ39327;Postpartum undersøgelse pga forbrug af andet centralstimulerende eller psykoaktivt mi	0		
DZ3933;Postpartum undersøgelse pga rusmiddelforbrug hos relateret person	0		
DZ3939;Postpartum undersøgelse ved mistanke om rusmiddelproblematik UNS	0		
DZ40;Kontakter mhp. forebyggende indgreb	0		
DZ400;Kontakt mhp. forebyggende indgreb relateret til kræftsygdom	0		
DZ400A;Kontakt mhp. forebyggende indgreb på andet organ ved kræftsygdom			2
DZ400B;Kontakt mhp. indgreb på andet organ som behandling ved kræftsygdom			2
DZ408;Kontakt mhp. andet forebyggende indgreb	0		
DZ408A;Kontakt mhp. indgreb på organ ved risiko for udvikling af sygdom	0		
DZ409;Kontakt mhp. forebyggende indgreb UNS	0		
DZ41;Kontakt mhp. foranstaltninger uden direkte behandlingsformål	0		
DZ410;Kontakt mhp. hårtransplantation	0		
DZ411;Kontakt mhp. kosmetisk kirurgi	0		
DZ412;Kontakt mhp. rutinemæssig eller rituel omskæring	0		
DZ412A;Kontakt mhp. rutinemæssig omskæring	0		
DZ412B;Kontakt mhp. rituel omskæring	0		
DZ418;Kontakt mhp. anden foranstaltning uden direkte behandlingsformål	0		
DZ418A;Kontakt mhp. fjernelse af uønsket hårvækst	0		
DZ418B;Kontakt mhp. anæstesiologisk ydelse	0		

Supplement 2 Nielsen et al. Mortality after paediatric emergency calls for patients with and without pre-existing comorbidity (2023)

DZ419;Kontakt mhp. ikke nærmere specificeret foranstaltning uden direkte behandlingsformål	0		
DZ42;Kontakter mhp. plastikkirurgisk efterbehandling	0		
DZ420;Kontakt mhp. plastikkirurgisk efterbehandling på hovedet eller halsen	0		
DZ421;Kontakt mhp. plastikkirurgisk efterbehandling af bryst	0		
DZ422;Kontakt mhp. plastikkirurgisk efterbehandling på andre dele af kroppen	0		
DZ423;Kontakt mhp. plastikkirurgisk efterbehandling på overekstremitet	0		
DZ424;Kontakt mhp. plastikkirurgisk efterbehandling på underekstremitet	0		
DZ428;Kontakt mhp. plastikkirurgisk efterbehandling i anden legemsregion	0		
DZ429;Kontakt mhp. plastikkirurgisk efterbehandling UNS	0		
DZ47;Kontakter mhp. andre ortopædiske efterbehandlinger	0		
DZ470;Kontakt mhp. fjernelse af internt fiksationsmateriale	0		
DZ478;Kontakt mhp. anden ortopædisk efterbehandling	0		
DZ478A;Kontakt mhp. skiftning af bandage eller skinne til samme type	0		
DZ478B;Kontakt mhp. skiftning af bandage eller skinne til anden type	0		
DZ479;Kontakt mhp. ortopædisk efterbehandling UNS	0		
DZ48;Kontakter mhp. andre efterbehandlinger efter kirurgisk indgreb	0		
DZ480;Kontakt mhp. tilsyn med forbindinger eller suturer efter operation	0		
DZ488;Kontakt mhp. anden efterbehandling efter operation	0		
DZ488C;Rekonstruktion efter tidligere tumorkirurgi			2
DZ488D;Kontakt mhp. postoperativ vurdering efter fedmekirurgi	0		
DZ489;Efterbehandling efter kirurgisk indgreb UNS	0		
DZ50;Kontakter mhp. genoptræning	0		
DZ500;Kontakt mhp. genoptræning efter hjertetilfælde	0		
DZ501;Kontakt mhp. anden fysioterapi	0		
DZ502;Kontakt mhp. genoptræning efter alkoholmisbrug	0		
DZ503;Kontakt mhp. genoptræning efter medicinmisbrug	0		
DZ504;Kontakt mhp. psykoterapi IKA	0		
DZ505;Kontakt mhp. taleterapi	0		
DZ506;Kontakt mhp. ortoptisk træning	0		
DZ507;Kontakt mhp. ergoterapi eller erhvervsgenoptræning IKA	0		
DZ507A;Kontakt mhp. ergoterapi	0		
DZ508;Kontakt mhp. anden form for genoptræning	0		

DZ508A;Kontakt mhp. genoptræning efter fødsel	0		
DZ508B;Kontakt mhp. specialiseret spiserehabilitering pga. sondeafhængighed	0		
DZ509;Kontakt mhp. genoptræning UNS	0		
DZ51;Særlige indikationer for sundhedsfaglig indsats	0		
DZ515;Kontakt mhp. palliativ behandling			2
DZ515S;Kontakt mhp. specialiseret palliativ indsats			2
DZ518;Anden særlig indikation for sundhedsfaglig indsats	0		
DZ518A;Kontakt mhp. afslutning af tidligt hjemmeophold (THO)	0		
DZ52;Kontakter mhp. donation af organer og væv	0		
DZ520;Kontakt mhp. donation af blod	0		
DZ521;Kontakt mhp. donation af hud	0		
DZ522;Kontakt mhp. donation af knogle	0		
DZ523;Kontakt mhp. donation af knoglemarv	0		
DZ524;Kontakt mhp. donation af nyre	0		
DZ525;Kontakt mhp. donation af hornhinde	0		
DZ526;Kontakt mhp. donation af lever	0		
DZ527;Kontakt mhp. donation af hjerte	0		
DZ528;Kontakt mhp. donation af andet organ eller væv	0		
DZ529;Kontakt mhp. donation af organ eller væv UNS	0		
DZ54;Rekonvalescens	0		
DZ540;Rekonvalescens efter operation	0		
DZ541;Rekonvalescens efter strålebehandling			2
DZ542;Rekonvalescens efter kemoterapi			2
DZ543;Rekonvalescens efter psykoterapi	0		
DZ544;Rekonvalescens efter behandling af knoglebrud	0		
DZ547;Rekonvalescens efter kombineret behandling	0		
DZ548;Rekonvalescens efter anden behandling	0		
DZ549;Rekonvalescens efter behandling UNS	0		
DZ55;Problemer med undervisning og læseevne	0		
DZ550;Analfabetisme eller ringe læseevne	0		
DZ551;Undervisning ikke tilgængelig eller uopnåelig	0		
DZ552;Dumpet til eksamen	0		

DZ553;Dårlig præstation i skole	0			
DZ554;Undervisningsmæssigt utilpasset med lærere eller i klassen	0			
DZ558;Andet problem med undervisning eller læseevne	0			
DZ559;Problem med undervisning eller læseevne UNS	0			
DZ56;Problemer med beskæftigelse og arbejdsløshed	0			
DZ560;Problem med arbejdsløshed	0			
DZ561;Problem med jobskift	0			
DZ562;Problem med trussel om afskedigelse	0			
DZ563;Problem med stressende arbejdsforhold	0			
DZ564;Problem med disharmoni med chef eller kolleger	0			
DZ565;Problem med ubehageligt arbejde	0			
DZ566;Problem med anden fysisk eller mental arbejdsbelastning	0			
DZ566A;Problem med anden fysisk arbejdsbelastning	0			
DZ566B;Problem med anden mental arbejdsbelastning	0			
DZ567;Andet eller ikke specificeret problem med beskæftigelse eller arbejdsløshed	0			
DZ57;Erhvervsmæssig udsættelse for risikofaktorer	0			
DZ570;Erhvervsmæssig udsættelse for støj	0			
DZ571;Erhvervsmæssig udsættelse for stråling	0			
DZ572;Erhvervsmæssig udsættelse for støv	0			
DZ573;Erhvervsmæssig udsættelse for anden luftforurening	0			
DZ574;Erhvervsmæssig udsættelse for giftige midler i landbrug	0			
DZ575;Erhvervsmæssig udsættelse for giftige midler i industri	0			
DZ576;Erhvervsmæssig udsættelse for ekstreme temperaturer	0			
DZ577;Erhvervsmæssig udsættelse for vibrationer	0			
DZ578;Erhvervsmæssig udsættelse for anden risikofaktor	0			
DZ579;Erhvervsmæssig udsættelse for risikofaktor UNS	0			
DZ58;Problemer med fysiske omgivelser	0			
DZ580;Udsættelse for støj	0			
DZ581;Udsættelse for luftforurening	0			
DZ582;Udsættelse for vandforurening	0			
DZ583;Udsættelse for jordforurening	0			
DZ584;Udsættelse for stråling	0			

DZ585;Udsættelse for anden forurening	0		
DZ586;Udsættelse for utilstrækkelig drikkevandsforsyning	0		
DZ587;Udsættelse for tobaksrygning	0		
DZ587A;Udsættelse for passiv rygning	0		
DZ588;Andet problem med fysiske omgivelser	0		
DZ589;Problem med fysiske omgivelser UNS	0		
DZ59;Problemer med boligforhold og økonomiske forhold	0		
DZ590;Problem med hjemløshed	0		
DZ590A;Posedame	0		
DZ590B;Vagabond	0		
DZ591;Problem med dårlige boligforhold	0		
DZ592;Problem med stridigheder med naboer, logerende eller husvært	0		
DZ593;Problem med at bo på institution	0		
DZ594;Problem med mangel på tilstrækkelig mad	0		
DZ595;Problem med ekstrem fattigdom	0		
DZ596;Problem med lav indkomst	0		
DZ597;Problem med utilstrækkelig social sikring eller velfærdsstøtte	0		
DZ598;Andet problem med boligforhold eller økonomisk forhold	0		
DZ599;Problem med boligforhold eller økonomisk forhold UNS	0		
DZ60;Andre problemer relateret til det sociale miljø	0		
DZ600;Problem med tilpasning til forandringer i livscyklus	0		
DZ601;Problem med atypisk forældresituation	0		
DZ602;Problem med at være aleneboende	0		
DZ603;Problem med kulturelle tilpasningsvanskeligheder	0		
DZ604;Problem med social udelukkelse eller afvisning	0		
DZ605;Problem med formodet diskrimination eller forfølgelse	0		
DZ608;Andet problem relateret til det sociale miljø	0		
DZ609;Problem relateret til det sociale miljø UNS	0		
DZ61;Problemer på grund af negative oplevelser i barndommen	0		
DZ610;Problem på grund af tab af følelsesmæssig kontakt i barndommen	0		
DZ611;Problem på grund af fjernelse fra barndomshjem	0		
DZ612;Problem på grund af ændret familiemønster i barndommen	0		

DZ613;Problem på grund af tab af selvfølelse i barndommen	0		
DZ614;Problem på grund af seksuelt overgreb fra nærtstående person i barndommen		1	
DZ615;Problem på grund af seksuelt overgreb fra fjerntstående person i barndommen		1	
DZ616;Problem pga. fysisk overgreb i barndommen		1	
DZ617;Problem på grund af stærkt skræmmende oplevelser i barndommen		1	
DZ618;Problem på grund af anden negativ hændelse i barndommen	0		
DZ619;Problem på grund af negativ hændelse i barndommen UNS	0		
DZ62;Andre problemer i forbindelse med opvæksten	0		
DZ620;Problem på grund af utilstrækkeligt opsyn og kontrol fra forældres side	0		
DZ621;Problem på grund af forældres overbeskyttelse	0		
DZ622;Problem på grund af opvækst på institution	0		
DZ623;Problem på grund af fjendtlighed over for barn eller barn gjort til syndebuk	0		
DZ624;Problem på grund af følelsesmæssig forsømmelse af barn	0		
DZ625;Andre problemer i forbindelse med opdragelsesmæssig forsømmelse	0		
DZ626;Problem på grund af urimeligt forældrepres eller anden fejl i opdragelsen	0		
DZ628;Andet problem i forbindelse med opvæksten	0		
DZ629;Problem i forbindelse med opvæksten UNS	0		
DZ63;Andre problemer med primære støttegruppe	0		
DZ630;Problem med forholdet til ægtefælle eller partner	0		
DZ631;Problem med forholdet til forældre eller svigerforældre	0		
DZ632;Problem med utilstrækkelig støtte fra familien	0		
DZ633;Problem med fravær af familiemedlem	0		
DZ634;Problem på grund af et familiemedlems forsvinden eller død	0		
DZ635;Problem på grund af opløsning af familien ved separation eller skilsmisse	0		
DZ636;Problem på grund af afhængigt familiemedlem, som behøver pleje i hjemmet	0		
DZ637;Andet problem på grund af belastende begivenhed i familien eller husstanden	0		
DZ638;Andet problem med primære støttegruppe	0		
DZ639;Problem med primære støttegruppe UNS	0		
DZ64;Problemer i forbindelse med visse psykosociale forhold	0		
DZ640;Problem i forbindelse med uønsket graviditet	0		
DZ641;Problem i forbindelse med mange fødsler		1	
DZ642;Problem i forbindelse med at udsætte sig for risikable eller skadelige fysiske påvirkninger		1	

DZ643;Problem i forbindelse med at udsætte sig for risikable eller skadelige psykiske påvirkninger		1	
DZ644;Problem i forbindelse med uoverensstemmelse med rådgivere	0		
DZ65;Problemer i forbindelse med andre psykosociale forhold	0		
DZ650;Problem i forbindelse med domfældelse uden fængselsstraf		1	
DZ651;Problem i forbindelse med fængsling eller anbringelse		1	
DZ651A;Problem i forbindelse med dom til psykiatrisk behandling		1	
DZ651B;Problem i forbindelse med dom til psykiatrisk anbringelse		1	
DZ652;Problem i forbindelse med løsladelse fra fængsel		1	
DZ653;Problem i forbindelse med andet retsligt forhold		1	
DZ654;Problem i forbindelse med udsættelse for kriminalitet eller terrorisme		1	
DZ655;Problem i forbindelse med udsættelse for katastrofe, krig eller andre fjendtligheder		1	
DZ658;Andet problem i forbindelse med psykosociale forhold	0		
DZ658A;Væsentlige psykosociale problemer	0		
DZ659;Problem i forbindelse med psykosociale forhold UNS	0		
DZ70;Rådgivning om seksuel holdning, adfærd eller orientering	0		
DZ700;Rådgivning vedrørende seksuel holdning	0		
DZ701;Rådgivning vedrørende seksuel adfærd eller orientering	0		
DZ702;Rådgivning vedrørende seksuel adfærd hos tredje part	0		
DZ703;Rådgivning om blandede problemer vedrørende seksuel holdning, adfærd eller orientering	0		
DZ708;Anden seksualrådgivning	0		
DZ709;Seksualrådgivning UNS	0		
DZ71;Personer i kontakt med sundhedsvæsenet mhp. rådgivning og lægelig vejledning IKA	0		
DZ710;Person i konsultation på anden persons vegne	0		
DZ711;Rådgivning ved bekymring for sygdom hos rask person	0		
DZ712;Kontakt mhp. forklaring af undersøgelsesresultat	0		
DZ713;Diætetisk rådgivning og kontrol	0		
DZ713A;Behov for diætetisk kontrol	0		
DZ713A1;Behov for kontrol af ketogen diæt	0		
DZ713B;Behov for diætetisk rådgivning	0		
DZ714;Rådgivning og kontrol ved alkoholmisbrug	0		
DZ715;Rådgivning og kontrol ved medicinmisbrug	0		
DZ716;Rådgivning og kontrol ved tobaksmisbrug	0		

DZ717;Rådgivning ifm. HIV-infektion	0		
DZ718;Anden rådgivning	0		
DZ718A;Rådgivning om muligheder for kræftbehandling			2
DZ718B;Rådgivning om behandlingsmuligheder UNS		1	
DZ718B1;Behov for præoperativ vurdering inden epilepsikirurgi		1	
DZ718B2;Behov for præoperativ vurdering inden fedmekirurgi		1	
DZ719;Rådgivning UNS	0		
DZ72;Problemer i forbindelse med livsstil	0		
DZ720;Problem med tobaksforbrug	0		
DZ720A;Problem med at være ryger	0		
DZ720B;Problem med at være passiv ryger	0		
DZ720E;Daglig rygning	0		
DZ721;Problem med alkoholforbrug	0		
DZ721C;Antal alkoholgenstande ugentligt overskrider 10	0		
DZ721D;Antal alkoholgenstande samme dag overskrider 4	0		
DZ722;Problem med stofbrug	0		
DZ723;Problem med mangel på fysisk aktivitet	0		
DZ724;Problem med forkerte kost- og spisevaner	0		
DZ725;Problem med seksuel højrisikoadfærd	0		
DZ726;Problem med spil eller væddemål	0		
DZ728;Andet problem med livsstil	0		
DZ7281;Problem med utilstrækkelig søvnhygiejne	0		
DZ729;Problem med livsstil UNS	0		
DZ73;Problemer med at klare tilværelsen	0		
DZ730;Problem med udbrændthed	0		
DZ731;Problem med forstærkede karaktertræk	0		
DZ732;Problem med mangel på afslapning og fritid	0		
DZ733;Problem med stress IKA	0		
DZ734;Problem med mangelfulde sociale færdigheder IKA	0		
DZ735;Problem med socialrollekonflikt IKA	0		
DZ736;Problem med aktivitetsbegrænsning på grund af funktionsnedsættelse	0		
DZ738;Andet problem med at klare tilværelsen	0		

DZ7381;Problem med adfærdsbetinget insomni hos barn	0		
DZ738A;Problem med væsentlige eller eksistentielle forhold	0		
DZ738B;Problem med manglende selvtillid	0		
DZ739;Problem med at klare tilværelsen UNS	0		
DZ74;Problemer i forbindelse med afhængighed af pleje	0		
DZ740;Problem med nedsat mobilitet	0		
DZ741;Problem med behov for personlig pleje	0		
DZ742;Problem med behov for hjælp i hjemmet	0		
DZ743;Problem med behov for konstant tilsyn	0		
DZ748;Andet problem i forbindelse med afhængighed af pleje	0		
DZ749;Problem i forbindelse med afhængighed af pleje UNS	0		
DZ749C;Causa socialis	0		
DZ75;Problemer i forbindelse med adgang til behandling og pleje	0		
DZ750;Problem med tilgængelighed af lægelig hjælp i hjemmet	0		
DZ751;Problem med ventetid til plejehjem	0		
DZ752;Problem med ventetid til undersøgelse eller behandling	0		
DZ753;Problem med manglende adgang eller tilgængelighed til sundhedsfaciliteter	0		
DZ754;Problem med manglende adgang til anden hjælpeindsats	0		
DZ755;Problem med manglende adgang til aflastningspleje	0		
DZ758;Andet problem med behandling eller pleje	0		
DZ759;Problem med behandling eller pleje UNS	0		
DZ76;Personer i kontakt med sundhedsvæsenet af andre årsager	0		
DZ760;Kontakt mhp. receptfornyelse	0		
DZ761;Kontakt mhp. helbredstilsyn eller pleje af hittebarn		1	
DZ762;Kontakt mhp. helbredstilsyn eller pleje af andet sundt barn	0		
DZ763;Rask ledsager	0		
DZ763B;Rask nyfødt som ledsager	0		
DZ764;Anden person i en af sundhedsvæsenets institutioner	0		
DZ765;Simulering	0		
DZ766;Kontakt afbrudt af patienten	0		
DZ766A;Kontakt afbrudt af patienten trods givet råd	0		
DZ768;Kontakt med sundhedsvæsenet under anden omstændighed	0		

Supplement 2 Nielsen et al. Mortality after paediatric emergency calls for patients with and without pre-existing comorbidity (2023)

DZ768A;Barns ophold på sygehus eller institution ifm. adoption	0		
DZ768B;Kontakt mhp. udlevering af journaloplysninger	0		
DZ768C;Barn eller ung indlagt begrundet i sociale forhold		1	
DZ768D;Rask forsøgsperson	0		
DZ768E;Kontakt pga. kønsidentitetsforhold	0		
DZ768E1;Kontakt pga. transkønnethed	0		
DZ768E2;Kontakt pga. kønsidentitetsforhold i barndommen	0		
DZ768E3;Kontakt pga. andre kønsidentitetsforhold	0		
DZ768E4;Kontakt pga. kønsidentitetsforhold UNS	0		
DZ768F;Kontakt mhp. samtale om pårørendes tilstand	0		
DZ768Z;Kontakt uden begrundelse i sygdom IKA	0		
DZ769;Person i kontakt med sundhedsvæsenet af årsag UNS	0		
DZ769A;Rask nyfødt	0		
DZ80;Familieanamnese med kræft	0		
DZ800;Familieanamnese med kræft i fordøjelsesorganer	0		
DZ801;Familieanamnese med kræft i bronkier eller lunger	0		
DZ802;Familieanamnese med kræft i anden del af luftvejene eller intratorakalt organ	0		
DZ803;Familieanamnese med kræft i mamma	0		
DZ804;Familieanamnese med kræft i kønsorganer	0		
DZ805;Familieanamnese med kræft i urinveje	0		
DZ806;Familieanamnese med leukæmi	0		
DZ807;Familieanamnese med kræft i lymfatisk eller bloddannende væv	0		
DZ808;Familieanamnese med kræft i andet organ eller system	0		
DZ809;Familieanamnese med kræft UNS	0		
DZ81;Familieanamnese med psykisk eller adfærdsmæssig sygdom	0		
DZ810;Familieanamnese med mental retardering	0		
DZ811;Familieanamnese med alkoholmisbrug	0		
DZ812;Familieanamnese med tobaksmisbrug	0		
DZ813;Familieanamnese med misbrug af anden psykoaktiv substans	0		
DZ814;Familieanamnese med andet stofmisbrug	0		
DZ818;Familieanamnese med anden psykisk eller adfærdsmæssig sygdom	0		
DZ82;Familieanamnese med visse kroniske og invaliderende sygdomme	0		

Supplement 2 Nielsen et al. Mortality after paediatric emergency calls for patients with and without pre-existing comorbidity (2023)

DZ820;Familieanamnese med epilepsi eller anden sygdom i nervesystemet	0		
DZ821;Familieanamnese med blindhed eller synsnedsættelse	0		
DZ822;Familieanamnese med døvhed eller hørenedsættelse	0		
DZ823;Familieanamnese med cerebrovaskulær sygdom	0		
DZ824;Familieanamnese med iskæmisk eller anden kredsløbssygdom	0		
DZ824A;Familieanamnese med arvelig disposition til hjertekarsygdom	0		
DZ824AA;Familieanamnese med langt QT-syndrom	0		
DZ825;Familieanamnese med astma eller anden kronisk sygdom i nedre luftveje	0		
DZ826;Familieanamnese med sygdom i bevægeapparatet	0		
DZ827;Familieanamnese med misdannelser eller kromosomabnormiteter	0		
DZ828;Familieanamnese med kronisk eller invaliderende sygdom IKA	0		
DZ83;Familieanamnese med andre specificerede sygdomme	0		
DZ830;Familieanamnese med HIV	0		
DZ831;Familieanamnese med anden infektiøs eller parasitær sygdom	0		
DZ832;Familieanamnese med sygdom i blod eller bloddannende organ eller immunsystem	0		
DZ832A;Familieanamnese med koagulationsdefekt	0		
DZ833;Familieanamnese med diabetes	0		
DZ834;Familieanamnese med anden endokrin, ernæringsbetinget eller metabolisk sygdom	0		
DZ835;Familieanamnese med sygdom i øjne eller ører	0		
DZ836;Familieanamnese med sygdom i åndedrætsorganerne	0		
DZ837;Familieanamnese med sygdom i fordøjelsesorgan	0		
DZ84;Familieanamnese med andre sygelige tilstande	0		
DZ840;Familieanamnese med sygdom i hud eller underhud	0		
DZ841;Familieanamnese med sygdom i nyre eller urinleder	0		
DZ842;Familieanamnese med anden sygdom i urin- eller kønsorganer	0		
DZ842A;Familiær disposition til sygdom i urin- eller kønsorgan	0		
DZ842A1;Familiær disposition til sygdom i prostata	0		
DZ842A1A;Familiær disposition til prostatakræft	0		
DZ843;Familieanamnese med indgifte	0		
DZ848;Familieanamnese med anden tilstand	0		
DZ848A;Familieanamnese med kendt genetisk defekt	0		
DZ848A1;Fam anamn. m hereditær non-polypøs kolorektal cancer (HNPCC)	0		

Supplement 2 Nielsen et al. Mortality after paediatric emergency calls for patients with and without pre-existing comorbidity (2023)

DZ848A2;Familieanamnese med hereditær bryst- og æggestokkræft (HBOC)	0		
DZ848B;Familieanamnese med allergi	0		
DZ85;Anamnese med kræft		1	
DZ850;Anamnese med kræft i fordøjelsesorganerne		1	
DZ850C;Anamnese med kræft i ventrikel		1	
DZ850D;Anamnese med kræft i tyktarm		1	
DZ850E;Anamnese med kræft i lever		1	
DZ850F;Anamnese med kræft i pancreas		1	
DZ850G;Anamnese med kræft i endetarm		1	
DZ851;Anamnese med kræft i luftrør, bronkier eller lunger		1	
DZ852;Anamnese med kræft i andet intratorakalt organ		1	
DZ853;Anamnese med kræft i mamma		1	
DZ854;Anamnese med kræft i kønsorganer		1	
DZ855;Anamnese med kræft i urinvejene		1	
DZ856;Anamnese med leukæmi		1	
DZ857;Anamnese med anden kræft i lymfatisk, bloddannende eller relateret væv		1	
DZ858;Anamnese med kræft i andet organ eller organsystem		1	
DZ859;Anamnese med kræft UNS		1	
DZ86;Anamnese med visse andre sygdomme	0		
DZ860;Anamnese med anden tumorsygdom	0		
DZ861;Anamnese med infektiøs eller parasitær sygdom	0		
DZ861A;Anamnese med COVID-19	0		
DZ862;Anamnese med sygdom i blod, bloddannende organ eller immunsystemet	0		
DZ862A;Asymptomatisk bærer af hæmofili	0		
DZ862A1;Asymptomatisk bærer af hæmofili A	0		
DZ862A2;Asymptomatisk bærer af hæmofili B	0		
DZ863;Anamnese med endokrin, ernæringsbetinget eller metabolisk sygdom		1	
DZ863D;Anamnese med type 2-diabetes		1	
DZ864;Anamnese med misbrug af psykoaktive stoffer	0		
DZ865;Anamnese med psykisk eller adfærdsmæssig forstyrrelse	0		
DZ865A;Anamnese med ubehandlet psykose		1	
DZ865AA;Varighed af ubehandlet psykose (VUP) < 3 mdr.		1	

Supplement 2 Nielsen et al. Mortality after paediatric emergency calls for patients with and without pre-existing comorbidity (2023)

DZ865AB;Varighed af ubehandlet psykose (VUP) 3-6 mdr.		1	
DZ865AC;Varighed af ubehandlet psykose > 6 mdr.		1	
DZ865B;Anamnese med skizofreni		1	
DZ866;Anamnese med sygdom i nervesystemet eller sanseorgan	0		
DZ867;Anamnese med kredsløbssygdom	0		
DZ867B;Anamnese med emboli eller trombose	0		
DZ87;Anamnese med andre sygdomme og tilstande	0		
DZ870;Anamnese med sygdom i luftvejene	0		
DZ871;Anamnese med sygdom i fordøjelsesorgan	0		
DZ872;Anamnese med sygdom i hud eller underhud	0		
DZ873;Anamnese med sygdom i knogler, muskler eller bindevæv	0		
DZ874;Anamnese med sygdom i urin- eller kønsorgan	0		
DZ875;Anamnese med komplikation til graviditet, fødsel eller barselsperiode		1	
DZ875A;Anamnese med cervixinsufficiens	0		
DZ875B;Anamnese med tidligere anlagt abdominal cerclage	0		
DZ875C;Anamnese med anal sphincterruptur	0		
DZ875D;Anamnese med sædefødsel	0		
DZ875E;Anamnese med ammeproblemer	0		
DZ876;Anamnese med tilstand opstået i den perinatale periode		1	
DZ876A;Anamnese med immaturitet		1	
DZ876B;Anamnese med præmaturitet		1	
DZ876C;Anamnese med asfyksi ved fødslen		1	
DZ877;Anamnese med medfødt misdannelse eller kromosomabnormitet		1	
DZ878;Anamnese med anden tilstand	0		
DZ879;Anamnese med sygdom UNS	0		
DZ88;Anamnese med allergi over for lægemidler og biologiske substanser		1	
DZ880;Anamnese med allergi over for penicillin		1	
DZ881;Anamnese med allergi over for andre antibiotika		1	
DZ882;Anamnese med allergi over for sulfonamider		1	
DZ883;Anamnese med allergi over for andre anti-infektionsmidler		1	
DZ884;Anamnese med allergi over for bedøvelsesmiddel		1	
DZ885;Anamnese med allergi over for narkotisk middel		1	

DZ886;Anamnese med allergi over for smertestillende lægemiddel		1	
DZ887;Anamnese med allergi over for serum eller vaccine		1	
DZ888;Anamnese med allergi over for andet lægemiddel eller biologisk substans		1	
DZ889;Anamnese med allergi over for lægemiddel eller biologisk substans UNS		1	
DZ89;Erhvervet mangel af ekstremitet	0		
DZ890;Enkeltsidig erhvervet mangel af finger eller fingre	0		
DZ891;Erhvervet mangel af hånd og håndled	0		
DZ892;Erhvervet mangel af arm oven for håndled	0		
DZ893;Erhvervet mangel af begge arme	0		
DZ894;Erhvervet mangel af fod og ankel	0		
DZ895;Erhvervet mangel af underekstremitet i højde med eller under knæet	0		
DZ895B;Exarticulatio genus facta	0		
DZ896;Erhvervet mangel af underekstremitet oven for knæet	0		
DZ897;Erhvervet mangel af begge underekstremiteter		1	
DZ898;Erhvervet mangel af både over- og underekstremiteter		1	
DZ899;Erhvervet mangel af ekstremitet UNS	0		
DZ90;Erhvervet mangel af organer IKA		1	
DZ900;Erhvervet mangel af del af hovedet eller hals		1	
DZ900A;Erhvervet mangel af larynx		1	
DZ900B;Erhvervet mangel af skjoldbruskkirtlen		1	
DZ901;Erhvervet mangel af bryst(er)		1	
DZ901B;Mastectomia bilateralis facta		1	
DZ901C;Lumpectomia facta		1	
DZ901D;Lumpectomia bilateralis facta		1	
DZ901E;Reductio mammae facta		1	
DZ901F;Reductio mammae bilateralis facta		1	
DZ901G;Mastectomia unilateralis facta		1	
DZ902;Erhvervet mangel af lunge		1	
DZ902B;Erhvervet mangel af del af lunge		1	
DZ903;Erhvervet mangel af del af mavesæk		1	
DZ904;Erhvervet mangel af anden del af fordøjelseskanalen		1	
DZ904A;Erhvervet mangel af del af colon		1	

Supplement 2 Nielsen et al. Mortality after paediatric emergency calls for patients with and without pre-existing comorbidity (2023)

DZ904B;Erhvervet mangel af colon		1	
DZ904C;Erhvervet mangel af endetarm		1	
DZ904D;Erhvervet mangel af galdeblære		1	
DZ904E;Erhvervet mangel af bugspytkirtlen		1	
DZ905;Erhvervet mangel af nyre		1	
DZ905B;Erhvervet mangel af begge nyrer		1	
DZ906;Erhvervet mangel af andet urinvejsorgan		1	
DZ906A;Erhvervet mangel af prostata		1	
DZ906B;Erhvervet mangel af del af urinblæren		1	
DZ907;Erhvervet mangel af kønsorgan		1	
DZ907A;Erhvervet mangel af livmoderen		1	
DZ907B;Erhvervet mangel af æggestok		1	
DZ907B1;Erhvervet mangel af begge æggestokke		1	
DZ907C;Erhvervet mangel af testikel		1	
DZ907C1;Erhvervet mangel af begge testikler		1	
DZ907D;Følger efter omskæring (findes ikke i SKS)		1	
DZ908;Erhvervet mangel af andet organ		1	
DZ908A;Erhvervet mangel af hypofysen		1	
DZ91;Personlige risikofaktorer IKA	0		
DZ910;Anamnese med allergi over for andet end lægemidler og biologiske substanser	0		
DZ911;Anamnese med manglende pasning af medicinsk behandling eller foranstaltning	0		
DZ911A;Komplekst compliancesvigt	0		
DZ912;Anamnese med dårlig personlig hygiejne	0		
DZ913;Anamnese med dårlig søvnhygiejne	0		
DZ914;Anamnese med psykisk traume IKA		1	
DZ914A;Tidligere udsættelse for særligt voldsomme oplevelser		1	
DZ915;Anamnese med selvpåført skade		1	
DZ915A;Anamnese med selvmordsforsøg		1	
DZ916;Anamnese med andet fysisk traume	0		
DZ917;Anamnese med kvindelig omskæring (FGM)	0		
DZ918;Anamnese med anden risikofaktor IKA	0		
DZ918A;Anamnese med sen spontan descensus testis	0		

Supplement 2 Nielsen et al. Mortality after paediatric emergency calls for patients with and without pre-existing comorbidity (2023)

DZ918B;Behov for indlæggelseskrævende omlægning af antiepileptisk behandling	0		
DZ918C;Anamnese med genetisk verificeret risikofaktor eller sygdomsrisiko	0		
DZ918D;Følger efter mandlig omskæring	0		
DZ918D1;Følger efter rutinemæssig eller rituel mandlig omskæring	0		
DZ918D2;Følger efter mandlig omskæring på lægefaglig indikation	0		
DZ92;Anamnese med tidligere behandling	0		
DZ920;Anamnese med graviditetsforebyggelse	0		
DZ921;Anamnese med langtidsbehandling med antikoagulantia		1	
DZ922;Anamnese med langtidsanvendelse af andet medikament		1	
DZ923;Anamnese med strålebehandling		1	
DZ924;Anamnese med større operation IKA	0		
DZ925;Anamnese med genoptræning	0		
DZ926;Anamnese med kemoterapi for kræft		1	
DZ928;Anamnese med anden medicinsk behandling	0		
DZ928A;Anamnese med behandlet syfilis	0		
DZ928B;Anamnese med behandlet non-descensus testis	0		
DZ928C;Anamnese med konisation af livmoderhals	0		
DZ928D;Anamnese m trakelektomi	0		
DZ928E;Anamnese med operation for stress urininkontinens	0		
DZ928F;Anamnese med operation for uterovaginal prolaps	0		
DZ928F1;Anamnese med operation for uterovaginal prolaps i samme kompartment	0		
DZ929;Anamnese med behandling UNS	0		
DZ93;Tilstand med kunstig legemsåbning		1	
DZ930;Tilstand med trakeostomi		1	
DZ931;Tilstand med gastrostomi		1	
DZ932;Tilstand med ileostomi		1	
DZ933;Tilstand med kolostomi		1	
DZ934;Tilstand med anden kunstig åbning på mave-tarm-kanalen		1	
DZ935;Tilstand med cystostomi		1	
DZ936;Tilstand med anden kunstig åbning på urinvejene		1	
DZ936A;Tilstand med nefrostomi		1	
DZ936B;Tilstand med ureterostomi		1	

DZ936C;Tilstand med uretrostomi		1	
DZ938;Tilstand med anden kunstig legemsåbning		1	
DZ939;Tilstand med kunstig legemsåbning UNS		1	
DZ94;Tilstand med transplanteret organ eller væv		1	
DZ940;Nyretransplanteret		1	
DZ941;Hjertetransplanteret		1	
DZ942;Lungetransplanteret		1	
DZ943;Hjerte-lunge-transplanteret		1	
DZ944;Levertransplanteret		1	
DZ945;Hudtransplanteret		1	
DZ946;Knogletransplanteret		1	
DZ947;Hornhindetransplanteret		1	
DZ948;Tilstand med andet organ- eller vævstransplantat		1	
DZ948A;Pancreastransplanteret		1	
DZ948B;Tyndtarmstransplanteret		1	
DZ948C;Knoglemarvstransplanteret		1	
DZ948C1;Knoglemarvstransplanteret med allogen knoglemarv		1	
DZ948C2;Knoglemarvstransplanteret med autolog knoglemarv		1	
DZ949;Tilstand med transplanteret organ eller væv UNS		1	
DZ95;Tilstand med hjerte- eller karimplantater og -transplantater		1	
DZ950;Tilstand med pacemaker, kardioverter-defibrillator (ICD) eller andet implanteret elektronisk kardielt hjælpemiddel		1	
DZ950B;Tilstand med biventrikulær pacemaker		1	
DZ950C;Tilstand med kardioverter-defibrillator (ICD)		1	
DZ950D;Tilstand med biventrikulær kardioverter-defibrillator (ICD)		1	
DZ950L;Tilstand med loop recorder		1	
DZ950P;Tilstand med pacemaker UNS		1	
DZ951;Tilstand med aortokoronar-bypass transplantat		1	
DZ952;Tilstand med kunstig hjerteklap		1	
DZ953;Tilstand med heterogen hjerteklap		1	
DZ954;Tilstand med anden hjerteklaperstatning		1	
DZ955;Tilstand med angioplastisk koronarimplantat eller -transplantat		1	
DZ958;Tilstand med andet implantat eller transplantat i hjerte eller kar		1	

Supplement 2 Nielsen et al. Mortality after paediatric emergency calls for patients with and without pre-existing comorbidity (2023)

DZ959;Tilstand med implantat eller transplantat i hjerte eller kar UNS		1	
DZ96;Tilstand med andre funktionelle implantater		1	
DZ960;Tilstand med urogenitalt implantat		1	
DZ961;Tilstand med intraokulær linse		1	
DZ962;Tilstand med otologisk eller audiologisk implantat		1	
DZ962A;Tilstand med audiologisk implantat		1	
DZ962B;Tilstand med otologisk implantat		1	
DZ963;Tilstand med kunstig strube		1	
DZ964;Tilstand med endokrint implantat		1	
DZ965;Tilstand med tandrods- eller kæbeimplantat	0		
DZ966;Tilstand med ortopædisk ledimplantat		1	
DZ966A;Tilstand med hofteledsprotese		1	
DZ966B;Tilstand med knæledsprotese		1	
DZ966C;Tilstand med skulderledsprotese		1	
DZ966D;Tilstand med fingerledsprotese		1	
DZ967;Tilstand med andet knogle- eller seneimplantat		1	
DZ967A;Tilstand med knogleimplantat		1	
DZ967B;Tilstand med seneimplantat		1	
DZ968;Tilstand med andet funktionelt implantat		1	
DZ968A;Tilstand med kunstig næse		1	
DZ968B;Tilstand med kunstigt øre		1	
DZ968C;Tilstand med kunstig næse og øje		1	
DZ968D;Tilstand med kunstigt mellemansigt		1	
DZ968E;Tilstand med corpus vitreum substitut		1	
DZ968F;Tilstand med bløddelsimplantat eller konturprotese		1	
DZ969;Tilstand med funktionelt implantat UNS		1	
DZ97;Tilstand med andre hjælpemidler	0		
DZ970;Tilstand med kunstigt øje	0		
DZ971;Tilstand med kunstig arm eller ben	0		
DZ971A;Tilstand med armprotese	0		
DZ971B;Tilstand med benprotese	0		
DZ972;Anvender tandprotese	0		

DZ973;Anvender briller eller kontaktlinser	0		
DZ973A;Anvender briller	0		
DZ973B;Anvender kontaktlinser	0		
DZ974;Anvender eksternt høreapparat	0		
DZ975;Anvender intrauterin antikonception	0		
DZ978;Tilstand med andet hjælpemiddel		1	
DZ978A;Tilstand med kateter a demeure		1	
DZ98;Andre postoperative tilstande		1	
DZ980;Tilstand med intestinal bypass eller anastomose		1	
DZ980A;Tilstand med intestinal anastomose		1	
DZ980A1;Tilstand med ileoanal pouch		1	
DZ980B;Tilstand med intestinal bypass		1	
DZ980C;Tilstand med gastric bypass		1	
DZ980D;Tilstand med gastric banding		1	
DZ980E;Tilstand med gastric sleeve resection		1	
DZ980F;Tilstand med åben Petersens defekt efter gastric bypass		1	
DZ980G;Tilstand med åben mesenteriel defekt efter gastric bypass		1	
DZ981;Tilstand med artrodese		1	
DZ982;Tilstand med cerebrospinalt dræn		1	
DZ988;Anden postoperativ tilstand		1	
DZ99;Afhængighed af hjælpemidler og apparater IKA		1	
DZ990;Afhængig af aspirator		1	
DZ991;Afhængig af respirator		1	
DZ991A;Kontakt pga. mangel på hjælpere til hjemmerespiratorbehandling		1	
DZ992;Afhængig af renal dialyse		1	
DZ993;Afhængig af kørestol		1	
DZ994;Afhængig af kunstigt hjerte		1	
DZ998;Afhængighed af andet hjælpemiddel eller apparat		1	
DZ998A;Afhængig af hjælpemiddel ved søvnapnø		1	
DZ998B;Afhængig af hjælpemiddel til gang		1	
DZ998C;Afhængig af hjælpemiddel til kommunikation		1	
DZ999;Afhængighed af hjælpemiddel eller apparat UNS		1	

Supplement 2 Nielsen et al. Mortality after paediatric emergency calls for patients with and without pre-existing comorbidity (2023)

DU0000;før graviditet	0			
DU01D0;1 graviditetsuge eller tidligere	0			
DU01D1;1 graviditetsuge + 1 dag	0			
DU01D2;1 graviditetsuge + 2 dage	0			
DU01D3;1 graviditetsuge + 3 dage	0			
DU01D4;1 graviditetsuge + 4 dage	0			
DU01D5;1 graviditetsuge + 5 dage	0			
DU01D6;1 graviditetsuge + 6 dage	0			
DU01DX;1 graviditetsuge, skønnet, termin usikker	0			
DU02D0;2 graviditetsuger	0			
DU02D1;2 graviditetsuger + 1 dag	0			
DU02D2;2 graviditetsuger + 2 dage	0			
DU02D3;2 graviditetsuger + 3 dage	0			
DU02D4;2 graviditetsuger + 4 dage	0			
DU02D5;2 graviditetsuger + 5 dage	0			
DU02D6;2 graviditetsuger + 6 dage	0			
DU02DX;2 graviditetsuger, skønnet, termin usikker	0			
DU03D0;3 graviditetsuger	0			
DU03D1;3 graviditetsuger + 1 dag	0			
DU03D2;3 graviditetsuger + 2 dage	0			
DU03D3;3 graviditetsuger + 3 dage	0			
DU03D4;3 graviditetsuger + 4 dage	0			
DU03D5;3 graviditetsuger + 5 dage	0			
DU03D6;3 graviditetsuger + 6 dage	0			
DU03DX;3 graviditetsuger, skønnet, termin usikker	0			
DU04D0;4 graviditetsuger	0			
DU04D1;4 graviditetsuger + 1 dag	0			
DU04D2;4 graviditetsuger + 2 dage	0			
DU04D3;4 graviditetsuger + 3 dage	0			
DU04D4;4 graviditetsuger + 4 dage	0			
DU04D5;4 graviditetsuger + 5 dage	0			
DU04D6;4 graviditetsuger + 6 dage	0			

DU04DX;4 graviditetsuger, skønnet, termin usikker	0			
DU05D0;5 graviditetsuger	0			
DU05D1;5 graviditetsuger + 1 dag	0			
DU05D2;5 graviditetsuger + 2 dage	0			
DU05D3;5 graviditetsuger + 3 dage	0			
DU05D4;5 graviditetsuger + 4 dage	0			
DU05D5;5 graviditetsuger + 5 dage	0			
DU05D6;5 graviditetsuger + 6 dage	0			
DU05DX;5 graviditetsuger, skønnet, termin usikker	0			
DU06D0;6 graviditetsuger	0			
DU06D1;6 graviditetsuger + 1 dag	0			
DU06D2;6 graviditetsuger + 2 dage	0			
DU06D3;6 graviditetsuger + 3 dage	0			
DU06D4;6 graviditetsuger + 4 dage	0			
DU06D5;6 graviditetsuger + 5 dage	0			
DU06D6;6 graviditetsuger + 6 dage	0			
DU06DX;6 graviditetsuger, skønnet, termin usikker	0			
DU07D0;7 graviditetsuger	0			
DU07D1;7 graviditetsuger + 1 dag	0			
DU07D2;7 graviditetsuger + 2 dage	0			
DU07D3;7 graviditetsuger + 3 dage	0			
DU07D4;7 graviditetsuger + 4 dage	0			
DU07D5;7 graviditetsuger + 5 dage	0			
DU07D6;7 graviditetsuger + 6 dage	0			
DU07DX;7 graviditetsuger, skønnet, termin usikker	0			
DU08D0;8 graviditetsuger	0			
DU08D1;8 graviditetsuger + 1 dag	0			
DU08D2;8 graviditetsuger + 2 dage	0			
DU08D3;8 graviditetsuger + 3 dage	0			
DU08D4;8 graviditetsuger + 4 dage	0			
DU08D5;8 graviditetsuger + 5 dage	0			
DU08D6;8 graviditetsuger + 6 dage	0			

DU08DX;8 graviditetsuger, skønnet, termin usikker	0		
DU09D0;9 graviditetsuger	0		
DU09D1;9 graviditetsuger + 1 dag	0		
DU09D2;9 graviditetsuger + 2 dage	0		
DU09D3;9 graviditetsuger + 3 dage	0		
DU09D4;9 graviditetsuger + 4 dage	0		
DU09D5;9 graviditetsuger + 5 dage	0		
DU09D6;9 graviditetsuger + 6 dage	0		
DU09DX;9 graviditetsuger, skønnet, termin usikker	0		
DU10D0;10 graviditetsuger	0		
DU10D1;10 graviditetsuger + 1 dag	0		
DU10D2;10 graviditetsuger + 2 dage	0		
DU10D3;10 graviditetsuger + 3 dage	0		
DU10D4;10 graviditetsuger + 4 dage	0		
DU10D5;10 graviditetsuger + 5 dage	0		
DU10D6;10 graviditetsuger + 6 dage	0		
DU10DX;10 graviditetsuger, skønnet, termin usikker	0		
DU11D0;11 graviditetsuger	0		
DU11D1;11 graviditetsuger + 1 dag	0		
DU11D2;11 graviditetsuger + 2 dage	0		
DU11D3;11 graviditetsuger + 3 dage	0		
DU11D4;11 graviditetsuger + 4 dage	0		
DU11D5;11 graviditetsuger + 5 dage	0		
DU11D6;11 graviditetsuger + 6 dage	0		
DU11DX;11 graviditetsuger, skønnet, termin usikker	0		
DU12D0;12 graviditetsuger	0		
DU12D1;12 graviditetsuger + 1 dag	0		
DU12D2;12 graviditetsuger + 2 dage	0		
DU12D3;12 graviditetsuger + 3 dage	0		
DU12D4;12 graviditetsuger + 4 dage	0		
DU12D5;12 graviditetsuger + 5 dage	0		
DU12D6;12 graviditetsuger + 6 dage	0		

DU12DX;12 graviditetsuger, skønnet, termin usikker	0		
DU13D0;13 graviditetsuger	0		
DU13D1;13 graviditetsuger + 1 dag	0		
DU13D2;13 graviditetsuger + 2 dage	0		
DU13D3;13 graviditetsuger + 3 dage	0		
DU13D4;13 graviditetsuger + 4 dage	0		
DU13D5;13 graviditetsuger + 5 dage	0		
DU13D6;13 graviditetsuger + 6 dage	0		
DU13DX;13 graviditetsuger, skønnet, termin usikker	0		
DU14D0;14 graviditetsuger	0		
DU14D1;14 graviditetsuger + 1 dag	0		
DU14D2;14 graviditetsuger + 2 dage	0		
DU14D3;14 graviditetsuger + 3 dage	0		
DU14D4;14 graviditetsuger + 4 dage	0		
DU14D5;14 graviditetsuger + 5 dage	0		
DU14D6;14 graviditetsuger + 6 dage	0		
DU14DX;14 graviditetsuger, skønnet, termin usikker	0		
DU15D0;15 graviditetsuger	0		
DU15D1;15 graviditetsuger + 1 dag	0		
DU15D2;15 graviditetsuger + 2 dage	0		
DU15D3;15 graviditetsuger + 3 dage	0		
DU15D4;15 graviditetsuger + 4 dage	0		
DU15D5;15 graviditetsuger + 5 dage	0		
DU15D6;15 graviditetsuger + 6 dage	0		
DU15DX;15 graviditetsuger, skønnet, termin usikker	0		
DU16D0;16 graviditetsuger	0		
DU16D1;16 graviditetsuger + 1 dag	0		
DU16D2;16 graviditetsuger + 2 dage	0		
DU16D3;16 graviditetsuger + 3 dage	0		
DU16D4;16 graviditetsuger + 4 dage	0		
DU16D5;16 graviditetsuger + 5 dage	0		
DU16D6;16 graviditetsuger + 6 dage	0		

DU16DX;16 graviditetsuger, skønnet, termin usikker	0		
DU17D0;17 graviditetsuger	0		
DU17D1;17 graviditetsuger + 1 dag	0		
DU17D2;17 graviditetsuger + 2 dage	0		
DU17D3;17 graviditetsuger + 3 dage	0		
DU17D4;17 graviditetsuger + 4 dage	0		
DU17D5;17 graviditetsuger + 5 dage	0		
DU17D6;17 graviditetsuger + 6 dage	0		
DU17DX;17 graviditetsuger, skønnet, termin usikker	0		
DU18D0;18 graviditetsuger	0		
DU18D1;18 graviditetsuger + 1 dag	0		
DU18D2;18 graviditetsuger + 2 dage	0		
DU18D3;18 graviditetsuger + 3 dage	0		
DU18D4;18 graviditetsuger + 4 dage	0		
DU18D5;18 graviditetsuger + 5 dage	0		
DU18D6;18 graviditetsuger + 6 dage	0		
DU18DX;18 graviditetsuger, skønnet, termin usikker	0		
DU19D0;19 graviditetsuger	0		
DU19D1;19 graviditetsuger + 1 dag	0		
DU19D2;19 graviditetsuger + 2 dage	0		
DU19D3;19 graviditetsuger + 3 dage	0		
DU19D4;19 graviditetsuger + 4 dage	0		
DU19D5;19 graviditetsuger + 5 dage	0		
DU19D6;19 graviditetsuger + 6 dage	0		
DU19DX;19 graviditetsuger, skønnet, termin usikker	0		
DU20D0;20 graviditetsuger	0		
DU20D1;20 graviditetsuger + 1 dag	0		
DU20D2;20 graviditetsuger + 2 dage	0		
DU20D3;20 graviditetsuger + 3 dage	0		
DU20D4;20 graviditetsuger + 4 dage	0		
DU20D5;20 graviditetsuger + 5 dage	0		
DU20D6;20 graviditetsuger + 6 dage	0		

DU20DX;20 graviditetsuger, skønnet, termin usikker	0		
DU21D0;21 graviditetsuger	0		
DU21D1;21 graviditetsuger + 1 dag	0		
DU21D2;21 graviditetsuger + 2 dage	0		
DU21D3;21 graviditetsuger + 3 dage	0		
DU21D4;21 graviditetsuger + 4 dage	0		
DU21D5;21 graviditetsuger + 5 dage	0		
DU21D6;21 graviditetsuger + 6 dage	0		
DU21DX;21 graviditetsuger, skønnet, termin usikker	0		
DU22D0;22 graviditetsuger	0		
DU22D1;22 graviditetsuger + 1 dag	0		
DU22D2;22 graviditetsuger + 2 dage	0		
DU22D3;22 graviditetsuger + 3 dage	0		
DU22D4;22 graviditetsuger + 4 dage	0		
DU22D5;22 graviditetsuger + 5 dage	0		
DU22D6;22 graviditetsuger + 6 dage	0		
DU22DX;22 graviditetsuger, skønnet, termin usikker	0		
DU23D0;23 graviditetsuger	0		
DU23D1;23 graviditetsuger + 1 dag	0		
DU23D2;23 graviditetsuger + 2 dage	0		
DU23D3;23 graviditetsuger + 3 dage	0		
DU23D4;23 graviditetsuger + 4 dage	0		
DU23D5;23 graviditetsuger + 5 dage	0		
DU23D6;23 graviditetsuger + 6 dage	0		
DU23DX;23 graviditetsuger, skønnet, termin usikker	0		
DU24D0;24 graviditetsuger	0		
DU24D1;24 graviditetsuger + 1 dag	0		
DU24D2;24 graviditetsuger + 2 dage	0		
DU24D3;24 graviditetsuger + 3 dage	0		
DU24D4;24 graviditetsuger + 4 dage	0		
DU24D5;24 graviditetsuger + 5 dage	0		
DU24D6;24 graviditetsuger + 6 dage	0		

DU24DX;24 graviditetsuger, skønnet, termin usikker	0		
DU25D0;25 graviditetsuger	0		
DU25D1;25 graviditetsuger + 1 dag	0		
DU25D2;25 graviditetsuger + 2 dage	0		
DU25D3;25 graviditetsuger + 3 dage	0		
DU25D4;25 graviditetsuger + 4 dage	0		
DU25D5;25 graviditetsuger + 5 dage	0		
DU25D6;25 graviditetsuger + 6 dage	0		
DU25DX;25 graviditetsuger, skønnet, termin usikker	0		
DU26D0;26 graviditetsuger	0		
DU26D1;26 graviditetsuger + 1 dag	0		
DU26D2;26 graviditetsuger + 2 dage	0		
DU26D3;26 graviditetsuger + 3 dage	0		
DU26D4;26 graviditetsuger + 4 dage	0		
DU26D5;26 graviditetsuger + 5 dage	0		
DU26D6;26 graviditetsuger + 6 dage	0		
DU26DX;26 graviditetsuger, skønnet, termin usikker	0		
DU27D0;27 graviditetsuger	0		
DU27D1;27 graviditetsuger + 1 dag	0		
DU27D2;27 graviditetsuger + 2 dage	0		
DU27D3;27 graviditetsuger + 3 dage	0		
DU27D4;27 graviditetsuger + 4 dage	0		
DU27D5;27 graviditetsuger + 5 dage	0		
DU27D6;27 graviditetsuger + 6 dage	0		
DU27DX;27 graviditetsuger, skønnet, termin usikker	0		
DU28D0;28 graviditetsuger	0		
DU28D1;28 graviditetsuger + 1 dag	0		
DU28D2;28 graviditetsuger + 2 dage	0		
DU28D3;28 graviditetsuger + 3 dage	0		
DU28D4;28 graviditetsuger + 4 dage	0		
DU28D5;28 graviditetsuger + 5 dage	0		
DU28D6;28 graviditetsuger + 6 dage	0		

DU28DX;28 graviditetsuger, skønnet, termin usikker	0		
DU29D0;29 graviditetsuger	0		
DU29D1;29 graviditetsuger + 1 dag	0		
DU29D2;29 graviditetsuger + 2 dage	0		
DU29D3;29 graviditetsuger + 3 dage	0		
DU29D4;29 graviditetsuger + 4 dage	0		
DU29D5;29 graviditetsuger + 5 dage	0		
DU29D6;29 graviditetsuger + 6 dage	0		
DU29DX;29 graviditetsuger, skønnet, termin usikker	0		
DU30D0;30 graviditetsuger	0		
DU30D1;30 graviditetsuger + 1 dag	0		
DU30D2;30 graviditetsuger + 2 dage	0		
DU30D3;30 graviditetsuger + 3 dage	0		
DU30D4;30 graviditetsuger + 4 dage	0		
DU30D5;30 graviditetsuger + 5 dage	0		
DU30D6;30 graviditetsuger + 6 dage	0		
DU30DX;30 graviditetsuger, skønnet, termin usikker	0		
DU31D0;31 graviditetsuger	0		
DU31D1;31 graviditetsuger + 1 dag	0		
DU31D2;31 graviditetsuger + 2 dage	0		
DU31D3;31 graviditetsuger + 3 dage	0		
DU31D4;31 graviditetsuger + 4 dage	0		
DU31D5;31 graviditetsuger + 5 dage	0		
DU31D6;31 graviditetsuger + 6 dage	0		
DU31DX;31 graviditetsuger, skønnet, termin usikker	0		
DU32D0;32 graviditetsuger	0		
DU32D1;32 graviditetsuger + 1 dag	0		
DU32D2;32 graviditetsuger + 2 dage	0		
DU32D3;32 graviditetsuger + 3 dage	0		
DU32D4;32 graviditetsuger + 4 dage	0		
DU32D5;32 graviditetsuger + 5 dage	0		
DU32D6;32 graviditetsuger + 6 dage	0		

DU32DX;32 graviditetsuger, skønnet, termin usikker	0		
DU33D0;33 graviditetsuger	0		
DU33D1;33 graviditetsuger + 1 dag	0		
DU33D2;33 graviditetsuger + 2 dage	0		
DU33D3;33 graviditetsuger + 3 dage	0		
DU33D4;33 graviditetsuger + 4 dage	0		
DU33D5;33 graviditetsuger + 5 dage	0		
DU33D6;33 graviditetsuger + 6 dage	0		
DU33DX;33 graviditetsuger, skønnet, termin usikker	0		
DU34D0;34 graviditetsuger	0		
DU34D1;34 graviditetsuger + 1 dag	0		
DU34D2;34 graviditetsuger + 2 dage	0		
DU34D3;34 graviditetsuger + 3 dage	0		
DU34D4;34 graviditetsuger + 4 dage	0		
DU34D5;34 graviditetsuger + 5 dage	0		
DU34D6;34 graviditetsuger + 6 dage	0		
DU34DX;34 graviditetsuger, skønnet, termin usikker	0		
DU35D0;35 graviditetsuger	0		
DU35D1;35 graviditetsuger + 1 dag	0		
DU35D2;35 graviditetsuger + 2 dage	0		
DU35D3;35 graviditetsuger + 3 dage	0		
DU35D4;35 graviditetsuger + 4 dage	0		
DU35D5;35 graviditetsuger + 5 dage	0		
DU35D6;35 graviditetsuger + 6 dage	0		
DU35DX;35 graviditetsuger, skønnet, termin usikker	0		
DU36D0;36 graviditetsuger	0		
DU36D1;36 graviditetsuger + 1 dag	0		
DU36D2;36 graviditetsuger + 2 dage	0		
DU36D3;36 graviditetsuger + 3 dage	0		
DU36D4;36 graviditetsuger + 4 dage	0		
DU36D5;36 graviditetsuger + 5 dage	0		
DU36D6;36 graviditetsuger + 6 dage	0		

DU36DX;36 graviditetsuger, skønnet, termin usikker	0		
DU37D0;37 graviditetsuger	0		
DU37D1;37 graviditetsuger + 1 dag	0		
DU37D2;37 graviditetsuger + 2 dage	0		
DU37D3;37 graviditetsuger + 3 dage	0		
DU37D4;37 graviditetsuger + 4 dage	0		
DU37D5;37 graviditetsuger + 5 dage	0		
DU37D6;37 graviditetsuger + 6 dage	0		
DU37DX;37 graviditetsuger, skønnet, termin usikker	0		
DU38D0;38 graviditetsuger	0		
DU38D1;38 graviditetsuger + 1 dag	0		
DU38D2;38 graviditetsuger + 2 dage	0		
DU38D3;38 graviditetsuger + 3 dage	0		
DU38D4;38 graviditetsuger + 4 dage	0		
DU38D5;38 graviditetsuger + 5 dage	0		
DU38D6;38 graviditetsuger + 6 dage	0		
DU38DX;38 graviditetsuger, skønnet, termin usikker	0		
DU39D0;39 graviditetsuger	0		
DU39D1;39 graviditetsuger + 1 dag	0		
DU39D2;39 graviditetsuger + 2 dage	0		
DU39D3;39 graviditetsuger + 3 dage	0		
DU39D4;39 graviditetsuger + 4 dage	0		
DU39D5;39 graviditetsuger + 5 dage	0		
DU39D6;39 graviditetsuger + 6 dage	0		
DU39DX;39 graviditetsuger, skønnet, termin usikker	0		
DU40D0;40 graviditetsuger	0		
DU40D1;40 graviditetsuger + 1 dag	0		
DU40D2;40 graviditetsuger + 2 dage	0		
DU40D3;40 graviditetsuger + 3 dage	0		
DU40D4;40 graviditetsuger + 4 dage	0		
DU40D5;40 graviditetsuger + 5 dage	0		
DU40D6;40 graviditetsuger + 6 dage	0		

DU40DX;40 graviditetsuger, skønnet, termin usikker	0		
DU41D0;41 graviditetsuger	0		
DU41D1;41 graviditetsuger + 1 dag	0		
DU41D2;41 graviditetsuger + 2 dage	0		
DU41D3;41 graviditetsuger + 3 dage	0		
DU41D4;41 graviditetsuger + 4 dage	0		
DU41D5;41 graviditetsuger + 5 dage	0		
DU41D6;41 graviditetsuger + 6 dage	0		
DU41DX;41 graviditetsuger, skønnet, termin usikker	0		
DU42D0;42 graviditetsuger	0		
DU42D1;42 graviditetsuger + 1 dag	0		
DU42D2;42 graviditetsuger + 2 dage	0		
DU42D3;42 graviditetsuger + 3 dage	0		
DU42D4;42 graviditetsuger + 4 dage	0		
DU42D5;42 graviditetsuger + 5 dage	0		
DU42D6;42 graviditetsuger + 6 dage	0		
DU42DX;42 graviditetsuger, skønnet, termin usikker	0		
DU43D0;43 graviditetsuger	0		
DU43D1;43 graviditetsuger + 1 dag	0		
DU43D2;43 graviditetsuger + 2 dage	0		
DU43D3;43 graviditetsuger + 3 dage	0		
DU43D4;43 graviditetsuger + 4 dage	0		
DU43D5;43 graviditetsuger + 5 dage	0		
DU43D6;43 graviditetsuger + 6 dage	0		
DU43DX;43 graviditetsuger, skønnet, termin usikker	0		
DU44D0;44 graviditetsuger	0		
DU44D1;44 graviditetsuger + 1 dag	0		
DU44D2;44 graviditetsuger + 2 dage	0		
DU44D3;44 graviditetsuger + 3 dage	0		
DU44D4;44 graviditetsuger + 4 dage	0		
DU44D5;44 graviditetsuger + 5 dage	0		
DU44D6;44 graviditetsuger + 6 dage	0		

Supplement 2 Nielsen et al. Mortality after paediatric emergency calls for patients with and without pre-existing comorbidity (2023)

DU44DX;44 graviditetsuger, skønnet, termin usikker	0			
DU45D0;45 graviditetsuger eller derover	0			
DU99DX;Graviditetsuge ikke kendt, termin usikker	0			
DU99DY;efter fødslen (post partum)	0			
DVRA01;Legemsvægt (kg)	0			
DVRA02A;Vægt af uterus	0			
DVRA10;Legemshøjde (cm)	0			
DVRB02;Tobaksforbrug, antal gram tobak per dag	0			
DVRB51;Alkoholforbrug, antal genstande per uge	0			
DVRK01;Peroperativ blødning (ml)	0			
