# Supplementary material for: Mortality after paediatric emergency calls for patients with or without pre-existing comorbidity: a nationwide population based cohort study
Source: Scand J Trauma Resusc Emerg Med. 2024 May 28;32:48. doi: 10.1186/s13049-024-01212-2 (PMC11134704; doi:10.1186/s13049-024-01212-2)
Supplement: Supplementary file 2 — Supplementary Material 2 [file 13049_2024_1212_MOESM2_ESM.docx]

**Supplement 1**

**Methodology descriptions.** Data management and definitions.

**Table S1.** Primary hospital ICD-10 diagnosis for patients with a hospital visit following an emergency call.

**Table S2.** Numerical data for Figure 2: Reasons for emergency call within the three comorbidity subgroups.

**Table S3.** Characteristics of the patients who were lost to follow-up for the complete 30 days.

**Data Management and definitions**

**Setting**

Each of the five Danish health regions is responsible for operating publicly funded healthcare services including primary healthcare, hospitals, and emergency medical services (EMS). Each region operates an emergency medical dispatch centre staffed by health professionals who answer calls and manage the dispatch of both ground-level and helicopter EMS (HEMS) units according to the level of urgency (A-E, A being the most urgent). Annually, there are 1,975 emergency calls to 1-1-2 regarding children per 100,000 inhabitants aged ≤15 years. All dispatch centres use the criteria-based decision support tool Danish Index for Emergency Care. Ambulances and rapid response vehicles, manned by emergency medical technicians and paramedics, can be assisted by 24-hour operative mobile emergency care units (MECUs) and/or helicopters, both of which are staffed with an experienced physician specialised in anesthesiology and intensive care and a specially trained paramedic who carry advanced equipment and supplemental drugs. The number of physician-staffed MECUs varies between the five health regions, and they are engaged in about 20-25% of ambulance dispatches. Helicopter EMS are run by the Central Denmark Region and operated by Norwegian Air Ambulance. HEMS is primarily dispatched to the country’s semi-rural and rural areas including more than 65 inhabited islands.

**Data** **sources and linkage**

Prehospital data were collected by ground EMS personnel as described in the Methods section (Data sources and linkage), and we supplied the dataset with data from *Hemsfile*, the operational database of the national helicopter EMS. HEMS missions were missing from the year 2019, which could lead to underestimated incidence rates. The reported results have been calculated using data from all six years (2016-2021) as they were similar to results calculated using all six years except 2019.

In 2019, the Danish National Patient Register was extensively revised for administrative purposes. We chose to link data from the quality database to the Danish National Patient Register using the same conditions both before and after this revision. The registries were linked using the timestamp of the first ambulance that had arrived at a hospital (if more than one prehospital unit had been dispatched). If a patient had arrived by HEMS, this timestamp was used instead. If no timestamps of hospital arrival were available, the timestamp from the emergency call was used. If a hospital contact had been initiated within 5 hours from the chosen timestamp, these were linked and subsequent hospital contacts were compiled into one hospital stay if the start time of one hospital contact was within 24 hours from the previous contact’s end time.

**Variables**

’Primary hospital diagnosis’ is the first primary diagnosis assigned by a physician at the receiving hospital, regardless of sequential admissions at different departments or facilities during that hospital stay. If a primary diagnosis from the ICD-10 chapters 18 (*R: ‘Symptoms, signs and abnormal clinical and laboratory findings, not elsewhere classified’*) or 21 (*Z: ‘Factors influencing health status and contact with health services’*) was assigned, we then searched for a specific diagnosis during the entire hospital stay. If only R or Z diagnoses were present throughout the hospital stay, we searched for a specific diagnosis among the additional diagnostic codes. ’Last diagnosis’ was the last diagnosis assigned by a physician before death occurred. In most cases, this had been a hospital physician, but in case of no hospital contact, it was the diagnosis assigned by either the HEMS or MECU physician. A few diagnostic codes were ‘overruled’ by the second to last diagnosis, e.g., if the true last was ‘*Cardiac arrest, unspecified*’ and the second to last was ‘*Drowning and nonfatal submersion*’.

| **Table S1.** Primary hospital ICD-10 diagnosis for patients with a hospital visit following an emergency call. N = 93,081. | | | | | | | | | | | | | | |
| --- | --- | --- | --- | --- | --- | --- | --- | --- | --- | --- | --- | --- | --- | --- |
|  | **0-2 years** | | | | **3-7 years** | | | | **8-15 years** | | | | **Total** | |
|  | **Urgency level** | | | | **Urgency level** | | | | **Urgency level** | | | |  | |
| No. (%) | Lights and sirens | | **No** lights and sirens | | Lights and sirens | | **No** lights and sirens | | Lights and sirens | | **No** lights and sirens | |  | |
| **ICD-10 Chapter** |  | | | | | | | | | | | | | |
| AB: I - Certain infectious and parasitic diseases | 1,208 | 4.7 | 647 | 6.1 | 325 | 2.9 | 211 | 2.9 | 223 | 1.2 | 212 | 1.1 | 2,826 | 3.0 |
| CD: II - Neoplasms | 23 | 0.1 | ≤10 | - | 24 | 0.2 | ≤10 | - | 38 | 0.2 | 20 | 0.1 | 113 | 0.1 |
| D: III - Diseases of blood, blood-forming organs and immune system | 30 | 0.1 | ≤10 | - | ≤10 | - | ≤10 | - | 21 | 0.1 | 15 | 0.1 | 95 | 0.1 |
| E: IV - Endocrine, nutritional and metabolic diseases | 277 | 1.1 | 84 | 0.8 | 140 | 1.2 | 47 | 0.6 | 232 | 1.3 | 152 | 0.8 | 932 | 1.0 |
| F: V - Mental and behavioural disorders | 36 | 0.1 | ≤10 | - | 53 | 0.5 | 13 | 0.2 | 493 | 2.7 | 502 | 2.5 | 1,104 | 1.2 |
| G: VI - Diseases of the nervous system | 917 | 3.6 | 185 | 1.8 | 1,114 | 9.9 | 246 | 3.4 | 1,939 | 10.6 | 627 | 3.1 | 5,028 | 5.4 |
| H: VII - Diseases of the eye and adnexa | 27 | 0.1 | 21 | 0.2 | 24 | 0.2 | 16 | 0.2 | 26 | 0.1 | 21 | 0.1 | 135 | 0.1 |
| H: VIII - Diseases of the ear and mastoid process | 316 | 1.2 | 177 | 1.7 | 29 | 0.3 | 40 | 0.5 | 12 | 0.1 | 18 | 0.1 | 592 | 0.6 |
| I: IX - Diseases of the circulatory system | 136 | 0.5 | ≤10 | - | 84 | 0.7 | 20 | 0.3 | 219 | 1.2 | 98 | 0.5 | 567 | 0.6 |
| J: X - Diseases of the respiratory system | 4,070 | 15.8 | 1,513 | 14.4 | 2,234 | 19.8 | 706 | 9.7 | 804 | 4.4 | 341 | 1.7 | 9,668 | 10.4 |
| K: XI - Diseases of the digestive system | 337 | 1.3 | 189 | 1.8 | 86 | 0.8 | 101 | 1.4 | 165 | 0.9 | 259 | 1.3 | 1,137 | 1.2 |
| L: XII - Diseases of the skin and subcutaneous tissue | 176 | 0.7 | 62 | 0.6 | 79 | 0.7 | 40 | 0.5 | 72 | 0.4 | 39 | 0.2 | 468 | 0.5 |
| M: XIII - Diseases of the musculoskeletal system and connective tissue | 24 | 0.1 | 38 | 0.4 | 61 | 0.5 | 72 | 1.0 | 236 | 1.3 | 398 | 2.0 | 829 | 0.9 |
| N: XIV - Diseases of the genitourinary system | 144 | 0.6 | 50 | 0.5 | 34 | 0.3 | 42 | 0.6 | 82 | 0.5 | 155 | 0.8 | 507 | 0.5 |
| O: XV - Pregnancy, childbirth and the puerperium | ≤10 | - | ≤10 | - | 0 | 0.0 | 0 | 0.0 | ≤10 | - | ≤10 | - | 11 | 0.0 |
| P: XVI - Certain conditions originating in the perinatal period | 676 | 2.6 | 112 | 1.1 | ≤10 | - | ≤10 | - | 0 | 0.0 | 0 | 0.0 | 796 | 0.9 |
| **Table S1 (continued).** Primary hospital ICD-10 diagnosis for patients with a hospital visit following an emergency call. N = 93,081. | | | | | | | | | | | | | | |
|  | **0-2 years** | | | | **3-7 years** | | | | **8-15 years** | | | | **Total** | |
|  | **Urgency level** | | | | **Urgency level** | | | | **Urgency level** | | | |  | |
| No. (%) | Lights and sirens | | **No** lights and sirens | | Lights and sirens | | **No** lights and sirens | | Lights and sirens | | **No** lights and sirens | |  | |
| **ICD-10 Chapter** |  | | | | | | | | | | | | | |
| Q: XVII - Congenital malformations, deformations and chromosomal abnormalities | 151 | 0.6 | 36 | 0.3 | 65 | 0.6 | 11 | 0.2 | 52 | 0.3 | 19 | 0.1 | 334 | 0.4 |
| R: XVIII - Symptoms, signs and abnormal clinical and laboratory findings | 10,858 | 42.2 | 2,425 | 23.0 | 2,969 | 26.3 | 1,142 | 15.6 | 3,891 | 21.4 | 3,310 | 16.5 | 24,595 | 26.4 |
| ST: XIX - Injury, poisoning and other external causes | 3,785 | 14.7 | 3,680 | 35.0 | 2,991 | 26.5 | 4,123 | 56.5 | 8,122 | 44.6 | 12,665 | 63.2 | 35,366 | 38.0 |
| S-T14: XIX - Injuries | 1,819 | 7.1 | 2,686 | 25.5 | 2,018 | 17.9 | 3,604 | 49.4 | 6,519 | 35.8 | 11,498 | 57.4 | 28,144 | 30.2 |
| T15-T19: XIX - Foreign bodies | 633 | 2.5 | 395 | 3.8 | 213 | 1.9 | 233 | 3.2 | 101 | 0.6 | 68 | 0.3 | 1,643 | 1.8 |
| T20-T35: XIX - Burns, corrosions and frostbite | 492 | 1.9 | 240 | 2.3 | 136 | 1.2 | 92 | 1.3 | 171 | 0.9 | 97 | 0.5 | 1,228 | 1.3 |
| T36-T65: XIX - Poisoning | 335 | 1.3 | 223 | 2.1 | 172 | 1.5 | 99 | 1.4 | 792 | 4.3 | 816 | 4.1 | 2,437 | 2.6 |
| T66-T78: XIX - Effects of external causes | 453 | 1.8 | 117 | 1.1 | 303 | 2.7 | 61 | 0.8 | 316 | 1.7 | 110 | 0.5 | 1,360 | 1.5 |
| T79: XIX - Early complications of trauma | 0 | 0.0 | 0 | 0.0 | 0 | 0.0 | 0 | 0.0 | 0 | 0.0 | 0 | 0.0 | 0 | 0.0 |
| T80-T88: XIX - Complications of surgical and medical care | 50 | 0.2 | 18 | 0.2 | 143 | 1.3 | 34 | 0.5 | 219 | 1.2 | 72 | 0.4 | 536 | 0.6 |
| T90-T98: XIX - Sequelae of injuries, poisoning and external causes | ≤10 | - | ≤10 | - | ≤10 | - | 0 | 0.0 | ≤10 | - | ≤10 | - | 18 | 0.0 |
| VXY: XX - External causes of morbidity and mortality | 0 | 0.0 | 0 | 0.0 | 0 | 0.0 | 0 | 0.0 | ≤10 | - | ≤10 | - | 9 | 0.0 |
| Z: XXI - Contact with health services | 2,524 | 9.8 | 1,278 | 12.1 | 942 | 8.4 | 452 | 6.2 | 1,587 | 8.7 | 1,186 | 5.9 | 7,969 | 8.6 |
|  |  |  |  |  |  |  |  |  |  |  |  |  |  |  |
| Total | 25,720 | 100 | 10,526 | 100 | 11,269 | 100 | 7,301 | 100 | 18,220 | 100 | 20,045 | 100 | 93,081 | 100 |
|  | | | | | | | | | | | | | | |
| No missing data.  *ICD-10* International Statistical Classification of Diseases and Related Health Problems, Tenth Revision.  Chapter XIX ‘Injury, poisoning and certain other consequences of external causes (S00-T98)’ is presented at both main chapter level and subchapter level. | | | | | | | | | | | | | | |

| **Table S2.** Numerical data for Figure 2: Reasons for emergency call within the three comorbidity subgroups. | | | | | | |
| --- | --- | --- | --- | --- | --- | --- |
|  | | | | | | |
| No. | Non-survivors | | | Survivors | | |
|  | n = 255 | | | n = 76,701 | | |
|  | **Comorbidity** | | | | | |
|  | None | Comorbidity | Severe chronic comorbidity | None | Comorbidity | Severe chronic comorbidity |
| **Dispatch criteria** | | | | | | |
| Trauma | 22 | <10 | <10 | 14,367 | 6,321 | 193 |
| Paediatric out-of-hospital cardiac arrest | 56 | 17 | 20 | 16 | 12 | <10 |
| Medical symptoms | 35 | 33 | 41 | 30,527 | 19,922 | 1,251 |
| Suspected death | <10 | <10 | <10 | <10 | <10 | <10 |
| Missing criteria | <10 | <10 | <10 | 2,530 | 1,455 | 100 |
|  | | | | | | |
| Total | 121 | 64 | 70 | 47,442 | 27,714 | 1,545 |
|  | | | | | | |
| Survivors and non-survivors refer to death within 7 days of the last emergency call. | | | | | | |

| **Table S3.** Characteristics of the patients who were lost to follow-up for the complete 30 days. | | |
| --- | --- | --- |
|  | | |
|  | **Lost to follow-up** | **Study population** |
| No. (%) | **N = 1,143** | **N = 76,956** |
| **Sex** |  |  |
| Female | 514 (45.0) | 35,522 (46.2) |
| Male | 629 (55.0) | 41,434 (53.8) |
| **Entry year** |  |  |
| 2016 | 201 (17.6) | 9,817 (12.8) |
| 2017 | 244 (21.4) | 12,061 (15.7) |
| 2018 | 223 (19.5) | 12,372 (16.1) |
| 2019 | 191 (16.7) | 13,063 (17.0) |
| 2020 | 152 (13.3) | 12,875 (16.7) |
| 2021 | 132 (11.6) | 16,768 (21.8) |
| **Dispatch criteria** |  |  |
| Trauma | 256 (22.4) | 20,913 (27.2) |
| Paediatric out-of-hospital cardiac arrest | 0 (0.0) | 122 (0.2) |
| Medical symptoms | 797 (69.7) | 51,809 (67.3) |
| Suspected death | 0 (0.0) | 21 (0.0) |
| Missing criteria | 90 (7.9) | 4,091 (5.3) |
|  | | |
